# Supplementary material for: Switchable chemoselective aryne reactions between nucleophiles and pericyclic reaction partners using either 3-methoxybenzyne or 3-silylbenzyne
Source: Nat Commun. 2024 Apr 30;15:3665. doi: 10.1038/s41467-024-47952-8 (PMC11063064; doi:10.1038/s41467-024-47952-8)
Supplement: Supplementary file 1 — Supplementary Information [file 41467_2024_47952_MOESM1_ESM.pdf]

SUPPLEMENTARY INFORMATION

Switchable Chemoselective Aryne Reactions between Nucleophiles and Pericyclic Reaction Partners using either 3-Methoxybenzyne or 3-Silylbenzyne

Hongcheng Tan,[1] Shuxin Yu,[1] Xiaoling Yuan,[1] Liyuan Chen,[1] Chunhui Shan,[2] Jiarong Shi,[1] Yang Li[1]*

[1]School of Chemistry and Chemical Engineering, Chongqing University, 174 Shazheng Street, Chongqing, P. R. China, 400030
[2]College of Chemistry, Chongqing Normal University, Chongqing, P. R. China 401331
*Correspondence to: y.li@cqu.edu.cn

Table of Contents

1. Supplementary Notes
2. Supplementary Methods
   2.1 Preparation of aryne precursors **1**
   2.2 Preparation of substrates
   2.3 Intermolecular aryne trapping reactions with various arynophiles
   2.4 Reactions of aryne precursors with substrates
   2.5 Asymmetric synthesis of dihydrexidine
   2.6 Reactions of aryne precursors with *n*-$BuNH_2$ and *t*-$BuNH_2$
   2.7 Crystal data and structure refinement for 10b and 34
   2.8 DFT calculations
3. Supplementary Figures for ^1H and ^{13}C NMR Spectra
4. Supplementary References

1. Supplementary Notes

All reagents were obtained from Adamas, Aladin, Accela, or Acros and used without further purification unless otherwise noted. To prevent moisture, CsF, KF, tetrabutylammonium difluorotriphenylsilicate (TBAT), Cs_2CO_3, K_2CO_3, and 18-c-6 were stored and used under inert atmosphere in Glovebox. The products were purified by column chromatography with Huanghai Silica Gel 50-75 um, ultrapure silica gel. ^{1}H and ^{13}C spectra were recorded in $CDCl_3$ (with 0.03% Me_4Si), DMSO-*d*6 using a Varian 400 spectrometer. Chemical shifts (δ) are reported in ppm downfield from Me_4Si (δ 0.00 for ^{1}H NMR in $CDCl_3$, DMSO-*d*6) or the solvent peak (δ 7.26 for ^{1}H NMR in $CDCl_3$, δ 2.50 for ^{1}H NMR in DMSO-*d*6, δ 77.23 for ^{13}C NMR in $CDCl_3$, δ 39.50 for ^{13}C NMR in DMSO-*d*6) as an internal reference with coupling constants (*J*) in hertz (Hz). Data are reported as follows: s = singlet, d = doublet, t = triplet, q = quartet, m = multiplet, brs = broad singlet. IR spectra were measured on a Nicolet iS50 FT-IR spectrometer using KBr plates. The high-resolution mass spectra (HRMS) were recorded on waters G2-XS QTOF (ESI). Single-crystal X-ray diffraction (XRD) data was collected on a Super Nova-CCD using graphite-monochromated Mo Kα radiation (λ = 0.71073 Å) at 298 K.

2. Supplementary Methods

2.1 Preparation of aryne precursors 1

General procedure for the preparation of aryne precursors 1 (Procedure A)

A solution of substituted *o*-bromophenol (5.0 mmol, 1.0 equiv) and hexamethyl disilylamine (HMDS) (2.09 mL, 10.0 mmol, 2.0 equiv) in THF (10 mL) was heated under reflux overnight. The resulting solution was concentrated directly on rotary evaporator and further dried under vacuum for one hour. The resulting crude material was dissolved in anhydrous THF (30 mL) and cooled to -78 °C under inert atmosphere. *n*-BuLi (2.5 M in hexanes, 3.0 mL, 7.5 mmol, 1.5 equiv) was added dropwise. After 10 minutes, triflic anhydride (Tf_2O) (1.26 mL, 7.5 mmol, 1.5 equiv) was added. After 10 minutes, 10% aq $NaHCO_3$ (10 mL) was added. The resulting mixture was extracted with EtOAc (30 mL x 3). The combined organic layers were washed with brine (20 mL), dried over Na_2SO_4, filtered, and concentrated. Flash column chromatography with pet ether afforded aryne precursors **1**.

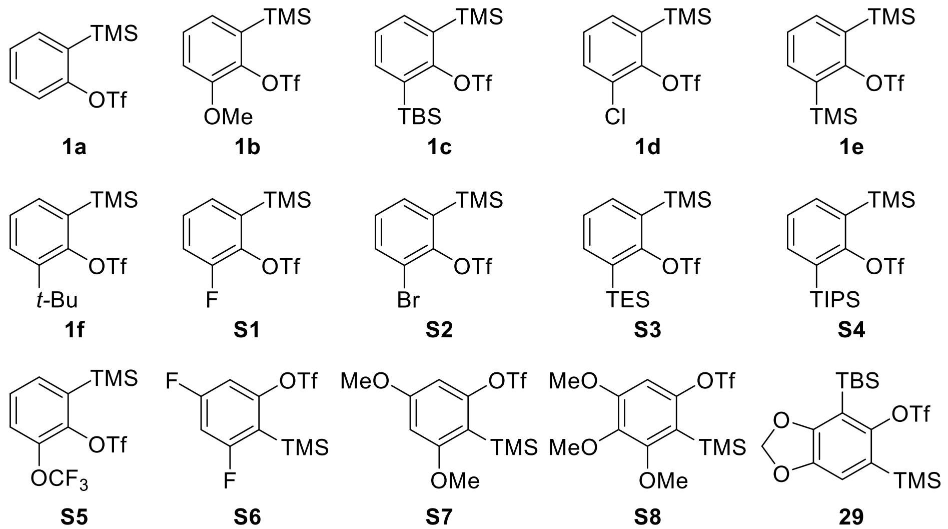

1a 1b 1c 1d 1e

1f S1 S2 S3 S4

S5 S6 S7 S8 29

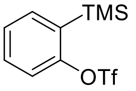

1a

Compound 1a: The preparation of compound **1a** was followed by procedure A, and the ^{1}H NMR of **1a** is identical with that reported in literature.[1] ^{1}H NMR (400 MHz, $CDCl_3$) δ 7.54 (dd, J_1 = 7.6, J_2 = 2.0 Hz, 1H), 7.44 (t, J = 7.6 Hz, 1H), 7.34 (dd, J_1 = 6.8, J_2 = 4.4 Hz, 2H), 0.37 (s, 9H) ppm.

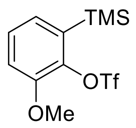

1b

Compound 1b: The preparation of compound **1b** was followed by procedure A, and the ^{1}H NMR of **1b** is identical with that reported in literature.[2] ^{1}H NMR (400 MHz, $CDCl_3$) δ 7.30 (t, J = 7.6 Hz, 1H), 7.07 (d, J = 7.6 Hz, 1H), 7.03 (d, J = 8.0 Hz, 1H), 3.86 (s, 3H), 0.39 (s, 9H) ppm.

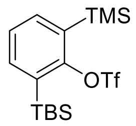

1c

Compound 1c: The preparation of compound **1c** was followed by procedure A, and the ^{1}H NMR of **1c** is identical with that reported in literature.[3] ^{1}H NMR (400 MHz, $CDCl_3$) δ 7.58 (d, J = 7.2 Hz, 2H), 7.35 (t, J = 7.2 Hz, 1H), 0.79 (s, 9H), 0.39 (s, 6H), 0.35 (s, 9H) ppm.

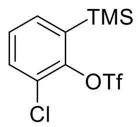

1d

Compound 1d: The preparation of compound **1d** was followed by procedure A, and the ^{1}H NMR of **1d** is identical with that reported in literature.[4] ^{1}H NMR (400 MHz, $CDCl_3$) δ 7.50 (dd, J_1 = 7.6, J_2 = 1.6 Hz, 1H), 7.46 (dd, J_1 = 7.6, J_2 = 1.6 Hz, 1H), 7.31 (t, J = 7.6 Hz, 1H), 0.41 (s, 9H) ppm.

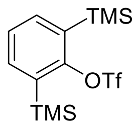

1e

Compound 1e: The preparation of compound **1e** was followed by procedure A, and the ^{1}H NMR of **1e** is identical with that reported in literature.[3] ^{1}H NMR (400 MHz, $CDCl_3$) δ 7.58 (d, J = 7.2 Hz, 2H), 7.36 (t, J = 7.2 Hz, 1H), 0.36 (s, 18H) ppm.

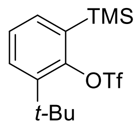

1f

Compound 1f: The preparation of compound **1f** was followed by procedure A, and the ^{1}H NMR of **1f** is identical with that reported in literature.[5] ^{1}H NMR (400 MHz, $CDCl_3$) δ 7.54 (dd, J_1 = 7.6, J_2 = 2.0 Hz, 1H), 7.40 (dd, J_1 = 7.2, J_2 = 2.0 Hz, 1H), 7.29 (t, J = 7.6 Hz, 1H), 1.43 (s, 9H), 0.36 (s, 9H) ppm.

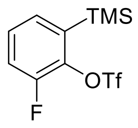

S1

Compound S1: The preparation of compound **S1** was followed by procedure A, and the ^{1}H NMR of **S1** is identical with that reported in literature.[4] ^{1}H NMR (400 MHz, $CDCl_3$) δ 7.36-7.18 (m, 3H), 0.40 (s, 9H) ppm.

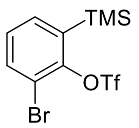

S2

Compound S2: The preparation of compound **S2** was followed by procedure A, and the ^{1}H NMR of **S2** is identical with that reported in literature.[6] ^{1}H NMR (400 MHz, $CDCl_3$) δ 7.67 (dd, J_1 = 8.0, J_2 = 2.0 Hz, 1H), 7.51 (dd, J_1 = 7.2, J_2 = 1.2 Hz, 1H), 7.23 (t, J = 7.6 Hz, 1H), 0.40 (s, 9H) ppm.

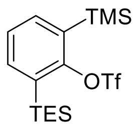

S3

Compound S3: The preparation of compound **S3** was followed by procedure A, and the ^{1}H NMR of **S3** is identical with that reported in literature.[3] ^{1}H NMR (400 MHz, $CDCl_3$) δ 7.58 (dd, J_1 = 7.2, J_2 = 2.0 Hz, 1H), 7.54 (dd, J_1 = 7.2, J_2 = 1.2 Hz, 1H), 7.35 (t, J = 7.6 Hz, 1H), 0.98-0.85 (m, 15H), 0.36 (s, 9H) ppm.

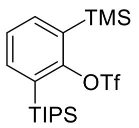

S4

Compound S4: The preparation of compound **S4** was followed by procedure A, and the ^{1}H NMR of **S4** is identical with that reported in literature.[3] ^{1}H NMR (400 MHz, $CDCl_3$) δ 7.39 (d, J = 2.0 Hz, 1H), 7.37 (d, J = 2.0 Hz, 1H), 6.94 (t, J = 7.2 Hz, 1H), 1.51-1.41 (m, 3H), 1.11 (d, J = 7.2 Hz, 18H), 0.32 (s, 9H) ppm.

Preparation of aryne precursor S5:

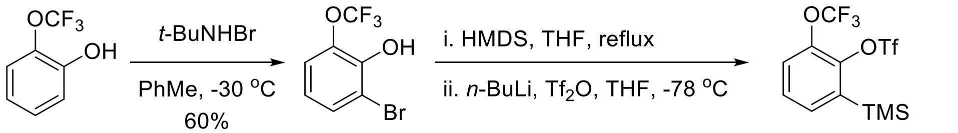

S5, 52% in two steps

2-Bromo-6-(trifluoromethoxy)phenol: To a solution of 2-(trifluoromethoxy)phenol (326 mg, 1.83 mmol, 1.0 equiv) in anhydrous toluene (10 mL) at -30 °C was slowly added *N*-bromo-2-methylpropan-2-amine (*t*-BuNHBr) (0.5M in toluene, 3.7 mL, 1.83 mmol, 1.0 equiv). After 30 minutes, 10% aq $NaHCO_3$ (20 mL) was added. The resulting mixture was extracted with EtOAc (10 mL x 3). The combined organic layers were washed with brine (20 mL), dried over Na_2SO_4, filtered, and concentrated. Flash column chromatography (pet ether: EtOAc = 40: 1) afforded 282 mg (60%) of 2-bromo-6-(trifluoromethoxy)phenol as a colorless oil. ^{1}H NMR (400 MHz, $CDCl_3$) δ 7.43 (dd, J_1 = 8.4, J_2 = 1.6 Hz, 1H), 7.23-7.18 (m, 1H), 6.83 (t, J = 8.4 Hz, 1H), 5.16 (brs, 1H) ppm; ^{13}C NMR (100 MHz, $CDCl_3$) δ 146.0, 137.0, 131.1, 122.0, 121.3, 120.8 (q, J = 257.6 Hz), 111.3 ppm; IR (thin film) 3418, 2927, 1660, 1557, 1384, 1245, 1141, 1094, 987, 861, 729, 596, 543, 478 (cm^{-1}); HRMS (ESI) m/z: calcd for $[C_7H_3BrF_3O_2]^-$ 254.9274; found 254.9267.

Compound S5: A solution of 2-bromo-6-(trifluoromethoxy)phenol (282 mg, 1.1 mmol, 1.0 equiv) and hexamethyl disilylamine (HMDS) (0.46 mL, 2.2 mmol, 2.0 equiv) in anhydrous THF (5 mL) was heated under reflux overnight. The solution was then concentrated directly on rotary evaporator and further dried under vacuum. The resulting crude material was dissolved in anhydrous THF (10 mL) and cooled to -78 °C under inert atmosphere. *n*-butyllithium (*n*-BuLi) (2.5 M in hexanes, 0.66 mL, 1.65 mmol, 1.5 equiv) was added dropwise. After 20 minutes, trifluoromethanesulfonic anhydride (Tf_2O) (0.28 mL, 1.65 mmol, 1.5 equiv) was added. After 10 minutes, 10% aq $NaHCO_3$ (20 mL) was added. The resulting mixture was extracted with pet ether (10 mL x 3). The combined organic layers were washed with brine (20 mL), dried

over Na_2SO_4, filtered, and concentrated. Flash column chromatography (pet ether) afforded 219 mg (52% in two steps) of **S5** as a colorless oil. ^{1}H NMR (400 MHz, $CDCl_3$) δ 7.48 (q, *J* = 4.4 Hz, 1H), 7.41 (d, *J* = 5.2 Hz, 2H), 0.43 (s, 9H) ppm; ^{13}C NMR (100 MHz, $CDCl_3$) δ 144.3, 140.7, 138.0, 134.2, 129.2, 123.1, 120.6 (q, *J* = 258.6 Hz), 118.9 (q, *J* = 318.5 Hz), -0.2 ppm; IR (thin film) 3418, 2927, 1660, 1557, 1384, 1245, 1141, 1094, 987, 861, 729, 596, 543, 478 (cm^{-1}); HRMS (ESI) m/z: calcd for $[C_{11}H_{13}F_6O_4SSi]^+$ 383.0203; found 383.0201.

Preparation of aryne precursors S6:

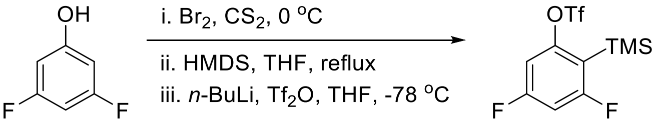

S6, 35% in three steps

Compound S6: To a solution of commercially available 3,5-difluorophenol (1.0 g, 7.7 mmol, 1.0 equiv) in CS_2 (10 mL) at 0 °C was slowly added a solution of Br_2 (0.39 mL, 7.7 mmol, 1.0 equiv) in CS_2 (4 mL) and the resulting mixture was allowed to warm to room temperature. After stirring for two hours, 10% aq $Na_2S_2O_3$ (20 mL) was added. The resulting mixture was extracted with diethyl ether (Et_2O) (10 mL x 3). The combined organic layers were washed with brine (30 mL), dried over Na_2SO_4, filtered, and concentrated to afford a crude pale-yellow oil, which was used directly in next step without further purification.
A solution of 2-bromo-3,5-difluorophenol and HMDS (3.2 mL, 15.4 mmol, 2.0 equiv) in anhydrous THF (5 mL) was heated under reflux overnight. The solution was then concentrated directly on rotary evaporator and further dried under vacuum. The resulting crude material was dissolved in anhydrous THF (30 mL) and cooled to -78 °C under inert atmosphere. *n*-BuLi (2.5 M in hexanes, 4.6 mL, 11.6 mmol, 1.5 equiv) was added dropwise. After 20 minutes, Tf_2O (2.0 mL, 11.6 mmol, 1.5 equiv) was added. After 10 minutes, 10% aq $NaHCO_3$ (20 mL) was added. The resulting mixture was extracted with pet ether (30 mL x 3). The combined organic layers were washed with brine (20 mL), dried over Na_2SO_4, filtered, and concentrated. Flash column chromatography (pet ether) afforded 901 mg (35% in three steps) of **S6** as a colorless oil. ^{1}H NMR (400 MHz, $CDCl_3$) δ 6.96 (d, *J* = 8.8 Hz, 1H), 6.79 (td, J_1 = 8.8, J_2 = 2.0 Hz, 1H), 0.41 (d, *J* = 2.0 Hz, 9H) ppm; ^{13}C NMR (100 MHz, $CDCl_3$) δ 167.8 (dd, J_1 = 244.8, J_2 = 13.7 Hz), 164.0 (dd, J_1 = 250.9, J_2 = 15.3 Hz), 154.4 (dd, J_1 = 18.4, J_2 = 13.1 Hz), 118.7 (q, *J* = 318.7 Hz), 116.3 (dd, J_1 = 34.7, J_2 = 4.2 Hz), 105.4-105.1 (m), 104.0 (dd, J_1 = 31.5, J_2 = 23.7 Hz), 0.77-0.19 (m) ppm; IR (thin film) 2955, 2914, 2848, 1655, 1637, 1612, 1467, 1424, 1384, 1259, 1213, 1094, 848, 816, 802 (cm^{-1}); HRMS (ESI) m/z: calcd for $[C_{10}H_{10}F_5O_3SSi]^-$ 333.0046; found 333.0038.

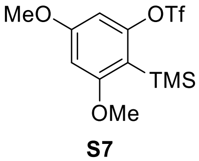

S7

Compound S7: The preparation of compound **S7** was followed by procedure A, and the ^{1}H NMR of **S7** is identical with that reported in literature.[7] ^{1}H NMR (400 MHz, $CDCl_3$) δ 6.49 (s, 1H), 6.38 (s, 1H), 3.81 (s, 3H), 3.79 (s, 3H), 0.32 (s, 9H) ppm.

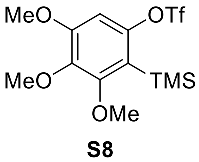

S8

Compound S8: The preparation of compound **S8** was followed by procedure A, and the ^{1}H NMR of **S8** is identical with that reported in literature.[7] ^{1}H NMR (400 MHz, $CDCl_3$) δ 6.65 (s, 1H), 3.91 (s, 3H), 3.85 (s, 3H), 3.83 (s, 3H), 0.35 (s, 9H) ppm.

Preparation of aryne precursors 29:

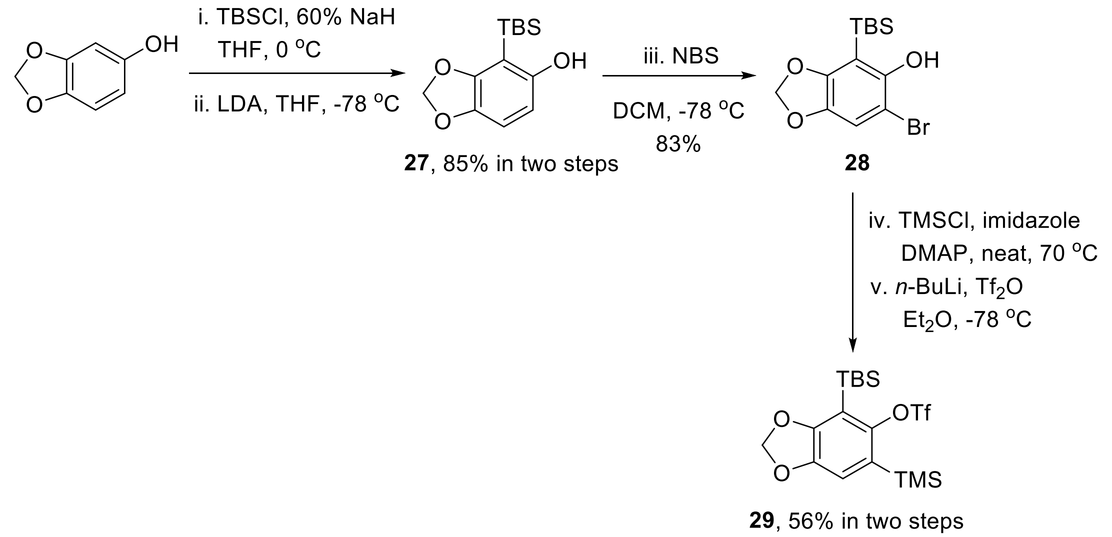

Compound 27: To a solution of sesamol (2.76 g, 20.0 mmol, 1.0 equiv) in anhydrous THF (40 mL) at 0 °C was slowly added NaH (60% in mineral oil, 880 mg, 22.0 mmol, 1.1 equiv). After 15 minutes, *tert*-butylchlorodimethylsilane (TBSCl) (3.92 g, 26.0 mmol, 1.3 equiv) was added and the resulting mixture was allowed to warm to room temperature. After two hours, the resulting mixture was quenched with saturated aqueous NH_4Cl (10 mL). The separated aqueous layer was extracted with EtOAc (20 mL x 3), and the combined organic layers were washed with brine (20 mL), dried over Na_2SO_4, filtered, and concentrated to afford a crude colorless oil, which was used directly in next step without further purification.

To a solution of the above crude colorless oil in anhydrous THF (10 mL) at -78 °C under inert atmosphere was added dropwise a solution of lithium diisopropylamide (LDA) (2.5 M in THF, 40.0 mL, 100 mmol, 5.0 equiv). The resulting mixture was stirred at -78 °C for 40 minutes, which was allowed to warm to room temperature for two hours. It was quenched with saturated aqueous NH_4Cl (10 mL). The separated aqueous layer was extracted with EtOAc (30 mL x 3), and the combined organic layers were washed with brine (50 mL), dried over Na_2SO_4, filtered, and concentrated. Flash column chromatography (pet ether: EtOAc = 20: 1) afforded 4.29 g (85% in two steps) of compound **27** as a brown solid. Mp: 62-64 °C; ^{1}H NMR (400 MHz, $CDCl_3$) δ 6.64 (d, J = 8.0 Hz, 1H), 6.14 (d, J = 8.4 Hz, 1H), 5.83 (s, 2H), 4.53 (s, 1H), 0.92 (s, 9H), 0.36 (s, 6H) ppm; ^{13}C NMR (100 MHz, $CDCl_3$) δ 155.6, 153.8, 140.4, 109.3, 106.5, 105.7, 100.3, 26.8, 18.3, -3.4 ppm; IR (thin film) 3531, 2927, 2855, 1628, 1470, 1419, 1246, 1137, 1051, 962, 823, 768, 680, 628 (cm^{-1}); HRMS (ESI) m/z: calcd for $[C_{13}H_{20}NaO_3Si]^+$ 275.1074; found 275.1082.

Compound 28: To a solution of compound **27** (4.04 g, 16.0 mmol, 1.0 equiv) in anhydrous DCM (40 mL) at -78 °C was added *N*-bromosuccinimide (NBS) (3.13 g, 17.6 mmol, 1.1 equiv). After 20 minutes, the resulting mixture was quenched with 10% aqueous $Na_2S_2O_3$ (10 mL), which was extracted with DCM (30 mL x 3). The combined organic layers were washed with brine (50 mL), dried over Na_2SO_4, filtered, and concentrated. Flash column chromatography with pet ether afforded 4.40 g (83%) of compound **28** as a white solid. Mp: 61-63 °C; ^{1}H NMR (400 MHz, $CDCl_3$) δ 6.90 (s, 1H), 5.85 (s, 2H), 5.37 (s, 1H), 0.92 (s, 9H), 0.35 (s, 6H) ppm; ^{13}C NMR (100 MHz, $CDCl_3$) δ 153.6, 151.2, 140.8, 111.8, 106.3, 100.8, 99.8, 26.8, 18.3, -3.3 ppm; IR (thin film) 3468, 3093, 2956, 2925, 2854, 1617, 1493, 1467, 1417, 1271, 1049, 946, 843, 768, 735, 678 (cm^{-1}); HRMS (ESI) m/z: calcd for $[C_{13}H_{20}BrO_3Si]^+$ 331.0360; found 331.0372.

Compound 29: To a flask containing compound **28** (3.30 g, 10.0 mmol, 1.0 equiv) was added sequentially imidazole (1.02 g, 15.0 mmol, 1.5 equiv), *N*, *N*-dimethylpyridin-4-amine (DMAP) (112.2 mg, 1.0 mmol, 0.1 equiv), and chlorotrimethylsilane (TMSCl) (1.90 mL, 15.0 mmol, 1.5 equiv). After stirring at 70 °C for two hours, the milky solution was diluted with pet ether (5 mL) and quenched with saturated aqueous NH_4Cl (10 mL). The separated aqueous layer was extracted with pet ether (20 mL x 3), and the combined organic layers were washed with brine (40 mL), dried over Na_2SO_4, filtered, and concentrated to afford a

crude colorless oil, which was used directly in next step without further purification.
To a solution of the above crude colorless oil in anhydrous Et_2O (40 mL) at -78 °C under inert atmosphere was added dropwise *n*-BuLi (2.5 M in hexanes, 6.0 mL, 15.0 mmol, 1.5 equiv). After 15 minutes, Tf_2O (2.52 mL, 15.0 mmol, 1.5 equiv) was added. After 10 minutes, the resulting mixture was quenched with saturated aqueous NH_4Cl (10 mL). The separated aqueous layer was extracted with pet ether (20 mL x 3), and the combined organic layers were washed with brine (50 mL), dried over Na_2SO_4, filtered, and concentrated. Flash column chromatography with pet ether afforded 2.56 g (56% in two steps) of compound **29** as a white solid. Mp: 92-94 °C; ^{1}H NMR (400 MHz, $CDCl_3$) δ 6.92 (s, 1H), 5.97 (s, 2H), 0.86 (s, 9H), 0.39 (s, 6H), 0.31 (s, 9H) ppm; ^{13}C NMR (100 MHz, $CDCl_3$) δ 155.1, 147.9, 146.1, 128.5, 118.8 (q, J = 318.9 Hz), 115.2, 114.6, 101.3, 27.6, 18.6, 0.8, -2.7 ppm; IR (thin film) 3572, 2955, 2859, 1611, 1573, 1502, 1471, 1396, 1361, 1216, 1139, 1049, 974, 890, 845, 769, 681, 619, 509 (cm^{-1}); HRMS (ESI) m/z: calcd for $[C_{17}H_{28}F_3O_5SSi_2]^+$ 457.1143; found 457.1157.

2.2 Preparation of substrates

Preparation of compound sub-1:

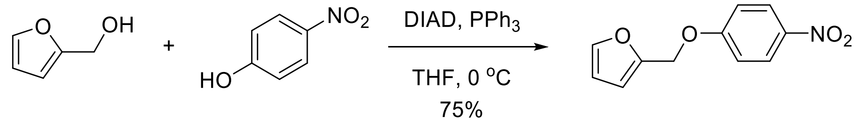

2-((4-Nitrophenoxy)methyl)furan: To a solution of triphenylphosphine (PPh_3) (1.70 g, 6.5 mmol, 1.3 equiv) in anhydrous THF (10 mL) at 0 °C under inert atmosphere was added dropwise diisopropyl azodicarboxylate (DIAD) (1.28 mL, 6.5 mmol, 1.3 equiv). After stirring for 10 minutes at 0 °C, a solution of furan-2-ylmethanol (0.43 mL, 5.0 mmol, 1.0 equiv) and 4-nitrophenol (904 mg, 6.5 mmol, 1.3 equiv) in THF (10 mL) was added slowly. The resulting mixture was allowed to warm to room temperature. Upon completion of the reaction, the resulting mixture was quenched with water (10 mL). The separated aqueous layer was extracted with EtOAc (20 mL x 3), and the combined organic layers were washed with brine (20 mL), dried over Na_2SO_4, filtered, and concentrated. Flash column chromatography (pet ether: EtOAc = 15: 1) afforded 822 mg (75%) of 2-((4-nitrophenoxy)methyl)furan as a white solid. Mp: 91-93 °C; ^{1}H NMR (400 MHz, $CDCl_3$) δ 8.21 (d, J = 9.2 Hz, 2H), 7.47 (d, J = 1.2 Hz, 1H), 7.05 (d, J = 9.2 Hz, 2H), 6.49 (d, J = 3.2 Hz, 1H), 6.41 (dd, J_1 = 3.2, J_2 = 1.2 Hz, 1H), 5.10 (s, 2H) ppm; ^{13}C NMR (100 MHz, $CDCl_3$) δ 163.5, 149.1, 143.8, 142.1, 126.1, 115.0, 111.1, 110.9, 63.0 ppm; IR (thin film) 3118, 3079, 2925, 2853, 2654, 2438, 1738, 1598, 1503, 1378, 1343, 1301, 1260, 1177, 1111, 985, 928, 843, 812, 735, 687, 627, 597, 500 (cm^{-1}); HRMS (ESI) m/z: calcd for $[C_{11}H_9NNaO_4]^+$ 242.0424; found 242.0422.

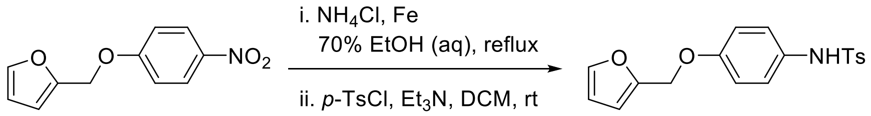

sub-**1**, 73% in two steps

Compound sub-1: A mixture of 2-((4-nitrophenoxy)methyl)furan (500 mg, 2.28 mmol, 1.0 equiv), iron powder (382 mg, 6.84 mmol, 3.0 equiv), and NH_4Cl (1.10 g, 20.53 mmol, 9.0 equiv) in ethanol (70%, 15 mL) was heated to reflux for one hour. The resulting mixture was cooled to room temperature, which was then filtered through a short pad of celite. The filtrate was dried over anhydrous Na_2SO_4, filtered, and concentrated to afford a crude pale-yellow oil, which was used directly in next step without further purification.
To the above crude pale-yellow oil in anhydrous DCM (10 mL) at room temperature under inert atmosphere was added triethylamine (Et_3N) (0.63 mL, 4.56 mmol, 2.0 equiv) and *p*-toluenesulfonyl chloride (*p*-TsCl) (652 mg, 3.42 mmol, 1.5 equiv). The resulting mixture was stirred at room temperature overnight, which was then quenched with water (10 mL). The separated aqueous layer was extracted with DCM (20 mL x 3), and the combined organic layers were washed with brine (20 mL), dried over Na_2SO_4, filtered, and concentrated. Flash column chromatography (pet ether: EtOAc = 5: 1) afforded 572 mg (73% in two steps) of compound sub-**1** as a white solid. Mp: 139-141 °C; ^{1}H NMR (400 MHz, $CDCl_3$) δ 7.57

(d, J = 8.4 Hz, 2H), 7.44 (dd, J_1 = 2.0, J_2 = 0.8 Hz, 1H), 7.21 (d, J = 8.0 Hz, 2H), 6.97 (d, J = 9.2 Hz, 2H), 6.84 (d, J = 8.8 Hz, 2H), 6.41 (d, J = 3.2 Hz, 1H), 6.39-6.35 (m, 2H), 4.94 (s, 2H), 2.39 (s, 3H) ppm; ^{13}C NMR (100 MHz, $CDCl_3$) δ 156.9, 150.1, 143.9, 143.4, 136.2, 129.8, 129.7, 127.5, 125.5, 115.7, 110.7, 110.3, 62.9, 21.7 ppm; IR (thin film) 3156, 3063, 2927, 2870, 1926, 1884, 1597, 1507, 1462, 1398, 1330, 1299, 1239, 1155, 1092, 1000, 923, 833, 751, 680, 556, 537, 522 (cm^{-1}); HRMS (ESI) m/z: calcd for $[C_{18}H_{17}NNaO_4S]^+$ 366.0770; found 366.0783.

Preparation of compound sub-2:

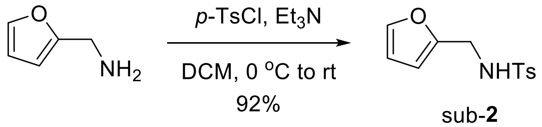

sub-**2**

Compound sub-2: To furfurylamine (0.45 mL, 5.0 mmol, 1.0 equiv) in anhydrous DCM (20 mL) at 0 °C under inert atmosphere was added Et_3N (1.39 mL, 10.0 mmol, 2.0 equiv) and *p*-TsCl (1.43 g, 7.5 mmol, 1.5 equiv). The resulting solution was stirred at room temperature overnight, which was then quenched with water (10 mL). The separated aqueous layer was extracted with DCM (20 mL x 3), and the combined organic layers were washed with brine (20 mL), dried over Na_2SO_4, filtered, and concentrated. Flash column chromatography (pet ether: EtOAc = 5: 1) afforded 1.16 g (92%) of compound sub-**2** as a white solid, the ^{1}H NMR of which is identical with that reported in literature.[8] ^{1}H NMR (400 MHz, $CDCl_3$) δ 7.72 (d, J = 8.4 Hz, 2H), 7.28 (d, J = 8.0 Hz, 2H), 7.25-7.23 (m, 1H), 6.22 (dd, J_1 = 3.2, J_2 = 2.0 Hz, 1H), 6.09 (d, J = 3.2 Hz, 1H), 4.69 (t, J = 5.2 Hz, 1H), 4.17 (d, J = 6.0 Hz, 2H), 2.42 (s, 3H) ppm.

Preparation of compound sub-3:

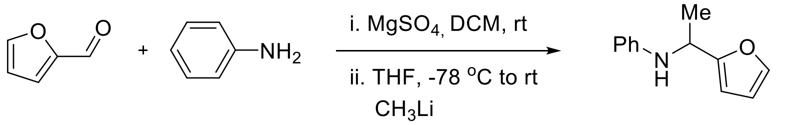

sub-**3**, 58% in two steps

Compound sub-3: To a solution of furan-2-carbaldehyde (0.41 mL, 5.0 mmol, 1.0 equiv) and aniline (0.46 mL, 5.0 mmol, 1.0 equiv) in anhydrous DCM (10 mL) was added anhydrous $MgSO_4$ (300 mg, 2.5 mmol, 0.5 equiv). A $CaCl_2$ drying tube was fitted to the flask and the resulting mixture was stirred at room temperature overnight, which was then filtered through a short pad of celite. The filtrate was dried over anhydrous Na_2SO_4, filtered, and concentrated to afford a crude mixture, which was used directly in next step without further purification.

To a stirred solution of the above crude mixture in anhydrous THF (20 mL) at -78 °C under inert atmosphere was added dropwise CH_3Li (1.6 M in diethoxymethane, 6.3 mL, 10.0 mmol, 2.0 equiv). Upon completion of the reaction, it was quenched with saturated aqueous NH_4Cl (10 mL). The separated aqueous layer was extracted with EtOAc (20 mL x 3), and the combined organic layers were washed with brine (20 mL), dried over Na_2SO_4, filtered, and concentrated. Flash column chromatography (pet ether: EtOAc = 10: 1) afforded 543.0 mg (58% in two steps) of compound sub-**3** as a yellow oil. ^{1}H NMR (400 MHz, $CDCl_3$) δ 7.38-7.33 (m, 1H), 7.18 (t, J = 8.4 Hz, 2H), 6.73 (t, J = 7.6 Hz, 1H), 6.65 (d, J = 7.6 Hz, 2H), 6.30 (dd, J_1 = 2.8, J_2 = 1.6 Hz, 1H), 6.17 (d, J = 3.2 Hz, 1H), 4.66 (q, J = 6.8 Hz, 1H), 3.87 (brs, 1H), 1.57 (d, J = 6.8 Hz, 3H) ppm; ^{13}C NMR (100 MHz, $CDCl_3$) δ 157.4, 147.2, 141.6, 129.4, 118.0, 113.7, 110.3, 105.3, 47.5, 21.1 ppm; IR (thin film) 3409, 3051, 2975, 2929, 2871, 1603, 1504, 1449, 1310, 1254, 1153, 1004, 748, 692, 598 (cm^{-1}); HRMS (ESI) m/z: calcd for $[C_{12}H_{14}NO]^+$188.1070; found 188.1068.

Preparation of compound sub-4:

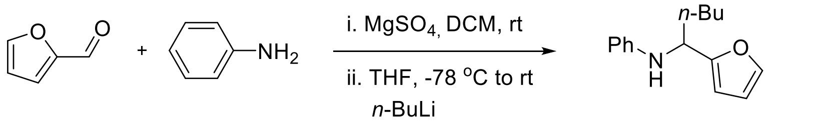

sub-**4**, 60% in two steps

Compound sub-4: To a solution of furan-2-carbaldehyde (0.41 mL, 5.0 mmol, 1.0 equiv) and aniline (0.46 mL, 5.0 mmol, 1.0 equiv) in anhydrous DCM (10 mL) was added anhydrous $MgSO_4$ (300 mg, 2.5 mmol, 0.5 equiv). A $CaCl_2$ drying tube was fitted to the flask and the resulting mixture was stirred at room temperature overnight, which was then filtered through a short pad of celite. The filtrate was dried over anhydrous Na_2SO_4, filtered, and concentrated to afford a crude mixture, which was used directly in next step without further purification.

To a stirred solution of the above crude mixture in anhydrous THF (20 mL) at -78 °C under inert atmosphere was added dropwise *n*-BuLi (2.5 M in hexanes, 4.0 mL, 10.0 mmol, 2.0 equiv). Upon completion of the reaction, it was quenched with saturated aqueous NH_4Cl (10 mL). The separated aqueous layer was extracted with EtOAc (20 mL x 3), and the combined organic layers were washed with brine (20 mL), dried over Na_2SO_4, filtered, and concentrated. Flash column chromatography (pet ether: EtOAc = 10: 1) afforded 688.0 mg (60% in two steps) of compound sub-**4** as a yellow oil. ^{1}H NMR (400 MHz, $CDCl_3$) δ 7.37-7.31 (m, 1H), 7.18-7.12 (m, 2H), 6.70 (t, J = 7.2 Hz, 1H), 6.62 (d, J = 8.0 Hz, 2H), 6.29 (dd, J_1 = 2.8, J_2 = 1.6 Hz, 1H), 6.16 (d, J = 3.2 Hz, 1H), 4.46 (t, J = 6.8 Hz, 1H), 3.89 (brs, 1H), 1.95-1.83 (m, 2H), 1.42-1.31 (m, 4H), 0.91 (t, J = 6.8 Hz, 3H) ppm; ^{13}C NMR (100 MHz, $CDCl_3$) δ 156.6, 147.5, 141.6, 129.4, 117.8, 113.6, 110.2, 106.0, 52.2, 35.1, 28.4, 22.7, 14.2 ppm; IR (thin film) 3414, 3052, 2956, 2931, 2859, 1602, 1503, 1465, 1429, 1314, 1255, 1150, 1009, 747, 691, 598 (cm^{-1}); HRMS (ESI) m/z: calcd for $[C_{15}H_{19}NNaO]^+$ 252.1359; found 252.1356.

Preparation of compound sub-5:

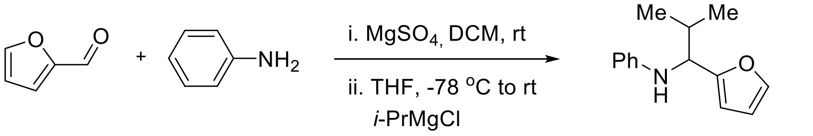

sub-**5**, 65% in two steps

Compound sub-5: To a solution of furan-2-carbaldehyde (0.41 mL, 5.0 mmol, 1.0 equiv) and aniline (0.46 mL, 5.0 mmol, 1.0 equiv) in anhydrous DCM (10 mL) was added anhydrous $MgSO_4$ (300 mg, 2.5 mmol, 0.5 equiv). A $CaCl_2$ drying tube was fitted to the flask and the resulting mixture was stirred at room temperature overnight, which was then filtered through a short pad of celite. The filtrate was dried over anhydrous Na_2SO_4, filtered, and concentrated to afford a crude mixture, which was used directly in next step without further purification.

To a stirred solution of the above crude mixture in anhydrous THF (20 mL) at -78 °C under inert atmosphere was added dropwise isopropylmagnesium chloride (*i*-PrMgCl) (2.0 M in THF, 5.0 mL, 10.0 mmol, 2.0 equiv). Upon completion of the reaction, it was quenched with saturated aqueous NH_4Cl (10 mL). The separated aqueous layer was extracted with EtOAc (20 mL x 3), and the combined organic layers were washed with brine (20 mL), dried over Na_2SO_4, filtered, and concentrated. Flash column chromatography (pet ether: EtOAc = 10: 1) afforded 700 mg (65% in two steps) of compound sub-**5** as a yellow oil. ^{1}H NMR (400 MHz, $CDCl_3$) δ 7.33 (dd, J_1 = 2.0, J_2 = 0.8 Hz, 1H), 7.13 (dd, J_1 = 8.8, J_2 = 7.6 Hz, 2H), 6.67 (t, J = 7.6 Hz, 1H), 6.60 (d, J = 7.6 Hz, 2H), 6.27 (dd, J_1 = 3.2, J_2 = 1.6 Hz, 1H), 6.13 (d, J = 3.2 Hz, 1H), 4.25 (d, J = 6.4 Hz, 1H), 3.96 (brs, 1H), 2.18 (dq, J_1 = 13.2, J_2 = 6.8 Hz, 1H), 1.02 (d, J = 6.8 Hz, 3H), 0.94 (d, J = 6.4 Hz, 3H) ppm; ^{13}C NMR (100 MHz, $CDCl_3$) δ 155.7, 147.8, 141.5, 129.4, 117.7, 113.6, 110.2, 106.8, 58.0, 33.0, 19.2, 19.1 ppm; IR (thin film) 3423, 3052, 2961, 2933, 2872, 1602, 1504, 1466, 1431, 1385, 1318, 1152, 1090, 1009, 860, 804, 748, 691, 598, 543, 474 (cm^{-1}); HRMS (ESI) m/z: calcd for $[C_{14}H_{17}NNaO]^+$ 238.1202; found 238.1211.

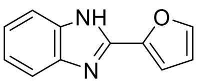

Fuberidazole

Compound Fuberidazole: The compound of Fuberidazole is commercially available.

Preparation of compound sub-6:

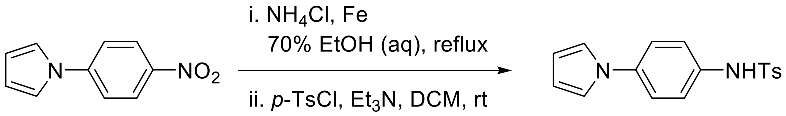

sub-**6**, 60% in two steps

Compound sub-6: A mixture of 1-(4-nitrophenyl)-1*H*-pyrrole (429.1 mg, 2.28 mmol, 1.0 equiv), iron powder (382 mg, 6.84 mmol, 3.0 equiv), and NH_4Cl (1.10 g, 20.53 mmol, 9.0 equiv) in ethanol (70%, 15 mL) was heated to reflux for one hour. The resulting mixture was cooled to room temperature, which was then filtered through a short pad of celite. The filtrate was dried over anhydrous Na_2SO_4, filtered, and concentrated to afford a crude pale-yellow oil, which was used directly in next step without further purification.

To the above crude pale-yellow oil in anhydrous DCM (10 mL) at room temperature under inert atmosphere was added Et_3N (0.63 mL, 4.56 mmol, 2.0 equiv) and *p*-TsCl (478.5 mg, 2.51 mmol, 1.1 equiv). The resulting mixture was stirred at room temperature overnight, which was then quenched with water (10 mL). The separated aqueous layer was extracted with DCM (20 mL x 3), and the combined organic layers were washed with brine (20 mL), dried over Na_2SO_4, filtered, and concentrated. Flash column chromatography (pet ether: EtOAc = 5: 1) afforded 427 mg (60% in two steps) of compound sub-**6** as a pale-yellow solid. Mp: 158-160 °C; ^1H NMR (400 MHz, $CDCl_3$) δ 7.70 (d, J = 8.0 Hz, 2H), 7.29-7.24 (m, 4H), 7.15 (d, J = 8.8 Hz, 2H), 7.07 (s, 1H), 7.04-7.01 (m, 2H), 6.36-6.32 (m, 2H), 2.40 (s, 3H) ppm; ^{13}C NMR (100 MHz, $CDCl_3$) δ 144.3, 138.5, 136.1, 134.1, 129.9, 127.5, 123.5, 121.3, 119.4, 110.8, 21.8 ppm; IR (thin film) 3271, 1596, 1519, 1470, 1332, 1161, 1091, 921, 815, 724, 665, 574, 547 (cm^{-1}); HRMS (ESI) m/z: calcd for $[C_{17}H_{17}N_2O_2S]^+$313.1005; found 313.1013.

Preparation of compound sub-7:

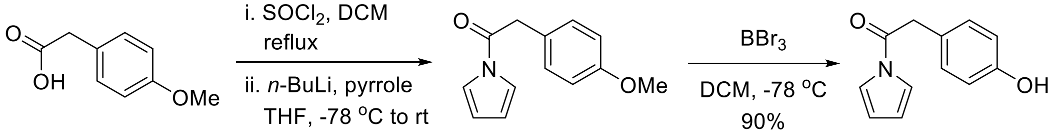

48% in two steps

sub-**7**

2-(4-Methoxyphenyl)-1-(1*H*-pyrrol-1-yl)ethan-1-one: To a solution of 2-(4-methoxyphenyl)acetic acid (1.99 g, 12.0 mmol, 1.2 equiv) and $SOCl_2$ (1.70 mL, 24.0 mmol, 2.4 equiv) in anhydrous DCM (10 mL) was heated to reflux for two hours, which was then cooled to room temperature. The excess thionyl chloride was removed under vacuum to afford a crude material, which was used directly in next step without further purification.

To a solution of pyrrole (0.69 mL, 10.0 mmol, 1.0 equiv) in anhydrous THF (20 mL) at -78 °C was added dropwise *n*-BuLi (2.5 M in hexanes, 4.8 mL, 12.0 mmol, 1.2 equiv) and the resulting mixture was stirred at -78 °C for 10 minutes. The above crude material was dissolved in anhydrous THF and added dropwise, the resulting mixture was allowed to warm to room temperature. Upon completion of the reaction, the resulting mixture was quenched with 10% aq $NaHCO_3$ (10 mL). The separated aqueous layer was extracted with EtOAc (20 mL x 3), and the combined organic layers were washed with brine (20 mL), dried over Na_2SO_4, filtered, and concentrated. Flash column chromatography (pet ether: EtOAc = 20: 1 to 8: 1) afforded 1.03 g (48% in two steps) of 2-(4-methoxyphenyl)-1-(1*H*-pyrrol-1-yl)ethan-1-one as a white solid. Mp: 98-100 °C; ^1H NMR (400 MHz, $CDCl_3$) δ 7.37 (s, 2H), 7.21 (d, J = 8.8 Hz, 2H), 6.89 (d, J = 8.8 Hz, 2H), 6.30 (t, J = 2.4 Hz, 2H), 4.09 (s, 2H), 3.80 (s, 3H) ppm; ^{13}C NMR (100 MHz, $CDCl_3$) δ 168.9, 159.1, 130.4, 125.2, 119.6, 114.5, 113.4, 55.4, 40.9 ppm; IR (thin film) 3134, 3006, 2956, 2836,

1721, 1614, 1541, 1466, 1353, 1322, 1306, 1251, 1179, 1077, 1036, 920, 823, 787, 745, 612, 550 (cm^{-1}); HRMS (ESI) m/z: calcd for $[C_{13}H_{13}NNaO_2]^+$ 238.0838; found 238.0848.

Compound sub-7: To a solution of 2-(4-methoxyphenyl)-1-(1*H*-pyrrol-1-yl)ethan-1-one (500 mg, 2.32 mmol, 1.0 equiv) in anhydrous DCM (10 mL) at -78 °C was added dropwise BBr_3 (1.0 M in DCM, 9.29 mL, 9.29 mmol, 4.0 equiv). The resulting mixture was stirred at -78 °C for two hours. Upon completion of the reaction, the resulting solution was quenched with 10% aq $NaHCO_3$ (10 mL). The separated aqueous layer was extracted with DCM (20 mL x 3), and the combined organic layers were washed with brine (20 mL), dried over Na_2SO_4, filtered, and concentrated. Flash column chromatography (pet ether: EtOAc = 8: 1 to 4: 1) afforded 421 mg (90%) of compound sub-**7** as a white solid. Mp: 141-143 °C; ^1H NMR (400 MHz, DMSO-*d*6) δ 9.33 (s, 1H), 7.51 (t, J = 2.4 Hz, 2H), 7.11 (d, J = 8.8 Hz, 2H), 6.71 (d, J = 8.4 Hz, 2H), 6.31 (t, J = 2.4 Hz, 2H), 4.16 (s, 2H) ppm; ^{13}C NMR (100 MHz, DMSO-*d*6) δ 169.5, 156.3, 130.4, 124.0, 119.6, 115.2, 112.9, 39.6 ppm; IR (thin film) 3141, 2910, 1914, 1712, 1618, 1520, 1467, 1396, 1356, 1247, 1173, 1116, 1076, 942, 840, 795, 741, 701, 610, 524, 498 (cm^{-1}); HRMS (ESI) m/z: calcd for $[C_{12}H_{11}NNaO_2]^+$ 224.0682; found 224.0695.

Preparation of compound sub-8:

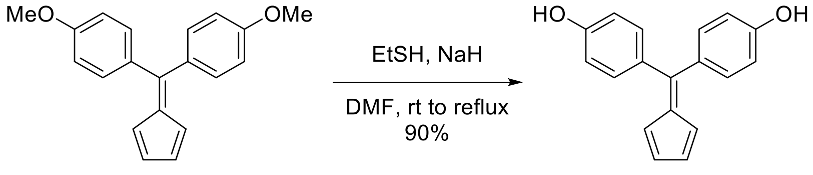

sub-**8**

Compound sub-8: The preparation of 4,4'-(cyclopenta-2,4-dien-1-ylidenemethylene) bis(methoxybenzene) from bis(4-methoxyphenyl)methanone followed a known procedure.[9] To a solution of ethanethiol (EtSH) (2.96 mL, 40.0 mmol, 10.0 equiv) in anhydrous DMF (15 mL) at room temperature was slowly added NaH (60% in mineral oil, 1.6 g, 40.0 mmol, 10.0 equiv). After 20 minutes, 4,4'-(cyclopenta-2,4-dien-1-ylidenemethylene)bis(methoxybenzene) (1.16 g, 4.0 mmol, 1.0 equiv) was added. The resulting mixture was heated to reflux for five hours. Upon completion of the reaction, it was quenched with saturated aqueous NH_4Cl (10 mL). The separated aqueous layer was extracted with EtOAc (40 mL x 3), and the combined organic layers were washed with water (200 mL x 3), brine (200 mL), dried over Na_2SO_4, filtered, and concentrated. Flash column chromatography (DCM: MeOH = 20: 1) afforded 944 mg (90%) of compound sub-**8** as a red solid. Mp: 200-202 °C; ^1H NMR (400 MHz, DMSO-*d*6) δ 9.92 (s, 2H), 7.08 (d, J = 8.4 Hz, 4H), 6.83 (d, J = 8.4 Hz, 4H), 6.53 (d, J = 6.4 Hz, 2H), 6.16 (d, J = 6.4 Hz, 2H) ppm; ^{13}C NMR (100 MHz, DMSO-*d*6) δ 158.8, 152.9, 140.4, 133.8, 131.6, 130.4, 123.7, 114.8 ppm; IR (thin film) 3426, 2919, 2852, 2344, 1780, 1637, 1399, 1384, 1094, 984, 860, 617, 543, 474 (cm^{-1}); HRMS (ESI) m/z: calcd for $[C_{18}H_{15}O_2]^+$ 263.1067; found 263.1074.

Preparation of compound sub-9:

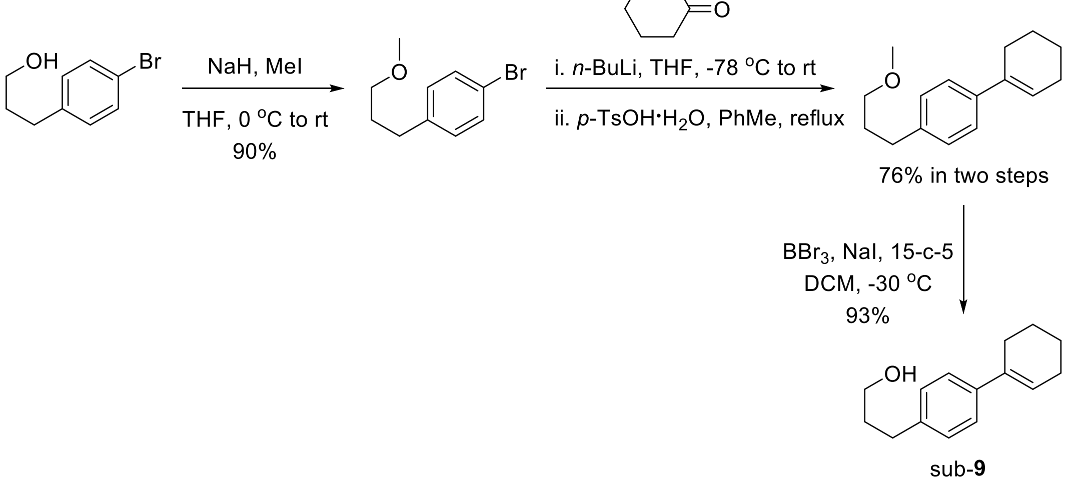

sub-**9**

1-Bromo-4-(3-methoxypropyl)benzene: To a solution of 3-(4-bromophenyl)propan-1-ol (1.53 mL, 10.0 mmol, 1.0 equiv) in anhydrous THF (20 mL) at 0 °C was slowly added NaH (60% in mineral oil, 800 mg, 20.0 mmol, 2.0 equiv). After one hour, iodomethane (MeI) (0.93 mL, 15.0 mmol, 1.5 equiv) was added dropwise and the resulting mixture was allowed to warm to room temperature. Upon completion of the reaction, the resulting solution was then quenched with cold water (10 mL). The separated aqueous layer was extracted with EtOAc (20 mL x 3), and the combined organic layers were washed with brine (20 mL), dried over Na_2SO_4, filtered, and concentrated. Flash column chromatography (pet ether: EtOAc = 20: 1) afforded 2.06 g (90%) of 1-bromo-4-(3-methoxypropyl)benzene as a colorless oil. ^{1}H NMR (400 MHz, $CDCl_3$) δ 7.39 (d, *J* = 8.4 Hz, 2H), 7.06 (d, *J* = 8.4 Hz, 2H), 3.36 (t, *J* = 6.0 Hz, 2H), 3.34 (s, 3H), 2.64 (t, *J* = 7.6 Hz, 2H), 1.89-1.82 (m, 2H) ppm; ^{13}C NMR (100 MHz, $CDCl_3$) δ 141.1, 131.6, 130.4, 119.7, 71.8, 58.8, 31.9, 31.3 ppm; IR (thin film) 3027, 2984, 2926, 2866, 2827, 1897, 1627, 1488, 1452, 1403, 1385, 1118, 1072, 1011, 834, 795, 709, 635, 526, 497 (cm^{-1}); HRMS (ESI) m/z: calcd for $[C_{10}H_{13}BrNaO]^+$ 251.0042; found 251.0038.

4'-(3-Methoxypropyl)-2,3,4,5-tetrahydro-1,1'-biphenyl: To a solution of 1-bromo-4-(3-methoxypropyl)benzene (1.15 g, 5.0 mmol, 1.0 equiv) in anhydrous THF (10 mL) at -78 °C was added dropwise *n*-BuLi (2.5 M in hexanes, 2.2 mL, 5.5 mmol, 1.1 equiv). The resulting mixture was stirred at -78 °C for one hour. Cyclohexanone (0.57 mL, 5.5 mmol, 1.1 equiv) was added dropwise and the resulting mixture was allowed to warm to room temperature. Upon completion of the reaction, the resulting solution was then quenched with saturated aqueous NH_4Cl (10 mL). The separated aqueous layer was extracted with EtOAc (20 mL x 3), and the combined organic layers were washed with brine (20 mL), dried over Na_2SO_4, filtered, and concentrated to afford a crude pale-yellow oil, which was used directly in next step without further purification.

To the above crude pale-yellow oil in anhydrous toluene (20 mL) was added a catalytic amount of *p*-toluenesulfonic acid monohydrate (*p*-TsOH·H_2O) (95 mg, 0.5 mmol, 0.1 equiv), which was heated to reflux for 30 minutes. Upon completion of the reaction, the resulting solution was directly evaporated on a rotary evaporator. Flash column chromatography (pet ether) afforded 875 mg (76% in two steps) of 4'-(3-methoxypropyl)-2,3,4,5-tetrahydro-1,1'-biphenyl as a colorless oil. ^{1}H NMR (400 MHz, $CDCl_3$) δ 7.31 (d, *J* = 8.4 Hz, 2H), 7.13 (d, *J* = 8.4 Hz, 2H), 6.13-6.08 (m, 1H), 3.39 (t, *J* = 6.4 Hz, 2H), 3.35 (s, 3H), 2.67 (t, *J* = 7.6 Hz, 2H), 2.43-2.38 (m,2H), 2.23-2.17 (m, 2H), 1.93-1.84 (m, 2H), 1.81-1.74 (m, 2H), 1.69-1.62 (m, 2H) ppm; ^{13}C NMR (100 MHz, $CDCl_3$) δ 140.4, 136.5, 128.5, 125.0, 124.3, 72.2, 58.8, 32.1, 31.4, 27.6, 26.1, 23.3, 22.4 ppm; IR (thin film) 3026, 2926, 2856, 2831, 1645, 1512, 1448, 1385, 1347, 1119, 1072, 1019, 920, 884, 831, 796, 559, 504 (cm^{-1}); HRMS (ESI) m/z: calcd for $[C_{16}H_{22}NaO]^+$ 253.1563; found 253.1559.

Compound sub-9: To a solution of 4'-(3-methoxypropyl)-2,3,4,5-tetrahydro-1,1'-biphenyl (461 mg, 2.0 mmol, 1.0 equiv), NaI (1.80 g, 12.0 mmol, 6.0 equiv), 15-c-5 (2.38 mL, 12.0 mmol, 6.0 equiv) in anhydrous DCM (10 mL) at -30 °C was added dropwise BBr_3 (1.0 M in DCM, 6.0 mL, 6.0 mmol, 3.0 equiv). The resulting mixture was stirred at -30 °C for three hours. Upon completion of the reaction, it was quenched with 10% aq $NaHCO_3$ (10 mL). The separated aqueous layer was extracted with DCM (20 mL x 3), and the combined organic layers were washed with brine (20 mL), dried over Na_2SO_4, filtered, and concentrated. Flash column chromatography (pet ether: EtOAc = 20: 1 to 8: 1) afforded 402 mg (93%) of compound sub-**9** as a colorless oil. ^{1}H NMR (400 MHz, $CDCl_3$) δ 7.31 (d, *J* = 8.0 Hz, 2H), 7.14 (d, *J* = 8.4 Hz, 2H), 6.12-6.07 (m, 1H), 3.68 (t, *J* = 6.4 Hz, 2H), 2.69 (t, *J* = 7.6 Hz, 2H), 2.43-2.37 (m, 2H), 2.23-2.17 (m, 2H), 1.94-1.85 (m, 2H), 1.81-1.74 (m, 2H), 1.69-1.62 (m, 2H), 1.26 (s, 1H) ppm; ^{13}C NMR (100 MHz, $CDCl_3$) δ 140.6, 140.2, 136.5, 128.5, 125.2, 124.4, 62.6, 34.4, 31.9, 27.6, 26.1, 23.3, 22.4 ppm; IR (thin film) 3024, 2924, 2857, 2831, 1909, 1639, 1512, 1436, 1415, 1346, 1275, 1135, 1053, 1020, 920, 860, 827, 798, 737, 699, 629, 557, 517, 459 (cm^{-1}); HRMS (ESI) m/z: calcd for $[C_{15}H_{20}NaO]^+$ 239.1406; found 239.1413.

Preparation of compound sub-10:

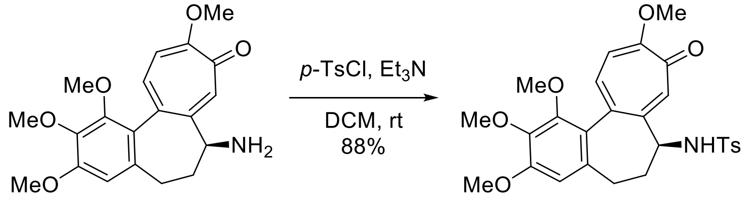

sub-**10**

Compound sub-10: The preparation of *N*-deacetyl-colchicine from colchicine followed a known procedure.[10] To *N*-deacetyl-colchicine (357 mg, 1.0 mmol, 1.0 equiv) in anhydrous DCM (10 mL) at room temperature under inert atmosphere was added Et_3N (0.28 mL, 2.0 mmol, 2.0 equiv) and *p*-TsCl (286 mg, 1.5 mmol, 1.5equiv). The resulting solution was stirred at room temperature overnight, which was then quenched with water (10 mL). The separated aqueous layer was extracted with DCM (20 mL x 3), and the combined organic layers were washed with brine (20 mL), dried over Na_2SO_4, filtered, and concentrated. Flash column chromatography (pet ether: EtOAc = 1: 10) afforded 450 mg (88%) of compound sub-**10** as a yellow solid. Mp: 119-121 °C; ^{1}H NMR (400 MHz, $CDCl_3$) δ 7.49 (d, *J* = 8.4 Hz, 2H), 7.22-7.16 (m, 2H), 7.10 (d, *J* = 8.4 Hz, 2H), 6.71 (d, *J* = 10.8 Hz, 1H), 6.47 (s, 1H), 5.07 (d, *J* = 7.6 Hz, 1H), 4.23-4.16 (m, 1H), 3.95 (s, 3H), 3.94 (s, 3H), 3.90 (s, 3H), 3.62 (s, 3H), 2.44 (dd, J_1 = 13.2, J_2 = 6.0 Hz, 1H), 2.33-2.15 (m, 5H), 1.79-1.70 (m, 1H) ppm; ^{13}C NMR (100 MHz, $CDCl_3$) δ 179.0, 164.0, 153.8, 151.2, 148.8, 143.6, 141.8, 137.5, 135.9, 135.1, 134.4, 132.5, 129.7, 127.2, 125.2, 112.0, 107.7, 61.5, 61.3, 56.4, 56.3, 55.9, 38.9, 30.2, 21.6 ppm; IR (thin film) 2932, 2846, 1617, 1588, 1560, 1487, 1458, 1396, 1324, 1253, 1159, 1092, 1016, 1002, 912, 844, 814, 706, 664, 568, 549, 485 (cm^{-1}); HRMS (ESI) m/z: calcd for $[C_{27}H_{30}NO_7S]^+$ 512.1737; found 512.1740.

Preparation of compound sub-11:

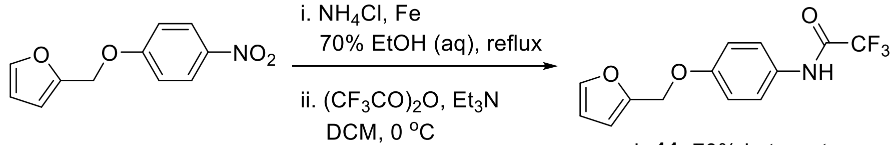

sub-**11**, 70% in two steps

Compound sub-11: A mixture of 2-((4-nitrophenoxy)methyl)furan (500 mg, 2.28 mmol, 1.0 equiv), iron powder (382 mg, 6.84 mmol, 3.0 equiv), and NH_4Cl (1.10 g, 20.53 mmol, 9.0 equiv) in ethanol (70%, 15 mL) was heated to reflux for one hour. The resulting mixture was cooled to room temperature, which was then filtered through a short pad of celite. The filtrate was dried over anhydrous Na_2SO_4, filtered, and concentrated to afford a crude pale-yellow oil, which was used directly in next step without further purification.

To the above crude pale-yellow oil in anhydrous DCM (10 mL) at 0 °C under inert atmosphere was added dropwise Et_3N (0.63 mL, 4.56 mmol, 2.0 equiv) and trifluoroacetic anhydride (0.48 mL, 3.42 mmol, 1.5 equiv). The resulting mixture was stirred at 0 °C for one hour, which was then quenched with water (10 mL). The separated aqueous layer was extracted with DCM (20 mL x 3), and the combined organic layers were washed with brine (20 mL), dried over Na_2SO_4, filtered, and concentrated. Flash column chromatography (pet ether: EtOAc = 5: 1) afforded 455 mg (70% in two steps) of compound sub-**11** as a white solid. Mp: 140-142 °C; ^{1}H NMR (400 MHz, $CDCl_3$) δ 7.88 (s, 1H), 7.47 (d, *J* = 8.8 Hz, 2H), 7.45 (d, *J* = 2.0 Hz, 1H), 6.99 (d, *J* = 8.8 Hz, 2H), 6.44 (d, *J* = 3.2 Hz, 1H), 6.38 (dd, J_1 = 3.2, J_2 = 1.6 Hz, 1H), 5.00 (s, 2H) ppm; ^{13}C NMR (100 MHz, $CDCl_3$) δ 156.7, 154.9 (q, *J* = 36.9 Hz), 150.1, 143.5, 128.7, 122.4, 116.0 (q, *J* = 286.8 Hz), 115.8, 110.8, 110.4, 62.9 ppm; IR (thin film) 3138, 3077, 2938, 2865, 1700, 1614, 1544, 1517, 1453, 1420, 1351, 1309, 1254, 1188, 1151, 997, 924, 885, 828, 743, 721, 678, 598, 534 (cm^{-1}); HRMS (ESI) m/z: calcd for $[C_{13}H_{10}F_3NNaO_3]^+$ 308.0505; found 308.0518.

Preparation of compound sub-12:

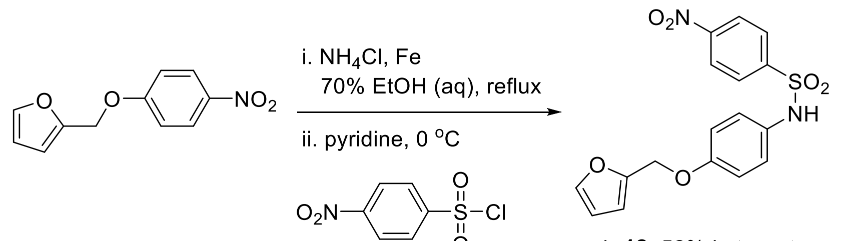

Compound sub-12: A mixture of 2-((4-nitrophenoxy)methyl)furan (500 mg, 2.28 mmol, 1.0 equiv), iron powder (382 mg, 6.84 mmol, 3.0 equiv), and NH_4Cl (1.10 g, 20.53 mmol, 9.0 equiv) in ethanol (70%, 15 mL) was heated to reflux for one hour. The resulting mixture was cooled to room temperature, which was then filtered through a short pad of celite. The filtrate was dried over anhydrous Na_2SO_4, filtered, and concentrated to afford a crude pale-yellow oil, which was used directly in next step without further purification.

To the above crude pale-yellow oil in anhydrous pyridine (6.0 mL) at 0 °C under inert atmosphere was added 4-nitrobenzene-1-sulfonyl chloride (758 mg, 3.42 mmol, 1.5 equiv). The resulting mixture was allowed to warm to room temperature. Upon completion of the reaction, the resulting mixture was quenched with water (10 mL). The separated aqueous layer was extracted with EtOAc (20 mL x 3), and the combined organic layers were washed with 10% aq HCl (50 mL x 3), dried over Na_2SO_4, filtered, and concentrated. Flash column chromatography (pet ether: EtOAc = 4: 1) afforded 443 mg (52% in two steps) of compound sub-**12** as a white solid. Mp: 205-207 °C; ^{1}H NMR (400 MHz, DMSO-*d6*) δ 10.28 (s, 1H), 8.36 (d, J = 8.8 Hz, 2H), 7.91 (d, J = 9.2 Hz, 2H), 7.72-7.61 (m, 1H), 6.98 (d, J = 9.2 Hz, 2H), 6.91 (d, J = 9.2 Hz, 2H), 6.54 (d, J = 3.2 Hz, 1H), 6.44 (dd, J_1 = 3.2, J_2 = 2.0 Hz, 1H), 4.96 (s, 2H) ppm; ^{13}C NMR (100 MHz, DMSO-*d6*) δ 155.6, 149.9, 149.7, 144.8, 143.5, 129.6, 128.3, 124.5, 123.9, 115.4, 110.6, 110.5, 61.6 ppm; IR (thin film) 3433, 3275, 2925, 1610, 1523, 1507, 1385, 1350, 1241, 1162, 1106, 1003, 854, 751, 742, 682, 541, 466 (cm^{-1}); HRMS (ESI) m/z: calcd for $[C_{17}H_{14}N_2NaO_6S]^+$ 397.0465; found 397.0468.

Preparation of compound sub-13:

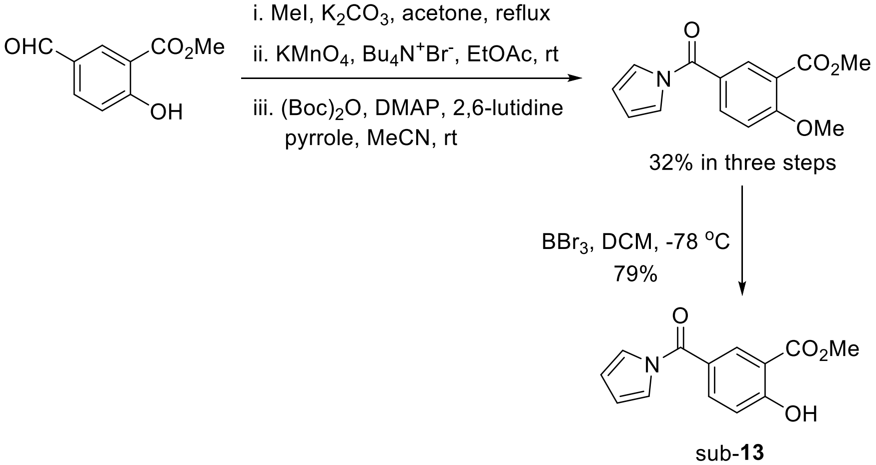

Methyl 2-methoxy-5-(1*H*-pyrrole-1-carbonyl)benzoate: A suspension of methyl 5-formyl-2-hydroxybenzoate (0.90 g, 5.0 mmol, 1.0 equiv), MeI (0.63 mL, 10.0 mmol, 2.0 equiv), and K_2CO_3 (1.38 g, 10.0 mmol, 2.0 equiv) in acetone (25 mL) was heated to reflux. Upon completion of the reaction, the resulting mixture was cooled to room temperature. It was filtered through a short plug of silica gel, rinsed with EtOAc, and concentrated to afford a crude white solid, which was used directly in next step without further purification.

To the above crude white solid in EtOAc (30 mL) was added a catalytic amount of tetrabutylammonium bromide ($Bu_4N^+Br^-$) (161 mg, 0.5 mmol, 0.1 equiv) and $KMnO_4$ (3.95 g, 25.0 mmol, 5.0 equiv). The resulting mixture was stirred at room temperature overnight, which was quenched with water (10 mL) and then concentrated HCl to tune the pH value to 2. Excess $KMnO_4$ was quenched with $FeSO_4$ powder, and the crude material was then filtered over a short pad of celite. The filtrate was extracted with EtOAc

(50 mL x 3), and the combined organic layers were washed with brine (50 mL), dried over Na_2SO_4, filtered, and concentrated to afford a crude white solid, which was used directly in next step without further purification.

To a solution of the above crude white solid in anhydrous MeCN (20 mL) at room temperature under inert atmosphere was added DMAP (31 mg, 0.25 mmol, 0.05 equiv), 2,6-lutidine (60 uL, 0.5 mmol, 0.1 equiv), pyrrole (0.87 mL, 12,5 mmol, 2.5 equiv), and di-*tert*-butyl dicarbonate ($(Boc)_2O$) (2.88 mL, 12.5 mmol, 2.5 equiv). The resulting mixture was stirred at room temperature overnight. Upon completion of the reaction, it was quenched with water (10 mL). The separated aqueous layer was extracted with EtOAc (20 mL x 3), and the combined organic layers were washed with brine (20 mL), dried over Na_2SO_4, filtered, and concentrated. Flash column chromatography (pet ether: EtOAc = 6: 1) afforded 415 mg (32% in three steps) of pure methyl 2-methoxy-5-(1*H*-pyrrole-1-carbonyl)benzoate as a white solid. Mp: 100-102 °C; ^{1}H NMR (400 MHz, $CDCl_3$) δ 8.25 (d, J = 2.4 Hz, 1H), 7.93 (dd, J_1 = 8.8, J_2 = 2.4 Hz, 1H), 7.27 (t, J = 2.4 Hz, 2H), 7.09 (d, J = 8.8 Hz, 1H), 6.36 (t, J = 2.0 Hz, 2H), 4.00 (s, 3H), 3.91 (s, 3H) ppm; ^{13}C NMR (100 MHz, $CDCl_3$) δ 166.5, 165.6, 162.3, 135.5, 133.9, 125.1, 121.5, 120.3, 113.4, 112.1, 56.6, 52.5 ppm; IR (thin film) 3134, 2955, 2924, 2852, 1702, 1689, 1608, 1501, 1431, 1400, 1335, 1288, 1244, 1199, 1155, 1100, 1014, 966, 902, 840, 758, 682, 616, 539 (cm^{-1}); HRMS (ESI) m/z: calcd for $[C_{14}H_{14}NO_4]^+$ 260.0917; found 260.0924.

Compound sub-13: To a solution of methyl 2-methoxy-5-(1*H*-pyrrole-1-carbonyl)benzoate (259 mg, 1.0 mmol, 1.0 equiv) in anhydrous DCM (5 mL) at -78 °C was added dropwise BBr_3 (1.0 M in DCM, 3.0 mL, 3.0 mmol, 3.0 equiv). The resulting mixture was stirred at -78 °C for one hour. Upon completion of the reaction, it was quenched with 10% aq $NaHCO_3$ (10 mL). The separated aqueous layer was extracted with DCM (10 mL x 3), and the combined organic layers were washed with brine (20 mL), dried over Na_2SO_4, filtered, and concentrated. Flash column chromatography (pet ether: EtOAc = 20: 1) afforded 194 mg (79%) of compound sub-**13** as a white solid. Mp: 75-77 °C; ^{1}H NMR (400 MHz, $CDCl_3$) δ 11.23 (s, 1H), 8.33 (d, J = 2.4 Hz, 1H), 7.90 (dd, J_1 = 8.8, J_2 = 2.4 Hz, 1H), 7.27 (t, J = 2.0 Hz, 2H), 7.10 (d, J = 8.4 Hz, 1H), 6.37 (t, J = 2.0 Hz, 2H), 3.98 (s, 3H) ppm; ^{13}C NMR (100 MHz, $CDCl_3$) δ 170.0, 166.4, 165.0, 137.1, 132.9, 124.3, 121.5, 118.3, 113.4, 112.5, 53.0 ppm; IR (thin film) 3149, 3139, 2963, 1698, 1668, 1588, 1490, 1441, 1366, 1322, 1294, 1264, 1224, 1089, 914, 838, 756, 729, 689, 650, 538 (cm^{-1}); HRMS (ESI) m/z: calcd for $[C_{13}H_{12}NO_4]^+$ 246.0761; found 246.0764.

Preparation of Furosemide methyl ester sub-14:

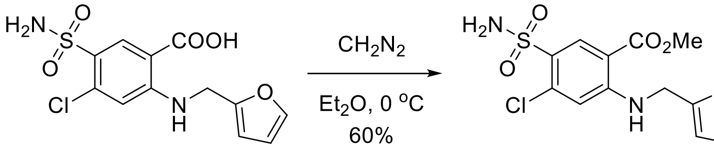

sub-**14**

Compound sub-14: To a solution of commercially available Furosemide (331 mg, 1.0 mmol, 1.0 equiv) in Et_2O (4.0 mL) at 0 °C was added excess amount of diazomethane, prepared from *N*,4-dimethyl-*N*-nitrosobenzenesulfonamide, and stirred for one hour. Upon completion of the reaction, it was then quenched with acetic acid. The separated aqueous layer was extracted with EtOAc (10 mL x 3), and the combined organic layers were washed with brine (20 mL), dried over Na_2SO_4, filtered, and concentrated. Flash column chromatography (pet ether: EtOAc = 3: 1) afforded 207 mg (60%) of Furosemide methyl ester sub-**14** as a white solid. Mp: 184-186 °C; ^{1}H NMR (400 MHz, $CDCl_3$) δ 8.65 (s, 1H), 8.62 (s, 1H), 7.40 (s, 1H), 6.87 (s, 1H), 6.35 (dd, J_1 = 2.8, J_2 = 2.0 Hz, 1H), 6.28 (d, J = 3.2 Hz, 1H), 4.97 (s, 2H), 4.45 (d, J = 6.0 Hz, 2H), 3.88 (s, 3H) ppm; ^{13}C NMR (100 MHz, $CDCl_3$) δ 167.8, 153.3, 150.4, 142.9, 137.4, 134.4, 125.6, 113.7, 110.7, 108.5, 108.0, 52.4, 40.5 ppm; IR (thin film) 3280, 3111, 2998, 2951, 2923, 2852, 1875, 1686, 1594, 1499, 1440, 1327, 1268, 1235, 1168, 1109, 1015, 937, 832, 738, 691, 589, 514 (cm^{-1}); HRMS (ESI) m/z: calcd for $[C_{13}H_{13}ClN_2NaO_5S]^+$ 367.0126; found 367.0139.

Preparation of compound sub-15:

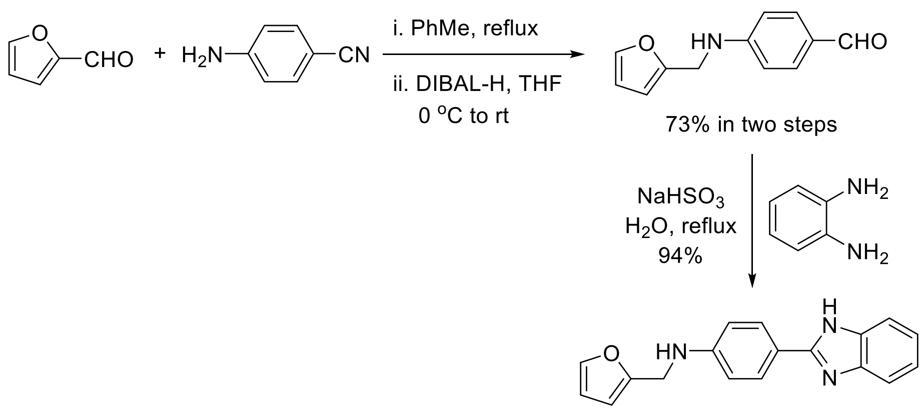

sub-**15**

4-((Furan-2-ylmethyl)amino)benzaldehyde: A solution of furan-2-carbaldehyde (0.41 mL, 5.0 mmol, 1.0 equiv) and 4-aminobenzonitrile (591 mg, 5.0 mmol, 1.0 equiv) in anhydrous toluene (15 mL) was heated under reflux for 24 hours. All the volatiles were removed under reduced pressure to afford a crude material, which was used directly in next step without further purification.

To a stirred solution of the above crude material in anhydrous THF (20 mL) at 0 °C under inert atmosphere was added dropwise diisobutylaluminum hydride (DIBAL-H) (1.0 M in toluene, 15.0 mL, 15.0 mmol, 3.0 equiv). Upon completion of the reaction, it was quenched with saturated aqueous NH_4Cl (10 mL). The separated aqueous layer was extracted with EtOAc (20 mL x 3), and the combined organic layers were washed with brine (20 mL), dried over Na_2SO_4, filtered, and concentrated. Flash column chromatography (pet ether: EtOAc = 8: 1 to 4: 1) afforded 734 mg (73% in two steps) of 4-((furan-2-ylmethyl)amino)benzaldehyde as a yellow solid. Mp: 75-77 °C; ^{1}H NMR (400 MHz, $CDCl_3$) δ 9.72 (s, 1H), 7.70 (d, J = 8.8 Hz, 2H), 7.37 (d, J = 1.2 Hz, 1H), 6.68 (d, J = 8.8 Hz, 2H), 6.33 (dd, J_1 = 3.2, J_2 = 2.0 Hz, 1H), 6.26 (d, J = 3.2 Hz, 1H), 4.81 (s, 1H), 4.39 (s, 2H) ppm; ^{13}C NMR (100 MHz, $CDCl_3$) δ 190.6, 152.9, 151.4, 142.5, 132.4, 127.2, 112.3, 110.6, 107.8, 40.7 ppm; IR (thin film) 3142, 2917, 2845, 2769, 1909, 1660, 1587, 1527, 1340, 1271, 1224, 1160, 1075, 1009, 915, 824, 759, 733, 604, 565, 508 (cm^{-1}); HRMS (ESI) m/z: calcd for $[C_{12}H_{11}NNaO_2]^+$ 224.0682; found 224.0683.

Compound sub-15: A mixture of 4-((furan-2-ylmethyl)amino)benzaldehyde (402 mg, 2.0 mmol, 1.0 equiv) and $NaHSO_3$ (2.29 g, 22.0 mmol, 11 equiv) in water (5.0 mL) was heated to reflux. A solution of *o*-phenylenediamine (216 mg, 2.0 mmol, 1.0 equiv) in water (5 mL) was added dropwise to the solution and the resulting mixture was refluxed for 15 hours. Upon completion of the reaction, it was cooled to room temperature. The precipitate was isolated by filtration, which was washed with water and dried under vacuum to afford 544 mg (94%) of compound sub-**15** as a white solid. Mp: 102-204 °C; ^{1}H NMR (400 MHz, DMSO-*d*6) δ 12.48 (brs, 1H), 7.89 (d, J = 8.8 Hz, 2H), 7.59 (d, J = 0.8 Hz, 1H), 7.49 (dd, J_1 = 5.2, J_2 = 2.8 Hz, 2H), 7.14-7.09 (m, 2H), 6.77 (d, J = 8.8 Hz, 2H), 6.63 (t, J = 5.6 Hz, 1H), 6.42- 6.38 (m, 1H), 6.34 (d, J = 2.8 Hz, 1H), 4.33 (d, J = 6.0 Hz, 2H) ppm; ^{13}C NMR (100 MHz, DMSO-*d*6) δ 152.9, 152.4, 149.8, 142.1, 127.6, 121.3, 117.7, 112.1, 110.4, 107.1 ppm; IR (thin film) 3052, 2924, 2855, 2790, 2744, 1885, 1611, 1499, 1476, 1446, 1421, 1393, 1315, 1271, 1185, 1113, 1073, 1009, 964, 917, 824, 744, 544 (cm^{-1}); HRMS (ESI) m/z: calcd for $[C_{18}H_{16}N_3O]^+$ 290.1288; found 290.1296.

Preparation of compound sub-16:

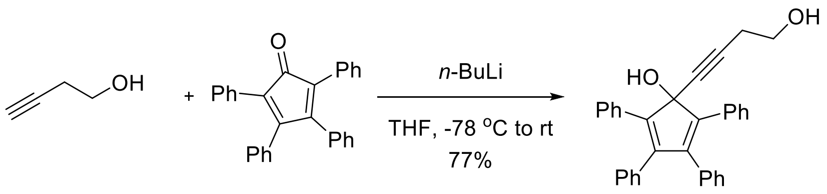

sub-**16**

Compound sub-16: To a solution of but-3-yn-1-ol (0.30 mL, 4.0 mmol, 1.0 equiv) in anhydrous THF (10 mL) at -78 °C was added dropwise *n*-BuLi (2.5 M in hexanes, 3.2 mL, 8.0 mmol, 2.0 equiv). The resulting solution was stirred at -78 °C for 30 minutes. A solution of tetraphenylcyclopentadienone (1.85 g, 4.8

mmol, 1.2 equiv) in anhydrous THF (20 mL) was added dropwise, and the resulting solution was allowed to warm to room temperature. Upon completion of the reaction, it was then quenched with saturated aqueous NH_4Cl (10 mL). The separated aqueous layer was extracted with EtOAc (20 mL x 3), and the combined organic layers were washed with brine (20 mL), dried over Na_2SO_4, filtered, and concentrated. Flash column chromatography (pet ether: EtOAc = 8: 1 to 3: 1) afforded 1.4 g (77%) of compound sub-**16** as a white solid. Mp: 77-79 °C; ^{1}H NMR (400 MHz, $CDCl_3$) δ 7.55-7.51 (m, 4H), 7.26-7.22 (m, 6H), 7.17-7.10 (m, 6H), 6.97-6.92 (m, 4H), 3.45 (t, J = 5.6 Hz, 2H), 2.57 (brs, 1H), 2.42 (t, J = 6.0 Hz, 2H), 1.25 (brs, 1H) ppm; ^{13}C NMR (100 MHz, $CDCl_3$) δ 143.2, 142.7, 134.8, 134.1, 130.1, 129.9, 128.3, 128.1, 127.7, 127.5, 83.8, 82.6, 81.4, 61.0, 23.7 ppm; IR (thin film) 3406, 3082, 1597, 1490, 1442, 1400, 1384, 1328, 1118, 1072, 1002, 913, 806, 740, 711, 698, 548, 479 (cm^{-1}); HRMS (ESI) m/z: calcd for $[C_{33}H_{26}NaO_2]^+$ 477.1825; found 477.1829.

2.3 Intermolecular aryne trapping reactions with various arynophiles

General procedure for intermolecular aryne trapping reactions with various arynophiles

Procedure B: A mixture of aryne precursor **1** (0.5 mmol, 1.0 equiv), arynophile **2** (1.5 mmol, 3.0 equiv), arynophile **3** (1.5 mmol, 3.0 equiv), 18-c-6 (264 mg, 1.0 mmol, 2.0 equiv), and CsF (228 mg, 1.5 mmol, 3.0 equiv) in anhydrous MeCN (5.0 mL) under inert atmosphere was stirred at room temperature overnight. The resulting mixture was filtered through a short pad of silica gel (EtOAc eluent, 20 mL). All the volatiles were removed on rotary evaporator. The crude material was purified by flash column chromatography (pet ether: EtOAc) to afford the corresponding products.

Procedure C: A mixture of aryne precursor **1** (0.5 mmol, 1.0 equiv), arynophile **2** (1.5 mmol, 3.0 equiv), arynophile **3** (1.5 mmol, 3.0 equiv) and CsF (456 mg, 3.0 mmol, 6.0 equiv) in anhydrous MeCN (5.0 mL) under inert atmosphere was stirred at room temperature overnight. The resulting mixture was filtered through a short pad of silica gel (EtOAc eluent, 20 mL). All the volatiles were removed on rotary evaporator. The crude material was purified by flash column chromatography (pet ether: EtOAc) to afford the corresponding products.

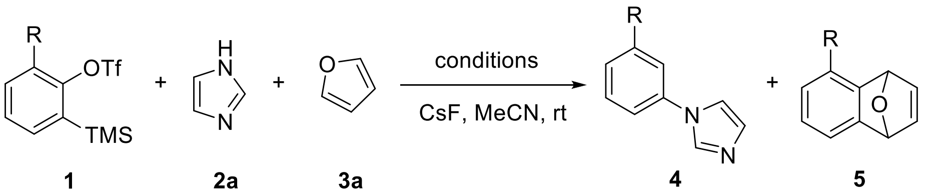

entry	R	additive	yield of **4**	yield of **5**	ratio of **4**: **5**
1	H, **1a**	no	36% (**4a**)	47% (**5a**)	1: 1.3
2	H, **1a**	18-c-6	57% (**4a**)	27% (**5a**)	2.1: 1
3	OMe, **1b**	18-c-6	74% **(4b)**	4% **(5b)**	19: 1
4	TBS, **1c**	no	7% (**4c**)	72% (**5c**)	1: 10
5	Cl, **1d**	18-c-6	34% (**4d**)	12% (**5d**)	3: 1
6	TMS, **1e**	no	21% (**4e**)	43% (**5e**)	1: 2
7	*t*-Bu, **1f**	no	35% (**4f**)	27% (**5f**)	1.3: 1
8	F, **S1**	18-c-6	52% (**S9**)	8% (**S10**)	6.5: 1
9	Br, **S2**	18-c-6	27% (**S11**)	13% (**S12**)	2.1: 1
10	TES, **S3**	no	15% (**S13**)	50% (**S14**)	1: 3.3
11	TIPS, **S4**	no	10% (**S15**)	58% (**S16**)	1: 5.8

Supplementary Table 1. Aryne trapping reactions with imidazole and furan

Reaction of 1a with imidazole (2a) and furan (3a): 41.1 mg (57%) of compound **4a** and 19.5 mg (27%) of compound **5a** were obtained by following general procedure B.

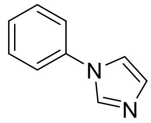

4a

Compound 4a: The ^{1}H NMR of **4a** is identical with that reported in literature.[11] ^{1}H NMR (400 MHz, $CDCl_3$) δ 7.85 (s, 1H), 7.50-7.46 (m, 2H), 7.40-7.35 (m, 3H), 7.28 (s, 1H), 7.21 (s, 1H) ppm.

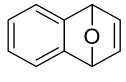

5a

Compound 5a: The ^{1}H NMR of **5a** is identical with that reported in literature.[12] ^{1}H NMR (400 MHz, $CDCl_3$) δ 7.26-7.25 (m, 2H), 7.03 (s, 2H), 6.99-6.96 (m, 2H), 5.72 (s, 2H) ppm.

Reaction of 1b with imidazole (2a) and furan (3a): 64.5 mg (74%) of compound **4b** and 3.5 mg (4%) of compound **5b** were obtained by following general procedure B.

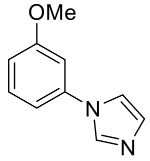

4b

Compound 4b: The ^{1}H NMR of **4b** is identical with that reported in literature.[13] ^{1}H NMR (400 MHz, $CDCl_3$) δ 7.84 (s, 1H), 7.36 (t, J = 8.0 Hz, 1H), 7.26 (s, 1H), 7.18 (s, 1H), 6.96 (d, J = 8.4 Hz, 1H), 6.89 (d, J = 8.4 Hz, 2H), 3.84 (s, 3H) ppm.

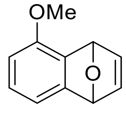

5b

Compound 5b: The ^{1}H NMR of **5b** is identical with that reported in literature.[14] ^{1}H NMR (400 MHz, $CDCl_3$) δ 7.07 (dd, J_1 = 5.6, J_2 = 1.6 Hz, 1H), 7.03 (dd, J_1 = 5.6, J_2 = 2.0 Hz, 1H), 6.97 (t, J = 7.2 Hz, 1H), 6.93 (d, J = 6.4 Hz, 1H), 6.59 (dd, J_1 = 8.0, J_2 = 0.8 Hz, 1H), 5.95 (s, 1H), 5.70 (s, 1H), 3.83 (s, 3H) ppm.

Reaction of 1c with imidazole (2a) and furan (3a): 9.0 mg (7%) of compound **4c** as a colorless oil and 93.0 mg (72%) of compound **5c** as a colorless oil were obtained by following general procedure B in the absence of 18-c-6.

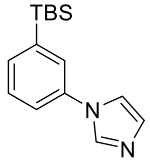

4c

Compound 4c: ^{1}H NMR (400 MHz, $CDCl_3$) δ 7.85 (s, 1H), 7.51 (d, J = 7.6 Hz, 1H), 7.48-7.44 (m, 1H), 7.39-7.34 (m, 1H), 7.28 (s, 1H), 7.22 (s, 1H), 0.90 (s, 9H), 0.31 (s, 6H) ppm; ^{13}C NMR (100 MHz, $CDCl_3$) δ 141.1, 136.9, 136.0, 133.9, 130.5, 129.1, 127.6, 122.4, 118.7, 26.6, 17.1, -6.0 ppm; IR (thin film) 3112, 3054, 2954, 2929, 2856, 1632, 1580, 1499, 1416, 1304, 1252, 1119, 1059, 830, 777, 698, 661 (cm^{-1}); HRMS (ESI) m/z: calcd for $[C_{15}H_{23}N_2Si]^+$ 259.1625; found 259.1636.

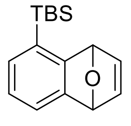

5c

Compound 5c: ^{1}H NMR (400 MHz, $CDCl_3$) δ 7.26 (d, J = 6.8 Hz, 1H), 7.09 (dd, J_1 = 8.0, J_2 = 1.2 Hz, 1H), 7.05-7.00 (m, 1H), 6.96 (t, J = 7.2 Hz, 1H), 5.83 (s, 1H), 5.70 (s, 1H), 0.93 (s, 9H), 0.34 (s, 3H), 0.33 (s, 3H) ppm; ^{13}C NMR (100 MHz, $CDCl_3$) δ 155.9, 147.8, 143.3, 143.1, 131.3, 129.8, 124.0, 121.0, 83.6, 82.3, 26.7, 17.4, -4.2, -4.8 ppm; IR (thin film) 3131, 3039, 2954, 2856, 2708, 1927, 1593, 1467, 1391, 1361, 1280, 1137, 1123, 1007, 892, 827, 710, 673, 578, 539, 478 (cm^{-1}); HRMS (ESI) m/z: calcd for $[C_{16}H_{23}OSi]^+$ 259.1513; found 259.1521.

Reaction of 1d with imidazole (2a) and furan (3a): 30.4 mg (34%) of compound **4d** and 10.7 mg (12%) of compound **5d** were obtained by following general procedure B.

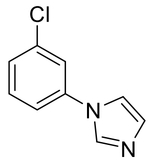

4d

Compound 4d: The ^{1}H NMR of **4d** is identical with that reported in literature.[15] ^{1}H NMR (400 MHz, $CDCl_3$) δ 7.86 (s, 1H), 7.46-7.38 (m, 2H), 7.35 (d, J = 7.6 Hz, 1H), 7.29 (d, J = 8.8 Hz, 2H), 7.21 (s, 1H) ppm.

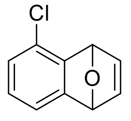

5d

Compound 5d: The ^{1}H NMR of **5d** is identical with that reported in literature.[16] ^{1}H NMR (400 MHz, $CDCl_3$) δ 7.13 (t, J = 3.6 Hz, 1H), 7.08 (dd, J_1 = 5.6, J_2 = 1.6 Hz, 1H), 7.06 (dd, J_1 = 5.6, J_2 = 2.0 Hz, 1H), 6.92 (d, J = 4.0 Hz, 2H), 5.88 (s, 1H), 5.75 (s, 1H) ppm.

Reaction of 1e with imidazole (2a) and furan (3a): 22.7 mg (21%) of compound **4e** and **4e'** as a 3: 1 mixture of regioisomers and 46.5 mg (43%) of compound **5e** were obtained by following general procedure B in the absence of 18-c-6.

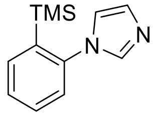

4e

Compound 4e: ^{1}H NMR (400 MHz, $CDCl_3$) δ 7.64-7.61 (m, 1H), 7.58 (s, 1H), 7.46-7.42 (m, 2H), 7.21-7.17 (m, 1H), 7.16 (s, 1H), 7.05 (t, J = 1.2 Hz, 1H), 0.06 (s, 9H) ppm; ^{13}C NMR (100 MHz, $CDCl_3$) δ 142.7, 138.3, 137.7, 135.8, 130.2, 129.1, 128.6, 127.5, 121.9, 0.3 ppm; IR (thin film) 3432, 3105, 3051, 2954, 2896, 2346, 1590, 1546, 1493, 1433, 1309, 1253, 1117, 1075, 964, 842, 769, 724, 665, 618, 546, 468 (cm^{-1}); HRMS (ESI) m/z: calcd for $[C_{12}H_{17}N_2Si]^+$ 217.1156; found 217.1162.

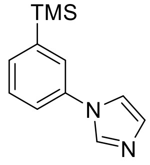

4e'

Compound 4e': ^{1}H NMR (400 MHz, $CDCl_3$) δ 7.90 (s, 1H), 7.53 (d, *J* = 7.2 Hz, 1H), 7.50-7.44 (m, 2H), 7.37-7.34 (m, 1H), 7.30 (s, 1H), 7.24 (s, 1H), 0.31 (s, 9H) ppm; ^{13}C NMR (100 MHz, $CDCl_3$) δ 143.7, 137.0, 136.0, 132.9, 130.2, 129.4, 126.5, 122.4, 118.8, -1.0 ppm; IR (thin film) 3429, 3120, 2956, 2368, 1578, 1498, 1402, 1304, 1249, 1110, 1063, 982, 847, 796, 753, 693, 659. 541, 472 (cm^{-1}); HRMS (ESI) m/z: calcd for $[C_{12}H_{17}N_2Si]^+$ 217.1156; found 217.1162.

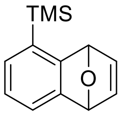

5e

Compound 5e: The ^{1}H NMR of **5e** is identical with that reported in literature.[17] ^{1}H NMR (400 MHz, $CDCl_3$) δ 7.25 (d, *J* = 7.2 Hz, 1H), 7.08 (d, *J* = 7.2 Hz, 1H), 7.03 (dd, J_1 = 5.6, J_2 = 1.6 Hz, 1H), 7.01 (dd, J_1 = 5.6, J_2 = 1.6 Hz, 1H), 6.95 (t, *J* = 7.2 Hz, 1H), 5.83 (s, 1H), 5.70 (s, 1H), 0.32 (s, 9H) ppm.

Reaction of 1f with imidazole (2a) and furan (3a): 35.0 mg (35%) of compound **4f** as a colorless oil and 27.0 mg (27%) of compound **5f** as a white solid were obtained by following general procedure B in the absence of 18-c-6.

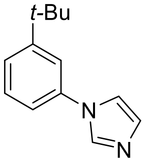

4f

Compound 4f: ^{1}H NMR (400 MHz, $CDCl_3$) δ 7.85 (s, 1H), 7.42-7.39 (m, 2H), 7.38-7.36 (m, 1H), 7.27 (s, 1H), 7.21-7.17 (m, 2H), 1.35 (s, 9H) ppm; ^{13}C NMR (100 MHz, $CDCl_3$) δ 153.7, 137.4, 136.0, 130.3, 129.7, 124.9, 119.1, 118.7, 35.1, 31.4 ppm; IR (thin film) 3419, 3113, 2962, 2868, 1608, 1589, 1501, 1364, 1307, 1250, 1108, 1059, 987, 793, 732, 701, 658, 549, 471 (cm^{-1}); HRMS (ESI) m/z: calcd for $[C_{13}H_{17}N_2]^+$ 201.1386; found 201.1390.

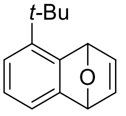

5f

Compound 5f: Mp: 49-51 °C; ^{1}H NMR (400 MHz, $CDCl_3$) δ 7.12 (d, *J* = 6.4 Hz, 1H), 7.04 (m, 2H), 6.98 (dd, J_1 = 8.0, J_2 = 1.2 Hz, 1H), 6.93 (dd, J_1 = 8.0, J_2 = 6.8 Hz, 1H), 6.15 (s, 1H), 5.68 (s, 1H), 1.37 (s, 9H) ppm; ^{13}C NMR (100 MHz, $CDCl_3$) δ 149.2, 146.7, 144.1, 143.8, 142.8, 125.2, 122.6, 118.4, 83.2, 82.1, 35.5, 31.5 ppm; IR (thin film) 3402, 3129, 3065, 2962, 2870, 1922, 1589, 1478, 1408, 1365, 1275, 1006, 879, 861, 834, 780, 714, 659, 619, 569, 492 (cm^{-1}); HRMS (ESI) m/z: calcd for $[C_{14}H_{17}O]^+$ 201.1274; found 201.1282.

Reaction of S1 with imidazole (2a) and furan (3a): 42.2 mg (52%) of compound **S9** and 6.5 mg (8%) of compound **S10** were obtained by following general procedure B.

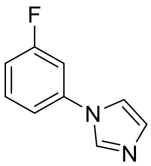

S9

Compound S9: The ^{1}H NMR of **S9** is identical with that reported in literature.[18] ^{1}H NMR (400 MHz, $CDCl_3$) δ 7.86 (s, 1H), 7.45 (dd, J_1 = 14.4, J_2 = 8.4 Hz, 1H), 7.27 (s, 1H), 7.22-7.17 (m, 2H), 7.16-7.04

(m, 2H) ppm.

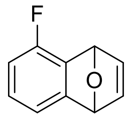

S10

Compound S10: The ^{1}H NMR of **S10** is identical with that reported in literature.[17] ^{1}H NMR (400 MHz, $CDCl_3$) δ 7.08-7.03 (m, 3H), 6.99-6.93 (m, 1H), 6.69 (t, J = 8.4 Hz, 1H), 5.97 (s, 1H), 5.74 (s, 1H) ppm.

Reaction of S2 with imidazole (2a) and furan (3a): 30.1 mg (27%) of compound **S11** and 14.5 mg (13%) of compound **S12** were obtained by following general procedure B.

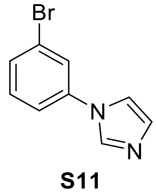

S11

Compound S11: The ^{1}H NMR of **S11** is identical with that reported in literature.[19] ^{1}H NMR (400 MHz, $CDCl_3$) δ 7.82 (s, 1H), 7.55 (s, 1H), 7.50 (dd, J_1 = 7.2, J_2 = 1.2 Hz, 1H), 7.38-7.30 (m, 2H), 7.25 (s, 1H), 7.19 (s, 1H) ppm.

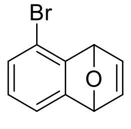

S12

Compound S12: The ^{1}H NMR of **S12** is identical with that reported in literature.[20] ^{1}H NMR (400 MHz, $CDCl_3$) δ 7.16 (d, J = 6.8 Hz, 1H), 7.09 (dd, J_1 = 5.6, J_2 = 1.6 Hz, 1H), 7.06 (dd, J_1 = 5.6, J_2 = 2.0 Hz, 2H), 6.85 (dd, J_1 = 8.0, J_2 = 7.2 Hz, 1H), 5.80 (s, 1H), 5.78 (s, 1H) ppm.

Reaction of S3 with imidazole (2a) and furan (3a): 19.4 mg (15%) of compound **S13** and **S13'** as a 2: 1 mixture of regioisomers and 64.6 mg (50%) of compound **S14** as a colorless oil were obtained by following general procedure B in the absence of 18-c-6.

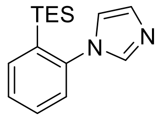

S13

Compound S13: ^{1}H NMR (400 MHz, $CDCl_3$) δ 7.61-7.57 (m, 1H), 7.56 (s, 1H), 7.47-7.42 (m, 2H), 7.22-7.17 (m, 1H), 7.16 (s, 1H), 7.04 (s, 1H), 0.85 (t, J = 7.6 Hz, 9H), 0.53 (q, J = 7.6 Hz, 6H) ppm; ^{13}C NMR (100 MHz, $CDCl_3$) δ 143.1, 138.3, 136.8, 134.7, 130.1, 129.2, 128.5, 127.8, 121.9, 7.6, 3.5 ppm; IR (thin film) 2953, 2913, 2874, 1636, 1589, 1492, 1460, 1434, 1384, 1308, 1294, 1235, 1115, 1073, 1056, 1003, 963, 815, 768, 731, 664, 574, 471 (cm^{-1}); HRMS (ESI) m/z: calcd for $[C_{15}H_{23}N_2Si]^+$ 259.1625; found 259.1629.

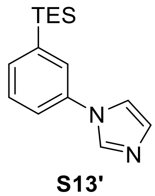

S13'

Compound S13': ^{1}H NMR (400 MHz, $CDCl_3$) δ 7.85 (s, 1H), 7.50-7.44 (m, 3H), 7.38-7.32 (dt, J_1 = 7.2, J_2 = 2.0 Hz, 1H), 7.28 (s, 1H), 7.21 (s, 1H), 1.00-0.95 (m, 9H), 0.86-0.79 (m, 6H) ppm; ^{13}C NMR (100 MHz, $CDCl_3$) δ 140.7, 137.1, 136.0, 133.6, 130.5, 129.3, 127.2, 122.3, 118.7, 7.5, 3.5 ppm; IR (thin film) 3114, 2954, 2909, 2876, 1632, 1578, 1497, 1462, 1417, 1384, 1304, 1244, 1119, 1058, 1011, 981, 906, 792, 729, 698, 660, 626, 587, 437 (cm^{-1}); HRMS (ESI) m/z: calcd for $[C_{15}H_{23}N_2Si]^+$ 259.1625; found 259.1636.

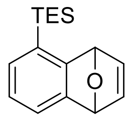

S14

Compound S14: ^{1}H NMR (400 MHz, $CDCl_3$) δ 7.25 (d, J = 6.8 Hz, 1H), 7.06 (dd, J_1 = 7.6, J_2 = 0.8 Hz, 1H), 7.03 (dd, J_1 = 5.6, J_2 = 2.0 Hz, 1H), 7.00 (dd, J_1 = 5.6, J_2 = 2.0 Hz, 1H), 6.95 (t, J = 7.2 Hz, 1H), 5.79 (s, 1H), 5.69 (s, 1H), 1.01-0.93 (m, 9H), 0.87-0.78 (m, 6H) ppm; ^{13}C NMR (100 MHz, $CDCl_3$) δ 155.7, 147.8, 143.4, 143.0, 130.9, 129.4, 124.2, 121.0, 83.1, 82.3, 7.6, 4.1 ppm; IR (thin film) 2953, 2913, 1462, 1416, 1392, 1279, 1236, 1137, 1007, 875, 850, 807, 784, 774, 752, 647, 620, 600, 539, 481 (cm^{-1}); HRMS (ESI) m/z: calcd for $[C_{16}H_{22}NaOSi]^+$ 281.1332; found 281.1344.

Reaction of S4 with imidazole (2a) and furan (3a): 15.0 mg (10%) of compound **S15** as a colorless oil and 87.1 mg (58%) of compound **S16** as a colorless oil were obtained by following general procedure B in the absence of 18-c-6.

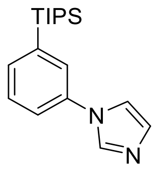

S15

Compound S15: ^{1}H NMR (400 MHz, $CDCl_3$) δ 7.96 (s, 1H), 7.52 (d, J = 7.2 Hz, 1H), 7.50-7.46 (m, 2H), 7.37 (d, J = 7.6 Hz, 1H), 7.29 (s, 1H), 7.27 (s, 1H), 1.49-1.36 (m, 3H), 1.10 (s, 9H), 1.08 (s, 9H) ppm; ^{13}C NMR (100 MHz, $CDCl_3$) δ 138.5, 136.7, 136.2, 135.0, 130.0, 129.2, 128.3, 122.3, 119.0, 18.7, 10.9 ppm; IR (thin film) 3429, 3129, 2945, 2864, 2378, 1627, 1498, 1483, 1391, 1302, 1114, 993, 858, 802, 660, 542, 513, 474 (cm^{-1}); HRMS (ESI) m/z: calcd for $[C_{18}H_{29}N_2Si]^+$ 301.2095; found 301.2101.

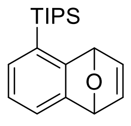

S16

Compound S16: ^{1}H NMR (400 MHz, $CDCl_3$) δ 7.25 (d, J = 6.8 Hz, 1H), 7.09 (dd, J_1 = 7.6, J_2 = 1.2 Hz, 1H), 7.04-7.00 (m, 2H), 6.95 (t, J = 7.2 Hz, 1H), 5.79 (s, 1H), 5.68 (s, 1H), 1.49-1.39 (m, 3H), 1.13 (d, J = 7.6 Hz, 9H), 1.05 (d, J = 7.6 Hz, 9H) ppm; ^{13}C NMR (100 MHz, $CDCl_3$) δ 156.3, 148.1, 143.2, 143.0, 132.1, 127.3, 124.0, 120.8, 83.9, 82.3, 18.9, 18.8, 12.0 ppm; IR (thin film) 3045, 3019, 2946, 2862, 1461, 1384, 1279, 1238, 1129, 1074, 1038, 1004, 921, 879, 849, 785, 753, 713, 676, 642, 588, 562, 509 (cm^{-1}); HRMS (ESI) m/z: calcd for $[C_{19}H_{29}OSi]^+$ 301.1982; found 301.1992.

Reaction of 1a with *N*-tosylated aniline (2b) and furan (3a): 81.0 mg (48%) of compound **S17** and 18.0 mg (25%) of compound **5a** were obtained by following general procedure B.

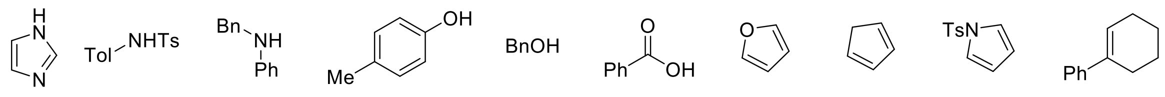

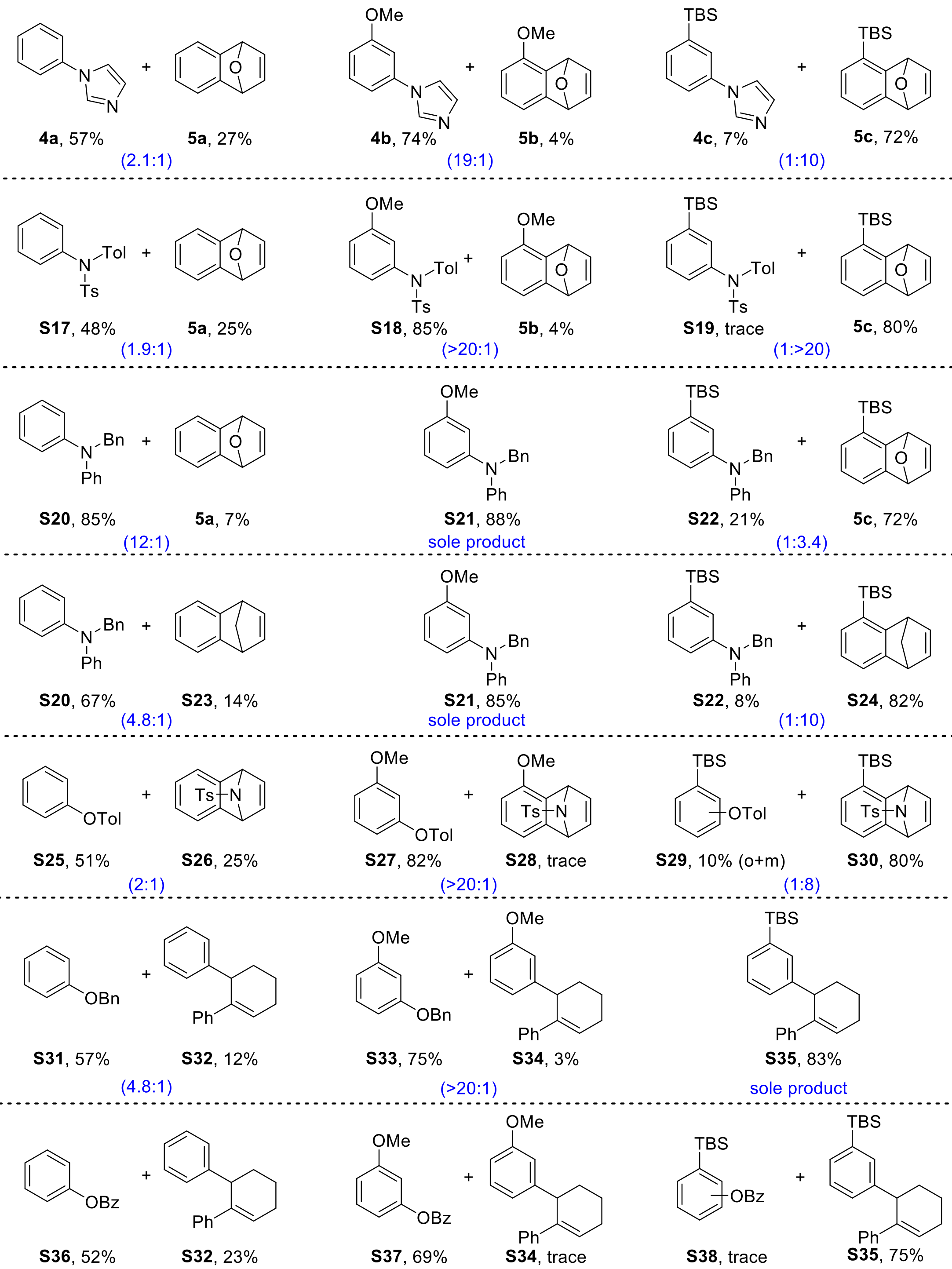

Entry	Trapping agents	Reaction with 1a	Reaction with 1b	Reaction with 1c
1	2a + 3a	**4a**, 57% + **5a**, 27% (2.1:1)	**4b**, 74% + **5b**, 4% (19:1)	**4c**, 7% + **5c**, 72% (1:10)
2	2b + 3a	**S17**, 48% + **5a**, 25% (1.9:1)	**S18**, 85% + **5b**, 4% (>20:1)	**S19**, trace + **5c**, 80% (1:>20)
3	2c + 3a	**S20**, 85% + **5a**, 7% (12:1)	**S21**, 88% sole product	**S22**, 21% + **5c**, 72% (1:3.4)
4	2c + 3b	**S20**, 67% + **S23**, 14% (4.8:1)	**S21**, 85% sole product	**S22**, 8% + **S24**, 82% (1:10)
5	2d + 3c	**S25**, 51% + **S26**, 25% (2:1)	**S27**, 82% + **S28**, trace (>20:1)	**S29**, 10% (o+m) + **S30**, 80% (1:8)
6	2e + 3d	**S31**, 57% + **S32**, 12% (4.8:1)	**S33**, 75% + **S34**, 3% (>20:1)	**S35**, 83% sole product
7	2f + 3d	**S36**, 52% + **S32**, 23% (2.3:1)	**S37**, 69% + **S34**, trace (>20:1)	**S38**, trace + **S35**, 75% (1:>20)

Supplementary Table 2. Intermolecular aryne trapping reactions with various arynophiles.

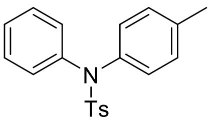

S17

Compound S17: The ^{1}H NMR of **S17** is identical with that reported in literature.[21] ^{1}H NMR (400 MHz, $CDCl_3$) δ 7.58 (d, *J* = 8.4 Hz, 2H), 7.30-7.27 (m, 5H), 7.25-7.23 (m, 2H), 7.15 (d, *J* = 8.4 Hz, 2H), 7.11 (d, *J* = 8.4 Hz, 2H), 2.43 (s, 3H), 2.32 (s, 3H) ppm.

Reaction of 1b with *N*-tosylated aniline (2b) and furan (3a): 156.2 mg (85%) of compound **S18** as a white solid and 3.5 mg (4%) of compound **5b** were obtained by following general procedure B (18-c-6, CsF and Cs_2CO_3 (489 mg, 1.5 mmol, 3.0 equiv) in MeCN (10.0 mL) was used instead of 18-c-6, and CsF in MeCN).

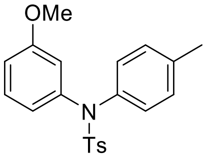

S18

Compound S18: Mp: 66-68 °C; ^{1}H NMR (400 MHz, $CDCl_3$) δ 7.60 (d, *J* = 7.6 Hz, 2H), 7.27 (d, *J* = 8.0 Hz, 2H), 7.19 (d, *J* = 8.0 Hz, 1H), 7.20-7.09 (m, 5H), 6.88-6.80 (m, 2H), 6.77 (dd, J_1 = 8.0, J_2 = 2.0 Hz, 1H), 3.74 (s, 3H), 2.43 (s, 3H), 2.32 (s, 3H) ppm; ^{13}C NMR (100 MHz, $CDCl_3$) δ 160.2, 143.7, 143.1, 139.0, 137.9, 137.8, 130.1, 129.9, 129.7, 128.7, 128.0, 120.2, 114.0, 112.9, 55.5, 21.8, 21.3 ppm; IR (thin film) 3030, 2925, 2822, 1920, 1601, 1508, 1487, 1353, 1288, 1230, 1163, 1091, 1043, 983, 939, 813, 776, 679, 650, 587, 546, 513 (cm^{-1}); HRMS (ESI) m/z: calcd for $[C_{21}H_{21}NNaO_3S]^+$ 390.1134; found 390.1144.

Reaction of 1c with *N*-tosylated aniline (2b) and furan (3a): trace amount of compound **S19** as a colorless oil and 103.4 mg (80%) of compound **5c** were obtained by following general procedure C.

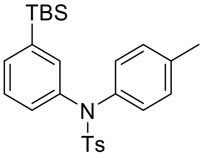

S19

Compound S19: ^{1}H NMR (400 MHz, $CDCl_3$) δ 7.57 (d, *J* = 8.4 Hz, 2H), 7.37-7.34 (m, 1H), 7.30 (d, *J* = 2.0 Hz, 1H), 7.28 (s, 1H), 7.24 (s, 1H), 7.17-7.13 (m, 2H), 7.11 (d, *J* = 8.4 Hz, 2H), 2.43 (s, 3H), 2.32 (s, 3H), 0.81 (s, 9H), 0.18 (s, 6H) ppm; ^{13}C NMR (100 MHz, $CDCl_3$) δ 143.6, 141.2, 139.5, 139.2, 138.1, 137.6, 133.9, 133.5, 130.1, 129.7, 129.1, 128.6, 128.5, 128.1, 26.6, 21.8, 21.3 17.1, -6.1 ppm; IR (thin film) 3060, 2953, 2926, 2855, 1599, 1507, 1470, 1397, 1357, 1258, 1165, 1092, 979, 937, 826, 772, 712, 677, 590, 550 (cm^{-1}); HRMS (ESI) m/z: calcd for $[C_{26}H_{33}NNaO_2SSi]^+$ 474.1893; found 474.1905.

Reaction of 1a with *N*-benzyl aniline (2c) and furan (3a): 110.2 mg (85%) of compound **S20** as a white solid and 5.0 mg (7%) of compound **5a** were obtained by following general procedure B.

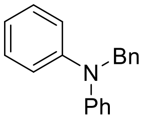

S20

Compound S20: The ^{1}H NMR of **S20** is identical with that reported in literature.[22] ^{1}H NMR (400 MHz, $CDCl_3$) δ 7.37-7.27 (m, 4H), 7.26-7.19 (m, 5H), 7.10-7.04 (m, 4H), 6.96-6.90 (m, 2H), 5.00 (s, 2H) ppm.

Reaction of 1b with *N*-benzyl aniline (2c) and furan (3a): 127.3 mg (88%) of compound **S21** as a

colorless oil was obtained solely by following general procedure B.

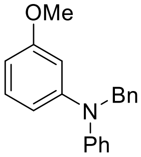

S21

Compound S21: ^{1}H NMR (400 MHz, $CDCl_3$) δ 7.37-7.20 (m, 7H), 7.16-7.11 (m, 3H), 6.98 (t, *J* = 7.6 Hz, 1H), 6.64 (d, *J* = 8.0 Hz, 1H), 6.59 (d, *J* = 2.0 Hz, 1H), 6.48 (d, *J* = 8.0 Hz, 1H), 5.00 (s, 2H), 3.72 (s, 3H) ppm; ^{13}C NMR (100 MHz, $CDCl_3$) δ 160.7, 149.6, 148.1, 139.4, 130.0, 129.5, 128.8, 127.0, 126.7, 122.2, 122.0, 112.7, 106.2, 56.6, 55.4 ppm; IR (thin film) 3435, 3082, 2948, 2832, 1589, 1491, 1356, 1204, 1166, 1010, 989, 836, 776, 699, 593, 452 (cm^{-1}); HRMS (ESI) m/z: calcd for $[C_{20}H_{20}NO]^+$ 290.1539; found 290.1548.

Reaction of 1c with *N*-benzyl aniline (2c) and furan (3a): 39.2 mg (21%) of compound **S22** as a colorless oil and 93.0 mg (72%) of compound **5c** were obtained by following general procedure C.

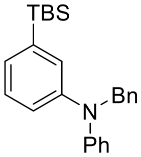

S22

Compound S22: ^{1}H NMR (400 MHz, $CDCl_3$) δ 7.35 (d, *J* = 6.8 Hz, 2H), 7.29 (t, *J* = 7.2 Hz, 2H), 7.25-7.18 (m, 5H), 7.12-7.08 (m, 2H), 7.05-7.01 (m, 2H), 6.90 (t, *J* = 7.2 Hz, 1H), 5.00 (s, 2H), 0.82 (s, 9H), 0.19 (s, 6H) ppm; ^{13}C NMR (100 MHz, $CDCl_3$) δ 148.5, 147.1, 139.5, 139.1, 129.4, 128.7, 128.6, 128.2, 128.0, 127.0, 126.8, 122.4, 120.9, 119.8, 56.6, 26.7, 17.1, -6.1 ppm; IR (thin film) 3433, 2953, 2854, 1599, 1496, 1407, 1248, 1091, 1009, 910, 833, 810, 772, 696, 662, 575, 458 (cm^{-1}); HRMS (ESI) m/z: calcd for $[C_{25}H_{32}NSi]^+$ 374.2299; found 374.2297.

Reaction of 1a with *N*-benzyl aniline (2c) and cyclopentadiene (3b): 86.9 mg (67%) of compound **S20** as a white solid and 10.0 mg (14%) of compound **S23** were obtained by following general procedure B.

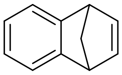

S23

Compound S23: The ^{1}H NMR of **S23** is identical with that reported in literature.[23] ^{1}H NMR (400 MHz, $CDCl_3$) δ 7.26-7.21 (m, 2H), 6.99-6.91 (m, 2H), 6.81 (t, *J* = 1.6 Hz, 2H), 3.94-3.87 (m, 2H), 2.33 (d, *J* = 7.2 Hz, 1H), 2.26 (d, *J* = 7.2 Hz, 1H) ppm.

Reaction of 1b with *N*-benzyl aniline (2c) and cyclopentadiene (3b): 123.0 mg (85%) of compound **S21** as a colorless oil was obtained solely by following general procedure B.

Reaction of 1c with *N*-benzyl aniline (2c) and cyclopentadiene (3b): 14.9 mg (8%) of compound **S22** as a colorless oil and 105.2 mg (82%) of compound **S24** as a colorless oil were obtained by following general procedure C.

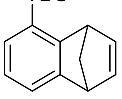

S24

Compound S24: ^{1}H NMR (400 MHz, $CDCl_3$) δ 7.23 (d, *J* = 7.2 Hz, 1H), 7.06 (dd, J_1 = 7.6, J_2 = 0.8 Hz,

1H), 6.92 (t, J = 7.2 Hz, 1H), 6.80 (dd, J_1 = 5.2, J_2 = 2.8 Hz, 1H), 6.76 (dd, J_1 = 5.2, J_2 = 2.8 Hz, 1H), 4.06 (s, 1H), 3.86 (s, 1H), 2.30 (dt, J_1 = 7.2, J_2 = 1.6 Hz, 1H), 2.20 (d, J = 6.8 Hz, 1H), 0.92 (s, 9H), 0.35 (s, 3H), 0.33 (s, 3H) ppm; ^{13}C NMR (100 MHz, $CDCl_3$) δ 158.6, 150.9, 143.5, 143.0, 130.9, 130.4, 123.3, 122.5, 69.5, 51.8, 50.2, 26.9, 17.6, -3.9, -4.3 ppm; IR (thin film) 3803, 3612, 3429, 3132, 3057, 2954, 2888, 2856, 1613, 1462, 1392, 1360, 1303, 1251, 1121, 1094, 1007, 829, 767, 719, 672, 540, 466 (cm^{-1}); HRMS (ESI) m/z: calcd for $[C_{17}H_{25}Si]^+$ 257.1720; found 257.1716.

Reaction of 1a with phenol (2d) and *N*-tosyl pyrrole (3c): 47.0 mg (51%) of compound **S25** and 37.2 mg (25%) of compound **S26** were obtained by following general procedure B.

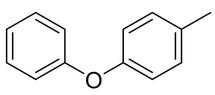

S25

Compound S25: The ^{1}H NMR of **S25** is identical with that reported in literature.[24] ^{1}H NMR (400 MHz, $CDCl_3$) δ 7.32 (t, J = 7.6 Hz, 2H), 7.15 (d, J = 8.0 Hz, 2H), 7.08 (t, J = 7.6 Hz, 1H), 6.99 (d, J = 8.0 Hz, 2H), 6.93 (d, J = 8.4 Hz, 2H), 2.35 (s, 3H) ppm.

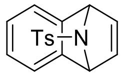

S26

Compound S26: The ^{1}H NMR of **S26** is identical with that reported in literature.[25] ^{1}H NMR (400 MHz, $CDCl_3$) δ 7.46 (d, J = 8.0 Hz, 2H), 7.09 (d, J = 8.0 Hz, 2H), 7.03 (t, J = 3.2 Hz, 2H), 6.78 (s, 4H), 5.45 (s, 2H), 2.34 (s, 3H) ppm.

Reaction of 1b with phenol (2d) and *N*-tosyl pyrrole (3c): 87.8 mg (82%) of compound **S27** and trace amount of compound **S28** as a white solid were obtained by following general procedure B (18-c-6, CsF and K_2CO_3 (414 mg, 3.0 mmol, 6.0 equiv) was used instead of 18-c-6, and CsF).

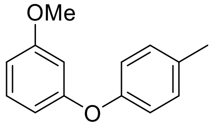

S27

Compound S27: The ^{1}H NMR of **S27** is identical with that reported in literature.[26] ^{1}H NMR (400 MHz, $CDCl_3$) δ 7.21 (t, J = 8.4 Hz, 1H), 7.15 (d, J = 8.4 Hz, 2H), 6.94 (d, J = 8.4 Hz, 2H), 6.63 (dd, J_1 = 8.4, J_2 = 1.6 Hz, 1H), 6.57-6.54 (m, 2H), 3.78 (s, 3H), 2.35 (s, 3H) ppm.

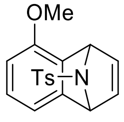

S28

Compound S28: Mp: 119-121 °C; ^{1}H NMR (400 MHz, $CDCl_3$) δ 7.43 (d, J = 8.4 Hz, 2H), 7.04 (d, J = 8.0 Hz, 2H), 6.89-6.86 (m, 1H), 6.85-6.83 (m, 1H), 6.76-6.69 (m, 2H), 6.29 (dd, J_1 = 7.6, J_2 = 1.2 Hz, 1H), 5.63 (s, 1H), 5.43 (s, 1H), 3.70 (s, 3H), 2.33 (s, 3H) ppm; ^{13}C NMR (100 MHz, $CDCl_3$) δ 153.2, 149.4, 143.1, 142.8, 142.6, 135.1, 134.0, 129.3, 128.4, 127.2, 114.8, 109.7, 68.3, 64.8, 55.5, 21.7 ppm; IR (thin film) 3064, 3005, 2927, 2842, 1616, 1481, 1336, 1269, 1154, 1094, 929, 797, 765, 683, 654, 597, 544, 484 (cm^{-1}); HRMS (ESI) m/z: calcd for $[C_{18}H_{17}NNaO_3S]^+$ 350.0821; found 350.0837.

Reaction of 1c with phenol (2d) and *N*-tosyl pyrrole (3c): 14.9 mg (10%) of compound **S29** and **S29'** as a 1: 5 mixture of regioisomers and 164.7 mg (80%) of compound **S30** as a white solid were obtained by following general procedure B (18-c-6 and KF (174 mg, 3.0 mmol, 6.0 equiv) in THF (5.0 mL) was used instead of 18-c-6, and CsF in MeCN).

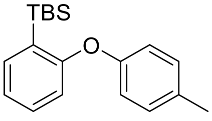

S29

Compound S29: ^{1}H NMR (400 MHz, $CDCl_3$) δ 7.47 (dd, J_1 = 7.6, J_2 = 2.0 Hz, 1H), 7.26-7.22 (m, 1H), 7.13 (d, J = 8.0 Hz, 2H), 7.04 (td, J_1 = 7.2, J_2 = 0.8 Hz, 1H), 6.89 (d, J = 8.4 Hz, 2H), 6.74 (d, J = 8.0 Hz, 1H), 2.33 (s, 3H), 0.93 (s, 9H), 0.28 (s, 6H) ppm; ^{13}C NMR (100 MHz, $CDCl_3$) δ 162.8, 154.7, 137.0, 132.8, 130.6, 130.4, 127.8, 122.3, 119.4, 116.5, 27.3, 20.9, 17.9, -4.4 ppm; IR (thin film) 2953, 2926, 2855, 1612, 1566, 1506, 1469, 1433, 1360, 1229, 1121, 836, 823, 811, 771, 739, 685, 659, 493 (cm^{-1}); HRMS (ESI) m/z: calcd for $[C_{19}H_{27}OSi]^+$ 299.1826; found 299.1826.

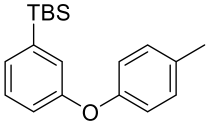

S29'

Compound S29': ^{1}H NMR (400 MHz, $CDCl_3$) δ 7.29 (t, J = 7.2 Hz, 1H), 7.22 (d, J = 7.2 Hz, 1H), 7.19 (d, J = 2.4 Hz, 1H), 7.13 (d, J = 8.0 Hz, 2H), 6.97-6.87 (m, 3H), 2.33 (s, 3H), 0.87 (s, 9H), 0.25 (s, 6H) ppm; ^{13}C NMR (100 MHz, $CDCl_3$) δ 156.9, 155.3, 140.3, 132.7, 130.4, 129.3, 129.0, 124.9, 119.0, 118.8, 26.7, 20.9, 17.1, -6.0 ppm; IR (thin film) 3058, 3031, 2954, 2929, 2889, 2857, 1570, 1505, 1471, 1404, 1361, 1110, 1010, 909, 829, 772, 694, 629, 577, 487 (cm^{-1}); HRMS (ESI) m/z: calcd for $[C_{19}H_{27}OSi]^+$ 299.1826; found 299.1837.

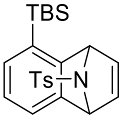

S30

Compound S30: Mp: 125-127 °C; ^{1}H NMR (400 MHz, $CDCl_3$) δ 7.52 (d, J = 8.4 Hz, 2H), 7.13 (d, J = 8.0 Hz, 2H), 7.07 (d, J = 6.8 Hz, 1H), 6.94 (dd, J_1 = 7.6, J_2 = 0.8 Hz, 1H), 6.80 (t, J = 7.2 Hz, 1H), 6.76-6.70 (m, 2H), 5.55 (s, 1H), 5.44 (s, 1H), 2.36 (s, 3H), 0.85 (s, 9H), 0.35 (s, 3H), 0.27 (s, 3H) ppm; ^{13}C NMR (100 MHz, $CDCl_3$) δ 154.1, 146.7, 143.5, 142.4, 142.2, 135.9, 131.4, 130.4, 129.6, 128.4, 124.0, 121.7, 68.9, 67.5, 26.6, 21.7, 17.3, -4.3, -4.5 ppm; IR (thin film) 3089, 3047, 2957, 2930, 2889, 2855, 1925, 1597, 1463, 1390, 1339, 1285, 1255, 1157, 1090, 1017, 939, 827, 771, 697, 610, 550, 504 (cm^{-1}); HRMS (ESI) m/z: calcd for $[C_{23}H_{29}NNaO_2SSi]^+$ 434.1580; found 434.1590.

Reaction of 1a with benzyl alcohol (2e) and 1-phenylcyclohexene (3d): 52.5 mg (57%) of compound **S31** and 14.1 mg (12%) of compound **S32** were obtained by following general procedure B.

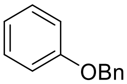

S31

Compound S31: The ^{1}H NMR of **S31** is identical with that reported in literature.[27] ^{1}H NMR (400 MHz, $CDCl_3$) δ 7.46 (d, J = 7.2 Hz, 2H), 7.40 (t, J = 7.2 Hz, 2H), 7.36-7.27 (m, 3H), 7.05-6.95 (m, 3H), 5.08 (s, 2H) ppm.

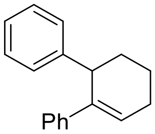

S32

Compound S32: The ^{1}H NMR of **S32** is identical with that reported in literature.[28] ^{1}H NMR (400 MHz,

$CDCl_3$) δ 7.27 (d, *J* = 7.6 Hz, 2H), 7.24-7.18 (m, 4H), 7.16 (t, *J* = 7.6 Hz, 2H), 7.13-7.06 (m, 2H), 6.39 (t, *J* = 4.0 Hz, 1H), 4.01 (s, 1H), 2.37-2.26 (m, 2H), 2.13-2.01 (m, 1H), 1.89-1.80 (m, 1H), 1.61-1.49 (m, 2H) ppm.

Reaction of 1b with benzyl alcohol (2e) and 1-phenylcyclohexene (3d): 80.3 mg (75%) of compound **S33** and 4.0 mg (3%) of compound **S34** as a colorless oil were obtained by following general procedure B (18-c-6 and KF (87.2 mg, 1.5 mmol, 3.0 equiv) in THF (5.0 mL) at -30 °C was used instead of 18-c-6, and CsF in MeCN at room temperature).

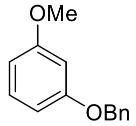
S33

Compound S33: The ^{1}H NMR of **S33** is identical with that reported in literature.[29] ^{1}H NMR (400 MHz, $CDCl_3$) δ 7.45 (d, *J* = 7.2 Hz, 2H), 7.40 (t, *J* = 7.2 Hz, 2H), 7.34 (t, *J* = 6.8 Hz, 1H), 7.20 (t, *J* = 8.0 Hz, 1H), 6.60 (d, *J* = 8.0 Hz, 1H), 6.57 (t, *J* = 2.4 Hz, 1H), 6.55 (d, *J* = 8.0 Hz, 1H), 5.06 (s, 2H), 3.80 (s, 3H) ppm.

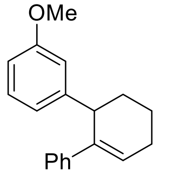
S34

Compound S34: ^{1}H NMR (400 MHz, $CDCl_3$) δ 7.31-7.27 (m, 2H), 7.22-7.16 (m, 2H), 7.16-7.13 (m, 1H), 7.12-7.07 (m, 1H), 6.82 (d, *J* = 7.6 Hz, 1H), 6.79-6.75 (m, 1H), 6.70-6.65 (m, 1H), 6.39 (t, *J* = 3.6 Hz, 1H), 3.99 (brs, 1H), 3.75 (s, 3H), 2.39-2.25 (m, 2H), 2.12-2.03 (m, 1H), 1.90-1.82 (m, 1H), 1.64-1.55 (m, 2H) ppm; ^{13}C NMR (100 MHz, $CDCl_3$) δ 159.6, 147.1, 142.1, 138.0, 129.2, 128.3, 128.1, 126.6, 126.0, 121.4, 114.9, 110.9, 55.3, 42.8, 32.7, 26.3, 17.7 ppm; IR (thin film) 3422, 2933, 2831, 1607, 1599, 1582, 1484, 1445, 1313, 1262, 1134, 1083, 1055, 995, 874, 762, 698, 541, 468 (cm^{-1}); HRMS (ESI) m/z: calcd for $[C_{19}H_{20}NaO]^+$ 287.1406; found 287.1416.

Reaction of 1c with benzyl alcohol (2e) and 1-phenylcyclohexene (3d): 144.7 mg (83%) of compound **S35** as a colorless oil was obtained solely by following general procedure C.

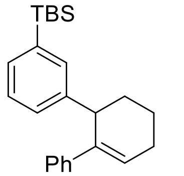
S35

Compound S35: ^{1}H NMR (400 MHz, $CDCl_3$) δ 7.31 (s, 1H), 7.26-7.21 (m, 3H), 7.21-7.17 (m, 2H), 7.16-7.11 (m, 2H), 7.09-7.04 (m, 1H), 6.35 (t, *J* = 3.6 Hz, 1H), 4.02 (brs, 1H), 2.37-2.24 (m, 2H), 2.14-2.05 (m, 1H), 1.87-1.80 (m, 1H), 1.62-1.56 (m, 2H), 0.78 (s, 9H), 0.22 (s, 3H), 0.20 (s, 3H) ppm; ^{13}C NMR (100 MHz, $CDCl_3$) δ 144.0, 142.3, 138.5, 137.3, 135.0, 132.0, 129.3, 128.2, 128.1, 127.3, 126.5, 126.2, 43.0, 33.0, 26.6, 26.3, 18.0, 17.1, -6.1 ppm; IR (thin film) 3424, 2952, 2927, 2854, 2361, 1638, 1493, 1470, 1403, 1361, 1248, 1117, 1005, 832, 787, 756, 695, 540, 474 (cm^{-1}); HRMS (ESI) m/z: calcd for $[C_{24}H_{32}NaSi]^+$ 371.2165; found 371.2173.

Reaction of 1a with benzoic acid (2f) and 1-phenylcyclohexene (3d): 51.5 mg (52%) of compound **S36** and 26.9 mg (23%) of compound **S32** were obtained by following general procedure B.

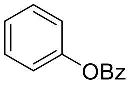

S36

Compound S36: The ^{1}H NMR of **S36** is identical with that reported in literature.[30] ^{1}H NMR (400 MHz, $CDCl_3$) δ 8.23 (d, *J* = 8.0 Hz, 2H), 7.65 (t, *J* = 7.6 Hz, 1H), 7.53 (t, *J* = 7.6 Hz, 2H), 7.45 (t, *J* = 8.0 Hz, 2H), 7.29 (t, *J* = 7.2 Hz, 1H), 7.24 (t, *J* = 6.4 Hz, 1H) ppm.

Reaction of 1b with benzoic acid (2f) and 1-phenylcyclohexene (3d): 78.7 mg (69%) of compound **S37** and trace amount of compound **S34** were obtained by following general procedure B (the amount of **1b**: **2f**: **3d** = 1: 1: 1 was used instead of 1: 3: 3).

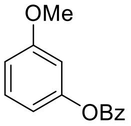

S37

Compound S37: The ^{1}H NMR of **S37** is identical with that reported in literature.[31] ^{1}H NMR (400 MHz, $CDCl_3$) δ 8.21 (d, *J* = 7.6 Hz, 2H), 7.65 (t, *J* = 7.2 Hz, 1H), 7.52 (t, *J* = 7.6 Hz, 2H), 7.33 (t, *J* = 8.4 Hz, 1H), 6.85-6.81 (m, 2H), 6.78 (s, 1H), 3.83 (s, 3H) ppm.

Reaction of 1c with benzoic acid (2f) and 1-phenylcyclohexene (3d): trace amount of compound **S38** as a colorless oil, trace amount of compound **S38'** as a white solid, and 130.7 mg (75%) of compound **S35** were obtained by following general procedure C.

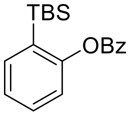

S38

Compound S38: ^{1}H NMR (400 MHz, $CDCl_3$) δ 8.23 (d, *J* = 7.2 Hz, 2H), 7.64 (t, *J* = 7.6 Hz, 1H), 7.52 (t, *J* = 8.0 Hz, 2H), 7.45-7.40 (m, 2H), 7.32 (d, *J* = 2.4 Hz, 1H), 7.24-7.19 (m, 1H), 0.90 (s, 9H), 0.30 (s, 6H) ppm; ^{13}C NMR (100 MHz, $CDCl_3$) δ 165.5, 150.6, 140.3, 133.7, 132.1, 130.4, 130.0, 128.8, 128.7, 127.4, 122.3, 26.7, 17.1, -6.0 ppm; IR (thin film) 3063, 3018, 2954, 2929, 2856, 1739, 1570, 1470, 1402, 1361, 1263, 1197, 1112, 1063, 1025, 882, 830, 772, 704, 577 (cm^{-1}); HRMS (ESI) m/z: calcd for $[C_{19}H_{24}NaO_2Si]^+$ 335.1438; found 335.1450.

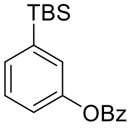

S38'

Compound S38': Mp: 75-77 °C; ^{1}H NMR (400 MHz, $CDCl_3$) δ 8.20 (d, *J* = 8.0 Hz, 2H), 7.65 (t, *J* = 7.2 Hz, 1H), 7.56-7.49 (m, 3H), 7.47-7.41 (m, 1H), 7.29-7.24 (m, 1H), 7.11 (d, *J* = 8.0 Hz, 1H), 0.90 (s, 9H), 0.23 (s, 6H) ppm; ^{13}C NMR (100 MHz, $CDCl_3$) δ 166.0, 156.1, 136.9, 133.7, 130.6, 130.4, 130.2, 129.3, 128.8, 125.4, 122.7, 26.8, 17.7, -4.6 ppm; IR (thin film) 3066, 3000, 2952, 2928, 2854, 1732, 1593, 1470, 1426, 1312, 1258, 1174, 1122, 1082, 1059, 1022, 830, 774, 752, 705, 581, 459 (cm^{-1}); HRMS (ESI) m/z: calcd for $[C_{19}H_{24}NaO_2Si]^+$ 335.1438; found 335.1445.

2.4 Reactions of aryne precursors with substrates

Reaction of 1a with compound sub-1:

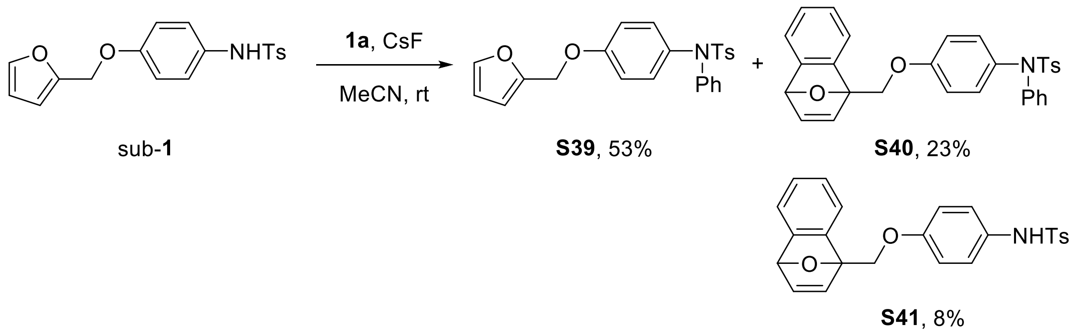

A mixture of aryne precursor **1a** (65.6 mg, 0.22 mmol, 1.1 equiv), compound sub-**1** (68.7 mg, 0.2 mmol, 1.0 equiv), and CsF (91.1 mg, 0.6 mmol, 3.0 equiv) in anhydrous MeCN (2.0 mL) under inert atmosphere was stirred at room temperature overnight. The resulting mixture was filtered through a short pad of silica gel (EtOAc eluent, 20 mL). All the volatiles were removed on a rotary evaporator. Flash column chromatography (pet ether: DCM = 2: 1 to 1: 6) afforded 44.5 mg (53%) of compound **S39** as a white solid, 22.8 mg (23%) of compound **S40** as a white solid, and 6.7 mg (8%) of compound **S41** as a white solid.

Compound S39: Mp: 151-153 °C; ^{1}H NMR (400 MHz, $CDCl_3$) δ 7.57 (d, J = 8.4 Hz, 2H), 7.44 (dd, J_1 = 2.0, J_2 = 0.8 Hz, 1H), 7.32-7.26 (m, 5H), 7.25-7.22 (m, 2H), 7.19 (d, J = 8.8 Hz, 2H), 6.91 (d, J = 9.2 Hz, 2H), 6.42 (d, J = 3.2 Hz, 1H), 6.38 (dd, J_1 = 3.2, J_2 = 2.0 Hz, 1H), 4.97 (s, 2H), 2.44 (s, 3H) ppm; ^{13}C NMR (100 MHz, $CDCl_3$) δ 157.9, 150.1, 143.7, 143.4, 142.1, 137.8, 134.7, 130.4, 129.7, 129.4, 128.1, 128.0, 127.3, 115.6, 110.8, 110.4, 62.8, 21.8 ppm; IR (thin film) 3125, 2923, 2855, 1595, 1507, 1487, 1453, 1355, 1330, 1240, 1183, 1158, 1088, 1006, 957, 929, 815, 749, 694, 678, 656, 594, 576, 546 (cm^{-1}); HRMS (ESI) m/z: calcd for $[C_{24}H_{21}NNaO_4S]^+$ 442.1083; found 442.1085.

Compound S40: Mp: 58-60 °C; ^{1}H NMR (400 MHz, $CDCl_3$) δ 7.59 (d, J = 8.0 Hz, 2H), 7.33-7.27 (m, 6H), 7.26-7.19 (m, 5H), 7.11 (dd, J_1 = 5.6, J_2 = 2.0 Hz, 1H), 7.04-6.95 (m, 5H), 5.76 (d, J = 1.6 Hz, 1H), 4.79 (d, J = 10.4 Hz, 1H), 4.69 (d, J = 10.8 Hz, 1H), 2.44 (s, 3H) ppm; ^{13}C NMR (100 MHz, $CDCl_3$) δ 158.3, 150.2, 148.5, 144.7, 143.7, 142.6, 142.1, 137.8, 134.9, 130.5, 129.7, 129.4, 128.0, 127.9, 127.3, 125.5, 125.3, 120.5, 119.9, 115.5, 91.2, 82.7, 66.2, 21.8 ppm; IR (thin film) 2961, 2927, 2854, 1597, 1504, 1451, 1384, 1341, 1286, 1240, 1156, 1091, 975, 817, 757, 695, 655, 577, 548 (cm^{-1}); HRMS (ESI) m/z: calcd for $[C_{30}H_{25}NNaO_4S]^+$ 518.1397; found 518.1399.

Compound S41: Mp: 77-79 °C; ^{1}H NMR (400 MHz, $CDCl_3$) δ 7.58 (d, J = 8.4 Hz, 2H), 7.28-7.25 (m, 1H), 7.24-7.20 (m, 3H), 7.10 (dd, J_1 = 5.6, J_2 = 1.6 Hz, 1H), 7.02-6.97 (m, 5H), 6.91 (d, J = 8.8 Hz, 2H), 6.29 (s, 1H), 5.76 (d, J = 1.2 Hz, 1H), 4.76 (d, J = 10.8 Hz, 1H), 4.66 (d, J = 10.4 Hz, 1H), 2.39 (s, 3H) ppm; ^{13}C NMR (100 MHz, $CDCl_3$) δ 157.4, 150.3, 148.5, 144.7, 144.0, 142.6, 136.3, 129.8, 129.7, 127.5, 125.7, 125.5, 125.3, 120.5, 119.8, 115.6, 91.3, 82.7, 66.3, 21.8 ppm; IR (thin film) 3431, 3253, 2972, 2857, 2371, 1615, 1508, 1453, 1395, 1336, 1156, 1094, 999, 857, 764, 667, 542, 473 (cm^{-1}); HRMS (ESI) m/z: calcd for $[C_{24}H_{21}NNaO_4S]^+$ 442.1083; found 442.1080.

Reaction of 1a with compound sub-2:

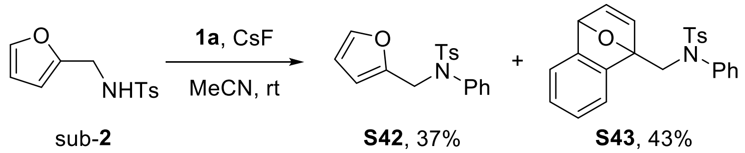

A mixture of aryne precursor **1a** (89.5 mg, 0.3 mmol, 1.5 equiv), compound sub-**2** (50.3 mg, 0.2 mmol, 1.0 equiv), and CsF (91.1 mg, 0.6 mmol, 3.0 equiv) in anhydrous MeCN (2.0 mL) under inert atmosphere was stirred at room temperature overnight. The resulting mixture was filtered through a short pad of silica gel (EtOAc eluent, 20 mL). All the volatiles were removed on a rotary evaporator. Flash column chromatography (pet ether: EtOAc = 8: 1) afforded 24.2 mg (37%) of compound **S42** as a white solid and 34.7 mg (43%) of compound **S43** as a white solid.

Compound S42: Mp: 99-101 °C; ^{1}H NMR (400 MHz, $CDCl_3$) δ 7.51 (d, J = 8.0 Hz, 2H), 7.27-7.22 (m, 6H), 7.04-6.99 (m, 2H), 6.24-6.19 (m, 1H), 6.09 (d, J = 2.8 Hz, 1H), 4.77 (s, 2H), 2.43 (s, 3H) ppm; ^{13}C

NMR (100 MHz, $CDCl_3$) δ 149.8, 143.6, 142.6, 139.3, 136.2, 129.6, 129.3, 129.1, 128.2, 127.9, 110.5, 109.7, 48.2, 21.7 ppm; IR (thin film) 3116, 3059, 2923, 1595, 1492, 1453, 1345, 1160, 1092, 814, 720, 699, 658, 569, 543 (cm^{-1}); HRMS (ESI) m/z: calcd for $[C_{18}H_{18}NO_2S]^+$ 312.1053; found 312.1064.
Compound S43: Mp: 166-168 °C; ^{1}H NMR (400 MHz, $CDCl_3$) δ 7.53 (d, J = 8.0 Hz, 2H), 7.33-7.23 (m, 6H), 7.14 (d, J = 6.4 Hz, 1H), 7.06-7.01 (m, 2H), 6.99-6.91 (m, 3H), 6.85 (d, J = 5.6 Hz, 1H), 5.50 (d, J =0.8 Hz, 1H), 4.74 (d, J = 15.2 Hz, 1H), 4.31 (d, J = 14.8 Hz, 1H), 2.44 (s, 3H) ppm; ^{13}C NMR (100 MHz, $CDCl_3$) δ 150.6, 149.0, 144.3, 143.9, 143.2, 140.3, 135.6, 129.7, 129.5, 129.0, 128.3, 128.0, 125.2, 125.1, 120.1, 119.9, 92.6, 82.2, 50.2, 21.8 ppm; IR (thin film) 3064, 2923, 1595, 1492, 1452, 1349, 1164, 895, 814, 723, 694, 657, 570, 545 (cm^{-1}); HRMS (ESI) m/z: calcd for $[C_{24}H_{21}NNaO_3S]^+$ 426.1134; found 426.1135.

Reaction of 1a with compound sub-3: A mixture of aryne precursor **1a** (89.5 mg, 0.3 mmol, 1.5 equiv), compound sub-**3** (37.4 mg, 0.2 mmol, 1.0 equiv), and CsF (91.1 mg, 0.6 mmol, 3.0 equiv) in anhydrous MeCN (2.0 mL) under inert atmosphere was stirred at room temperature overnight. The resulting mixture was filtered through a short pad of silica gel (EtOAc eluent, 20 mL). All the volatiles were removed on a rotary evaporator. Flash column chromatography (pet ether: EtOAc = 50: 1) afforded 14.2 mg (27%) of compound **S44** as a colorless oil, 8.1 mg (12%) of compound **S45** as a colorless oil, 19.0 mg (28%) of compound **S46** as a colorless oil and 14.7 mg (28%) of compound **S47** as a colorless oil. The structures of **S45** and **S46** are estimated based on **S53** and **S54**.

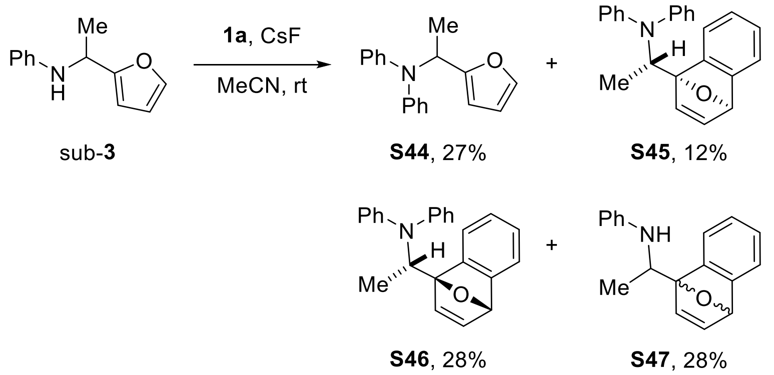

Compound S44: ^{1}H NMR (400 MHz, $CDCl_3$) δ 7.35 (s, 1H), 7.23 (t, J = 7.2 Hz, 4H), 6.97 (t, J = 7.6 Hz, 2H), 6.86-6.82 (m, 4H), 6.26 (dd, J_1 = 3.2, J_2 = 2.0 Hz, 1H), 6.07- 6.04 (m, 1H), 5.39 (q, J = 6.8 Hz, 1H), 1.52 (d, J = 7.2 Hz, 3H) ppm; ^{13}C NMR (100 MHz, $CDCl_3$) δ 156.2, 146.6, 141.6, 129.2, 122.9, 122.1, 110.3, 107.3, 51.4, 17.1 ppm; IR (thin film) 3058, 2981, 2935, 1589, 1494, 1449, 1373, 1283, 1233, 1148, 1012, 746, 700, 598 (cm^{-1}); HRMS (ESI) m/z: calcd for $[C_{18}H_{18}NO]^+$ 264.1383; found 264.1389.
Compound S45: ^{1}H NMR (400 MHz, $CDCl_3$) δ 7.42-7.39 (m, 1H), 7.31-7.26 (m, 4H), 7.22-7.19 (m, 1H), 7.08-7.04 (m, 4H), 7.03-6.97 (m, 4H), 6.93 (dd, J_1 = 5.6, J_2 = 1.6 Hz, 1H), 6.67 (d, J = 5.6 Hz, 1H), 5.65 (d, J = 2.0 Hz, 1H), 5.32 (q, J = 7.2 Hz, 1H), 1.34 (d, J = 7.2 Hz, 3H) ppm; ^{13}C NMR (100 MHz, $CDCl_3$) δ 151.1, 150.3, 147.1, 144.0, 143.8, 129.4, 125.1, 123.5, 122.3, 120.1, 120.0, 96.7, 81.4, 52.4, 15.6 ppm; IR (thin film) 3061, 3018, 2934, 1588, 1495, 1452, 1283, 1241, 1101, 991, 908, 746, 691, 629 (cm^{-1}); HRMS (ESI) m/z: calcd for $[C_{24}H_{22}NO]^+$ 340.1696; found 340.1698.
Compound S46: ^{1}H NMR (400 MHz, $CDCl_3$) δ 7.26-7.21 (m, 4H), 7.19 (d, J = 7.2 Hz, 1H), 7.00 (t, J = 7.2 Hz, 2H), 6.96-6.90 (m, 6H), 6.84 (t, J = 7.2 Hz, 1H), 6.81-6.76 (m, 2H), 5.65 (d, J = 1.6 Hz, 1H), 5.20 (q, J = 6.8 Hz, 1H), 1.55 (d, J = 7.2 Hz, 3H) ppm; ^{13}C NMR (100 MHz, $CDCl_3$) δ 151.2, 149.6, 147.1, 144.2, 143.7, 129.3, 124.9, 124.0, 122.3, 121.1, 120.1, 96.6, 82.3, 52.9, 16.8 ppm; IR (thin film) 3059, 3019, 2932, 1588, 1495, 1453, 1284, 1241, 1101, 1027, 910, 746, 690, 609 (cm^{-1}); HRMS (ESI) m/z: calcd for $[C_{24}H_{22}NO]^+$ 340.1696; found 340.1697.
Compound S47: ^{1}H NMR (400 MHz, $CDCl_3$) δ 7.26-7.18 (m, 7H), 7.13 (d, J = 6.4 Hz, 1H), 7.09 (dd, J_1 = 5.6, J_2 = 2.0 Hz, 1H), 7.03-6.98 (m, 3H), 6.98-6.92 (m, 2H), 6.90 (d, J = 5.6 Hz, 1H), 6.84 (d, J = 5.6 Hz, 1H), 6.76-6.69 (m, 4H), 6.67-6.64 (m, 2H), 5.76 (d, J = 2.0 Hz, 1H), 5.74 (d, J = 2.0 Hz, 1H), 4.57-4.49 (m, 1H), 4.01 (brs, 1H), 1.48 (d, J = 6.4 Hz, 3H), 1.41 (d, J = 6.8 Hz, 3H) ppm; ^{13}C NMR (100 MHz, $CDCl_3$) δ 151.4, 151.0, 148.6, 148.5, 147.4, 147.3, 145.0, 144.2, 144.0, 143.0, 129.6, 129.5, 125.3, 125.2,

125.1, 125.0, 120.5, 120.2, 120.1, 117.7, 117.4, 113.5, 113.2, 96.6, 96.3, 82.4, 82.1, 47.7, 47.1, 16.6, 16.5 ppm; IR (thin film) 3406, 3048, 3017, 2976, 2930, 1601, 1507, 1453, 1317, 1154, 989, 903, 747, 690, 657, 625 (cm^{-1}); HRMS (ESI) m/z: calcd for $[C_{18}H_{18}NO]^+$ 264.1383; found 264.1392.

Reaction of 1a with compound sub-4:

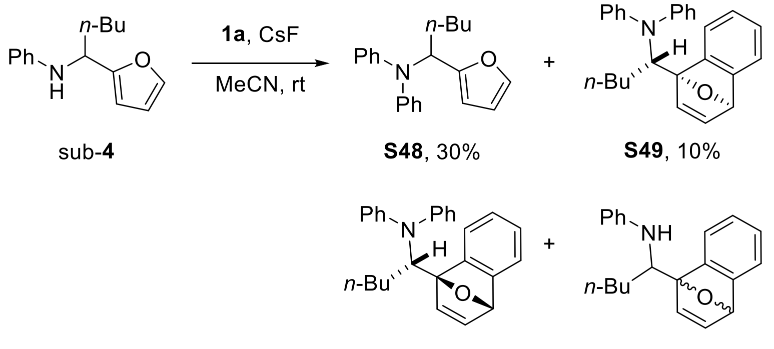

A mixture of aryne precursor **1a** (89.5 mg, 0.3 mmol, 1.5 equiv), compound sub-**4** (45.9 mg, 0.2 mmol, 1.0 equiv), and CsF (91.1 mg, 0.6 mmol, 3.0 equiv) in anhydrous MeCN (2.0 mL) under inert atmosphere was stirred at room temperature overnight. The resulting mixture was filtered through a short pad of silica gel (EtOAc eluent, 20 mL). All the volatiles were removed on a rotary evaporator. Flash column chromatography (pet ether: EtOAc = 50: 1) afforded 18.3 mg (30%) of compound **S48** as a colorless oil, 7.6 mg (10%) of compound **S49** as a colorless oil, 22.9 mg (30%) of compound **S50** as a colorless oil and 16.5 mg (27%) of compound **S51** as a colorless oil. The structures of **S49** and **S50** are estimated based on **S53** and **S54**.

Compound S48: ^{1}H NMR (400 MHz, $CDCl_3$) δ 7.34-7.30 (m, 1H), 7.24-7.19 (m, 4H), 6.96 (t, J = 7.2 Hz, 2H), 6.84 (d, J = 7.6 Hz, 4H), 6.25 (dd, J_1 = 3.2, J_2 = 2.0 Hz, 1H), 6.04 (d, J = 3.2 Hz, 1H), 5.19 (t, J = 7.6 Hz, 1H), 1.97-1.88 (m, 2H), 1.52-1.45 (m, 1H), 1.41-1.30 (m, 3H), 0.89 (t, J = 7.2 Hz, 3H) ppm; ^{13}C NMR (100 MHz, $CDCl_3$) δ 155.5, 147.0, 141.5, 129.2, 122.9, 122.0, 110.2, 107.7, 56.5, 31.0, 29.1, 22.8, 14.2 ppm; IR (thin film) 3059, 3035, 2956, 2931, 2860, 1588, 1495, 1465, 1282, 1147, 1012, 744, 699, 597 (cm^{-1}); HRMS (ESI) m/z: calcd for $[C_{21}H_{24}NO]^+$ 306.1852; found 306.1852.

Compound S49: ^{1}H NMR (400 MHz, $CDCl_3$) δ 7.29-7.23 (m, 5H), 7.22-7.11 (m, 5H), 7.01-2-6.94 (m, 4H), 6.91 (dd, J_1 = 5.6, J_2 = 2.0 Hz, 1H), 6.70 (d, J = 5.6 Hz, 1H), 5.68 (d, J = 1.6 Hz, 1H), 5.19 (dd, J_1 = 10.8, J_2 = 2.8 Hz, 1H), 1.85-1.75 (m, 1H), 1.68 -1.61 (m, 1H), 1.52-1.37 (m, 2H), 1.35-1.27 (m, 1H), 1.25-1.15 (m, 1H), 0.79 (t, J = 7.2 Hz, 3H) ppm; ^{13}C NMR (100 MHz, $CDCl_3$) δ 151.0, 150.4, 144.1, 143.8, 129.3, 125.1, 125.0, 123.5, 122.0, 120.1, 119.7, 97.2, 81.5, 57.7, 29.4, 28.4, 22.7, 14.1 ppm; IR (thin film) 3018, 2954, 2927, 2867, 1586, 1496, 1450, 1291, 933, 747, 701, 690, 630 (cm^{-1}); HRMS (ESI) m/z: calcd for $[C_{27}H_{28}NO]^+$ 382.2165; found 382.2173.

Compound S50: ^{1}H NMR (400 MHz, $CDCl_3$) δ 7.25-7.20 (m, 4H), 7.17 (d, J = 6.8 Hz, 1H), 7.00-6.94 (m, 6H), 6.93-6.88 (m, 2H), 6.82-6.76 (m, 2H), 6.64 (d, J = 7.2 Hz, 1H), 5.65 (d, J = 2.0 Hz, 1H), 5.03 (dd, J_1 = 11.2, J_2 = 2.0 Hz, 1H), 2.00-1.90 (m, 1H), 1.81-1.71 (m, 2H), 1.62-1.54 (m, 1H), 1.46-1.33 (m, 2H), 0.91 (t, J = 7.2 Hz, 3H) ppm; ^{13}C NMR (100 MHz, $CDCl_3$) δ 151.2, 149.9, 147.5, 144.4, 143.4, 129.3, 124.8, 123.8, 122.2, 121.0, 120.0, 96.5, 82.1, 58.2, 30.7, 29.6, 23.0, 14.3 ppm; IR (thin film) 3061, 3018, 2955, 2929, 2858, 1588, 1496, 1453, 1282, 911, 746, 700, 690, 656 (cm^{-1}); HRMS (ESI) m/z: calcd for $[C_{27}H_{28}NO]^+$ 382.2165; found 382.2173.

Compound S51: ^{1}H NMR (400 MHz, $CDCl_3$) δ 7.24-7.16 (m, 7H), 7.06-7.03 (m, 2H), 7.02-6.96 (m, 3H), 6.95-6.91 (m, 1H), 6.89-6.84 (m, 2H), 6.82 (d, J = 5.6 Hz, 1H), 6.74 (d, J = 7.6 Hz, 2H), 6.72-6.63 (m, 4H), 5.73 (d, J = 1.6 Hz, 1H), 5.71 (d, J = 1.6 Hz, 1H), 4.41 (dd, J_1 = 9.6, J_2 = 2.8 Hz, 1H), 4.34 (dd, J_1 = 8.4, J_2 = 4.0 Hz, 1H), 3.94 (brs, 2H), 1.99-1.88 (m, 2H), 1.80-1.72 (m, 1H), 1.69-1.63 (m, 1H), 1.57-1.27 (m, 8H), 0.91 (t, J = 7.2 Hz, 3H), 0.87 (t, J = 7.2 Hz, 3H) ppm; ^{13}C NMR (100 MHz, $CDCl_3$) δ 151.3, 151.0, 149.1, 148.9, 148.8, 148.7, 144.6, 144.2, 144.1, 143.4, 129.6, 129.5, 125.3, 125.2, 125.1, 125.0, 120.6, 120.2, 120.1, 117.4, 117.1, 113.1, 112.9, 97.2, 96.9, 82.2, 81.9, 52.4, 52.2, 33.0, 32.6, 29.0, 28.9,

23.2, 23.0, 14.3, 14.2 ppm; IR (thin film) 3410, 3018, 2955, 2930, 2858, 1600, 1507, 1454, 1318, 1250, 1146, 862, 746, 690, 657 (cm^{-1}); HRMS (ESI) m/z: calcd for $[C_{21}H_{24}NO]^+$ 306.1852; found 306.1854.

Reaction of 1a with compound sub-5:

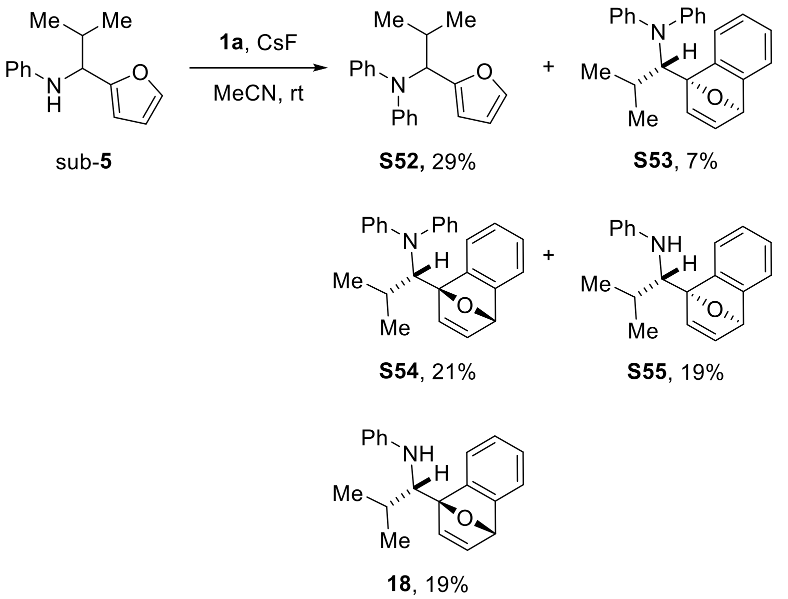

A mixture of aryne precursor **1a** (89.5 mg, 0.3 mmol, 1.5 equiv), compound sub-**5** (43.1 mg, 0.2 mmol, 1.0 equiv), and CsF (91.1 mg, 0.6 mmol, 3.0 equiv) in anhydrous MeCN (2.0 mL) under inert atmosphere was stirred at room temperature overnight. The resulting mixture was filtered through a short pad of silica gel (EtOAc eluent, 20 mL). All the volatiles were removed on a rotary evaporator. Flash column chromatography (pet ether: EtOAc = 50: 1) afforded afforded 16.9 mg (29%) of compound **S52** as a colorless oil, 5.1 mg (7%) of compound **S53** as a colorless oil, 15.4 mg (21%) of compound **S54** as a colorless oil, 11.1 mg (19%) of compound **S55** as a colorless oil and 11.1 mg (19%) of compound **18** as a colorless oil.

Compound S52: ^{1}H NMR (400 MHz, $CDCl_3$) δ 7.29-7.27 (m, 1H), 7.22 (dd, J_1 = 8.4, J_2 = 7.6 Hz, 4H), 6.95 (t, J = 7.2 Hz, 2H), 6.91 (d, J = 7.6 Hz, 4H), 6.24 (dd, J_1 = 3.2, J_2 = 2.0 Hz, 1H), 6.03 (d, J = 3.2 Hz, 1H), 4.81 (d, J = 10.4 Hz, 1H), 2.49-2.39 (m, 1H), 1.21 (d, J = 6.8Hz, 3H), 0.94 (d, J = 6.4 Hz, 3H) ppm; ^{13}C NMR (100 MHz, $CDCl_3$) δ 154.5, 147.9, 141.1, 129.2, 123.2, 121.9, 110.1, 108.4, 64.3, 30.6, 21.0, 20.9 ppm; IR (thin film) 3431, 3062, 2963, 2874, 2375, 1591, 1498, 1391, 1262, 1150, 1108, 1002, 859, 812, 742, 699, 541, 472 (cm^{-1}); HRMS (ESI) m/z: calcd for $[C_{20}H_{21}NNaO]^+$ 314.1515; found 314.1523.

Compound S53: ^{1}H NMR (400 MHz, $CDCl_3$) δ 7.30 (d, J = 8.0 Hz, 4H), 7.24 (t, J = 8.4 Hz, 4H), 7.20-7.17 (m, 1H), 7.15-7.13 (m, 1H), 7.00 (d, J = 5.6 Hz, 1H), 6.95-6.90 (m, 4H), 6.88 (dd, J_1 = 5.6, J_2 = 2.0 Hz, 1H), 5.78 (d, J = 2.0 Hz, 1H), 4.82 (d, J = 10.8 Hz, 1H), 2.52-2.44 (m, 1H), 1.03 (d, J = 6.8 Hz, 3H), 0.85 (d, J = 6.4 Hz, 3H) ppm; ^{13}C NMR (100 MHz, $CDCl_3$) δ 152.0, 149.9, 146.0, 142.8, 129.2, 125.0, 124.8, 123.1, 121.5, 120.1, 120.0, 97.3, 81.7, 65.7, 28.1, 22.2, 21.8 ppm; IR (thin film) 3385, 3064, 2959, 2924, 1589, 1493, 1472, 1386, 1259, 1057, 937, 752, 690, 634 (cm^{-1}); HRMS (ESI) m/z: calcd for $[C_{26}H_{26}NO]^+$ 368.2009; found 368.2011.

Compound S54: ^{1}H NMR (400 MHz, $CDCl_3$) δ 7.23 (d, J = 7.6 Hz, 4H), 7.16 (d, J = 7.2 Hz, 1H), 7.12 (d, J = 5.6 Hz, 1H), 7.08-6.94 (m, 7H), 6.87 (t, J = 7.2 Hz, 1H), 6.71 (t, J = 7.6 Hz, 1H), 6.48 (d, J = 7.2 Hz, 1H), 5.65 (d, J = 1.6 Hz, 1H), 4.62 (d, J = 10.8 Hz, 1H), 2.54-2.44 (m, 1H), 1.20 (d, J = 6.4 Hz, 3H), 0.96 (d, J = 6.8 Hz, 3H) ppm; ^{13}C NMR (100 MHz, $CDCl_3$) δ 150.5, 149.8, 146.5, 143.2, 129.4, 124.7, 121.9, 119.9, 96.7, 82.1, 65.9, 30.3, 22.3, 21.3 ppm; IR (thin film) 3061, 2959, 2870, 1589, 1496, 1454, 1350, 1281, 1223, 1001, 890, 748, 692, 634 (cm^{-1}); HRMS (ESI) m/z: calcd for $[C_{26}H_{26}NO]^+$ 368.2009; found 368.2019.

Compound S55: ^{1}H NMR (400 MHz, $CDCl_3$) δ 7.20-7.11 (m, 3H), 7.02 (dd, J_1 = 5.6, J_2 = 2.0 Hz, 1H), 6.93-6.82 (m, 3H), 6.78 (t, J = 8.0 Hz,1H), 6.69-6.60 (m, 3H), 5.71 (d, J = 1.6 Hz, 1H), 4.33 (d, J = 4.8 Hz, 1H), 4.07 (s, 1H), 2.38-2.26 (m, 1H), 1.14 (d, J = 6.8 Hz, 3H), 1.09 (d, J = 6.8 Hz, 3H) ppm; ^{13}C

NMR (100 MHz, $CDCl_3$) δ 150.5, 149.8, 149.3, 144.3, 143.6, 129.5, 125.1, 124.9, 120.4, 120.0, 116.8, 112.9, 97.5, 82.2, 55.9, 32.0, 22.4, 18.3 ppm; IR (thin film) 3422, 2970, 2852, 2347, 1601, 1509, 1385, 1094, 984, 863, 784, 744, 693, 538, 476 (cm^{-1}); HRMS (ESI) m/z: calcd for $[C_{20}H_{21}NNaO]^+$ 314.1515; found 314.1520.

Compound 18: ^{1}H NMR (400 MHz, $CDCl_3$) δ 7.22-7.18 (m, 4H), 6.99-6.94 (m, 2H), 6.92 (dd, J_1 = 5.6, J_2 = 2.0 Hz, 1H), 6.79-6.75 (m, 3H), 6.70 (t, J = 7.2 Hz, 1H), 5.70 (d, J = 2.0 Hz, 1H), 4.37 (d, J = 7.6 Hz, 1H), 4.06 (d, J = 9.2 Hz, 1H), 2.38-2.30 (m, 1H), 1.09 (d, J = 6.8 Hz, 3H), 1.02 (d, J = 6.8 Hz, 3H) ppm; ^{13}C NMR (100 MHz, $CDCl_3$) δ 151.1, 149.8, 149.3, 144.9, 143.5, 129.7, 125.2, 125.1, 120.3, 120.1, 117.4, 113.2, 97.2, 82.0, 56.7, 30.7, 22.7, 17.4 ppm; IR (thin film) 3855, 3414, 3052, 2958, 2927, 2871, 2346, 1600, 1509, 1453, 1384, 1305, 1123, 999, 948, 862, 746, 690, 629, 540, 474 (cm^{-1}); HRMS (ESI) m/z: calcd for $[C_{20}H_{21}NNaO]^+$ 314.1515; found 314.1520.

Reaction of 1a with Fuberidazole:

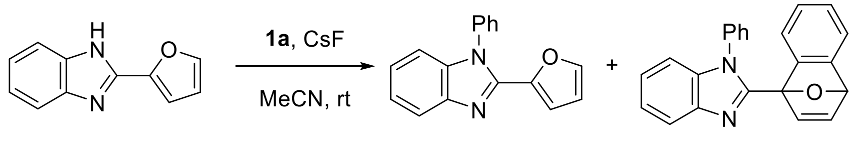

Fuberidazole **S56**, 48% **S57**, 33%

A mixture of aryne precursor **1a** (89.5 mg, 0.3 mmol, 1.5 equiv), commercially available Fuberidazole (36.8 mg, 0.2 mmol, 1.0 equiv), and CsF (91.1 mg, 0.6 mmol, 3.0 equiv) in anhydrous MeCN (2.0 mL) under inert atmosphere was stirred at room temperature overnight. The resulting mixture was filtered through a short pad of silica gel (EtOAc eluent, 20 mL). All the volatiles were removed on a rotary evaporator. Flash column chromatography (pet ether: EtOAc = 10: 1) afforded 25.0 mg (48%) of compound **S56** as a white solid and 22.2 mg (33%) of compound **S57** as a white solid.

Compound S56: Mp: 105-107 °C; ^{1}H NMR (400 MHz, $CDCl_3$) δ 7.86 (d, J = 8.0 Hz, 1H), 7.59-7.55 (m, 3H), 7.46 (d, J = 0.8 Hz, 1H), 7.41-7.36 (m, 2H), 7.30 (t, J = 7.6 Hz, 1H), 7.21 (t, J = 7.6 Hz, 1H), 7.07 (d, J = 8.0 Hz, 1H), 6.33 (dd, J_1 = 3.2, J_2 = 1.6 Hz, 1H), 6.15 (d, J = 3.6 Hz, 1H) ppm; ^{13}C NMR (100 MHz, $CDCl_3$) δ 144.7, 144.3, 144.2, 143.0, 137.3, 136.5, 130.1, 129.6, 128.1, 123.7, 123.2, 120.0, 112.3, 111.6, 110.3 ppm; IR (thin film) 3133, 3090, 3054, 2925, 1592, 1499, 1454, 1423, 1415, 1374, 1324, 1227, 1193, 1118, 1075, 1006, 884, 742, 699, 631, 588, 491 (cm^{-1}); HRMS (ESI) m/z: calcd for $[C_{17}H_{13}N_2O]^+$ 261.1022; found 261.1029.

Compound S57: Mp: 126-128 °C; ^{1}H NMR (400 MHz, $CDCl_3$) δ 7.95 (d, J = 8.0 Hz, 1H), 7.39-7.24 (m, 9H), 7.17-7.13 (m, 2H), 6.95 (dd, J_1 = 5.2, J_2 = 1.6 Hz, 1H), 6.92-6.86 (m, 2H), 5.66 (d, J = 2.0 Hz, 1H) ppm; ^{13}C NMR (100 MHz, $CDCl_3$) δ 149.3, 148.9, 148.4, 144.4, 143.1, 142.4, 137.9, 136.1, 128.9, 128.8, 128.4, 125.2, 125.1, 124.0, 123.1, 121.6, 120.4, 120.0, 111.0, 89.5, 83.1 ppm; IR (thin film) 3053, 2924, 2874, 1596, 1498, 1453, 1403, 1268, 1195, 1103, 1019, 947, 861, 814, 795, 758, 691, 646, 531 (cm^{-1}); HRMS (ESI) m/z: calcd for $[C_{23}H_{17}N_2O]^+$ 337.1335; found 337.1345.

Reaction of 1a with compound sub-6:

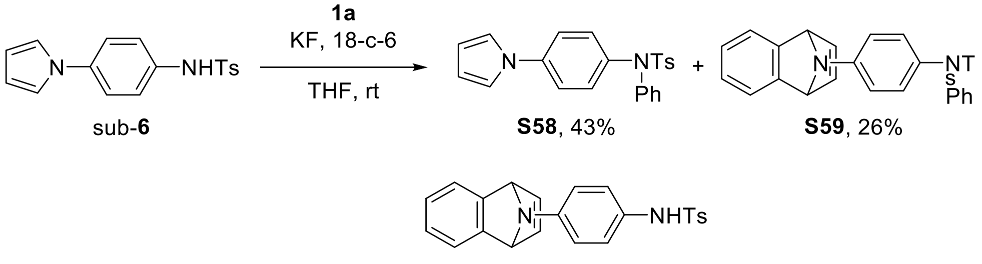

sub-**6** **S58**, 43% **S59**, 26% **S60**, 20%

A mixture of aryne precursor **1a** (89.5 mg, 0.3 mmol, 1.5 equiv), compound sub-**6** (62.5 mg, 0.2 mmol, 1.0 equiv), 18-crown-6 (105.7 mg, 0.4 mmol, 2.0 equiv), and KF (46.5 mg, 0.8 mmol, 4.0 equiv) in anhydrous THF (2.0 mL) under inert atmosphere was stirred at room temperature overnight. The resulting

mixture was filtered through a short pad of silica gel (EtOAc eluent, 20 mL). All the volatiles were removed on a rotary evaporator. Flash column chromatography (pet ether: EtOAc = 5: 1 to 1: 1) afforded 33.4 mg (43%) of compound **S58** as a white solid, 24.2 mg (26%) of compound **S59** as a colorless oil and 15.5 mg (20%) of compound **S60** as a colorless oil.

Compound S58: Mp: 104-106 °C; ^{1}H NMR (400 MHz, $CDCl_3$) δ 7.60 (d, J = 8.0 Hz, 2H), 7.35-7.31 (m, 6H), 7.30-7.26 (m, 5H), 7.04 (t, J = 2.0 Hz, 2H), 6.34 (t, J = 2.0 Hz, 2H), 2.45 (s, 3H) ppm; ^{13}C NMR (100 MHz, $CDCl_3$) δ 144.0, 141.7, 139.9, 139.0, 137.6, 129.8, 129.7, 129.6, 128.4, 128.0, 127.8, 121.1, 119.5, 111.1, 21.8 ppm; IR (thin film) 3061, 2923, 1596, 1515, 1487, 1354, 1332, 1163, 1091, 814, 723, 695, 663, 581, 549 (cm^{-1}); HRMS (ESI) m/z: calcd for $[C_{23}H_{21}N_2O_2S]^+$ 389.1318; found 389.1330.

Compound S59: ^{1}H NMR (400 MHz, $CDCl_3$) δ 7.48 (d, J = 8.4 Hz, 2H), 7.28 (d, J = 8.4 Hz, 2H), 7.25-7.24 (m, 2H), 7.23-7.18 (m, 5H), 7.05 (d, J = 8.8 Hz, 2H), 6.95- 6.91 (m, 4H), 6.72 (d, J = 8.8 Hz, 2H), 5.38 (t, J = 1.2 Hz, 2H), 2.42 (s, 3H) ppm; ^{13}C NMR (100 MHz, $CDCl_3$) δ 148.5, 146.5, 143.5, 142.2, 142.1, 137.7, 134.1, 129.6, 129.5, 129.3, 128.1, 128.0, 127.2, 125.2, 121.7, 118.5, 69.4, 21.8 ppm; IR (thin film) 3378, 3060, 2923, 1595, 1506, 1451, 1350, 1163, 1091, 813, 753, 695, 660, 579, 548 (cm^{-1}); HRMS (ESI) m/z: calcd for $[C_{29}H_{25}N_2O_2S]^+$ 465.1631; found 465.1634.

Compound S60: ^{1}H NMR (400 MHz, $CDCl_3$) δ 7.45 (d, J = 8.0 Hz, 2H), 7.24 (dd, J_1 = 5.2, J_2 = 2.8 Hz, 2H), 7.12 (d, J = 8.0 Hz, 2H), 6.95-6.90 (m, 4H), 6.82 (d, J = 8.8 Hz, 2H), 6.67 (d, J = 8.8 Hz, 2H), 6.27 (s, 1H), 5.35 (t, J = 1.6 Hz, 2H), 2.36 (s, 3H) ppm; ^{13}C NMR (100 MHz, $CDCl_3$) δ 148.4, 145.6, 143.7, 142.1, 136.1, 129.6, 129.3, 127.5, 125.2, 124.9, 121.8, 118.7, 69.5, 21.7 ppm; IR (thin film) 3259, 3062, 2923, 1597, 1508, 1452, 1398, 1331, 1160, 1091, 924, 813, 735, 664, 572, 545 (cm^{-1}); HRMS (ESI) m/z: calcd for $[C_{23}H_{21}N_2O_2S]^+$ 389.1318; found 389.1325.

Reaction of 1a with compound sub-7:

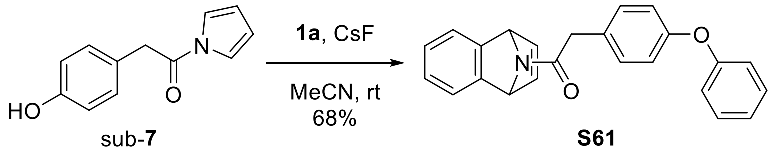

A mixture of aryne precursor **1a** (89.5 mg, 0.3 mmol, 1.5 equiv), compound sub-**7** (40.2 mg, 0.2 mmol, 1.0 equiv), and CsF (91.1 mg, 0.6 mmol, 3.0 equiv) in anhydrous MeCN (2.0 mL) under inert atmosphere was stirred at room temperature overnight. The resulting mixture was filtered through a short pad of silica gel (EtOAc eluent, 20 mL). All the volatiles were removed on a rotary evaporator. Flash column chromatography (pet ether: EtOAc = 8: 1) afforded 48.1 mg (68%) of compound **S61** as a white solid. Mp: 146-148 °C; ^{1}H NMR (400 MHz, $CDCl_3$) δ 7.34-7.28 (m, 3H), 7.13-7.07 (m, 4H), 7.04 (dd, J_1 = 5.6, J_2 = 2.4 Hz, 1H), 7.00-6.93 (m, 4H), 6.92-6.87 (m, 3H), 5.93 (s, 1H), 5.57 (s, 1H), 3.57 (s, 2H) ppm; ^{13}C NMR (100 MHz, $CDCl_3$) δ 166.5, 157.5, 156.3, 148.3, 148.1, 144.3, 142.5, 130.5, 129.9, 129.0, 125.5, 125.2, 123.4, 121.6, 120.4, 119.3, 118.9, 66.0, 63.7, 40.6 ppm; IR (thin film) 3426, 3184, 2404, 1629. 1586, 1500, 1402, 1279, 1237, 1152, 1108, 993, 864, 790, 753, 695, 638, 543, 473 (cm^{-1}); HRMS (ESI) m/z: calcd for $[C_{24}H_{19}NNaO_2]^+$ 376.1308; found 376.1311.

Reaction of 1a with compound sub-8:

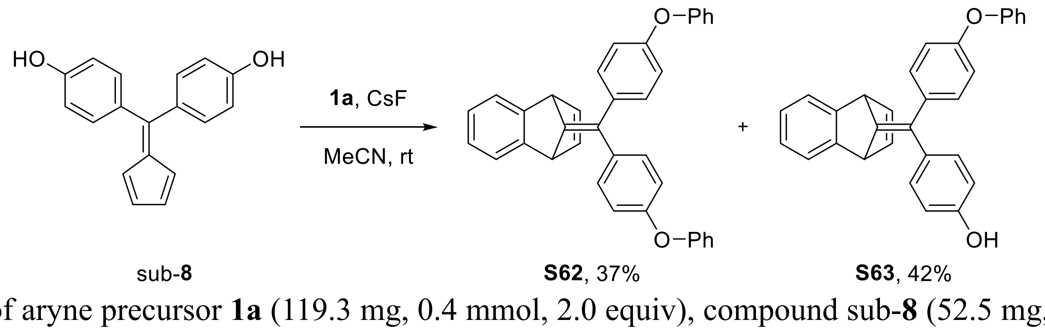

A mixture of aryne precursor **1a** (119.3 mg, 0.4 mmol, 2.0 equiv), compound sub-**8** (52.5 mg, 0.2 mmol, 1.0 equiv), and CsF (121.5 mg, 0.8 mmol, 4.0 equiv) in anhydrous MeCN (2.0 mL) under inert atmosphere

was stirred at room temperature overnight. The resulting mixture was filtered through a short pad of silica gel (EtOAc eluent, 20 mL). All the volatiles were removed on a rotary evaporator. Flash column chromatography (pet ether: EtOAc = 10: 1 to 4: 1) afforded 36.3 mg (37%) of compound **S62** as a colorless oil and 34.8 mg (42%) of compound **S63** as a yellow oil.
Compound S62: ^{1}H NMR (400 MHz, $CDCl_3$) δ 7.40-7.33 (m, 4H), 7.28 (dd, J_1 = 5.2, J_2 = 3.2 Hz, 2H), 7.12 (t, J = 7.2 Hz, 2H), 7.08-6.99 (m, 12H), 6.94 (d, J = 8.8 Hz, 4H), 4.46 (t, J = 2.0 Hz, 2H) ppm; ^{13}C NMR (100 MHz, $CDCl_3$) δ 164.2, 157.2, 156.3, 149.8, 143.1, 135.4, 131.3, 130.0, 125.2, 123.6, 121.5, 119.3, 118.3, 114.0, 52.5 ppm; IR (thin film) 3425, 2919, 2851, 2375, 1630, 1503, 1488, 1400, 1232, 1098, 986, 864, 751, 690, 543, 475 (cm^{-1}); HRMS (ESI) m/z: calcd for $[C_{36}H_{27}O_2]^+$ 491.2006; found 491.2011.
Compound S63: ^{1}H NMR (400 MHz, DMSO-*d6*) δ 9.47 (s, 1H), 7.41 (t, J = 8.0 Hz, 2H), 7.33-7.28 (m, 2H), 7.16 (t, J = 7.2 Hz, 1H), 7.08-7.01 (m, 4H), 6.99-6.93 (m, 6H), 6.79 (d, J = 8.8 Hz, 2H), 6.73 (d, J = 8.4 Hz, 2H), 4.40-4.37 (m, 2H) ppm; ^{13}C NMR (100 MHz, DMSO-*d6*) δ 163.2, 156.4, 156.3, 155.5, 149.4, 149.3, 142.9, 142.8, 135.0, 130.8, 130.4, 130.1, 124.7, 123.6, 121.2, 119.0, 117.9, 115.0, 113.2, 51.8, 51.7 ppm; IR (thin film) 3426, 2919, 2849, 2360, 1633, 1400, 1384, 1098, 986, 860, 691, 657, 543, 473 (cm^{-1}); HRMS (ESI) m/z: calcd for $[C_{30}H_{23}O_2]^+$ 415.1693; found 415.1705.

Reaction of 1a with compound sub-9:

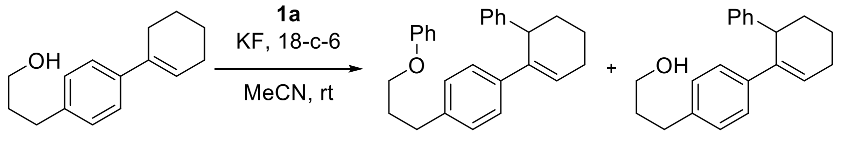

sub-**9** **S64**, 36% **S65**, 45%

A mixture of aryne precursor **1a** (89.5 mg, 0.3 mmol, 1.5 equiv), compound sub-**9** (43.3 mg, 0.2 mmol, 1.0 equiv), 18-crown-6 (105.7 mg, 0.4 mmol, 2.0 equiv), and KF (46.5 mg, 0.8 mmol, 4.0 equiv) in anhydrous MeCN (2.0 mL) under inert atmosphere was stirred at room temperature overnight. The resulting mixture was filtered through a short pad of silica gel (EtOAc eluent, 20 mL). All the volatiles were removed on a rotary evaporator. Flash column chromatography (pet ether: EtOAc = 20: 1 to 4: 1) afforded 26.5 mg (36%) of compound **S64** as a white solid and 26.3 mg (45%) of compound **S65** as a colorless oil.
Compound S64: Mp: 54-56 °C; ^{1}H NMR (400 MHz, $CDCl_3$) δ 7.33-7.29 (m, 2H), 7.29-7.26 (m, 2H), 7.26-7.23 (m, 4H), 7.20-7.13 (m, 1H), 7.06 (d, J = 8.4 Hz, 2H), 6.97 (t, J = 7.6 Hz, 1H), 6.93-6.87 (m, 2H), 6.43 (t, J = 4.0 Hz, 1H), 4.04 (brs, 1H), 3.95 (t, J = 6.4 Hz, 2H), 2.78-2.67 (t, J = 7.2 Hz 2H), 2.42-2.29 (m, 2H), 2.15-2.03 (m, 3H), 1.93-1.86 (m, 1H), 1.64-1.57 (m, 2H) ppm; ^{13}C NMR (100 MHz, $CDCl_3$) δ 159.2, 145.5, 139.8, 139.7, 137.7, 129.6, 128.8, 128.4, 128.3, 127.4, 126.0, 125.9, 120.7, 114.8, 67.0, 42.7, 32.8, 31.9, 30.9, 26.3, 17.5 ppm; IR (thin film) 3435, 3024, 2924, 2866, 2356, 1598, 1493, 1467, 1446, 1390, 1293, 1244, 1079, 995, 851, 798, 751, 694, 541, 475 (cm^{-1}); HRMS (ESI) m/z: calcd for $[C_{27}H_{28}NaO]^+$ 391.2032; found 391.2041.
Compound S65: ^{1}H NMR (400 MHz, $CDCl_3$) δ 7.26-7.18 (m, 6H), 7.15-7.10 (m, 1H), 7.00 (d, J = 8.4 Hz, 2H), 6.38 (t, J = 3.6 Hz, 1H), 4.00 (s, 1H), 3.62 (t, J = 6.4 Hz, 2H), 2.60 (t, J = 7.2 Hz, 2H), 2.39-2.24 (m, 2H), 2.12-2.02 (m, 1H), 1.88-1.77 (m, 3H), 1.57-1.51 (m, 2H), 1.24 (brs, 1H) ppm; ^{13}C NMR (100 MHz, $CDCl_3$) δ 145.5, 140.0, 139.7, 137.6, 128.8, 128.4, 128.3, 127.4, 126.0, 125.9, 62.5, 42.7, 34.2, 32.8, 31.8, 26.3, 17.5 ppm; IR (thin film) 3425, 3029, 2931, 2360, 1637, 1400, 1384, 1095, 986, 859, 803, 701, 543, 474 (cm^{-1}); HRMS (ESI) m/z: calcd for $[C_{21}H_{24}NaO]^+$ 315.1719; found 315.1722.

Reaction of 1a with *N*-deacetyl *N*-tosyl Colchicine sub-10:

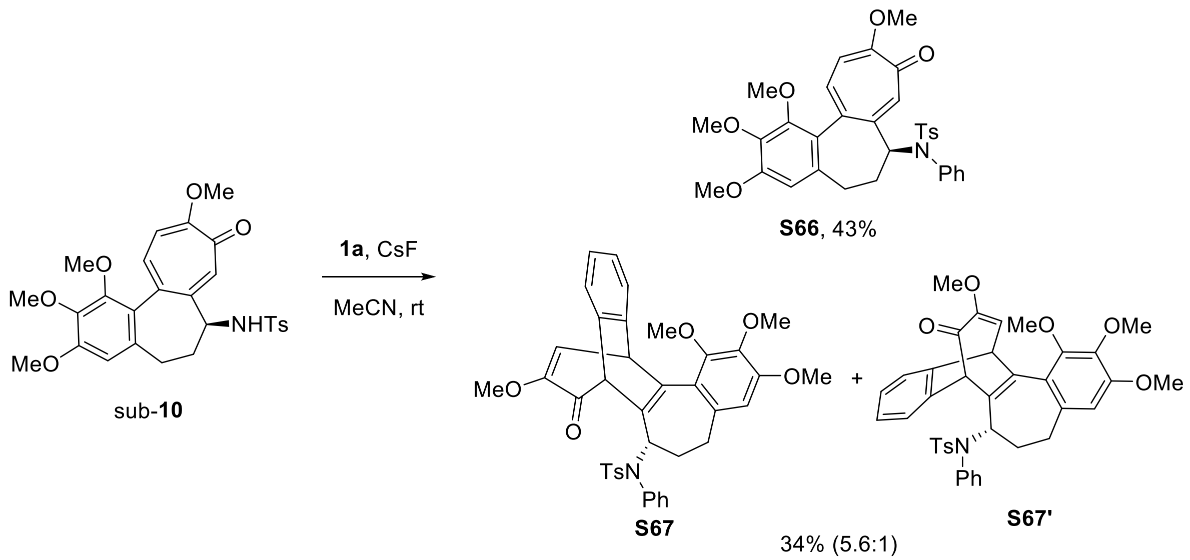

A mixture of aryne precursor **1a** (89.5 mg, 0.3 mmol, 1.5 equiv), *N*-deacetyl *N*-tosyl Colchicine sub-**10** (102.3 mg, 0.2 mmol, 1.0 equiv), and CsF (91.1 mg, 0.6 mmol, 3.0 equiv) in anhydrous MeCN (2.0 mL) under inert atmosphere was stirred at room temperature overnight. The resulting mixture was filtered through a short pad of silica gel (EtOAc eluent, 20 mL). All the volatiles were removed on a rotary evaporator. Flash column chromatography (pet ether: EtOAc = 2: 1 to 1: 4) afforded 50.5 mg (43%) of compound **S66** as a white solid and 45.1 mg (34%) compound **S67** and **S67'** as a colorless oil, which is a 5.6: 1 mixture of isomers.

Compound S66: Mp: 128-130 °C; ^{1}H NMR (400 MHz, $CDCl_3$) δ 7.77 (s, 1H), 7.37-7.27 (m, 6H), 7.24 (d, J = 8.0 Hz, 2H), 7.12 (d, J = 8.0 Hz, 2H), 6.82 (d, J = 10.4 Hz, 1H), 6.48 (s, 1H), 5.02 (dd, J_1 = 12.0, J_2 = 6.4 Hz, 1H), 4.02 (s, 3H), 3.99 (s, 3H), 3.92 (s, 3H), 3.81 (s, 3H), 2.42-2.37 (m, 1H), 2.36 (s, 3H), 2.23 (td, J_1 = 13.2, J_2 = 6.8 Hz, 1H), 1.93-1.82 (m, 1H), 1.33 (td, J_1 = 12.4, J_2 = 6.8 Hz, 1H) ppm; ^{13}C NMR (100 MHz, $CDCl_3$) δ 179.4, 164.3, 153.7, 151.7, 151.4, 144.0, 142.1, 137.1, 136.1, 135.8, 134.8, 134.1, 133.3, 132.9, 129.5, 129.1, 129.0, 128.1, 125.8, 112.0, 107.5, 61.8, 61.6, 61.2, 56.5, 56.3, 37.3, 30.4, 21.7 ppm; IR (thin film) 3418, 3334, 2956, 2839, 2424, 1693, 1595, 1487, 1442, 1409, 1330, 1233, 1163, 1099, 987, 859, 788, 694, 542, 472 (cm^{-1}); HRMS (ESI) m/z: calcd for $[C_{33}H_{34}NO_7S]^+$ 588.2050; found 588.2053.

Compounds S67 and S67': ^{1}H NMR (400 MHz, $CDCl_3$) δ 7.75 (d, J = 8.4 Hz, 2H), 7.47-7.34 (m, 3.08H), 7.33-7.27 (m, 1.18H), 7.23-7.06 (m, 3.08H), 6.96-6.90 (m, 2H), 6.77 (t, J = 8.0 Hz, 2H), 6.63 (d, J = 9.2 Hz, 0.18H), 6.53 (d, J = 7.6 Hz, 2H), 6.47 (s, 0.18H), 6.39 (s, 1H), 6.08 (d, J = 9.6 Hz, 1H), 5.54 (s, 0.18H), 5.32 (dd, J_1 = 11.2, J_2 = 8.8 Hz, 1H), 4.72 (d, J = 9.6 Hz, 1H), 4.61 (dd, J_1 = 12.8, J_2 = 6.0 Hz, 1H), 4.52 (s, 1H), 4.47 (d, J = 9.2 Hz, 0.18H), 3.98 (s, 0.54H), 3.87 (s, 0.54H), 3.85 (s, 0.54H), 3.82 (s, 3H), 3.77 (s, 3H), 3.57 (s, 0.54H), 3.35 (s, 3H), 2.64-2.56 (m, 2.18H), 2.54 (s, 3H), 2.50 (s, 3H), 2.44 (dd, J_1 = 14.0, J_2 = 7.6 Hz, 1H), 2.34 (s, 0.54H), 2.18-2.06 (m, 0.36H), 1.90-1.81 (m, 1H), 1.71-1.65 (m, 0.18H) ppm; ^{13}C NMR (100 MHz, $CDCl_3$) δ 187.0, 152.9, 152.6, 147.4, 146.9, 143.9, 143.7, 140.8, 139.4, 138.1, 135.7, 135.5, 133.6, 132.5, 130.0, 128.7, 128.3, 128.1, 127.3, 126.9, 125.9, 124.4, 122.0, 120.9, 106.8, 66.4, 63.9, 61.7, 61.4, 56.0, 54.7, 48.3, 35.7, 31.3, 21.8 ppm; IR (thin film) 3432, 2976, 2851, 1685, 1654, 1491, 1458, 1342, 1162, 1128, 1093, 861, 700, 661, 547, 475 (cm^{-1}); HRMS (ESI) m/z: calcd for $[C_{39}H_{37}NNaO_7S]^+$ 686.2183; found 686.2191.

The structure of **S67** was confirmed by NOESY experiment, as the following interaction was observed:

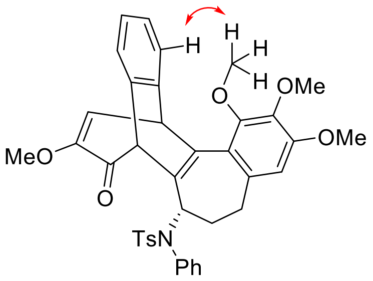

S67

Reaction of 1a with compound sub-11:

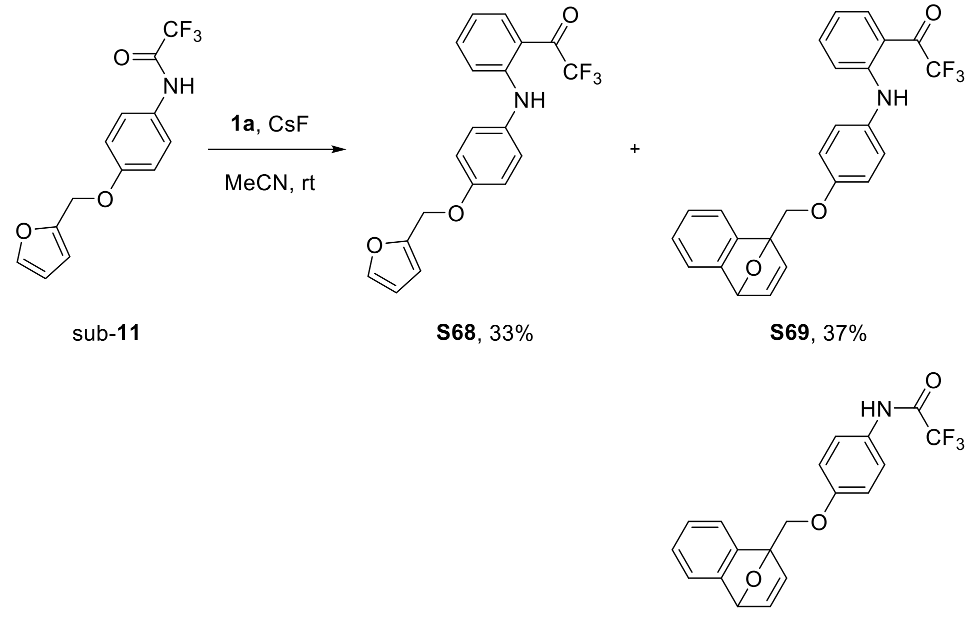

sub-**11** **S68**, 33% **S69**, 37% **S70**, 20%

A mixture of aryne precursor **1a** (89.5 mg, 0.3 mmol, 1.5 equiv), compound sub-**11** (57.0 mg, 0.2 mmol, 1.0 equiv), and CsF (91.1 mg, 0.6 mmol, 3.0 equiv) in anhydrous MeCN (2.0 mL) under inert atmosphere was stirred at room temperature overnight. The resulting mixture was filtered through a short pad of silica gel (EtOAc eluent, 20 mL). All the volatiles were removed on a rotary evaporator. Flash column chromatography (pet ether: EtOAc = 20: 1 to 4: 1) afforded 23.8 mg (33%) of compound **S68** as a yellow solid, 32.4 mg (37%) of compound **S69** as a yellow solid, and 14.5 mg (20%) of compound **S70** as a colorless oil.

Compound S68: Mp: 91-93 °C; ^{1}H NMR (400 MHz, $CDCl_3$) δ 10.12 (s, 1H), 7.83 (d, J = 8.4 Hz, 1H), 7.47 (dd, J_1 = 2.0, J_2 = 0.8 Hz, 1H), 7.36 (t, J = 8.0 Hz, 1H), 7.19 (d, J = 8.8 Hz, 2H), 7.03 (d, J = 8.8 Hz, 2H), 7.00 (d, J = 8.8 Hz, 1H), 6.72 (t, J = 8.4Hz, 1H), 6.46 (d, J = 3.2 Hz, 1H), 6.40 (dd, J_1 = 3.2, J_2 = 1.6 Hz, 1H), 5.03 (s, 2H) ppm; ^{13}C NMR (100 MHz, $CDCl_3$) δ 181.1 (q, J = 33.2 Hz), 156.7, 152.7, 150.3, 143.4, 137.1, 132.3, 132.1 (q, J = 4.1 Hz), 127.0, 117.4 (q, J = 289.3 Hz), 116.6, 116.2, 114.5, 111.5, 110.8, 110.3, 63.0 ppm; IR (thin film) 3293, 2928, 1645, 1591, 1570, 1518, 1432, 1363, 1336, 1287, 1196, 1152, 1127, 1111, 995, 939, 836, 749, 669, 600, 512 (cm^{-1}); HRMS (ESI) m/z: calcd for $[C_{19}H_{15}F_3NO_3]^+$ 362.0999; found 362.1003.

Compound S69: Mp: 99-101 °C; ^{1}H NMR (400 MHz, $CDCl_3$) δ 10.14 (s, 1H), 7.86-7.81 (m, 1H), 7.37 (t, J = 8.4 Hz, 1H), 7.28 (dd, J_1 = 5.2, J_2 = 2.8 Hz, 2H), 7.23 (d, J = 8.8 Hz, 2H), 7.14 (dd, J_1 = 5.2, J_2 = 1.6 Hz, 1H), 7.10 (d, J = 8.8 Hz, 2H), 7.05-7.01 (m, 4H), 6.73 (t, J = 8.4 Hz, 1H), 5.79 (d, J = 2.0 Hz, 1H), 4.85 (d, J = 10.4 Hz, 1H), 4.75 (d, J = 10.4 Hz, 1H) ppm; ^{13}C NMR (100 MHz, $CDCl_3$) δ 181.1 (q, J = 33.2 Hz), 157.1, 152.7, 150.3, 148.6, 144.8, 142.6, 137.1, 132.4, 132.1 (q, J = 4.1 Hz), 127.1, 125.5, 125.3, 120.5, 119.9, 117.4 (q, J = 289.2 Hz), 116.6, 116.0, 114.5, 111.6, 91.4, 82.7, 66.4 ppm; IR (thin film) 2926, 2856, 2812, 1652, 1614, 1570, 1513, 1459, 1240, 1192, 1160, 1124, 1047, 939, 836, 751, 690,

669, 642, 515 (cm^{-1}); HRMS (ESI) m/z: calcd for $[C_{25}H_{18}F_3NNaO_3]^+$ 460.1131; found 460.1129.
Compound S70: ^1H NMR (400 MHz, $CDCl_3$) δ 7.88 (s, 1H), 7.51 (d, *J* = 8.8 Hz, 2H), 7.28 (t, *J* = 2.8 Hz, 1H), 7.24 (t, *J* = 2.4 Hz, 1H), 7.12 (dd, J_1 = 5.6, J_2 = 2.0 Hz, 1H), 7.06 (d, *J* = 9.2 Hz, 2H), 7.03-6.99 (m, 3H), 5.77 (d, *J* = 2.0 Hz, 1H), 4.82 (d, *J* = 10.8 Hz, 1H), 4.72 (d, *J* = 10.8 Hz, 1H) ppm; ^{13}C NMR (100 MHz, $CDCl_3$) δ 157.1, 154.9 (q, *J* = 36.8 Hz), 150.2, 148.4, 144.8, 142.5, 128.8, 125.5, 125.3, 122.5, 120.5, 119.8, 116.0 (q, *J* = 286.8 Hz), 115.6, 91.3, 82.7, 66.3 ppm; IR (thin film) 3137, 3071, 2925, 1717, 1611, 1554, 1512, 1455, 1421, 1286, 1237, 1199, 1155, 1050, 975, 901, 830, 761, 692, 643, 516 (cm^{-1}); HRMS (ESI) m/z: calcd for $[C_{19}H_{14}F_3NNaO_3]^+$ 384.0818; found 384.0815.

Reaction of 1a with compound sub-12:

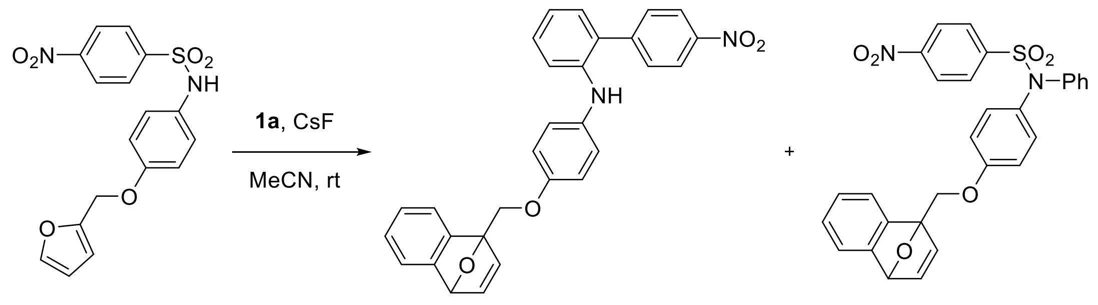

sub-**12** **S71**, 29% **S72**, 40%

A mixture of aryne precursor **1a** (89.5 mg, 0.3 mmol, 1.5 equiv), compound sub-**12** (74.9 mg, 0.2 mmol, 1.0 equiv), and CsF (91.1 mg, 0.6 mmol, 3.0 equiv) in anhydrous MeCN (2.0 mL) under inert atmosphere was stirred at room temperature overnight. The resulting mixture was filtered through a short pad of silica gel (EtOAc eluent, 20 mL). All the volatiles were removed on a rotary evaporator. Flash column chromatography (pet ether: EtOAc = 8: 1) afforded 26.8 mg (29%) of compound **S71** as a red solid and 42.1 mg (40%) of compound **S72** as a white solid.
Compound S71: Mp: 45-47 °C; ^1H NMR (400 MHz, $CDCl_3$) δ 8.30 (d, *J* = 8.8 Hz, 2H), 7.69 (d, *J* = 9.2 Hz, 2H), 7.30-7.27 (m, 2H), 7.22 (dd, J_1 = 7.6, J_2 = 1.2 Hz, 1H), 7.17 (dd, J_1 = 8.4, J_2 = 0.8 Hz, 1H), 7.12 (dd, J_1 = 5.6, J_2 = 2.0 Hz, 1H), 7.07-6.93 (m, 9H), 5.77 (d, *J* = 2.0 Hz, 1H), 5.36 (brs, 1H), 4.80 (d, *J* = 10.8 Hz, 1H), 4.70 (d, *J* = 10.8 Hz, 1H) ppm; ^{13}C NMR (100 MHz, $CDCl_3$) δ 155.0, 150.3, 148.7, 147.3, 146.6, 144.7, 142.7, 142.1, 136.3, 130.8, 130.5, 129.9, 128.0, 125.5, 125.3, 124.4, 122.6, 120.7, 120.5, 119.9, 116.5, 116.0, 91.4, 82.7, 66.6 ppm; IR (thin film) 3581, 3406, 2975, 2925, 1600, 1510, 1453, 1401, 1347, 1225, 1107, 1005, 976, 854, 823, 746, 691, 641, 541, 475 (cm^{-1}); HRMS (ESI) m/z: calcd for $[C_{29}H_{22}N_2NaO_4]^+$ 485.1472; found 485.1468.
Compound S72: Mp: 172-174 °C; ^1H NMR (400 MHz, $CDCl_3$) δ 8.34 (d, *J* = 8.8 Hz, 2H), 7.88 (d, *J* = 8.8 Hz, 2H), 7.37-7.29 (m, 3H), 7.28-7.26 (m, 2H), 7.25-7.19 (m, 4H), 7.11 (dd, J_1 = 5.6, J_2 = 1.6 Hz, 1H), 7.03-6.98 (m, 5H), 5.76 (d, *J* = 2.0 Hz, 1H), 4.80 (d, *J* = 10.8 Hz, 1H), 4.71 (d, *J* = 10.8 Hz, 1H) ppm; ^{13}C NMR (100 MHz, $CDCl_3$) δ 158.8, 150.3, 150.2, 148.4, 146.3, 144.8, 142.5, 141.3, 134.0, 130.4, 129.8, 129.2, 128.2, 128.1, 125.6, 125.3, 124.4, 120.6, 119.8, 115.8, 91.2, 82.7, 66.3 ppm; IR (thin film) 3430, 3112, 2961, 2925, 2854, 1605, 1530, 1505, 1451, 1354, 1239, 1166, 1092, 978, 856, 760, 740, 691, 607, 566, 538, 469 (cm^{-1}); HRMS (ESI) m/z: calcd for $[C_{29}H_{22}N_2NaO_6S]^+$ 549.1091; found 549.1094.

Reaction of 1a with compound sub-13:

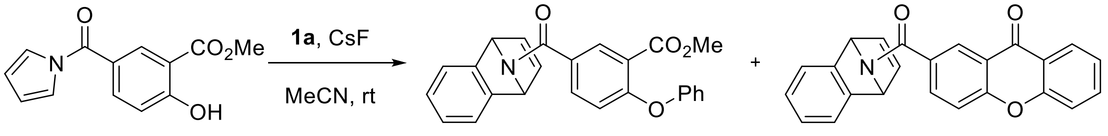

sub-**13** **S73**, 19% **S74**, 38%

A mixture of aryne precursor **1a** (89.5 mg, 0.3 mmol, 1.5 equiv), compound sub-**13** (49.0 mg, 0.2 mmol, 1.0 equiv), and CsF (91.1 mg, 0.6 mmol, 3.0 equiv) in anhydrous MeCN (2.0 mL) under inert atmosphere was stirred at room temperature overnight. The resulting mixture was filtered through a short pad of silica

gel (EtOAc eluent, 20 mL). All the volatiles were removed on a rotary evaporator. Flash column chromatography (pet ether: EtOAc = 10: 1) afforded 15.1 mg (19%) of compound **S73** as a white solid and 27.8 mg (38%) of compound **S74** as a white solid.

Compound S73: Mp: 133-135 °C; ^{1}H NMR (400 MHz, $CDCl_3$) δ 8.10 (d, J = 2.4 Hz, 1H), 7.60 (dd, J_1 = 8.8, J_2 = 2.4 Hz, 1H), 7.40-7.35 (m, 3H), 7.23-7.14 (m, 3H), 7.05-7.02 (m, 2H), 7.02-6.98 (m, 2H), 7.96-7.90 (m, 2H), 5.98 (s, 1H), 5.57 (s, 1H), 3.85 (s, 3H) ppm; ^{13}C NMR (100 MHz, $CDCl_3$) δ 166.3, 165.7, 159.1, 156.5, 148.2, 147.9, 144.8, 142.4, 133.4, 132.1, 130.2, 129.1, 125.7, 125.4, 124.5, 122.5, 121.8, 120.6, 119.6, 119.5, 68.6, 64.3, 52.6 ppm; IR (thin film) 3433, 2956, 2864, 2367, 1702, 1636, 1477, 1399, 1384, 1336, 1243, 1086, 982, 857, 760, 692, 543, 474 (cm^{-1}); HRMS (ESI) m/z: calcd for $[C_{25}H_{20}NO_4]^+$ 398.1387; found 398.1394.

Compound S74: Mp: 139-141 °C; ^{1}H NMR (400 MHz, $CDCl_3$) δ 8.53 (d, J = 2.0 Hz, 1H), 8.36 (dd, J_1 = 8.0, J_2 = 1.6 Hz, 1H), 7.94 (dd, J_1 = 8.8, J_2 = 2.4 Hz, 1H), 7.77 (ddd, J_1 = 8.8, J_2 = 7.2, J_3 = 1.6 Hz, 1H), 7.57-7.52 (m, 2H), 7.46-7.37 (m, 2H), 7.25-7.16 (m, 2H), 7.04-6.95 (m, 3H), 6.03 (s, 1H), 5.61 (s, 1H) ppm; ^{13}C NMR (100 MHz, $CDCl_3$) δ 176.9, 166.0, 157.8, 156.3, 148.2, 147.9, 144.6, 142.5, 135.5, 134.9, 130.4, 127.0, 126.9, 125.7, 125.5, 124.7, 122.1, 121.9, 121.5, 120.7, 119.0, 118.3, 68.6, 64.3 ppm; IR (thin film) 3433, 2970, 2927, 2848, 2341, 1661, 1609, 1489, 1466, 1398, 1314, 1254, 1108, 983, 938, 861, 786, 756, 672, 541, 471 (cm^{-1}); HRMS (ESI) m/z: calcd for $[C_{24}H_{16}NO_3]^+$ 366.1125; found 366.1126.

Reaction of 1a with Furosemide methyl ester sub-14:

A mixture of aryne precursor **1a** (89.5 mg, 0.3 mmol, 1.5 equiv), Furosemide methyl ester sub-**14** (69.0 mg, 0.2 mmol, 1.0 equiv), and CsF (91.1 mg, 0.6 mmol, 3.0 equiv) in anhydrous MeCN (2.0 mL) under inert atmosphere was stirred at room temperature overnight. The resulting mixture was filtered through a short pad of silica gel (EtOAc eluent, 20 mL). All the volatiles were removed on a rotary evaporator. Flash column chromatography (pet ether: EtOAc = 10: 1 to 3: 1) afforded 13.5 mg (16%) of compound **S75** as a colorless oil, 10.9 mg (11%) of compound **S76** as a colorless oil, 19.9 mg (20%) of compound **S77** as a white solid, and 14.9 mg (13%) of compound **S78** as a white solid.

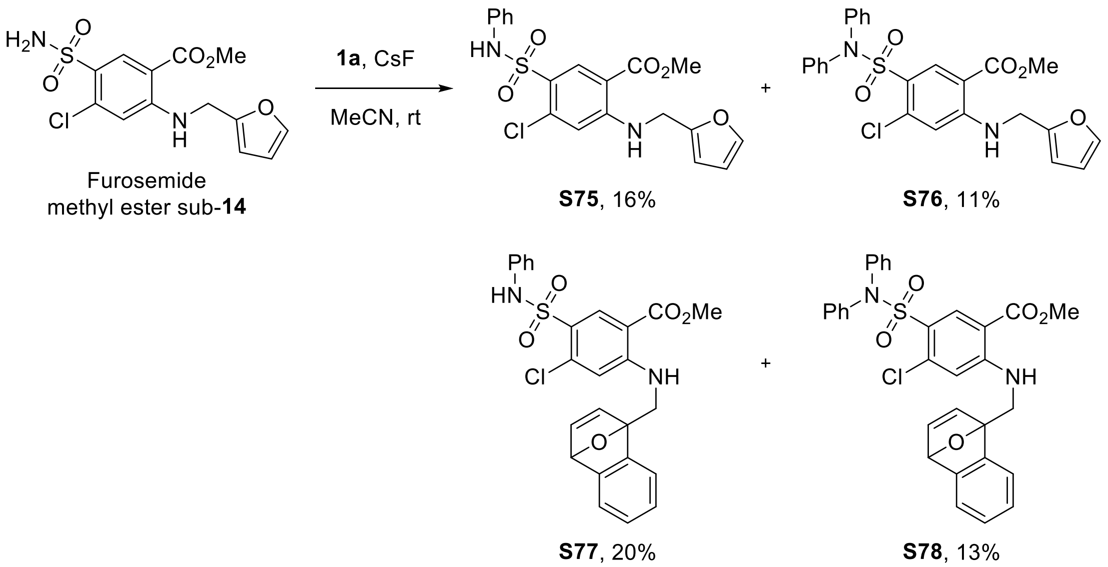

Compound S75: ^{1}H NMR (400 MHz, $CDCl_3$) δ 8.62 (t, J = 5.6 Hz, 1H), 8.51 (s, 1H), 7.38 (dd, J_1 = 2.0, J_2 = 0.8 Hz, 1H), 7.25-7.20 (m, 2H), 7.13-7.07 (m, 3H), 6.85 (brs, 1H), 6.81 (s, 1H), 6.34 (dd, J_1 = 3.2, J_2 = 1.6 Hz, 1H), 6.26-6.24 (m, 1H), 4.40 (d, J = 5.6 Hz, 2H), 3.84 (s, 3H) ppm; ^{13}C NMR (100 MHz, $CDCl_3$) δ 167.7, 153.4, 150.2, 142.9, 137.3, 136.7, 136.4, 129.6, 125.6, 121.7, 121.4, 113.7, 110.7, 108.7, 108.1, 52.3, 40.5 ppm; IR (thin film) 3422, 2919, 2846, 2360, 1712, 1637, 1400, 1385, 1094, 984, 861, 665, 543, 474 (cm^{-1}); HRMS (ESI) m/z: calcd for $[C_{19}H_{17}ClN_2NaO_5S]^+$ 443.0439; found 443.0444.

Compound S76: ^{1}H NMR (400 MHz, $CDCl_3$) δ 8.63 (t, J = 5.2 Hz, 1H), 8.46 (s, 1H), 7.42-7.38 (m, 5H), 7.31-7.27 (m, 4H), 7.24-7.19 (m, 2H), 6.86 (s, 1H), 6.36 (dd, J_1 = 3.2, J_2 = 2.0 Hz, 1H), 6.28 (d, J = 3.6 Hz, 1H), 4.44 (d, J = 5.6 Hz, 2H), 3.81 (s, 3H) ppm; ^{13}C NMR (100 MHz, $CDCl_3$) δ 167.8, 153.3, 150.3, 142.9, 141.6, 138.9, 137.7, 129.4, 128.9, 127.6, 122.8, 113.9, 110.7, 108.6, 108.1, 52.3, 40.5 ppm; IR (thin

film) 3420, 2922, 2849, 2338, 1635, 1402, 1384, 1091, 984, 862, 758, 623, 543, 475 (cm^{-1}); HRMS (ESI) m/z: calcd for $[C_{25}H_{21}ClN_2NaO_5S]^+$ 519.0752; found 519.0757.

Compound S77: Mp: 236-238 °C; ^1H NMR (400 MHz, $CDCl_3$) δ 8.64 (t, J = 4.4 Hz, 1H), 8.51 (s, 1H), 7.26-7.21 (m, 3H), 7.17 (dd, J_1 = 4.8, J_2 = 2.8 Hz, 1H), 7.15-7.13 (m, 2H), 7.12-7.06 (m, 2H), 7.02-6.99 (m, 2H), 6.91 (s, 1H), 6.86-6.84 (m, 2H), 5.77 (d, J = 1.6 Hz, 1H), 4.15 (dd, J_1 = 13.6, J_2 = 5.2 Hz, 1H), 4.03 (dd, J_1 = 13.6, J_2 = 4.4 Hz, 1H), 3.82 (s, 3H) ppm; ^{13}C NMR (100 MHz, $CDCl_3$) δ 167.6, 153.8, 150.5, 148.1, 146.2, 142.2, 137.4, 136.8, 136.4, 129.6, 125.7, 125.6, 125.4, 121.6, 121.4, 120.7, 119.2, 113.8, 108.9, 91.5, 82.7, 52.4, 42.5 ppm; IR (thin film) 3433, 2928, 2854, 2366, 1684, 1593, 1492, 1399, 1384, 1345, 1131, 1091, 990, 860, 693, 660, 544, 473 (cm^{-1}); HRMS (ESI) m/z: calcd for $[C_{25}H_{21}ClN_2NaO_5S]^+$ 519.0752; found 519.0764.

Compound S78: Mp: 92-94 °C; ^1H NMR (400 MHz, $CDCl_3$) δ 8.63 (t, J = 4.4 Hz, 1H), 8.46 (s, 1H), 7.43-7.39 (m, 4H), 7.32-7.27 (m, 6H), 7.24-7.19 (m, 3H), 7.16 (dd, J_1 = 5.6, J_2 = 2.0 Hz, 1H), 7.04-7.01 (m, 2H), 6.95 (s, 1H), 6.88 (d, J = 5.6 Hz, 1H), 5.79 (d, J = 1.6 Hz, 1H), 4.20 (dd, J_1 = 13.2, J_2 = 5.2 Hz, 1H), 4.07 (dd, J_1 = 13.2, J_2 = 4.4 Hz, 1H), 3.78 (s, 3H) ppm; ^{13}C NMR (100 MHz, $CDCl_3$) δ 167.6, 153.7, 150.5, 148.1, 146.3, 142.3, 141.6, 138.9, 137.7, 129.4, 128.9, 127.6, 125.7, 125.4, 122.7, 120.7, 119.2, 114.0, 108.7, 91.5, 82.7, 52.3, 42.5 ppm; IR (thin film) 3422, 2922, 2369, 1691, 1637, 1594, 1490, 1384, 1227, 1089, 987, 862, 757, 698, 586, 543, 475 (cm^{-1}); HRMS (ESI) m/z: calcd for $[C_{31}H_{25}ClN_2NaO_5S]^+$ 595.1065; found 595.1070.

Reaction of 1a with compound sub-15:

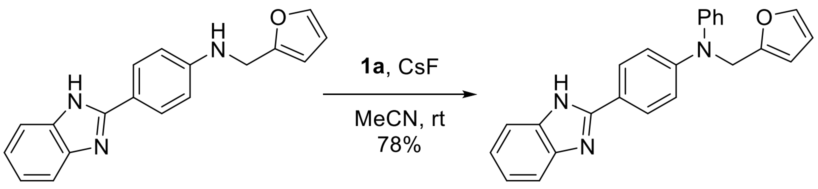

sub-**15** **24**

A mixture of aryne precursor **1a** (223.8 mg, 0.75 mmol, 1.5 equiv), compound sub-**15** (144.7 mg, 0.5 mmol, 1.0 equiv), and CsF (227.9 mg, 1.5 mmol, 3.0 equiv) in anhydrous MeCN (5.0 mL) under inert atmosphere was stirred at room temperature overnight. The resulting mixture was filtered through a short pad of silica gel (EtOAc eluent, 20 mL). All the volatiles were removed on a rotary evaporator. Flash column chromatography (DCM: MeOH = 30: 1) afforded 142.5 mg (78%) of compound **24** as a white solid. Mp: 189-191 °C; ^1H NMR (400 MHz, DMSO-*d6*) δ 12.64 (s, 1H), 7.99 (d, J = 8.8 Hz, 2H), 7.60 (d, J = 0.8 Hz, 1H), 7.53 (brs, 2H), 7.39 (t, J = 8.0 Hz, 2H), 7.26 (d, J = 7.6 Hz, 2H), 7.17-7.12 (m, 3H), 7.05 (d, J = 8.8 Hz, 2H), 6.37 (dd, J_1 = 3.2, J_2 = 2.0 Hz, 1H), 6.33 (d, J = 2.8 Hz, 1H), 5.00 (s, 2H) ppm; ^{13}C NMR (100 MHz, DMSO-*d6*) δ 151.6, 151.5, 148.7, 146.3, 142.4, 129.6, 127.4, 124.3, 124.0, 121.6, 120.9, 117.0, 110.4, 108.1, 48.5 ppm; IR (thin film) 3054, 2854, 1610, 1592, 1498, 1475, 1438, 1398, 1364, 1317, 1255, 1012, 970, 917, 810, 745, 691, 649, 596, 511 (cm^{-1}); HRMS (ESI) m/z: calcd for $[C_{24}H_{20}N_3O]^+$ 366.1601; found 366.1609.

Reaction of benzyne precursor 1a with compound sub-16: The reaction between Kobayashi benzyne precursor **1a** and compound sub-**16** has been examined under various conditions, such as in MeCN, THF, and DCM. However, only complex mixtures were obtained.

Reaction of 1b with compound sub-1:

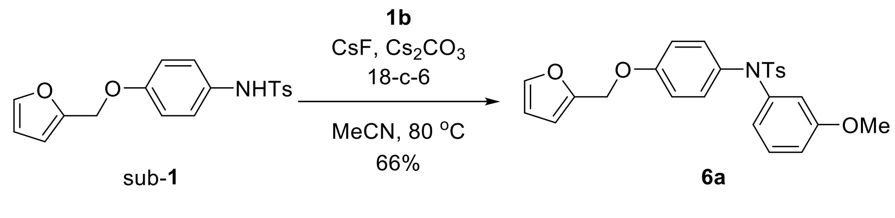

sub-**1** **6a**

A mixture of aryne precursor **1b** (98.5 mg, 0.3 mmol, 1.5 equiv), compound sub-**1** (68.7 mg, 0.2 mmol, 1.0 equiv), 18-crown-6 (105.7 mg, 0.4 mmol, 2.0 equiv), Cs_2CO_3 (130.3 mg, 0.4 mmol, 2.0 equiv), and CsF (91.1 mg, 0.6 mmol, 3.0 equiv) in anhydrous MeCN (2.0 mL) under inert atmosphere was stirred at 80 °C overnight. The resulting mixture was filtered through a short pad of silica gel (EtOAc eluent, 20 mL). All the volatiles were removed on a rotary evaporator. Flash column chromatography (pet ether: DCM = 1: 1) afforded 59.3 mg (66%) of compound **6a** as a white solid. Mp: 103-105 °C; ^{1}H NMR (400 MHz, $CDCl_3$) δ 7.59 (d, J = 8.4 Hz, 2H), 7.45 (d, J_1 = 1.6 Hz, 1H), 7.26 (d, J = 8.4 Hz, 2H), 7.20-7.15 (m, 3H), 6.91 (d, J = 9.2 Hz, 2H), 6.84-6.80 (m, 2H), 6.79-6.75 (m, 1H), 6.43 (d, J = 3.2 Hz, 1H), 6.38 (dd, J_1 = 3.2, J_2 = 2.0 Hz, 1H), 4.97 (s, 2H), 3.74 (s, 3H), 2.43 (s, 3H) ppm; ^{13}C NMR (100 MHz, $CDCl_3$) δ 160.2, 158.0, 150.1, 143.7, 143.4, 143.2, 137.7, 134.6, 130.4, 129.9, 129.7, 128.1, 119.9, 115.6, 113.8, 112.8, 110.8, 110.4, 62.8, 55.5, 21.8 ppm; IR (thin film) 3116, 2920, 2837, 1599, 1506, 1448, 1348, 1313, 1232, 1169, 1094, 1035, 997, 953, 884, 819, 746, 692, 653, 596, 554 (cm^{-1}); HRMS (ESI) m/z: calcd for $[C_{25}H_{23}NNaO_5S]^+$ 472.1189; found 472.1198.

Reaction of 1b with compound sub-2:

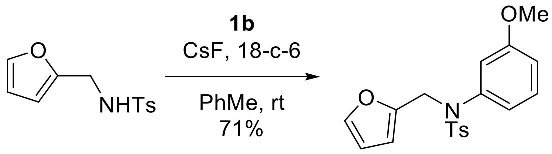

sub-**2** **7a**

A mixture of aryne precursor **1b** (98.5 mg, 0.3 mmol, 1.5 equiv), sub-**2** (50.3 mg, 0.2 mmol, 1.0 equiv), 18-crown-6 (105.7 mg, 0.4 mmol, 2.0 equiv), and CsF (91.1 mg, 0.6 mmol, 3.0 equiv) in anhydrous toluene (2.0 mL) under inert atmosphere was stirred at room temperature overnight. The resulting mixture was filtered through a short pad of silica gel (EtOAc eluent, 20 mL). All the volatiles were removed on a rotary evaporator. Flash column chromatography (pet ether: EtOAc = 8: 1) afforded 50.8 mg (71%) of **7a** as a colorless oil. ^{1}H NMR (400 MHz, $CDCl_3$) δ 7.53 (d, J = 8.4 Hz, 2H), 7.26 (s, 1H), 7.24 (d, J = 8.0 Hz, 2H), 7.14 (t, J = 8.4 Hz, 1H), 6.80 (dd, J_1 = 8.0, J_2 = 0.8 Hz, 1H), 6.60-6.56 (m, 2H), 6.23-6.19 (m, 1H), 6.10 (d, J = 3.2 Hz, 1H), 4.75 (s, 2H), 3.69 (s, 3H), 2.42 (s, 3H) ppm; ^{13}C NMR (100 MHz, $CDCl_3$) δ 160.0, 149.8, 143.6, 142.6, 140.5, 136.2, 129.6, 129.5, 127.9, 121.1, 115.0, 114.2, 110.5, 109.8, 55.4, 48.2, 21.7 ppm; IR (thin film) 3120, 3002, 2940, 2836, 1600, 1485, 1451, 1349, 1286, 1162, 1092, 1043, 932, 813, 745, 692, 658, 571, 546 (cm^{-1}); HRMS (ESI) m/z: calcd for $[C_{19}H_{20}NO_3S]^+$ 342.1158; found 342.1164.

Reaction of 1b with compound sub-3:

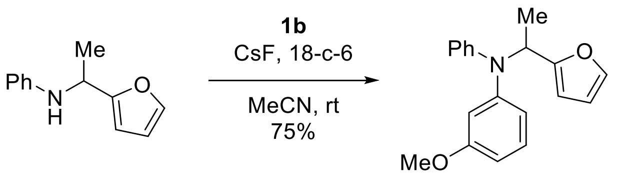

sub-**3** **8a**

A mixture of aryne precursor **1b** (98.5 mg, 0.3 mmol, 1.5 equiv), compound sub-**3** (37.4 mg, 0.2 mmol, 1.0 equiv), 18-crown-6 (105.7 mg, 0.4 mmol, 2.0 equiv), and CsF (91.1 mg, 0.6 mmol, 3.0 equiv) in anhydrous MeCN (2.0 mL) under inert atmosphere was stirred at room temperature overnight. The resulting mixture was filtered through a short pad of silica gel (EtOAc eluent, 20 mL). All the volatiles were removed on a rotary evaporator. Flash column chromatography (pet ether: EtOAc = 50: 1) afforded 44.0 mg (75%) of compound **8a** as a colorless oil. ^{1}H NMR (400 MHz, $CDCl_3$) δ 7.35 (s, 1H), 7.25 (t, J = 8.4 Hz, 2H), 7.12 (t, J = 8.4 Hz, 1H), 7.03 (t, J = 7.2 Hz, 1H), 6.88 (d, J = 7.6 Hz, 2H), 6.49 (dd, J_1 = 8.4, J_2 = 2.0 Hz, 1H), 6.43 (dd, J_1 = 8.4, J_2 = 1.6 Hz, 1H), 6.35 (t, J = 2.0 Hz, 1H), 6.27 (dd, J_1 = 2.8, J_2 = 1.6 Hz, 1H), 6.06 (d, J = 2.8 Hz, 1H), 5.37 (q, J = 6.8 Hz, 1H), 3.70 (s, 3H), 1.52 (d, J = 6.8 Hz, 3H) ppm; ^{13}C NMR (100 MHz, $CDCl_3$) δ 160.6, 156.2, 148.4, 145.9, 141.6, 129.8, 129.3, 124.6, 123.1, 114.0, 110.3, 107.3, 107.2, 106.5, 55.3, 51.5, 17.0 ppm; IR (thin film) 3444, 2935, 2833, 1591, 1492, 1452, 1259, 1206,

1167, 1044, 748, 701, 598 (cm^{-1}); HRMS (ESI) m/z: calcd for $[C_{19}H_{20}NO]^+$ 278.1539; found 278.1538.

Reaction of 1b with compound sub-4:

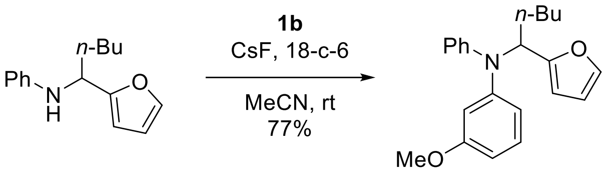

sub-**4** **9a**

A mixture of aryne precursor **1b** (98.5 mg, 0.3 mmol, 1.5 equiv), compound sub-**4** (45.9 mg, 0.2 mmol, 1.0 equiv), 18-crown-6 (105.7 mg, 0.4 mmol, 2.0 equiv), and CsF (91.1 mg, 0.6 mmol, 3.0 equiv) in anhydrous MeCN (2.0 mL) under inert atmosphere was stirred at room temperature overnight. The resulting mixture was filtered through a short pad of silica gel (EtOAc eluent, 20 mL). All the volatiles were removed on a rotary evaporator. Flash column chromatography (pet ether: EtOAc = 50: 1) afforded 51.7 mg (77%) of compound **9a** as a colorless oil. ^{1}H NMR (400 MHz, $CDCl_3$) δ 7.36-7.31 (m, 1H), 7.24 (t, J = 7.6 Hz, 2H), 7.12 (t, J = 8.0 Hz, 1H), 7.02 (t, J = 7.2 Hz, 1H), 6.88 (d, J = 7.6 Hz, 2H), 6.49 (dd, J_1 = 8.4, J_2 = 2.0 Hz, 1H), 6.44 (dd, J_1 = 8.4, J_2 = 1.6 Hz, 1H), 6.35 (t, J = 2.0 Hz, 1H), 6.26 (dd, J_1 = 3.2, J_2 = 2.0 Hz, 1H), 6.06 (d, J = 3.2 Hz, 1H), 5.19 (t, J = 7.2 Hz, 1H), 3.70 (s, 3H), 1.98-1.88 (m, 2H), 1.54-1.45 (m, 1H), 1.42-1.31 (m, 3H), 0.90 (t, J = 7.2 Hz, 3H) ppm; ^{13}C NMR (100 MHz, $CDCl_3$) δ 160.6, 155.4, 148.8, 146.3, 141.5, 129.7, 129.2, 124.5, 123.0, 114.1, 110.3, 107.7, 107.3, 106.4, 56.6, 55.3, 31.0, 29.1, 22.8, 14.2 ppm; IR (thin film) 3059, 2955, 2860, 1591, 1495, 1465, 1258, 1166, 1045, 805, 742, 700, 598 (cm^{-1}); HRMS (ESI) m/z: calcd for $[C_{22}H_{26}NO_2]^+$ 336.1958; found 336.1960.

Reaction of 1b with compound sub-5:

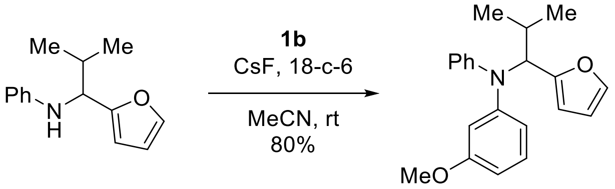

sub-**5** **10a**

A mixture of aryne precursor **1b** (98.5 mg, 0.3 mmol, 1.5 equiv), compound sub-**5** (43.1 mg, 0.2 mmol, 1.0 equiv), 18-crown-6 (105.7 mg, 0.4 mmol, 2.0 equiv), and CsF (91.1 mg, 0.6 mmol, 3.0 equiv) in anhydrous MeCN (2.0 mL) under inert atmosphere was stirred at room temperature overnight. The resulting mixture was filtered through a short pad of silica gel (EtOAc eluent, 20 mL). All the volatiles were removed on a rotary evaporator. Flash column chromatography (pet ether: EtOAc = 50: 1) afforded 51.4 mg (80%) of compound **10a** as a colorless oil. ^{1}H NMR (400 MHz, $CDCl_3$) δ 7.28 (d, J = 0.8 Hz, 1H), 7.26-7.20 (m, 2H), 7.10 (t, J = 8.4 Hz, 1H), 7.01 (t, J = 7.2 Hz, 1H), 6.94 (d, J = 7.6 Hz, 2H), 6.51-6.45 (m, 2H), 6.43 (t, J = 2.0 Hz, 1H), 6.24 (dd, J_1 = 2.8, J_2 = 1.6 Hz, 1H), 6.04 (d, J = 3.2 Hz, 1H), 4.79 (d, J = 10.8 Hz, 1H), 3.71 (s, 3H), 2.49-2.39 (m, 1H), 1.21 (d, J = 6.4 Hz, 3H), 0.93 (d, J = 6.4 Hz, 3H) ppm; ^{13}C NMR (100 MHz, $CDCl_3$) δ 160.5, 154.4, 149.6, 147.2, 141.1, 129.6, 129.2, 124.7, 122.9, 114.4, 110.1, 108.4, 107.7, 106.2, 64.3, 55.3, 30.5, 21.0, 20.9 ppm; IR (thin film) 3440, 2959, 2871, 2833, 2349, 1591, 1496, 1467, 1386, 1230, 1163, 1049, 1012, 962, 856, 735, 700, 598, 543, 478 (cm^{-1}); HRMS (ESI) m/z: calcd for $[C_{21}H_{23}NNaO_2]^+$ 344.1621; found 344.1622.

Reaction of S5 with compound sub-5:

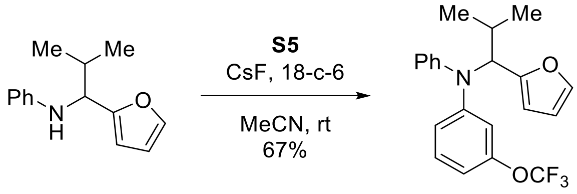

A mixture of aryne precursor **S5** (114.7 mg, 0.3 mmol, 1.5 equiv), compound sub-**5** (43.1 mg, 0.2 mmol, 1.0 equiv), 18-crown-6 (105.7 mg, 0.4 mmol, 2.0 equiv), and CsF (91.1 mg, 0.6 mmol, 3.0 equiv) in anhydrous MeCN (2.0 mL) under inert atmosphere was stirred at room temperature overnight. The resulting mixture was filtered through a short pad of silica gel (EtOAc eluent, 20 mL). All the volatiles were removed on a rotary evaporator. Flash column chromatography (pet ether: EtOAc = 50: 1) afforded 50.3 mg (67%) of compound **17a** as a colorless oil. ^{1}H NMR (400 MHz, $CDCl_3$) δ 7.33-7.27 (m, 3H), 7.17-7.09 (m, 2H), 7.01-6.96 (m, 2H), 6.70-6.62 (m, 3H), 6.24 (dd, J_1 = 3.2, J_2 = 2.0 Hz, 1H), 6.05 (d, J = 3.2 Hz, 1H), 4.75 (d, J = 10.8 Hz, 1H), 2.48-2.39 (m, 1H), 1.19 (d, J = 6.4 Hz, 3H), 0.93 (d, J = 6.4 Hz, 3H) ppm; ^{13}C NMR (100 MHz, $CDCl_3$) δ 153.7, 150.4, 150.2, 146.0, 141.4, 129.7, 129.6, 127.3, 125.0, 120.7 (q, J = 255.1 Hz), 116.9, 111.2, 111.1, 110.2, 108.7, 64.6, 30.5, 21.0, 20.9 ppm; IR (thin film) 3423, 2959, 1612, 1594, 1491, 1385, 1259, 1220, 1160, 1091, 1010, 859, 792, 741, 703, 659, 544, 484 (cm^{-1}); HRMS (ESI) m/z: calcd for $[C_{21}H_{21}F_3NO_2]^+$ 376.1519; found 376.1523.

Reaction of S6 with compound sub-5:

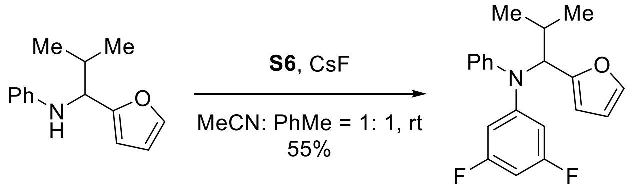

A mixture of aryne precursor **S6** (133.7 mg, 0.4 mmol, 2.0 equiv), compound sub-**5** (43.1 mg, 0.2 mmol, 1.0 equiv) and CsF (121.5 mg, 0.8 mmol, 4.0 equiv) in anhydrous MeCN-toluene (1:1, 2.0 mL) under inert atmosphere was stirred at room temperature overnight. The resulting mixture was filtered through a short pad of silica gel (EtOAc eluent, 20 mL). All the volatiles were removed on a rotary evaporator. Flash column chromatography (pet ether: EtOAc = 50: 1) afforded 36.0 mg (55%) of compound **17b** as a colorless oil. ^{1}H NMR (400 MHz, $CDCl_3$) δ 7.36-7.31 (m, 2H), 7.30-7.28 (m, 1H), 7.26-7.21 (m, 1H), 6.99-6.95 (m, 2H), 6.25 (dd, J_1 = 3.2, J_2 = 2.0 Hz, 1H), 6.24-6.18 (m, 2H), 6.17-6.12 (m, 1H), 6.04 (d, J = 3.2 Hz, 1H), 4.69 (d, J = 10.8 Hz, 1H), 2.44-2.35 (m, 1H), 1.20 (d, J = 6.4 Hz, 3H), 0.92 (d, J = 6.4 Hz, 3H) ppm; ^{13}C NMR (100 MHz, $CDCl_3$) δ 163.8 (dd, J_1 = 241.4, J_2 = 16.0 Hz), 153.3, 151.9 (t, J = 12.7 Hz), 144.3, 141.5, 129.7, 129.4, 126.6, 110.2, 108.9, 99.1-98.8 (m), 93.2 (t, J = 26.1 Hz), 64.5, 30.3, 20.9, 20.8 ppm; IR (thin film) 3432, 2962, 2927, 2358, 1633, 1592, 1480, 1385, 1111, 1074, 989, 827, 735, 706, 669, 543, 474 (cm^{-1}); HRMS (ESI) m/z: calcd for $[C_{20}H_{20}F_2NO]^+$ 328.1507; found 328.1508.

Reaction of S7 with compound sub-5:

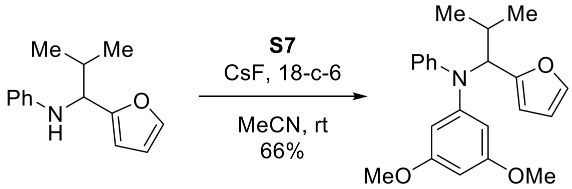

A mixture of aryne precursor **S7** (107.5 mg, 0.3 mmol, 1.5 equiv), compound sub-**5** (43.1 mg, 0.2 mmol, 1.0 equiv), 18-crown-6 (105.7 mg, 0.4 mmol, 2.0 equiv), and CsF (91.1 mg, 0.6 mmol, 3.0 equiv) in anhydrous MeCN (2.0 mL) under inert atmosphere was stirred at room temperature overnight. The resulting mixture was filtered through a short pad of silica gel (EtOAc eluent, 20 mL). All the volatiles

were removed on a rotary evaporator. Flash column chromatography (pet ether: EtOAc = 50: 1) afforded 46.4 mg (66%) of compound **17c** as a colorless oil. ^{1}H NMR (400 MHz, $CDCl_3$) δ 7.30-7.26 (m, 1H), 7.26-7.20 (m, 2H), 7.03 (t, J = 7.6 Hz, 1H), 6.97-6.93 (m, 2H), 6.26-6.22 (m, 1H), 6.06-6.02 (m, 4H), 4.77 (d, J = 10.8 Hz, 1H), 3.68 (s, 6H), 2.49-2.39 (m, 1H), 1.21 (d, J = 6.4 Hz, 3H), 0.93 (d, J = 6.4 Hz, 3H) ppm; ^{13}C NMR (100 MHz, $CDCl_3$) δ 161.3, 154.4, 150.4, 146.7, 141.1, 129.2, 125.5, 123.5, 110.2, 108.5, 99.5, 92.7, 64.3, 55.4, 30.5, 21.0, 20.9 ppm; IR (thin film) 2959, 2871, 2837, 1590, 1496, 1461, 1229, 1203, 1152, 1068, 1012, 810, 736, 707, 598 (cm^{-1}); HRMS (ESI) m/z: calcd for $[C_{22}H_{26}NO_3]^+$ 352.1907; found 352.1910.

Reaction of S8 with compound sub-5:

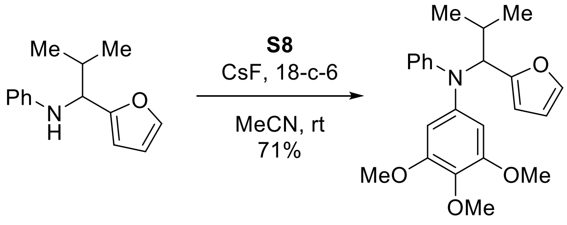

sub-**5** **17d**

A mixture of aryne precursor **S8** (116.5 mg, 0.3 mmol, 1.5 equiv), compound sub-**5** (43.1 mg, 0.2 mmol, 1.0 equiv), 18-crown-6 (105.7 mg, 0.4 mmol, 2.0 equiv), and CsF (91.1 mg, 0.6 mmol, 3.0 equiv) in anhydrous MeCN (2.0 mL) under inert atmosphere was stirred at room temperature overnight. The resulting mixture was filtered through a short pad of silica gel (EtOAc eluent, 20 mL). All the volatiles were removed on a rotary evaporator. Flash column chromatography (pet ether: EtOAc = 50: 1) afforded 54.2 mg (71%) of compound **17d** as a colorless oil. ^{1}H NMR (400 MHz, $CDCl_3$) δ 7.31-7.27 (m, 1H), 7.21-7.16 (m, 2H), 6.87-6.81 (m, 3H), 6.26 (dd, J_1 = 3.2, J_2 = 2.0 Hz, 1H), 6.16 (s, 2H), 6.06 (d, J = 3.2 Hz, 1H), 4.78 (d, J = 10.8 Hz, 1H), 3.84 (s, 3H), 3.71 (s, 6H), 2.47-2.37 (m, 1H), 1.22 (d, J = 6.4 Hz, 3H), 0.94 (d, J = 6.4 Hz, 3H) ppm; ^{13}C NMR (100 MHz, $CDCl_3$) δ 154.5, 153.5, 149.0, 142.1, 141.0, 134.9, 129.1, 119.9, 119.3, 110.3, 108.5, 104.0, 64.1, 61.2, 56.2, 30.4, 21.0, 20.9 ppm; IR (thin film) 3058, 2959, 2872, 1588, 1496, 1464, 1412, 1229, 1128, 1011, 808, 744, 705, 599 (cm^{-1}); HRMS (ESI) m/z: calcd for $[C_{23}H_{28}NO_4]^+$ 382.2013; found 382.2020.

Reaction of 1b with Fuberidazole:

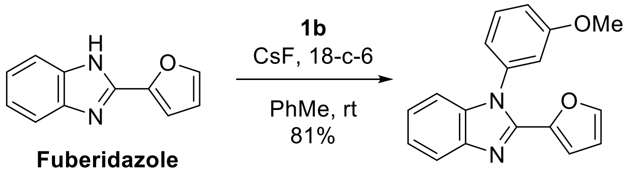

Fuberidazole **11a**

A mixture of aryne precursor **1b** (98.5 mg, 0.3 mmol, 1.5 equiv), commercially available Fuberidazole (36.8 mg, 0.2 mmol, 1.0 equiv), 18-crown-6 (105.7 mg, 0.4 mmol, 2.0 equiv), and CsF (91.1 mg, 0.6 mmol, 3.0 equiv) in anhydrous toluene (2.0 mL) under inert atmosphere was stirred at room temperature overnight. The resulting mixture was filtered through a short pad of silica gel (EtOAc eluent, 20 mL). All the volatiles were removed on a rotary evaporator. Flash column chromatography (pet ether: EtOAc = 8: 1) afforded 47.0 mg (81%) of compound **11a** as a white solid. Mp: 126-128 °C; ^{1}H NMR (400 MHz, $CDCl_3$) δ 7.86 (d, J = 8.0 Hz, 1H), 7.51-7.47 (m, 2H), 7.32 (t, J = 8.0 Hz, 1H), 7.23 (d, J = 7.2 Hz, 1H), 7.12 (d, J = 8.0 Hz, 2H), 7.00 (ddd, J_1 = 8.0, J_2 = 2.0, J_3 = 0.8 Hz, 1H), 6.93 (t, J = 2.0 Hz, 1H), 6.37 (dd, J_1 = 3.6, J_2 = 1.6 Hz, 1H), 6.22 (d, J = 3.6 Hz, 1H), 3.83 (s, 3H) ppm; ^{13}C NMR (100 MHz, $CDCl_3$) δ 160.9, 144.7, 144.4, 144.3, 143.1, 137.6, 137.3, 130.8, 123.7, 123.3, 120.3, 120.1, 115.6, 113.6, 112.4, 111.7, 110.4, 55.8 ppm; IR (thin film) 3142, 3058, 2924, 2851, 1602, 1494, 1450, 1376, 1324, 1269, 1243, 1225, 1041, 1020, 899, 844, 813, 738, 697, 595 (cm^{-1}); HRMS (ESI) m/z: calcd for $[C_{18}H_{15}N_2O_2]^+$ 291.1128; found 291.1140.

Reaction of 1b with compound sub-6:

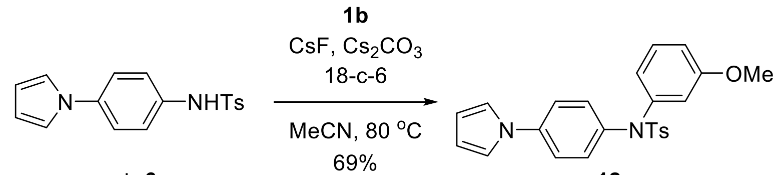

sub-**6** **12a**

A mixture of aryne precursor **1b** (98.5 mg, 0.3 mmol, 1.5 equiv), compound sub-**6** (62.5 mg, 0.2 mmol, 1.0 equiv), 18-crown-6 (105.7 mg, 0.4 mmol, 2.0 equiv), Cs_2CO_3 (130.3 mg, 0.4 mmol, 2.0 equiv), and CsF (91.1 mg, 0.6 mmol, 3.0 equiv) in anhydrous MeCN (2.0 mL) under inert atmosphere was stirred at 80 °C overnight. The resulting mixture was filtered through a short pad of silica gel (EtOAc eluent, 20 mL). All the volatiles were removed on a rotary evaporator. Flash column chromatography (pet ether: DCM = 1: 1) afforded 57.8 mg (69%) of compound **12a** as a white solid. Mp: 109-111 °C; ^{1}H NMR (400 MHz, $CDCl_3$) δ 7.62 (d, *J* = 8.0 Hz, 2H), 7.34-7.32 (m, 4H), 7.29 (d, *J* = 8.0 Hz, 2H), 7.22 (t, *J* = 8.0 Hz, 1H), 7.06-7.04 (m, 2H), 6.86-6.80 (m, 3H), 6.36-6.33 (m, 2H), 3.76 (s, 3H), 2.44 (s, 3H) ppm; ^{13}C NMR (100 MHz, $CDCl_3$) δ 160.4, 144.0, 142.7, 139.9, 138.9, 137.6, 130.1, 129.8, 129.8, 128.0, 121.1, 120.3, 119.4, 114.3, 113.2, 111.0, 55.6, 21.8 ppm; IR (thin film) 2939, 2836, 1600, 1516, 1489, 1354, 1332, 1165, 1091, 814, 724, 691, 664, 573, 547 (cm^{-1}); HRMS (ESI) m/z: calcd for $[C_{24}H_{23}N_2O_3S]^+$ 419.1424; found 419.1424.

Reaction of 1b with compound sub-7:

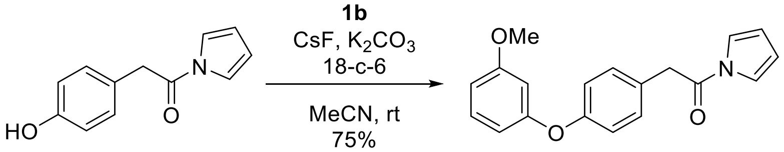

sub-**7** **13a**

A mixture of aryne precursor **1b** (98.5 mg, 0.3 mmol, 1.5 equiv), compound sub-**7** (40.2 mg, 0.2 mmol, 1.0 equiv), 18-crown-6 (105.7 mg, 0.4 mmol, 2.0 equiv), K_2CO_3 (55.3 mg, 0.4 mmol, 2.0 equiv), and CsF (91.1 mg, 0.6 mmol, 3.0 equiv) in anhydrous MeCN (2.0 mL) under inert atmosphere was stirred at room temperature overnight. The resulting mixture was filtered through a short pad of silica gel (EtOAc eluent, 20 mL). All the volatiles were removed on a rotary evaporator. Flash column chromatography (pet ether: EtOAc = 8: 1) afforded 46.1 mg (75%) of compound **13a** as a white solid. Mp: 90-92 °C; ^{1}H NMR (400 MHz, $CDCl_3$) δ 7.37 (s, 2H), 7.26-7.19 (m, 3H), 6.99 (d, *J* = 8.4 Hz, 2H), 6.68-6.64 (m, 1H), 6.61-6.55 (m, 2H), 6.38-6.24 (m, 2H), 4.12 (s, 2H), 3.77 (s, 3H) ppm; ^{13}C NMR (100 MHz, $CDCl_3$) δ 168.6, 161.2, 158.4, 156.7, 130.8, 130.4, 128.0, 119.6, 119.4, 113.6, 111.4, 109.4, 105.3, 55.6, 41.0 ppm; IR (thin film) 3421, 3262, 3146, 2917, 2375, 1716, 1606, 1464, 1400, 1356, 1318, 1270, 1219, 1140, 1112, 992, 864, 740, 695, 541, 472 (cm^{-1}); HRMS (ESI) m/z: calcd for $[C_{19}H_{17}NNaO_3]^+$ 330.1101; found 330.1109.

Reaction of 1b with compound sub-8:

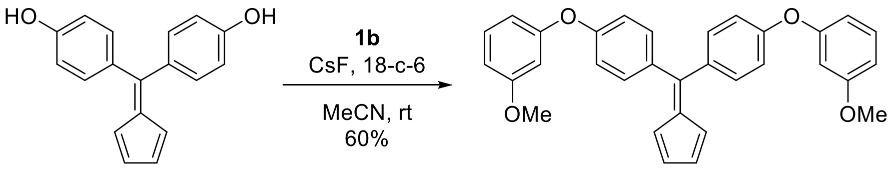

sub-**8** **14a**

A mixture of aryne precursor **1b** (131.4 mg, 0.4 mmol, 2.0 equiv), compound sub-**8** (52.5 mg, 0.2 mmol, 1.0 equiv), 18-crown-6 (105.7 mg, 0.4 mmol, 2.0 equiv), and CsF (121.5 mg, 0.8 mmol, 4.0 equiv) in

anhydrous MeCN (2.0 mL) under inert atmosphere was stirred at room temperature overnight. The resulting mixture was filtered through a short pad of silica gel (EtOAc eluent, 20 mL). All the volatiles were removed on a rotary evaporator. Flash column chromatography (pet ether) afforded 56.9 mg (60%) of compound **14a** as a red oil. ^{1}H NMR (400 MHz, $CDCl_3$) δ 7.30 (d, J = 8.4 Hz, 4H), 7.27 (d, J = 8.0 Hz, 2H), 7.01 (d, J = 8.8 Hz, 4H), 6.72 (d, J = 2.0 Hz, 1H), 6.70 (dd, J_1 = 2.4, J_2 = 0.8 Hz, 1H), 6.69-6.68 (m, 1H), 6.67-6.64 (m, 3H), 6.62-6.59 (m, 2H), 6.33-6.30 (m, 2H), 3.81 (s, 6H) ppm; ^{13}C NMR (100 MHz, $CDCl_3$) δ 161.3, 158.4, 157.7, 151.1, 143.4, 136.2, 134.1, 132.1, 130.5, 124.5, 117.7, 112.0, 109.8, 105.9, 55.6 ppm; IR (thin film) 3454, 3326, 1604, 1594, 1569, 1505, 1447, 1361, 1322, 1222, 1169, 1108, 920, 842, 805, 774, 737, 673, 560, 506 (cm^{-1}); HRMS (ESI) m/z: calcd for $[C_{32}H_{27}O_4]^+$ 475.1904; found 475.1918.

Reaction of 1b with compound sub-9:

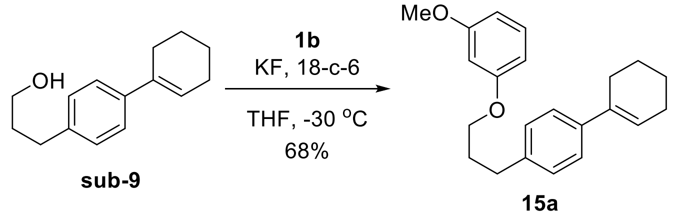

sub-9 **15a**

A mixture of aryne precursor **1b** (98.5 mg, 0.3 mmol, 1.5 equiv), compound sub-**9** (43.3 mg, 0.2 mmol, 1.0 equiv), 18-crown-6 (105.7 mg, 0.4 mmol, 2.0 equiv), and KF (46.5 mg, 0.8 mmol, 4.0 equiv) in anhydrous THF (2.0 mL) under inert atmosphere was stirred at -30 °C overnight. The resulting mixture was filtered through a short pad of silica gel (EtOAc eluent, 20 mL). All the volatiles were removed on a rotary evaporator. Flash column chromatography (pet ether) afforded 43.9 mg (68%) of compound **15a** as a white solid. Mp: 26-28 °C; ^{1}H NMR (400 MHz, $CDCl_3$) δ 7.32 (d, J = 8.4 Hz, 2H), 7.21-7.14 (m, 3H), 6.51 (dt, J_1 = 8.4, J_2 = 2.4 Hz, 2H), 6.48 (t, J = 2.4 Hz, 1H), 6.15-6.08 (m, 1H), 3.96 (t, J = 6.4 Hz, 2H), 3.80 (s, 3H), 2.80 (t, J = 7.2 Hz, 2H), 2.44-2.39 (m, 2H), 2.25-2.19 (m, 2H), 2.14-2.07 (m, 2H), 1.83-1.76 (m, 2H), 1.71-1.64 (m, 2H) ppm; ^{13}C NMR (100 MHz, $CDCl_3$) δ 161.1, 160.5, 140.6, 139.9, 136.5, 130.0, 128.6, 125.1, 124.4, 107.0, 106.4, 101.2, 67.1, 55.5, 31.9, 31.0, 27.6, 26.1, 23.3, 22.4 ppm; IR (thin film) 3433, 2928, 2833, 2346, 1602, 1492, 1452, 1385, 1334, 1287, 1265, 1201, 1153, 1081, 1049, 990, 833, 798, 761, 687, 543, 463 (cm^{-1}); HRMS (ESI) m/z: calcd for $[C_{22}H_{27}O2]^+$ 323.2006; found 323.2021.

Reaction of 1b with *N*-deacetyl *N*-tosyl Colchicine sub-10:

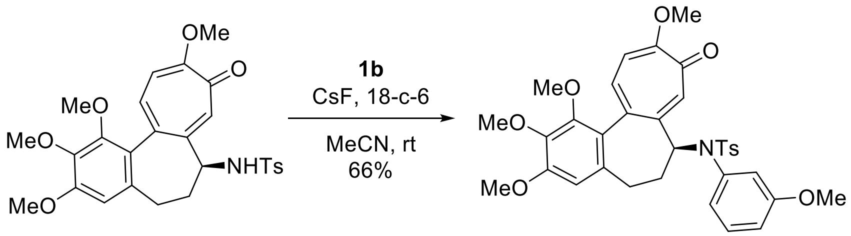

sub-**10** **16a**

A mixture of aryne precursor **1b** (98.5 mg, 0.3 mmol, 1.5 equiv), *N*-deacetyl *N*-tosyl Colchicine sub-**10** (102.3 mg, 0.2 mmol, 1.0 equiv), 18-crown-6 (105.7 mg, 0.4 mmol, 2.0 equiv), and CsF (91.1 mg, 0.6 mmol, 3.0 equiv) in anhydrous MeCN (2.0 mL) under inert atmosphere was stirred at room temperature overnight. The resulting mixture was filtered through a short pad of silica gel (EtOAc eluent, 20 mL). All the volatiles were removed on a rotary evaporator. Flash column chromatography (pet ether: EtOAc = 1: 2 to 1: 4) afforded 81.5 mg (66%) of compound **16a** as a white solid. Mp: 118-120 °C; ^{1}H NMR (400 MHz, $CDCl_3$) δ 7.77 (s, 1H), 7.33 (d, J = 10.8 Hz, 1H), 7.28 (d, J = 8.4 Hz, 2H), 7.19 (t, J = 8.0 Hz, 1H), 7.13 (d, J = 8.4 Hz, 2H), 6.91-6.88 (m, 1H), 6.87-6.83 (m, 2H), 6.81 (d, J = 10.8 Hz, 1H), 6.48 (s, 1H), 4.98 (dd, J_1 = 12.0, J_2 = 6.4 Hz, 1H), 4.01 (s, 3H), 3.99 (s, 3H), 3.92 (s, 3H), 3.81 (s, 3H), 3.77 (s, 3H), 2.41 (dd, J_1 = 14.4, J_2 = 6.4 Hz, 1H), 2.36 (s, 3H), 2.24 (td, J_1 = 12.8, J_2 = 6.8 Hz, 1H), 1.96-1.88 (m, 1H), 1.41 (td, J_1 = 12.0, J_2 = 5.6 Hz, 1H) ppm; ^{13}C NMR (100 MHz, $CDCl_3$) δ 179.4, 164.3, 159.9, 153.7,

151.6, 151.4, 144.0, 142.1, 138.2, 136.2, 135.8, 134.7, 134.2, 133.0, 129.5, 129.4, 128.1, 125.8, 125.3, 118.9, 115.1, 112.0, 107.6, 61.8, 61.6, 61.3, 56.5, 56.4, 55.7, 37.1, 30.4, 21.7 ppm; IR (thin film) 3440, 2933, 2838, 1599, 1487, 1400, 1348, 1321, 1253, 1161, 1139, 1095, 1003, 928, 856, 698, 665, 543, 480 (cm^{-1}); HRMS (ESI) m/z: calcd for $[C_{34}H_{35}NNaO_8S]^+$ 640.1976; found 640.1983.

Reaction of 1b with compound sub-11:

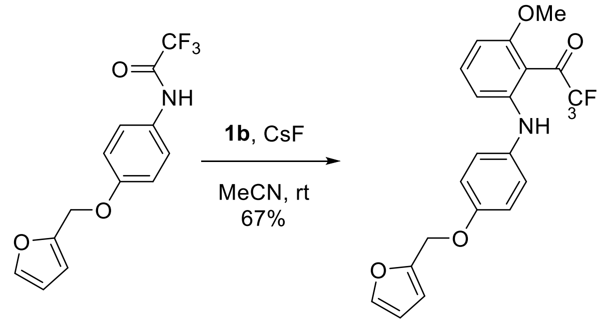

sub-**11** **20a**

A mixture of aryne precursor **1b** (98.5 mg, 0.3 mmol, 1.5 equiv), compound sub-**11** (57.0 mg, 0.2 mmol, 1.0 equiv), and CsF (91.1 mg, 0.6 mmol, 3.0 equiv) in anhydrous MeCN (2.0 mL) under inert atmosphere was stirred at room temperature overnight. The resulting mixture was filtered through a short pad of silica gel (EtOAc eluent, 20 mL). All the volatiles were removed on a rotary evaporator. Flash column chromatography (pet ether: EtOAc = 15: 1) afforded 52.4 mg (67%) of compound **20a** as a yellow solid. Mp: 93-95 °C; ^{1}H NMR (400 MHz, $CDCl_3$) δ 9.08 (s, 1H), 7.51 (d, J = 1.2 Hz, 1H), 7.29 (d, J = 8.0 Hz, 1H), 7.19 (d, J = 8.8 Hz, 2H), 7.03 (d, J = 9.2 Hz, 2H), 6.64 (d, J = 8.8 Hz, 1H), 6.49 (d, J = 3.2 Hz, 1H), 6.44 (dd, J_1 = 2.8, J_2 = 1.6 Hz, 1H), 6.29 (d, J = 8.0 Hz, 1H), 5.05 (s, 2H), 3.92 (s, 3H) ppm; ^{13}C NMR (100 MHz, $CDCl_3$) δ 183.9 (q, J = 36.0 Hz), 162.0, 156.1, 150.9, 150.4, 143.4, 136.6, 133.2, 126.0, 117.0 (q, J = 287.5 Hz), 116.1, 110.8, 110.2, 107.4, 107.1, 99.5, 63.0, 55.9 ppm; IR (thin film) 3148, 3013, 2926, 1645, 1606, 1567, 1513, 1468, 1398, 1270, 1234, 1189, 1089, 999, 915, 868, 822, 795, 743, 658, 538 (cm^{-1}); HRMS (ESI) m/z: calcd for $[C_{20}H_{17}F_3NO_4]^+$ 392.1104; found 392.1103.

Reaction of 1b with compound sub-12:

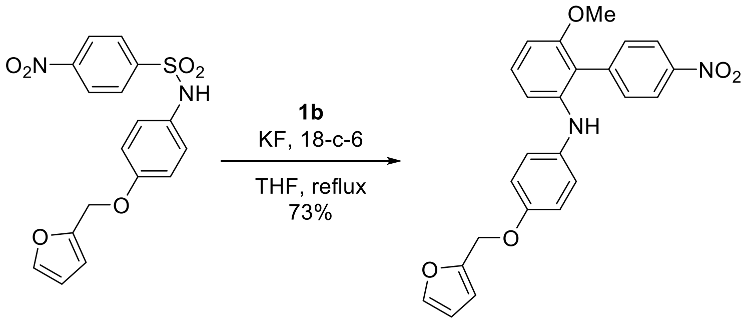

sub-**12** **21a**

A mixture of aryne precursor **1b** (98.5 mg, 0.3 mmol, 1.5 equiv), compound sub-**12** (74.9 mg, 0.2 mmol, 1.0 equiv), 18-crown-6 (105.7 mg, 0.4 mmol, 2.0 equiv), and KF (46.5 mg, 0.8 mmol, 4.0 equiv) in anhydrous THF (2.0 mL) under inert atmosphere was heated to reflux overnight. The resulting mixture was cooled to room temperature and filtered through a short pad of silica gel (EtOAc eluent, 20 mL). All the volatiles were removed on a rotary evaporator. Flash column chromatography (pet ether: EtOAc = 5: 1) afforded 60.8 mg (73%) of compound **21a** as a yellow solid. Mp: 163-165 °C; ^{1}H NMR (400 MHz, $CDCl_3$) δ 8.30 (d, J = 8.8 Hz, 2H), 7.56 (d, J = 8.8 Hz, 2H), 7.45 (dd, J_1 = 1.6, J_2 = 0.8 Hz, 1H), 7.20 (t, J = 8.0 Hz, 1H), 6.98 (d, J = 9.2 Hz, 2H), 6.90 (d, J = 8.8 Hz, 2H), 6.75 (d, J = 7.6 Hz, 1H), 6.51 (d, J = 8.4 Hz, 1H), 6.42 (d, J = 3.2 Hz, 1H), 6.38 (dd, J_1 = 3.2, J_2 = 1.6 Hz, 1H), 5.02 (brs, 1H), 4.96 (s, 2H), 3.72 (s, 3H) ppm; ^{13}C NMR (100 MHz, $CDCl_3$) δ 157.5, 154.7, 150.5, 147.3, 143.7, 143.3, 142.6, 135.9,

132.4, 130.0, 124.2, 123.4, 116.2, 116.1, 110.7, 110.1, 108.4, 102.5, 63.1, 55.9 ppm; IR (thin film) 3431, 3381, 2924, 2854, 1601, 1508, 1466, 1400, 1351, 1086, 999, 856, 736, 541, 427 (cm^{-1}); HRMS (ESI) m/z: calcd for $[C_{24}H_{21}N_2O_5]^+$ 417.1445; found 417.1453.

Reaction of 1b with compound sub-13:

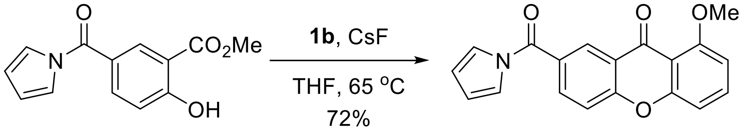

sub-**13** **22a**

A mixture of aryne precursor **1b** (98.5 mg, 0.3 mmol, 1.5 equiv), compound sub-**13** (49.0 mg, 0.2 mmol, 1.0 equiv), and CsF (91.1 mg, 0.6 mmol, 3.0 equiv) in anhydrous THF (2.0 mL) under inert atmosphere was stirred at 65 °C overnight. The resulting mixture was cooled to room temperature and filtered through a short pad of silica gel (EtOAc eluent, 20 mL). All the volatiles were removed on a rotary evaporator. Flash column chromatography (pet ether: EtOAc = 4: 1) afforded 46.0 mg (72%) of compound **22a** as a white solid. Mp: 210-212 °C; ^{1}H NMR (400 MHz, $CDCl_3$) δ 8.69 (d, J = 2.4 Hz, 1H), 8.11 (dd, J_1 = 8.4, J_2 = 2.0 Hz, 1H), 7.66 (t, J = 8.4 Hz, 1H), 7.56 (d, J = 8.4 Hz, 1H), 7.32-7.29 (m, 2H), 7.11 (dd, J_1 = 8.4, J_2 = 0.8 Hz, 1H), 6.87 (d, J = 8.4 Hz, 1H), 6.39-6.35 (m, 2H), 4.04 (s, 3H) ppm; ^{13}C NMR (100 MHz, $CDCl_3$) δ 175.7, 166.5, 161.1, 158.1, 157.4, 135.8, 135.4, 129.3, 128.9, 122.6, 121.5, 118.5, 113.7, 112.7, 110.3, 106.5, 56.8 ppm; IR (thin film) 3427, 3137, 2961, 2856, 2359, 1691, 1662, 1604, 1468, 1404, 1259, 1092, 996, 860, 802, 762, 539, 472 (cm^{-1}); HRMS (ESI) m/z: calcd for $[C_{19}H_{13}NNaO_4]^+$ 342.0737; found 342.0746.

Reaction of 1b with Furosemide methyl ester sub-14:

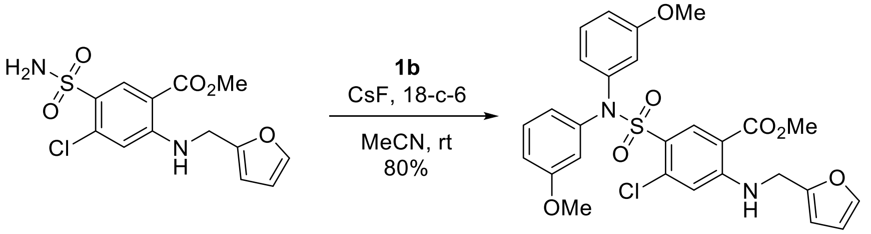

Furosemide
methyl ester sub-**14** **23a**

A mixture of aryne precursor **1b** (197.0 mg, 0.6 mmol, 3.0 equiv), Furosemide methyl ester sub-**14** (69.0 mg, 0.2 mmol, 1.0 equiv), 18-crown-6 (105.7 mg, 0.4 mmol, 2.0 equiv), and CsF (182.3 mg, 1.2 mmol, 6.0 equiv) in anhydrous MeCN (2.0 mL) under inert atmosphere was stirred at room temperature overnight. The resulting mixture was filtered through a short pad of silica gel (EtOAc eluent, 20 mL). All the volatiles were removed on a rotary evaporator. Flash column chromatography (pet ether: DCM = 1: 2) afforded 89.1 mg (80%) of compound **23a** as a colorless oil. ^{1}H NMR (400 MHz, $CDCl_3$) δ 8.63 (t, J = 5.6 Hz, 1H), 8.50 (s, 1H), 7.40 (dd, J_1 = 1.6, J_2 = 0.8 Hz, 1H), 7.18 (t, J = 8.0 Hz, 2H), 6.98 (dd, J_1 = 2.0, J_2 = 0.8 Hz, 1H), 6.97-6.95 (m, 3H), 6.85 (s, 1H), 6.78 (dd, J_1 = 2.4, J_2 = 0.8 Hz, 1H), 6.76 (dd, J_1 = 2.4, J_2 = 0.8 Hz, 1H), 6.36 (dd, J_1 = 3.2, J_2 = 1.6 Hz, 1H), 6.28 (d, J = 3.2 Hz, 1H), 4.44 (d, J = 5.6 Hz, 2H), 3.82 (s, 3H), 3.74 (s, 6H) ppm; ^{13}C NMR (100 MHz, $CDCl_3$) δ 167.8, 160.2, 153.3, 150.3, 142.9, 142.5, 138.9, 137.7, 129.9, 122.8, 121.0, 114.5, 113.9, 113.4, 110.7, 108.6, 108.1, 55.5, 52.3, 40.5 ppm; IR (thin film) 3418, 3334, 2956, 2837, 2424, 1693, 1595, 1487, 1442, 1408, 1329, 1260, 1175, 1099, 987, 859, 788, 694, 565, 541, 472 (cm^{-1}); HRMS (ESI) m/z: calcd for $[C_{27}H_{25}ClN_2NaO_7S]^+$ 579.0963; found 579.0973.

Reaction of 1b with compound 24:

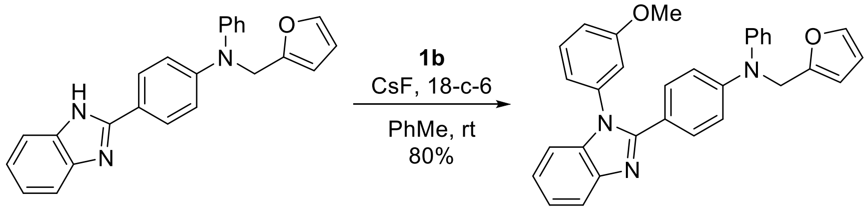

24 **25a**

A mixture of aryne precursor **1b** (98.5 mg, 0.3 mmol, 1.5 equiv), compound **24** (73.1 mg, 0.2 mmol, 1.0 equiv), 18-crown-6 (105.7 mg, 0.4 mmol, 2.0 equiv), and CsF (91.1 mg, 0.6 mmol, 3.0 equiv) in anhydrous toluene (2.0 mL) under inert atmosphere was stirred at room temperature overnight. The resulting mixture was filtered through a short pad of silica gel (EtOAc eluent, 20 mL). All the volatiles were removed on a rotary evaporator. Flash column chromatography (pet ether: EtOAc = 4: 1) afforded 75.4 mg (80%) of compound **25a** as a yellow oil. ^{1}H NMR (400 MHz, $CDCl_3$) δ 7.84 (d, J = 8.0 Hz, 1H), 7.45 (d, J = 8.8 Hz, 2H), 7.40 (t, J = 8.0 Hz, 1H), 7.35-7.29 (m, 4H), 7.23-7.18 (m, 4H), 7.12 (t, J = 7.6 Hz, 1H), 7.00 (dd, J_1 = 8.4, J_2 = 2.4 Hz, 1H), 6.92 (d, J = 7.6 Hz, 1H), 6.87 (t, J = 2.0 Hz, 1H), 6.83 (d, J = 9.2 Hz, 2H), 6.26 (dd, J_1 = 3.2, J_2 = 2.0 Hz, 1H), 6.13 (d, J = 2.8 Hz, 1H), 4.87 (s, 2H), 3.78 (s, 3H) ppm; ^{13}C NMR (100 MHz, $CDCl_3$) δ 160.9, 152.6, 151.7, 149.2, 146.8, 142.0, 138.5, 137.4, 130.8, 130.5, 129.8, 125.3, 124.7, 123.1, 120.0, 119.4, 116.6, 114.7, 113.3, 110.5, 110.4, 108.0, 55.7, 49.6 ppm; IR (thin film) 3851, 3440, 2923, 2851, 2347, 1592, 1532, 1493, 1384, 1326, 1262, 1109, 995, 853, 745, 697, 542, 475 (cm^{-1}); HRMS (ESI) m/z: calcd for $[C_{31}H_{26}N_3O_2]^+$ 472.2020; found 472.2023.

Reaction of 1b with compound sub-16:

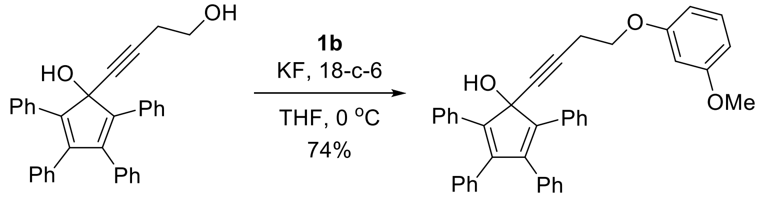

sub-**16** **26a**

A mixture of aryne precursor **1b** (98.5 mg, 0.3 mmol, 1.5 equiv), compound sub-**16** (90.9 mg, 0.2 mmol, 1.0 equiv), 18-crown-6 (105.7 mg, 0.4 mmol, 2.0 equiv), and KF (46.5 mg, 0.8 mmol, 4.0 equiv) in anhydrous THF (2.0 mL) under inert atmosphere was stirred at 0 °C overnight. The resulting mixture was filtered through a short pad of silica gel (EtOAc eluent, 20 mL). All the volatiles were removed on a rotary evaporator. Flash column chromatography (pet ether: EtOAc = 4: 1) afforded 83.0 mg (74%) of compound **26a** as a yellow solid. Mp: 46-48 °C; ^{1}H NMR (400 MHz, $CDCl_3$) δ 7.55-7.51 (m, 4H), 7.21-7.17 (m, 7H), 7.16-7.09 (m, 6H), 6.97-6.93 (m, 4H), 6.53 (dd, J_1 = 8.4, J_2 = 1.6 Hz, 1H), 6.45-6.40 (m, 2H), 3.88 (t, J = 7.2 Hz, 2H), 3.77 (s, 3H), 2.66 (t, J = 6.8 Hz, 2H), 2.52 (s, 1H) ppm; ^{13}C NMR (100 MHz, $CDCl_3$) δ 161.0, 159.9, 143.3, 142.6, 134.9, 134.0, 130.2, 130.1, 130.0, 128.2, 128.0, 127.5, 127.4, 106.9, 106.8, 101.4, 82.9, 81.6, 81.3, 66.0, 55.5, 20.3 ppm; IR (thin film) 3429, 3057, 1682, 1594, 1492, 1441, 1385, 1204, 1151, 1118, 993, 851, 806, 760, 711, 697, 588, 547, 492 (cm^{-1}); HRMS (ESI) m/z: calcd for $[C_{40}H_{32}NaO_3]^+$ 583.2244; found 583.2249.

Reaction of 1c with compound sub-1:

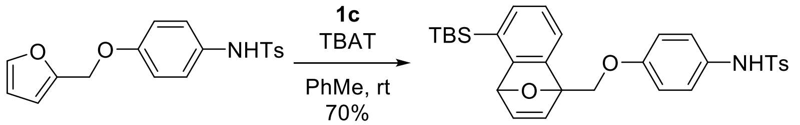

sub-**1** **6b**

A mixture of aryne precursor **1c** (123.8 mg, 0.3 mmol, 1.5 equiv), compound sub-**1** (68.7 mg, 0.2 mmol, 1.0 equiv), and TBAT (323.9 mg, 0.6 mmol, 3.0 equiv) in anhydrous toluene (4.0 mL) under inert atmosphere was stirred at room temperature overnight. The resulting mixture was filtered through a short pad of silica gel (EtOAc eluent, 20 mL). All the volatiles were removed on a rotary evaporator. Flash

column chromatography (pet ether: DCM = 1: 3) afforded 74.7 mg (70%) of compound **6b** as a white solid. Mp: 171-173 °C; ^{1}H NMR (400 MHz, $CDCl_3$) δ 7.59 (d, J = 8.0 Hz, 2H), 7.22 (d, J = 8.0 Hz, 3H), 7.10 (dd, J_1 = 7.6, J_2 = 0.8 Hz, 1H), 7.07 (dd, J_1 = 5.6, J_2 = 2.0 Hz, 1H), 7.00 (d, J = 8.8 Hz, 2H), 6.98-6.94 (m, 2H), 6.90 (d, J = 9.2 Hz, 2H), 6.51 (s, 1H), 5.85 (d, J = 1.6 Hz, 1H), 4.76 (d, J = 10.4 Hz, 1H), 4.64 (d, J = 10.4 Hz, 1H), 2.39 (s, 3H), 0.91 (s, 9H), 0.34 (s, 3H), 0.31 (s, 3H) ppm; ^{13}C NMR (100 MHz, $CDCl_3$) δ 157.3, 156.9, 147.1, 144.5, 143.9, 142.7, 136.2, 131.6, 130.0, 129.8, 127.5, 125.6, 124.0, 120.2, 115.6, 90.7, 83.6, 66.4, 26.7, 21.8, 17.4, -4.3, -4.7 ppm; IR (thin film) 3221, 2952, 2928, 2855, 1598, 1508, 1464, 1385, 1341, 1248, 1163, 1092, 1054, 990, 908, 831, 795, 769, 669, 589, 554 (cm^{-1}); HRMS (ESI) m/z: calcd for $[C_{30}H_{35}NNaO_4SSi]^+$ 556.1948; found 556.1957.
The structure of **6b** was confirmed by NOESY experiment, as the following interaction was observed:

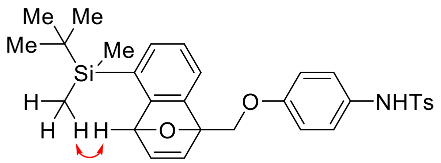

6b

Reaction of 1c with compound sub-2:

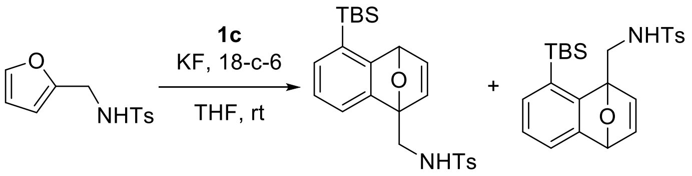

sub-**2** **7b**, 77% **7b'**, 10%

A mixture of aryne precursor **1c** (123.8 mg, 0.3 mmol, 1.5 equiv), compound sub-**2** (50.3 mg, 0.2 mmol, 1.0 equiv), 18-crown-6 (105.7 mg, 0.4 mmol, 2.0 equiv), and KF (46.5 mg, 0.8 mmol, 4.0 equiv) in anhydrous THF (2.0 mL) under inert atmosphere was stirred at room temperature overnight. The resulting mixture was filtered through a short pad of silica gel (EtOAc eluent, 20 mL). All the volatiles were removed on a rotary evaporator. Flash column chromatography (pet ether: EtOAc = 6: 1) afforded 68.0 mg (77%) of compound **7b** as a white solid and 8.8 mg (10%) of compound **7b'** as a white solid.
Compound 7b: Mp: 148-150 °C; ^{1}H NMR (400 MHz, $CDCl_3$) δ 7.79 (d, J = 8.0 Hz, 2H), 7.31 (d, J = 8.0 Hz, 2H), 7.10 (d, J = 7.2 Hz, 1H), 7.06 (d, J = 7.6 Hz, 1H), 6.99 (d, J = 5.2 Hz, 1H), 6.94 (t, J = 7.6 Hz, 1H), 6.74 (d, J = 5.2 Hz, 1H), 5.74 (d, J = 1.2 Hz, 1H), 4.98 (dd, J_1 = 6.4, J_2 = 5.2 Hz, 1H), 3.93 (dd, J_1 = 13.2, J_2 = 7.2 Hz, 1H), 3.73 (dd, J_1 = 13.2, J_2 = 4.4 Hz, 1H), 2.42 (s, 3H), 0.88 (s, 9H), 0.31 (s, 3H), 0.28 (s, 3H) ppm; ^{13}C NMR (100 MHz, $CDCl_3$) δ 156.9, 146.2, 145.0, 143.8, 142.5, 136.7, 131.5, 130.0, 129.9, 127.4, 124.1, 120.0, 90.9, 83.4, 42.3, 26.6, 21.7, 17.3, -4.3, -4.8 ppm; IR (thin film) 3268, 3046, 2953, 2927, 2855, 1598, 1494, 1462, 1332, 1251, 1163, 1091, 995, 831, 770, 666, 562 (cm^{-1}); HRMS (ESI) m/z: calcd for $[C_{24}H_{31}NNaO_3SSi]^+$ 464.1686; found 464.1694.
Compound 7b': Mp: 98-100 °C; ^{1}H NMR (400 MHz, $CDCl_3$) δ 7.78 (d, J = 8.0 Hz, 2H), 7.30 (d, J = 8.0 Hz, 2H), 7.19 (d, J = 7.2 Hz, 1H), 7.13 (d, J = 7.6 Hz, 1H), 6.98 (d, J = 5.2 Hz, 1H), 6.92 (t, J = 7.2 Hz, 1H), 6.79 (d, J = 5.6 Hz, 1H), 5.58 (d, J = 1.6 Hz, 1H), 4.85 (d, J = 10.0 Hz, 1H), 4.34 (dd, J_1 = 12.4, J_2 = 10.0 Hz, 1H), 3.43 (d, J = 12.4 Hz, 1H), 2.41 (s, 3H), 0.87 (s, 9H), 0.36 (s, 3H), 0.32 (s, 3H) ppm; ^{13}C NMR (100 MHz, $CDCl_3$) δ 154.5, 149.9, 145.1, 143.8, 142.4, 136.6, 133.4, 130.1, 130.0, 127.5, 124.1, 120.9, 93.6, 81.6, 45.0, 27.2, 21.7, 17.8, -1.2, -2.1 ppm; IR (thin film) 3267, 3056, 2954, 2928, 2856, 1598, 1494, 1463, 1333, 1255, 1163, 1089, 988, 834, 814, 771, 665, 550 (cm^{-1}); HRMS (ESI) m/z: calcd for $[C_{24}H_{31}NNaO_3SSi]^+$ 464.1686; found 464.1688.
The structures of **7b** and **7b'** were confirmed by NOESY experiment, as the following interactions were observed:

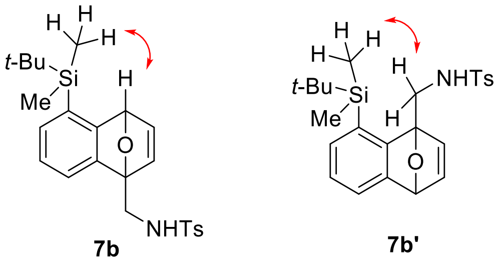

7b **7b'**

Reaction of 1c with compound sub-3:

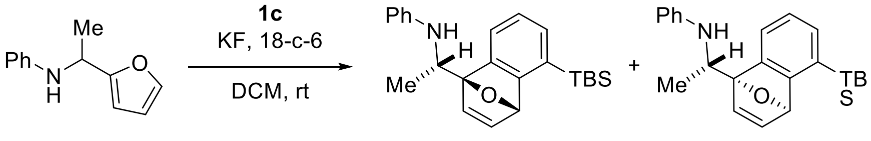

sub-**3** **8b**, 44% **8b'**, 43%

A mixture of aryne precursor **1c** (123.8 mg, 0.3 mmol, 1.5 equiv), compound sub-**3** (37.4 mg, 0.2 mmol, 1.0 equiv), 18-crown-6 (105.7 mg, 0.4 mmol, 2.0 equiv), and KF (46.5 mg, 0.8 mmol, 4.0 equiv) in anhydrous DCM (2.0 mL) under inert atmosphere was stirred at room temperature overnight. The resulting mixture was filtered through a short pad of silica gel (EtOAc eluent, 20 mL). All the volatiles were removed on a rotary evaporator. Flash column chromatography (pet ether: EtOAc = 50: 1) afforded 33.2 mg (44%) of compound **8b** as a colorless oil and 32.5 mg (43%) of compound **8b'** as a colorless oil.
Compound 8b: ^{1}H NMR (400 MHz, $CDCl_3$) δ 7.25-7.19 (m, 3H), 7.09 (d, J = 7.6 Hz, 1H), 7.01-6.96 (m, 2H), 6.88 (d, J = 5.6 Hz, 1H), 6.75-6.70 (m, 3H), 5.83 (d, J = 0.8 Hz, 1H), 4.53 (q, J = 5.6 Hz, 1H), 4.02 (brs, 1H), 1.40 (d, J = 6.4 Hz, 3H), 0.92 (s, 9H), 0.35 (s, 3H), 0.31 (s, 3H) ppm; ^{13}C NMR (100 MHz, $CDCl_3$) δ 158.1, 147.4, 147.3, 144.1, 144.1, 131.3, 129.6, 129.5, 123.9, 120.7, 117.7, 113.6, 95.9, 83.1, 47.8, 26.7, 17.4, 16.6, -4.2, -4.7 ppm; IR (thin film) 3405, 3051, 2952, 2927, 2884, 2855, 1602, 1507, 1470, 1250, 1003, 828, 768, 748, 720, 690 (cm^{-1}); HRMS (ESI) m/z: calcd for $[C_{24}H_{32}NOSi]^+$ 378.2248; found 378.2259.
Compound 8b': ^{1}H NMR (400 MHz, $CDCl_3$) δ 7.19 (t, J = 7.6 Hz, 2H), 7.12 (d, J = 7.2 Hz, 1H), 7.07-7.03 (m, 2H), 6.89 (t, J = 7.6 Hz, 1H), 6.81 (d, J = 5.2 Hz, 1H), 6.70 (t, J = 7.2 Hz, 1H), 6.66 (d, J = 8.0 Hz, 2H), 5.86 (s, 1H), 4.50 (q, J = 5.6 Hz, 1H), 4.00 (brs, 1H), 1.46 (d, J = 6.4 Hz, 3H), 0.92 (s, 9H), 0.34 (s, 3H), 0.31 (s, 3H) ppm; ^{13}C NMR (100 MHz, $CDCl_3$) δ 157.7, 147.4, 147.1, 144.9, 143.1, 131.2, 129.5, 129.4, 124.0, 121.1, 117.3, 113.2, 95.7, 83.4, 47.0, 26.7, 17.4, 16.5, -4.2, -4.7 ppm; IR (thin film) 3411, 3049, 2953, 2927, 2855, 1602, 1507, 1470, 1250, 1044, 906, 829, 768, 747, 720, 675 (cm^{-1}); HRMS (ESI) m/z: calcd for $[C_{24}H_{32}NOSi]^+$ 378.2248; found 378.2246.
The structures of **8b** and **8b'** were confirmed by NOESY experiment, as the following interactions were observed:

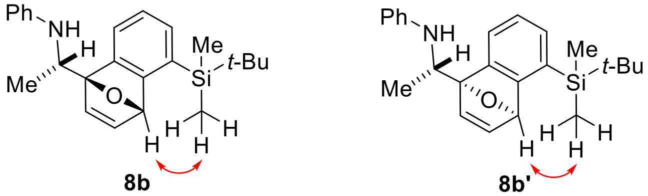

8b **8b'**

Reaction of 1c with compound sub-4:

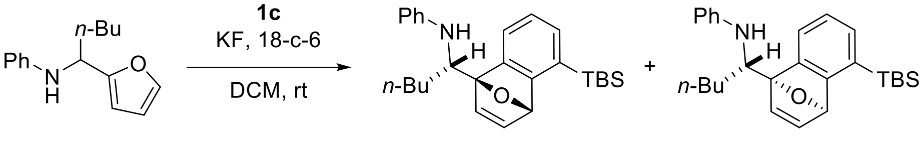

sub-**4** **9b**, 48% **9b'**, 37%

A mixture of aryne precursor **1c** (123.8 mg, 0.3 mmol, 1.5 equiv), compound sub-**4** (45.9 mg, 0.2 mmol, 1.0 equiv), 18-crown-6 (105.7 mg, 0.4 mmol, 2.0 equiv), and KF (46.5 mg, 0.8 mmol, 4.0 equiv) in DCM (2.0 mL) under inert atmosphere was stirred at room temperature overnight. The resulting mixture was filtered through a short pad of silica gel (EtOAc eluent, 20 mL). All the volatiles were removed on a rotary evaporator. Flash column chromatography (pet ether: EtOAc = 50: 1) afforded 40.3 mg (48%) of compound **9b** as a colorless oil and 31.1 mg (37%) of compound **9b'** as a colorless oil.

Compound 9b: ^{1}H NMR (400 MHz, $CDCl_3$) δ 7.22-7.17 (m, 3H), 7.06 (d, *J* = 6.8 Hz, 1H), 6.97-6.92 (m, 2H), 6.79 (d, *J* = 5.6 Hz, 1H), 6.74-6.67 (m, 3H), 5.79 (d, *J* = 1.2 Hz, 1H), 4.37 (dd, J_1 = 9.2, J_2 = 2.0 Hz, 1H), 3.91 (brs, 1H), 1.93-1.85 (m, 1H), 1.65-1.60 (m, 1H), 1.45-1.25 (m, 4H), 0.90 (s, 9H), 0.86 (t, *J* = 7.2 Hz, 3H), 0.33 (s, 3H), 0.29 (s, 3H) ppm; ^{13}C NMR (100 MHz, $CDCl_3$) δ 158.0, 148.8, 147.8, 144.1, 131.3, 129.7, 129.4, 123.9, 120.6, 117.4, 113.1, 96.4, 82.9, 52.4, 32.7, 29.0, 26.7, 23.0, 17.4, 14.3, -4.2, -4.7 ppm; IR (thin film) 3373, 3052, 2953, 2928, 2856, 1601, 1507, 1470, 1250, 1124, 830, 768, 746, 691, 674 (cm^{-1}); HRMS (ESI) m/z: calcd for $[C_{27}H_{38}NOSi]^+$ 420.2717; found 420.2726.

Compound 9b': ^{1}H NMR (400 MHz, $CDCl_3$) δ 7.19-7.14 (m, 2H), 7.03-7.00 (m, 3H), 6.85-6.80 (m, 2H), 6.69-6.63 (m, 3H), 5.82 (d, *J* = 1.6 Hz, 1H), 4.33 (dd, J_1 = 8.4, J_2 = 4.0 Hz, 1H), 3.96 (brs, 1H), 1.99-1.90 (m, 1H), 1.79-1.70 (m, 1H), 1.65-1.58 (m, 1H), 1.49-1.43 (m, 1H), 1.38-1.29 (m, 2H), 0.91 (s, 9H), 0.89 (t, *J* = 7.2 Hz, 3H), 0.33 (s, 3H), 0.29 (s, 3H) ppm; ^{13}C NMR (100 MHz, $CDCl_3$) δ 157.7, 148.6, 147.5, 144.5, 143.5, 131.1, 129.5, 129.3, 123.8, 121.0, 117.0, 112.9, 96.3, 83.2, 52.0, 32.9, 29.0, 26.7, 23.3, 17.4, 14.3, -4.2, -4.7 ppm; IR (thin film) 3415, 3052, 2954, 2927, 2856, 1601, 1507, 1470, 1250, 1125, 830, 768, 746, 690, 675 (cm^{-1}); HRMS (ESI) m/z: calcd for $[C_{27}H_{38}NOSi]^+$ 420.2717; found 420.2728.

The structures of **9b** and **9b'** were confirmed by NOESY experiment, as the following interactions were observed:

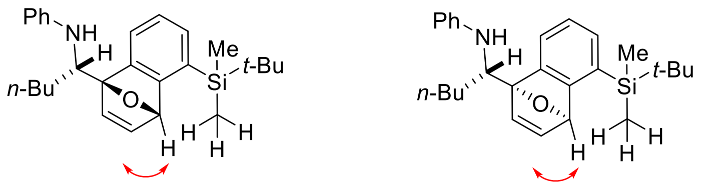

9b **9b'**

Reaction of 1c with compound sub-5:

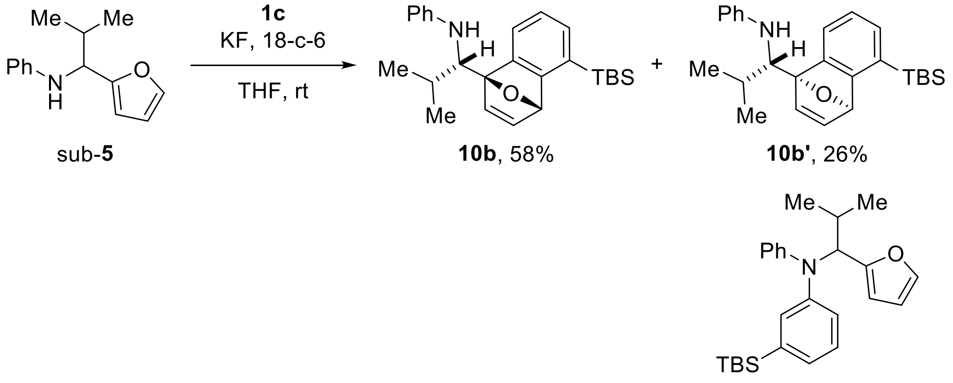

S79, <5%

A mixture of aryne precursor **1c** (123.8 mg, 0.3 mmol, 1.5 equiv), compound sub-**5** (43.1 mg, 0.2 mmol, 1.0 equiv), 18-crown-6 (105.7 mg, 0.4 mmol, 2.0 equiv), and KF (46.5 mg, 0.8 mmol, 4.0 equiv) in anhydrous THF (2.0 mL) under inert atmosphere was stirred at room temperature overnight. The resulting mixture was filtered through a short pad of silica gel (EtOAc eluent, 20 mL). All the volatiles were removed on a rotary evaporator. Flash column chromatography (pet ether: EtOAc = 50: 1) afforded 47.1 mg (58%) of compound **10b** as a white solid, 21.1 mg (26%) of compound **10b'** as a colorless oil and 4.1 mg (<5%) of compound **S79** as a colorless oil.

Compound 10b: Mp: 126-128 °C; ^{1}H NMR (400 MHz, $CDCl_3$) δ 7.22-7.17 (m, 3H), 7.05 (dd, J_1 = 8.0, J_2 = 1.2 Hz, 1H), 6.93 (t, *J* = 7.2 Hz, 1H), 6.90 (dd, J_1 = 5.6, J_2 = 2.0 Hz, 1H), 6.79-6.75 (m, 3H), 6.70 (t,

J = 7.6 Hz, 1H), 5.79 (d, J = 2.0 Hz, 1H), 4.37 (d, J = 2.8 Hz, 1H), 2.39-2.32 (m, 1H), 1.07 (d, J = 6.8 Hz, 3H), 1.01 (d, J = 6.8 Hz, 3H), 0.90 (s, 9H), 0.33 (s, 3H), 0.29 (s, 3H) ppm; ^{13}C NMR (100 MHz, $CDCl_3$) δ 157.8, 149.2, 148.6, 144.8, 143.4, 131.2, 129.7, 129.4, 123.9, 120.8, 117.4, 113.3, 96.3, 83.0, 56.7, 30.7, 26.7, 22.7, 17.4, -4.2, -4.7 ppm; IR (thin film) 3422, 3054, 2956, 2927, 2855, 1602, 1508, 1470, 1385, 1249, 1102, 1008, 871, 834, 797, 769, 691, 543, 475 (cm^{-1}); HRMS (ESI) m/z: calcd for $[C_{26}H_{36}NOSi]^+$ 406.2561; found 406.2567. The X-ray crystal structure of compound **10b** was determined and its structural information was shown in Supplementary Fig 2 and Supplementary Tables 3-5.

Compound 10b': ^{1}H NMR (400 MHz, $CDCl_3$) δ 7.15 (t, J = 7.6 Hz, 2H), 7.01-6.96 (m, 2H), 6.90 (d, J = 7.2 Hz, 1H), 6.82 (d, J = 5.6 Hz, 1H), 6.76 (t, J = 7.2 Hz, 1H), 6.66-6.62 (m, 3H), 5.82 (d, J = 1.6 Hz, 1H), 4.33 (d, J = 3.2 Hz, 1H), 4.07 (brs, 1H), 2.36-2.28 (m, 1H), 1.14 (d, J = 7.2 Hz, 3H), 1.10 (d, J = 6.8 Hz, 3H), 0.90 (s, 9H), 0.32 (s, 3H), 0.29 (s, 3H) ppm; ^{13}C NMR (100 MHz, $CDCl_3$) δ 157.3, 149.3, 148.3, 144.1, 143.7, 131.0, 129.4, 129.1, 123.8, 120.9, 116.7, 112.9, 96.9, 83.2, 55.6, 32.1, 26.8, 22.4, 18.3, 17.4, -4.2, -4.6 ppm; IR (thin film) 3423, 3053, 2955, 2856, 2369, 1600, 1508, 1466, 1388, 1251, 1125, 968, 834, 770, 746, 713, 694, 543, 473 (cm^{-1}); HRMS (ESI) m/z: calcd for $[C_{26}H_{36}NOSi]^+$ 406.2561; found 406.2568.

Compound S79: ^{1}H NMR (400 MHz, $CDCl_3$) δ 7.28-7.26 (m, 1H), 7.24-7.14 (m, 4H), 7.08 (d, J = 2.4 Hz, 1H), 6.92-6.84 (m, 4H), 6.22 (dd, J_1 = 3.2, J_2 = 1.6 Hz, 1H), 6.02 (d, J = 3.2 Hz, 1H), 4.79 (d, J = 10.4 Hz, 1H), 2.48-2.36 (m, 1H), 1.20 (d, J = 6.4 Hz, 3H), 0.92 (d, J = 6.8 Hz, 3H), 0.83 (s, 9H), 0.20 (s, 3H), 0.19 (s, 3H) ppm; ^{13}C NMR (100 MHz, $CDCl_3$) δ 154.5, 148.8, 146.0, 141.1, 138.7, 131.0, 129.1, 129.0, 128.3, 125.5, 121.0, 120.6, 110.1, 108.4, 64.2, 30.6, 26.7, 21.1, 21.0, 17.1, -6.0, -6.1 ppm; IR (thin film) 3429, 2955, 2927, 2855, 1597, 1497, 1470, 1385, 1256, 1114, 1012, 833, 808, 769, 733, 698, 542, 474 (cm^{-1}); HRMS (ESI) m/z: calcd for $[C_{26}H_{36}NOSi]^+$ 406.2561; found 406.2558.

The structures of **10b'** was confirmed by NOESY experiment, as the following interaction was observed:

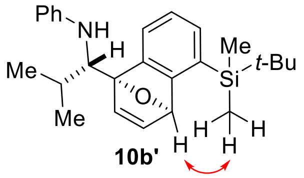

Conversion of 10b to compound 18:

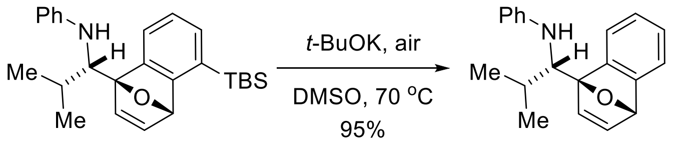

To a solution of **10b** (40.6 mg, 0.1 mmol, 1.0 equiv) in DMSO (0.5 mL) under air at room temperature was added *t*-BuOK (16.8 mg, 0.15 mmol, 1.5 equiv). The resulting mixture was heated to 70 °C overnight. After cooling back to room temperature, the resulting mixture was quenched with water (10 mL). The separated aqueous layer was extracted with EtOAc (10 mL x 3), and the combined organic layers were washed with water (100 mL x 2), brine (20 mL), dried over Na_2SO_4, filtered, and concentrated. Flash column chromatography (pet ether: EtOAc = 50: 1) afforded 27.7 mg (95%) of compound **18** as a colorless oil.

Reaction of 1c with Fuberidazole:

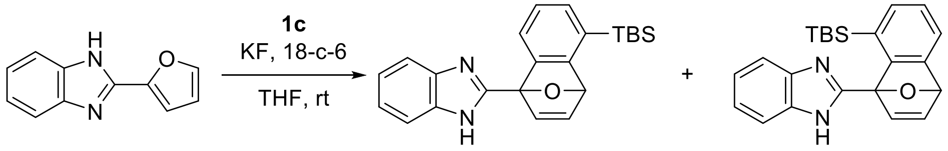

A mixture of aryne precursor **1c** (123.8 mg, 0.3 mmol, 1.5 equiv), commercially available Fuberidazole (36.8 mg, 0.2 mmol, 1.0 equiv), 18-crown-6 (105.7 mg, 0.4 mmol, 2.0 equiv), and KF (46.5 mg, 0.8 mmol, 4.0 equiv) in anhydrous THF (2.0 mL) under inert atmosphere was stirred at room temperature overnight. The resulting mixture was filtered through a short pad of silica gel (EtOAc eluent, 20 mL). All the volatiles were removed on a rotary evaporator. Flash column chromatography (pet ether: EtOAc = 8: 1) afforded 33.7 mg (45%) of compound **11b** as a white solid and 30.7 mg (41%) of compound **11b'** as a white solid.
Compound 11b: Mp: 76-78 °C; ^{1}H NMR (400 MHz, $CDCl_3$) δ 9.55 (brs, 1H), 7.89 (brs, 1H), 7.51-7.36 (m, 2H), 7.33-7.29 (m, 3H), 7.13-7.10 (m, 2H), 6.96 (t, J = 7.2 Hz, 1H), 5.99 (d, J = 1.6 Hz, 1H), 0.94 (s, 9H), 0.36 (s, 3H), 0.35 (s, 3H) ppm; ^{13}C NMR (100 MHz, $CDCl_3$) δ 155.0, 149.3, 147.9, 143.7, 143.3, 133.6, 131.8, 130.0, 124.4, 123.1, 121.3, 120.0, 111.3, 88.9, 84.2, 26.6, 17.4, -4.2, -4.8 ppm; IR (thin film) 3421, 2922, 1636, 1384, 1271, 1249, 1099, 984, 862, 829, 798, 745, 704, 544, 475 (cm^{-1}); HRMS (ESI) m/z: calcd for $[C_{23}H_{27}N_2OSi]^+$ 375.1887; found 375.1891.
Compound 11b': Mp: 93-95 °C; ^{1}H NMR (400 MHz, $CDCl_3$) δ 9.32 (brs, 1H), 7.88 (d, J = 6.0 Hz, 1H), 7.70 (d, J = 5.6 Hz, 1H), 7.40-7.35 (m, 1H), 7.32-7.29 (m, 3H), 7.23 (dd, J_1 = 7.6, J_2 = 1.2 Hz, 1H), 7.12 (dd, J_1 = 5.6, J_2 = 2.0 Hz, 1H), 7.03 (t, J = 7.2 Hz, 1H), 5.77 (d, J = 2.0 Hz, 1H), 0.74 (s, 9H), -0.06 (s, 3H), -0.49 (s, 3H) ppm; ^{13}C NMR (100 MHz, $CDCl_3$) δ 156.3, 149.6, 148.9, 143.9, 143.0, 142.1, 134.2, 133.5, 131.9, 124.3, 124.1, 122.7, 120.9, 120.5, 111.1, 91.7, 82.4, 27.1, 17.7, -3.9, -4.8 ppm; IR (thin film) 3465, 2955, 2851, 2349, 2284, 1634, 1403, 1384, 1280, 1088, 983, 928, 864, 786, 742, 530, 475 (cm^{-1}); HRMS (ESI) m/z: calcd for $[C_{23}H_{27}N_2OSi]^+$ 375.1887; found 375.1889.
The structure of **11b** was confirmed by NOESY experiment, as the following interaction was observed:

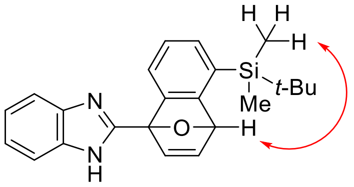

11b

Conversion of 11b to compound 19:

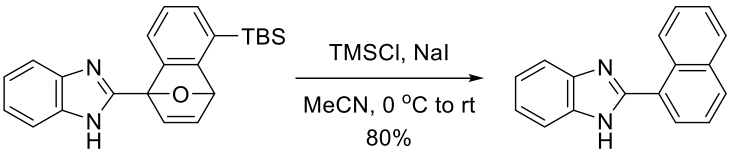

11b **19**

To a suspension of **11b** (30 mg, 0.08 mmol, 1.0 equiv) and NaI (60 mg, 0.4 mmol, 5.0 equiv) in MeCN (1.0 mL) at 0 °C was slowly added chlorotrimethylsilane (TMSCl) (51 uL, 0.4 mmol, 5.0 equiv). After 10 minutes, the resulting mixture was allowed to warm to room temperature. Upon completion of the reaction, the resulting solution was then quenched with 10% aq $Na_2S_2O_3$ (2 mL). The separated aqueous layer was extracted with EtOAc (10 mL x 3), and the combined organic layers were washed with brine (10 mL), dried over Na_2SO_4, filtered, and concentrated. Flash column chromatography (pet ether: acetone = 5: 1) afforded 15.6 mg (80%) of compound **19** as a white solid, the ^{1}H NMR of which is identical with that reported in literature.[32] ^{1}H NMR (400 MHz, DMSO-$d6$) δ 12.92 (s, 1H), 9.11 (d, J = 8.4 Hz, 1H), 8.10 (d, J = 8.4 Hz, 1H), 8.07-8.00 (m, 2H), 7.78 (d, J = 7.6 Hz, 1H), 7.71-7.56 (m, 4H), 7.30-7.23 (m, 2H) ppm.

Conversion of 11b' to compound 19:

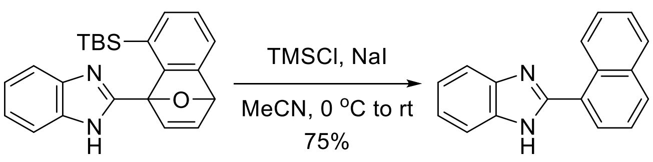

11b' 19

To a suspension of **11b'** (30 mg, 0.08 mmol, 1.0 equiv) and NaI (60 mg, 0.4 mmol, 5.0 equiv) in MeCN (1.0 mL) at 0 °C was slowly added TMSCl (51 uL, 0.4 mmol, 5.0 equiv). After 10 minutes, the resulting mixture was allowed to warm to room temperature. Upon completion of the reaction, the resulting solution was then quenched with 10% aq $Na_2S_2O_3$ (2 mL). The separated aqueous layer was extracted with EtOAc (10 mL x 3), and the combined organic layers were washed with brine (10 mL), dried over Na_2SO_4, filtered, and concentrated. Flash column chromatography (pet ether: acetone = 5: 1) afforded 14.7 mg (75%) of compound **19** as a white solid.

Reaction of 1c with compound sub-6:

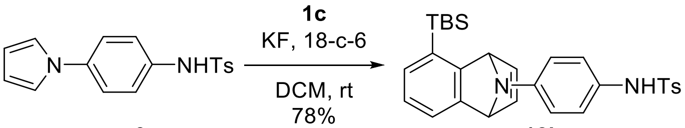

sub-**6** **12b**

A mixture of aryne precursor **1c** (123.8 mg, 0.3 mmol, 1.5 equiv), compound sub-**6** (62.5 mg, 0.2 mmol, 1.0 equiv), 18-crown-6 (105.7 mg, 0.4 mmol, 2.0 equiv), and KF (46.5 mg, 0.8 mmol, 4.0 equiv) in anhydrous DCM (2.0 mL) under inert atmosphere was stirred at room temperature overnight. The resulting mixture was filtered through a short pad of silica gel (EtOAc eluent, 20 mL). All the volatiles were removed on a rotary evaporator. Flash column chromatography (pet ether: DCM = 1: 1) afforded 78.4 mg (78%) of compound **12b** as a colorless oil. ^{1}H NMR (400 MHz, $CDCl_3$) δ 7.49 (d, J = 8.0 Hz, 2H), 7.27 (d, J = 6.4 Hz, 1H), 7.14 (d, J = 8.0 Hz, 2H), 7.05 (dd, J_1 = 7.6, J_2 = 0.8 Hz, 1H), 6.94-6.89 (m, 2H), 6.86-6.82 (m, 3H), 6.67 (d, J = 8.8 Hz, 2H), 6.36 (s, 1H), 5.49 (s, 1H), 5.31 (s, 1H), 2.36 (s, 3H), 0.89 (s, 9H), 0.34 (s, 3H), 0.33 (s, 3H) ppm; ^{13}C NMR (100 MHz, $CDCl_3$) δ 155.0, 147.4, 145.4, 143.7, 142.1, 141.6, 136.2, 131.5, 130.6, 129.6, 129.3, 127.5, 124.8, 123.9, 122.2, 118.8, 71.4, 68.5, 26.8, 21.7, 17.4, -4.1, -4.4 ppm; IR (thin film) 3258, 3045, 2926, 2855, 1598, 1508, 1462, 1334, 1161, 1092, 930, 825, 769, 736, 670, 574, 545 (cm^{-1}); HRMS (ESI) m/z: calcd for $[C_{29}H_{35}N_2O_2SSi]^+$ 503.2183; found 503.2184.

Reaction of 1c with compound sub-7:

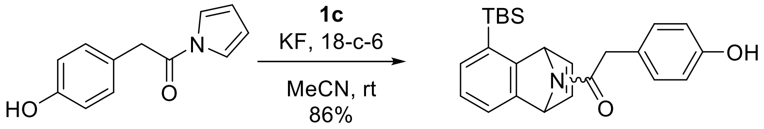

sub-**7** **13b**

A mixture of aryne precursor **1c** (123.8 mg, 0.3 mmol, 1.5 equiv), compound sub-**7** (40.2 mg, 0.2 mmol, 1.0 equiv), 18-crown-6 (105.7 mg, 0.4 mmol, 2.0 equiv), and KF (46.5 mg, 0.8 mmol, 4.0 equiv) in anhydrous MeCN (2.0 mL) under inert atmosphere was stirred at room temperature overnight. The resulting mixture was filtered through a short pad of silica gel (EtOAc eluent, 20 mL). All the volatiles were removed on a rotary evaporator. Flash column chromatography (pet ether: EtOAc = 4: 1) afforded 67.4 mg (86%) of compound **13b** as a 1: 1 mixture of conformational isomers. ^{1}H NMR (400 MHz, $CDCl_3$) δ 7.28 (d, J = 6.8 Hz, 1H), 7.16 (d, J = 7.2 Hz, 1H), 7.09 (d, J = 4.4 Hz, 1H), 7.07 (d, J = 4.4 Hz, 1H), 7.02 (dd, J_1 = 5.6, J_2 = 2.4 Hz, 1H), 7.01-6.95 (m, 2H), 6.95-6.88 (m, 5H), 6.86 (dd, J_1 = 5.2, J_2 = 2.0 Hz, 1H), 6.79 (dd, J_1 = 5.6, J_2 = 2.4 Hz, 1H), 6.64 (d, J = 8.8 Hz, 2H), 6.60 (d, J = 8.4 Hz, 2H), 6.08 (s, 1H), 5.94 (s, 1H), 5.74 (s, 1H), 5.56 (s, 1H), 3.55-3.45 (m, 4H), 0.93 (s, 9H), 0.91 (s, 9H), 0.37 (s, 3H), 0.35 (s, 3H), 0.33 (s, 6H) ppm; ^{13}C NMR (100 MHz, $CDCl_3$) δ 166.5, 166.1, 155.7, 155.6, 154.6, 154.4, 146.8, 146.7, 144.7, 144.1, 142.6, 142.5, 131.7, 131.6, 131.2, 130.1, 129.8, 125.1, 125.0, 124.4, 124.0, 122.2,

121.1, 116.1, 116.0, 66.9, 65.7, 64.6, 63.2, 40.2, 40.1, 26.8, 26.7, 17.3, 17.2, -4.2, -4.3, -4.5, -4.6 ppm; IR (thin film) 3368, 2952, 2925, 2854, 2341, 1645, 1612, 1594, 1518, 1417, 1390, 1359, 1252, 1231, 1216, 1097, 911, 859, 796, 660, 529, 477 (cm^{-1}); HRMS (ESI) m/z: calcd for $[C_{24}H_{30}NO_2Si]^+$ 392.2040; found 392.2047.

Reaction of 1c with compound sub-8:

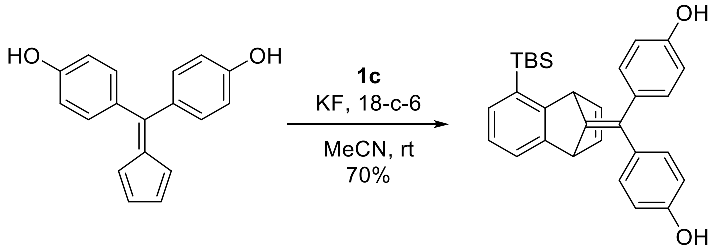

A mixture of aryne precursor **1c** (123.8 mg, 0.3 mmol, 1.5 equiv), compound sub-**8** (52.5 mg, 0.2 mmol, 1.0 equiv), 18-crown-6 (105.7 mg, 0.4 mmol, 2.0 equiv), and KF (46.5 mg, 0.8 mmol, 4.0 equiv) in anhydrous MeCN (2.0 mL) under inert atmosphere was stirred at room temperature overnight. The resulting mixture was filtered through a short pad of silica gel (EtOAc eluent, 20 mL). All the volatiles were removed on a rotary evaporator. Flash column chromatography (pet ether: EtOAc = 5: 1) afforded 63.4 mg (70%) of compound **14b** as a white solid. Mp: 240-242 °C; ^1H NMR (400 MHz, DMSO-*d*6) δ 9.40 (s, 1H), 9.35 (s, 1H), 7.35 (d, J = 6.8 Hz, 1H), 7.03-6.93 (m, 4H), 6.75 (t, J = 8.4 Hz, 4H), 6.69 (dd, J_1 = 8.4, J_2 = 6.8 Hz, 4H), 4.47 (s, 1H), 4.37 (s, 1H), 0.67 (s, 9H), 0.24 (s, 3H), 0.15 (s, 3H) ppm; ^{13}C NMR (100 MHz, DMSO-*d*6) δ 162.1, 156.6, 156.1, 156.0, 148.3, 143.7, 142.0, 130.8, 130.6, 130.5, 130.3, 130.2, 128.9, 123.6, 121.9, 114.8, 114.7, 113.7, 53.1, 51.3, 26.3, 16.6, -4.5, -4.6 ppm; IR (thin film) 3515, 3411, 3035, 2952, 2924, 2852, 1664, 1607, 1508, 1391, 1267, 1167, 1125, 912, 823, 810, 770, 751, 608, 548, 478 (cm^{-1}); HRMS (ESI) m/z: calcd for $[C_{30}H_{33}O_2Si]^+$ 453.2244; found 453.2255.

Reaction of 1c with compound sub-9:

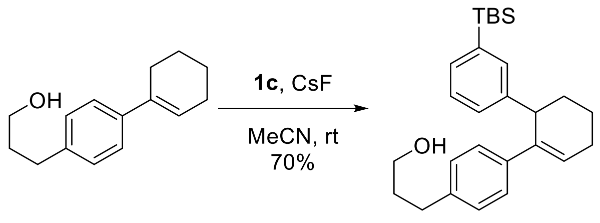

A mixture of aryne precursor **1c** (123.8 mg, 0.3 mmol, 1.5 equiv), compound sub-**9** (43.3 mg, 0.2 mmol, 1.0 equiv), and CsF (91.1 mg, 0.6 mmol, 3.0 equiv) in anhydrous MeCN (2.0 mL) under inert atmosphere was stirred at room temperature overnight. The resulting mixture was filtered through a short pad of silica gel (EtOAc eluent, 20 mL). All the volatiles were removed on a rotary evaporator. Flash column chromatography (pet ether: EtOAc = 10: 1) afford 56.9 mg (70%) of compound **15b** as a colorless oil. ^1H NMR (400 MHz, $CDCl_3$) δ 7.32 (s, 1H), 7.27-7.24 (m, 1H), 7.24-7.13 (m, 4H), 6.98 (d, J = 8.0 Hz, 2H), 6.35 (t, J = 4.0 Hz, 1H), 4.01 (brs, 1H), 3.61 (t, J = 6.4 Hz, 2H), 2.59 (t, J = 7.6 Hz, 2H), 2.37-2.26 (m, 2H), 2.13-2.05 (m, 1H), 1.90-1.77 (m, 3H), 1.61-1.54 (m, 2H), 1.33 (brs, 1H), 0.79 (s, 9H), 0.23 (s, 3H), 0.22 (s, 3H) ppm; ^{13}C NMR (100 MHz, $CDCl_3$) δ 144.0, 139.9, 138.1, 137.3, 135.0, 131.9, 129.3, 128.2, 127.5, 127.4, 127.3, 126.1, 62.5, 42.9, 34.3, 33.0, 31.7, 26.7, 26.3, 17.9, 17.1, -6.1 ppm; IR (thin film) 3386, 3021, 2928, 2854, 1892, 1637, 1513, 1462, 1403, 1360, 1247, 1148, 1117, 1072, 1007, 937, 831, 803, 768, 710, 668, 551, 462 (cm^{-1}); HRMS (ESI) m/z: calcd for $[C_{27}H_{38}NaOSi]^+$ 429.2584; found 429.2586.

Reaction of 1c with *N*-deacetyl *N*-tosyl Colchicine sub-10:

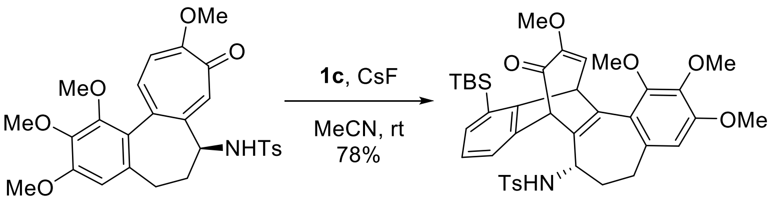

sub-**10** **16b**

A mixture of aryne precursor **1c** (123.8 mg, 0.3 mmol, 1.5 equiv), *N*-deacetyl *N*-tosyl Colchicine sub-**10** (102.3 mg, 0.2 mmol, 1.0 equiv), and CsF (91.1 mg, 0.6 mmol, 3.0 equiv) in anhydrous MeCN (2.0 mL) under inert atmosphere was stirred at room temperature overnight. The resulting mixture was filtered through a short pad of silica gel (EtOAc eluent, 20 mL). All the volatiles were removed on rotary evaporator. Flash column chromatography (DCM: MeCN = 20: 1) afforded 109.5 mg (78%) of compound **16b** as a white solid. Mp: 118-120 °C; ^{1}H NMR (400 MHz, $CDCl_3$) δ 7.61 (d, J = 8.0 Hz, 2H), 7.41 (d, J = 7.6 Hz, 1H), 7.24 (dd, J_1 = 7.2, J_2 = 0.8 Hz, 1H), 7.16 (d, J = 8.0 Hz, 2H), 7.09 (t, J = 7.6 Hz, 1H), 6.45 (s, 1H), 6.40 (d, J = 9.6 Hz, 1H), 4.97 (d, J = 8.4 Hz, 1H), 4.90 (s, 1H), 4.79 (d, J = 9.6 Hz, 1H), 4.01-3.94 (m, 1H), 3.90 (s, 3H), 3.86 (s, 3H), 3.56 (s, 3H), 3.46 (s, 3H), 2.48-2.38 (m, 1H), 2.33 (s, 3H), 2.15 (dd, J_1 = 13.2, J_2 = 6.0 Hz, 1H), 1.88 (td, J_1 = 12.4, J_2 = 7.6 Hz, 1H), 1.67 (dd, J_1 = 12.8, J_2 = 7.6 Hz, 1H), 0.56 (s, 9H), 0.35 (s, 3H), 0.21 (s, 3H) ppm; ^{13}C NMR (100 MHz, $CDCl_3$) δ 186.5, 153.4, 151.9, 150.7, 146.0, 143.3, 143.0, 141.3, 136.9, 136.8, 136.1, 135.4, 134.3, 132.9, 129.7, 128.3, 127.7, 125.0, 124.2, 122.5, 108.8, 62.6, 61.6, 61.4, 56.2, 54.7, 52.9, 48.2, 42.4, 31.7, 26.6, 21.6, 17.2, -2.9, -3.6 ppm; IR (thin film) 3433, 2928, 2863, 1683, 1615, 1490, 1404, 1136, 1093, 1004, 851, 772, 724, 544, 477 (cm^{-1}); HRMS (ESI) m/z: calcd for $[C_{39}H_{47}NNaO_7SSi]^+$ 724.2735; found 724.2734.

The structure of **16b** was confirmed by NOESY experiment, as the following interactions were observed:

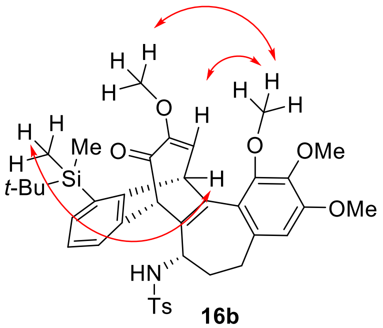

16b

Reaction of 1c with compound sub-11:

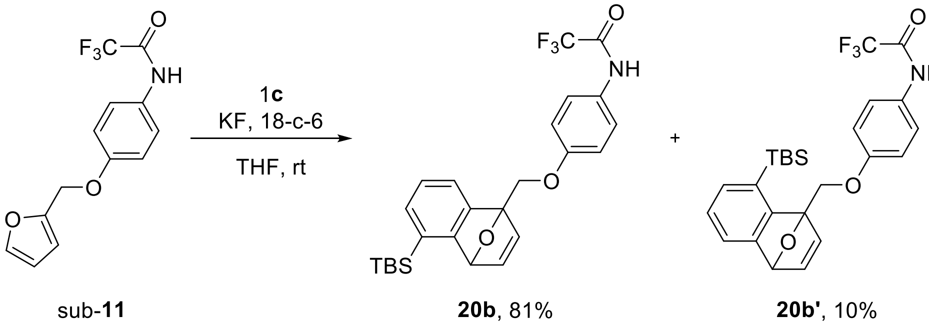

sub-**11** **20b**, 81% **20b'**, 10%

A mixture of aryne precursor **1c** (123.8 mg, 0.3 mmol, 1.5 equiv), compound sub-**11** (57.0 mg, 0.2 mmol, 1.0 equiv), 18-crown-6 (105.7 mg, 0.4 mmol, 2.0 equiv), and KF (46.5 mg, 0.8 mmol, 4.0 equiv) in anhydrous THF (2.0 mL) under inert atmosphere was stirred at room temperature overnight. The resulting mixture was filtered through a short pad of silica gel (EtOAc eluent, 20 mL). All the volatiles were removed on a rotary evaporator. Flash column chromatography (pet ether: EtOAc = 8: 1 to 4: 1) afforded 77.0 mg (81%) of compound **20b** as a white solid and 9.5 mg (10%) of compound **20b'** as a colorless oil.

Compound 20b: Mp: 157-159 °C; ^{1}H NMR (400 MHz, $CDCl_3$) δ 7.96 (s, 1H), 7.50 (d, J = 9.2 Hz, 2H), 7.24 (d, J = 6.8 Hz, 1H), 7.12-7.08 (m, 2H), 7.05 (d, J = 9.2 Hz, 2H), 7.01-6.96 (m, 2H), 5.86 (d, J = 2.0 Hz, 1H), 4.82 (d, J = 10.8 Hz, 1H), 4.70 (d, J = 10.4 Hz, 1H), 0.91 (s, 9H), 0.34 (s, 3H), 0.32 (s, 3H) ppm; ^{13}C NMR (100 MHz, $CDCl_3$) δ 157.2, 156.9, 154.9 (q, J = 37.0 Hz), 147.1, 144.6, 142.6, 131.6, 130.0, 128.8, 124.1, 122.5, 120.3, 116.1 (q, J = 286.7 Hz), 115.6, 90.8, 83.7, 66.4, 26.7, 17.4, -4.2, -4.7 ppm; IR (thin film) 3153, 2953, 2928, 2856, 1719, 1615, 1559, 1512, 1471, 1250, 1197, 1149, 1052, 993, 873, 830, 769, 731, 662, 584, 516 (cm^{-1}); HRMS (ESI) m/z: calcd for $[C_{25}H_{28}F_3NNaO_3Si]^+$ 498.1683; found 498.1693.

Compound 20b': ^{1}H NMR (400 MHz, $CDCl_3$) δ 7.86 (s, 1H), 7.50 (d, J = 9.2 Hz, 2H), 7.27 (d, J = 8.0 Hz, 1H), 7.20 (d, J = 8.0 Hz, 1H), 7.09 (dd, J_1 = 5.2, J_2 = 1.6 Hz, 1H), 7.04-7.01 (m, 3H), 6.98 (t, J = 7.2 Hz, 1H), 5.72 (d, J = 1.6 Hz, 1H), 5.06 (d, J = 10.8 Hz, 1H), 4.69 (d, J = 10.8 Hz, 1H), 0.93 (s, 9H), 0.38 (s, 3H), 0.37 (s, 3H) ppm; ^{13}C NMR (100 MHz, $CDCl_3$) δ 157.2, 155.0, 154.9 (q, J = 36.9 Hz), 149.9, 144.9, 142.4, 133.4, 130.2, 128.7, 124.1, 122.5, 121.1, 116.1 (q, J = 286.8 Hz), 115.7, 94.3, 81.9, 68.2, 27.3, 17.9, -1.3, -2.0 ppm; IR (thin film) 3148, 2955, 2929, 2856, 1719, 1606, 1555, 1511, 1463, 1383, 1284, 1235, 1198, 1155, 1059, 988, 937, 904, 823, 771, 717, 676, 515, 476 (cm^{-1}); HRMS (ESI) m/z: calcd for $[C_{25}H_{28}F_3NNaO_3Si]^+$ 498.1683; found 498.1689.

The structures of **20b** and **20b'** were confirmed by NOESY experiment, as the following interactions were observed:

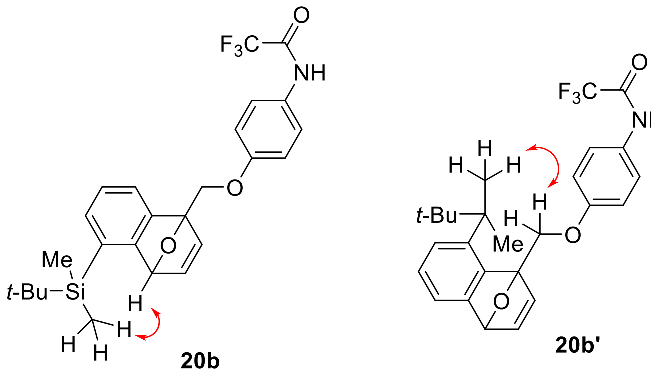

Reaction of 1c with compound sub-12:

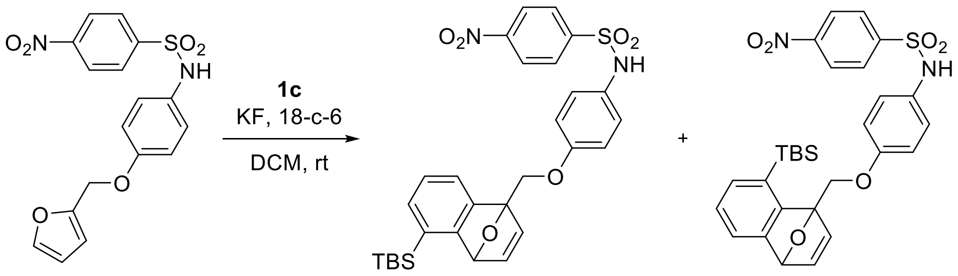

A mixture of aryne precursor **1c** (123.8 mg, 0.3 mmol, 1.5 equiv), compound sub-**12** (74.9 mg, 0.2 mmol, 1.0 equiv), 18-crown-6 (105.7 mg, 0.4 mmol, 2.0 equiv), and KF (46.5 mg, 0.8 mmol, 4.0 equiv) in anhydrous DCM (2.0 mL) under inert atmosphere was stirred at room temperature overnight. The resulting mixture was filtered through a short pad of silica gel (EtOAc eluent, 20 mL). All the volatiles were removed on a rotary evaporator. Flash column chromatography (DCM) afforded 64.4 mg (57%) of compound **21b** as a yellow solid and 15.8 mg (14%) of compound **21b'** as a colorless oil.

Compound 21b: Mp: 83-85 °C; ^{1}H NMR (400 MHz, $CDCl_3$) δ 8.27 (d, J = 8.8 Hz, 2H), 7.87 (d, J = 8.8 Hz, 2H), 7.21 (d, J = 6.8 Hz, 1H), 7.12-7.07 (m, 2H), 7.01-6.95 (m, 4H), 6.91 (d, J = 8.8 Hz, 2H), 6.64 (s, 1H), 5.87 (d, J = 1.6 Hz, 1H), 4.77 (d, J = 10.4 Hz, 1H), 4.65 (d, J = 10.4 Hz, 1H), 0.91 (s, 9H), 0.34 (s, 3H), 0.31 (s, 3H) ppm; ^{13}C NMR (100 MHz, $CDCl_3$) δ 158.0, 156.9, 150.4, 147.0, 145.0, 144.6, 142.5, 131.7, 130.1, 128.8, 128.4, 126.2, 124.4, 124.0, 120.2, 115.9, 90.7, 83.7, 66.4, 26.7, 17.4, -4.3, -4.7 ppm;

IR (thin film) 3533, 2950, 2928, 2883, 1633, 1532, 1508, 1402, 1349, 1170, 1092, 992, 855, 833, 737, 543, 464 (cm^{-1}); HRMS (ESI) m/z: calcd for $[C_{29}H_{32}N_2NaO_6SSi]^+$ 587.1643; found 587.1649.

Compound 21b': ^{1}H NMR (400 MHz, $CDCl_3$) δ 8.27 (d, J = 8.8 Hz, 2H), 7.84 (d, J = 9.2 Hz, 2H), 7.29-7.26 (m, 1H), 7.20 (dd, J_1 = 7.6, J_2 = 0.8 Hz, 1H), 7.09 (dd, J_1 = 5.6, J_2 = 1.6 Hz, 1H), 7.00-6.96 (m, 4H), 6.87 (d, J = 8.8 Hz, 2H), 6.52 (s, 1H), 5.73 (d, J = 1.6 Hz, 1H), 5.00 (d, J = 10.8 Hz, 1H), 4.62 (d, J = 10.8 Hz, 1H), 0.92 (s, 9H), 0.37 (s, 3H), 0.35 (s, 3H) ppm; ^{13}C NMR (100 MHz, $CDCl_3$) δ 158.0, 154.7, 150.4, 149.8, 144.9, 142.3, 133.4, 130.2, 128.8, 128.2, 126.3, 124.4, 124.2, 121.2, 115.9, 94.2, 81.9, 68.0, 27.3, 17.9, -1.4, -2.0 ppm; IR (thin film) 3432, 2925, 2854, 2363, 1529, 1507, 1400, 1091, 984, 857, 739, 544, 474 (cm^{-1}); HRMS (ESI) m/z: calcd for $[C_{29}H_{32}N_2NaO_6SSi]^+$ 587.1643; found 587.1648.

The structures of **21b** and **21b'** were confirmed by NOESY experiment, as the following interactions were observed:

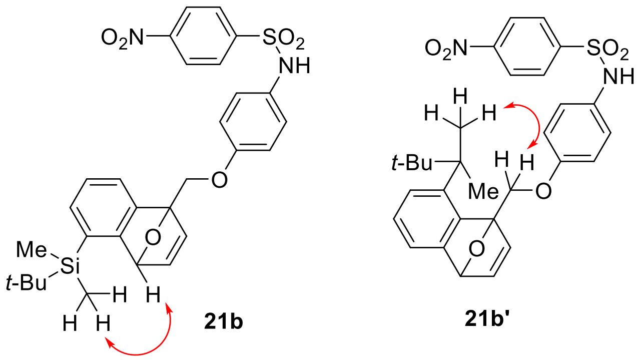

Reaction of 1c with compound sub-13:

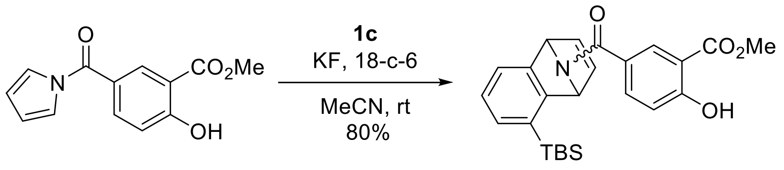

A mixture of aryne precursor **1c** (123.8 mg, 0.3 mmol, 1.5 equiv), compound sub-**13** (49.0 mg, 0.2 mmol, 1.0 equiv), 18-crown-6 (105.7 mg, 0.4 mmol, 2.0 equiv), and KF (46.5 mg, 0.8 mmol, 4.0 equiv) in anhydrous MeCN (2.0 mL) under inert atmosphere was stirred at room temperature overnight. The resulting mixture was filtered through a short pad of silica gel (EtOAc eluent, 20 mL). All the volatiles were removed on a rotary evaporator. Flash column chromatography (pet ether: EtOAc = 10: 1) afforded 69.7 mg (80%) of compound **22b** as a 1: 0.6 mixture of conformational isomers. ^{1}H NMR (400 MHz, $CDCl_3$) δ 11.06 (s, 1.6H), 8.11 (d, J = 1.6 Hz, 1.6H), 7.67 (d, J = 8.4 Hz, 1.6H), 7.36 (d, J = 6.8 Hz, 1H), 7.22-7.06 (m, 3.8H), 7.01-6.96 (m, 3.2H), 6.89 (s, 1.6H), 6.07 (s, 0.6H), 5.91 (s, 1H), 5.63 (s, 1H), 5.53 (s, 0.6H), 3.95 (s, 4.8H), 0.94 (s, 5.4H), 0.76 (s, 9H), 0.42 (s, 1.8H), 0.38 (s, 1.8H), 0.23 (s, 3H), 0.17 (s, 3H) ppm; ^{13}C NMR (100 MHz, $CDCl_3$) δ 170.3, 167.9, 164.0, 163.9, 154.8, 154.7, 147.1, 145.2, 145.1, 142.3, 142.0, 135.7, 131.8, 131.0, 130.0, 125.8, 124.4, 124.1, 122.4, 121.0, 117.9, 112.4, 69.8, 68.4, 65.4, 64.1, 52.7, 26.7, 26.5, 17.4, 17.1, -4.3, -4.5 ppm; IR (thin film) 3422, 2953, 2855, 1679, 1654, 1591, 1490, 1294, 1284, 1245, 1122, 1088, 835, 770, 689, 540, 478 (cm^{-1}); HRMS (ESI) m/z: calcd for $[C_{25}H_{29}NNaO_4Si]^+$ 458.1758; found 458.1773.

Reaction of 1c with Furosemide methyl ester sub-14:

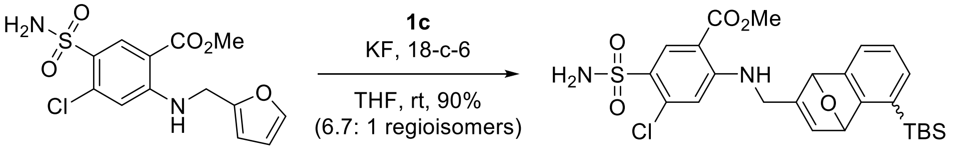

Furosemide methyl ester sub-**14** **23b**

A mixture of aryne precursor **1c** (123.8 mg, 0.3 mmol, 1.5 equiv), Furosemide methyl ester sub-**14** (69.0 mg, 0.2 mmol, 1.0 equiv), 18-crown-6 (105.7 mg, 0.4 mmol, 2.0 equiv), and KF (46.5 mg, 0.8 mmol, 4.0 equiv) in anhydrous THF (2.0 mL) under inert atmosphere was stirred at room temperature overnight. The resulting mixture was filtered through a short pad of silica gel (EtOAc eluent, 20 mL). All the volatiles were removed on a rotary evaporator. Flash column chromatography (DCM) afforded 96.3 mg (90%) of compound **23b** as a 6.7: 1 mixture of regioisomers. ^{1}H NMR (400 MHz, $CDCl_3$) δ 8.63 (t, J = 4.4 Hz, 1H), 8.58-8.54 (m, 1.3H), 7.23-7.18 (m, 1.15H), 7.16-7.08 (m, 2.3H), 7.02-6.97 (m, 1.15H), 6.95 (s, 1H), 6.89-6.83 (m, 1.3H), 5.89 (d, J = 1.6 Hz, 1H), 5.74 (d, J = 1.6 Hz, 0.15H), 5.07 (s, 2H), 5.05 (s, 0.3H), 4.36 (dd, J_1 = 12.8, J_2 = 5.6 Hz, 0.15H), 4.21 (dd, J_1 = 13.6, J_2 = 5.6 Hz, 1H), 4.03 (dd, J_1 = 13.6, J_2 = 4.4 Hz, 1H), 3.96 (dd, J_1 = 12.8, J_2 = 3.2 Hz, 0.15H), 3.86 (s, 0.45H), 3.85 (s, 3H), 0.95 (s, 1.35H), 0.91 (s, 9H), 0.43 (s, 0.45H), 0.43 (s, 0.45H), 0.34 (s, 3H), 0.31 (s, 3H) ppm; ^{13}C NMR (100 MHz, $CDCl_3$) δ 167.7, 157.1, 153.6, 153.5, 150.0, 146.8, 146.6, 146.1, 142.4, 142.1, 137.4, 134.4, 134.3, 133.5, 131.8, 130.3, 129.9, 125.5, 125.4, 124.2, 124.1, 121.2, 119.6, 113.7, 113.6, 108.5, 93.7, 91.1, 83.7, 82.0, 52.4, 45.1, 42.5, 27.3, 26.7, 17.9, 17.4, -1.2, -1.9, -4.3, -4.8 ppm; IR (thin film) 3414, 3320, 3117, 2954, 2929, 2855, 2371, 1695, 1597, 1571, 1501, 1396, 1328, 1227, 1164, 1089, 996, 935, 832, 769, 720, 538, 474 (cm^{-1}); HRMS (ESI) m/z: calcd for $[C_{25}H_{31}ClN_2NaO_5SSi]^+$ 557.1304; found 557.1314.

Reaction of 1c with compound 24:

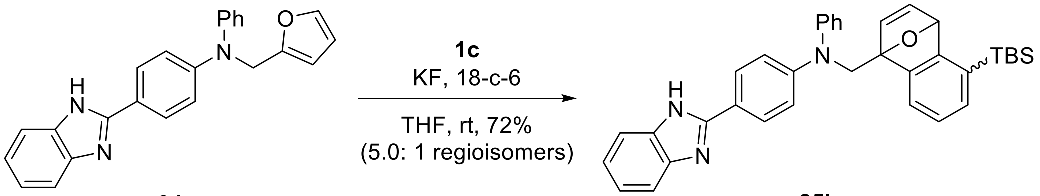

24 **25b**

A mixture of aryne precursor **1c** (123.8 mg, 0.3 mmol, 1.5 equiv), compound **24** (73.1 mg, 0.2 mmol, 1.0 equiv), 18-crown-6 (105.7 mg, 0.4 mmol, 2.0 equiv), and KF (46.5 mg, 0.8 mmol, 4.0 equiv) in anhydrous THF (2.0 mL) under inert atmosphere was stirred at room temperature overnight. The resulting mixture was filtered through a short pad of silica gel (EtOAc eluent, 20 mL). All the volatiles were removed on a rotary evaporator. Flash column chromatography (DCM) afforded 80.0 mg (72%) of compound **25b** as a 5.0: 1 mixture of regioisomers. Upon recrystallization with EtOAc-pet ether, **25b'** was obtained as a single isomer.

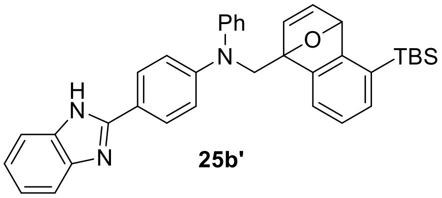

25b'

Compound 25b': Mp: 146-148 °C; ^{1}H NMR (400 MHz, $CDCl_3$) δ 7.88 (d, J = 8.8 Hz, 2H), 7.56 (brs, 2H), 7.41-7.36 (m, 2H), 7.32-7.29 (m, 2H), 7.21-7.16 (m, 3H), 7.08-7.01 (m, 4H), 6.94-6.87 (m, 2H), 6.61 (d, J = 5.6 Hz, 1H), 5.80 (d, J = 1.6 Hz, 1H), 4.91 (d, J = 16.0 Hz, 1H), 4.52 (d, J = 16.4 Hz, 1H), 0.89 (s, 9H), 0.31 (s, 3H), 0.27 (s, 3H) ppm; ^{13}C NMR (100 MHz, $CDCl_3$) δ 157.4, 152.3, 150.9, 148.3, 147.3, 144.0, 143.5, 142.1, 131.5, 130.1, 129.9, 129.8, 127.8, 126.0, 125.3, 125.1, 124.9, 123.9, 122.7, 120.5, 119.8, 117.3, 117.0, 93.1, 83.1, 52.2, 26.7, 17.4, -4.3, -4.7 ppm; IR (thin film) 3429, 2936, 1614, 1497, 1396, 1258, 1099, 992, 862, 829, 747, 626, 543, 468 (cm^{-1}); HRMS (ESI) m/z: calcd for $[C_{36}H_{38}N_3OSi]^+$ 556.2779; found 556.2783.

The structures of **25b'** were confirmed by NOESY experiment, as the following interactions were observed:

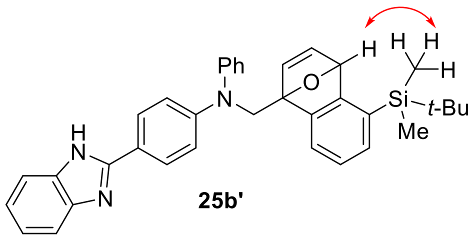

25b'

Reaction of 1c with compound sub-16:

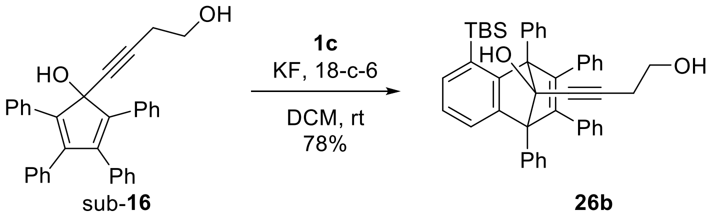

A mixture of aryne precursor **1c** (123.8 mg, 0.3 mmol, 1.5 equiv), compound sub-**16** (90.9 mg, 0.2 mmol, 1.0 equiv), 18-crown-6 (105.7 mg, 0.4 mmol, 2.0 equiv), and KF (46.5 mg, 0.8 mmol, 4.0 equiv) in anhydrous DCM (2.0 mL) under inert atmosphere was stirred at room temperature overnight. The resulting mixture was filtered through a short pad of silica gel (EtOAc eluent, 20 mL). All the volatiles were removed on a rotary evaporator. Flash column chromatography (pet ether: EtOAc = 5: 1) afforded 100.6 mg (78%) of compound **26b** as a white solid. Mp: 115-117 °C; ^{1}H NMR (400 MHz, $CDCl_3$) δ 7.67-7.62 (m, 2H), 7.60 (d, J = 7.6 Hz, 1H), 7.54 (dd, J_1 = 7.6, J_2 = 0.8 Hz, 1H), 7.48-7.44 (m, 2H), 7.35-7.26 (m, 6H), 7.23-7.16 (m, 4H), 7.11-7.07 (m, 2H), 7.02-6.97 (m, 3H), 6.89-6.85 (m, 2H), 3.56 (s, 2H), 2.52 (s, 1H), 2.35 (td, J_1 = 6.0, J_2 = 1.2 Hz, 2H), 1.61 (brs, 1H), 1.04 (s, 9H), 0.00 (s, 3H), -0.94 (s, 3H) ppm; ^{13}C NMR (100 MHz, $CDCl_3$) δ 155.6, 154.6, 150.5, 149.7, 137.7, 137.3, 135.9, 135.8, 135.4, 134.0, 131.6, 131.2, 130.1, 129.6, 128.1, 127.7, 127.6, 127.4, 127.2, 127.0, 126.9, 124.8, 123.8, 100.5, 83.8, 81.8, 77.0, 72.2, 61.1, 29.0, 23.3, 19.5, 0.1, -3.6 ppm; IR (thin film) 3422, 3055, 2929, 2856, 1602, 1498, 1473, 1384, 1251, 1090, 988, 908, 833, 810, 776, 746, 729, 702, 586, 544 (cm^{-1}); HRMS (ESI) m/z: calcd for $[C_{45}H_{44}NaO_2Si]^+$ 667.3003; found 667.3006.

2.5 Asymmetric synthesis of dihydrexidine:

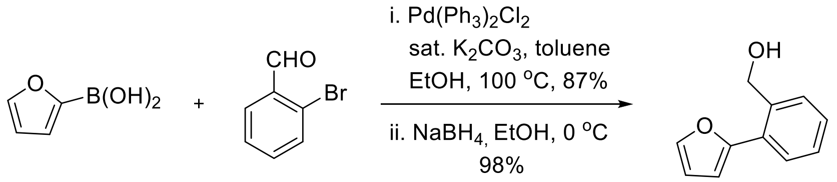

30

2-(Furan-2-yl)benzaldehyde: A mixture of furan-2-ylboronic acid (2.46 g, 22.0 mmol, 1.1 equiv), 2-bromobenzaldehyde (2.33 mL, 20.0 mmol, 1.0 equiv), $Pd(PPh_3)_2Cl_2$ (701.9 mg, 1.0 mmol, 0.05 equiv), and saturated aqueous solution of K_2CO_3 (10.0 mL) in toluene (30.0 mL) and ethanol (20.0 mL) was heated at 100 °C for four hours under nitrogen atmosphere, which was then cooled to room temperature. The separated aqueous layer was extracted with EtOAc (20 mL x 3), and the combined organic layers were washed with brine (50 mL), dried over Na_2SO_4, filtered, and concentrated. Flash column chromatography (pet ether: EtOAc = 20: 1) afforded 3.0 g (87%) of 2-(furan-2-yl)benzaldehyde as a colorless oil, the ^{1}H NMR of which is identical with that reported in literature.[33] ^{1}H NMR (400 MHz, $CDCl_3$) δ 10.38 (s, 1H), 7.98 (d, J = 8.0 Hz, 1H), 7.69 (d, J = 7.6 Hz, 1H), 7.64-7.59 (m, 2H), 7.44 (t, J = 7.6 Hz, 1H), 6.64 (d, J = 3.2 Hz, 1H), 6.57 (dd, J_1 = 3.2, J_2 = 2.0 Hz, 1H) ppm.

Compound 30: To a solution of 2-(furan-2-yl)benzaldehyde (3.0 g, 17.42 mmol, 1.0 equiv) in anhydrous EtOH (10.0 mL) at 0 °C was added $NaBH_4$ (988.7 mg, 26.13 mmol, 1.5 equiv). After 10 minutes, it was quenched by water (20 mL). The separated aqueous layer was extracted with EtOAc (20 mL x 3), and the combined organic layers were washed with brine (50 mL), dried over Na_2SO_4, filtered, and concentrated. Flash column chromatography (pet ether: EtOAc = 8: 1) afforded 2.97 g (98%) of compound **30** as a white solid, the ^{1}H NMR of which is identical with that reported in literature.[34] ^{1}H NMR (400 MHz, $CDCl_3$) δ 7.69-7.63 (m, 1H), 7.56-7.48 (m, 2H), 7.39-7.31 (m, 2H), 6.68 (d, *J* = 3.2 Hz, 1H), 6.52 (dd, J_1 = 3.2, J_2 = 2.0 Hz, 1H), 4.80 (s, 2H), 2.10 (brs, 1H) ppm.

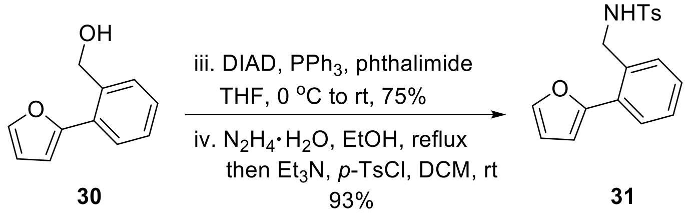

2-(2-(Furan-2-yl)benzyl)isoindoline-1,3-dione: To a solution of PPh_3 (5.64 g, 21.49 mmol, 1.3 equiv) in anhydrous THF (30 mL) at 0 °C under inert atmosphere was added dropwise DIAD (4.23 mL, 21.49 mmol, 1.3 equiv). After stirring for 10 minutes at 0 °C, a solution of compound **30** (2.88 g, 16.53 mmol, 1.0 equiv) and phthalimide (3.74 g, 21.49 mmol, 1.3 equiv) in anhydrous THF (30 mL) was added dropwise over five minutes. The resulting mixture was allowed to warm to room temperature overnight, which was then quenched with water (20 mL). The separated aqueous layer was extracted with EtOAc (25 mL x 3), and the combined organic layers were washed with brine (40 mL), dried over Na_2SO_4, filtered, and concentrated. Flash column chromatography (pet ether: EtOAc = 15: 1) afforded 3.76 g (75%) of 2-(2-(furan-2-yl)benzyl)isoindoline-1,3-dione as a white solid. Mp: 113-115 °C; ^{1}H NMR (400 MHz, $CDCl_3$) δ 7.90-7.86 (m, 2H), 7.76-7.72 (m, 2H), 7.61 (d, *J* = 7.6 Hz, 1H), 7.58-7.54 (m, 1H), 7.29 (t, *J* = 7.2 Hz, 1H), 7.23 (t, *J* = 8.0 Hz, 1H), 7.14 (d, *J* = 7.6 Hz, 1H), 6.63 (d, *J* = 3.2 Hz, 1H), 6.53 (dd, J_1 = 3.2, J_2 = 1.6 Hz, 1H), 5.16 (s, 2H) ppm; ^{13}C NMR (100 MHz, $CDCl_3$) δ 168.3, 153.2, 142.6, 134.3, 133.0, 132.2, 129.6, 128.6, 128.3, 127.6, 126.6, 123.6, 111.6, 109.1, 40.0 ppm; IR (thin film) 3064, 1771, 1715, 1613, 1505, 1467, 1392, 959, 760, 731, 717,530 (cm^{-1}); HRMS (ESI) m/z: calcd for $[C_{19}H_{14}NO_3]^+$ 304.0968; found 304.0978.

Compound 31: A solution of 2-(2-(furan-2-yl)benzyl)isoindoline-1,3-dione (3.03 g, 10.0 mmol, 1.0 equiv) and 80% hydrazine hydrate ($N_2H_4 \cdot H_2O$) (1.82 mL, 30.0 mmol, 3.0 equiv) in EtOH (40 mL) was heated under reflux for three hours. The resulting mixture was cooled to room temperature and filtered to remove phthalhydrazide. The precipitate was further washed with cold EtOH (10 mL x 2). The combined organic layers were concentrated to obtain a crude white solid, which was used directly in next step without further purification.

To the above crude white solid in anhydrous DCM (40 mL) at room temperature under inert atmosphere was added Et_3N (3.47 mL, 25.0 mmol, 2.5 equiv) and *p*-TsCl (2.29 g, 12.0 mmol, 1.2 equiv). The resulting mixture was stirred at room temperature overnight, which was then quenched with water (20 mL). The separated aqueous layer was extracted with DCM (20 mL x 3), and the combined organic layers were washed with brine (40 mL), dried over Na_2SO_4, filtered, and concentrated. Flash column chromatography (pet ether: DCM = 1: 1 to 1: 2) afforded 3.04 g (93% in two steps) of compound **31** as a white solid. Mp: 99-101 °C; ^{1}H NMR (400 MHz, $CDCl_3$) δ 7.69 (d, *J* = 8.0 Hz, 2H), 7.49 (d, *J* = 7.2 Hz, 1H), 7.39-7.35 (m, 1H), 7.31-7.27 (m, 2H), 7.26-7.24 (m, 2H), 7.23-7.17 (m, 1H), 6.49 (d, *J* = 3.2 Hz, 1H), 6.45 (dd, J_1 = 3.2, J_2 = 1.6 Hz, 1H), 4.82 (t, *J* = 6.0 Hz, 1H), 4.30 (d, *J* = 6.4 Hz, 2H), 2.42 (s, 3H) ppm; ^{13}C NMR (100 MHz, $CDCl_3$) δ 153.2, 143.4, 142.7, 137.2, 132.7, 130.9, 130.1, 129.7, 128.3, 128.2, 127.3, 111.8, 108.7, 46.6, 21.7 ppm; IR (thin film) 3265, 2921, 2850, 1596, 1403, 1320, 1156, 1091, 1042, 812, 760, 660, 553, 542 (cm^{-1}); HRMS (ESI) m/z: calcd for $[C_{18}H_{17}NNaO_3S]^+$ 350.0821; found 350.0820.

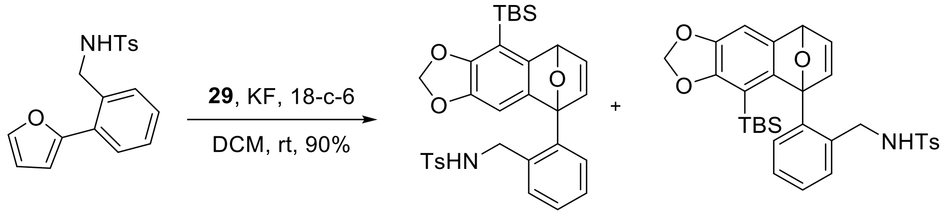

31 **32a**, 41% **32b**, 49%

A mixture of aryne precursor **29** (2.74 g, 6.0 mmol, 1.5 equiv), **31** (1.31 g, 4.0 mmol, 1.0 equiv), 18-crown-6 (2.11 g, 8.0 mmol, 2.0 equiv), and KF (929.6 mg, 16.0 mmol, 4.0 equiv) in anhydrous DCM (40 mL) under inert atmosphere was stirred at room temperature overnight. The resulting mixture was filtered through a short pad of silica gel (EtOAc eluent, 20 mL). All the volatiles were removed on a rotary evaporator. Flash column chromatography (pet ether: EtOAc = 5: 1) afforded 921.3 mg (41%) of compound **32a** as a colorless oil and 1.10 g (49%) of compound **32b** as a colorless oil.

Compound 32a: ^{1}H NMR (400 MHz, $CDCl_3$) δ 7.52 (d, J = 8.4 Hz, 2H), 7.49 (d, J = 8.0 Hz, 1H), 7.35-7.31 (m, 1H), 7.29 (d, J = 5.6 Hz, 1H), 7.23-7.21 (m, 2H), 7.14-7.11 (m, 3H), 6.23 (s, 1H), 5.85 (d, J = 1.6 Hz, 1H), 5.83 (d, J = 2.0 Hz, 1H), 5.81 (d, J = 1.2 Hz, 1H), 5.60-5.55 (m, 1H), 4.12-4.08 (m, 2H), 2.36 (s, 3H), 0.94 (s, 9H), 0.40 (s, 3H), 0.37 (s, 3H) ppm; ^{13}C NMR (100 MHz, $CDCl_3$) δ 150.0, 149.3, 145.0, 144.8, 144.5, 143.4, 142.8, 138.3, 135.8, 133.5, 132.8, 129.5, 128.9, 128.2, 128.1, 127.1, 113.4, 104.6, 100.7, 94.2, 83.7, 46.5, 26.6, 21.7, 17.8, -3.3, -3.7 ppm; IR (thin film) 3301, 3036, 2927, 2856, 1598, 1495, 1470, 1389, 1259, 1160, 1050, 924, 810, 773, 698, 665, 552 (cm^{-1}); HRMS (ESI) m/z: calcd for $[C_{31}H_{35}NNaO_5SSi]^+$ 584.1897; found 584.1902.

Compound 32b: ^{1}H NMR (400 MHz, $CDCl_3$) δ 7.64 (d, J = 7.6 Hz, 1H), 7.50 (d, J = 8.0 Hz, 2H), 7.43 (d, J = 5.6 Hz, 1H), 7.35-7.27 (m, 3H), 7.16 (d, J = 8.0 Hz, 2H), 7.11 (dd, J_1 = 5.2, J_2 = 1.6 Hz, 1H), 6.59 (s, 1H), 5.88 (d, J = 1.2 Hz, 1H), 5.78 (d, J = 1.2 Hz, 1H), 5.53-5.46 (m, 2H), 3.92-3.87 (m, 2H), 2.43 (s, 3H), 0.64 (s, 9H), -0.18 (s, 3H), -0.45 (s, 3H) ppm; ^{13}C NMR (100 MHz, $CDCl_3$) δ 151.3, 150.7, 145.4, 143.9, 143.0, 142.8, 142.7, 137.6, 137.5, 135.9, 133.6, 130.3, 130.0, 129.5, 128.0, 127.1, 116.2, 104.4, 100.2, 97.5, 81.7, 46.5, 27.4, 21.7, 18.1, -1.5, -3.1 ppm; IR (thin film) 3289, 3063, 2927, 2856, 1598, 1494, 1470, 1374, 1260, 1162, 1055, 927, 811, 769, 701, 554, 544 (cm^{-1}); HRMS (ESI) m/z: calcd for $[C_{31}H_{35}NNaO_5SSi]^+$ 584.1897; found 584.1901.

The structures of **32a** and **32b** were confirmed by NOESY experiment, as the following interactions were observed:

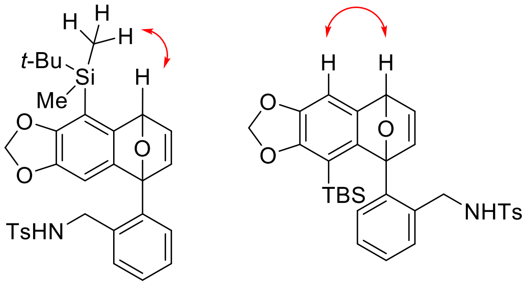

32a **32b**

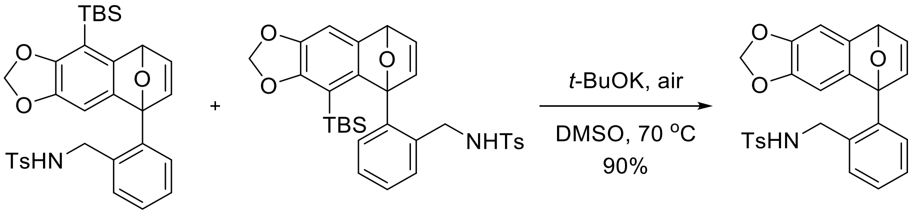

32a **32b** **33**

To a solution of mixture **32a** and **32b** (1.69 g, 3.0 mmol, 1.0 equiv) in DMSO (5.0 mL) under air at room temperature was added t-BuOK (504.9 mg, 4.5 mmol, 1.5 equiv). The resulting mixture was heated to 70 °C for overnight, which was then cooled to room temperature. It was quenched with water (20 mL).

The separated aqueous layer was extracted with EtOAc (20 mL x 3), and the combined organic layers were washed with water (200 mL x 3), brine (50 mL), dried over Na_2SO_4, filtered, and concentrated. Flash column chromatography (pet ether: DCM = 1: 2) afforded 1.21 g (90%) of **33** as a pale-yellow oil. ^1H NMR (400 MHz, $CDCl_3$) δ 7.55-7.51 (m, 3H), 7.37-7.34 (m, 1H), 7.33-7.27 (m, 3H), 7.16-7.12 (m, 3H), 6.71 (s, 1H), 6.20 (s, 1H), 5.91 (d, J = 1.2 Hz, 1H), 5.86 (d, J = 1.2 Hz, 1H), 5.58 (d, J = 1.6 Hz, 1H), 5.46 (dd, J_1 = 8.8, J_2 = 4.0 Hz, 1H), 4.05 (dd, J_1 = 13.2, J_2 = 9.2 Hz, 1H), 3.94 (dd, J_1 = 12.8, J_2 = 4.0 Hz, 1H), 2.40 (s, 3H) ppm; ^{13}C NMR (100 MHz, $CDCl_3$) δ 145.5, 144.9, 144.6, 144.3, 143.2, 142.9, 137.6, 135.8, 133.3, 133.2, 129.5, 129.3, 128.4, 128.2, 127.2, 103.9, 103.8, 101.6, 94.7, 82.6, 46.5, 21.6 ppm; IR (thin film) 3298, 3061, 2890, 1619, 1598, 1497, 1460, 1322, 1159, 1037, 921, 813, 771, 689, 552 (cm^{-1}); HRMS (ESI) m/z: calcd for $[C_{25}H_{22}NO_5S]^+$ 448.1213; found 448.1221.

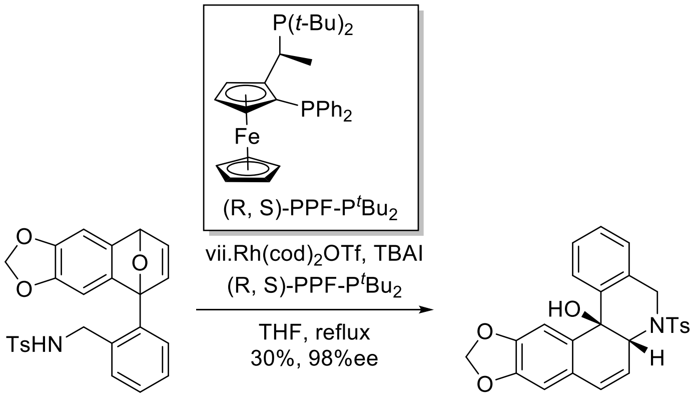

33 **34**

A mixture of $Rh(cod)_2OTf$ (46.8 mg, 0.1 mmol, 0.05 equiv) and (R, S)-PPF-P^tBu_2 Josiphos ligand (65.1 mg, 0.12 mmol, 0.06 equiv) in anhydrous THF (30 mL) under inert atmosphere was pre-stirred at room temperature for 10 minutes. Tetrabutylammoniuiodide (TBAI) (147.7 mg, 0.4 mmol, 0.2 equiv) was then added, and the resulting mixture was stirred for another 10 minutes. A solution of compound **33** (895 mg, 2.0 mmol, 1.0 equiv) in anhydrous THF (10 mL) was added dropwise, and the resulting mixture was heated to reflux overnight. It was cooled to room temperature and quenched with water (10 mL). The separated aqueous layer was extracted with EtOAc (10 mL x 3), and the combined organic layers were washed with brine (20 mL), dried over Na_2SO_4, filtered, and concentrated. Flash column chromatography (pet ether: EtOAc = 1: 3) afforded 268.5 mg (30%) of compound **34** as a light brown solid, which was determined to be 98% ee by HPLC (HPLC conditions: Chiralcel AD-H column, hexane/i-PrOH = 80/20, 1.00 mL/min, λ = 250nm, t_R(major) = 25.0 min, t_R(minor) = 37.2 min) (Supplementary Fig **1**). Mp: 205-207 ℃; ^1H NMR (400 MHz, $CDCl_3$) δ 7.82 (d, J = 8.4 Hz, 2H), 7.55 (s, 1H), 7.36 (d, J = 8.0 Hz, 2H), 7.19 (td, J_1 = 7.6, J_2 = 1.6 Hz, 1H), 7.10 (t, J = 7.6 Hz, 1H), 7.06 (d, J = 7.6 Hz, 1H), 7.01 (d, J = 7.6 Hz, 1H), 6.47 (s, 1H), 6.06 (dd, J_1 = 10.0, J_2 = 2.8 Hz, 1H), 6.01 (d, J = 1.6 Hz, 1H), 6.00 (d, J = 1.2 Hz, 1H), 5.18 (dd, J_1 = 10.0, J_2 = 2.0 Hz, 1H), 5.11 (t, J = 2.0 Hz, 1H), 4.85 (d, J = 15.6 Hz, 1H), 4.34 (d, J = 15.6 Hz, 1H), 2.83 (s, 1H), 2.46 (s, 3H) ppm; ^{13}C NMR (100 MHz, $CDCl_3$) δ 147.5, 147.4, 144.3, 136.6, 136.3, 132.4, 131.7, 131.0, 130.3, 128.5, 128.2, 127.8, 127.6, 126.0, 125.9, 123.7, 108.0, 107.4, 101.5, 72.5, 61.7, 45.1, 21.8 ppm; IR (thin film) 3507, 2921, 1597, 1501, 1482, 1351, 1255, 1163, 1038, 963, 769, 671, 568, 550 (cm^{-1}); HRMS (ESI) m/z: calcd for $[C_{25}H_{22}NO_5S]^+$ 448.1213; found 448.1217. The X-ray crystal structure of compound **34** was determined and its structural information was shown in Supplementary Fig 3 and Supplementary Tables 6-8.

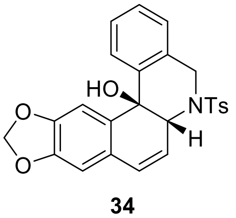

34

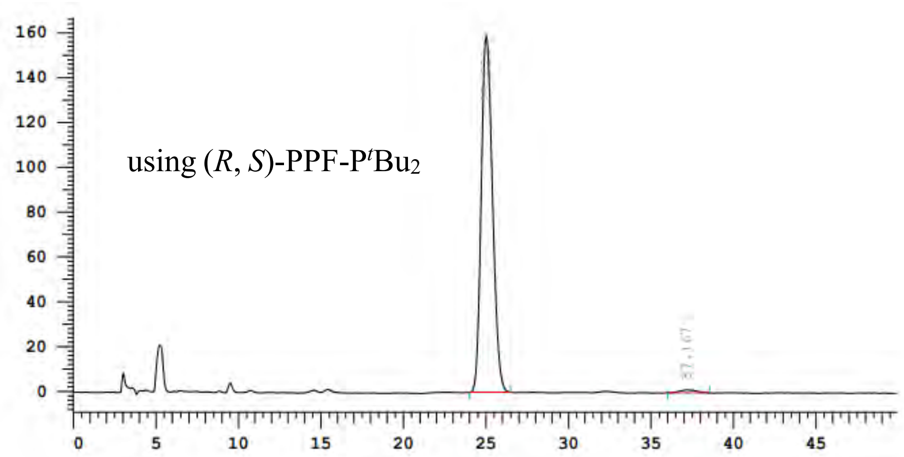

Peak #	Retention Time (min)	Area(mAU*s)	Area(%)
1	25.020	7504011	98.767
2	37.167	93707	1.233
		7597718	100.000

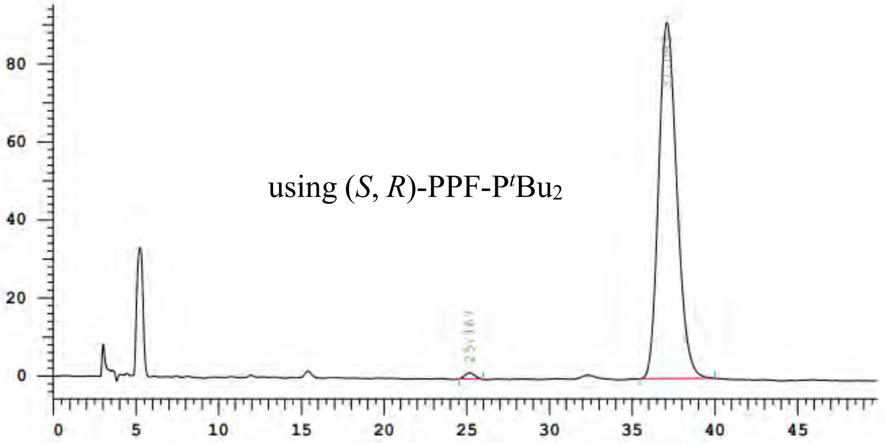

Peak #	Retention Time (min)	Area(mAU*s)	Area(%)
1	25.167	65791	0.942
2	37.093	6920481	99.058
		6986272	100.000

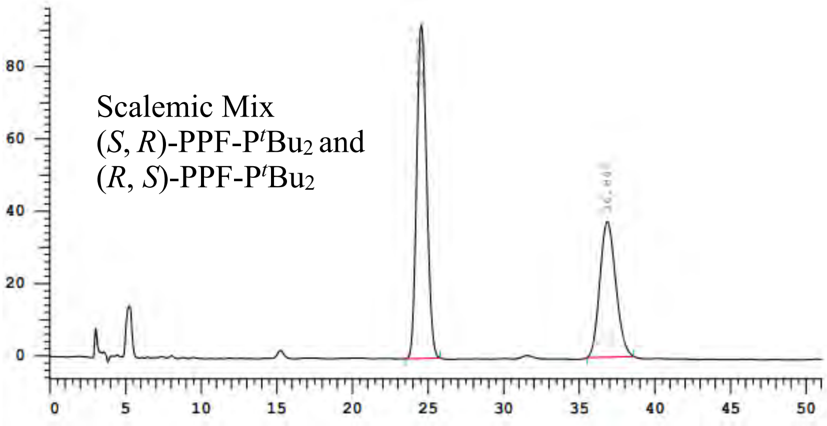

Supplementary Figure 1. HPLC study on compound **34.**

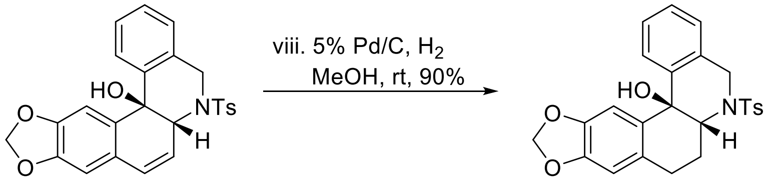

34 **S80**

Compound S80: A suspension of compound **34** (223.8 mg, 0.5 mmol, 1.0 equiv) and 5% palladium on charcoal (20 mg) in methanol (10 mL) under hydrogen atmosphere was stirred at room temperature overnight. Hydrogen was replaced by nitrogen, and the resulting mixture was filtered through a short pad of silica gel (EtOAc eluent, 20 mL). All the volatiles were removed on a rotary evaporator. Flash column chromatography (pet ether: DCM = 1: 4) afforded 202.3 mg (90%) of compound **S80** as a white solid. Mp: 96-98 °C; ^{1}H NMR (400 MHz, $CDCl_3$) δ 7.77 (d, J = 8.0 Hz, 2H), 7.29 (d, J = 8.0 Hz, 2H), 7.25 (s, 1H), 7.21-7.12 (m, 3H), 7.08 (d, J = 7.6 Hz, 1H), 6.51 (s, 1H), 5.95 (d, J = 1.2 Hz, 1H), 5.94 (d, J = 1.2 Hz, 1H), 4.72 (d, J = 15.2 Hz, 1H), 4.31 (d, J = 15.6 Hz, 1H), 4.26 (dd, J_1 = 10.8, J_2 = 4.4 Hz, 1H), 2.81-2.72 (m, 1H), 2.59-2.52 (m, 1H), 2.49 (s, 1H), 2.41 (s, 3H), 1.86-1.79 (m, 1H), 1.67-1.61 (m, 1H) ppm; ^{13}C NMR (100 MHz, $CDCl_3$) δ 147.5, 146.3, 143.9, 138.3, 136.1, 131.8, 131.2, 130.0, 129.3, 128.1, 128.0, 127.7, 127.6, 126.6, 108.3, 108.2, 101.2, 72.4, 59.9, 44.6, 27.9, 24.6, 21.7 ppm; IR (thin film) 3500, 2923, 1597, 1502, 1483, 1340, 1237, 1161, 1039, 937, 766, 665, 554 (cm^{-1}); HRMS (ESI) m/z: calcd for $[C_{25}H_{23}NNaO_5S]^+$ 472.1189; found 472.1196.

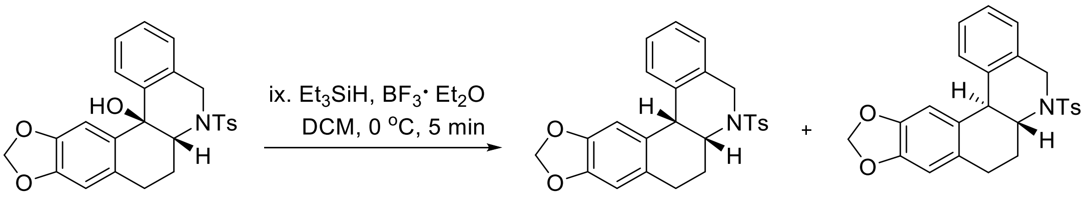

S80 **S81**, 45% **35**, 44%

To a solution of compound **S80** (134.9 mg, 0.3 mmol, 1.0 equiv) and triethylsilane (Et_3SiH) (0.96 mL, 6.0 mmol, 20.0 equiv) in DCM (1 mL) at 0 °C was slowly added boron trifluoride diethyl etherate ($BF_3 \cdot Et_2O$) (0.15 mL, 1.2 mmol, 4.0 equiv). After five minutes, the resulting mixture was quenched with saturated aqueous NH_4Cl (10 mL). The separated aqueous layer was extracted with EtOAc (20 mL x 3), and the combined organic layers were washed with brine (20 mL), dried over Na_2SO_4, filtered, and concentrated. Flash column chromatography with toluene afforded 58.5 mg (45%) of compound **S81** as a colorless oil and 57.2 mg (44%) of compound **35** as a colorless oil.

Compound S81: ^{1}H NMR (400 MHz, $CDCl_3$) δ 7.72 (d, J = 8.0 Hz, 2H), 7.26 (d, J = 8.0 Hz, 2H), 7.16-7.06 (m, 3H), 6.92-6.88 (m, 1H), 6.59 (s, 1H), 6.52 (s, 1H), 5.93 (d, J = 1.2 Hz, 1H), 5.92 (d, J = 1.2 Hz, 1H), 4.44 (d, J = 14.8 Hz, 1H), 4.37 (d, J = 14.8 Hz, 1H), 4.31-4.26 (m, 1H), 3.82 (d, J = 5.2 Hz, 1H), 2.85-2.76 (m, 1H), 2.73-2.65 (m, 1H), 2.40 (s, 3H), 1.90-1.83 (m, 1H), 1.71-1.63 (m, 1H) ppm; ^{13}C NMR (100 MHz, $CDCl_3$) δ 147.0, 145.9, 143.6, 137.8, 136.2, 132.1, 129.9, 129.6, 128.2, 128.1, 127.6, 127.5, 126.7, 126.5, 110.5, 109.0, 101.1, 53.6, 45.7, 42.9, 28.1, 26.2, 21.7 ppm; IR (thin film) 2922, 1597, 1502, 1483, 1344, 1224, 1161, 1038, 940, 849, 741, 664, 559, 550 (cm^{-1}); HRMS (ESI) m/z: calcd for $[C_{25}H_{24}NO_4S]^+$ 434.1421; found 434.1424.

Compound 35: ^{1}H NMR (400 MHz, $CDCl_3$) δ 7.42 (d, J = 8.0 Hz, 2H), 7.22 (d, J = 8.0 Hz, 1H), 7.09 (t, J = 7.6 Hz, 1H), 7.00 (d, J = 8.0 Hz, 2H), 6.91 (t, J = 7.2 Hz, 1H), 6.84 (s, 1H), 6.75 (d, J = 7.2 Hz, 1H), 6.67 (s, 1H), 5.96 (d, J = 1.2 Hz, 1H), 5.93 (d, J = 1.6 Hz, 1H), 4.65 (d, J = 16.0 Hz, 1H), 4.45 (d, J = 16.0 Hz, 1H), 4.05 (d, J = 11.2 Hz, 1H), 3.13 (td, J_1 = 11.6, J_2 = 2.4 Hz, 1H), 3.00-2.87 (m, 2H), 2.80-2.73 (m, 1H), 2.28 (s, 3H), 2.04-1.93 (m, 1H) ppm; ^{13}C NMR (100 MHz, $CDCl_3$) δ 146.6, 145.8, 143.4, 138.8, 135.7, 135.5, 131.3, 129.4, 127.7, 127.4, 126.5, 125.9, 125.8, 123.8, 109.6, 109.3, 101.2, 61.3, 47.8, 44.5, 31.4, 30.1, 21.6 ppm; IR (thin film) 3478, 2924, 1598, 1503, 1485, 1160, 1089, 1038, 947, 808, 746, 665, 551 (cm^{-1}); HRMS (ESI) m/z: calcd for $[C_{25}H_{24}NO_4S]^+$ 434.1421; found 434.1429.

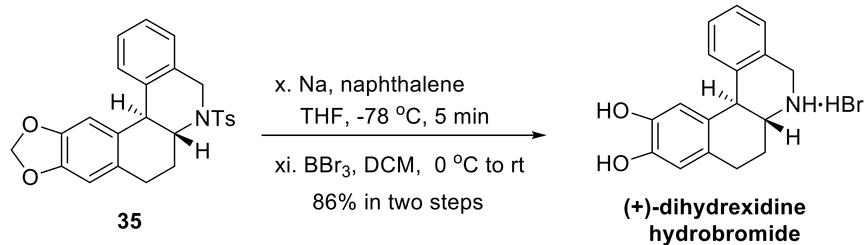

35 **(+)-dihydrexidine hydrobromide**

To a solution of compound **35** (43.4 mg, 0.1 mmol, 1.0 equiv) in anhydrous THF (5 mL) at -78 °C under inert atmosphere was added dropwise a pre-made Na-naphthalene solution (1.0 M in THF, 0.6 mL, 0.6 mmol, 6.0 equiv). The resulting mixture was stirred at -78 °C for five minutes, which was allowed to warm to room temperature. The resulting mixture was filtered through a short pad of silica gel (DCM eluent, 20 mL) to remove excess naphthalene. The eluent (DCM: MeOH = 5: 1, 30 mL) was concentrated to afford a crude colorless oil, which was used directly in next step without further purification.

To the above crude colorless oil in DCM (5 mL) at 0 °C was added dropwise BBr_3 (1.0 M in DCM, 0.5 mL, 0.5 mmol, 5.0 equiv). The resulting solution was allowed to warm to room temperature and stirred overnight. All the volatiles were directly removed on a rotary evaporator, the resulting crude oil was triturated with diethyl ether (Et_2O) and isolated by filtration to afford 29.9 mg (86%) of (+)-dihydrexidine·HBr as a pale yellow solid, the ^{1}H NMR of which is identical with that reported in literature.[35] ^{1}H NMR (400 MHz, CD_3OD) δ 7.52 (d, J = 7.2 Hz, 1H), 7.45-7.36 (m, 3H), 6.81 (s, 1H), 6.67 (s, 1H), 4.46 (s, 2H), 4.22 (d, J = 11.2 Hz, 1H), 3.09-3.02 (m, 1H), 2.93-2.87 (m, 1H), 2.84-2.78 (m, 1H), 2.34-2.27 (m, 1H), 2.01-1.94 (m, 1H) ppm. $[\alpha]_D^{27}$ = +29.0 (c 0.20, EtOH). {lit[35] $[\alpha]_D^{28}$ = +37.0 (c 0.20, EtOH)}.

2.6 Reactions of aryne precursors with *n*-$BuNH_2$ and *t*-$BuNH_2$

Reaction of 1e with *n*-$BuNH_2$ and *t*-$BuNH_2$:

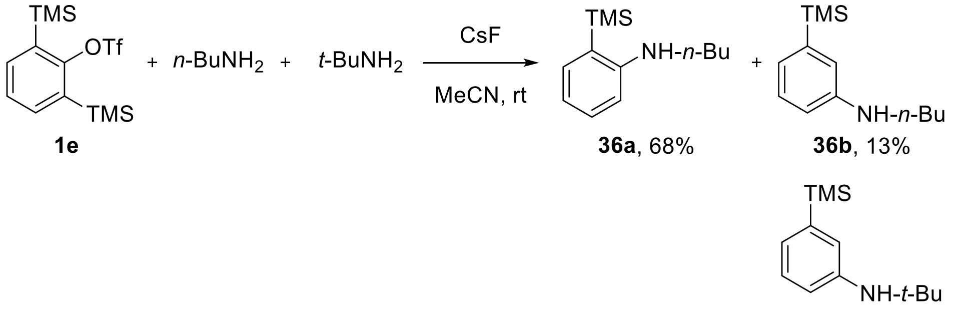

A mixture of aryne precursor **1e** (185.3 mg, 0.5 mmol, 1.0 equiv), *n*-$BuNH_2$ (109.7 mg, 1.5 mmol, 3.0 equiv), *t*-$BuNH_2$ (109.7 mg, 1.5 mmol, 3.0 equiv), and CsF (228 mg, 1.5 mmol, 3.0 equiv) in anhydrous MeCN (5.0 mL) under inert atmosphere was stirred at room temperature overnight. The resulting mixture was filtered through a short pad of silica gel (EtOAc eluent, 20 mL). All the volatiles were removed on rotary evaporator. Flash column chromatography (pet ether: EtOAc = 100: 1 to 20: 1) afforded 75.3 mg (68%) of compound **36a** as a colorless oil, 14.4 mg (13%) of compound **36b** as a colorless oil, and 7.7 mg (7%) of compound **36c** as a colorless oil.

Compound 36a: ^{1}H NMR (400 MHz, $CDCl_3$) δ 7.33-7.26 (m, 2H), 6.73 (td, J_1 = 7.2, J_2 = 0.4 Hz, 1H), 6.62 (d, J = 8.4 Hz, 1H), 3.76 (brs, 1H), 3.15 (t, J = 6.8 Hz, 2H), 1.71-1.62 (m, 2H), 1.54-1.43 (m, 2H), 1.00 (t, J = 7.6 Hz, 3H), 0.35 (s, 9H) ppm; ^{13}C NMR (100 MHz, $CDCl_3$) δ 153.3, 135.2, 131.0, 122.5, 117.0, 109.9, 44.0, 31.9, 20.6, 14.1, -0.3 ppm; IR (thin film) 3437, 2958, 2929, 2868, 1590, 1571, 1504, 1442, 1404, 1310, 1248, 1118, 987, 842, 753, 719, 690, 620, 540, 455 (cm^{-1}); HRMS (ESI) m/z: calcd for $[C_{13}H_{24}NSi]^+$ 222.1673; found 222.1678.

Compound 36b: ^{1}H NMR (400 MHz, $CDCl_3$) δ 7.18 (t, J = 7.6 Hz, 1H), 6.85 (d, J = 7.2 Hz, 1H), 6.75 (d, J = 2.4 Hz, 1H), 6.60 (ddd, J_1 = 8.0, J_2 = 2.8, J_3 = 1.2 Hz, 1H), 3.58 (brs, 1H), 3.12 (t, J = 6.8 Hz, 2H), 1.65-1.57 (m, 2H), 1.49-1.38 (m, 2H), 0.97 (t, J = 7.2 Hz, 3H), 0.24 (s, 9H) ppm; ^{13}C NMR (100 MHz, $CDCl_3$) δ 148.1, 141.4, 128.9, 122.3, 118.1, 113.1, 43.9, 32.0, 20.5, 14.1, -0.9 ppm; IR (thin film) 3417,

2956, 2929, 2346, 1592, 1575, 1476, 1403, 1315, 1247, 1116, 990, 837, 752, 691, 620, 542, 472 (cm^{-1}); HRMS (ESI) m/z: calcd for $[C_{13}H_{24}NSi]^+$ 222.1673; found 222.1680.

Compound 36c: ^{1}H NMR (400 MHz, $CDCl_3$) δ 7.17 (t, J = 7.6 Hz,1H), 6.92 (d, J = 7.2 Hz, 1H), 6.88 (d, J = 2.4 Hz, 1H), 6.78 (ddd, J_1 = 8.0, J_2 = 2.4, J_3 = 1.2 Hz, 1H), 3.40 (brs, 1H), 1.35 (s, 9H), 0.25 (s, 9H) ppm; ^{13}C NMR (100 MHz, $CDCl_3$) δ 146.2, 141.2, 128.5, 123.5, 123.1, 118.0, 51.7, 30.3, -0.9 ppm; IR (thin film) 3412, 2957, 2899, 1591, 1574, 1510, 1476, 1391, 1364, 1316, 1121, 997, 871, 836, 752, 690, 620, 542, 479 (cm^{-1}); HRMS (ESI) m/z: calcd for $[C_{13}H_{24}NSi]^+$ 222.1673; found 222.1677.

Reaction of 1c with *n*-BuNH₂ and *t*-BuNH₂:

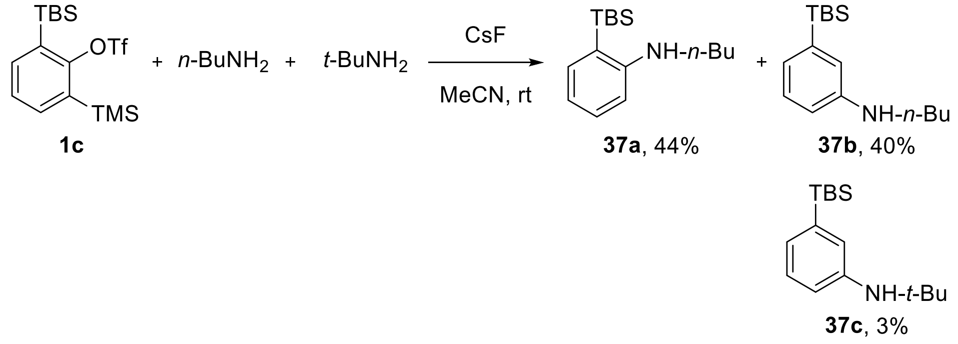

A mixture of aryne precursor **1c** (206.3 mg, 0.5 mmol, 1.0 equiv), n-BuNH$_2$ (109.7 mg, 1.5 mmol, 3.0 equiv), t-BuNH$_2$ (109.7 mg, 1.5 mmol, 3.0 equiv), and CsF (228 mg, 1.5 mmol, 3.0 equiv) in anhydrous MeCN (5.0 mL) under inert atmosphere was stirred at room temperature overnight. The resulting mixture was filtered through a short pad of silica gel (EtOAc eluent, 20 mL). All the volatiles were removed on rotary evaporator. Flash column chromatography (pet ether: EtOAc = 100: 1 to 20: 1) afforded 58.0 mg (44%) of compound **37a** as a colorless oil, 52.7 mg (40%) of compound **37b** as a colorless oil, and 4.0 mg (3%) of compound **37c** as a white solid.

Compound 37a: ^{1}H NMR (400 MHz, $CDCl_3$) δ 7.28-7.25 (m, 1H), 7.25-7.22 (m, 1H), 6.68 (td, J_1 = 7.6, J_2 = 1.2 Hz, 1H), 6.57 (d, J = 8.0 Hz, 1H), 3.88 (brs, 1H), 3.12-3.06 (m, 2H), 1.66-1.58 (m, 2H), 1.50-1.40 (m, 2H), 0.97 (t, J = 7.2 Hz, 3H), 0.92 (s, 9H), 0.35 (s, 6H) ppm; ^{13}C NMR (100 MHz, $CDCl_3$) δ 154.0, 137.0, 130.8, 119.8, 116.5, 109.9, 44.0, 31.9, 27.1, 20.7, 18.4, 14.1, -3.7 ppm; IR (thin film) 3453, 2956, 2927, 2855, 2350, 1589, 1570, 1400, 1385, 1310, 1258, 1114, 1004, 832, 810, 769, 750, 541, 461 (cm^{-1}); HRMS (ESI) m/z: calcd for $[C_{16}H_{30}NSi]^+$ 264.2142; found 264.2146.

Compound 37b: ^{1}H NMR (400 MHz, $CDCl_3$) δ 7.17 (t, J = 7.6 Hz, 1H), 6.84 (dd, J_1 = 6.8, J_2 = 0.4 Hz, 1H), 6.75 (d, J = 2.0 Hz, 1H), 6.61 (dd, J_1 = 8.0, J_2 = 2.4 Hz, 1H), 3.56 (brs, 1H), 3.12 (t, J = 7.2 Hz, 2H), 1.67-1.57 (m, 2H), 1.49-1.41 (m, 2H), 0.97 (t, J = 7.2 Hz, 3H), 0.89 (s, 9H), 0.25 (s, 6H) ppm; ^{13}C NMR (100 MHz, $CDCl_3$) δ 147.7, 138.7, 128.5, 123.7, 119.4, 113.1, 43.9, 32.0, 26.8, 20.5, 17.1, 14.2, -5.9 ppm; IR (thin film) 3417, 2956, 2855, 1592, 1576, 1504, 1471, 1404, 1246, 1117, 1008, 990, 832, 807, 769, 699, 662, 542, 442 (cm^{-1}); HRMS (ESI) m/z: calcd for $[C_{16}H_{30}NSi]^+$ 264.2142; found 264.2145.

Compound 37c: Mp: 30-32 °C; ^{1}H NMR (400 MHz, $CDCl_3$) δ 7.16 (t, J = 7.6 Hz, 1H), 6.93 (d, J = 7.2 Hz, 1H), 6.90 (d, J = 2.4 Hz, 1H), 6.78 (ddd, J_1 = 8.0, J_2 = 2.4, J_3 = 0.8 Hz, 1H), 3.20 (brs, 1H), 1.34 (s, 9H), 0.89 (s, 9H), 0.26 (s, 6H) ppm; ^{13}C NMR (100 MHz, $CDCl_3$) δ 146.0, 138.4, 128.1, 125.1, 124.4, 118.5, 51.8, 30.3, 26.8, 17.2, -6.0 ppm; IR (thin film) 3399, 2954, 2880, 1591, 1514, 1471, 1390, 1314, 1247, 1224, 1122, 1007, 832, 808, 771, 700, 662, 575, 439 (cm^{-1}); HRMS (ESI) m/z: calcd for $[C_{16}H_{30}NSi]^+$ 264.2142; found 264.2147.

Reaction of 1b with *n*-BuNH₂ and *t*-BuNH₂:

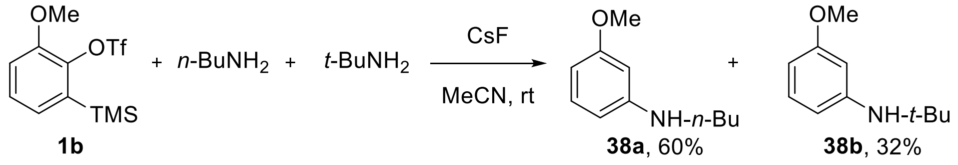

A mixture of aryne precursor **1b** (164.2 mg, 0.5 mmol, 1.0 equiv), *n*-$BuNH_2$ (109.7 mg, 1.5 mmol, 3.0 equiv), *t*-$BuNH_2$ (109.7 mg, 1.5 mmol, 3.0 equiv), and CsF (228 mg, 1.5 mmol, 3.0 equiv) in anhydrous MeCN (5.0 mL) under inert atmosphere was stirred at room temperature overnight. The resulting mixture was filtered through a short pad of silica gel (EtOAc eluent, 20 mL). All the volatiles were removed on rotary evaporator. Flash column chromatography (pet ether: EtOAc = 50: 1 to 20: 1) afforded 53.8 mg (60%) of compound **38a** as a colorless oil, and 28.7 mg (32%) of compound **38b** as a colorless oil.

Compound 38a: The ^{1}H NMR of **38a** is identical with that reported in literature.[36] ^{1}H NMR (400 MHz, $CDCl_3$) δ 7.08 (t, *J* = 8.0 Hz, 1H), 6.28-6.24 (m, 1H), 6.24-6.21 (m, 1H), 6.17 (t, *J* = 2.4 Hz, 1H), 3.78 (s, 3H), 3.63 (brs, 1H), 3.10 (t, *J* = 7.2 Hz, 2H), 1.64-1.56 (m, 2H), 1.48-1.38 (m, 2H), 0.96 (t, *J* = 7.2 Hz, 3H) ppm.

Compound 38b: The ^{1}H NMR of **38b** is identical with that reported in literature.[37] ^{1}H NMR (400 MHz, $CDCl_3$) δ 7.09-7.02 (m, 1H), 6.37-6.28 (m, 3H), 3.77 (s, 3H), 2.87 (brs, 1H), 1.35 (s, 9H) ppm.

2.7 Crystal data and structure refinements for 10b and 34:

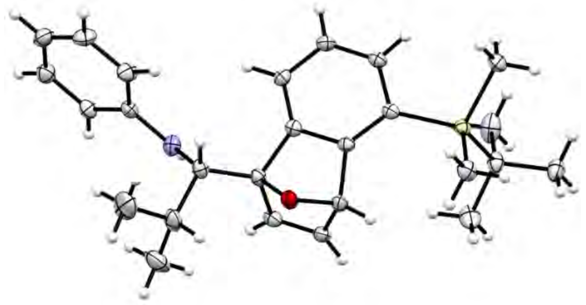

Supplementary Figure 2. Thermal ellipsoid plot (30% probability) of X-ray structure of **10b**. Hydrogen, white; Carbon, gray; oxygen, red; silicon, yellow; nitrogen, purple. CCDC of **10b**: 2234236.

Supplementary Table 3. Crystal data and structure refinement for 10b.

Empirical formula	$C_{26}NOSiH_{35}$
Formula weight	405.64
Temperature/K	298.15
Crystal system	triclinic
Space group	P-1
a/Å	12.1108(6)
b/Å	14.4696(10)
c/Å	16.0311(11)
α/°	112.388(6)
β/°	90.239(5)
γ/°	113.638(6)
Volume/Å^3	2338.4(3)
Z	2
ρ_{calc}g/cm^3	1.152
μ/mm^{-1}	0.117
F(000)	880.0
Crystal size/mm^3	0.15 × 0.15 × 0.1
Radiation	MoKα (λ = 0.71073)
2Θ range for data collection/°	6.646 to 58.138
Index ranges	$-16 \leq h \leq 15$, $-18 \leq k \leq 19$, $-21 \leq l \leq 20$
Reflections collected	20539

Independent reflections	10706 [R_{int} = 0.0317, R_{sigma} = 0.0564]
Data/restraints/parameters	10706/0/550
Goodness-of-fit on F^2	0.959
Final R indexes [I>=2σ (I)]	R_1 = 0.0633, wR_2 = 0.1717
Final R indexes [all data]	R_1 = 0.0993, wR_2 = 0.2027

Supplementary Table 4. Bond Lengths for 10b.

Atom	Atom	Length/Å	Atom	Atom	Length/Å
C1	C2	1.374(4)	C28	C29	1.363(5)
C1	C6	1.386(4)	C29	C30	1.372(4)
C2	C3	1.370(4)	C30	C31	1.377(4)
C3	C4	1.371(5)	C31	C32	1.387(4)
C4	C5	1.373(5)	C32	N2	1.398(4)
C5	C6	1.388(4)	C36	C37	1.519(4)
C6	N1	1.392(4)	C36	N2	1.460(3)
C7	C8	1.490(5)	C36	C35A	1.64(2)
C8	C9	1.513(5)	C36	C35B	1.46(2)
C8	C10	1.544(4)	C37	C38	1.534(4)
C10	C11	1.514(4)	C37	C46	1.539(3)
C10	N1	1.454(4)	C37	O2	1.464(3)
C11	C12	1.533(4)	C38	C39	1.316(4)
C11	C20	1.535(4)	C39	C40	1.527(4)
C11	O1	1.461(3)	C40	C41	1.522(3)
C12	C13	1.312(4)	C40	O2	1.451(3)
C13	C14	1.519(4)	C41	C42	1.393(3)
C14	C15	1.535(4)	C41	C46	1.398(4)
C14	O1	1.445(3)	C42	C43	1.416(3)
C15	C16	1.386(3)	C42	Si2	1.884(3)
C15	C20	1.401(4)	C43	C44	1.370(4)
C16	C17	1.420(4)	C44	C45	1.397(4)
C16	Si1	1.879(3)	C45	C46	1.371(3)
C17	C18	1.367(4)	C47	Si2	1.863(3)
C18	C19	1.393(4)	C48	Si2	1.865(3)
C19	C20	1.371(4)	C49	C50	1.533(4)
C21	Si1	1.865(3)	C50	C51	1.537(4)
C22	Si1	1.867(3)	C50	C52	1.525(4)
C23	C24	1.527(5)	C50	Si2	1.894(3)
C24	C25	1.527(5)	C33A	C35A	1.38(3)
C24	C26	1.539(4)	C34A	C35A	1.52(2)
C24	Si1	1.891(3)	C33B	C35B	1.41(2)
C27	C28	1.374(4)	C34B	C35B	1.53(2)
C27	C32	1.390(4)			

Supplementary Table 5. Bond Angles for 10b.

Atom	Atom	Atom	Angle/°	Atom	Atom	Atom	Angle/°
C2	C1	C6	121.2(3)	C29	C30	C31	121.3(3)
C3	C2	C1	120.8(3)	C30	C31	C32	120.6(3)
C2	C3	C4	118.5(3)	C27	C32	N2	119.7(2)
C5	C4	C3	121.3(3)	C31	C32	C27	117.7(3)
C4	C5	C6	120.6(3)	C31	C32	N2	122.6(3)
C1	C6	C5	117.5(3)	C37	C36	C35A	109.9(8)
C1	C6	N1	119.7(2)	N2	C36	C37	107.0(2)

Supplementary Table 5. Bond Angles for 10b.

Atom	Atom	Atom	Angle/°	Atom	Atom	Atom	Angle/°
C5	C6	N1	122.7(3)	N2	C36	C35A	114.2(8)
C7	C8	C9	110.1(4)	C35B	C36	C37	119.1(10)
C7	C8	C10	110.9(3)	C35B	C36	N2	110.5(7)
C9	C8	C10	114.7(3)	C36	C37	C38	119.9(2)
C11	C10	C8	113.5(2)	C36	C37	C46	118.6(2)
N1	C10	C8	113.7(2)	C38	C37	C46	104.8(2)
N1	C10	C11	108.4(2)	O2	C37	C36	112.1(2)
C10	C11	C12	119.9(2)	O2	C37	C38	99.5(2)
C10	C11	C20	118.7(2)	O2	C37	C46	98.34(19)
C12	C11	C20	105.0(2)	C39	C38	C37	106.6(2)
O1	C11	C10	111.0(2)	C38	C39	C40	105.6(3)
O1	C11	C12	99.8(2)	C41	C40	C39	106.6(2)
O1	C11	C20	98.77(19)	O2	C40	C39	100.3(2)
C13	C12	C11	106.0(2)	O2	C40	C41	100.3(2)
C12	C13	C14	106.2(2)	C42	C41	C40	133.4(2)
C13	C14	C15	106.3(2)	C42	C41	C46	122.9(2)
O1	C14	C13	100.4(2)	C46	C41	C40	103.6(2)
O1	C14	C15	100.0(2)	C41	C42	C43	114.0(2)
C16	C15	C14	133.5(2)	C41	C42	Si2	126.97(19)
C16	C15	C20	123.2(2)	C43	C42	Si2	119.02(19)
C20	C15	C14	103.3(2)	C44	C43	C42	123.4(2)
C15	C16	C17	113.8(2)	C43	C44	C45	121.0(2)
C15	C16	Si1	126.8(2)	C46	C45	C44	117.2(3)
C17	C16	Si1	119.35(19)	C41	C46	C37	105.5(2)
C18	C17	C16	123.5(3)	C45	C46	C37	133.1(2)
C17	C18	C19	121.1(3)	C45	C46	C41	121.4(2)
C20	C19	C18	117.2(3)	C49	C50	C51	109.2(3)
C15	C20	C11	105.3(2)	C49	C50	Si2	110.4(2)
C19	C20	C11	133.4(2)	C51	C50	Si2	109.7(2)
C19	C20	C15	121.2(2)	C52	C50	C49	108.9(3)
C23	C24	C26	108.1(3)	C52	C50	C51	108.1(3)
C23	C24	Si1	110.5(2)	C52	C50	Si2	110.5(2)
C25	C24	C23	110.2(3)	C32	N2	C36	121.0(2)
C25	C24	C26	108.9(3)	C40	O2	C37	96.00(18)
C25	C24	Si1	109.8(2)	C42	Si2	C50	110.20(11)
C26	C24	Si1	109.3(2)	C47	Si2	C42	110.87(14)
C6	N1	C10	122.7(2)	C47	Si2	C48	108.20(15)
C14	O1	C11	95.95(19)	C47	Si2	C50	109.95(15)
C16	Si1	C24	110.16(12)	C48	Si2	C42	107.71(13)
C21	Si1	C16	111.06(14)	C48	Si2	C50	109.86(14)
C21	Si1	C22	107.98(15)	C33A	C35A	C36	115.0(14)
C21	Si1	C24	110.13(15)	C33A	C35A	C34A	115.5(15)
C22	Si1	C16	107.40(13)	C34A	C35A	C36	104.7(15)
C22	Si1	C24	110.05(15)	C36	C35B	C34B	114.7(13)
C28	C27	C32	120.4(3)	C33B	C35B	C36	128.1(15)
C29	C28	C27	121.7(3)	C33B	C35B	C34B	115.7(16)
C28	C29	C30	118.2(3)				

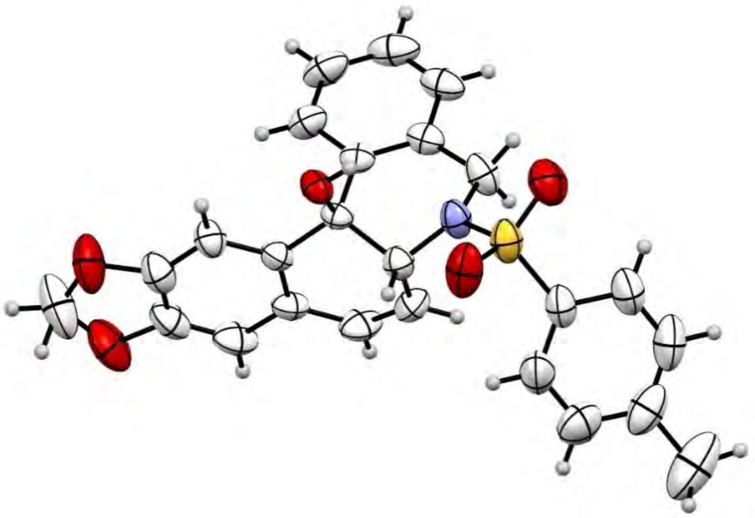

Supplementary Figure 3. Thermal ellipsoid plot (30% probability) of X-ray structure of **34**. Hydrogen, white; Carbon, gray; oxygen, red; sulphur, yellow; nitrogen, purple. CCDC of **34**: 2288207.

Supplementary Table 6. The crystal parameters of complex 34.

Empirical formula	$C_{25}H_{21}NO_5S$
Formula weight	447.49
T/ K	298
Crystal system	triclinic
Space group	P1
a/Å	8.3990(4)
b/Å	11.1334(7)
c/Å	13.4100(6)
α/°	65.767(5)
β/°	80.002(4)
γ/°	75.115(5)
Volume/Å^3	1101.78(11)
Z	1
ρ_{calc}g/cm^3	1.349
μ/mm^{-1}	0.184
F(000)	468.0
Crystal size/mm^3	1.0 × 0.8 × 0.6
Radiation	Mo Kα (λ = 0.71073)
2Θ range for data collection/°	4.1 to 58.164
Index ranges	-11 ≤ h ≤ 11, -14 ≤ k ≤ 15, -18 ≤ l ≤ 17
Reflections collected	16014
Independent reflections	9952 [R_{int} = 0.0382, R_{sigma} = 0.0720]
Data/restraints/parameters	9952/3/585
Goodness-of-fit on F^2	0.934
Data/restraints/parameters	9952/3/585
Final R indexes [I>=2σ (I)]	R_1 = 0.0558, wR_2 = 0.1429
Final R indexes [all data]	R_1 = 0.0903, wR_2 = 0.1739
Largest diff. peak/hole / e Å^{-3}	0.19/-0.28

Flack parameter	-0.03(4)

Supplementary Table 7. Bond Lengths for 34.

Atom	Atom	Length/Å	Atom	Atom	Length/Å
S2	O10	1.429(5)	C42	C43	1.501(8)
S2	O9	1.426(4)	C5	C8	1.446(9)
S2	N2	1.631(5)	C5	C4	1.414(8)
S2	C44	1.767(6)	C31	C32	1.398(8)
S1	O4	1.427(4)	C31	C33	1.468(9)
S1	O5	1.432(5)	C27	C28	1.360(9)
S1	N1	1.624(5)	C27	C32	1.371(10)
S1	C19	1.760(7)	C38	C39	1.383(8)
O3	C11	1.425(6)	C17	C12	1.395(7)
O8	C36	1.437(6)	C17	C18	1.494(8)
O1	C2	1.383(8)	C17	C16	1.406(8)
O1	C1	1.412(11)	C29	C28	1.381(8)
O2	C3	1.371(8)	C12	C13	1.395(8)
O2	C1	1.407(13)	C7	C2	1.364(8)
N2	C35	1.466(7)	C41	C40	1.393(9)
N2	C43	1.472(7)	C39	C40	1.359(10)
O6	C27	1.376(7)	C2	C3	1.370(10)
O6	C26	1.422(11)	C19	C24	1.395(8)
N1	C10	1.465(7)	C19	C20	1.381(10)
N1	C18	1.465(7)	C9	C8	1.330(8)
O7	C28	1.375(7)	C34	C33	1.322(8)
O7	C26	1.395(11)	C3	C4	1.378(10)
C11	C10	1.538(7)	C14	C13	1.382(9)
C11	C6	1.518(7)	C14	C15	1.390(10)
C11	C12	1.536(7)	C16	C15	1.355(10)
C10	C9	1.511(8)	C44	C45	1.371(8)
C30	C36	1.511(7)	C44	C49	1.389(9)
C30	C31	1.400(7)	C45	C46	1.387(10)
C30	C29	1.404(7)	C49	C48	1.363(10)
C35	C36	1.536(7)	C47	C48	1.392(11)
C35	C34	1.509(8)	C47	C46	1.371(11)
C37	C42	1.400(7)	C47	C50	1.499(11)
C37	C36	1.534(7)	C24	C23	1.364(11)
C37	C38	1.393(7)	C20	C21	1.372(11)
C6	C5	1.399(7)	C23	C22	1.380(13)
C6	C7	1.403(8)	C22	C21	1.393(12)
C42	C41	1.386(7)	C22	C25	1.518(12)

Supplementary Table 8. Bond Angles for 34.

Atom	Atom	Atom	Angle/˚	Atom	Atom	Atom	Angle/˚
O10	S2	N2	107.1(3)	C28	C27	O6	110.1(6)
O10	S2	C44	107.4(3)	C28	C27	C32	121.3(6)
O9	S2	O10	120.5(3)	C32	C27	O6	128.6(7)
O9	S2	N2	106.5(2)	C39	C38	C37	120.0(6)
O9	S2	C44	107.4(3)	C12	C17	C18	122.6(5)
N2	S2	C44	107.4(3)	C12	C17	C16	120.0(6)
O4	S1	O5	120.1(3)	C16	C17	C18	117.4(5)
O4	S1	N1	106.6(2)	C28	C29	C30	116.5(5)
O4	S1	C19	107.4(3)	C17	C12	C11	120.8(5)

Supplementary Table 8. Bond Angles for 34.

Atom	Atom	Atom	Angle/°	Atom	Atom	Atom	Angle/°
O5	S1	N1	106.6(3)	C17	C12	C13	118.3(5)
O5	S1	C19	107.7(3)	C13	C12	C11	120.9(5)
N1	S1	C19	108.0(3)	C2	C7	C6	116.7(6)
C2	O1	C1	104.9(7)	C42	C41	C40	121.1(6)
C3	O2	C1	105.0(6)	O7	C28	C29	126.9(6)
C35	N2	S2	119.8(3)	C27	C28	O7	110.1(5)
C35	N2	C43	115.8(4)	C27	C28	C29	123.0(6)
C43	N2	S2	119.0(4)	C40	C39	C38	120.5(6)
C27	O6	C26	104.5(6)	C7	C2	O1	127.7(6)
C10	N1	S1	121.1(4)	C7	C2	C3	122.8(6)
C18	N1	S1	118.6(4)	C3	C2	O1	109.4(6)
C18	N1	C10	115.2(4)	C24	C19	S1	120.8(5)
C28	O7	C26	105.3(6)	C20	C19	S1	120.0(5)
O3	C11	C10	109.1(4)	C20	C19	C24	119.2(7)
O3	C11	C6	107.3(4)	C8	C9	C10	120.2(5)
O3	C11	C12	110.2(4)	C39	C40	C41	119.9(5)
C6	C11	C10	110.0(4)	N2	C43	C42	111.1(4)
C6	C11	C12	110.7(4)	C33	C34	C35	119.4(5)
C12	C11	C10	109.5(4)	C27	C32	C31	118.1(6)
N1	C10	C11	106.6(4)	C34	C33	C31	122.0(5)
N1	C10	C9	114.2(5)	N1	C18	C17	110.5(5)
C9	C10	C11	110.0(5)	O2	C3	C4	127.9(6)
C31	C30	C36	118.4(4)	C2	C3	O2	110.4(7)
N2	C35	C36	106.7(4)	C13	C14	C15	120.6(6)
N2	C35	C34	114.8(4)	C15	C16	C17	121.0(6)
C34	C35	C36	109.6(4)	C3	C4	C5	117.4(6)
C42	C37	C36	119.8(4)	C45	C44	S2	120.4(5)
C38	C37	C42	120.1(5)	C45	C44	C49	120.1(6)
C38	C37	C36	120.0(5)	C49	C44	S2	119.5(5)
C5	C6	C11	117.9(5)	C14	C13	C12	120.7(6)
C5	C6	C7	121.7(5)	C44	C45	C46	119.3(7)
C7	C6	C11	120.1(5)	C48	C49	C44	119.5(6)
C37	C42	C43	122.9(5)	C48	C47	C50	122.2(8)
C41	C42	C37	118.3(5)	C46	C47	C48	117.9(7)
C41	C42	C43	118.8(5)	C46	C47	C50	119.9(8)
O8	C36	C30	107.7(4)	O7	C26	O6	109.9(6)
O8	C36	C35	109.0(4)	C16	C15	C14	119.4(6)
O8	C36	C37	108.5(4)	C49	C48	C47	121.5(7)
C30	C36	C35	108.5(4)	C23	C24	C19	119.9(7)
C30	C36	C37	113.2(4)	C21	C20	C19	120.3(7)
C37	C36	C35	109.7(4)	C24	C23	C22	121.4(7)
C6	C5	C8	119.1(5)	C23	C22	C21	118.4(7)
C6	C5	C4	119.6(6)	C23	C22	C25	120.9(9)
C4	C5	C8	121.3(5)	C21	C22	C25	120.7(10)
C30	C31	C33	118.0(5)	C47	C46	C45	121.6(7)
C32	C31	C30	120.3(5)	O2	C1	O1	109.9(7)
C32	C31	C33	121.7(5)	C20	C21	C22	120.7(8)

2.8 Density functional theory (DFT) calculations

Computational methods

All of the DFT calculations conducted in this study were carried out using the GAUSSIAN 09[38] series of programs. DFT method M06-2X[39] with a standard 6-31+G(d) basis set was used for the geometry optimizations in acetonitrile solvent.

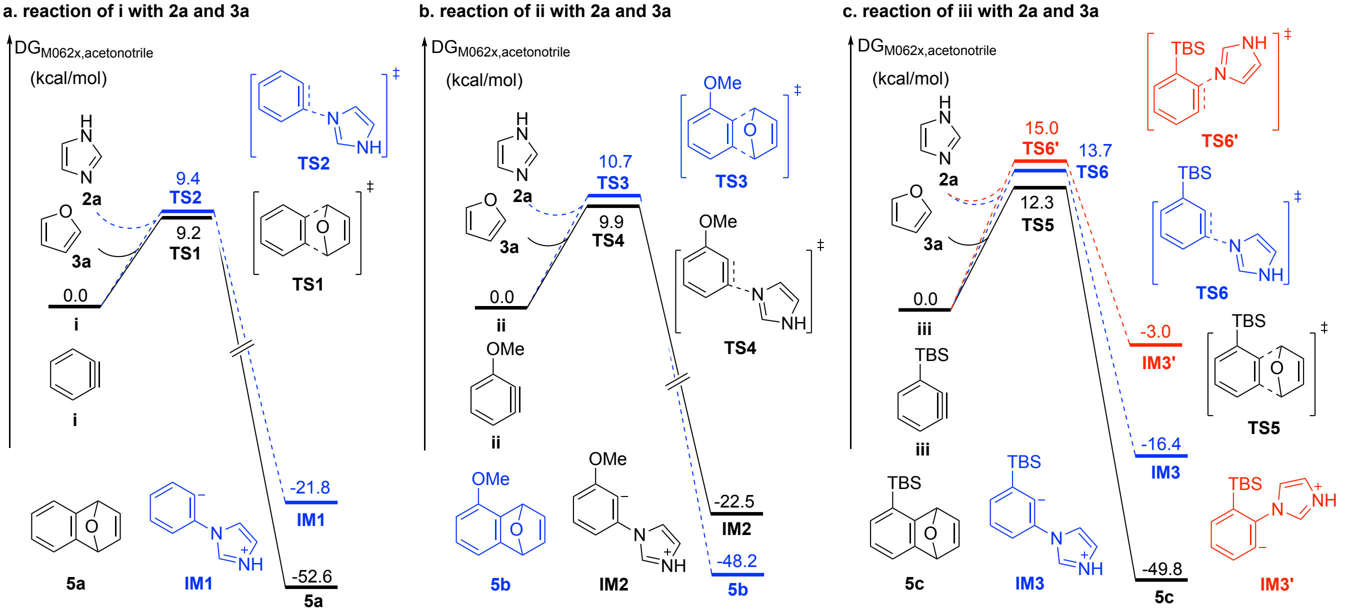

Supplementary Figure 4. a. DFT Calculations for [4+2]-cycloaddition and nucleophilic addition reactions of benzyne **i**. b. DFT Calculations for [4+2]-cycloaddition and nucleophilic addition reactions of 3-mehtoxybenzyne **ii**. c. DFT Calculations for [4+2]-cycloaddition and nucleophilic addition reactions of 3-(TBS)benzyne **iii**. The free energy values are calculated at the M06-2X/6-31+G(d) level of theory in acetonitrile with SMD model using the Gaussian 09 series of programs.

DFT calculations were employed to reveal the chemoselectivity for [4+2] cycloaddition and nucleophilic addition reactions of benzyne, 3-mehtoxybenzyne, and 3-(TBS)benzyne. As shown in Supplementary Fig 4a, when benzyne **i** is used, it can react with furan (**3a**) via transition state **TS1** with a free energy barrier of 9.2 kcal/mol. By contrast, nucleophilic addition with imidazole (**2a**) would occur via transition state **TS2** to yield zwitterionic intermediate **IM1** with a free energy barrier of 9.4 kcal/mol. Therefore, the estimated chemoselectivity is 1.4:1. As shown in Supplementary Fig 4b, the calculated free energy barrier for [4+2] cycloaddition via transition state **TS3** is 0.8 kcal/mol higher than that for nucleophilic addition via **TS4** when 3-methoxybenzyne **ii** is used, indicating that nucleophilic addition is preferred. Alternatively, when 3-(TBS)benzyne **iii** is considered, the calculated free energy barrier for [4+2] cycloaddition reaction via transition state **TS5** is 1.4 kcal/mol and 2.7 kcal/mol lower than those for nucleophilic additions via either **TS6** or **TS6'**, respectively (Supplementary Fig 4c). The calculated results suggest an excellent selectivity in favor of the [4+2] cycloaddition reaction.

3. Supplementary Figures for ^{1}H and ^{13}C NMR Spectra

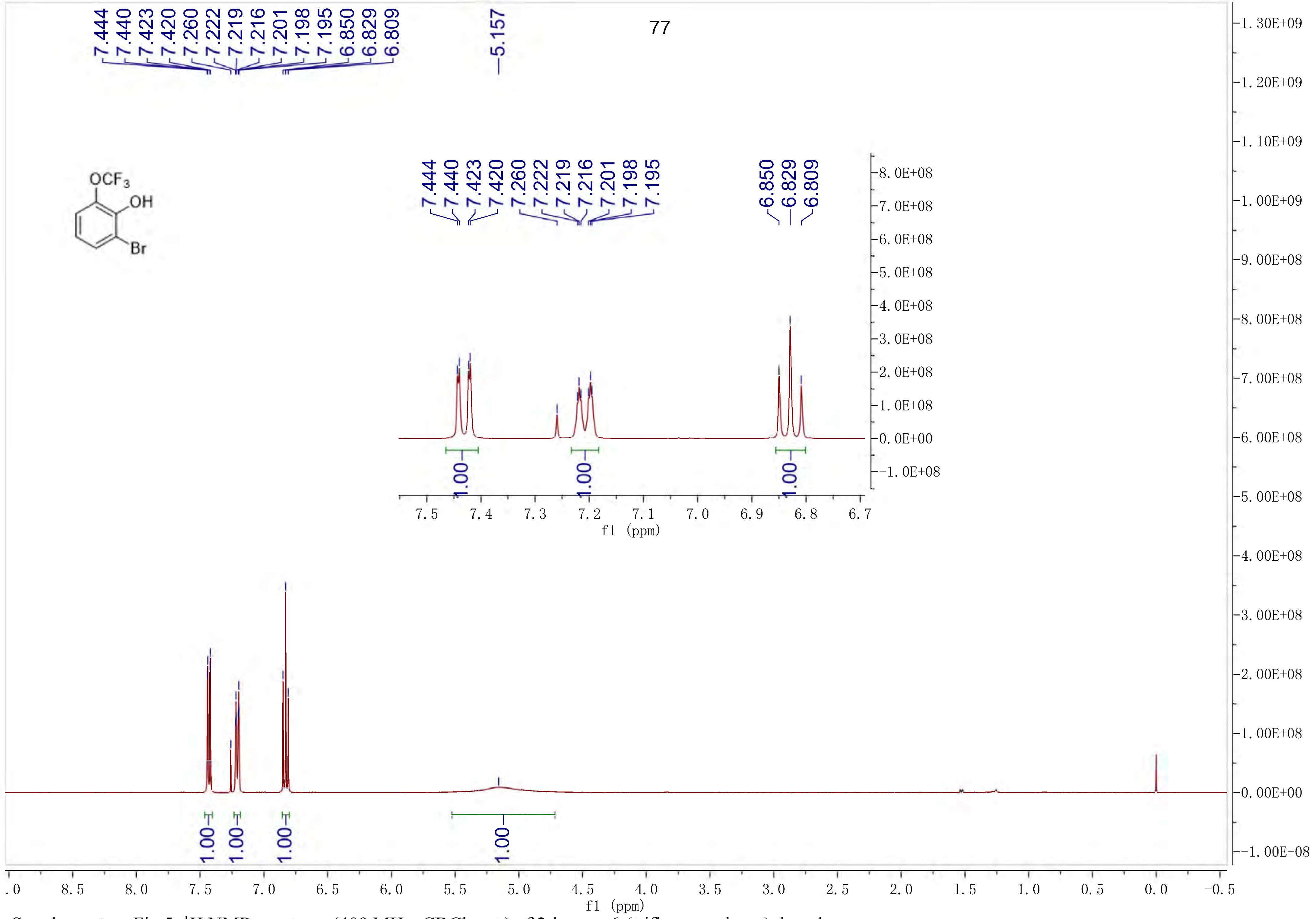

Supplementary Fig 5. ^{1}H NMR spectrum (400 MHz, $CDCl_3$, r.t.) of 2-bromo-6-(trifluoromethoxy)phenol.

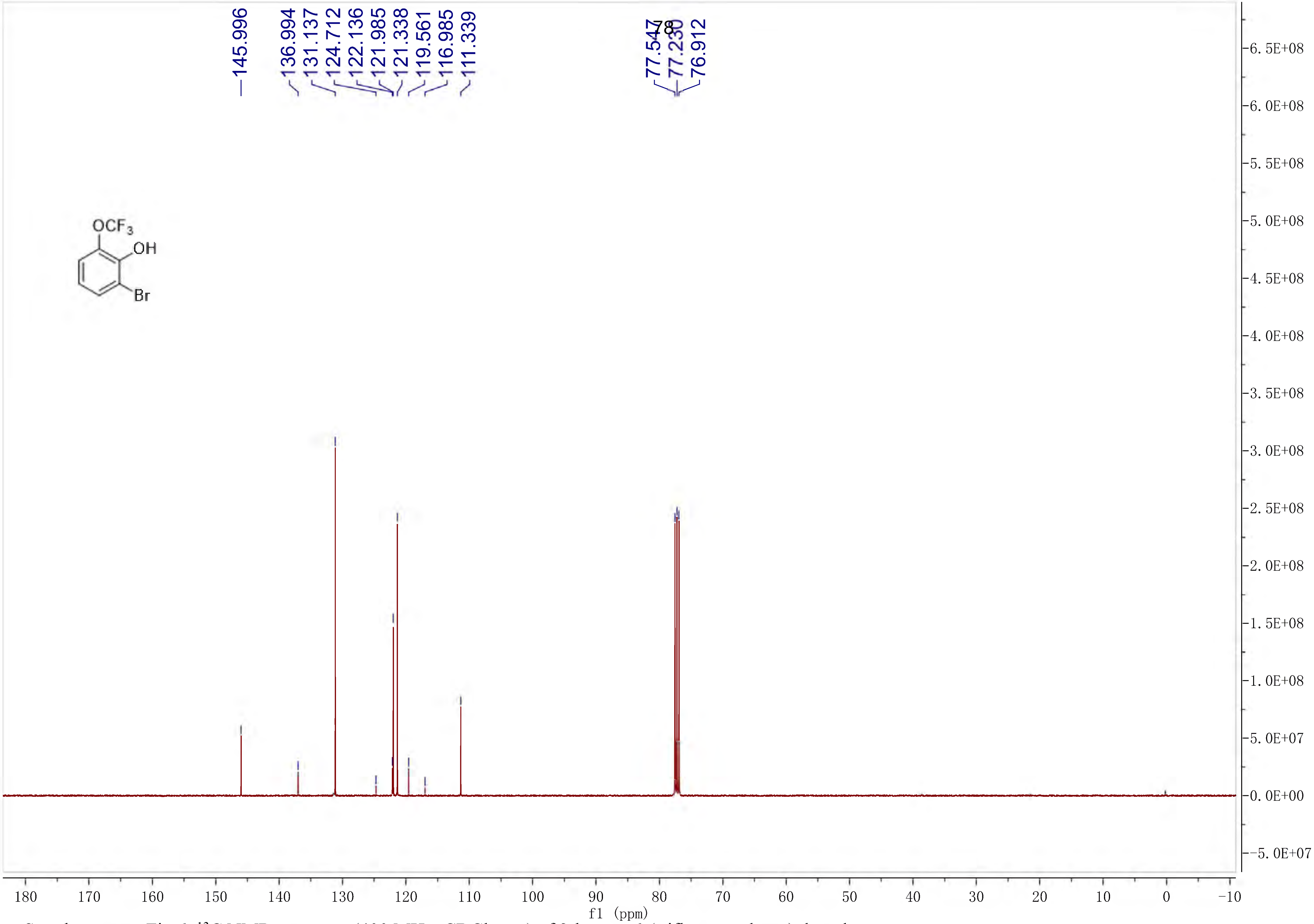

Supplementary Fig 6. ^{13}C NMR spectrum (400 MHz, $CDCl_3$, r.t.) of 2-bromo-6-(trifluoromethoxy)phenol.

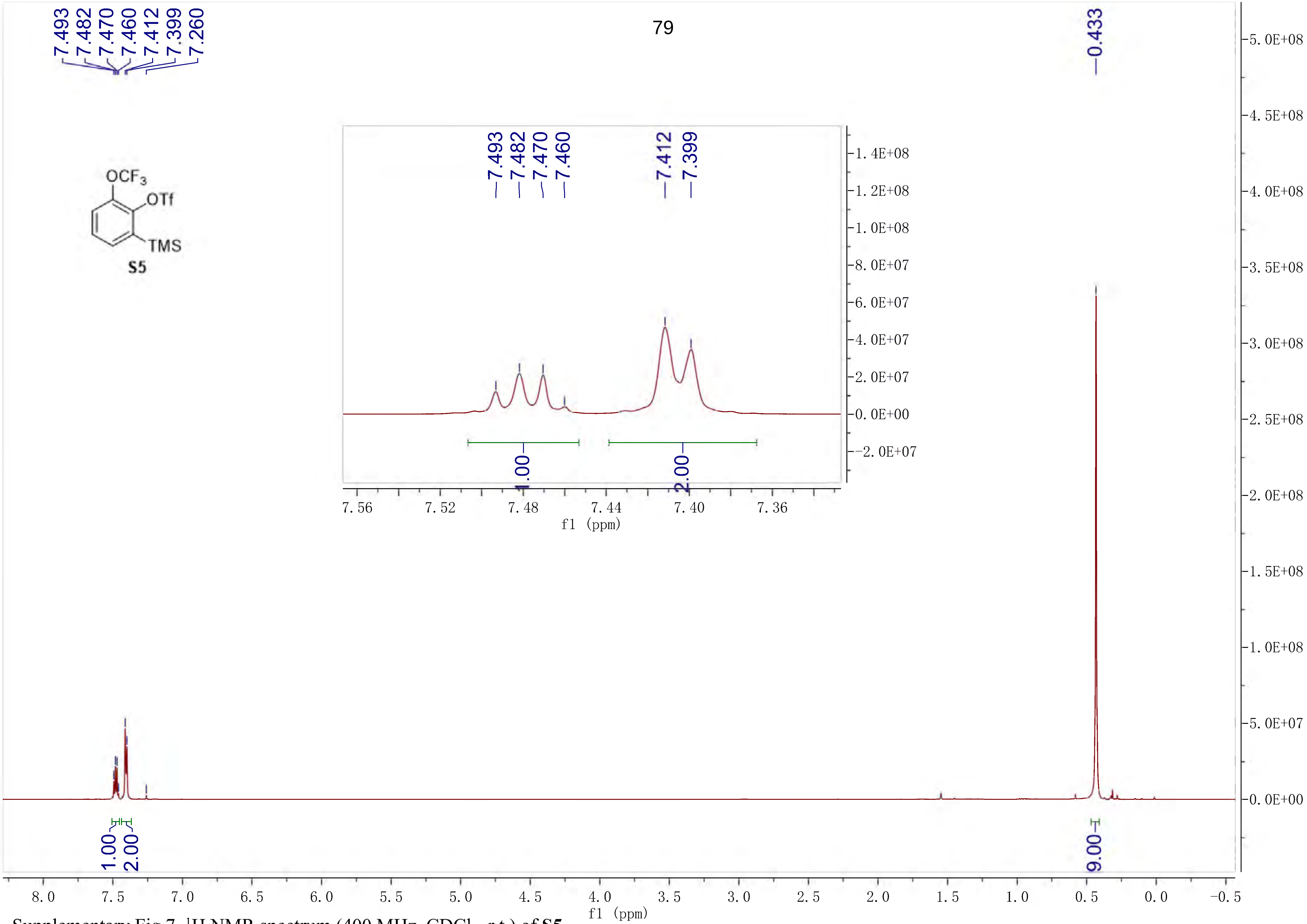

Supplementary Fig 7. ^{1}H NMR spectrum (400 MHz, $CDCl_3$, r.t.) of **S5**.

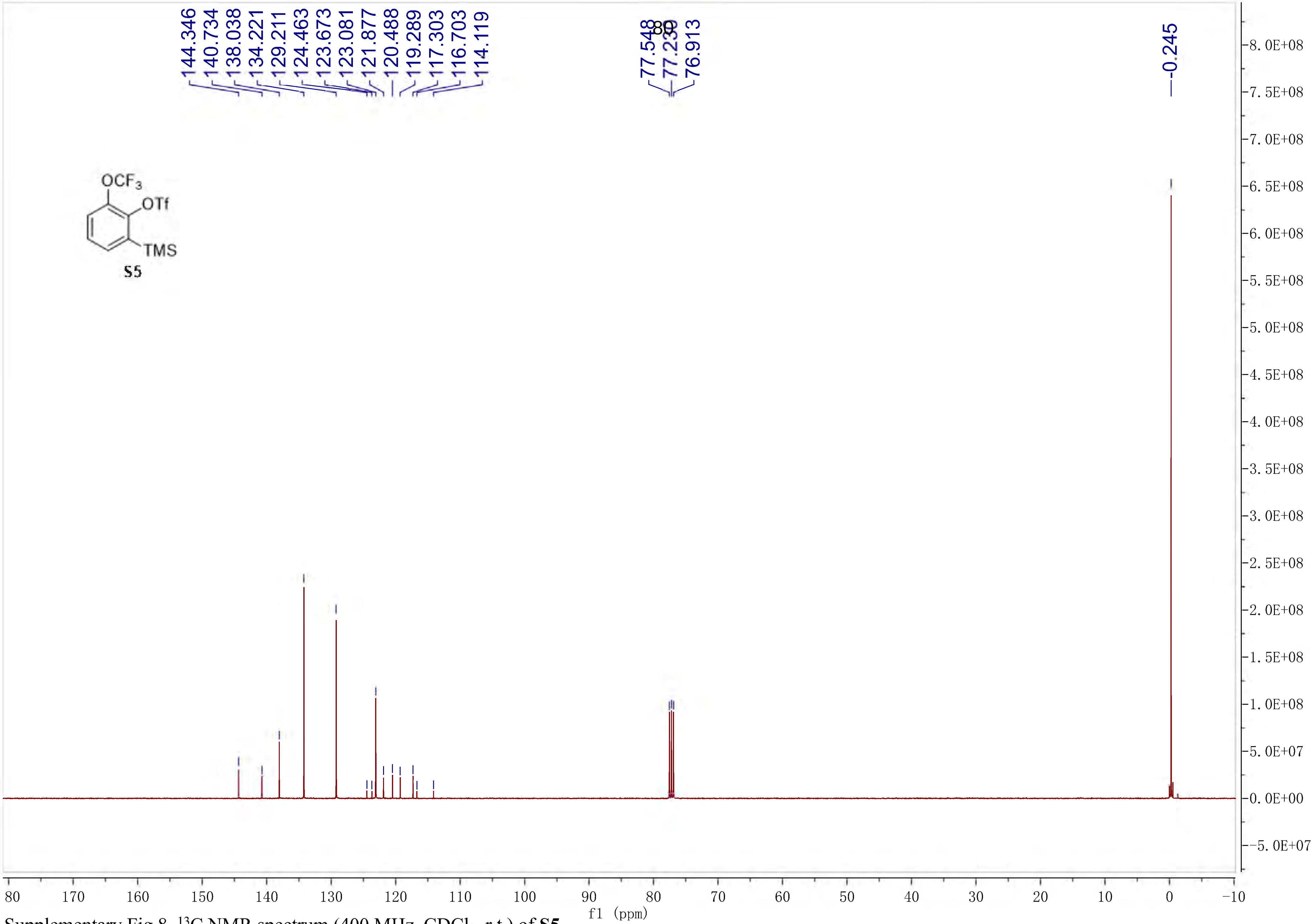

Supplementary Fig 8. ^{13}C NMR spectrum (400 MHz, $CDCl_3$, r.t.) of **S5**.

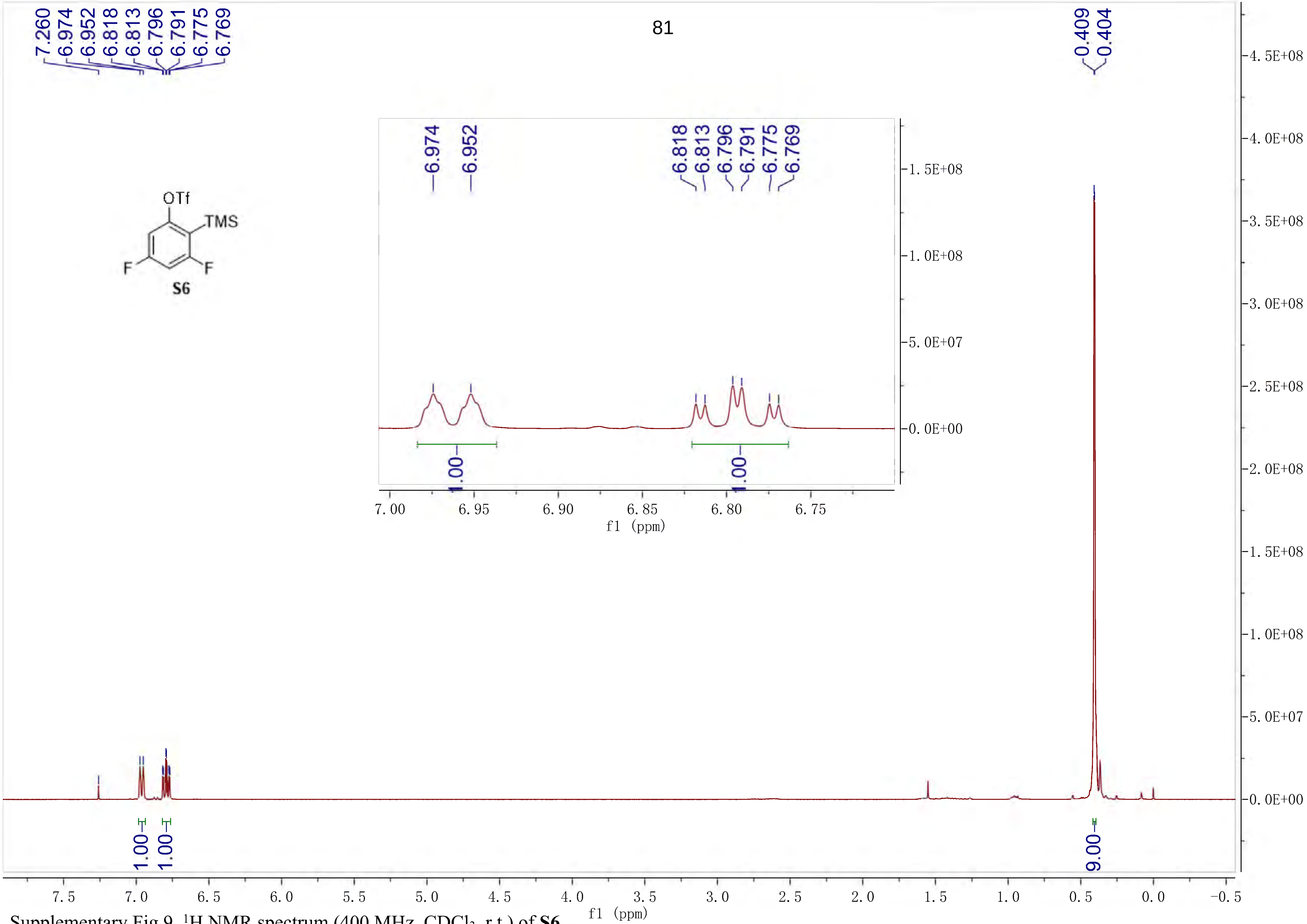

Supplementary Fig 9. ^{1}H NMR spectrum (400 MHz, $CDCl_3$, r.t.) of **S6**.

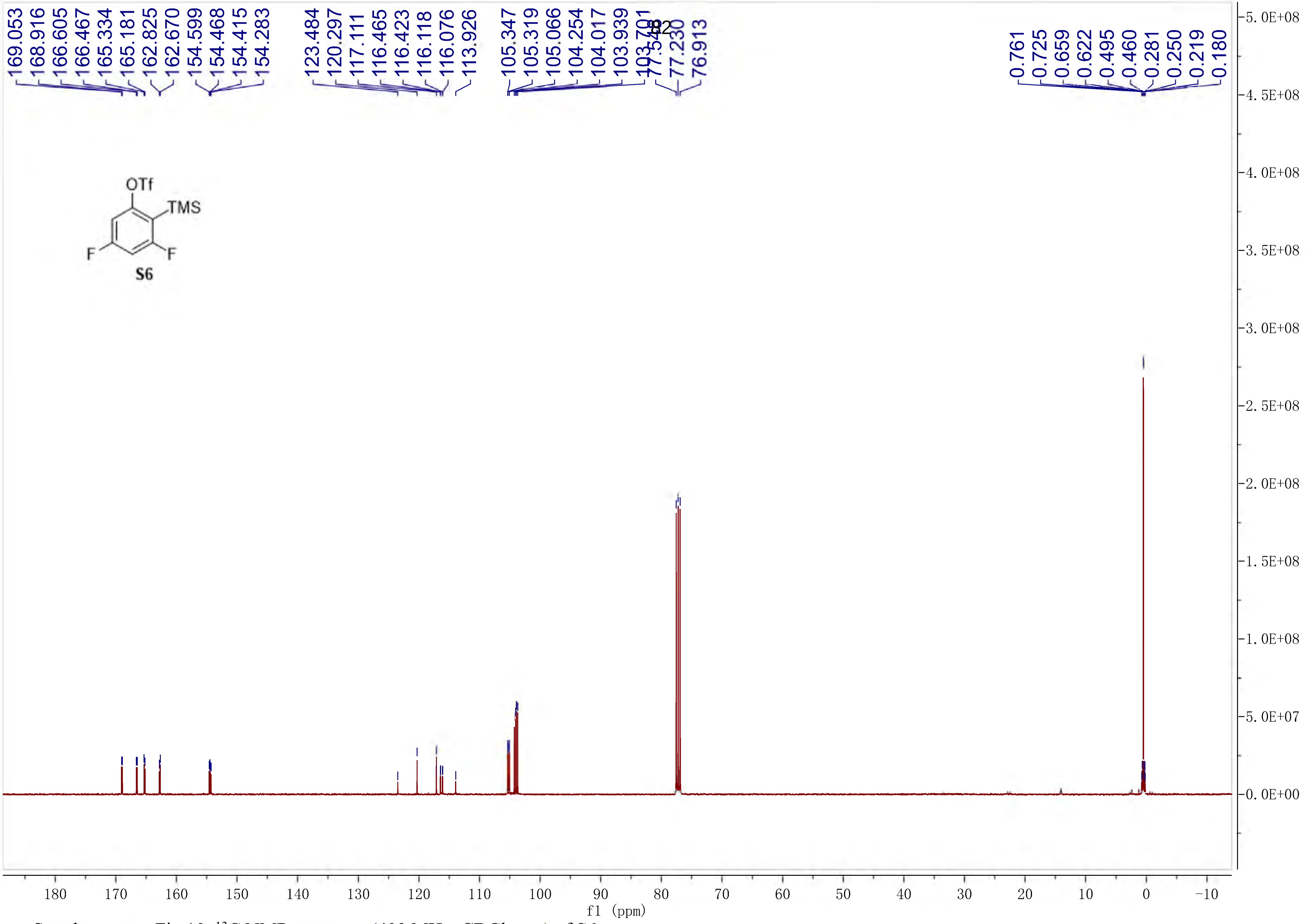

Supplementary Fig 10. ^{13}C NMR spectrum (400 MHz, $CDCl_3$, r.t.) of **S6**.

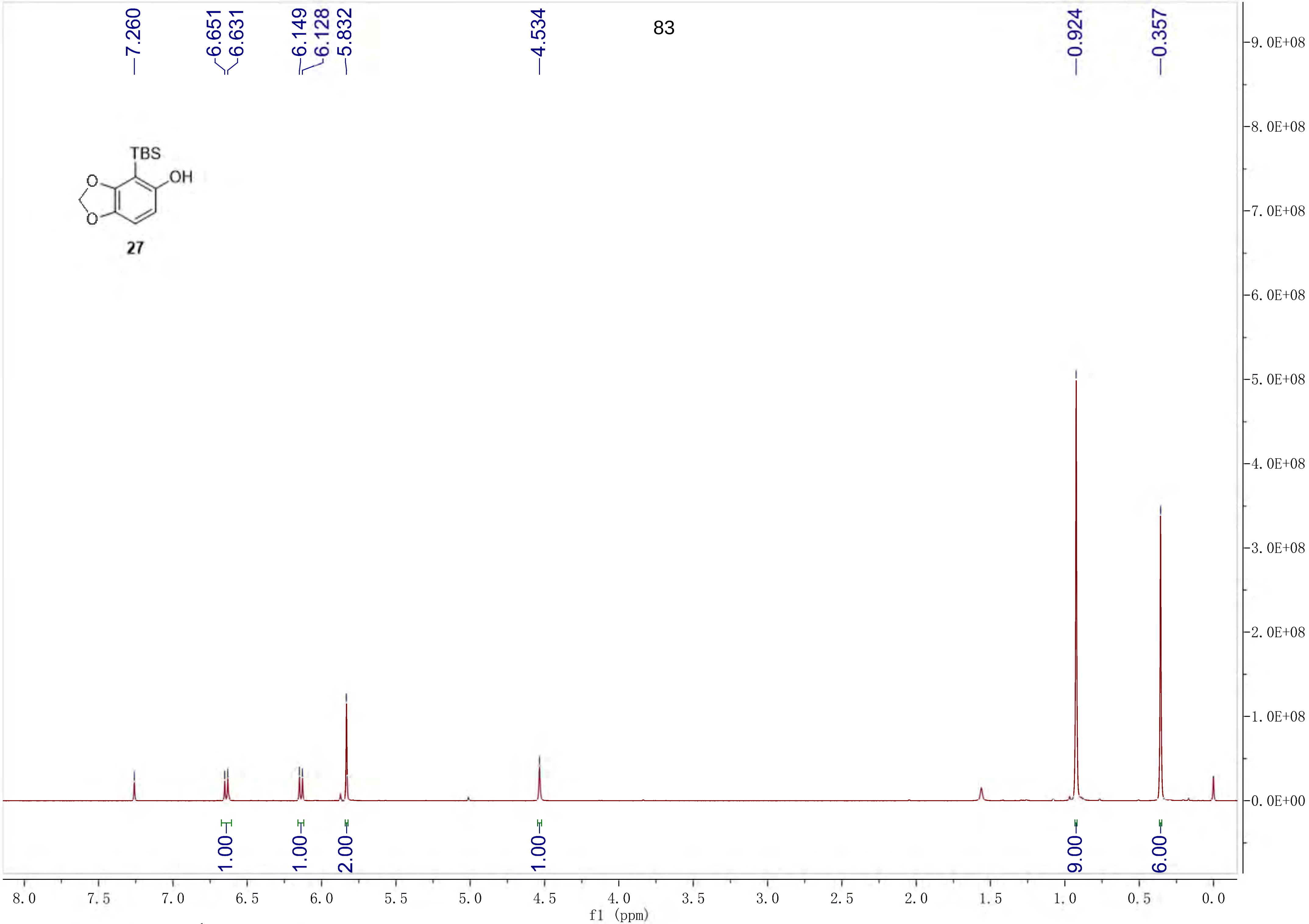

Supplementary Fig 11. ^{1}H NMR spectrum (400 MHz, $CDCl_3$, r.t.) of **27**.

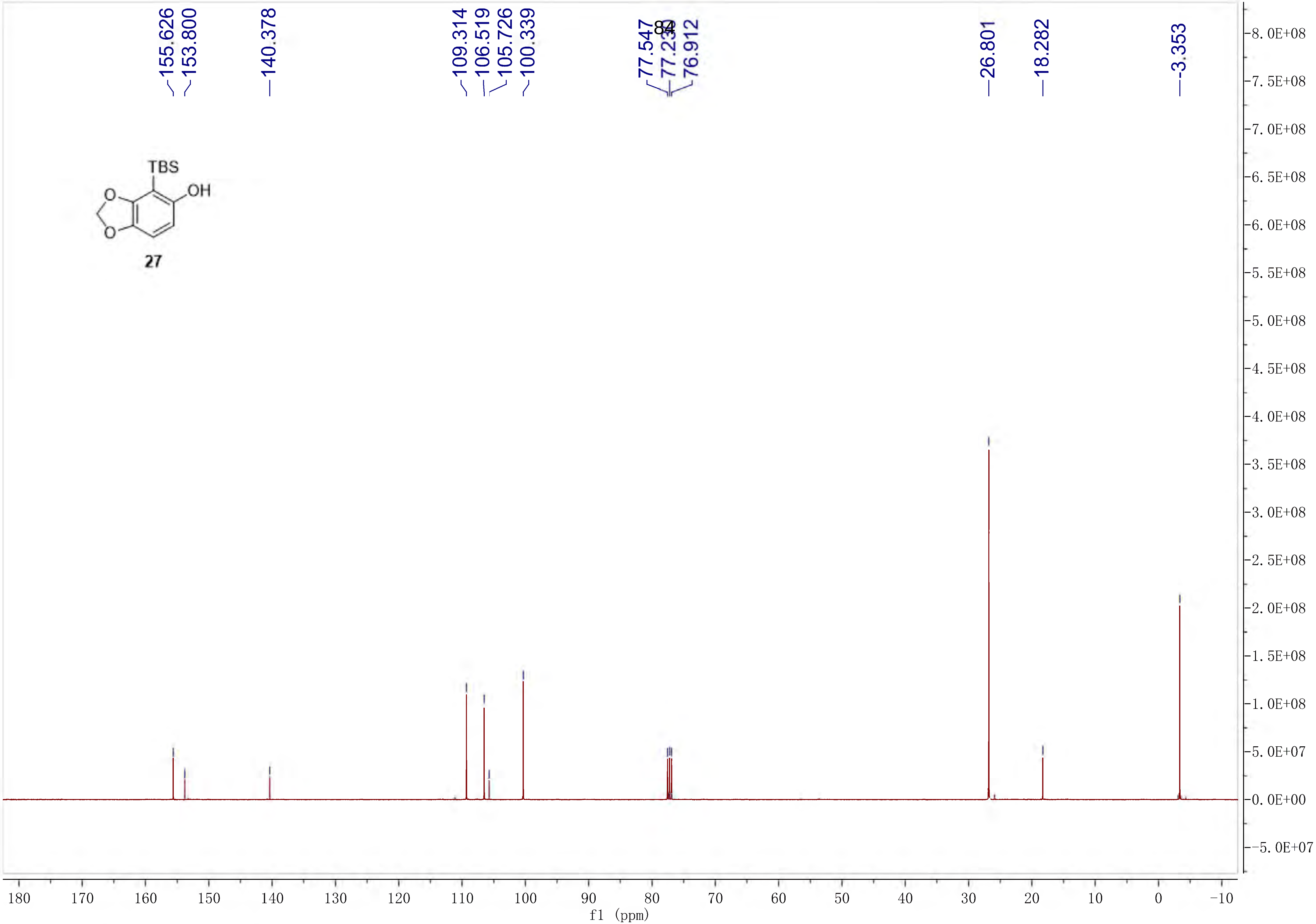

Supplementary Fig 12. ^{13}C NMR spectrum (400 MHz, $CDCl_3$, r.t.) of **27**.

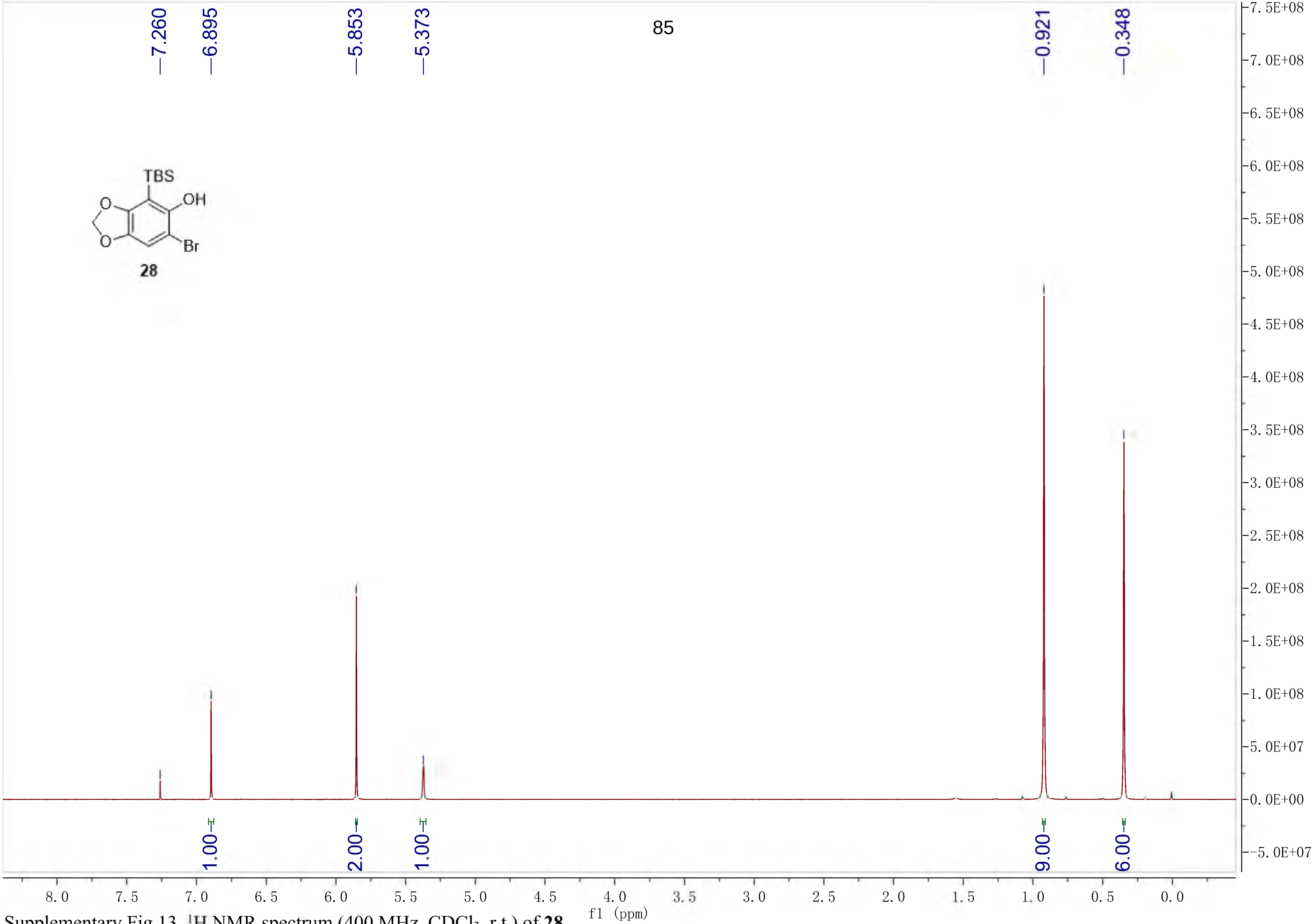

Supplementary Fig 13. ^{1}H NMR spectrum (400 MHz, $CDCl_3$, r.t.) of **28**.

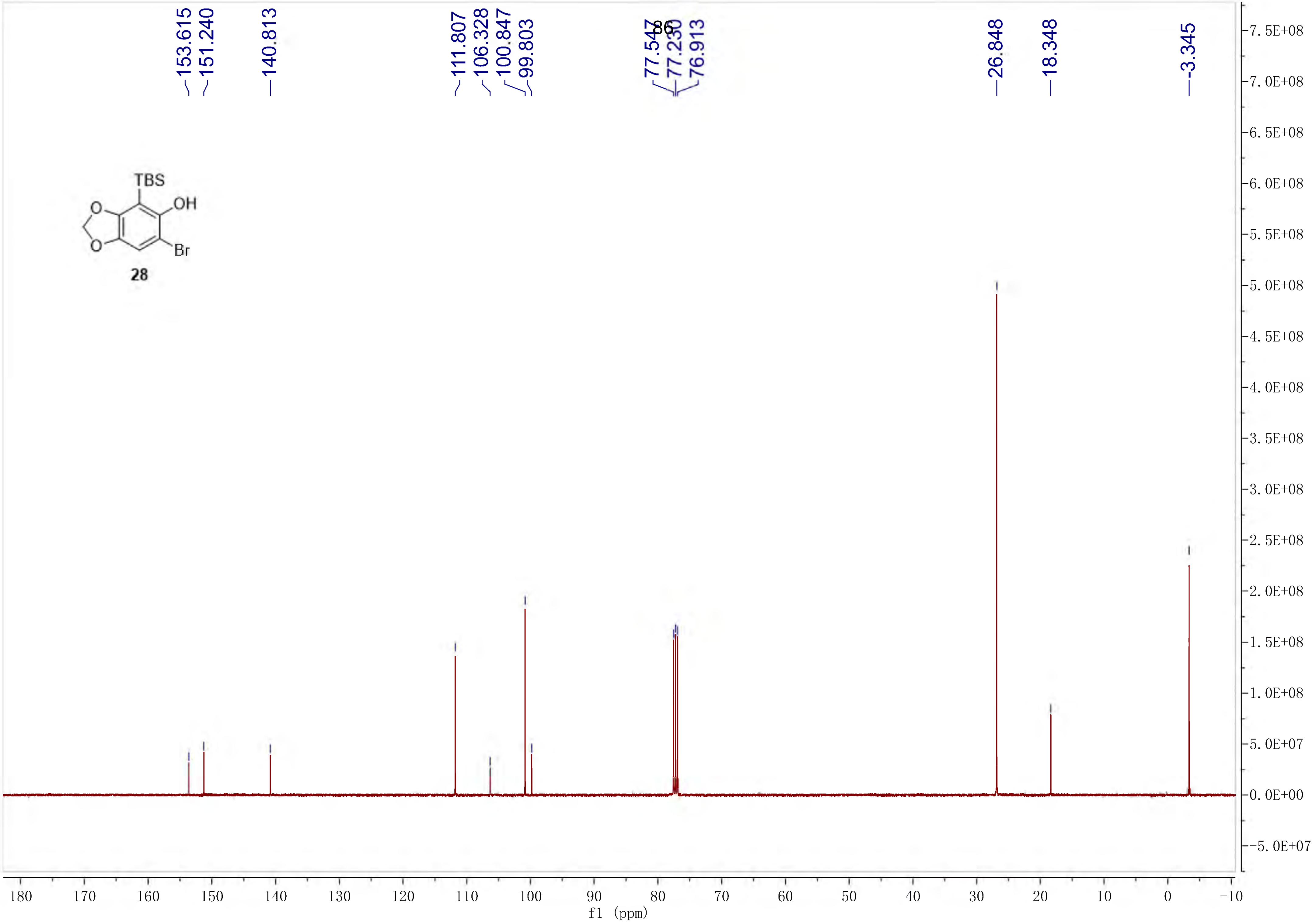

Supplementary Fig 14. ^{13}C NMR spectrum (400 MHz, $CDCl_3$, r.t.) of **28**.

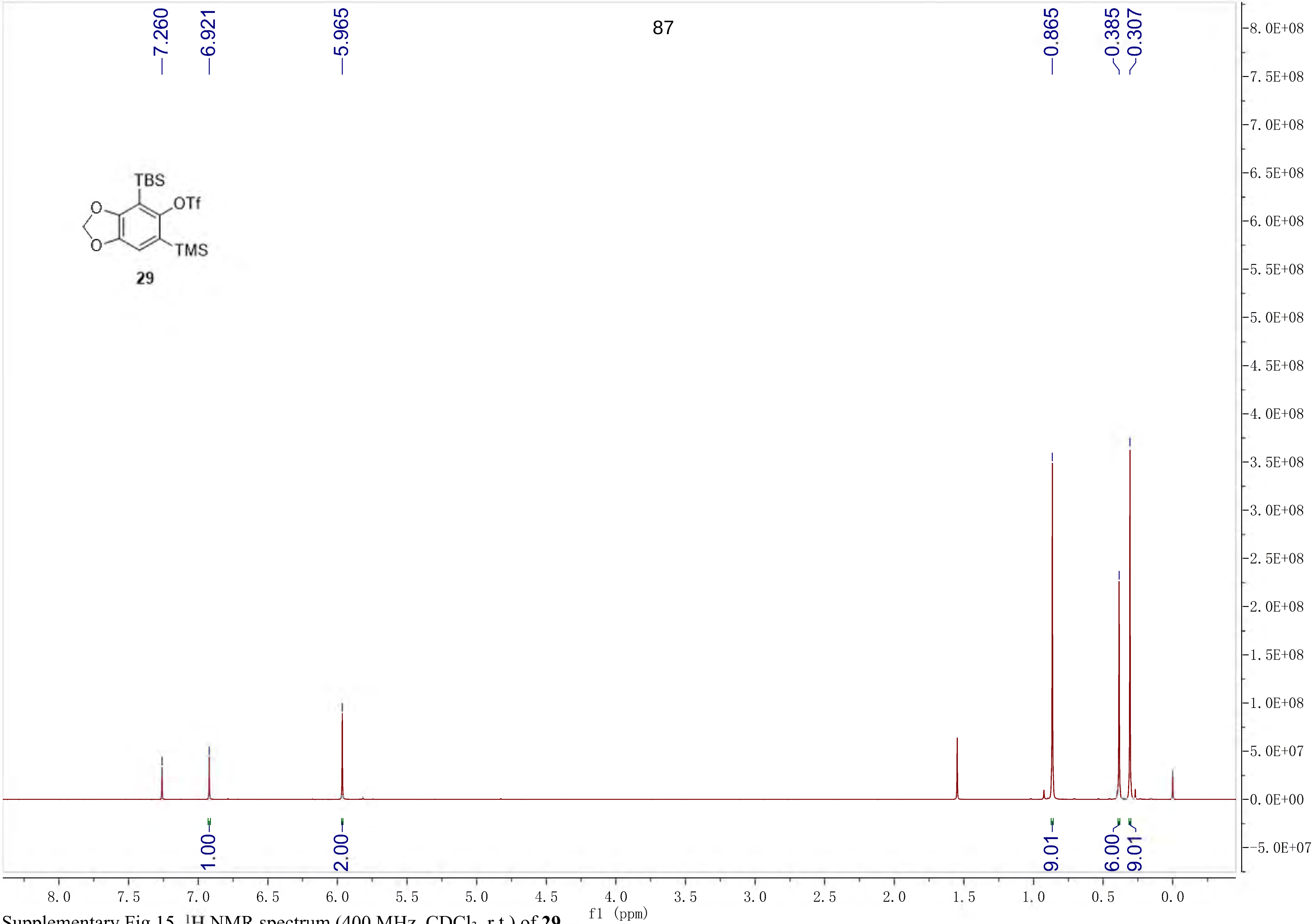

Supplementary Fig 15. ^{1}H NMR spectrum (400 MHz, $CDCl_3$, r.t.) of **29**.

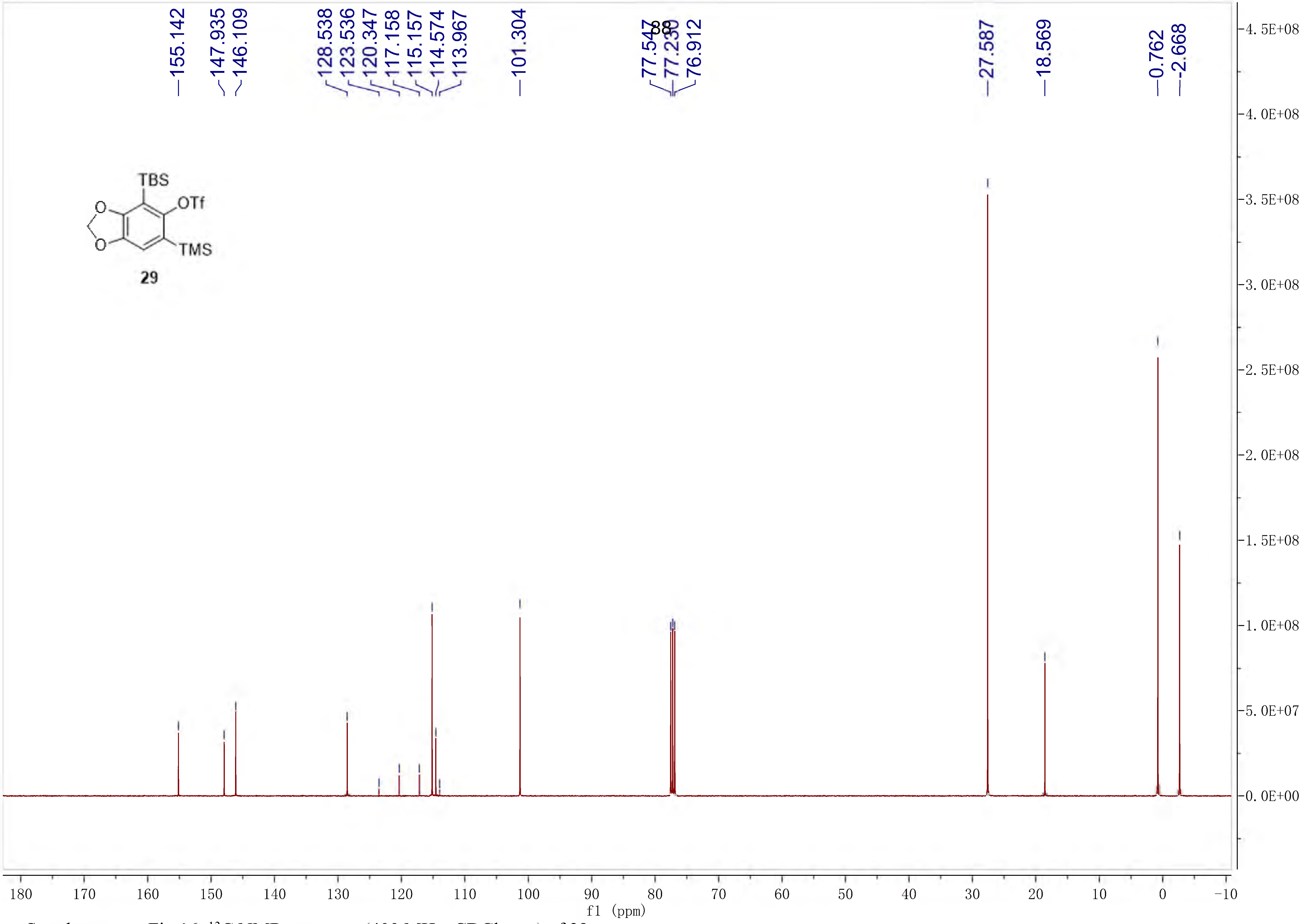

Supplementary Fig 16. ^{13}C NMR spectrum (400 MHz, $CDCl_3$, r.t.) of **29**.

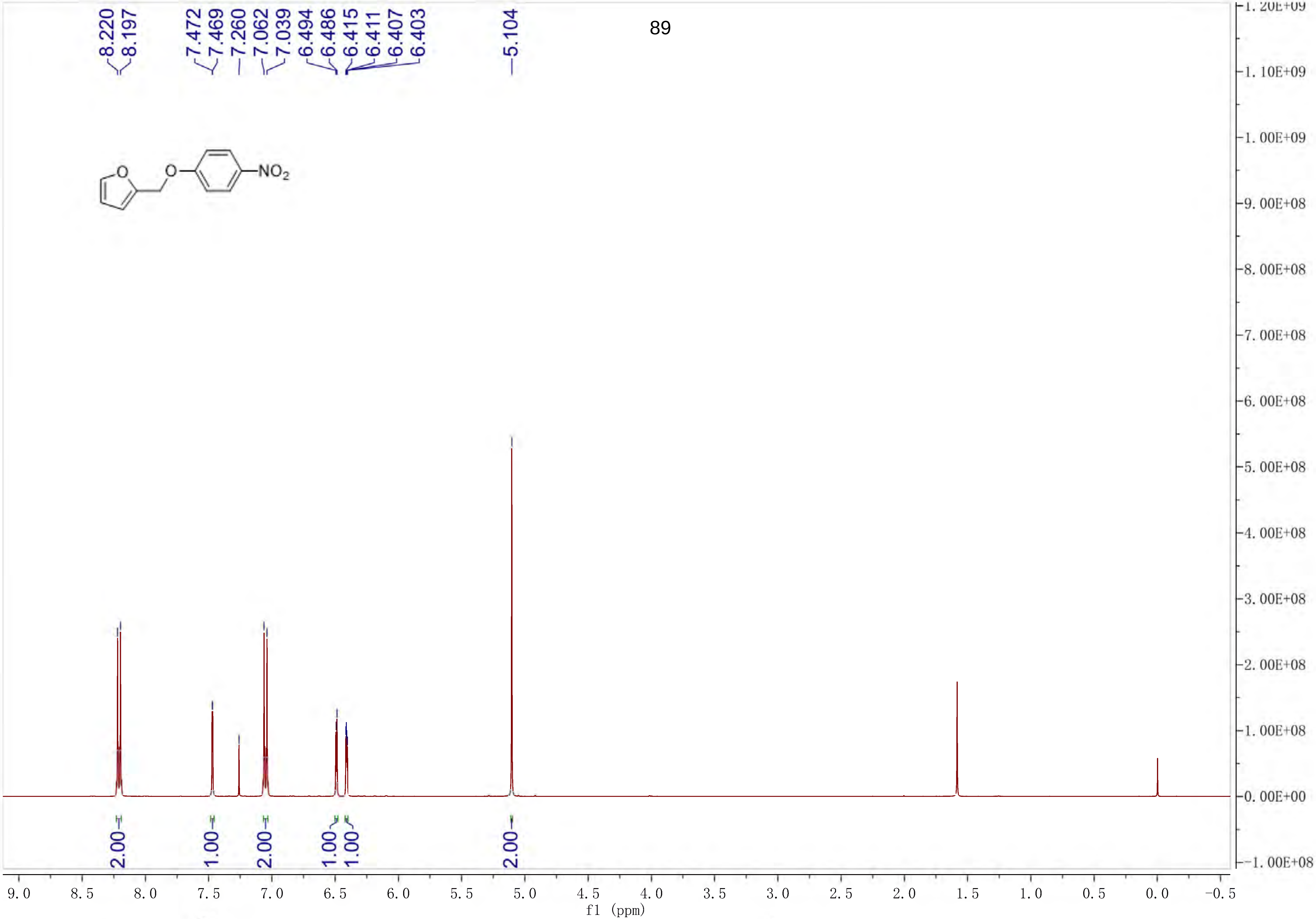

Supplementary Fig 17. ^{1}H NMR spectrum (400 MHz, $CDCl_3$, r.t.) of 2-((4-nitrophenoxy)methyl)furan.

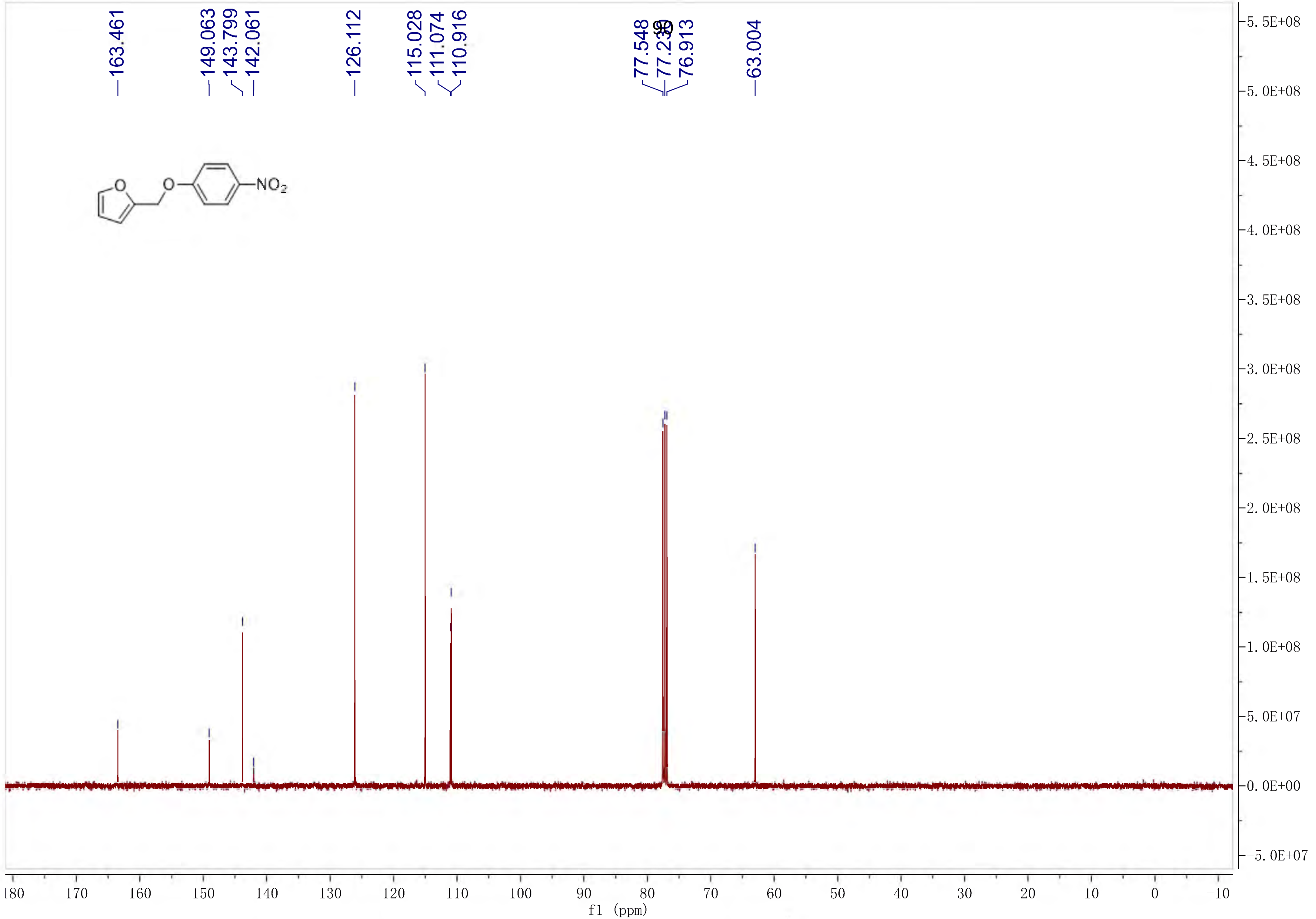

Supplementary Fig 18. ^{13}C NMR spectrum (400 MHz, $CDCl_3$, r.t.) of 2-((4-nitrophenoxy)methyl)furan.

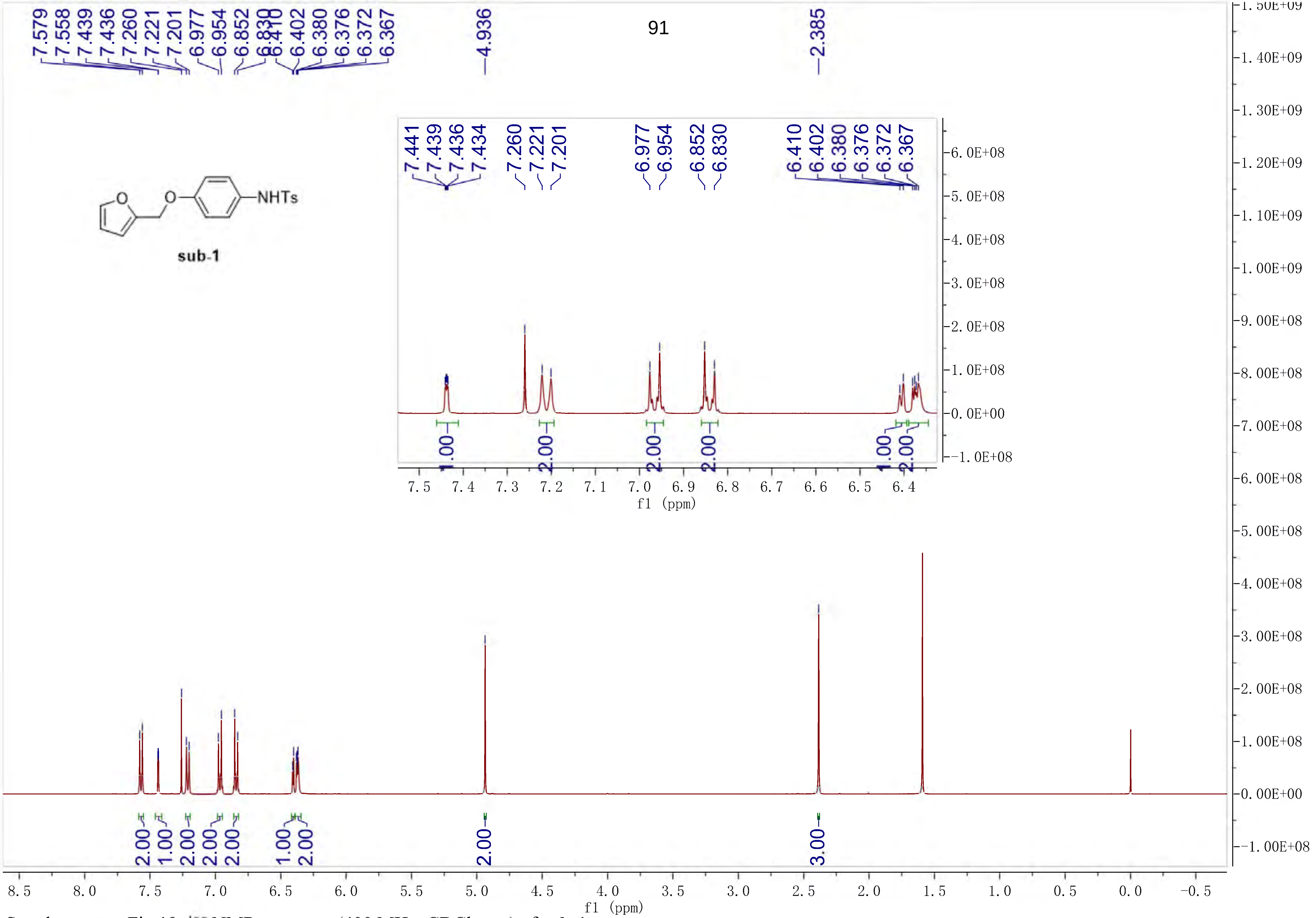

Supplementary Fig 19. ^{1}H NMR spectrum (400 MHz, $CDCl_3$, r.t.) of **sub-1**.

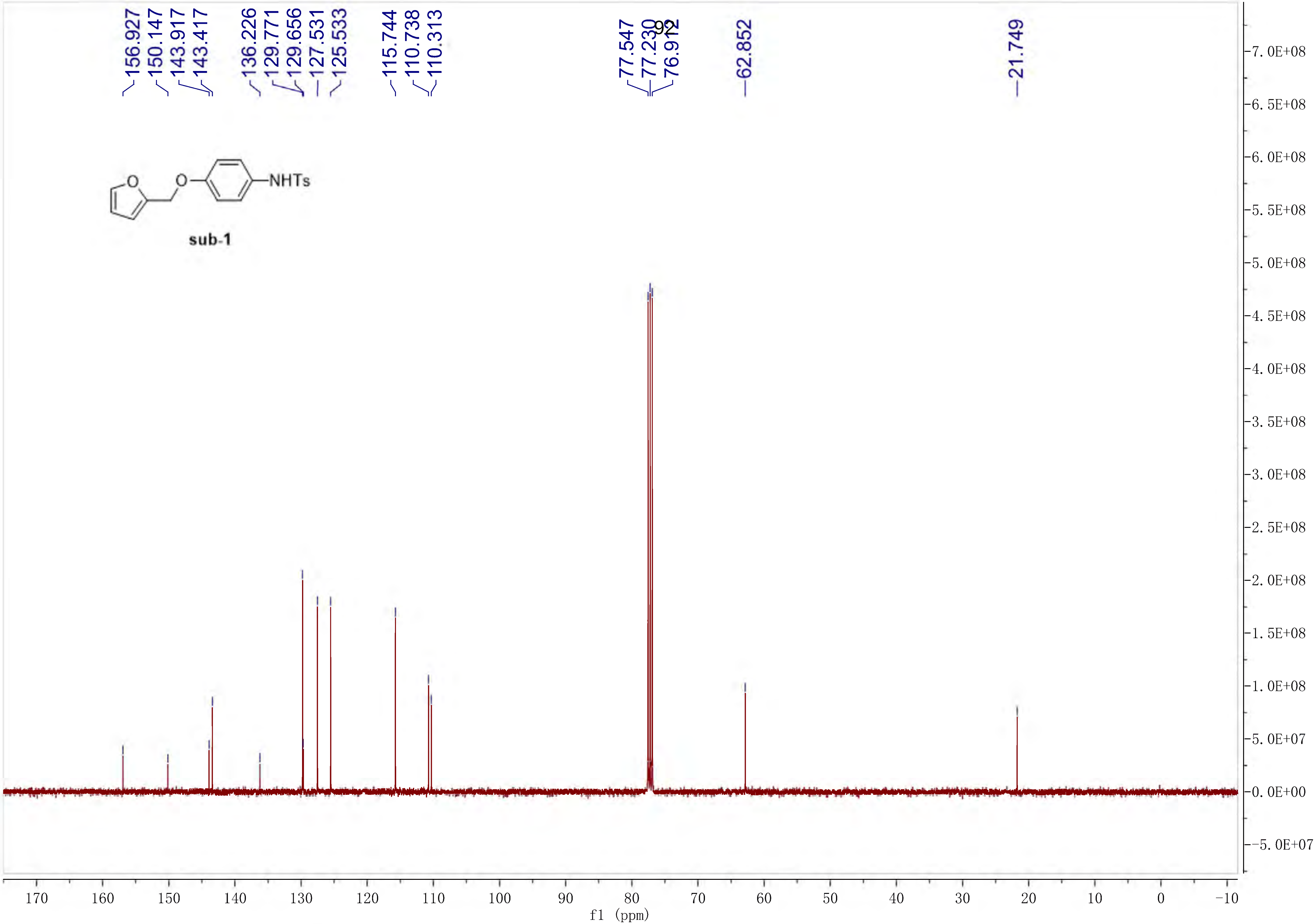

Supplementary Fig 20. ^{13}C NMR spectrum (400 MHz, $CDCl_3$, r.t.) of **sub-1**.

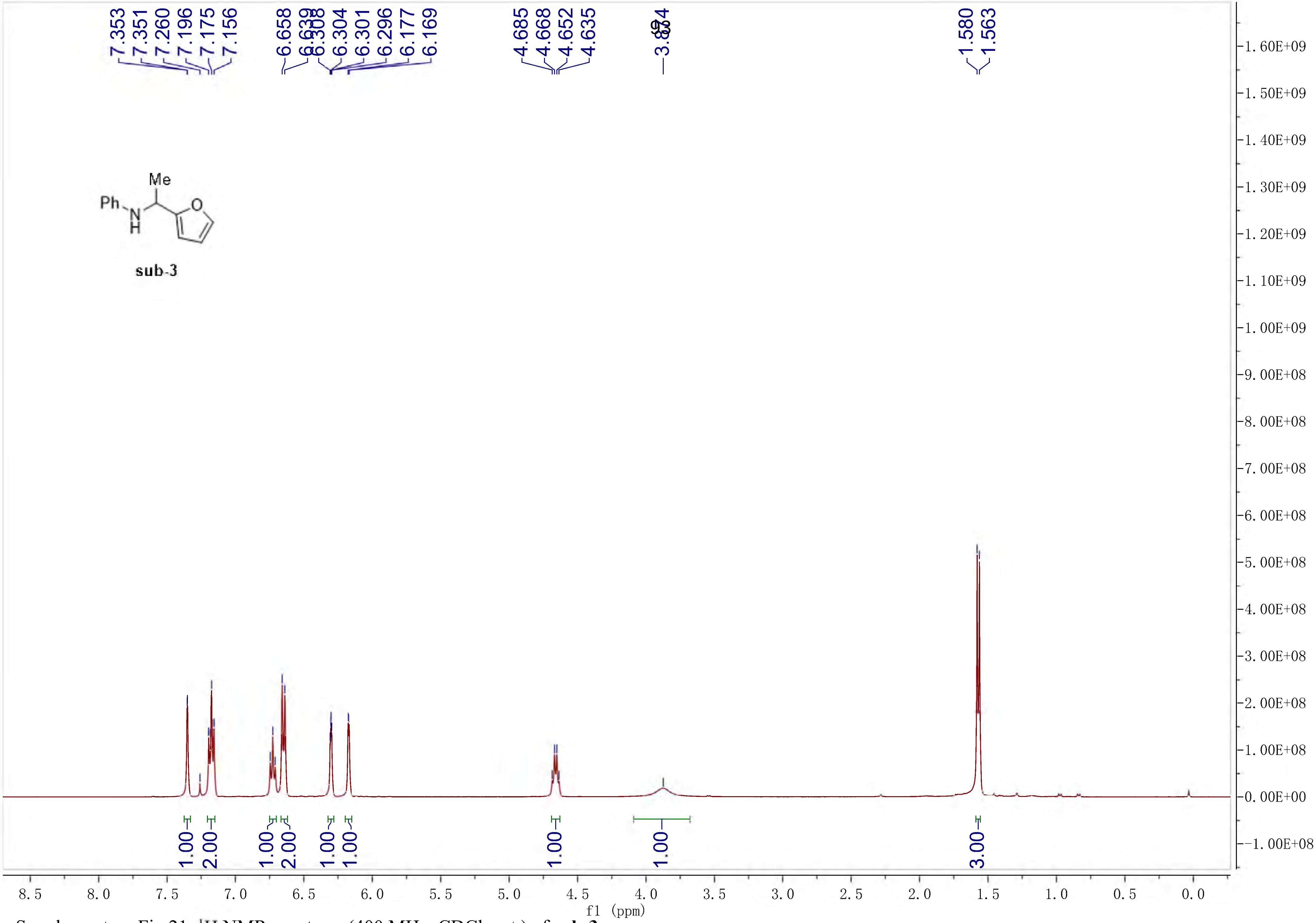

Supplementary Fig 21. ^{1}H NMR spectrum (400 MHz, $CDCl_3$, r.t.) of **sub-3**.

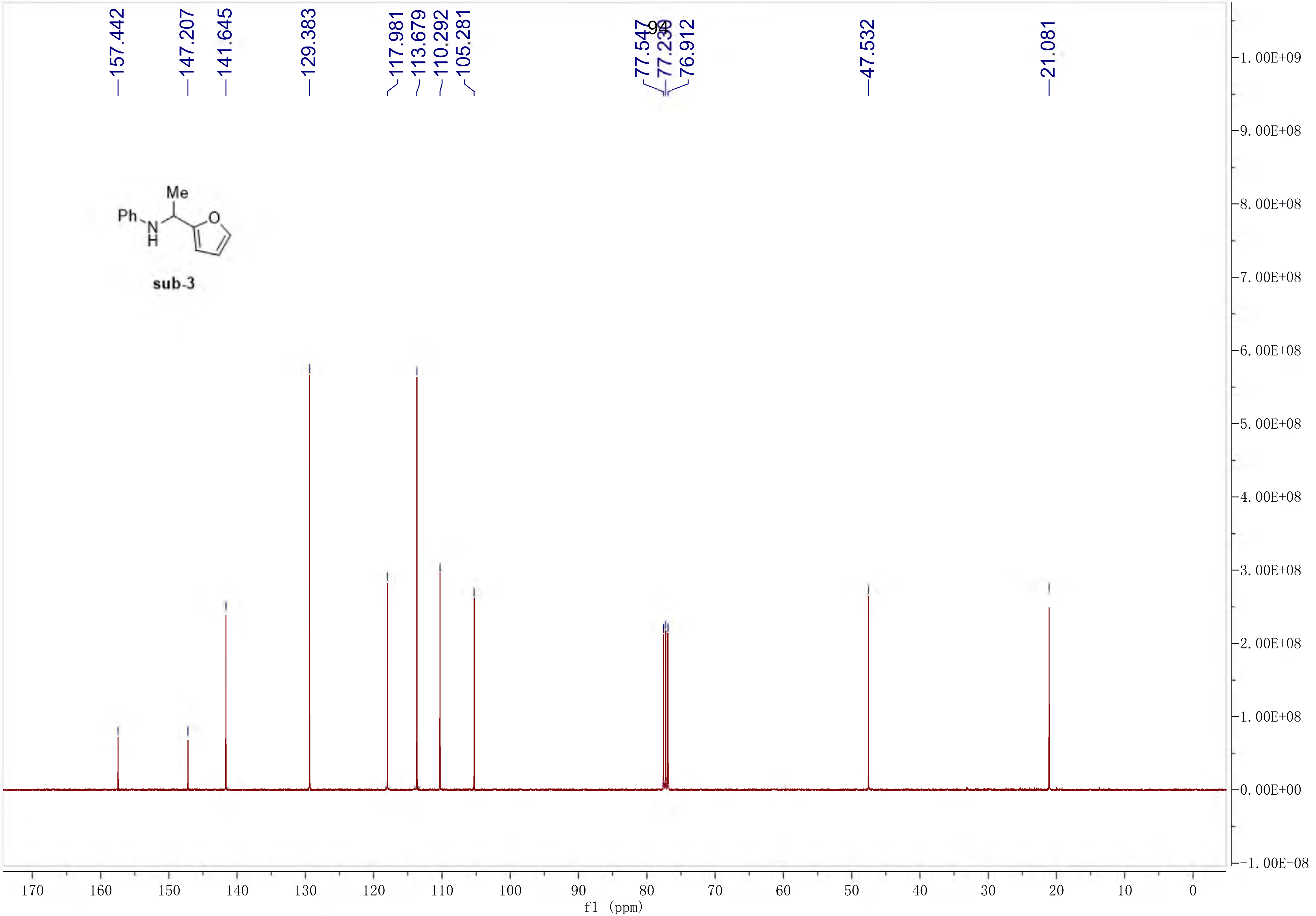

Supplementary Fig 22. ^{13}C NMR spectrum (400 MHz, $CDCl_3$, r.t.) of **sub-3**.

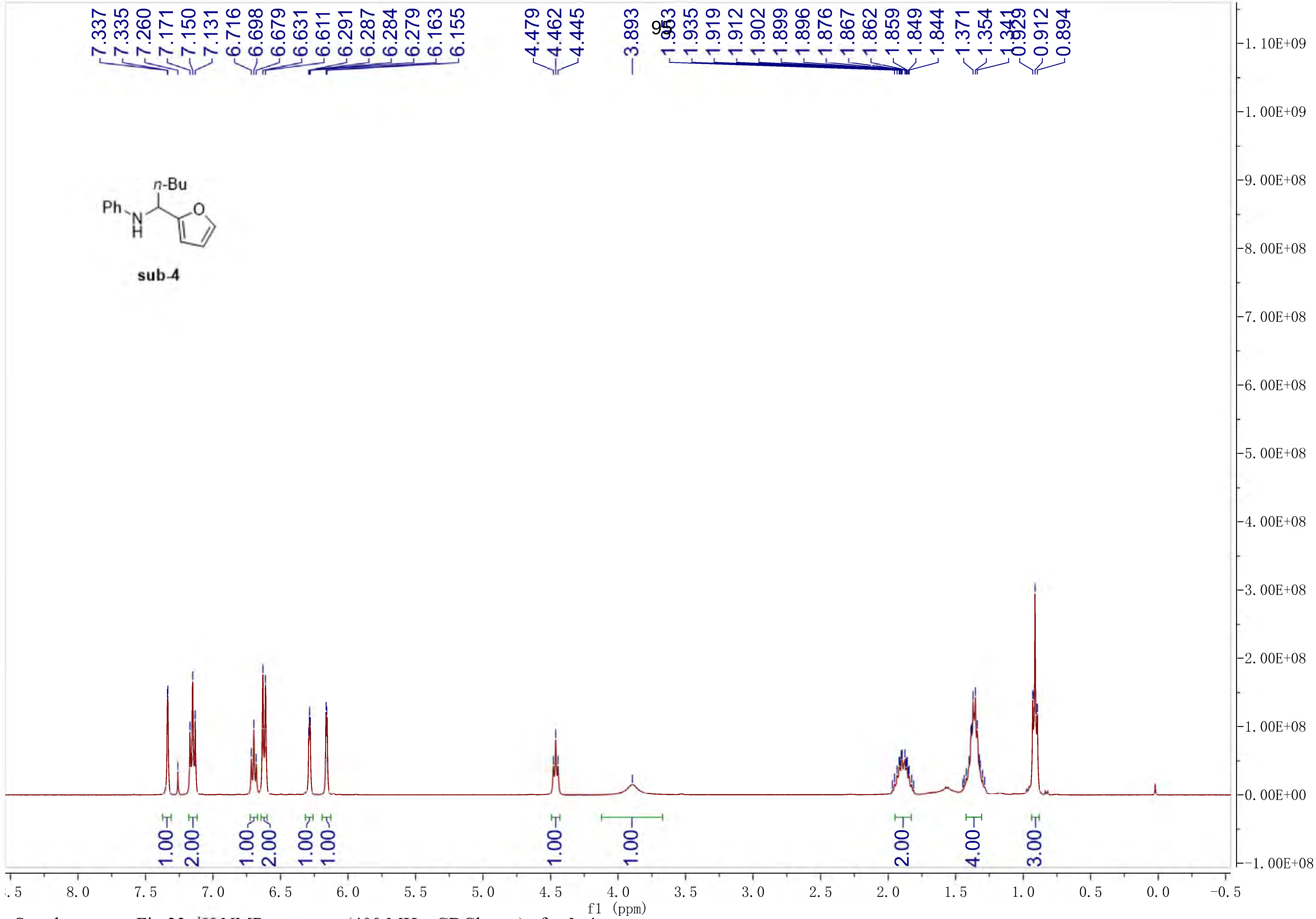

Supplementary Fig 23. ^{1}H NMR spectrum (400 MHz, $CDCl_3$, r.t.) of **sub-4**.

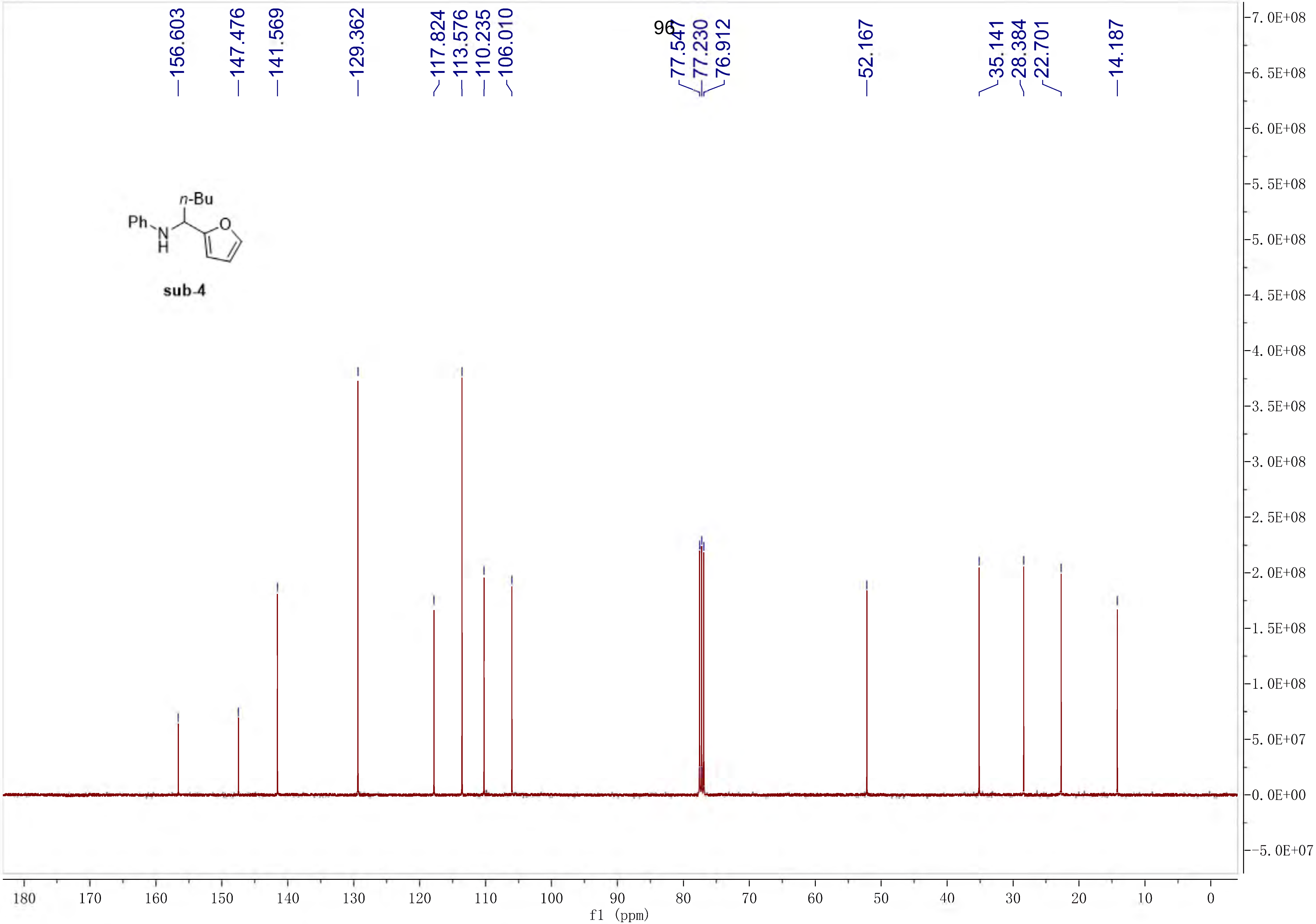

Supplementary Fig 24. ^{13}C NMR spectrum (400 MHz, $CDCl_3$, r.t.) of **sub-4**.

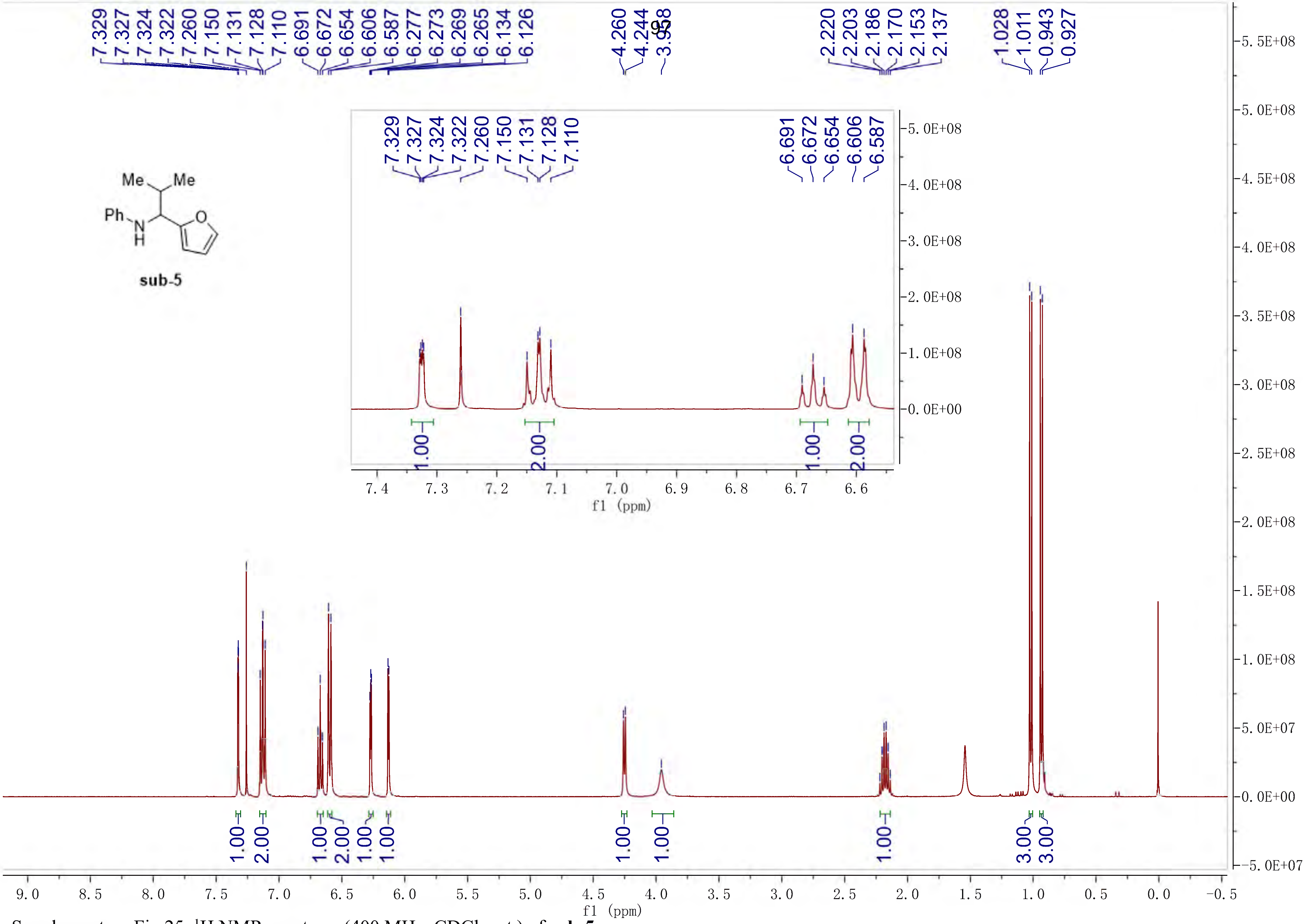

Supplementary Fig 25. ^{1}H NMR spectrum (400 MHz, $CDCl_3$, r.t.) of **sub-5**.

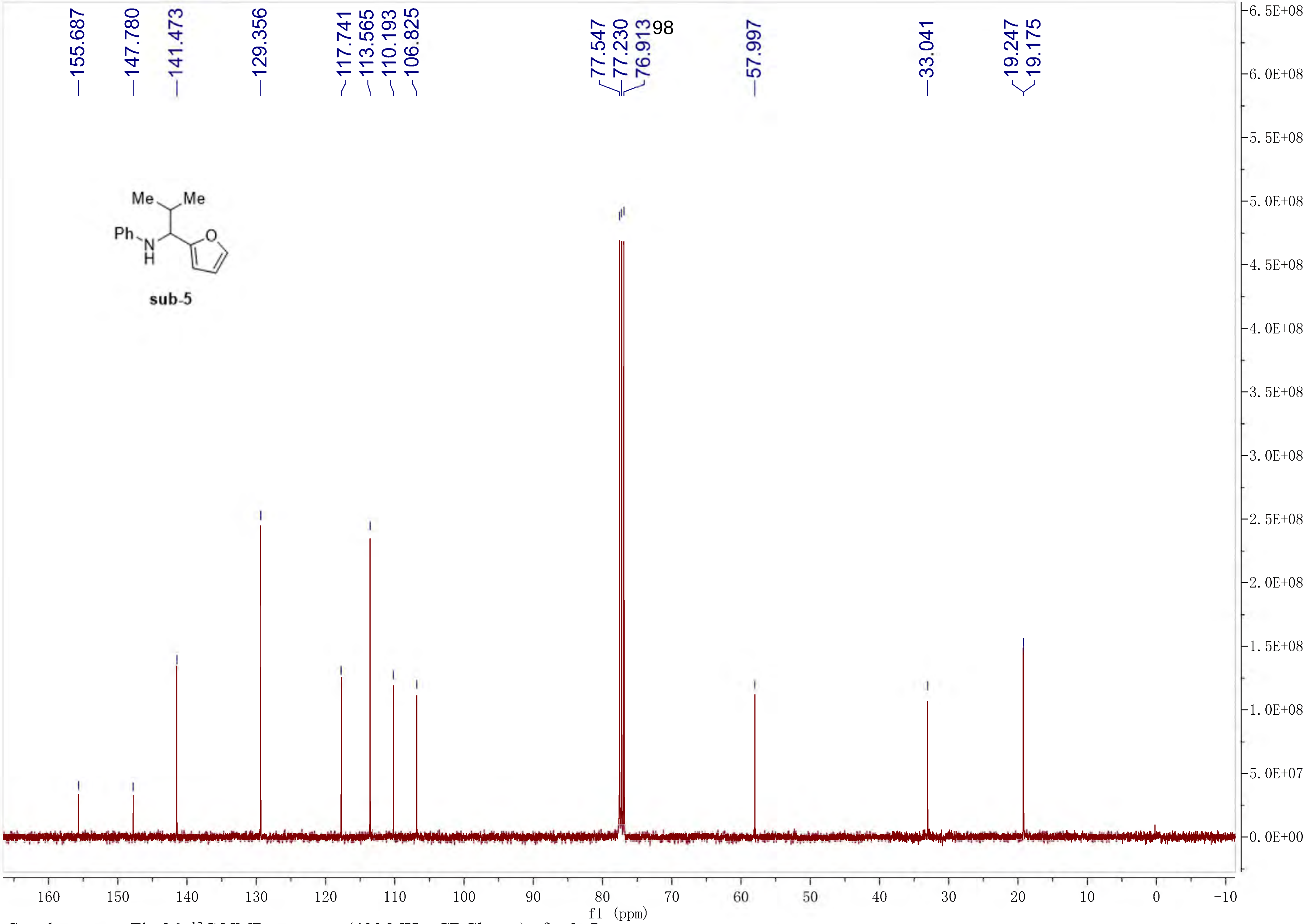

Supplementary Fig 26. ^{13}C NMR spectrum (400 MHz, $CDCl_3$, r.t.) of **sub-5**.

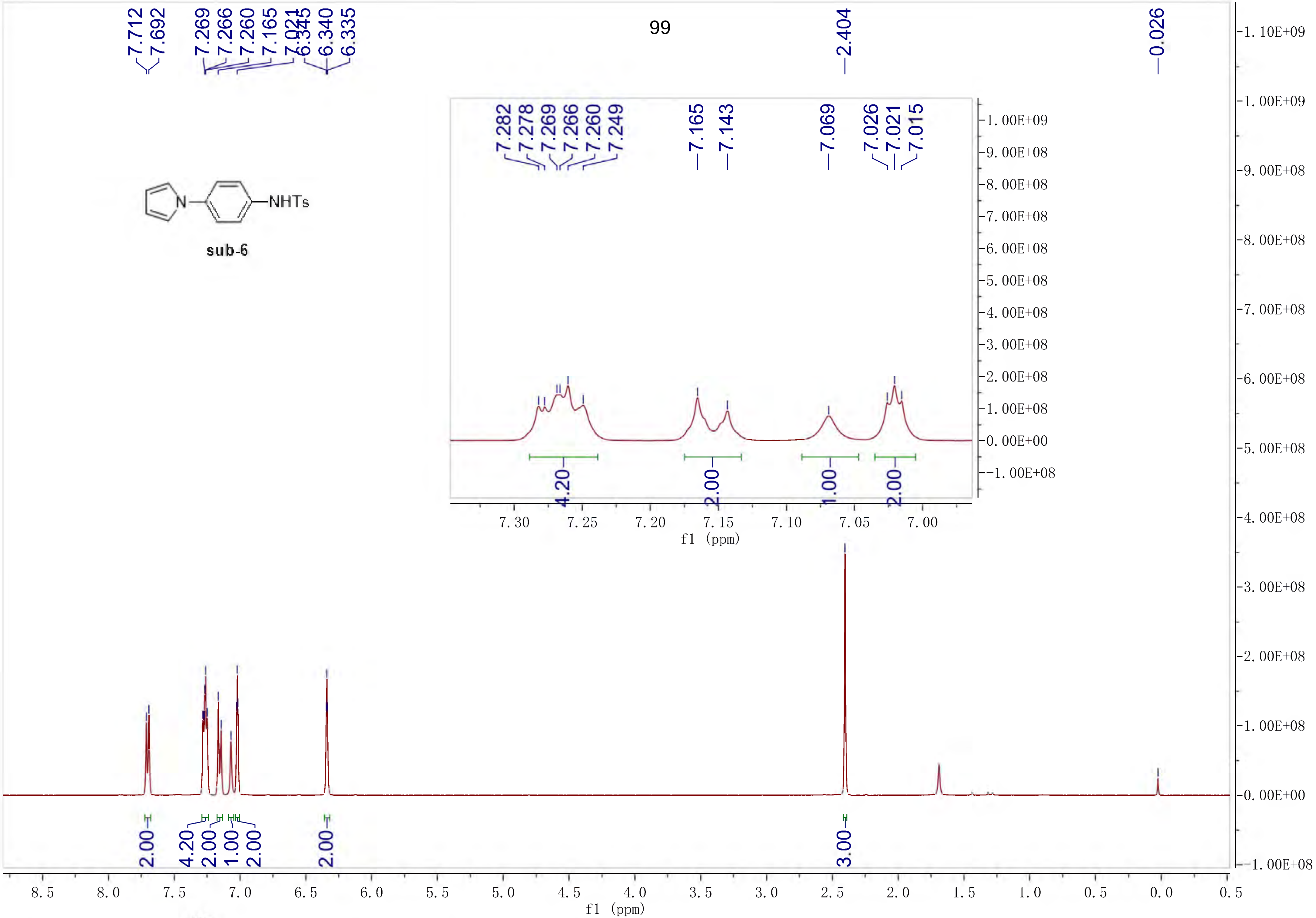

Supplementary Fig 27. ^{1}H NMR spectrum (400 MHz, $CDCl_3$, r.t.) of **sub-6**.

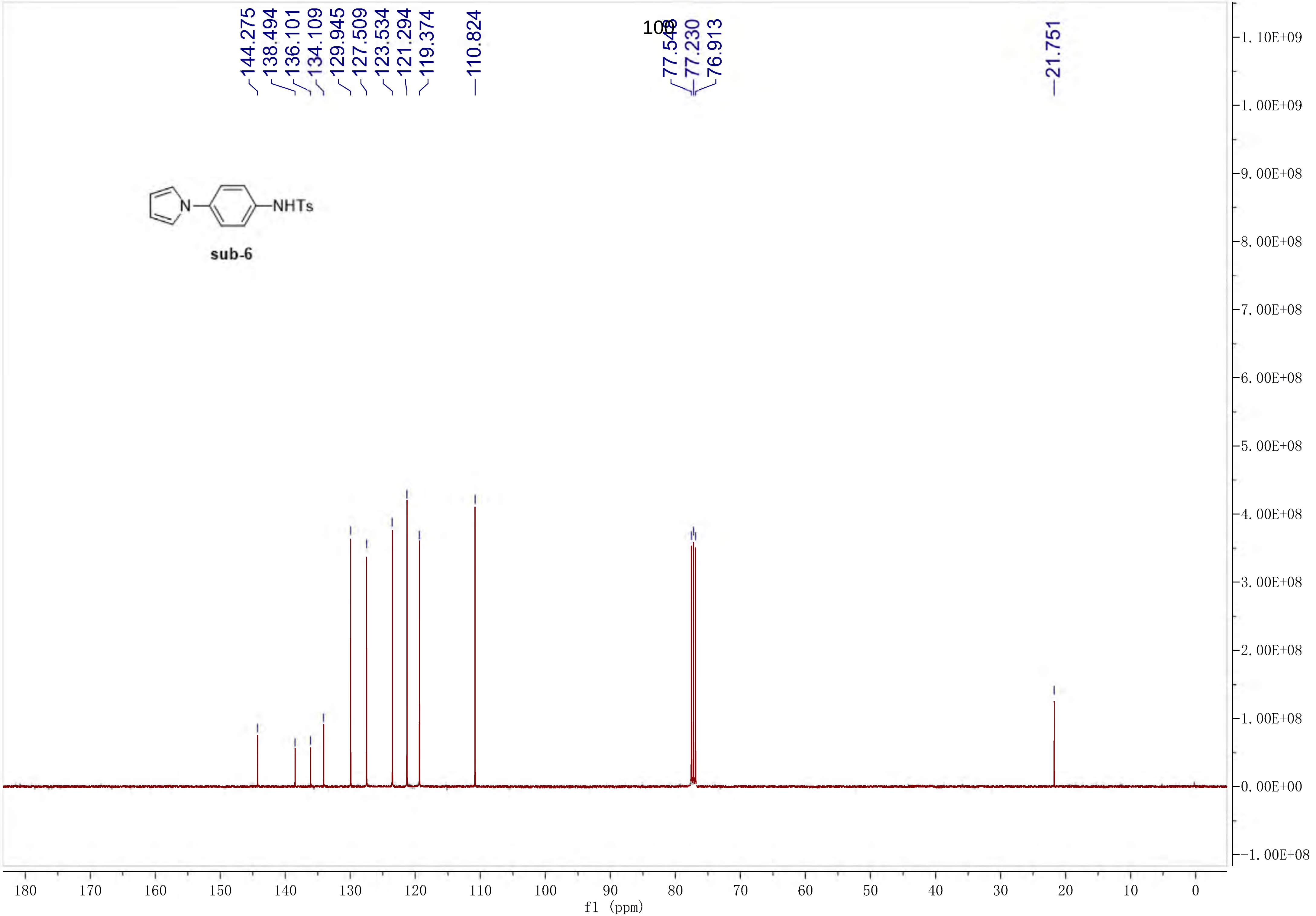

Supplementary Fig 28. ^{13}C NMR spectrum (400 MHz, $CDCl_3$, r.t.) of **sub-6**.

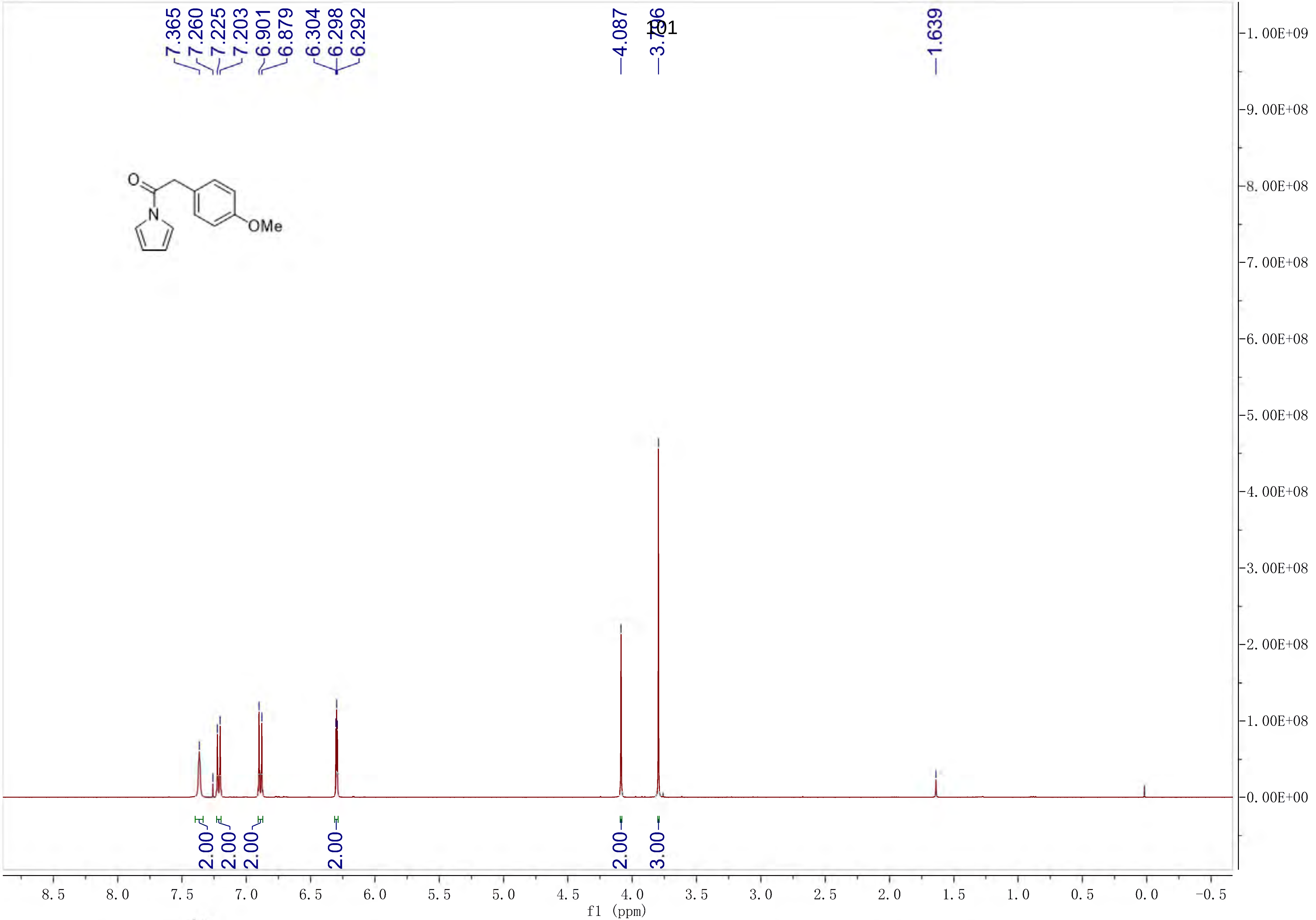

Supplementary Fig 29. ^{1}H NMR spectrum (400 MHz, $CDCl_3$, r.t.) of 2-(4-methoxyphenyl)-1-(1*H*-pyrrol-1-yl)ethan-1-one.

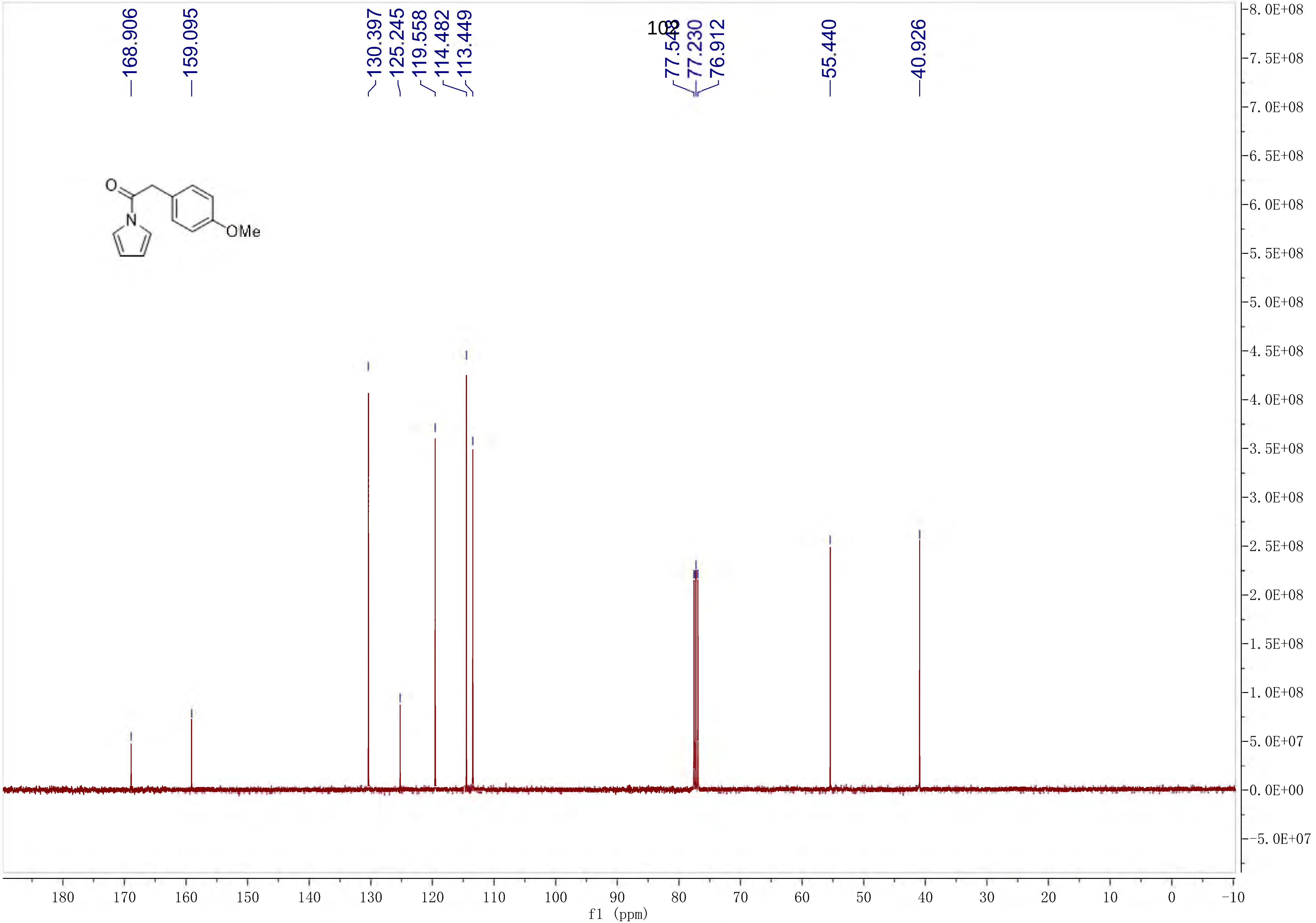

Supplementary Fig 30. ^{13}C NMR spectrum (400 MHz, $CDCl_3$, r.t.) of 2-(4-methoxyphenyl)-1-(1*H*-pyrrol-1-yl)ethan-1-one.

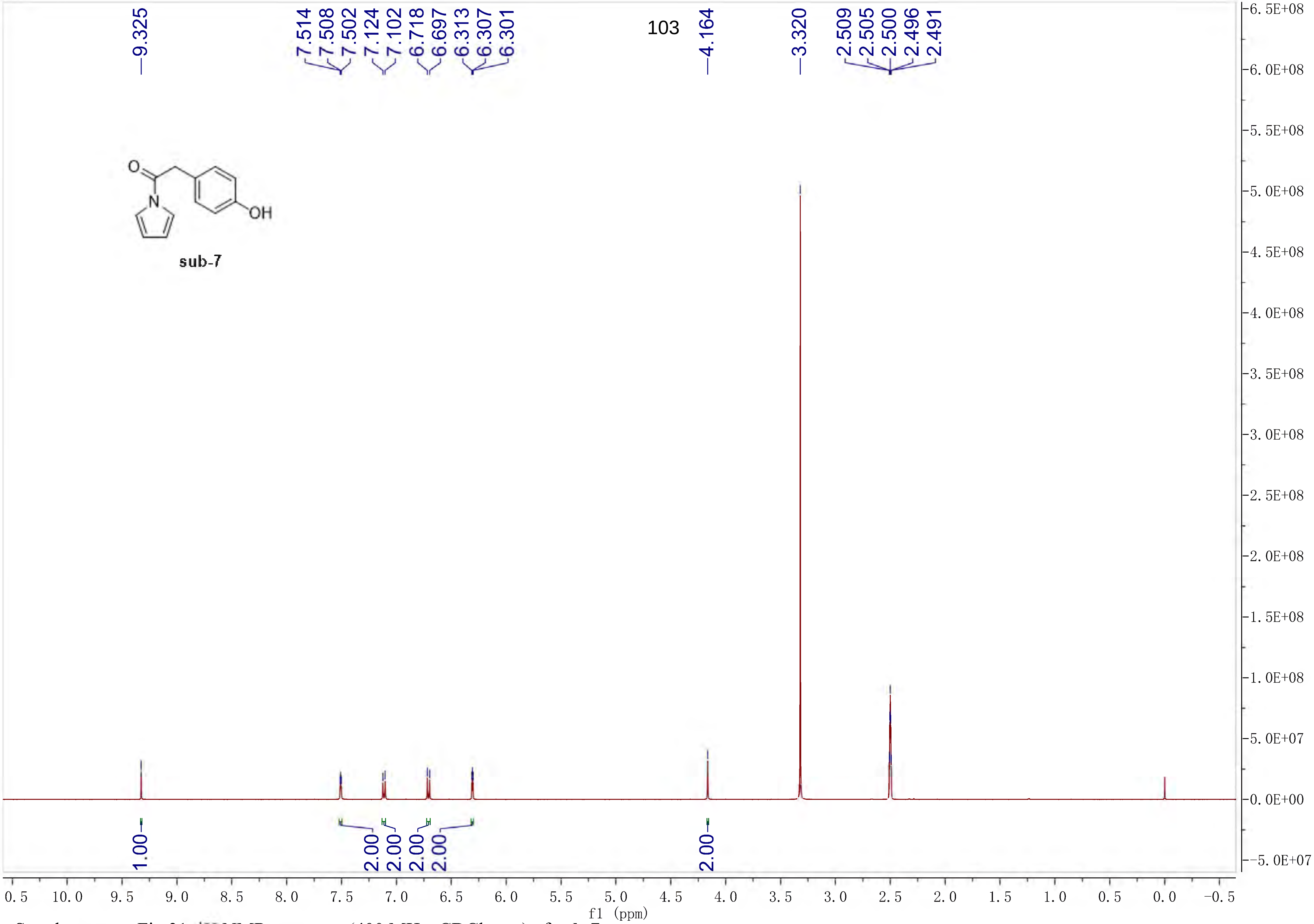

Supplementary Fig 31. ^1H NMR spectrum (400 MHz, $CDCl_3$, r.t.) of **sub-7**.

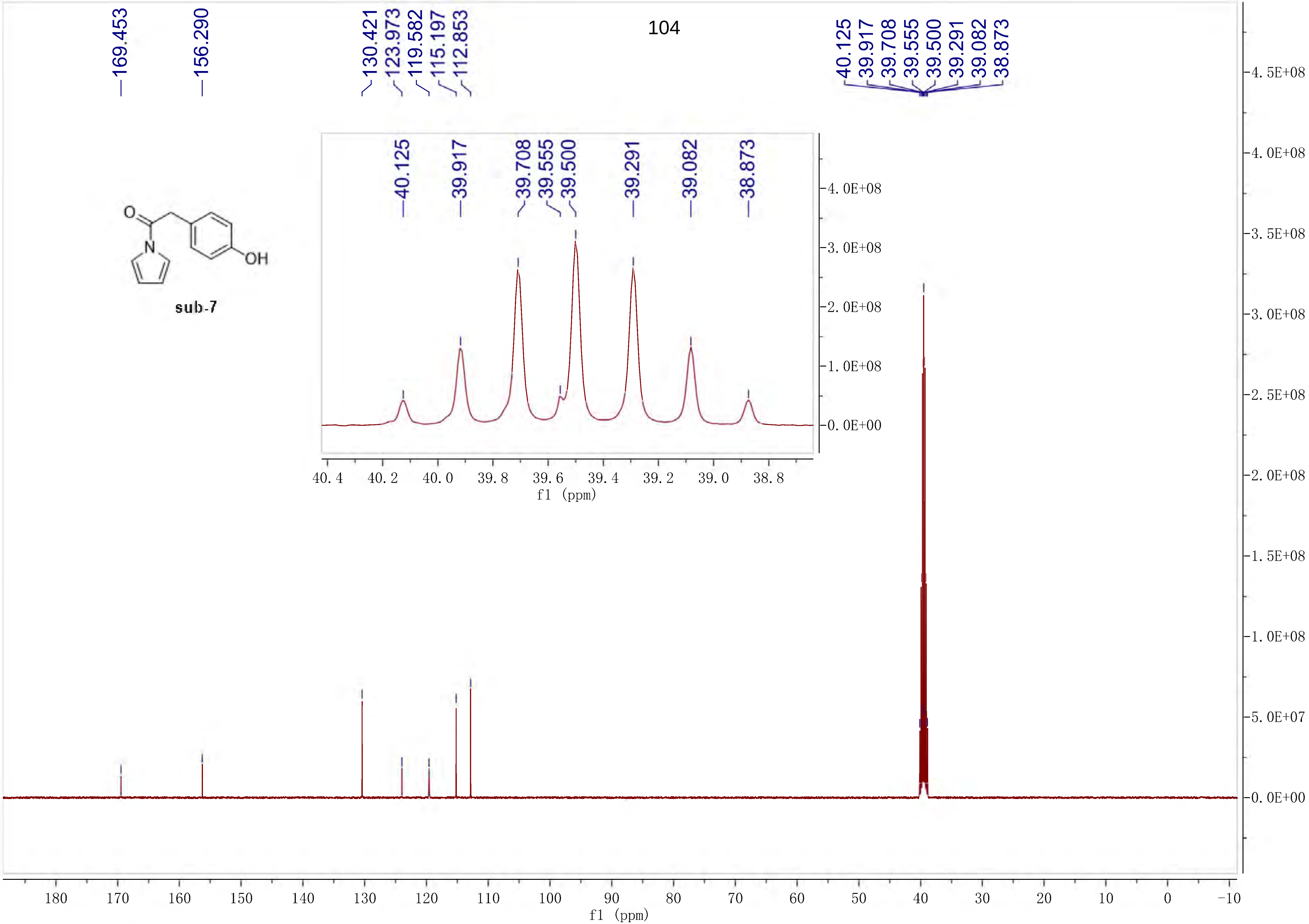

Supplementary Fig 32. ^{13}C NMR spectrum (400 MHz, $CDCl_3$, r.t.) of **sub-7**.

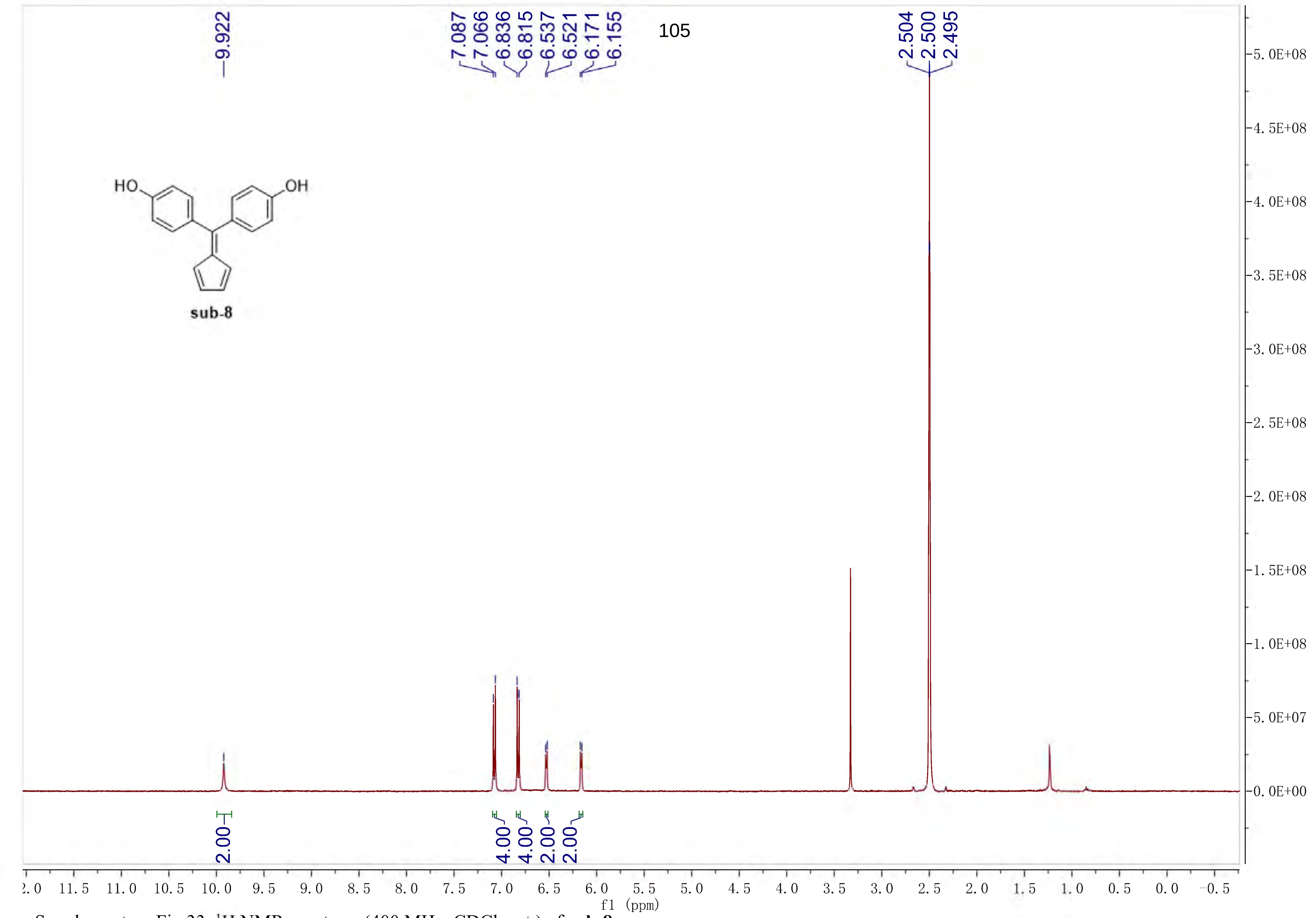

Supplementary Fig 33. ^{1}H NMR spectrum (400 MHz, $CDCl_3$, r.t.) of **sub-8**.

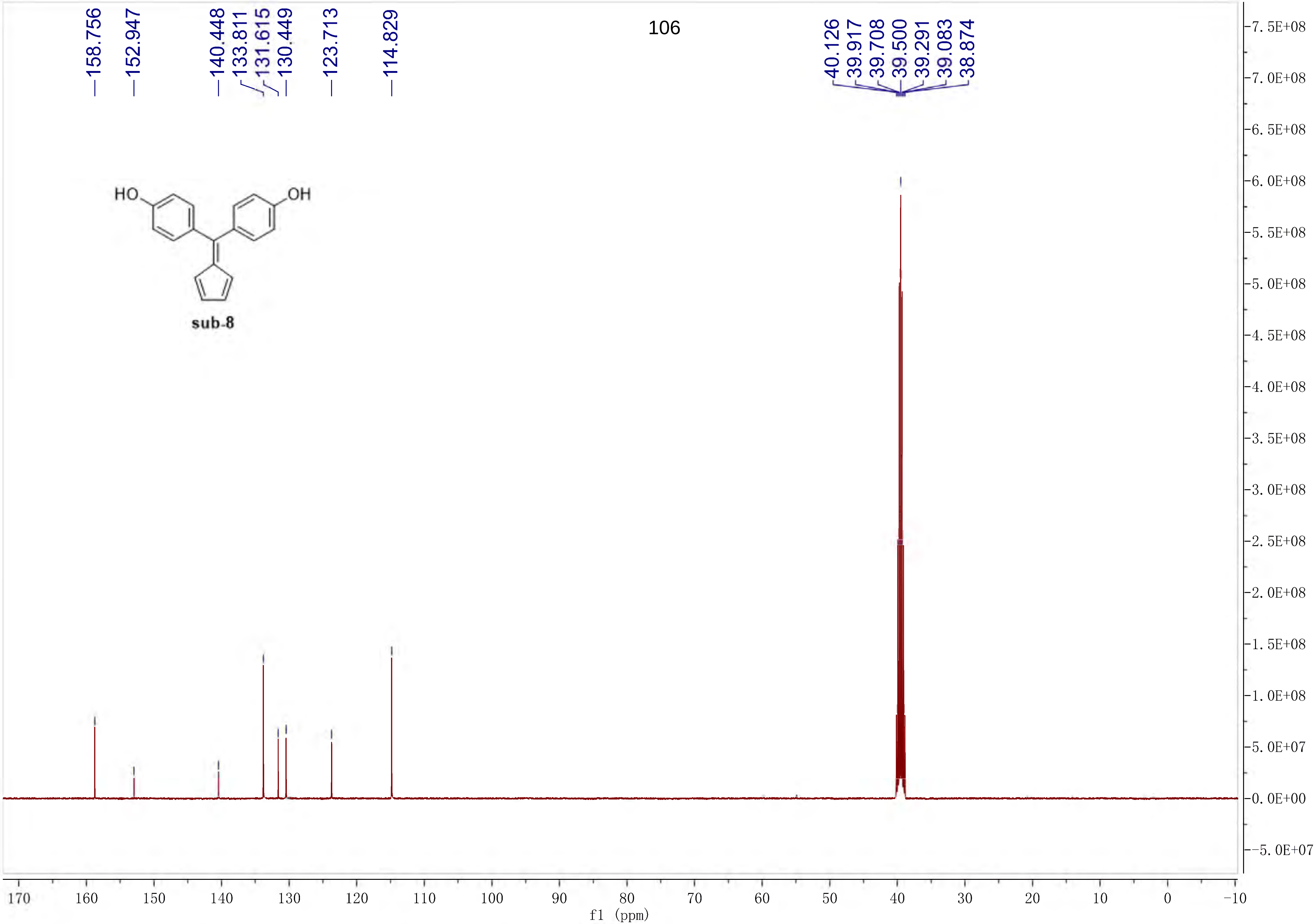

sub-8

Supplementary Fig 34. ^{13}C NMR spectrum (400 MHz, $CDCl_3$, r.t.) of **sub-8**.

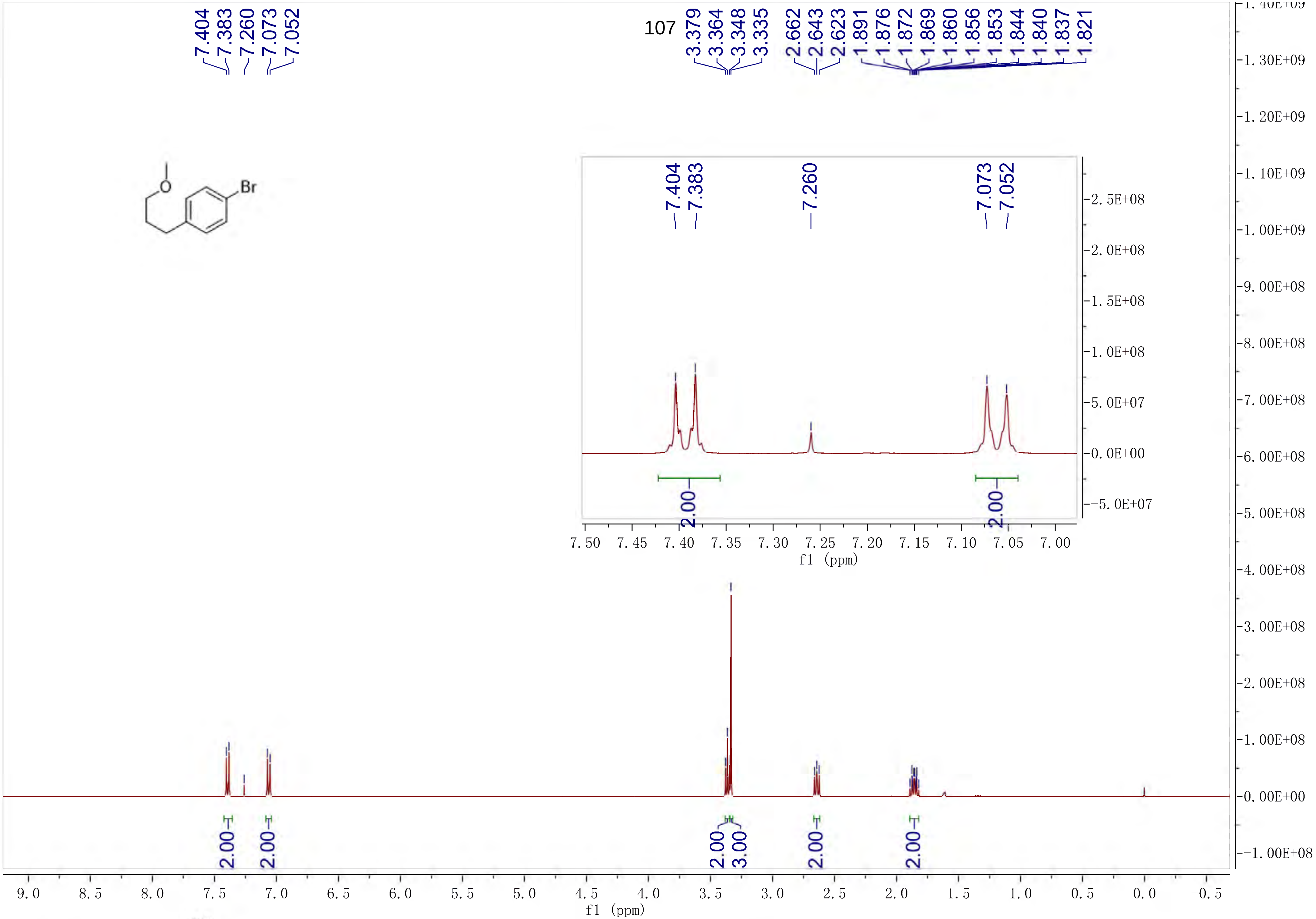

Supplementary Fig 35. ^{1}H NMR spectrum (400 MHz, $CDCl_3$, r.t.) of 1-bromo-4-(3-methoxypropyl)benzene.

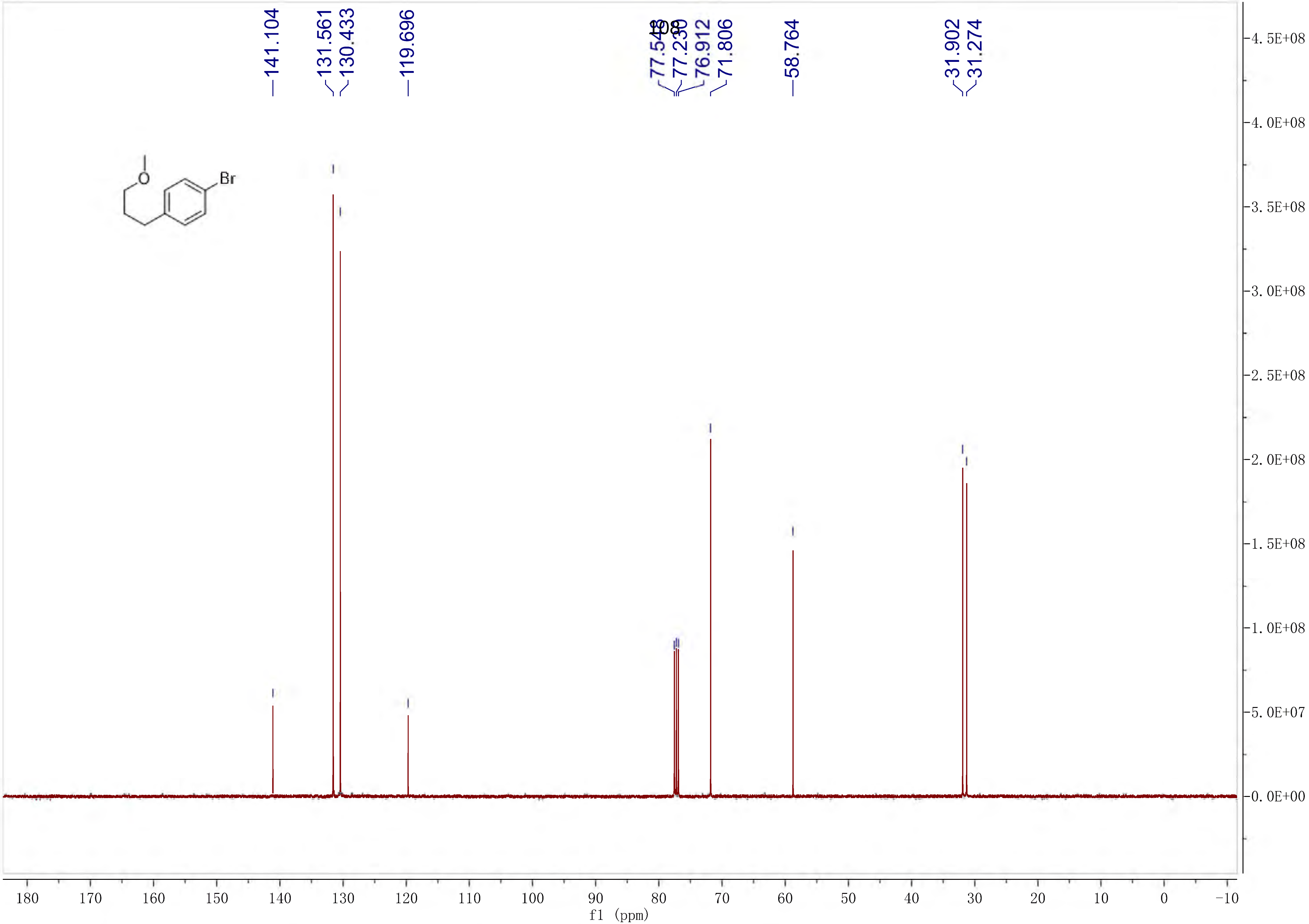

Supplementary Fig 36. ^{13}C NMR spectrum (400 MHz, $CDCl_3$, r.t.) of 1-bromo-4-(3-methoxypropyl)benzene.

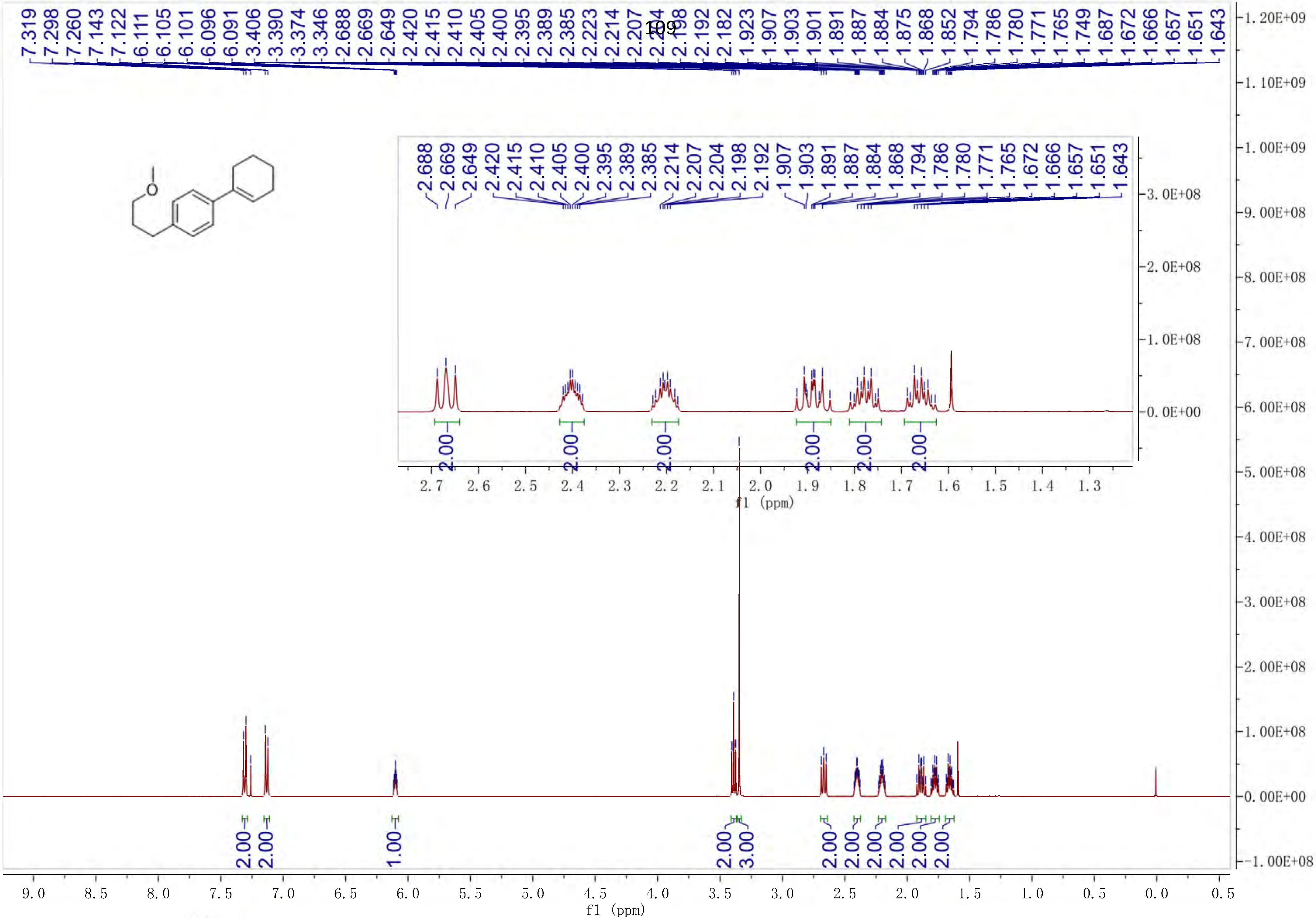

Supplementary Fig 37. ^{1}H NMR spectrum (400 MHz, $CDCl_3$, r.t.) of 4'-(3-methoxypropyl)-2,3,4,5-tetrahydro-1,1'-biphenyl.

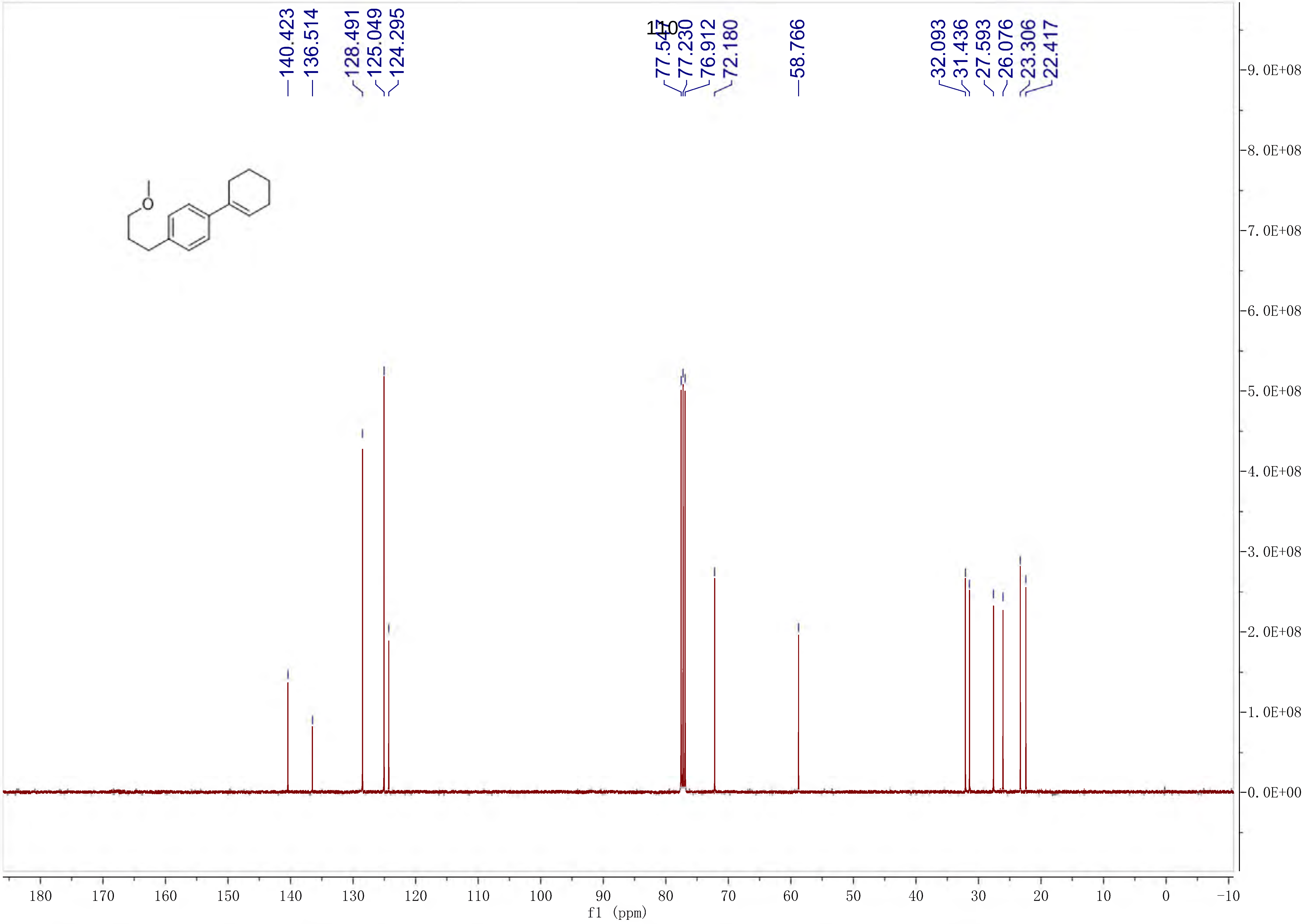

Supplementary Fig 38. ^{13}C NMR spectrum (400 MHz, $CDCl_3$, r.t.) of 4'-(3-methoxypropyl)-2,3,4,5-tetrahydro-1,1'-biphenyl.

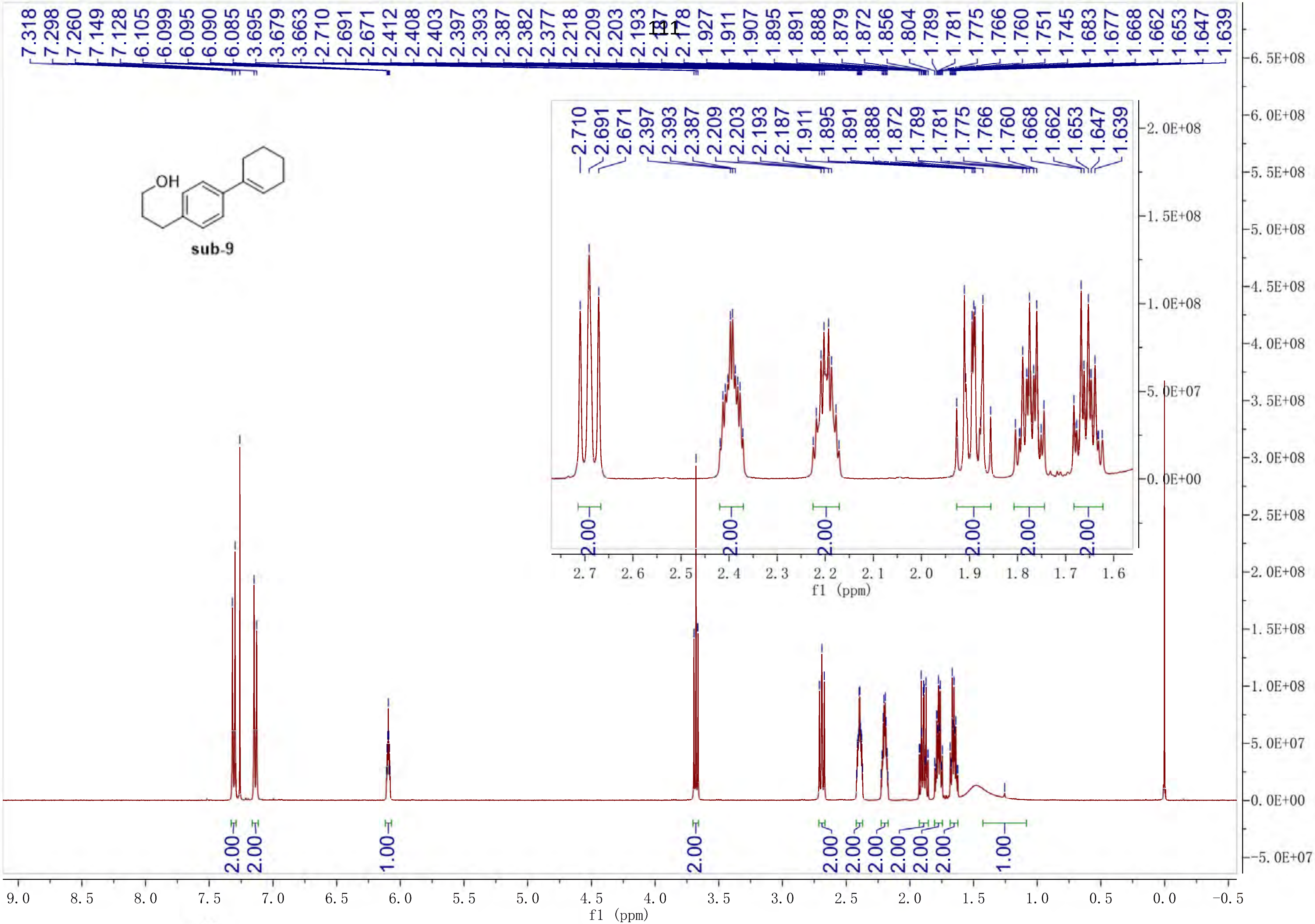

Supplementary Fig 39. ^{1}H NMR spectrum (400 MHz, $CDCl_3$, r.t.) of **sub-9**.

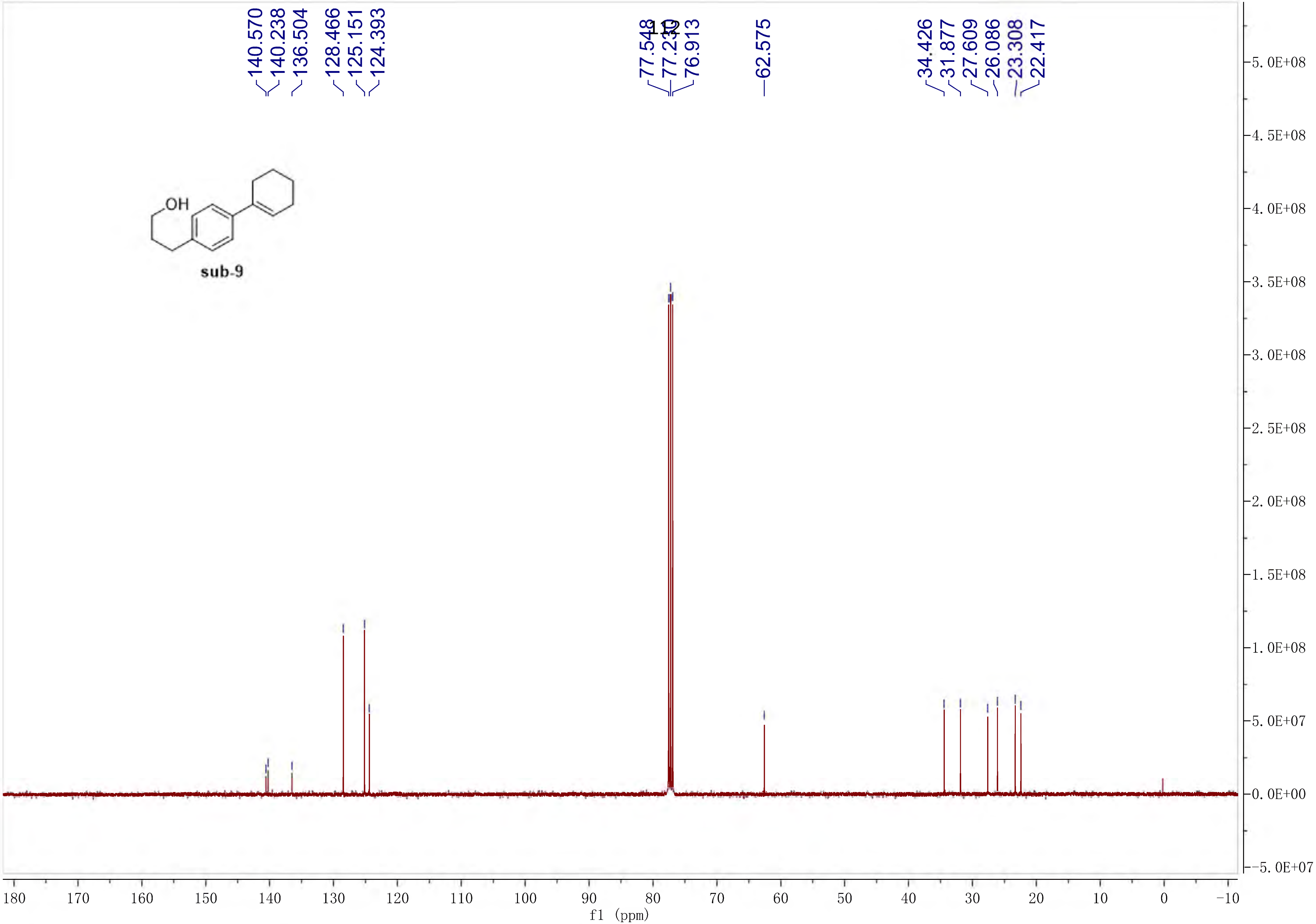

Supplementary Fig 40. ^{13}C NMR spectrum (400 MHz, $CDCl_3$, r.t.) of **sub-9**.

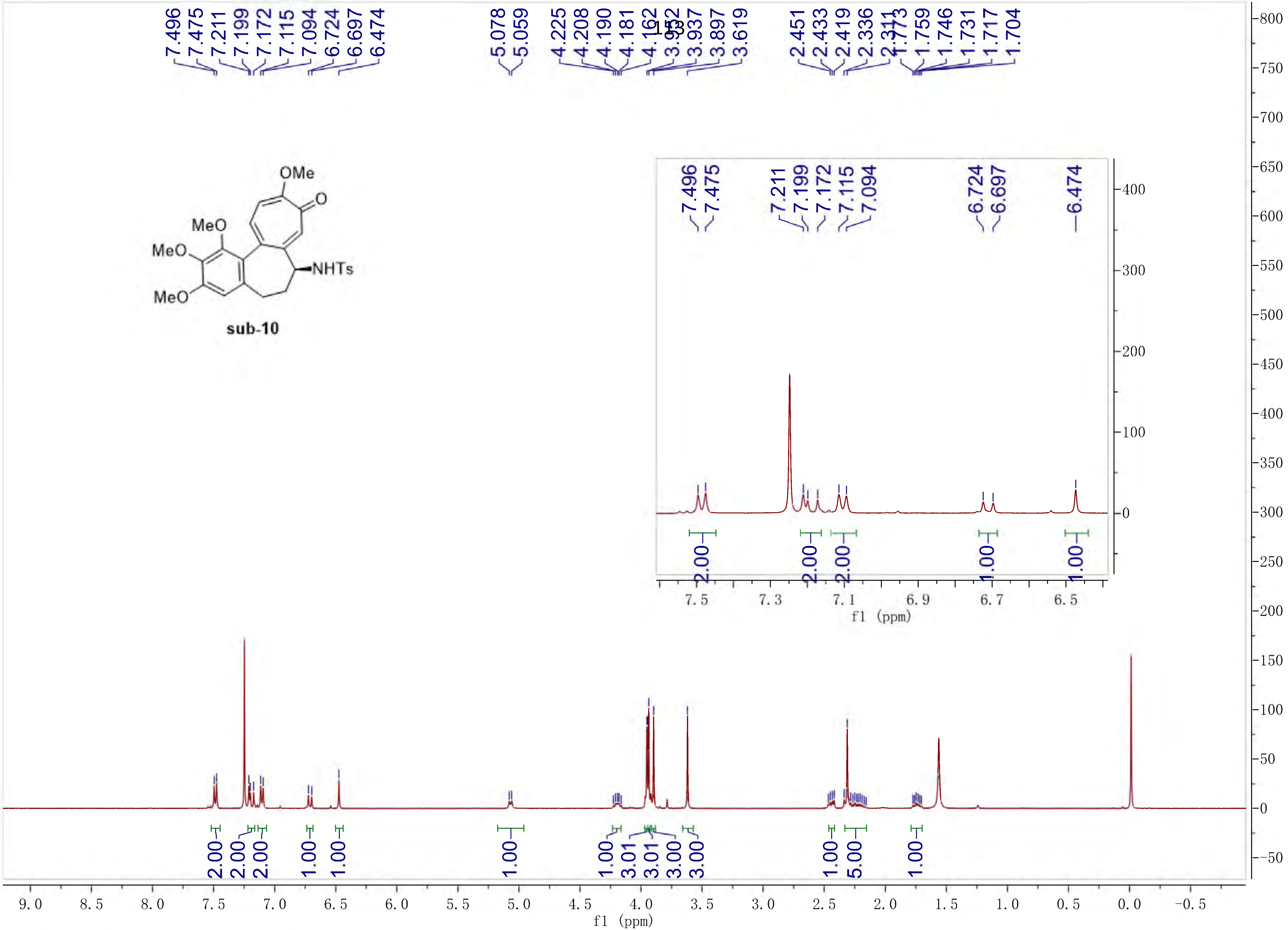

Supplementary Fig 41. ^{1}H NMR spectrum (400 MHz, $CDCl_3$, r.t.) of **sub-10**.

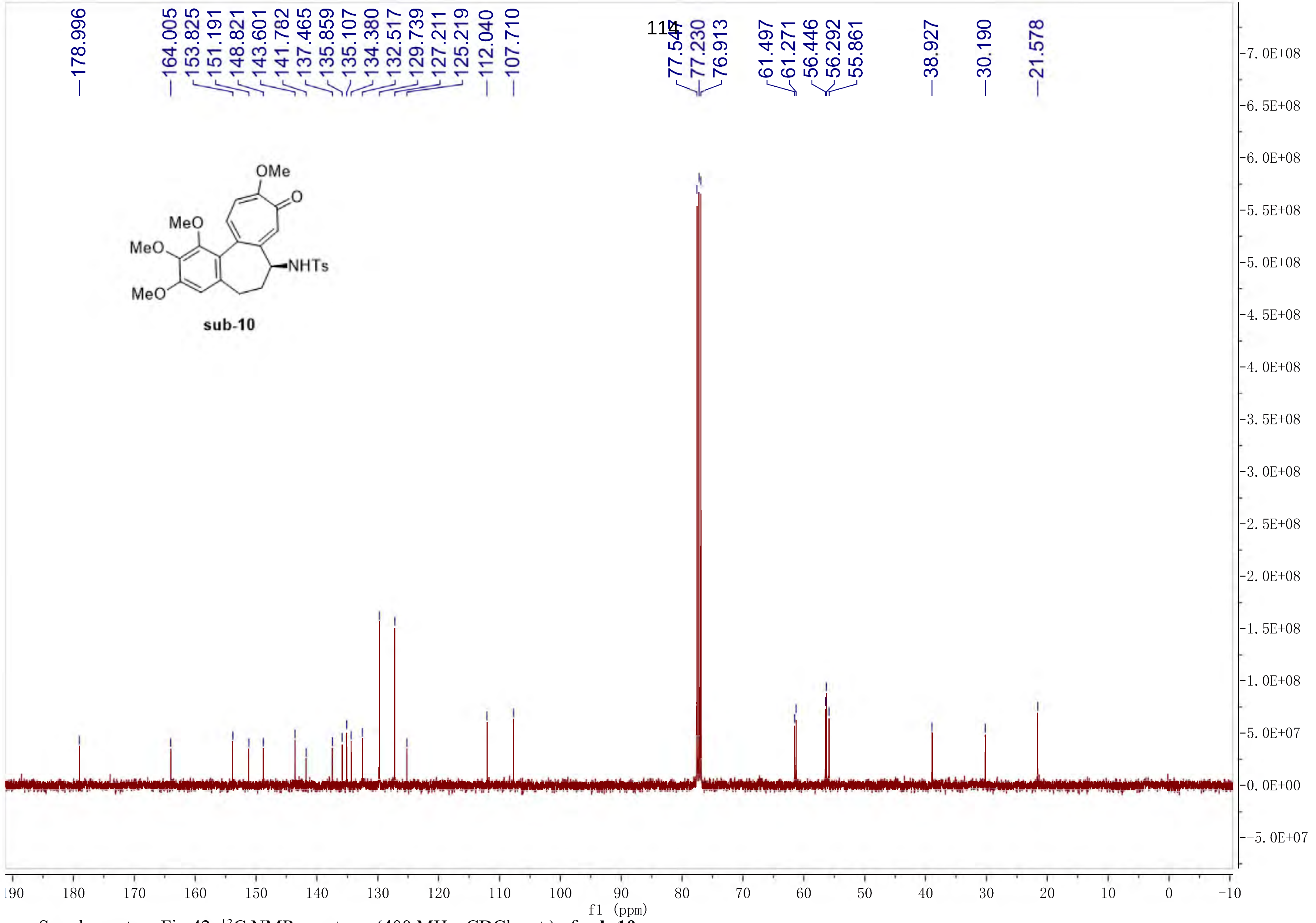

Supplementary Fig 42. ^{13}C NMR spectrum (400 MHz, $CDCl_3$, r.t.) of **sub-10**.

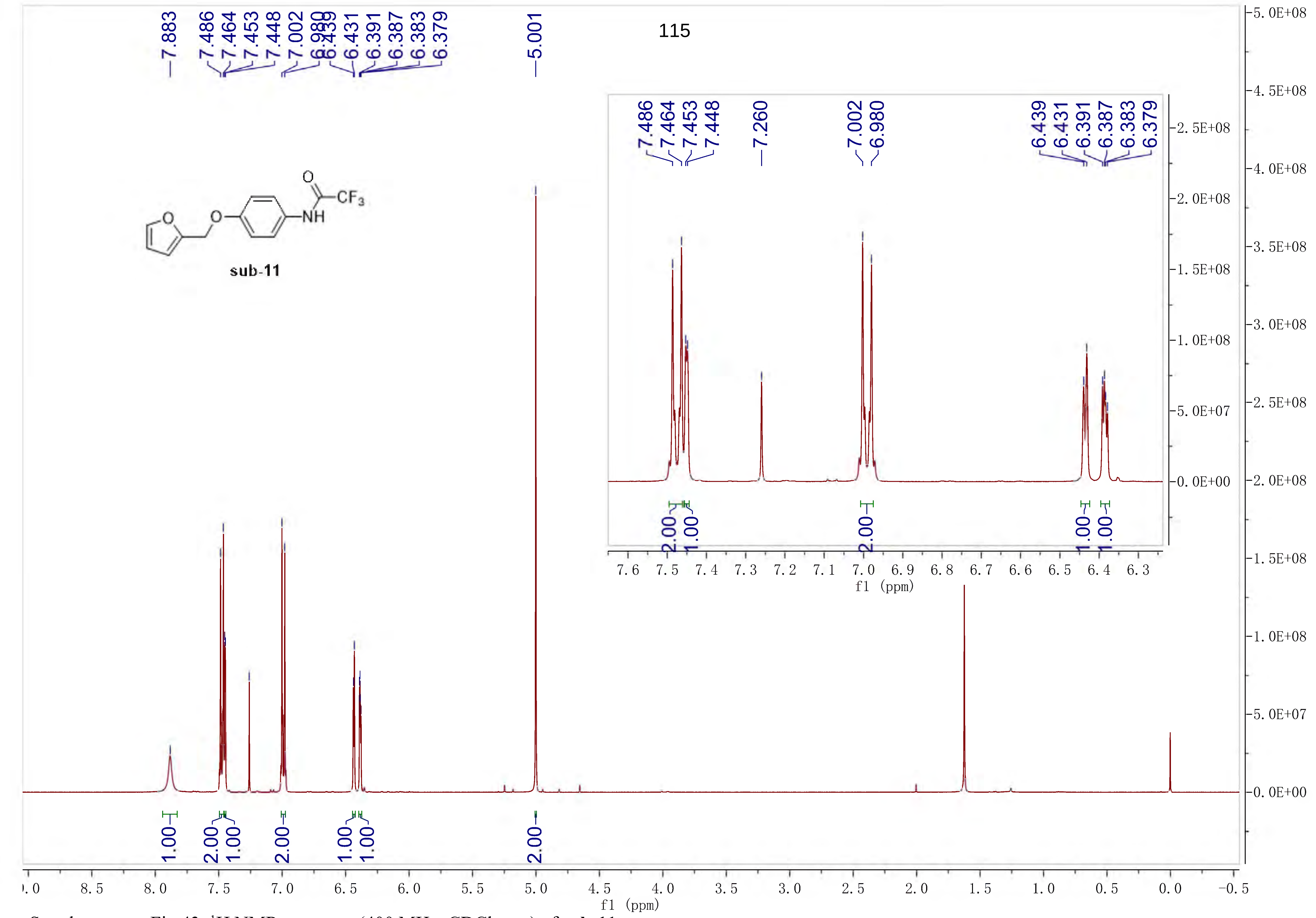

Supplementary Fig 43. ^{1}H NMR spectrum (400 MHz, $CDCl_3$, r.t.) of **sub-11**.

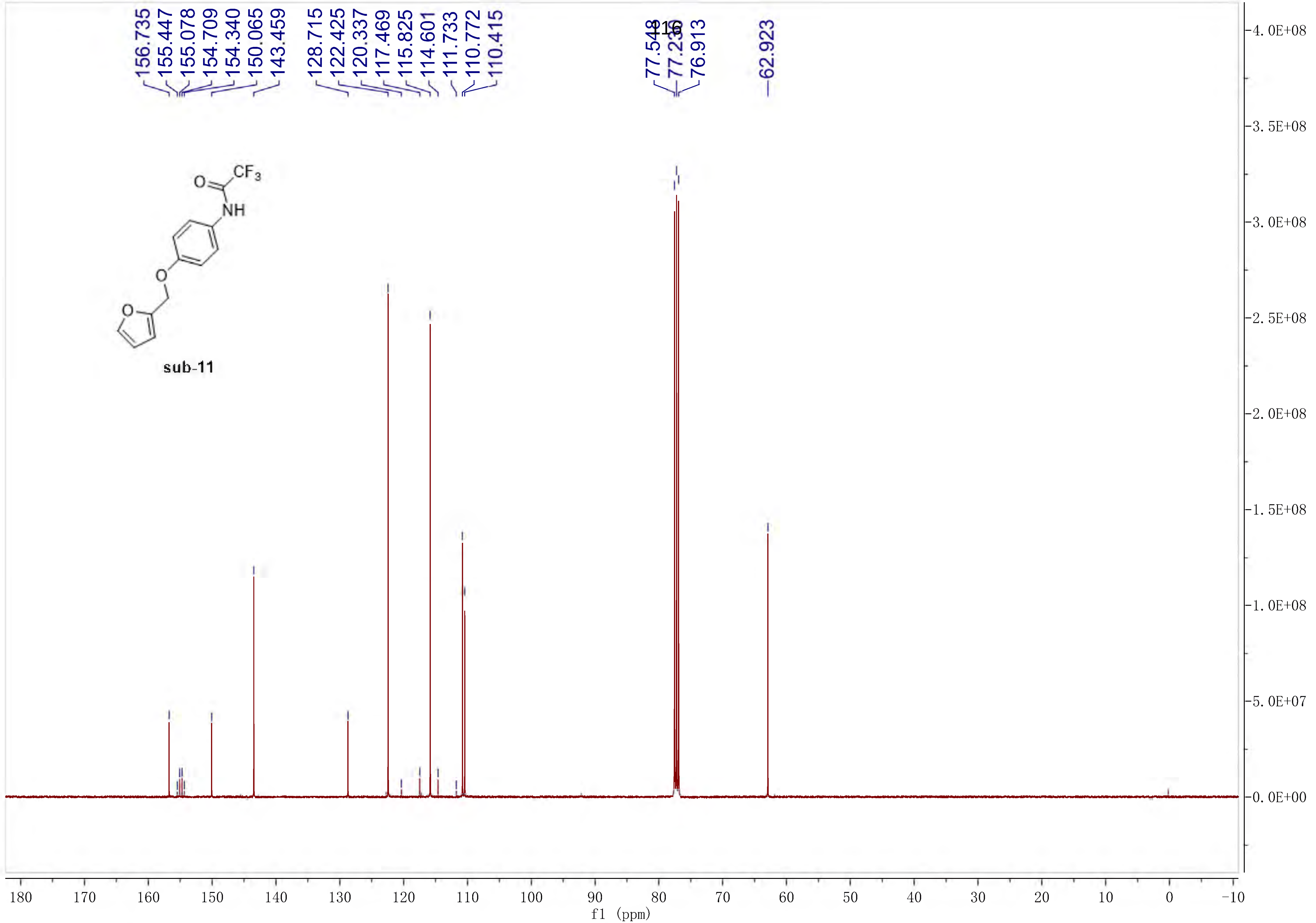

Supplementary Fig 44. ^{13}C NMR spectrum (400 MHz, $CDCl_3$, r.t.) of **sub-11**.

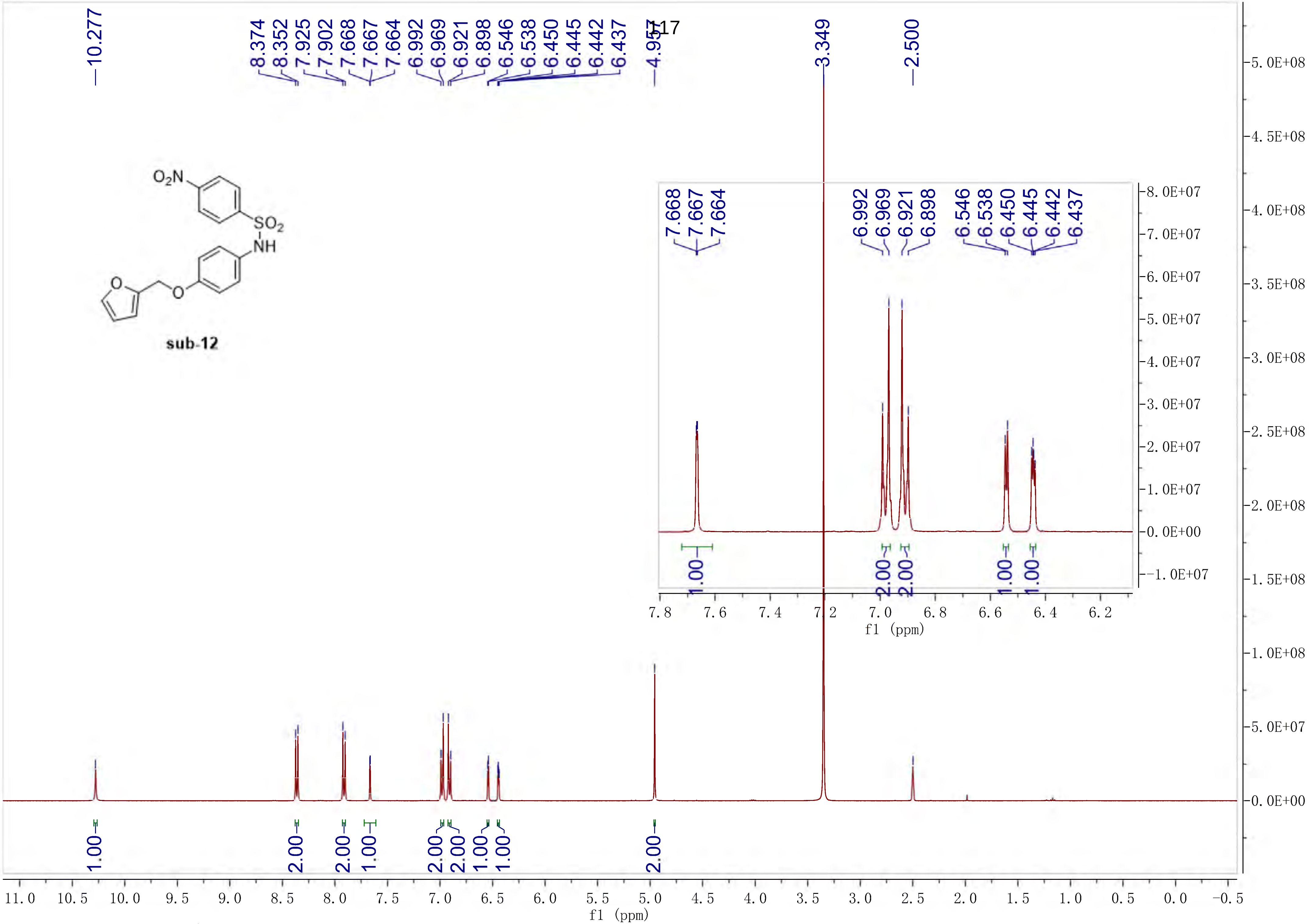

Supplementary Fig 45. ^{1}H NMR spectrum (400 MHz, $CDCl_3$, r.t.) of **sub-12**.

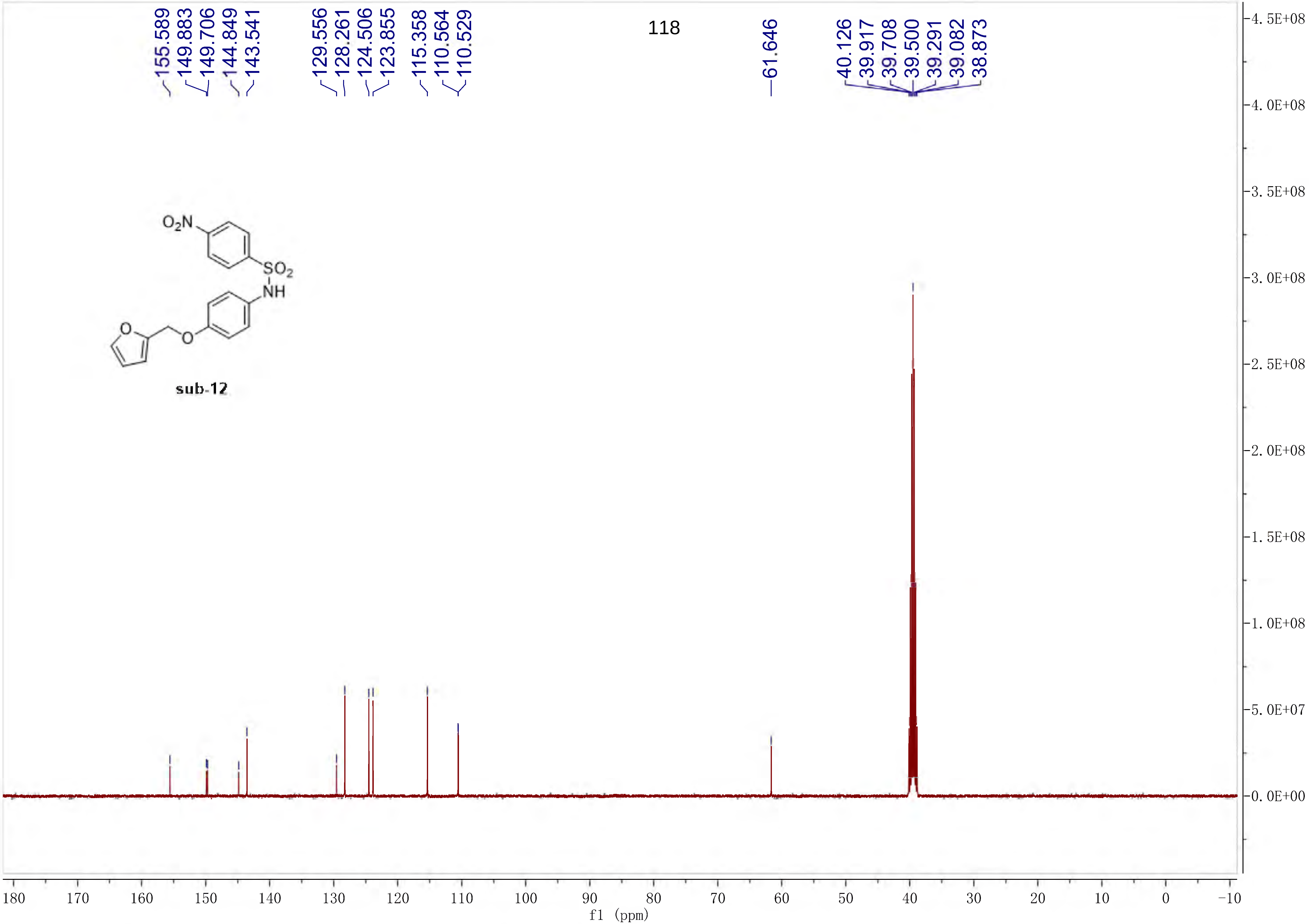

Supplementary Fig 46. ^{13}C NMR spectrum (400 MHz, $CDCl_3$, r.t.) of **sub-12**.

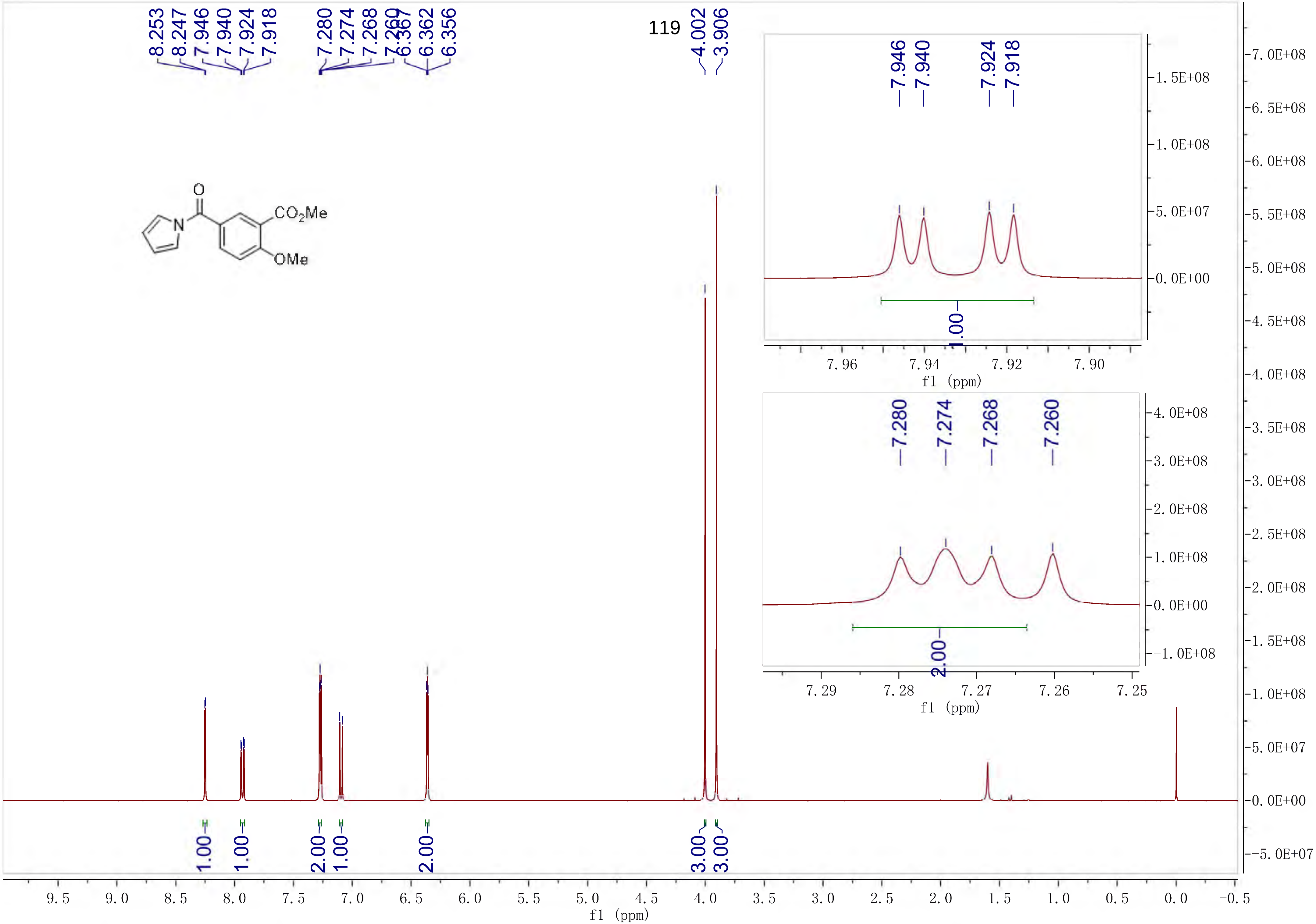

Supplementary Fig 47. ^{1}H NMR spectrum (400 MHz, $CDCl_3$, r.t.) of methyl 2-methoxy-5-(1*H*-pyrrole-1-carbonyl)benzoate.

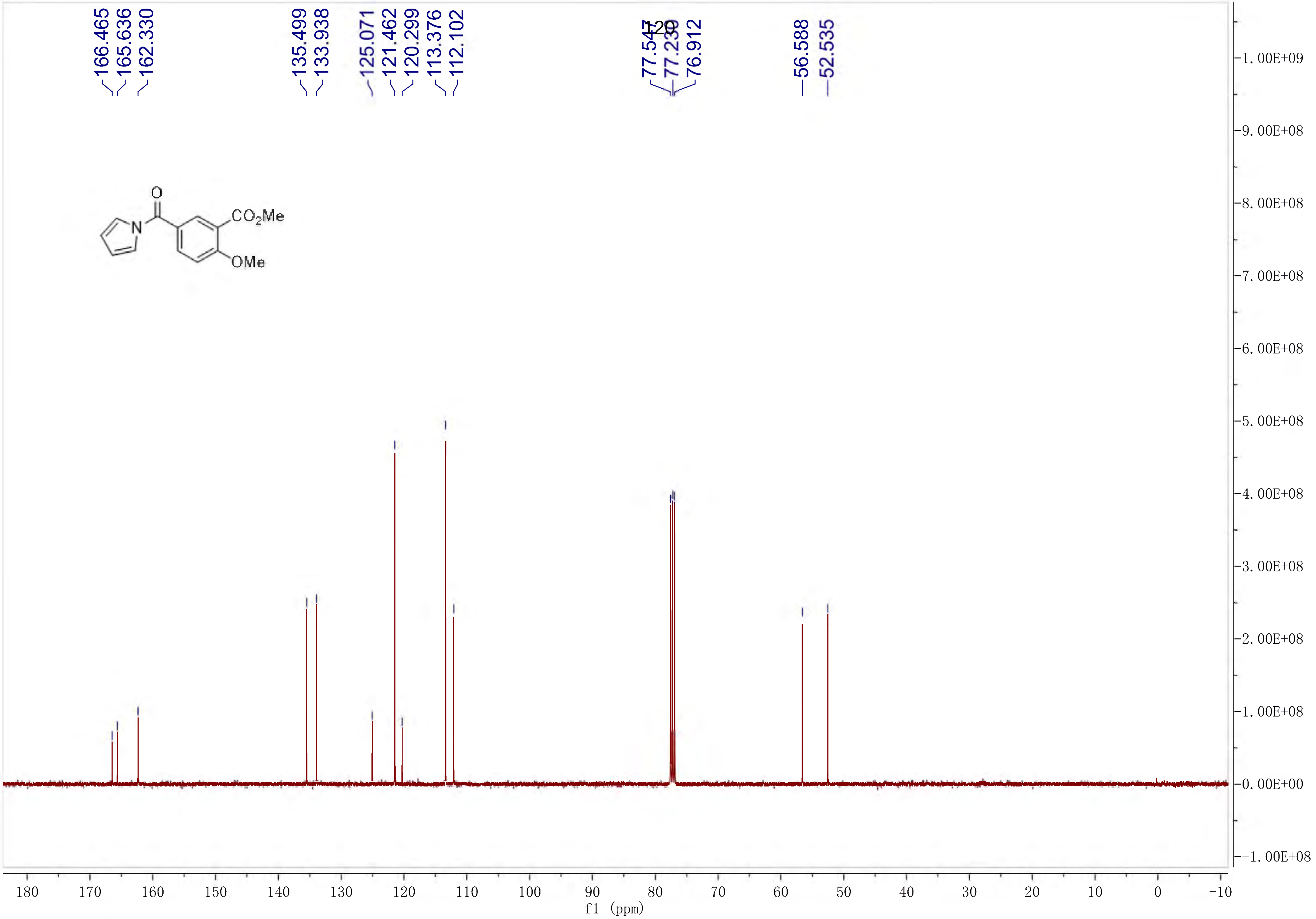

Supplementary Fig 48. ^{13}C NMR spectrum (400 MHz, $CDCl_3$, r.t.) of methyl 2-methoxy-5-(1*H*-pyrrole-1-carbonyl)benzoate.

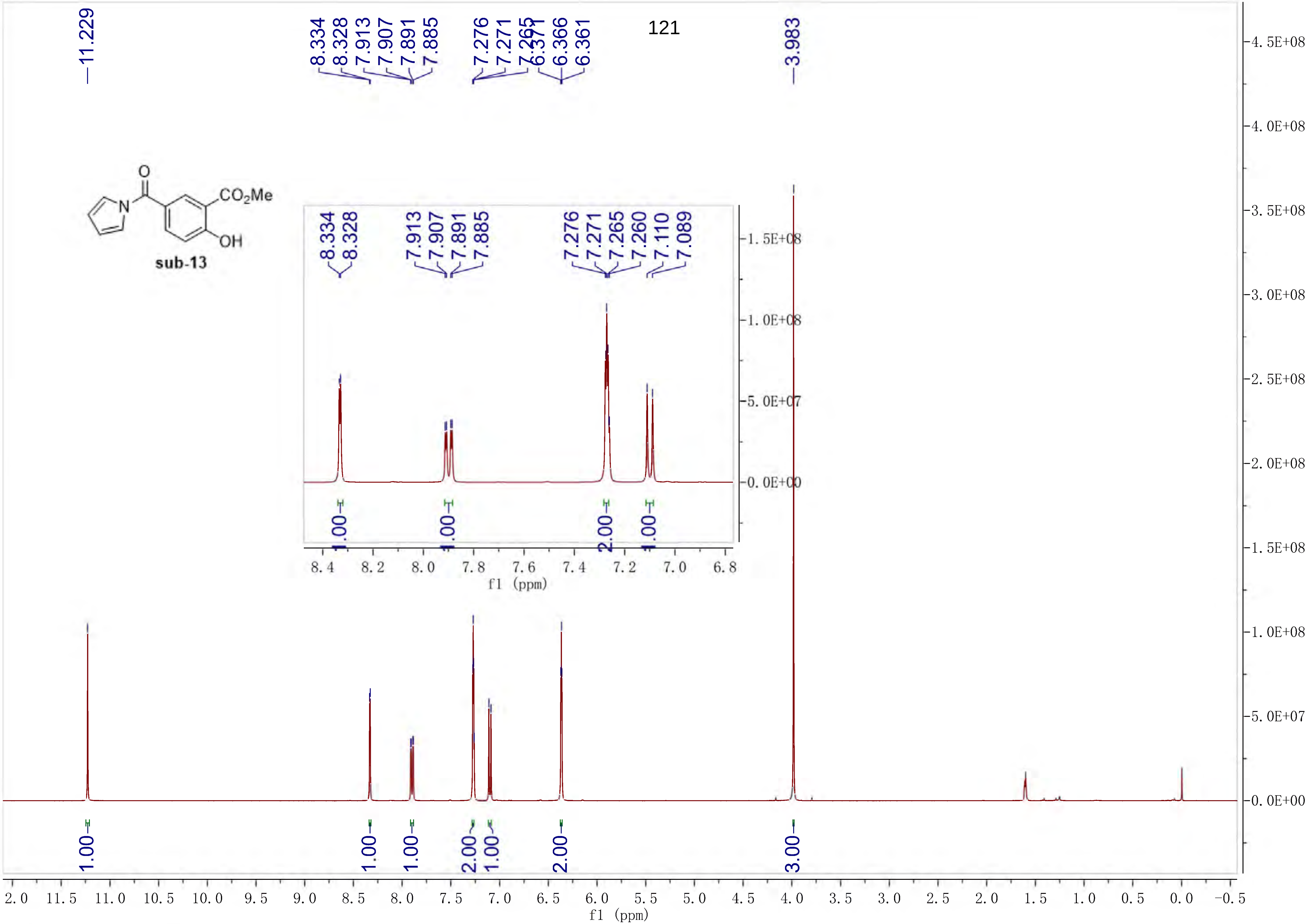

Supplementary Fig 49. ^{1}H NMR spectrum (400 MHz, $CDCl_3$, r.t.) of **sub-13**.

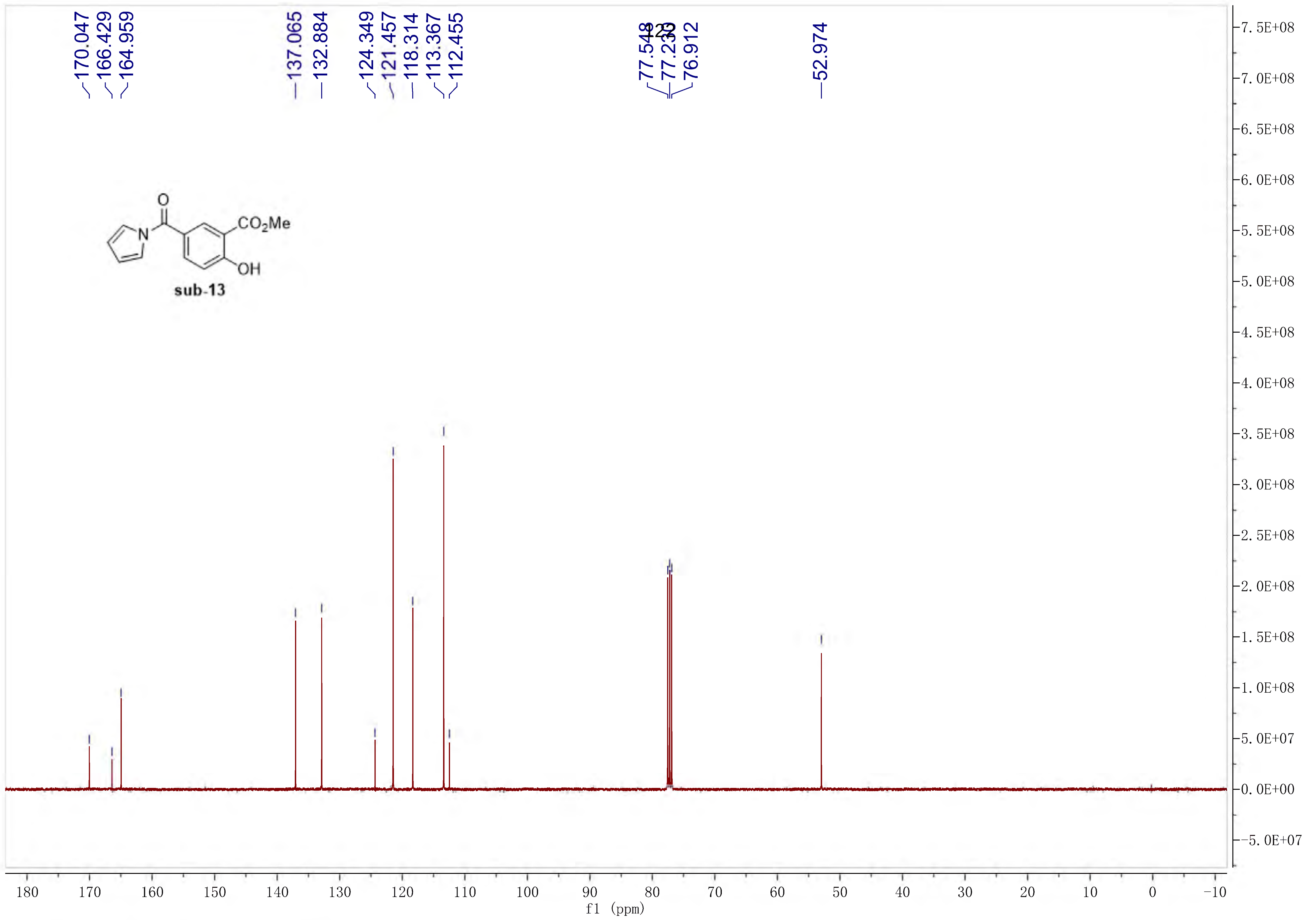

Supplementary Fig 50. ^{13}C NMR spectrum (400 MHz, $CDCl_3$, r.t.) of **sub-13**.

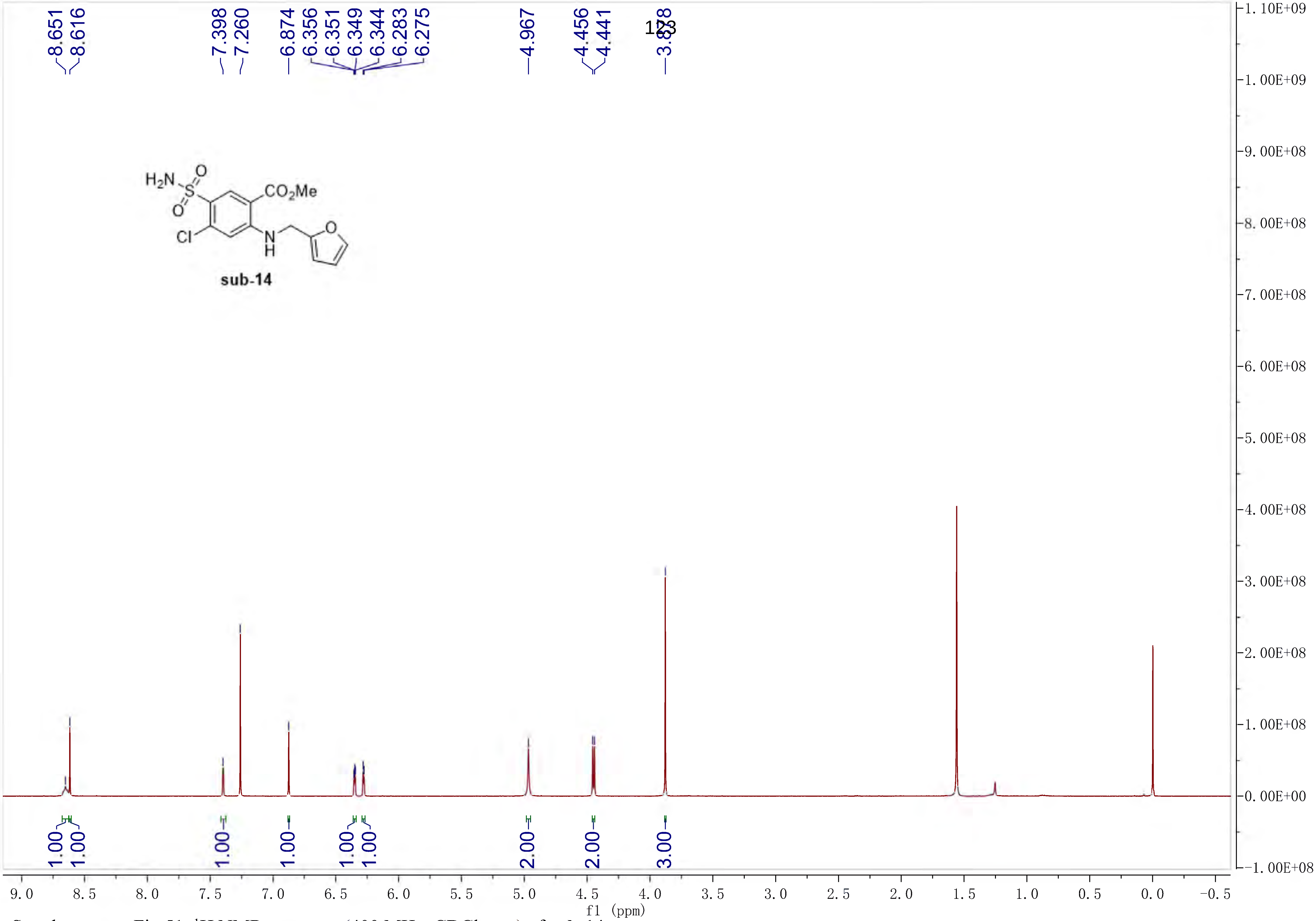

Supplementary Fig 51. ^{1}H NMR spectrum (400 MHz, $CDCl_3$, r.t.) of **sub-14**.

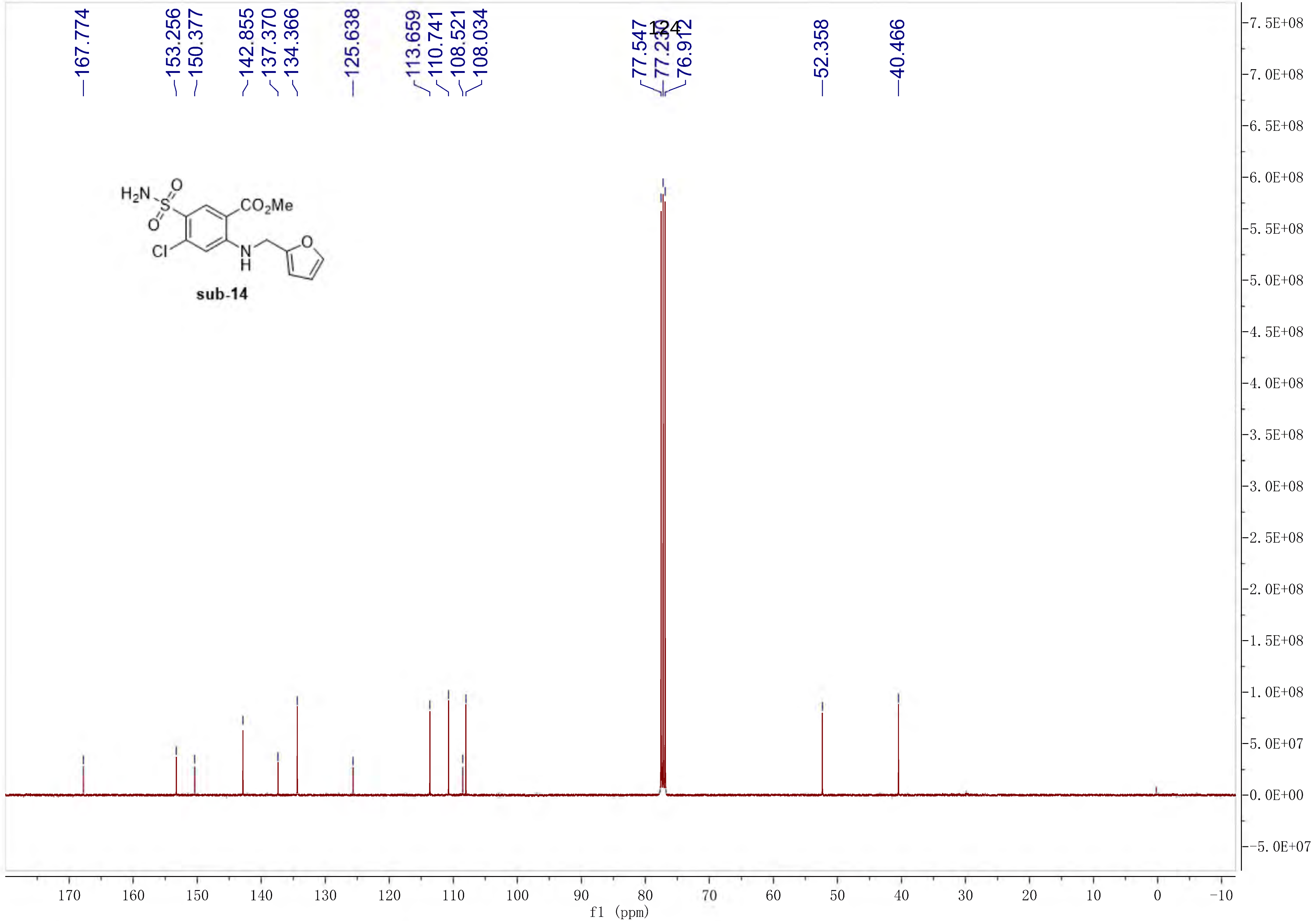

Supplementary Fig 52. ^{13}C NMR spectrum (400 MHz, $CDCl_3$, r.t.) of **sub-14**.

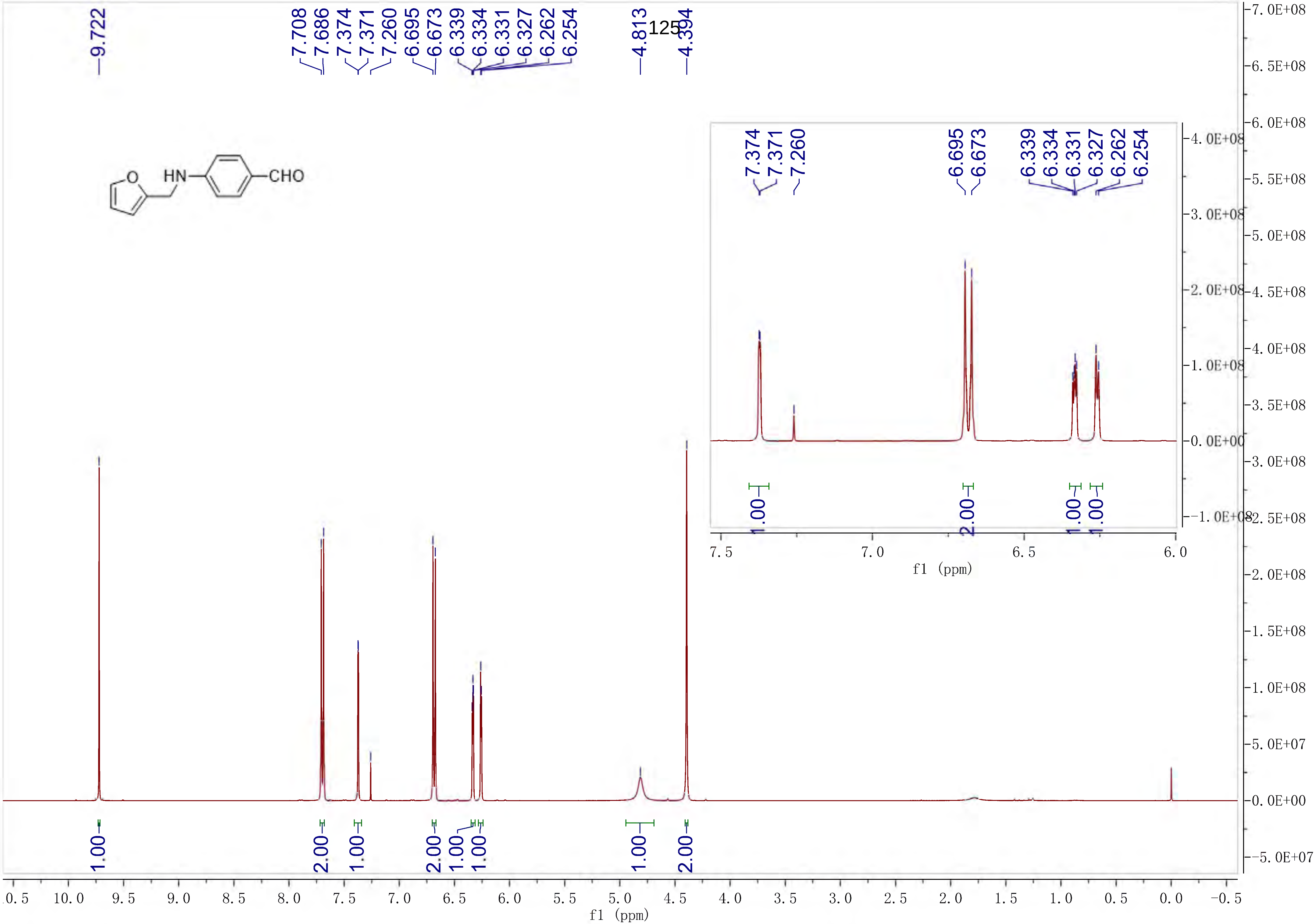

Supplementary Fig 53. ^{1}H NMR spectrum (400 MHz, $CDCl_3$, r.t.) of 4-((furan-2-ylmethyl)amino)benzaldehyde.

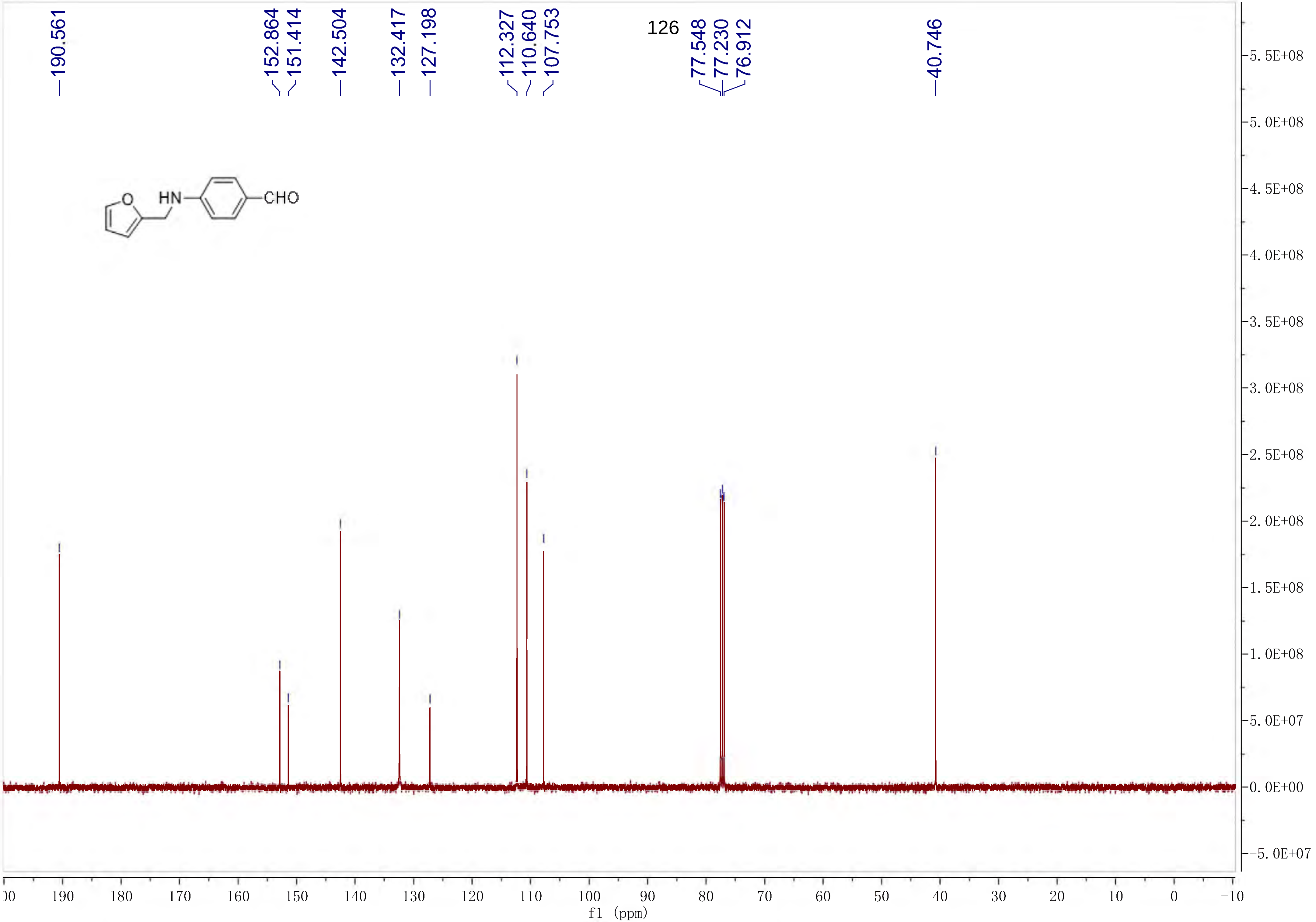

Supplementary Fig 54. ^{13}C NMR spectrum (400 MHz, $CDCl_3$, r.t.) of 4-((furan-2-ylmethyl)amino)benzaldehyde.

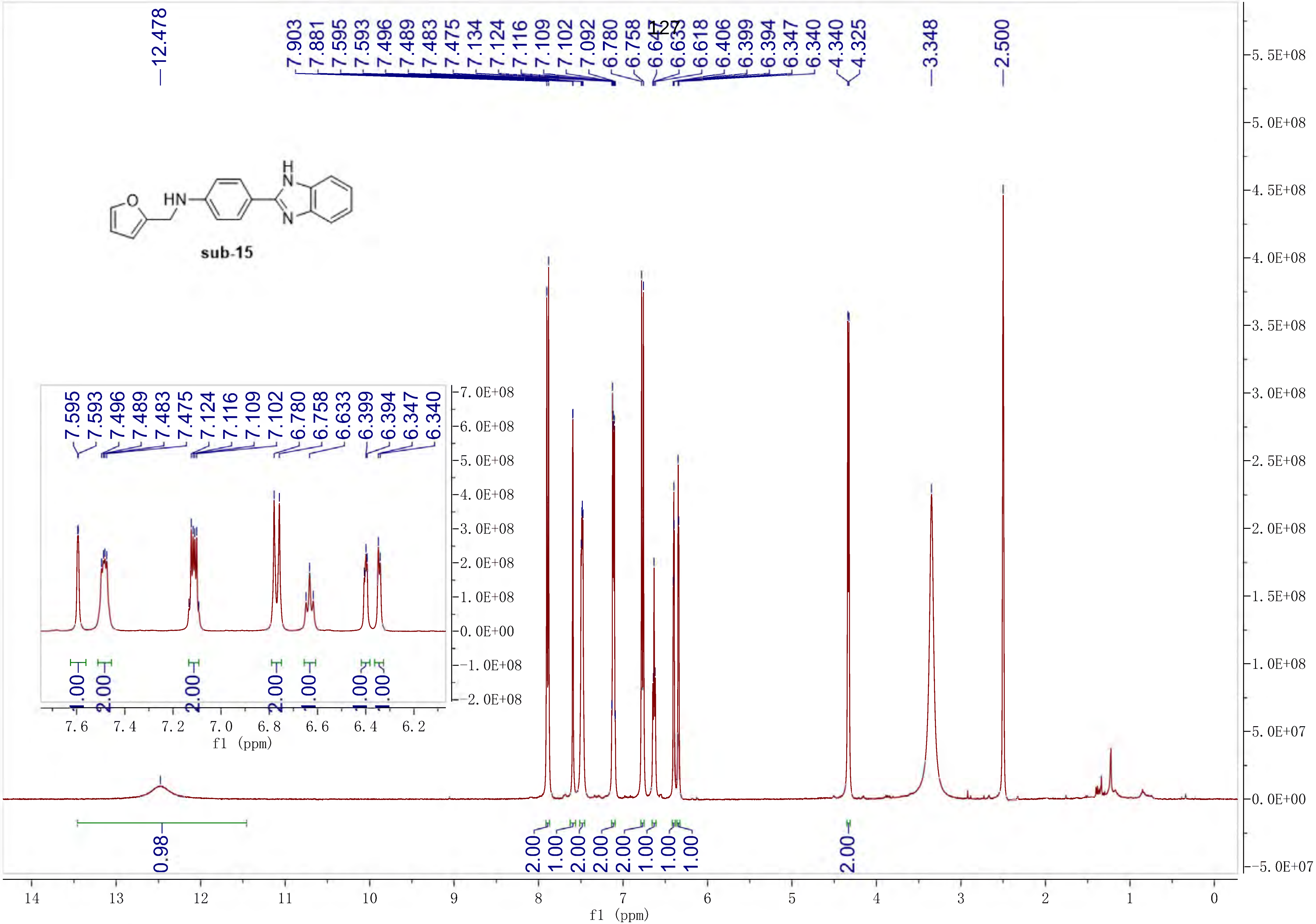

Supplementary Fig 55. ^{1}H NMR spectrum (400 MHz, $CDCl_3$, r.t.) of **sub-15**.

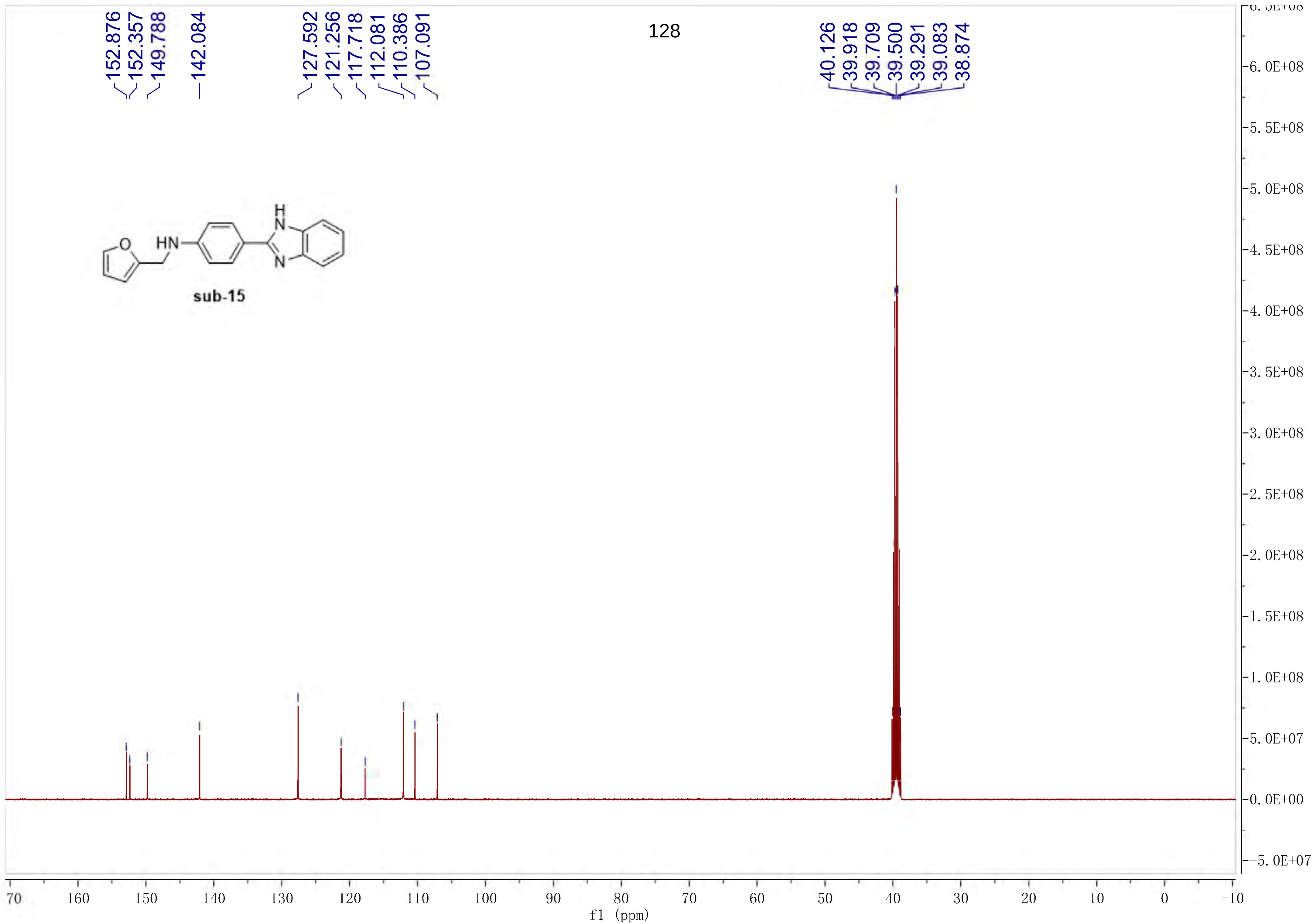

Supplementary Fig 56. ^{13}C NMR spectrum (400 MHz, $CDCl_3$, r.t.) of **sub-15**.

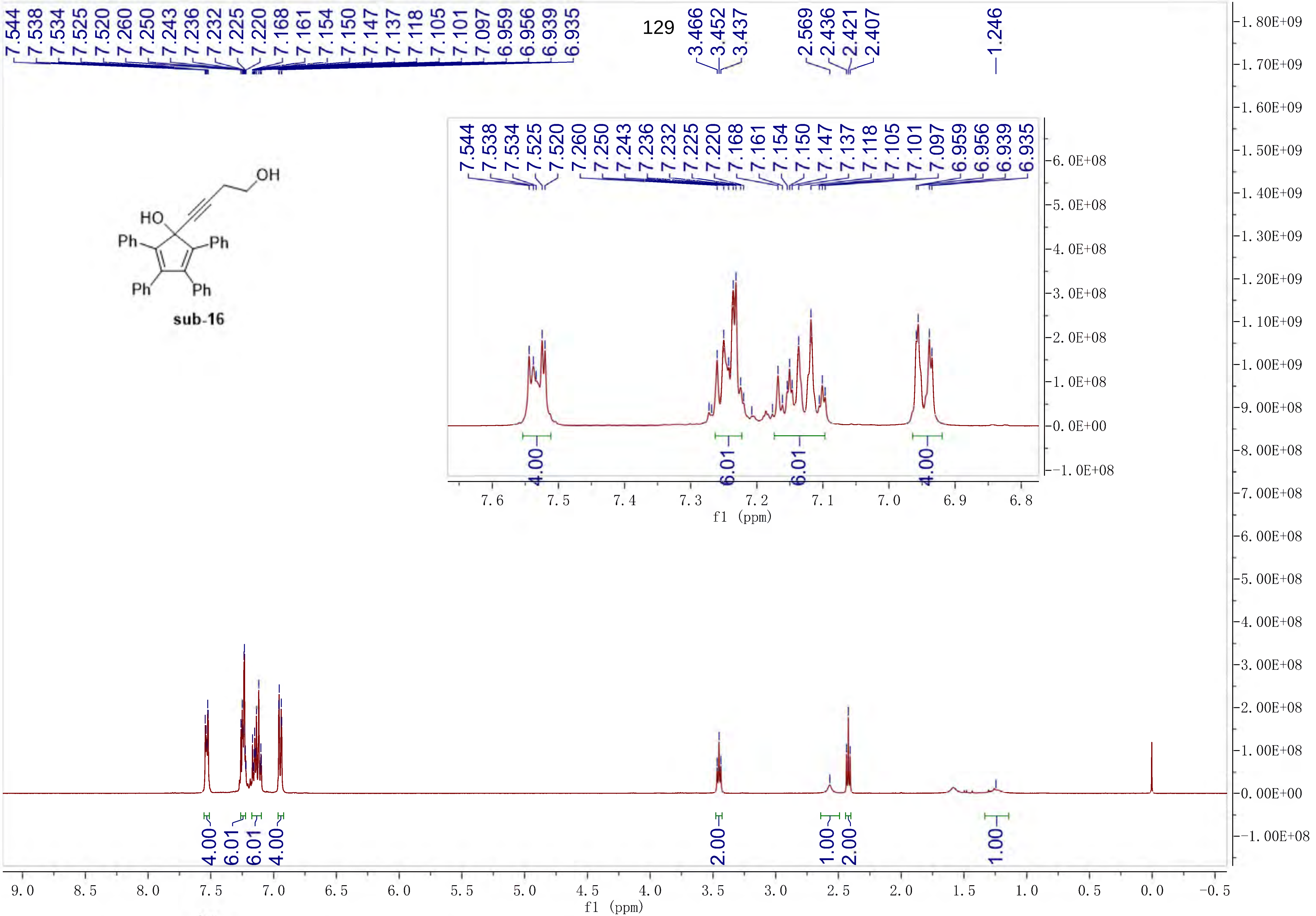

Supplementary Fig 57. ^{1}H NMR spectrum (400 MHz, $CDCl_3$, r.t.) of **sub-16**.

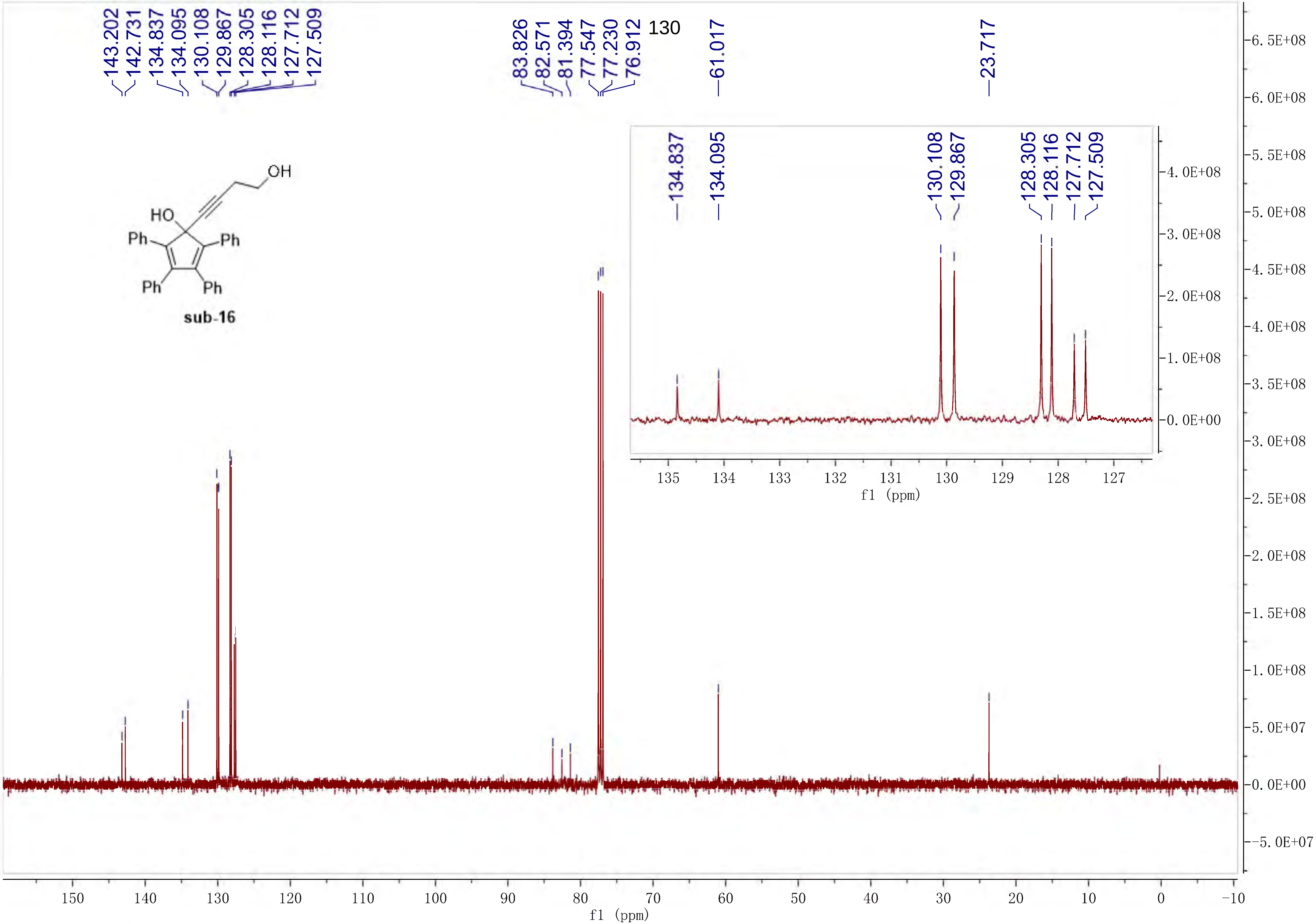

Supplementary Fig 58. ^{13}C NMR spectrum (400 MHz, $CDCl_3$, r.t.) of **sub-16**.

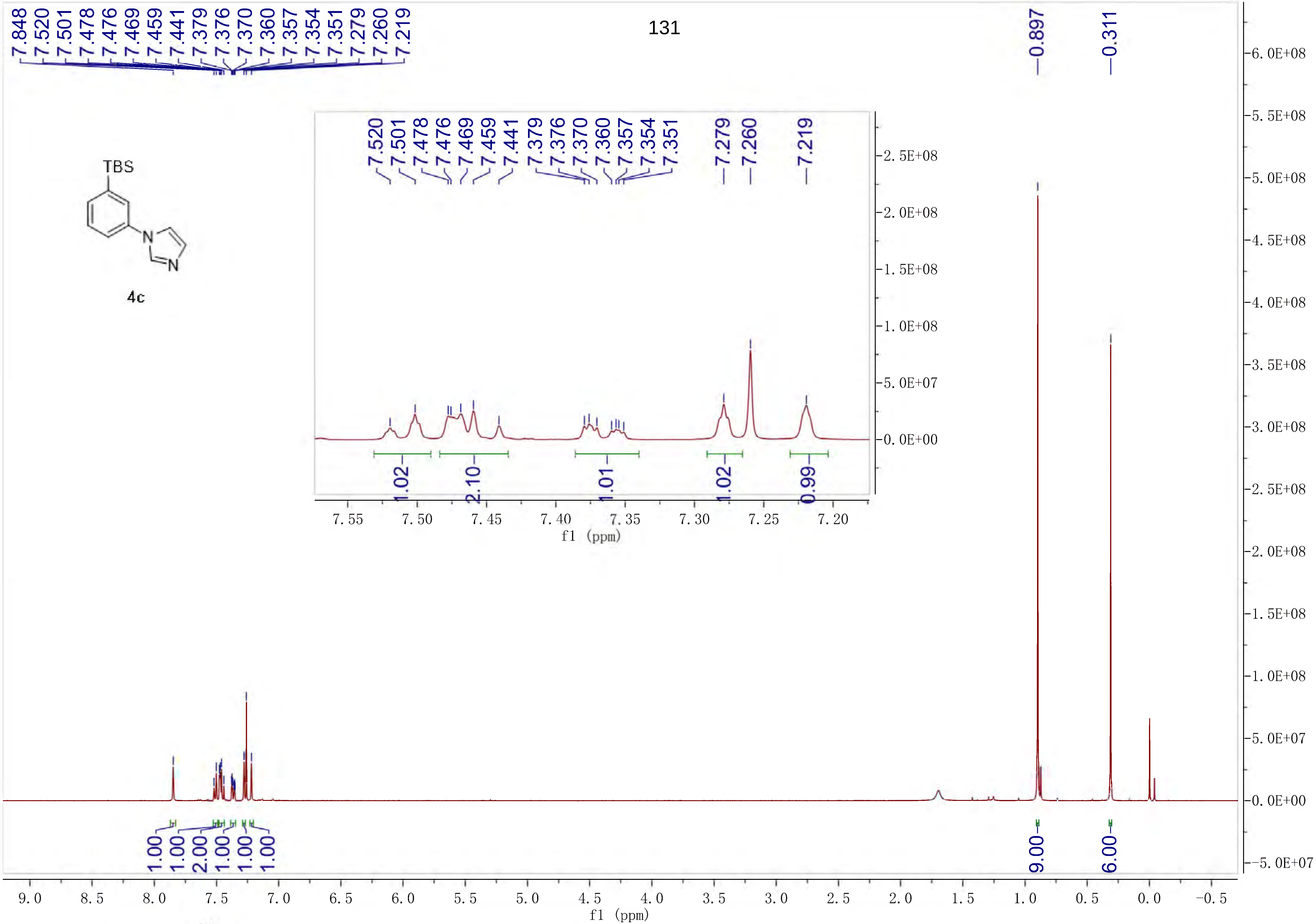

Supplementary Fig 59. ^{1}H NMR spectrum (400 MHz, $CDCl_3$, r.t.) of **4c**.

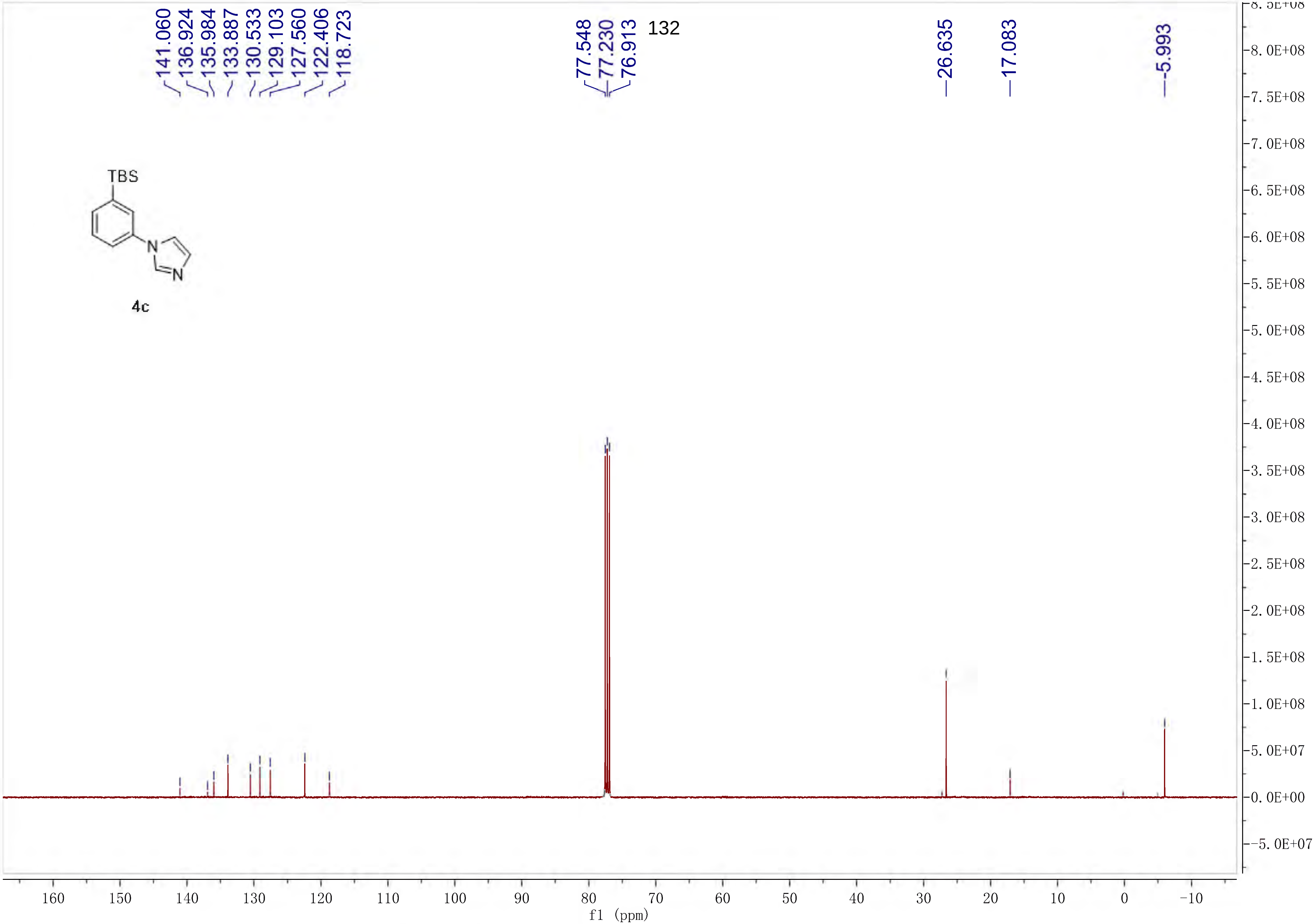

Supplementary Fig 60. ^{13}C NMR spectrum (400 MHz, $CDCl_3$, r.t.) of **4c**.

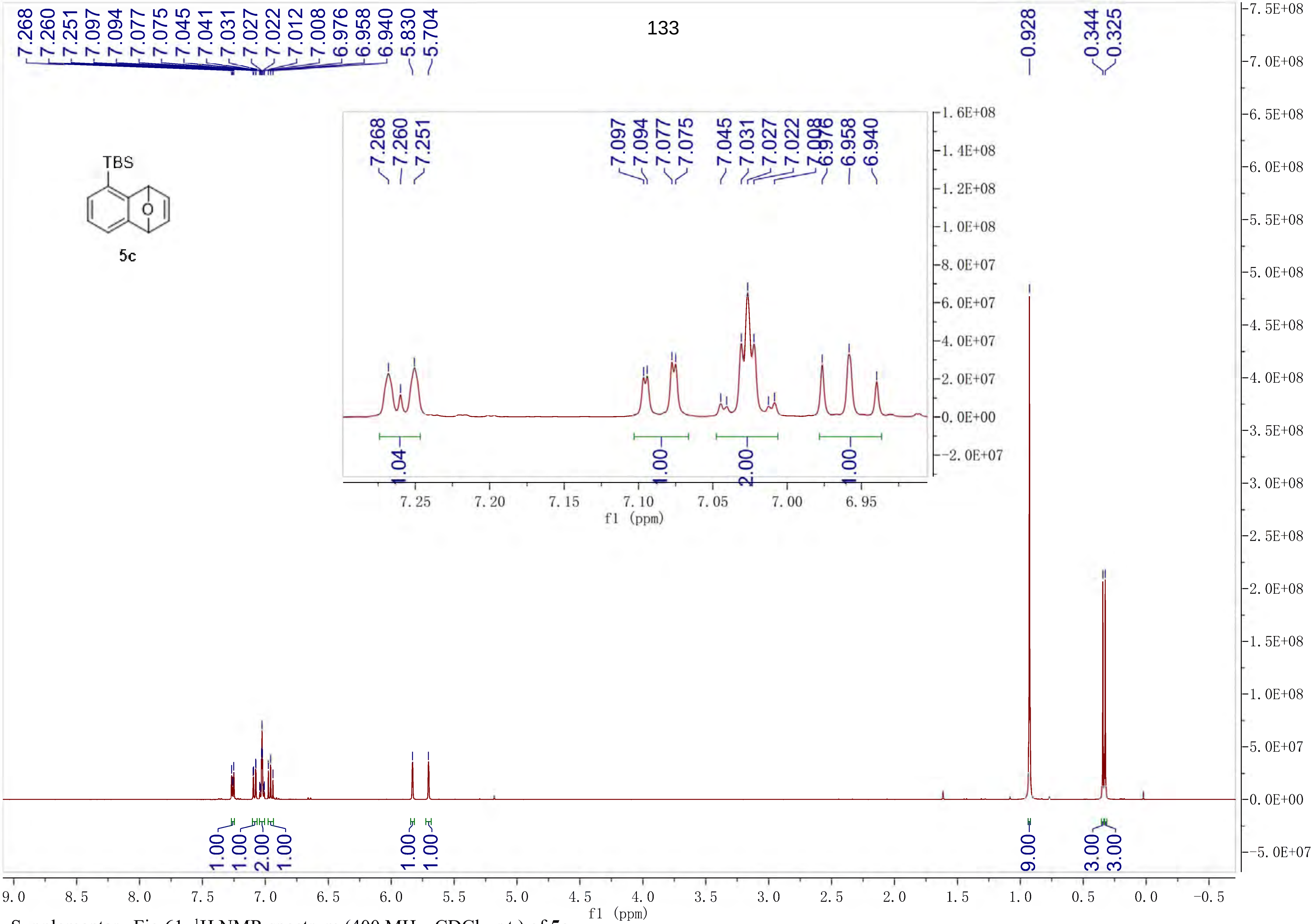

Supplementary Fig 61. ^{1}H NMR spectrum (400 MHz, $CDCl_3$, r.t.) of **5c**.

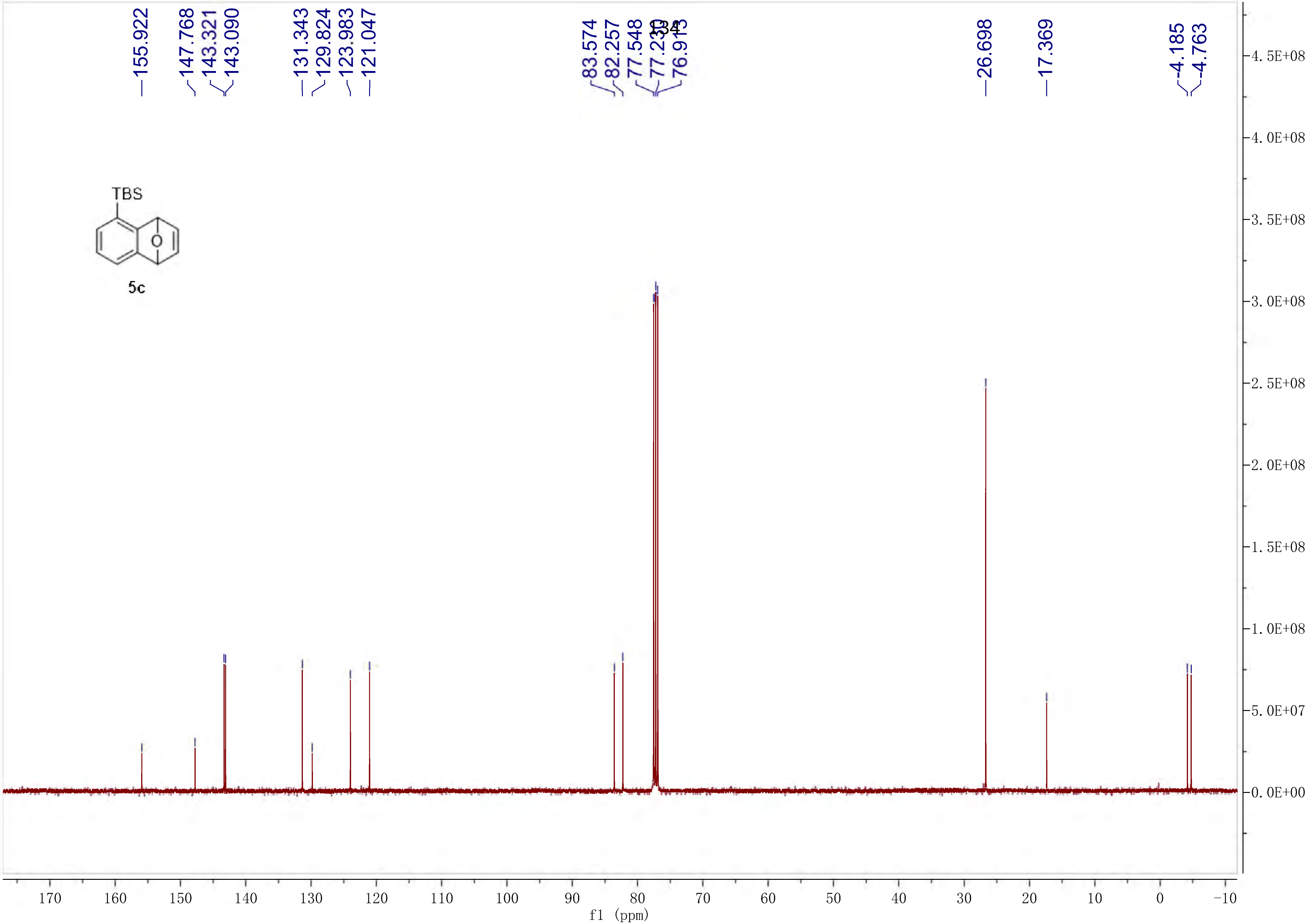

Supplementary Fig 62. ^{13}C NMR spectrum (400 MHz, $CDCl_3$, r.t.) of **5c**.

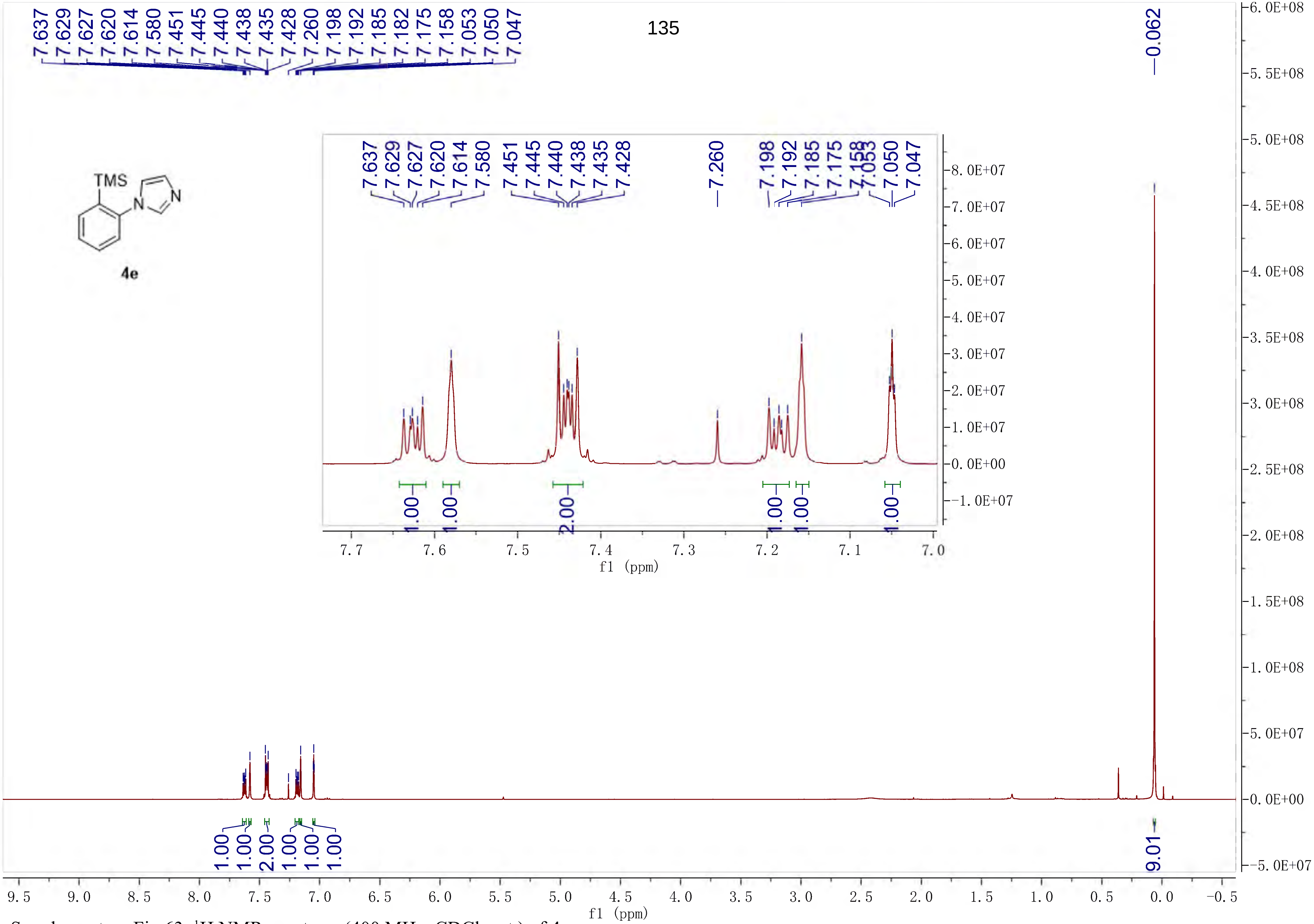

4e

Supplementary Fig 63. ^{1}H NMR spectrum (400 MHz, $CDCl_3$, r.t.) of **4e**.

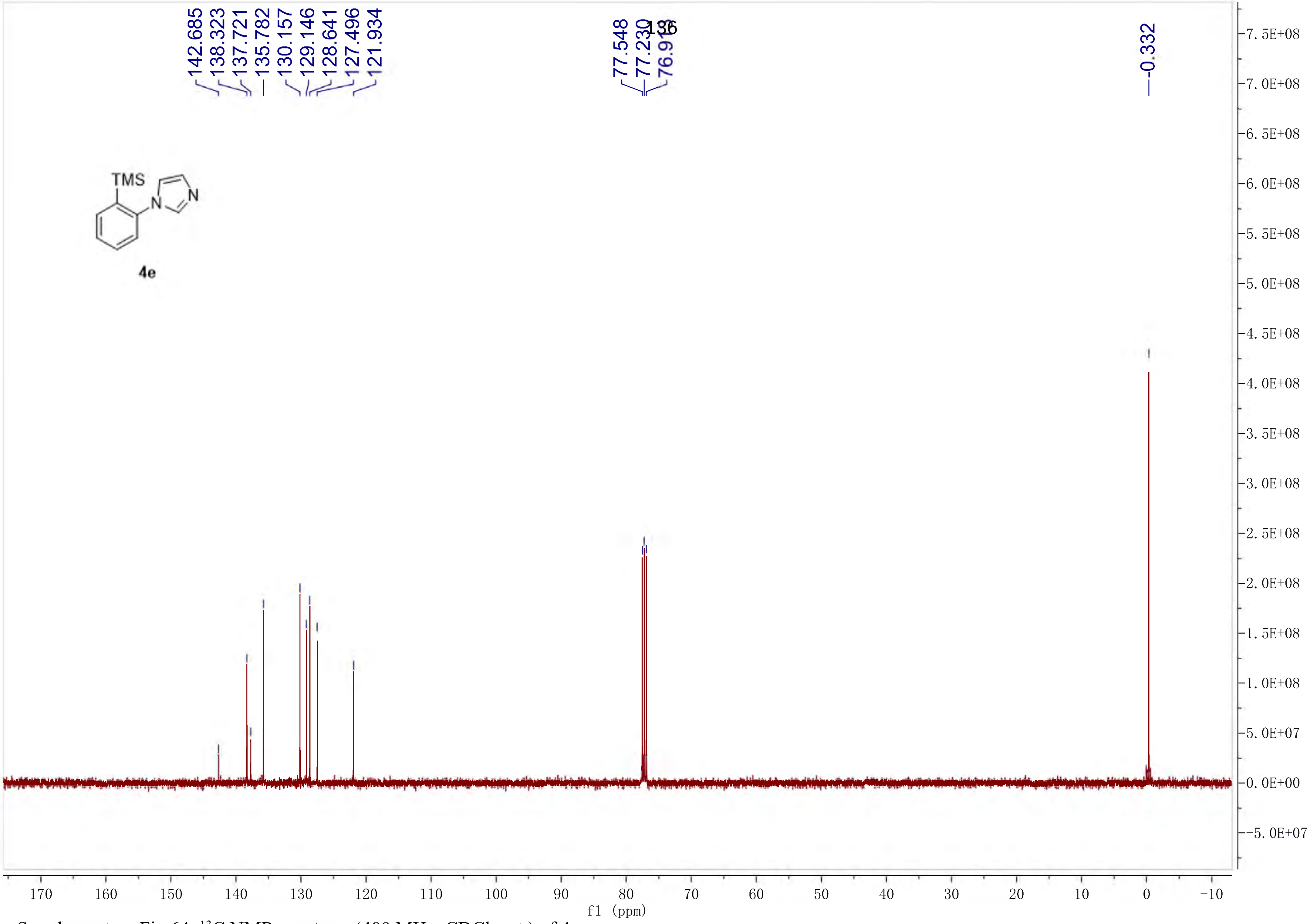

Supplementary Fig 64. ^{13}C NMR spectrum (400 MHz, $CDCl_3$, r.t.) of **4e**.

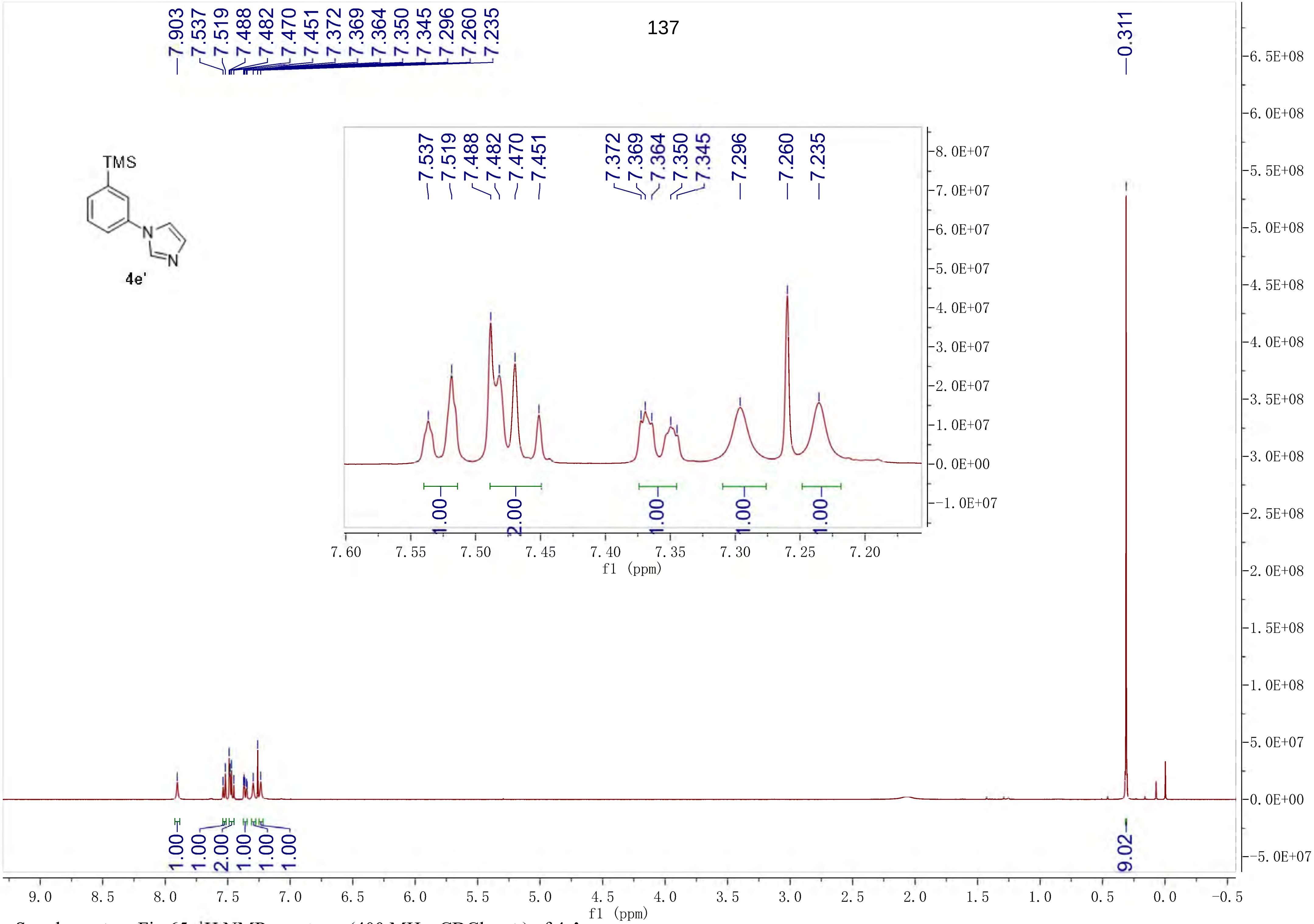

Supplementary Fig 65. ^{1}H NMR spectrum (400 MHz, $CDCl_3$, r.t.) of **4e'**.

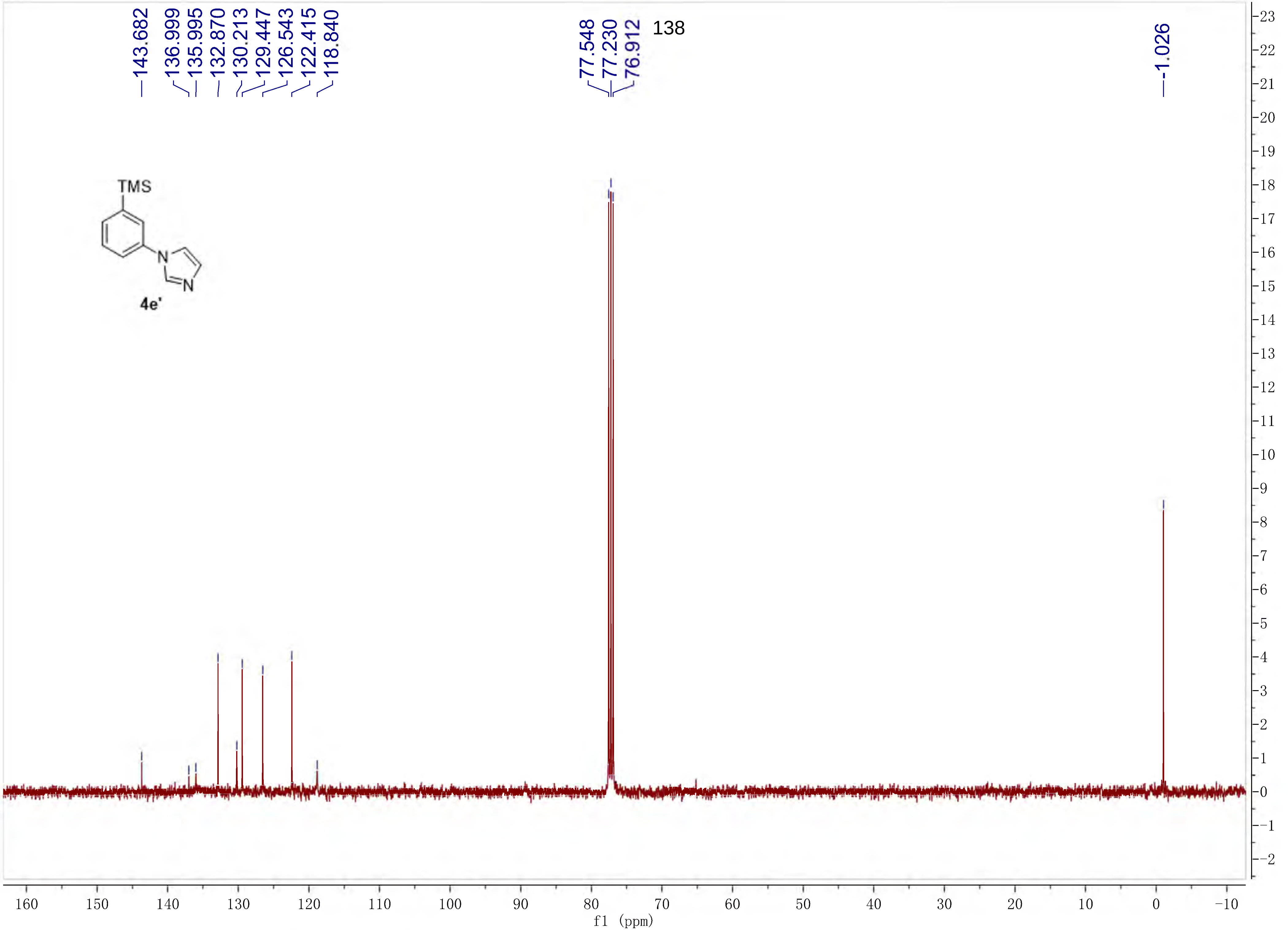

Supplementary Fig 66. ^{13}C NMR spectrum (400 MHz, $CDCl_3$, r.t.) of **4e’**.

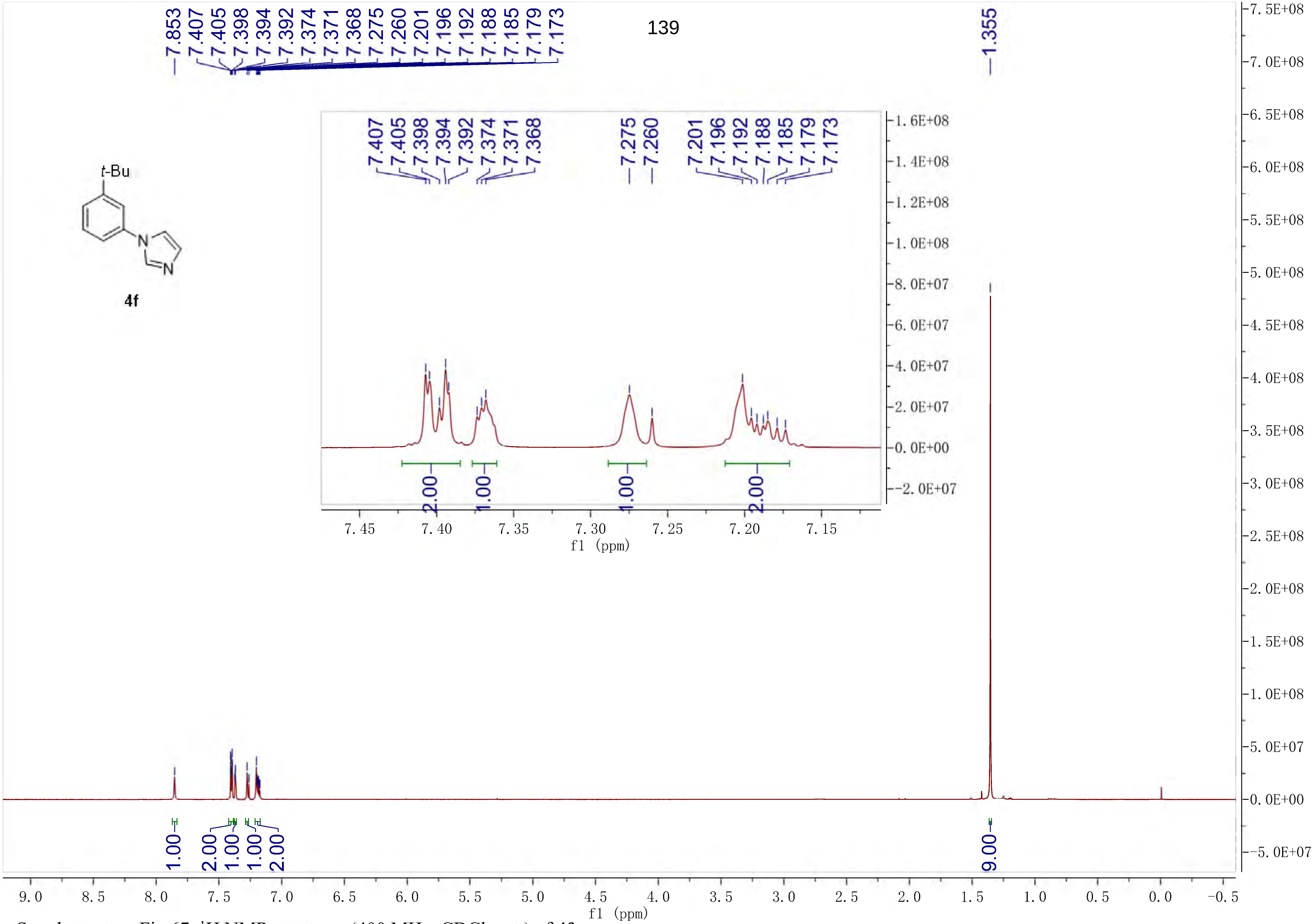

Supplementary Fig 67. ^{1}H NMR spectrum (400 MHz, $CDCl_3$, r.t.) of **4f**.

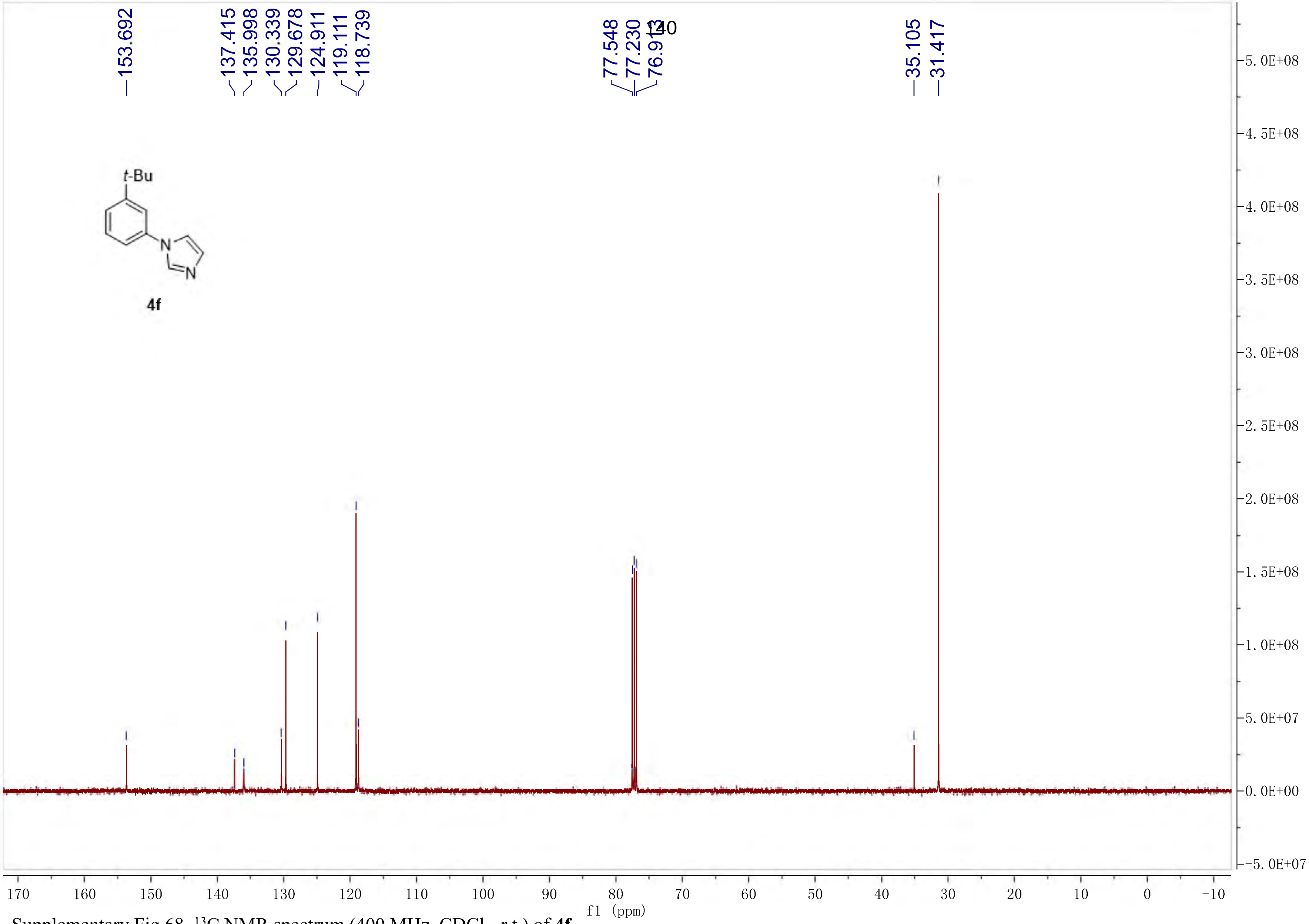

Supplementary Fig 68. ^{13}C NMR spectrum (400 MHz, $CDCl_3$, r.t.) of **4f**.

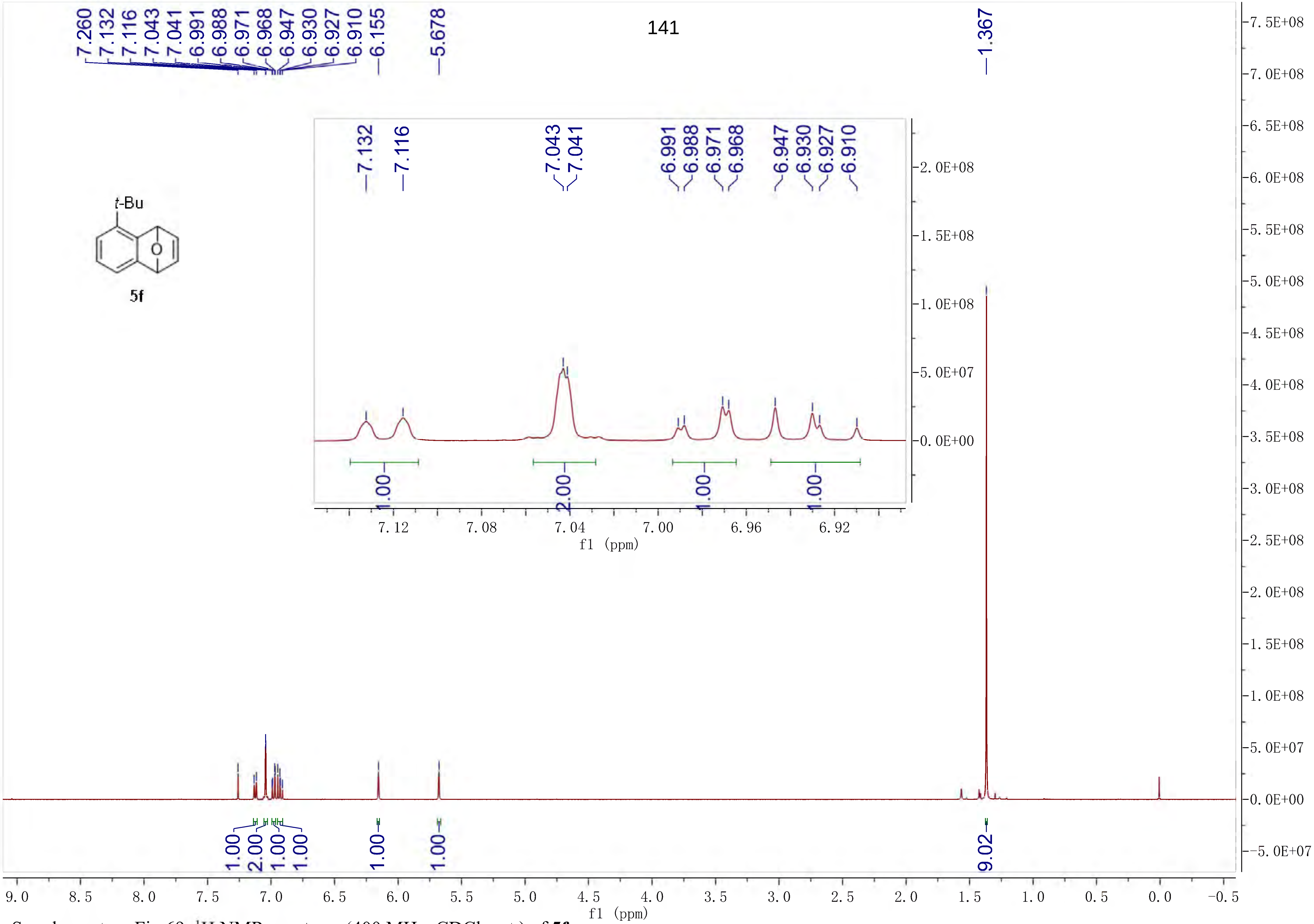

Supplementary Fig 69. ^{1}H NMR spectrum (400 MHz, $CDCl_3$, r.t.) of **5f**.

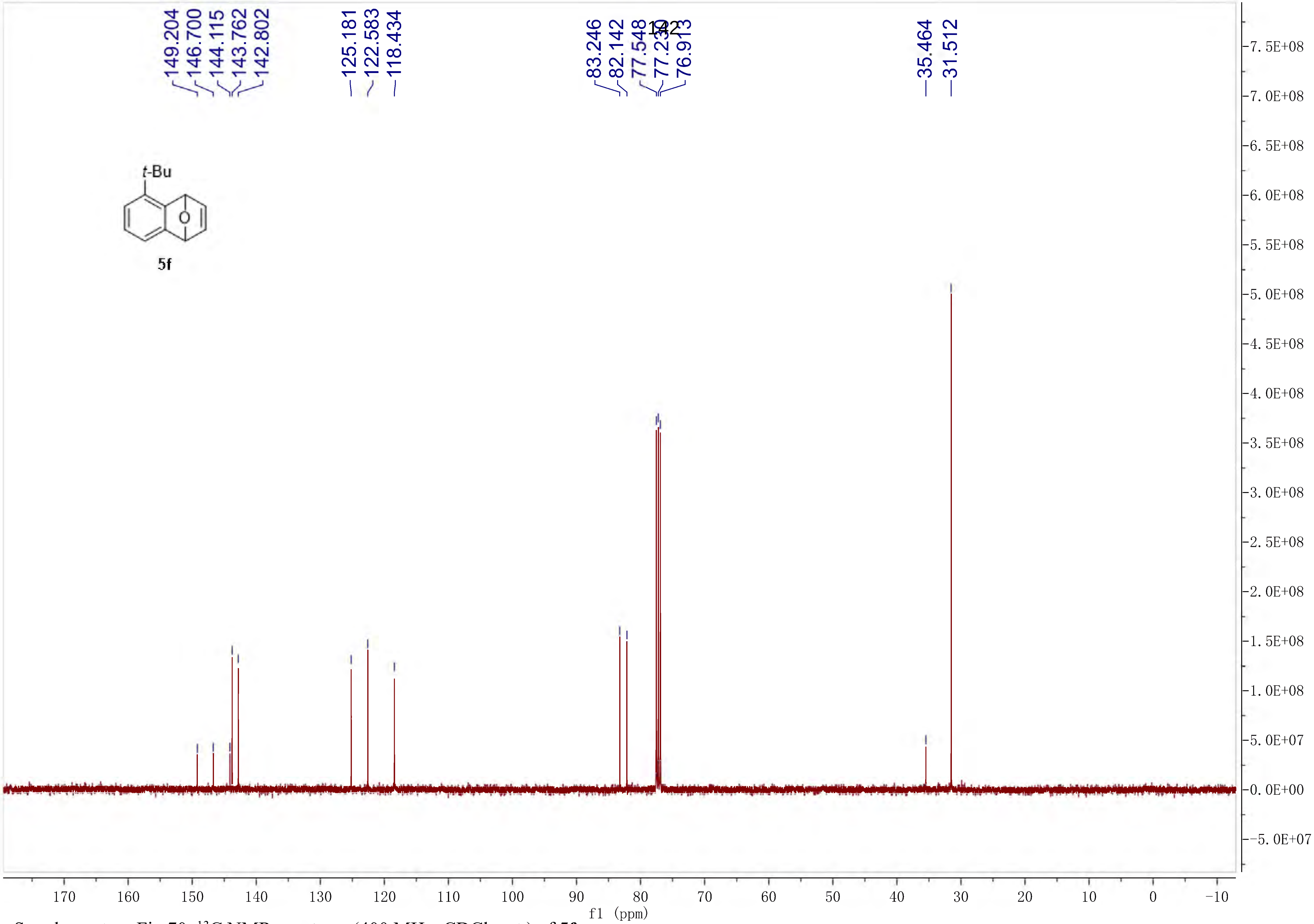

Supplementary Fig 70. ^{13}C NMR spectrum (400 MHz, $CDCl_3$, r.t.) of **5f**.

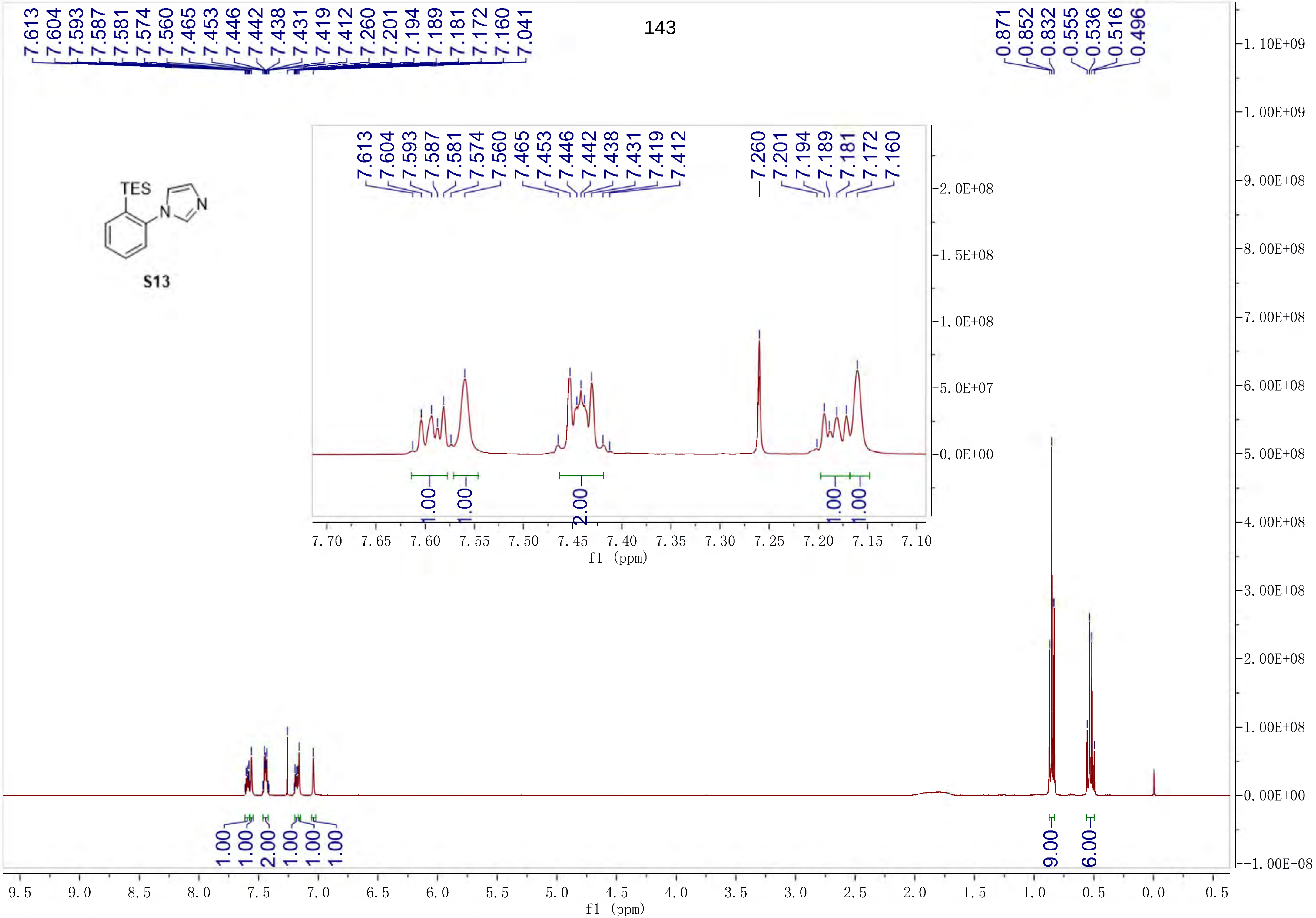

Supplementary Fig 71. ^{1}H NMR spectrum (400 MHz, $CDCl_3$, r.t.) of **S13**.

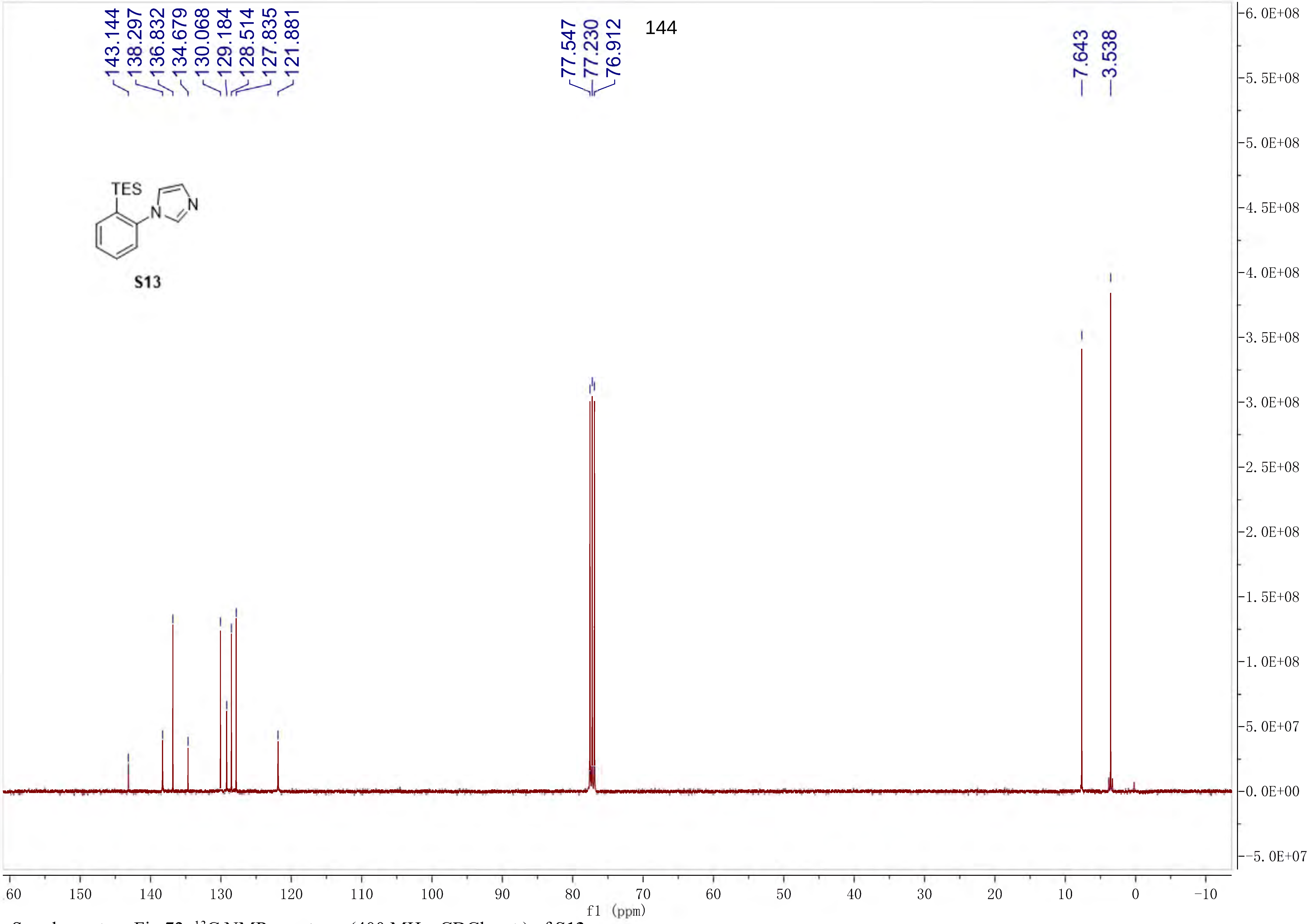

Supplementary Fig 72. ^{13}C NMR spectrum (400 MHz, $CDCl_3$, r.t.) of **S13**.

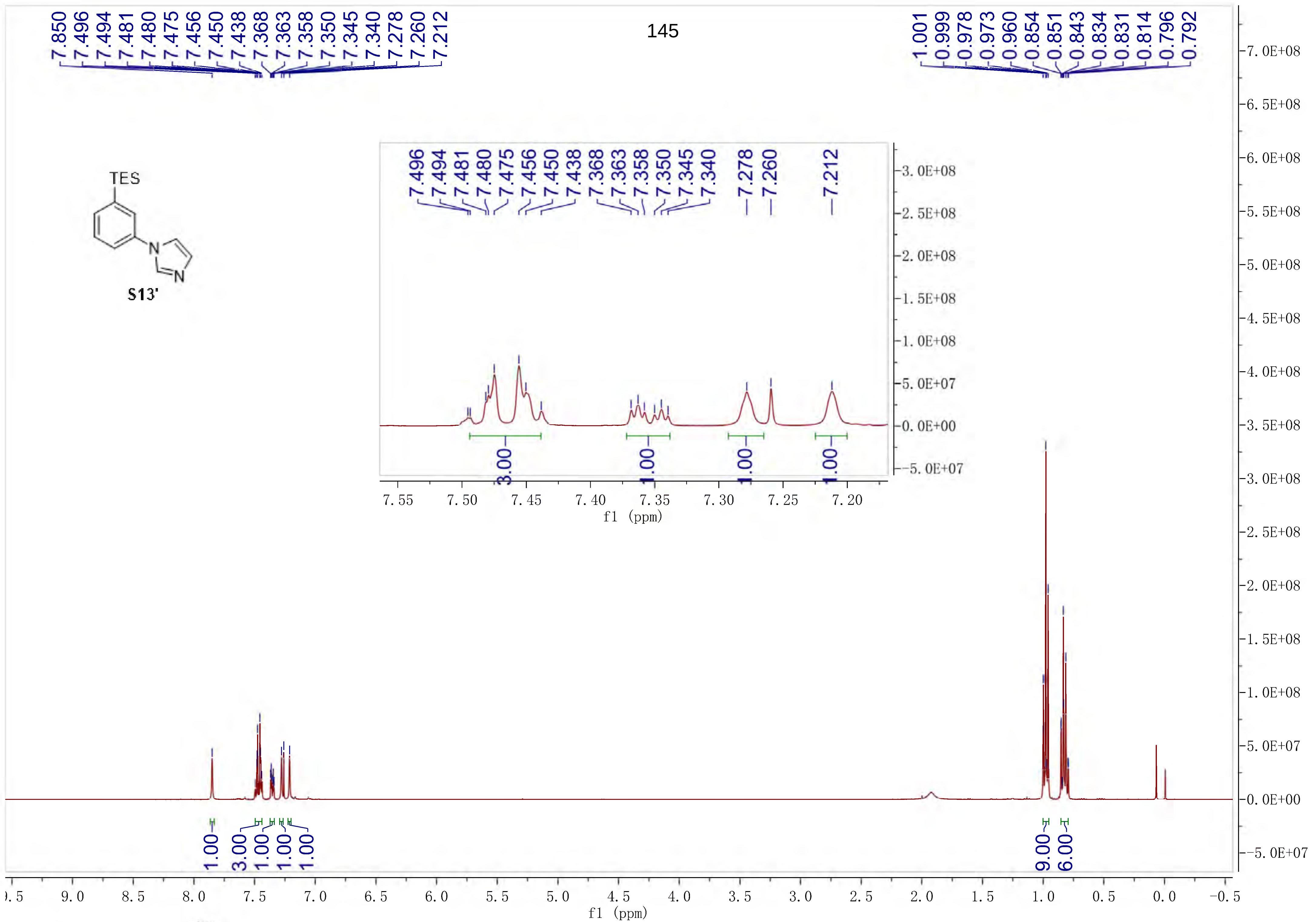

Supplementary Fig 73. ^{1}H NMR spectrum (400 MHz, $CDCl_3$, r.t.) of **S13’**.

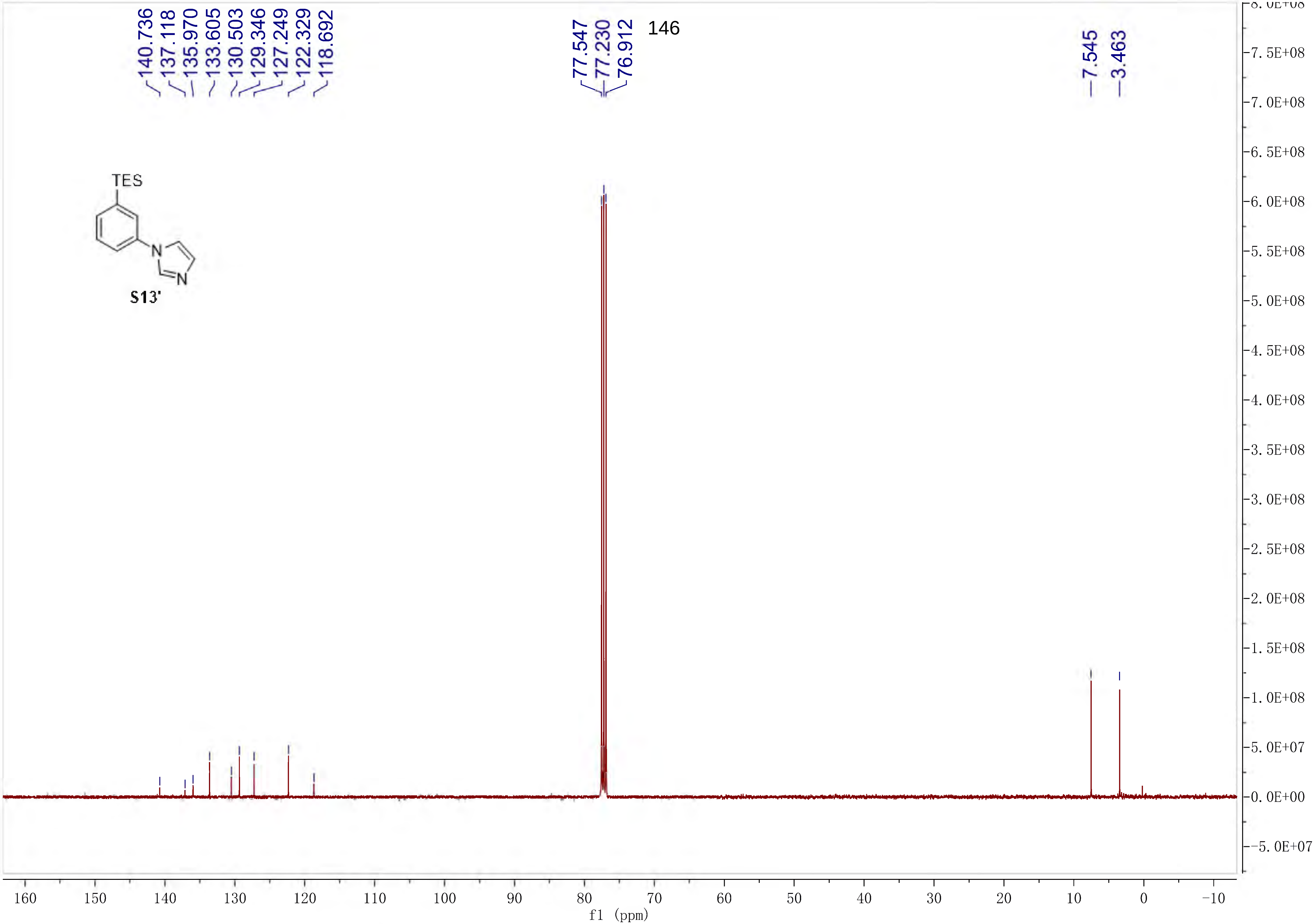

Supplementary Fig 74. ^{13}C NMR spectrum (400 MHz, $CDCl_3$, r.t.) of **S13’**.

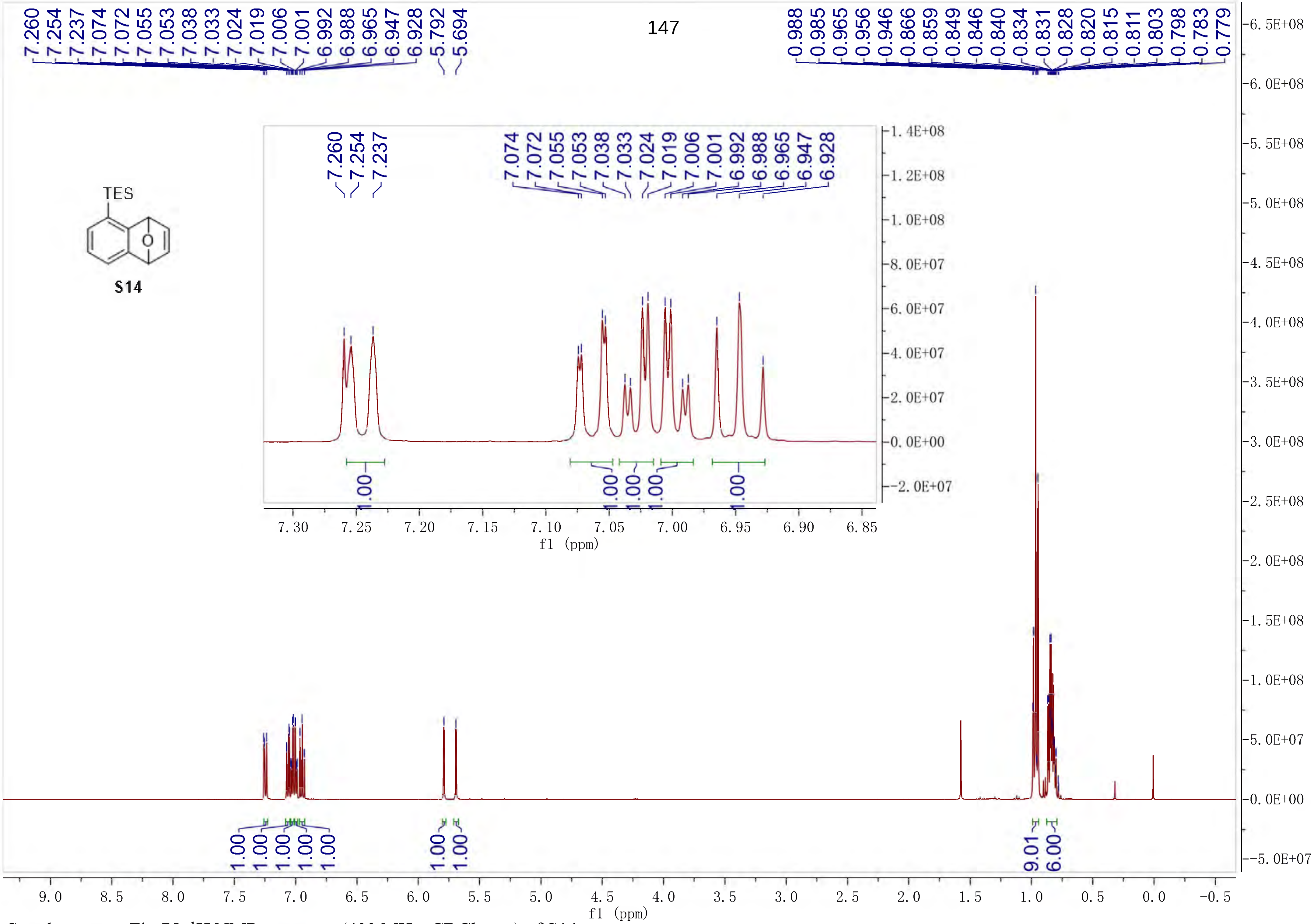

Supplementary Fig 75. ^{1}H NMR spectrum (400 MHz, $CDCl_3$, r.t.) of **S14**.

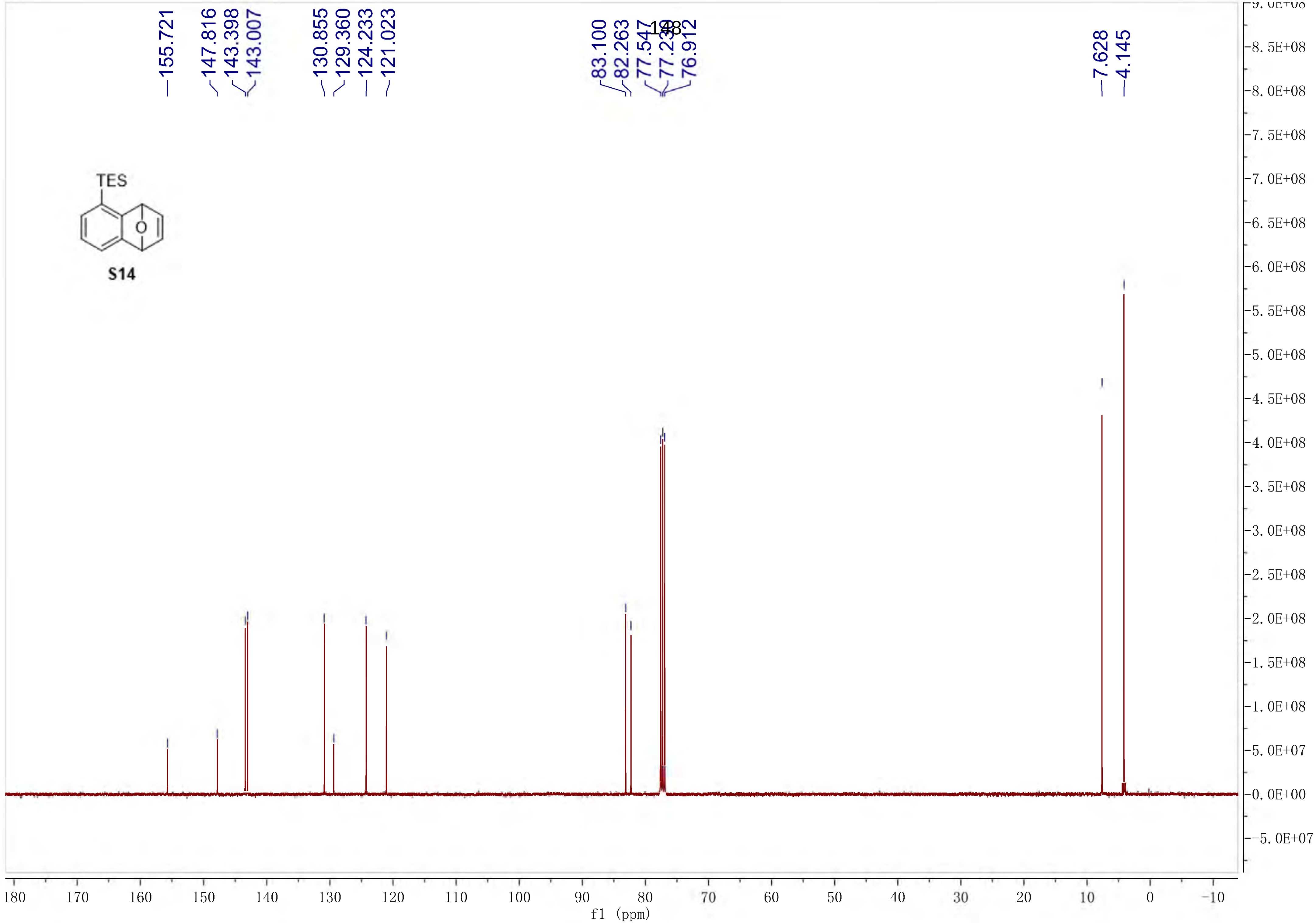

Supplementary Fig 76. ^{13}C NMR spectrum (400 MHz, $CDCl_3$, r.t.) of **S14**.

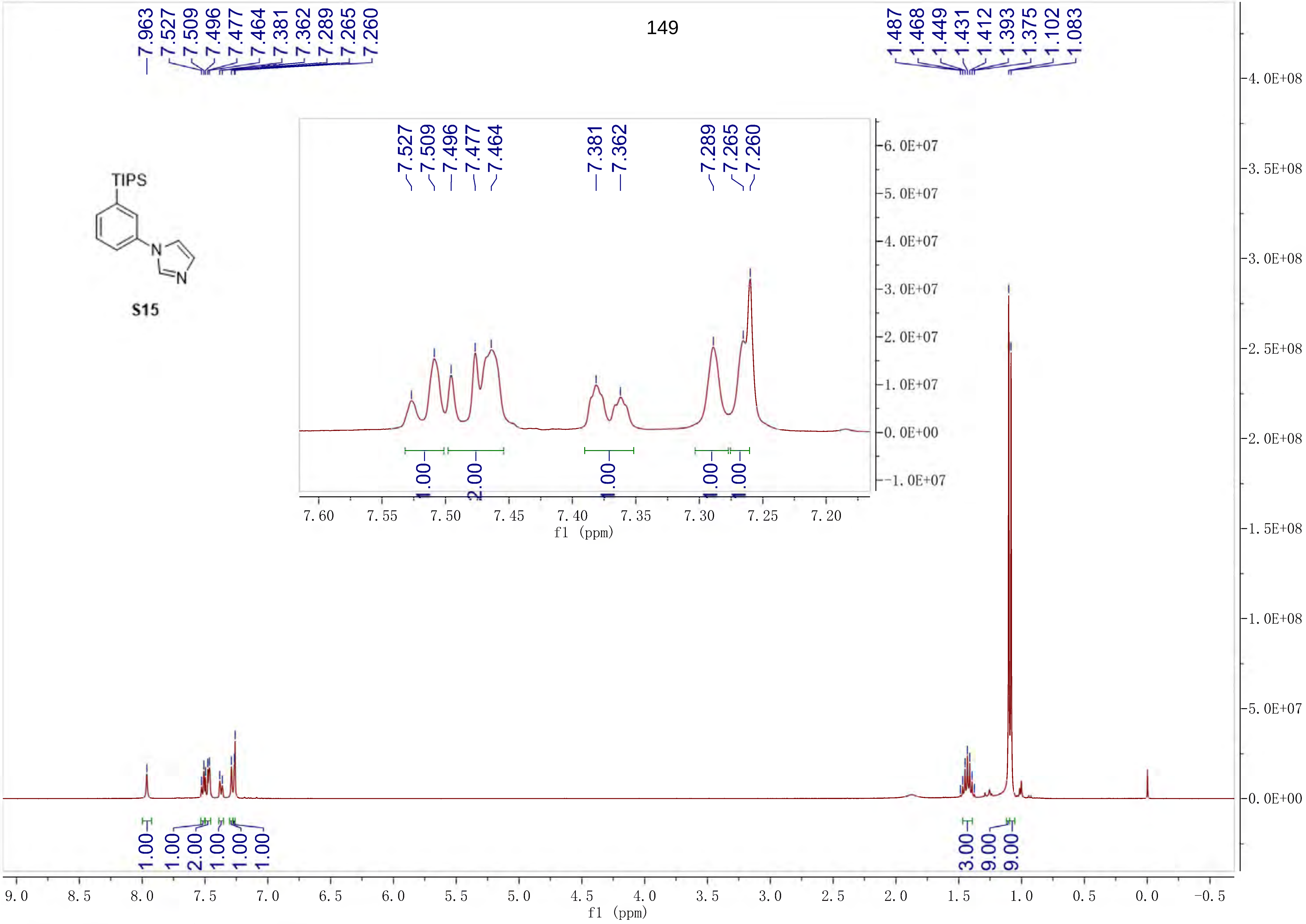

Supplementary Fig 77. ^{1}H NMR spectrum (400 MHz, $CDCl_3$, r.t.) of **S15**.

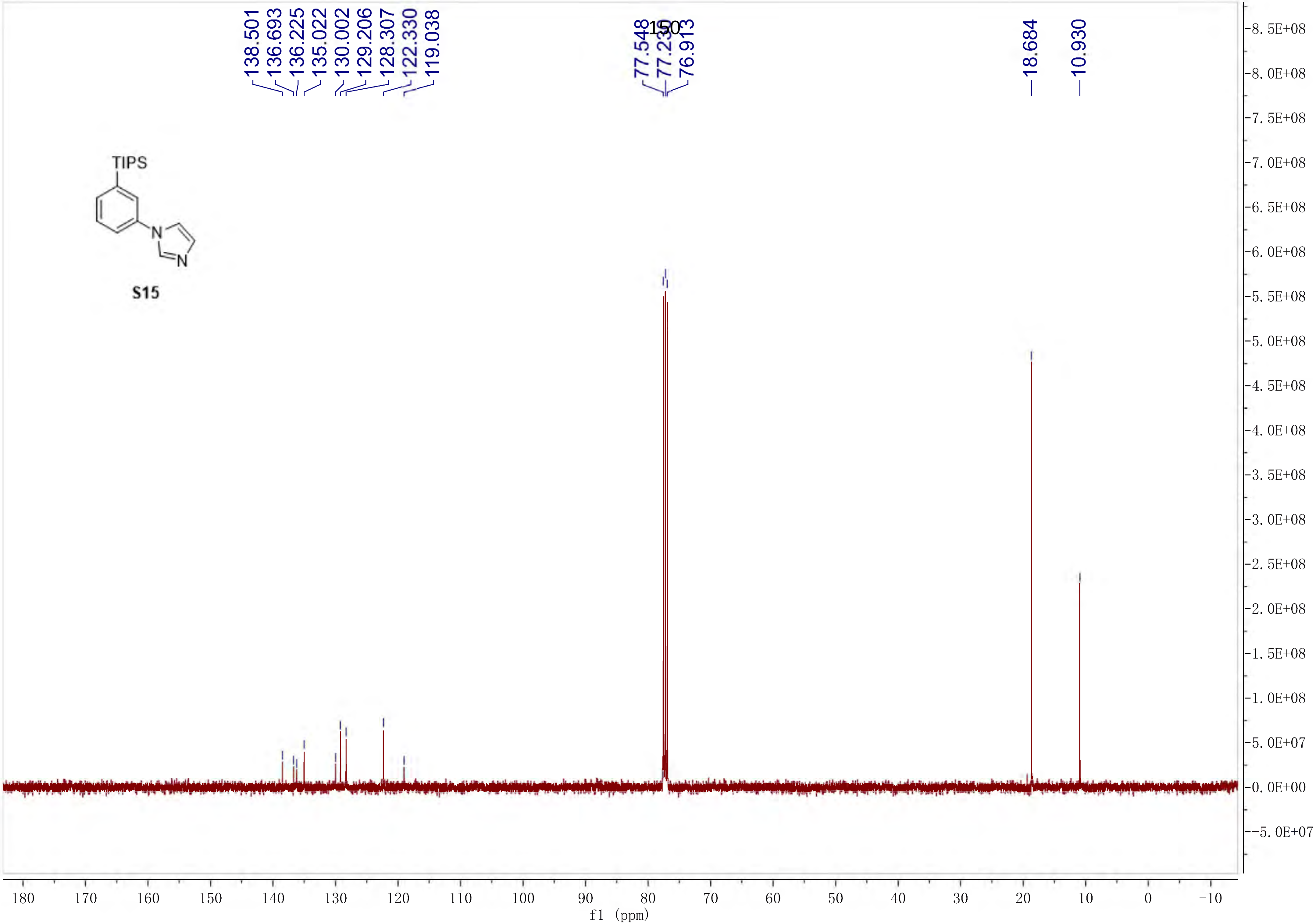

Supplementary Fig 78. ^{13}C NMR spectrum (400 MHz, $CDCl_3$, r.t.) of **S15**.

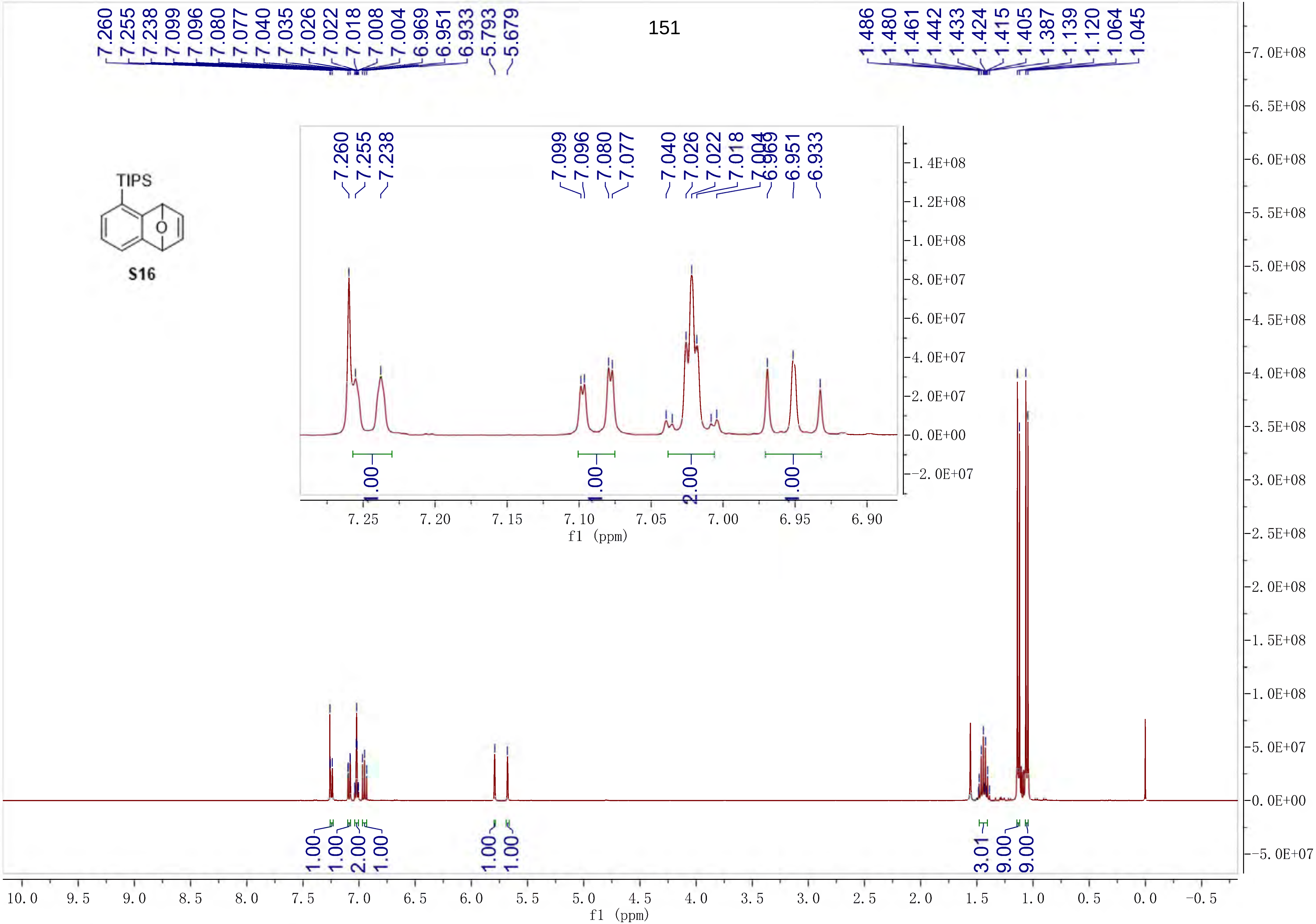

Supplementary Fig 79. ^{1}H NMR spectrum (400 MHz, $CDCl_3$, r.t.) of **S16**.

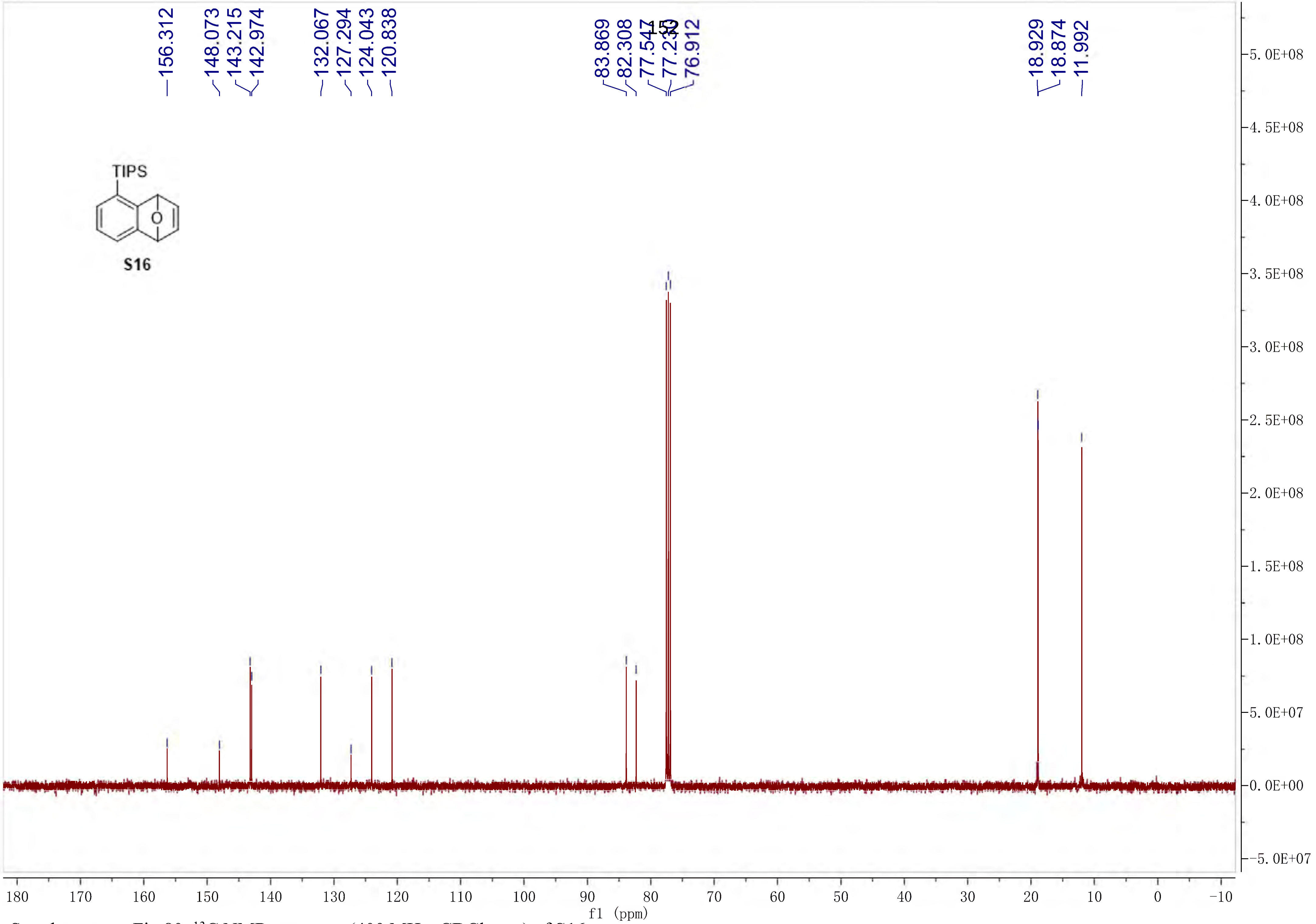

Supplementary Fig 80. ^{13}C NMR spectrum (400 MHz, $CDCl_3$, r.t.) of **S16**.

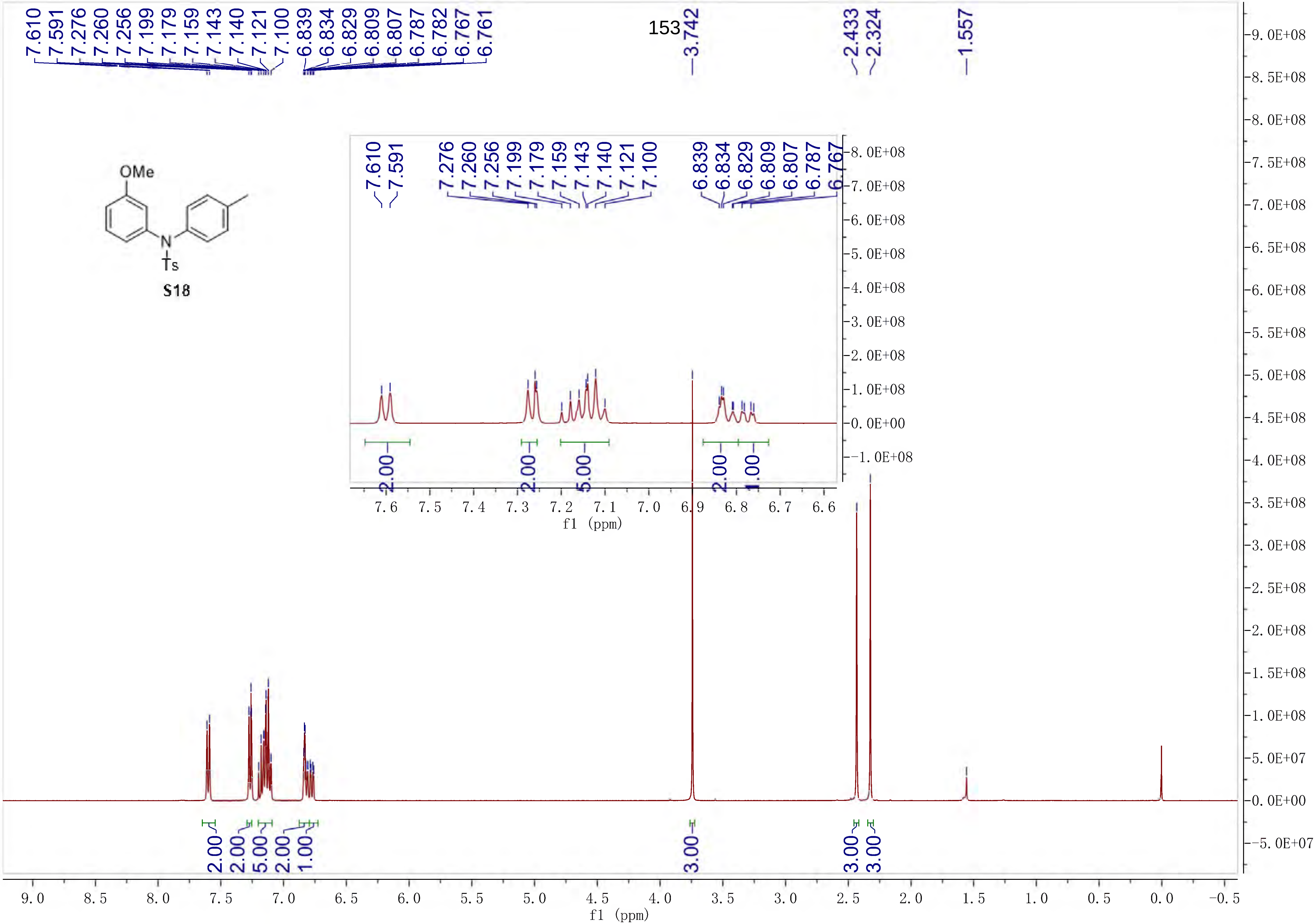

Supplementary Fig 81. ^{1}H NMR spectrum (400 MHz, $CDCl_3$, r.t.) of **S18**.

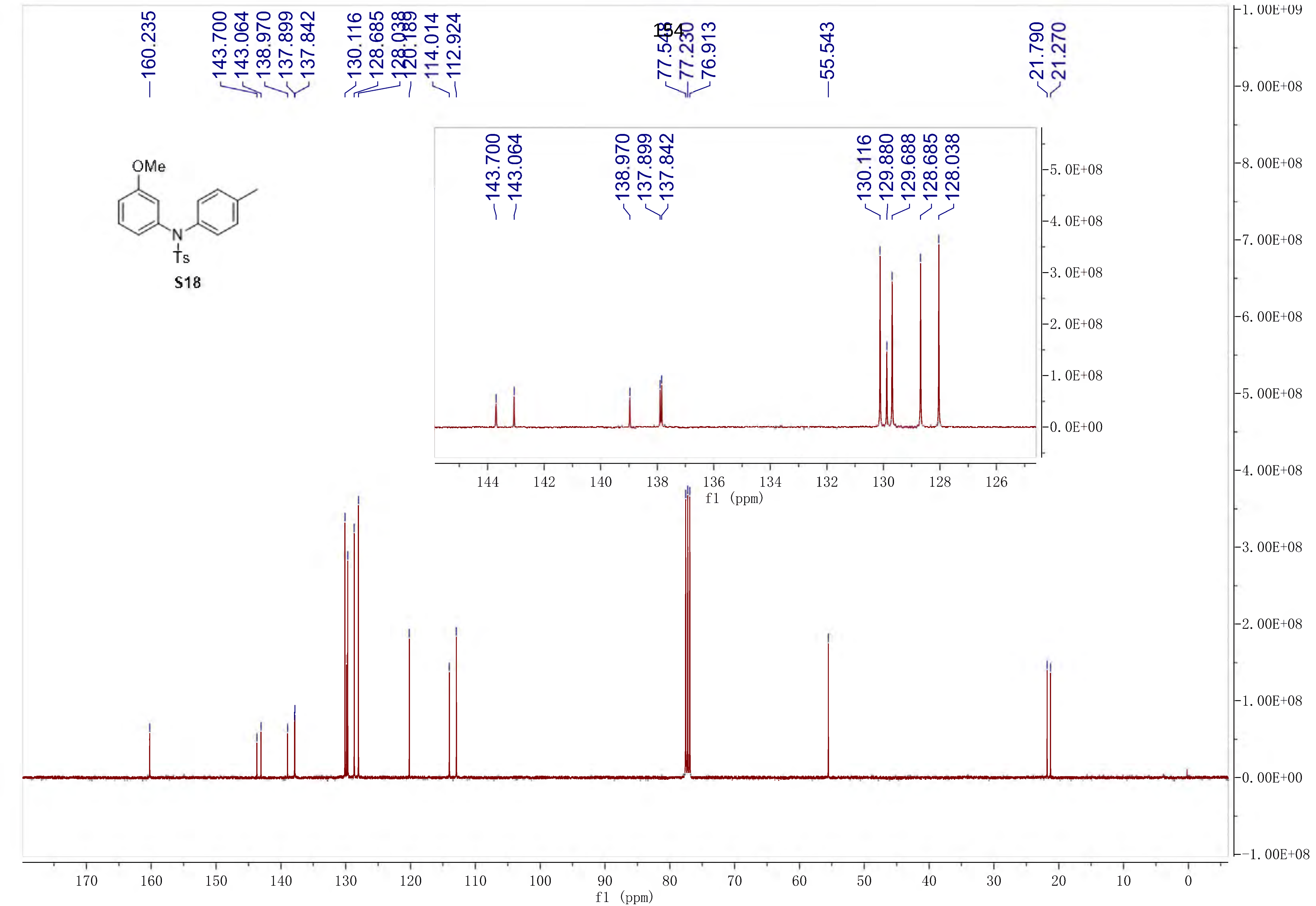

Supplementary Fig 82. ^{13}C NMR spectrum (400 MHz, $CDCl_3$, r.t.) of **S18**.

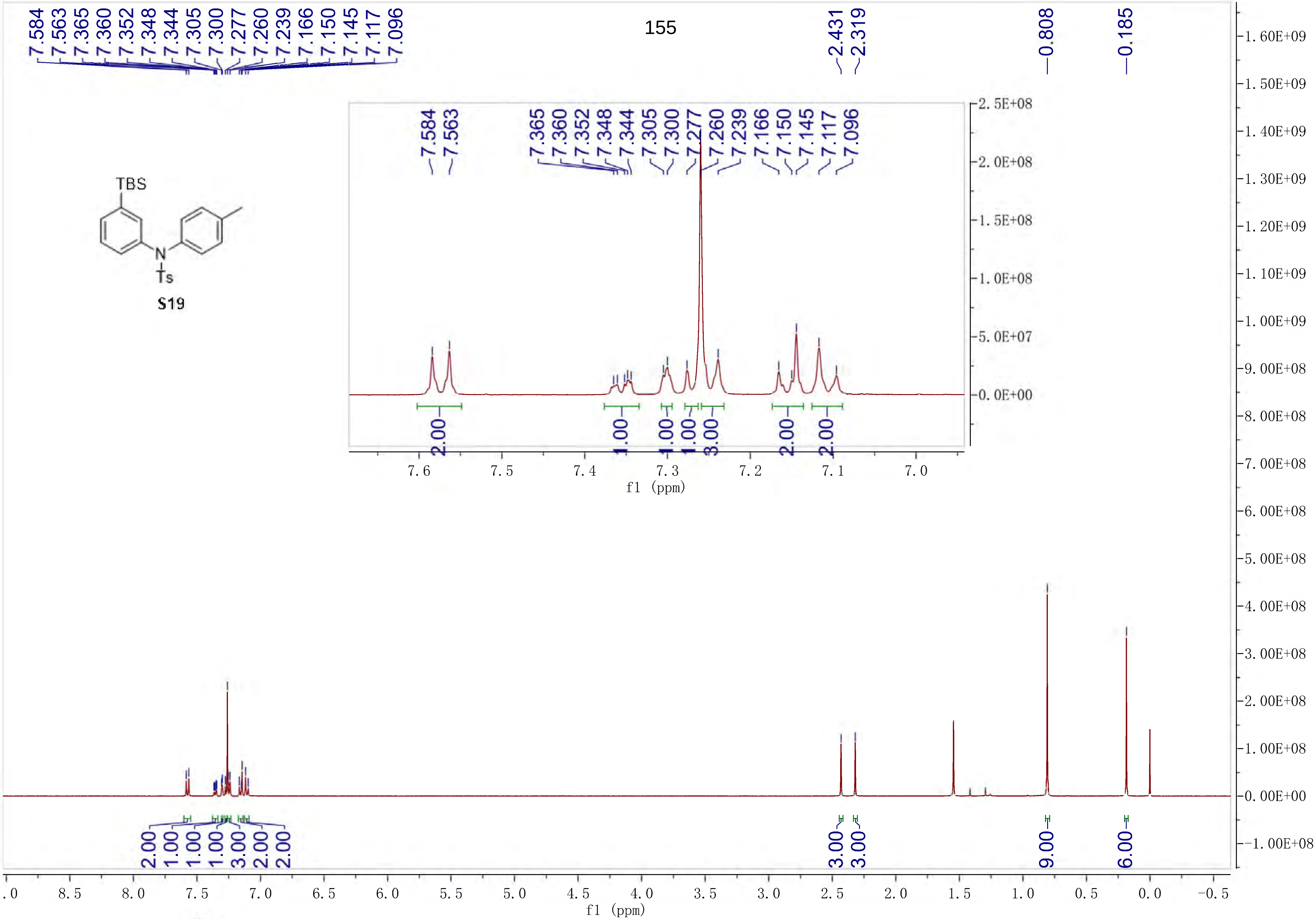

Supplementary Fig 83. ^{1}H NMR spectrum (400 MHz, $CDCl_3$, r.t.) of **S19**.

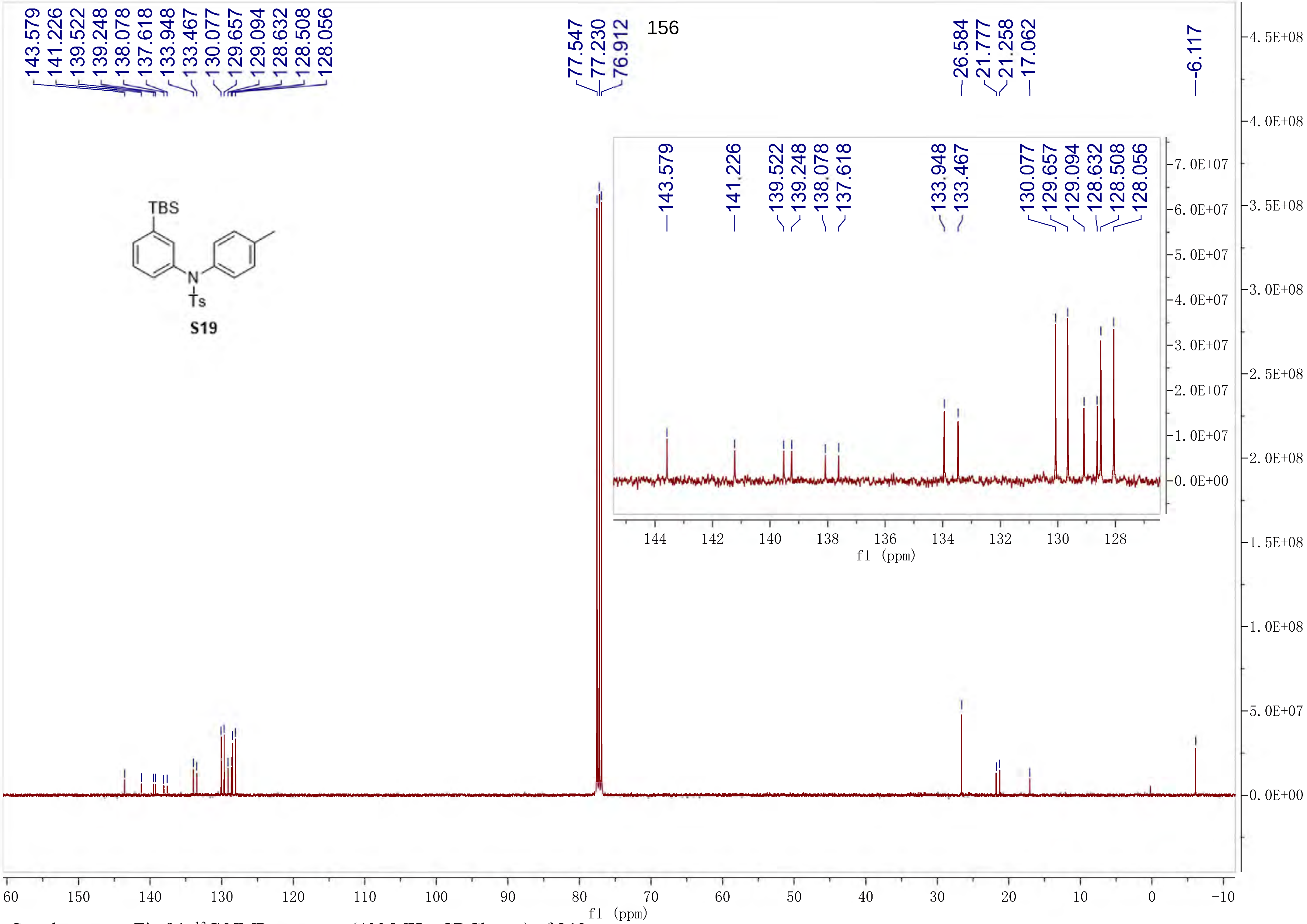

Supplementary Fig 84. ^{13}C NMR spectrum (400 MHz, $CDCl_3$, r.t.) of **S19**.

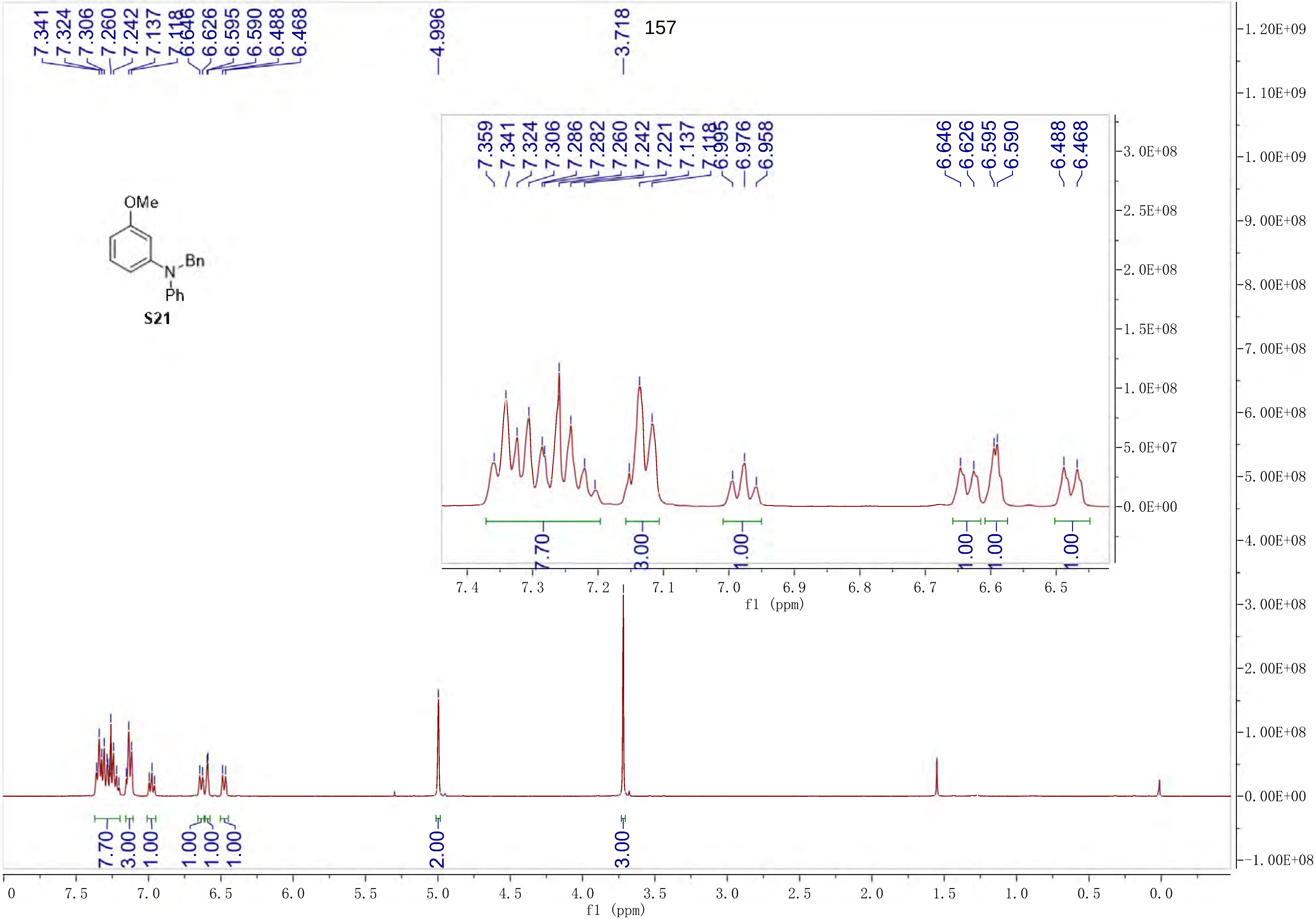

Supplementary Fig 85. ^{1}H NMR spectrum (400 MHz, $CDCl_3$, r.t.) of **S21**.

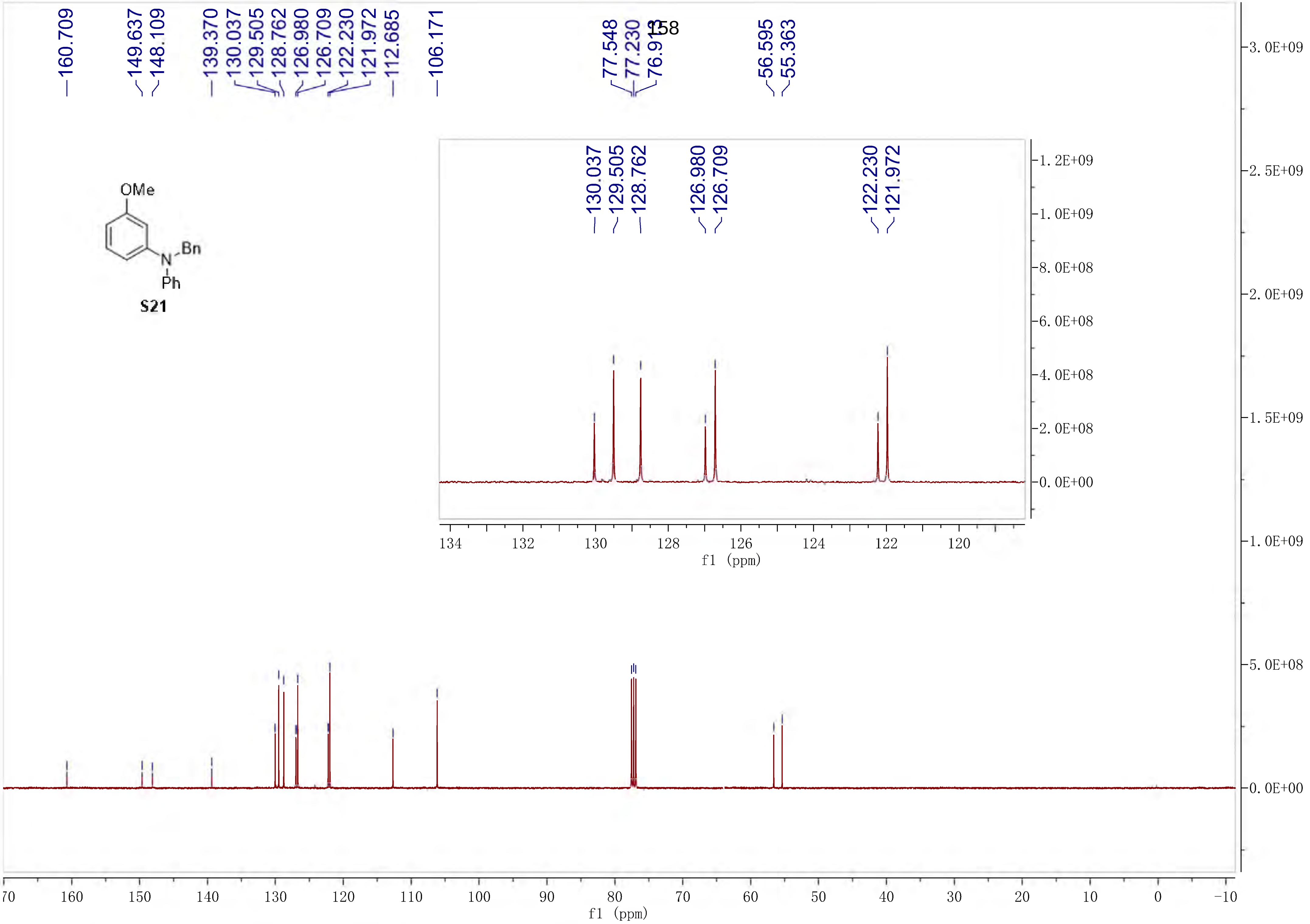

Supplementary Fig 86. ^{13}C NMR spectrum (400 MHz, $CDCl_3$, r.t.) of **S21**.

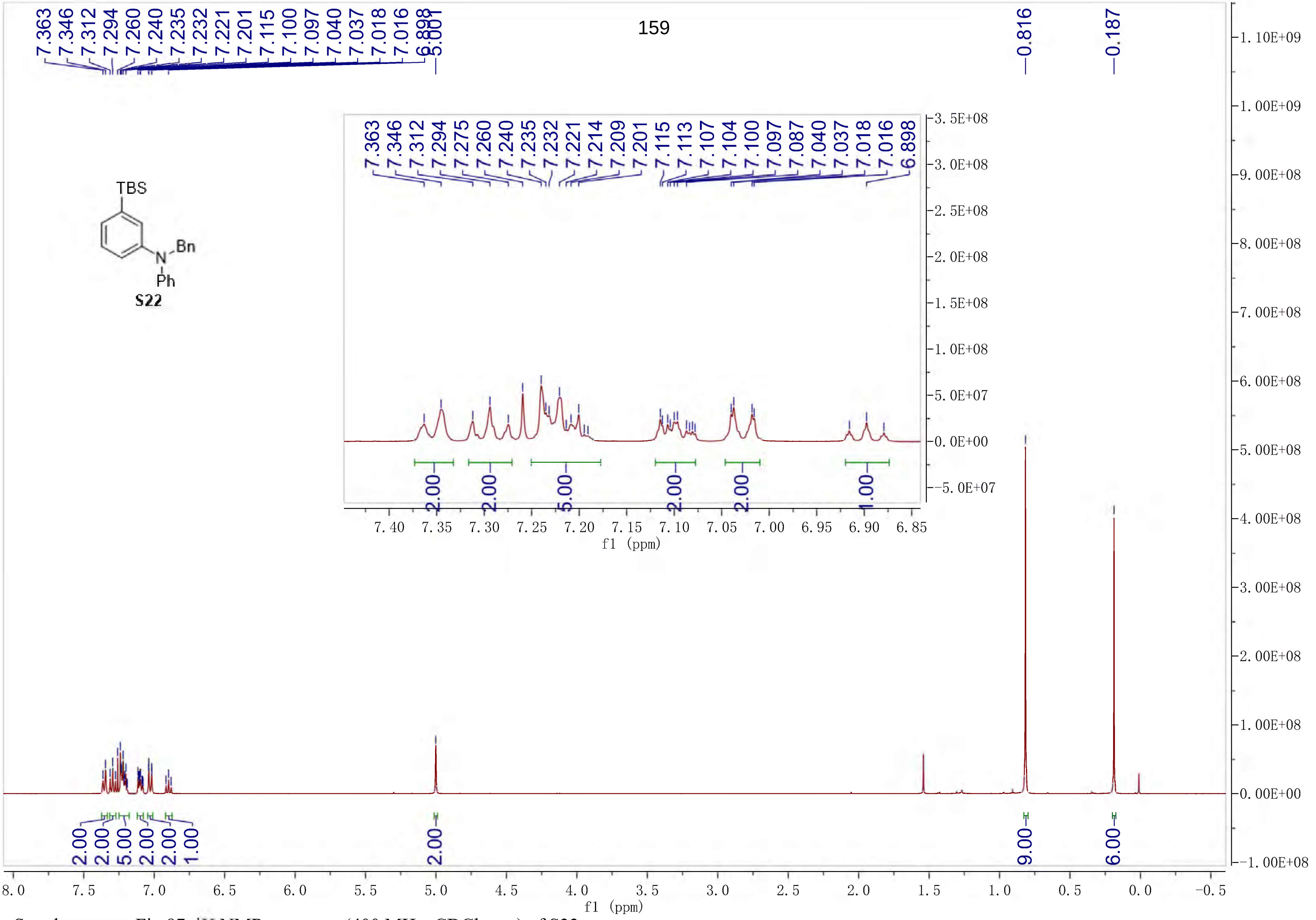

Supplementary Fig 87. ^{1}H NMR spectrum (400 MHz, $CDCl_3$, r.t.) of **S22**.

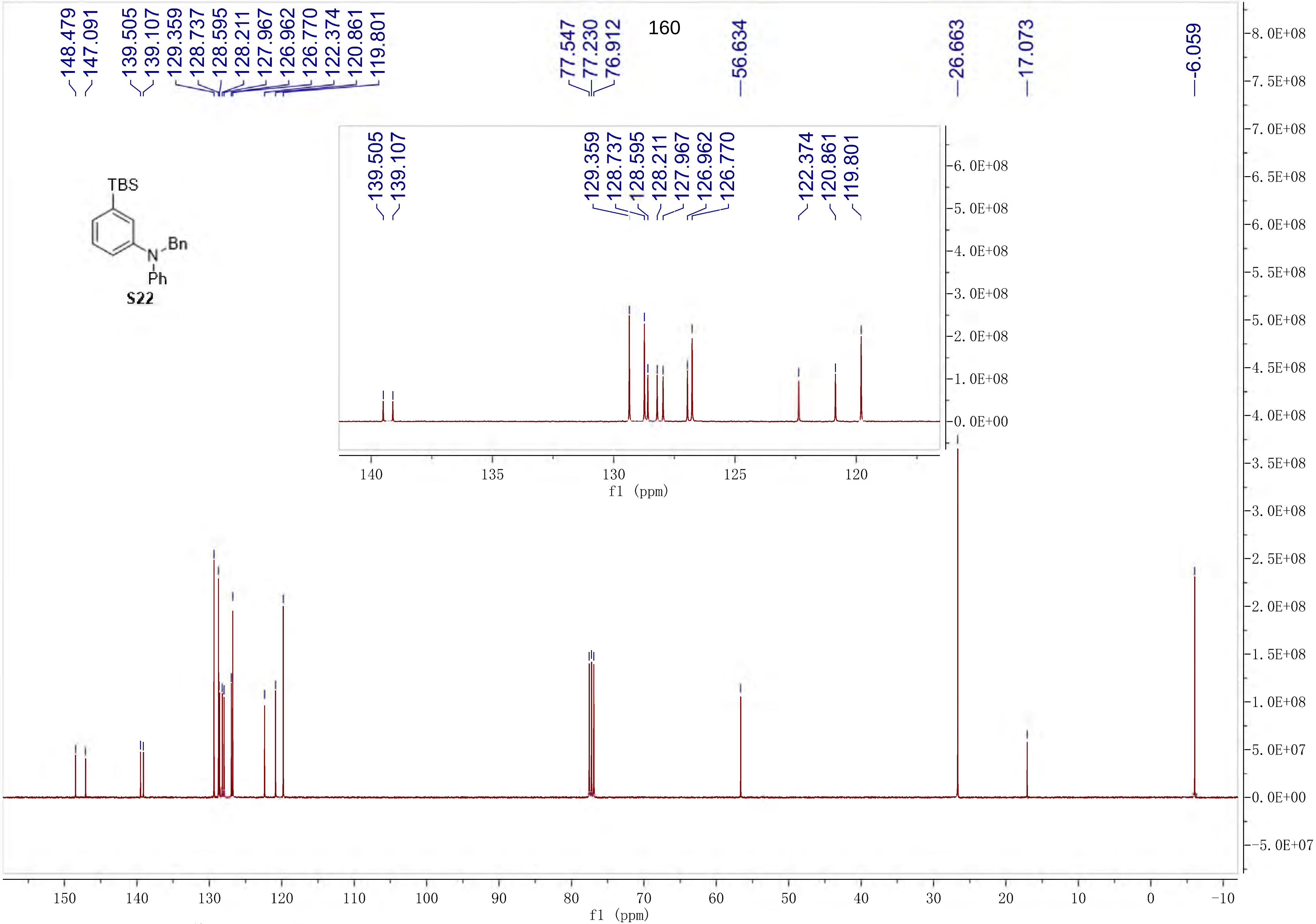

Supplementary Fig 88. ^{13}C NMR spectrum (400 MHz, $CDCl_3$, r.t.) of **S22**.

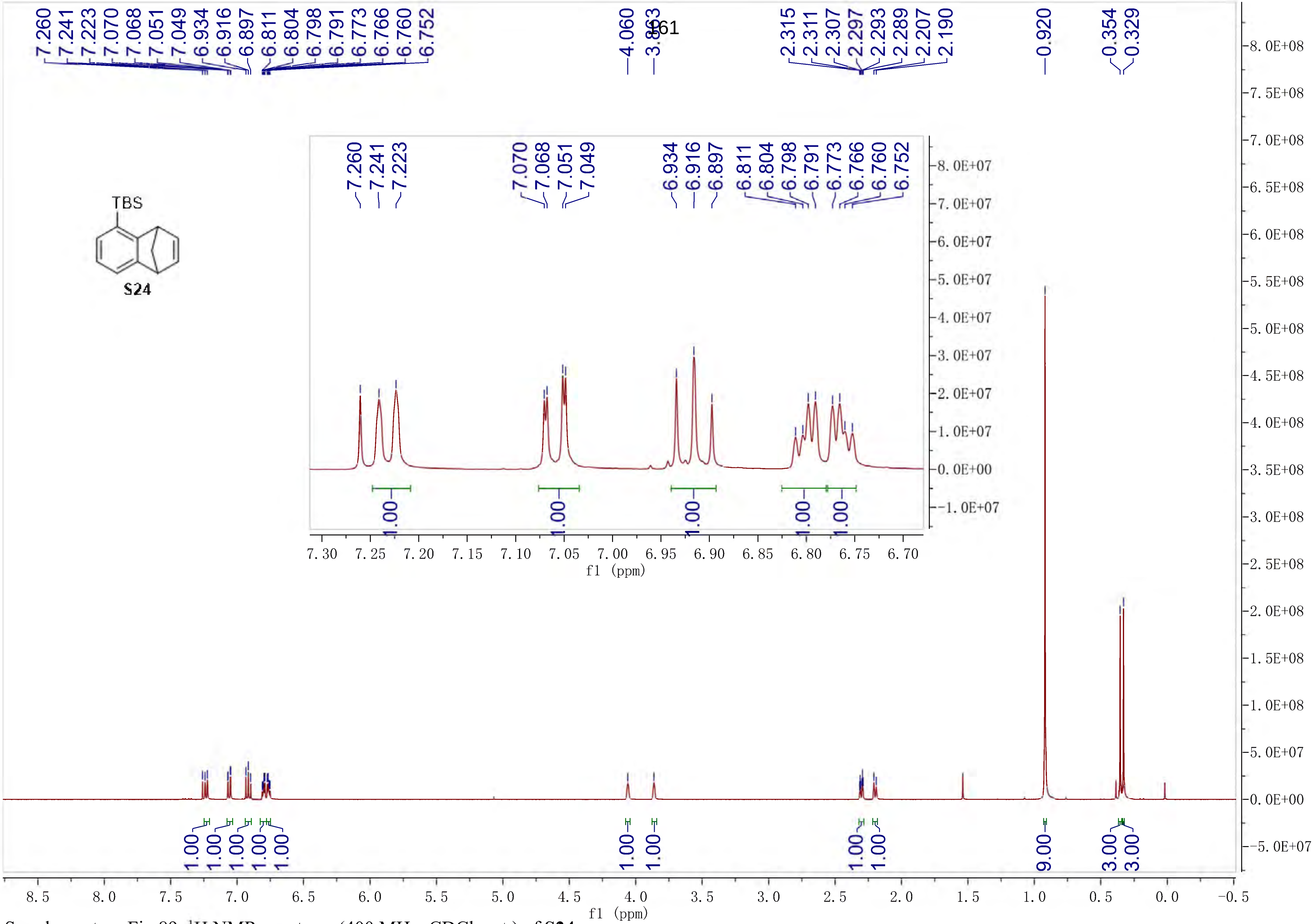

Supplementary Fig 89. ^{1}H NMR spectrum (400 MHz, $CDCl_3$, r.t.) of **S24**.

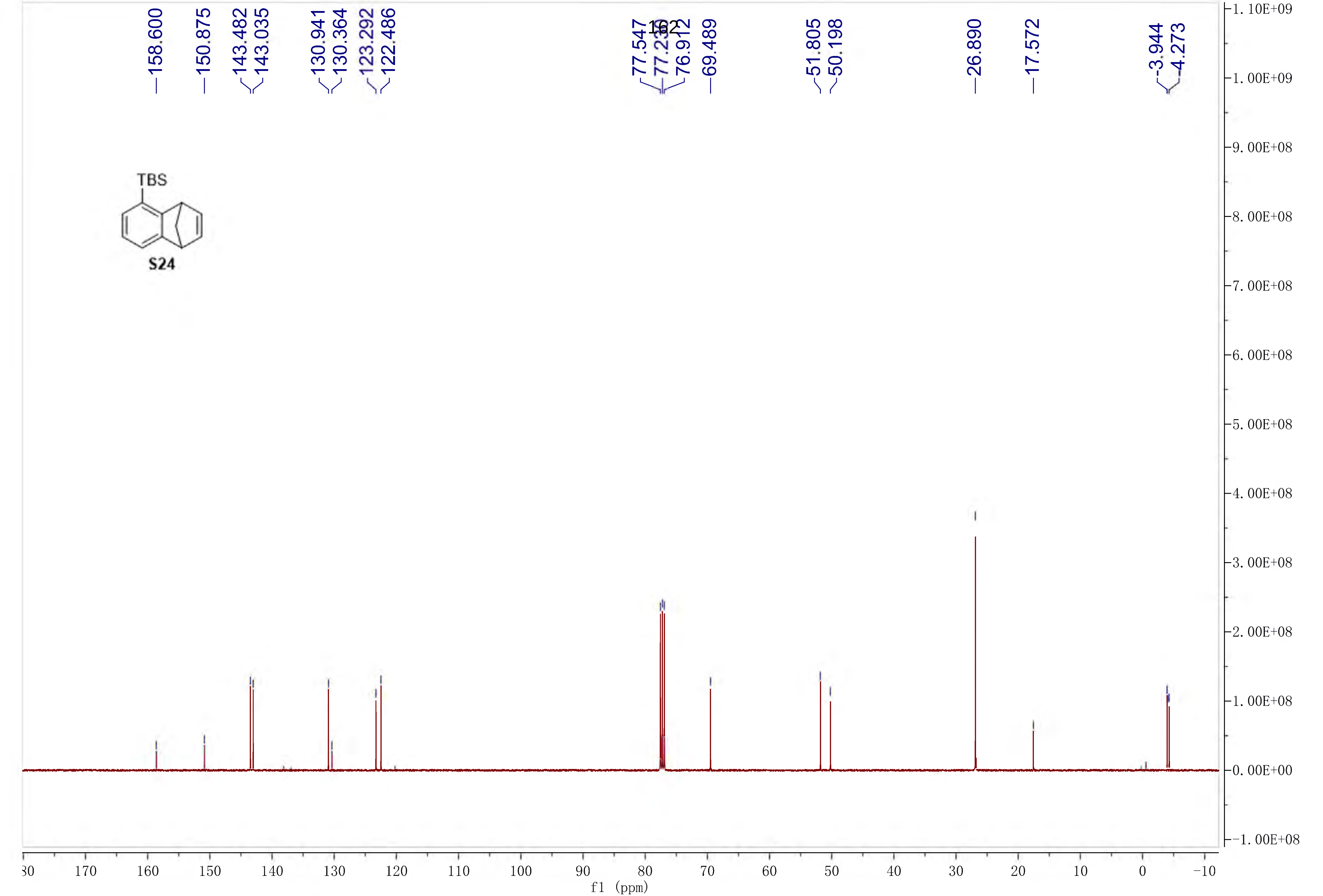

Supplementary Fig 90. ^{13}C NMR spectrum (400 MHz, $CDCl_3$, r.t.) of **S24**.

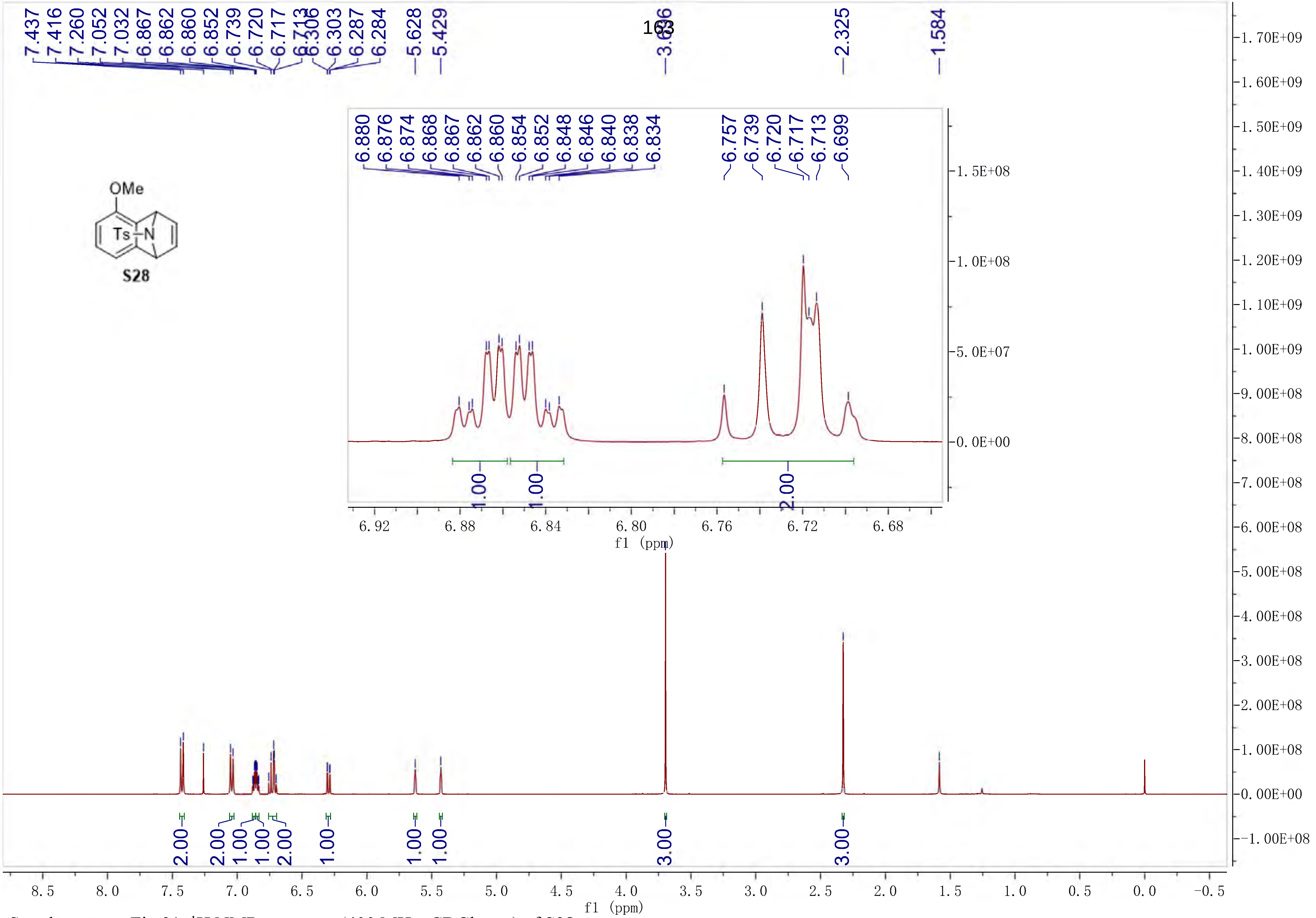

Supplementary Fig 91. ^{1}H NMR spectrum (400 MHz, $CDCl_3$, r.t.) of **S28**.

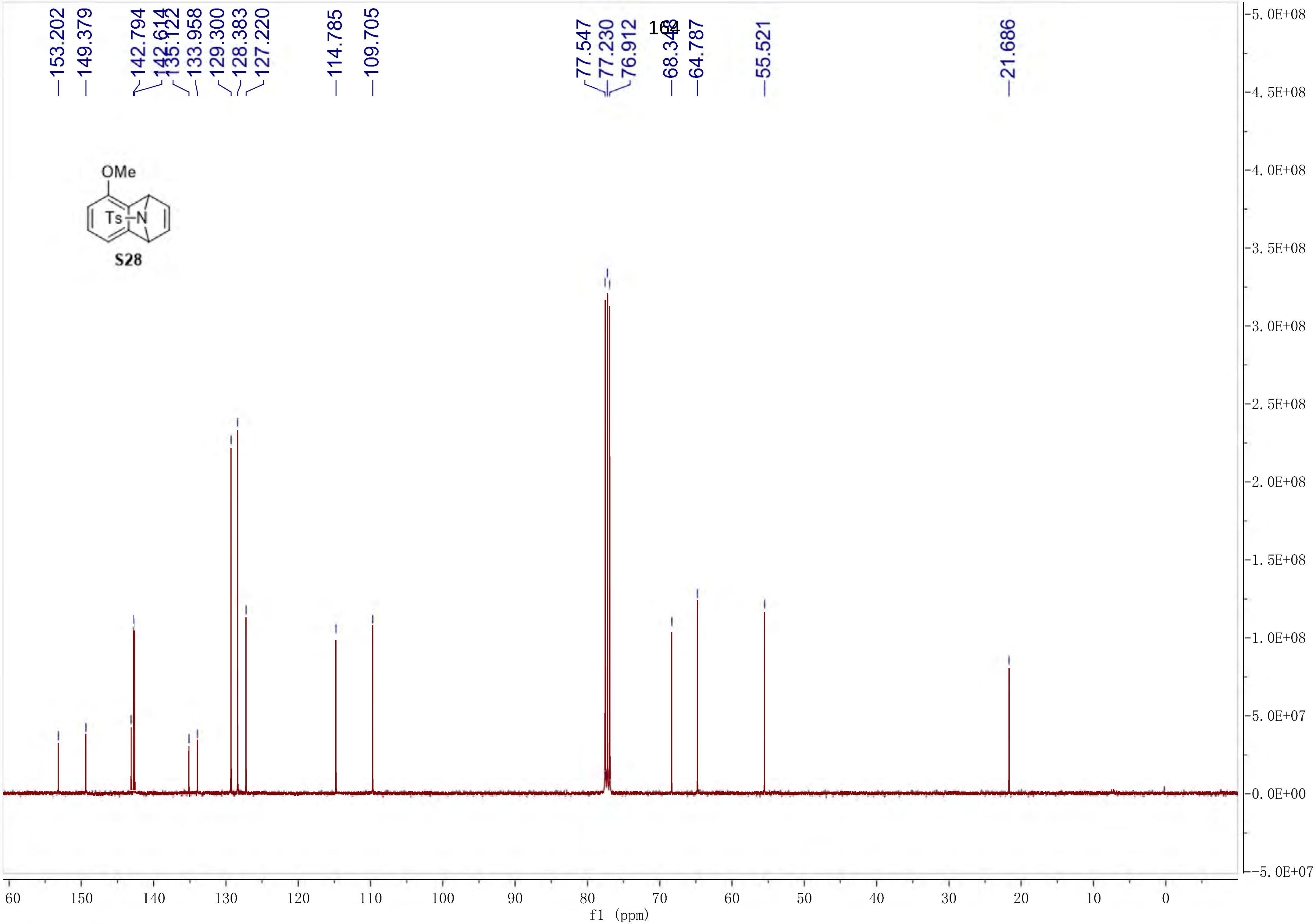

Supplementary Fig 92. ^{13}C NMR spectrum (400 MHz, $CDCl_3$, r.t.) of **S28**.

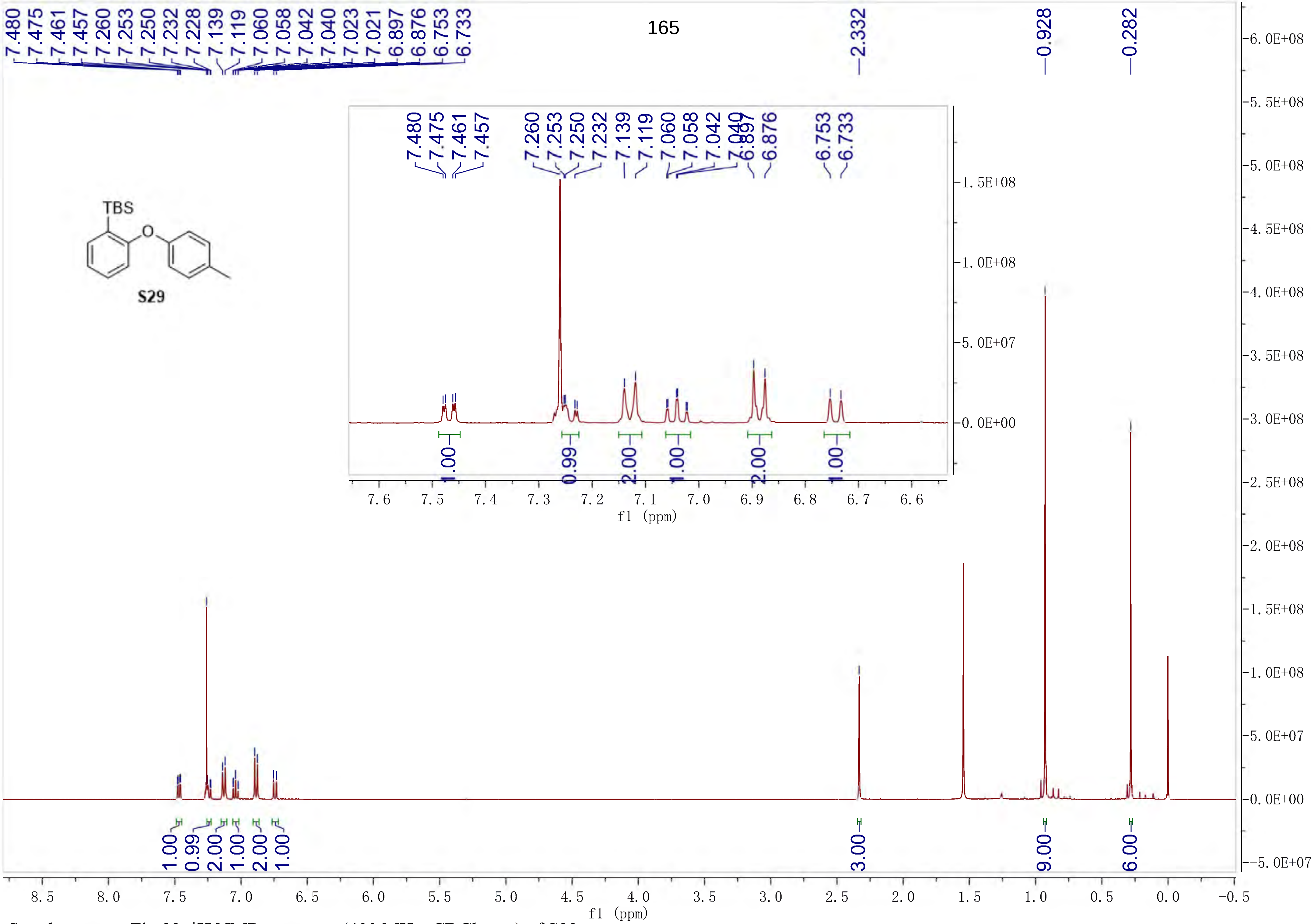

Supplementary Fig 93. ^{1}H NMR spectrum (400 MHz, $CDCl_3$, r.t.) of **S29**.

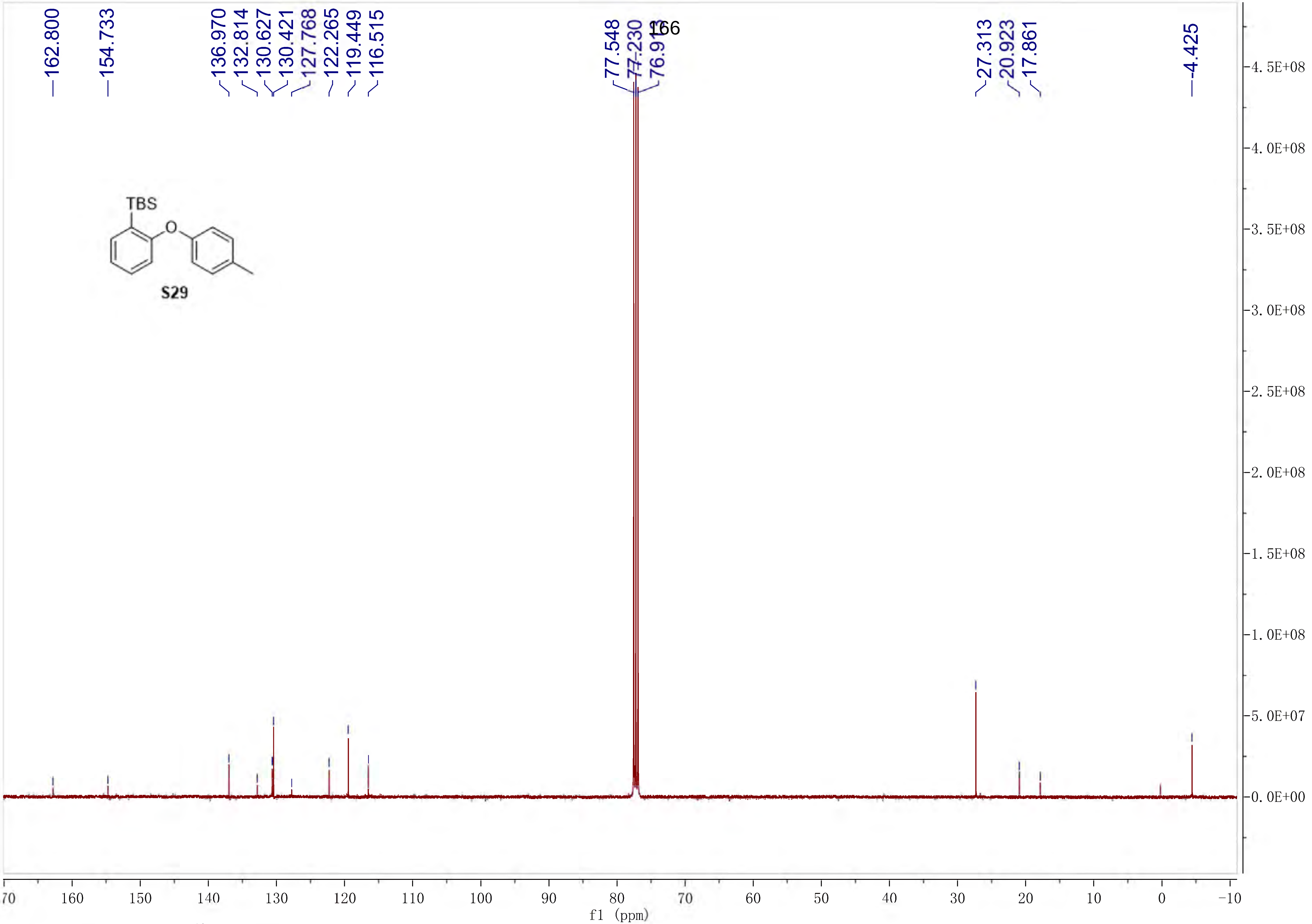

Supplementary Fig 94. ^{13}C NMR spectrum (400 MHz, $CDCl_3$, r.t.) of **S29**.

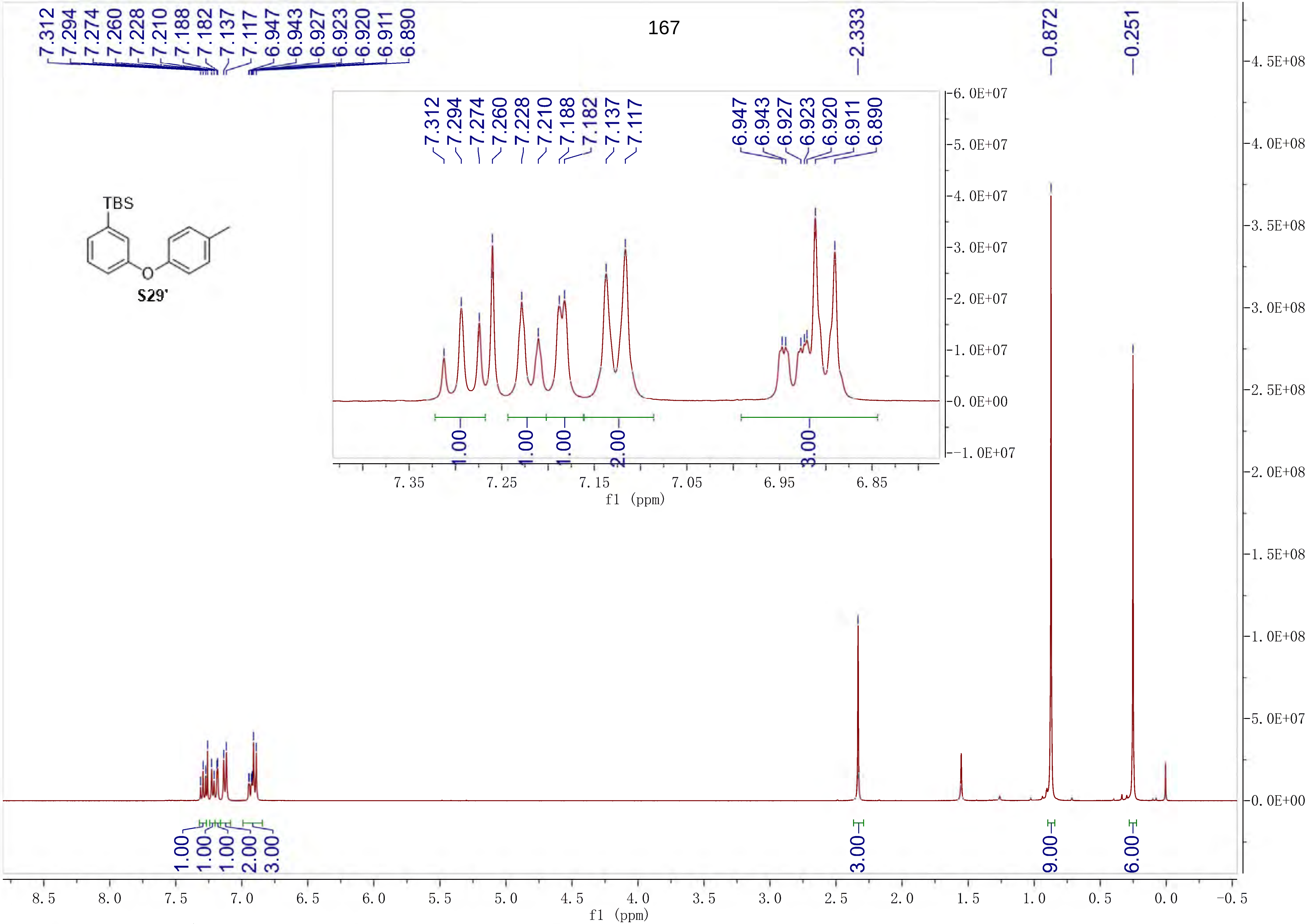

Supplementary Fig 95. ^{1}H NMR spectrum (400 MHz, $CDCl_3$, r.t.) of **S29'**.

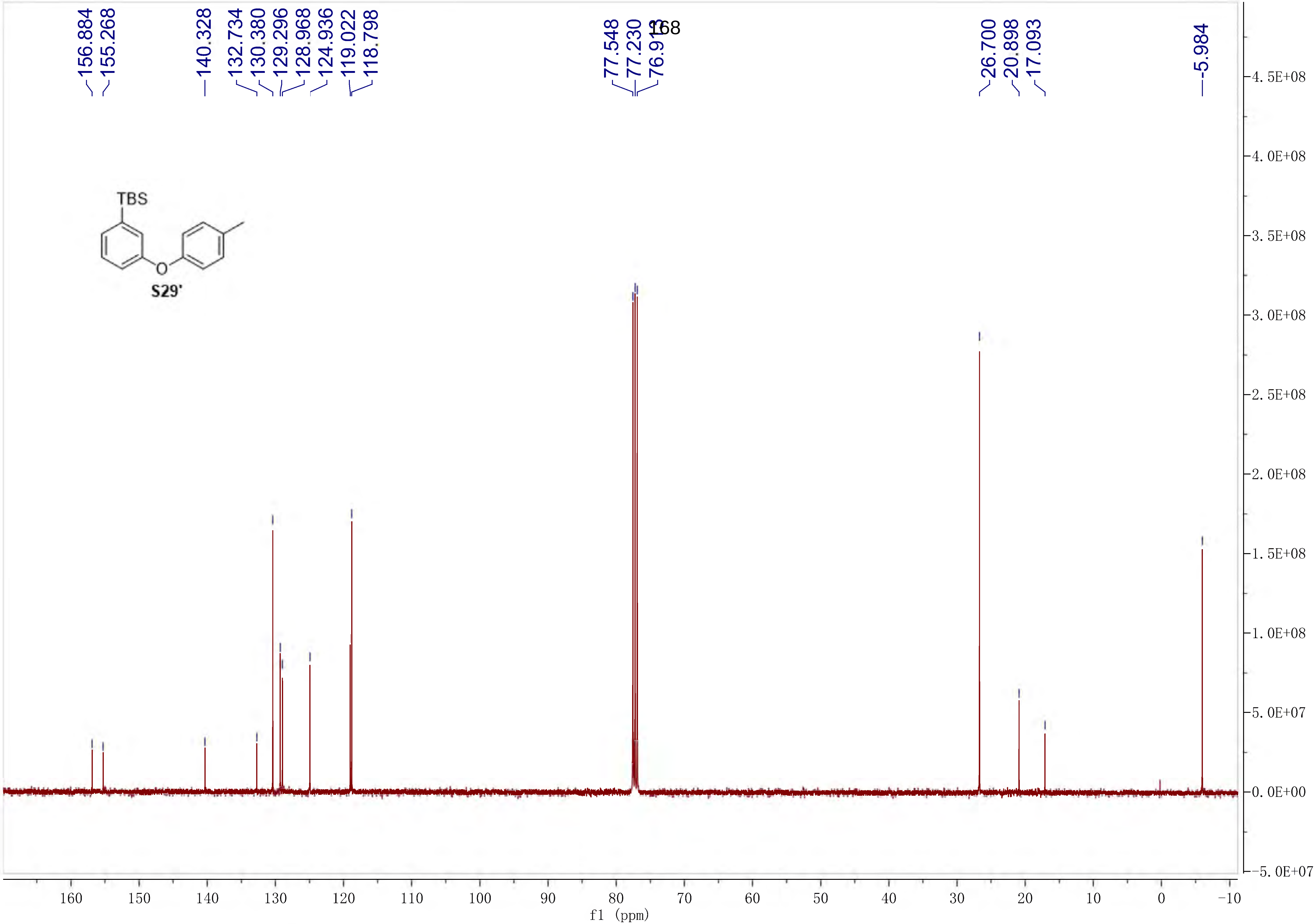

Supplementary Fig 96. ^{13}C NMR spectrum (400 MHz, $CDCl_3$, r.t.) of **S29'**.

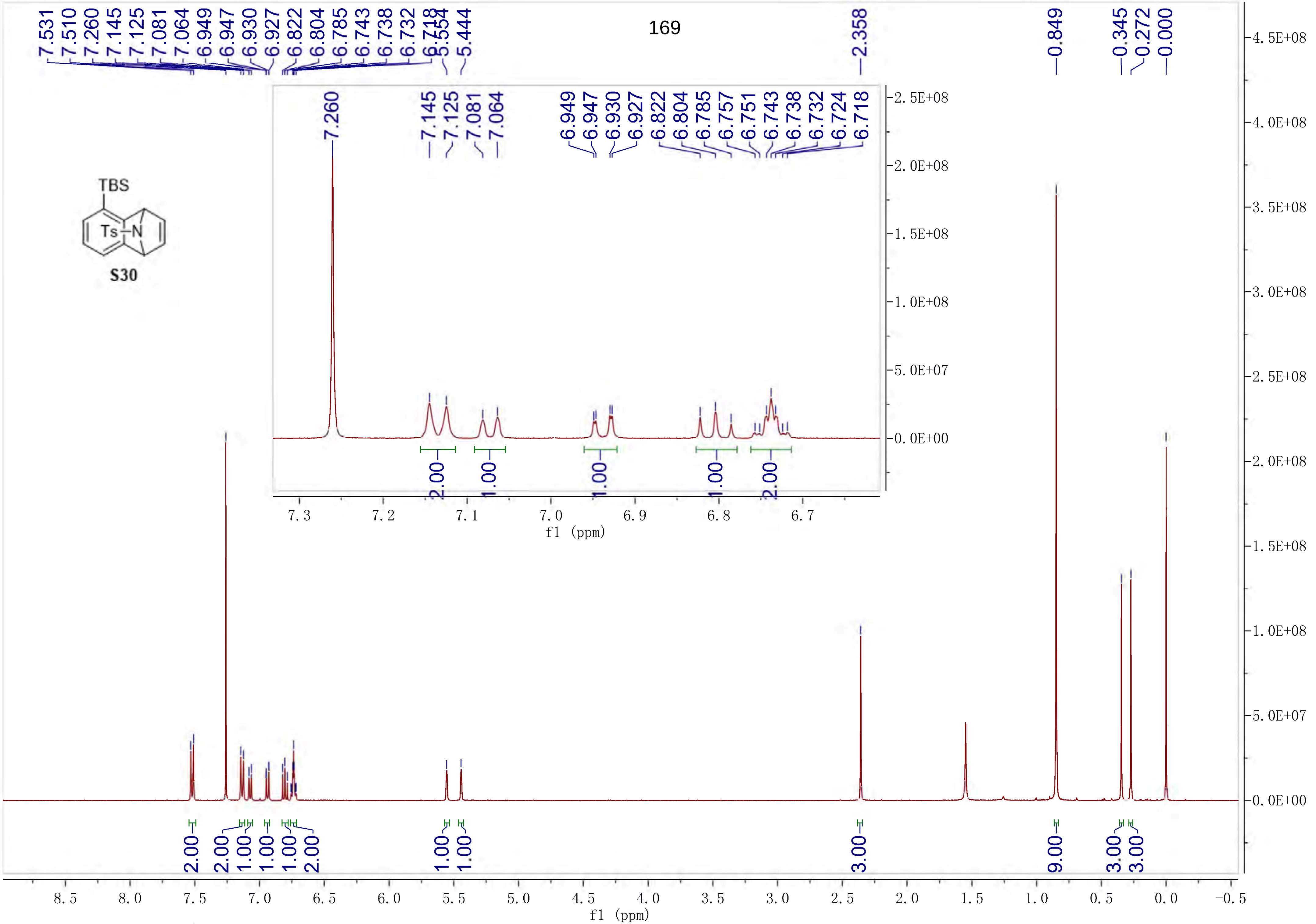

Supplementary Fig 97. ^{1}H NMR spectrum (400 MHz, $CDCl_3$, r.t.) of **S30**.

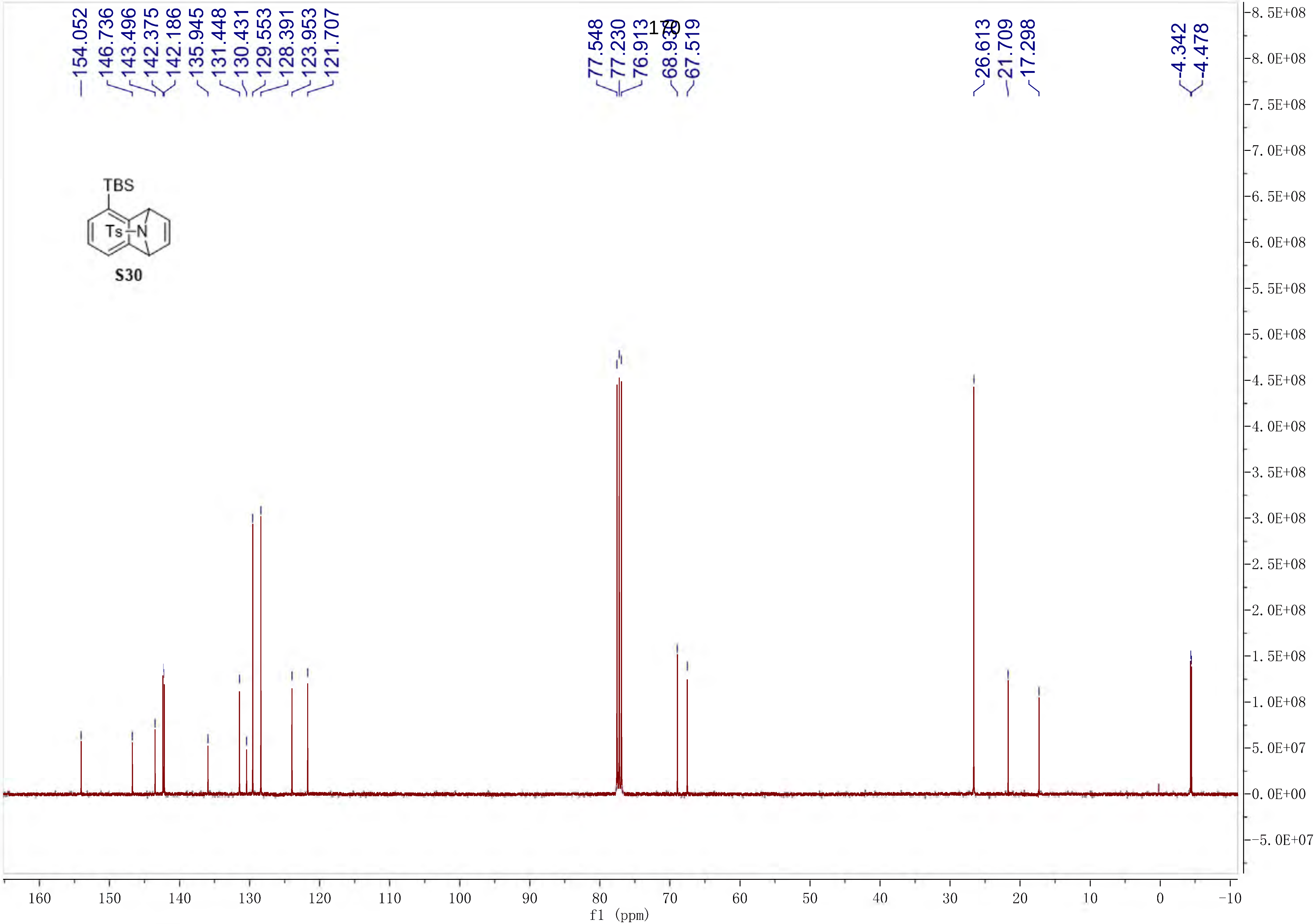

Supplementary Fig 98. ^{13}C NMR spectrum (400 MHz, $CDCl_3$, r.t.) of **S30**.

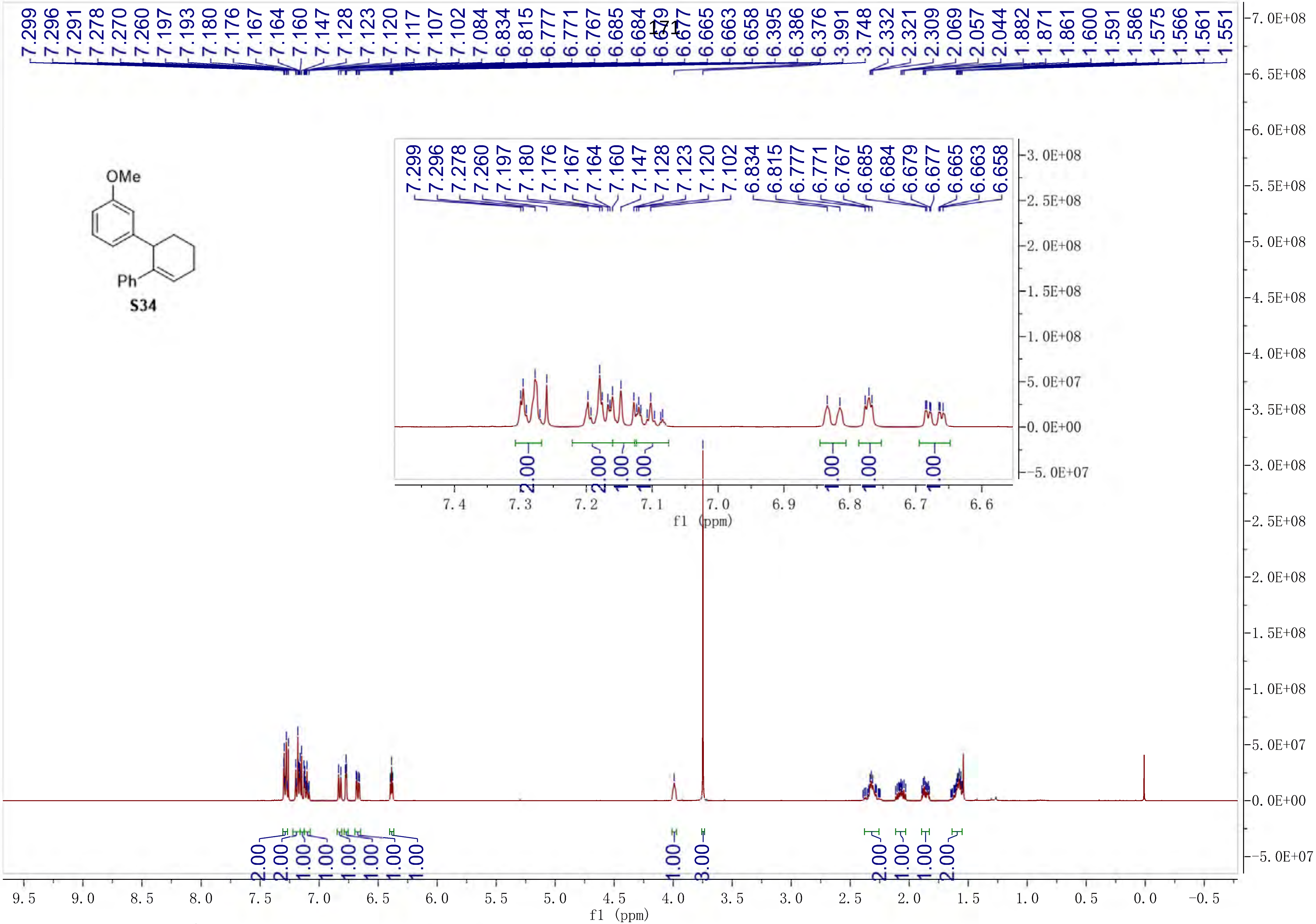

Supplementary Fig 99. ^{1}H NMR spectrum (400 MHz, $CDCl_3$, r.t.) of **S34**.

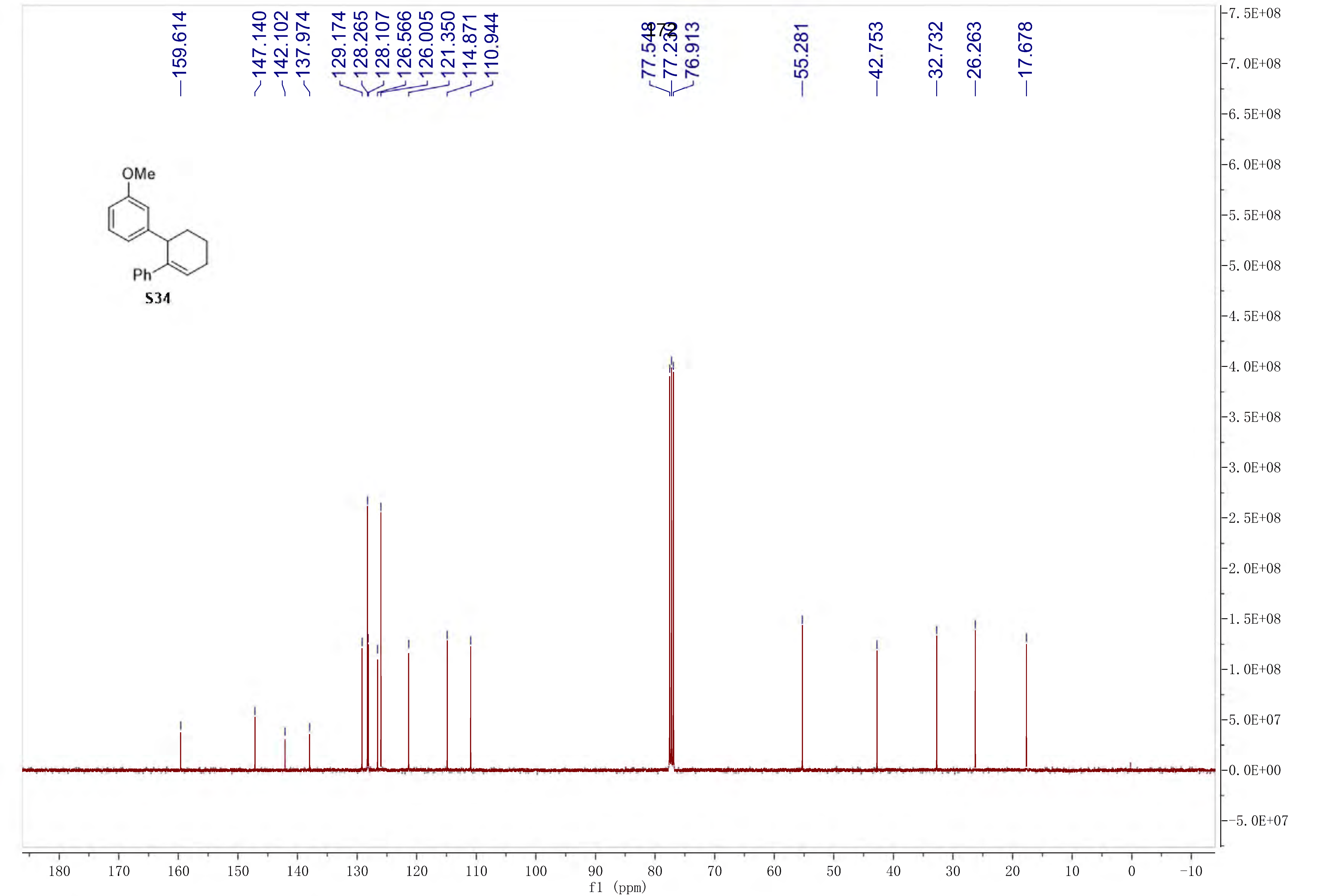

Supplementary Fig 100. ^{13}C NMR spectrum (400 MHz, $CDCl_3$, r.t.) of **S34**.

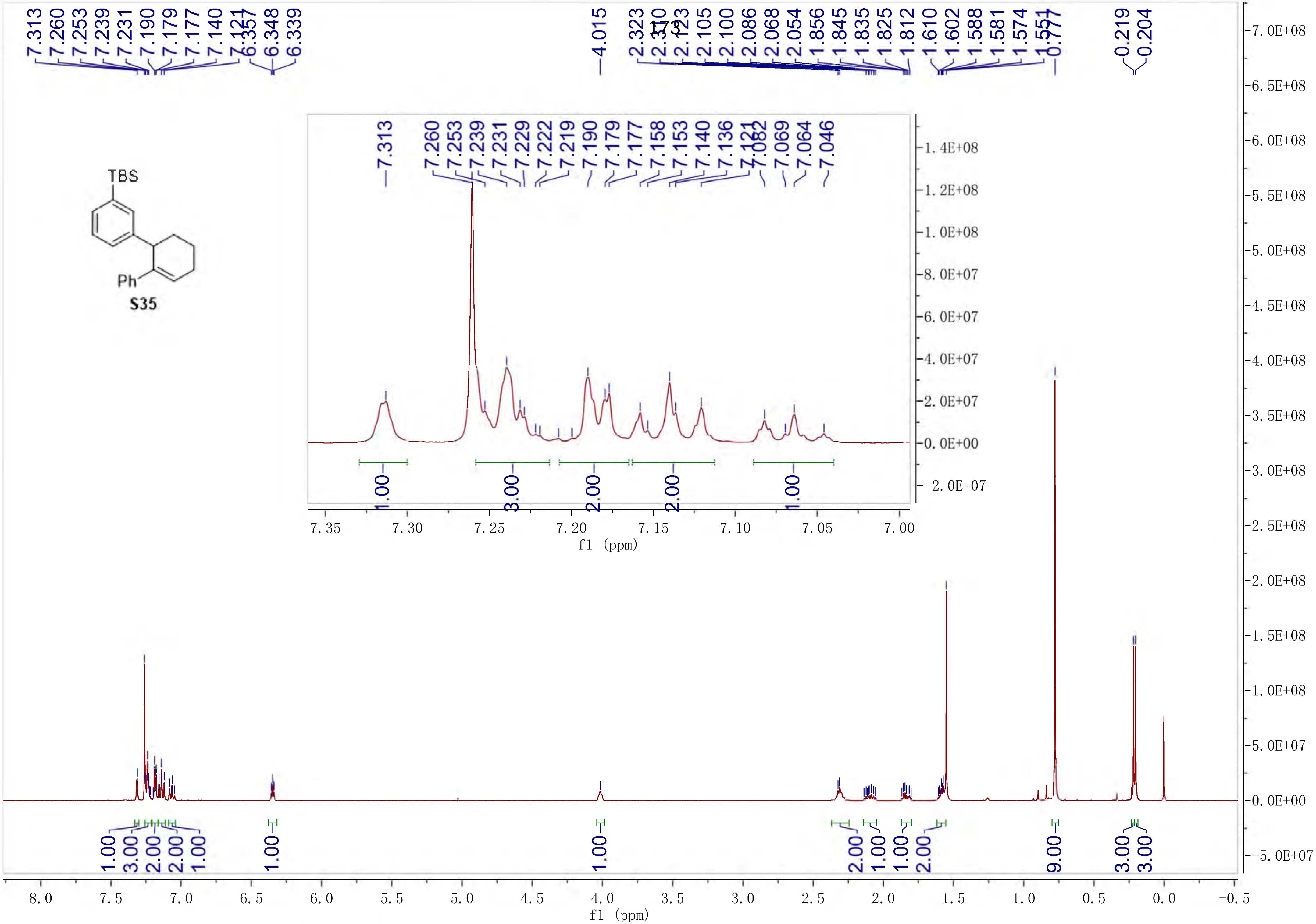

Supplementary Fig 101. ^{1}H NMR spectrum (400 MHz, $CDCl_3$, r.t.) of **S35**.

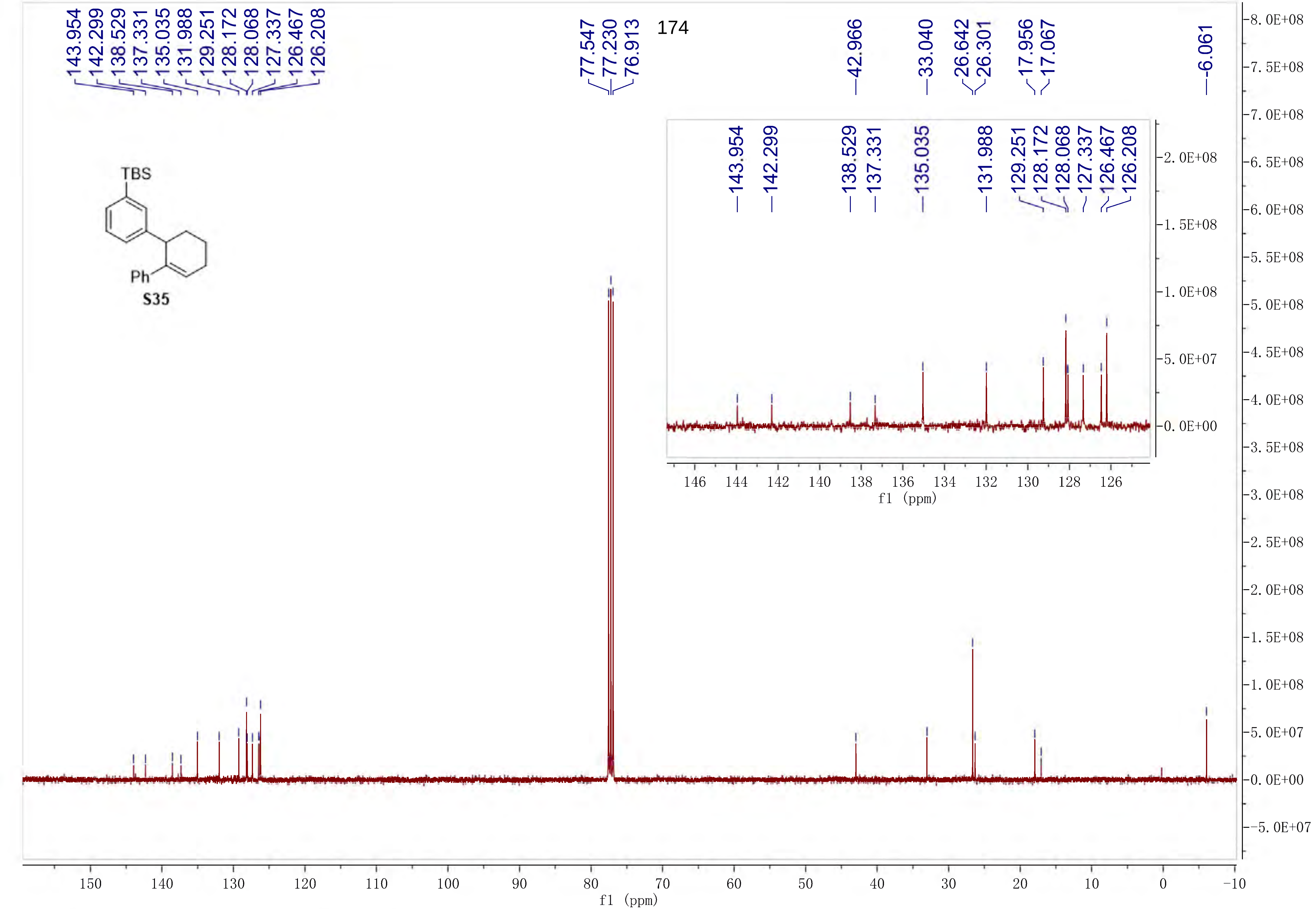

Supplementary Fig 102. ^{13}C NMR spectrum (400 MHz, $CDCl_3$, r.t.) of **S35**.

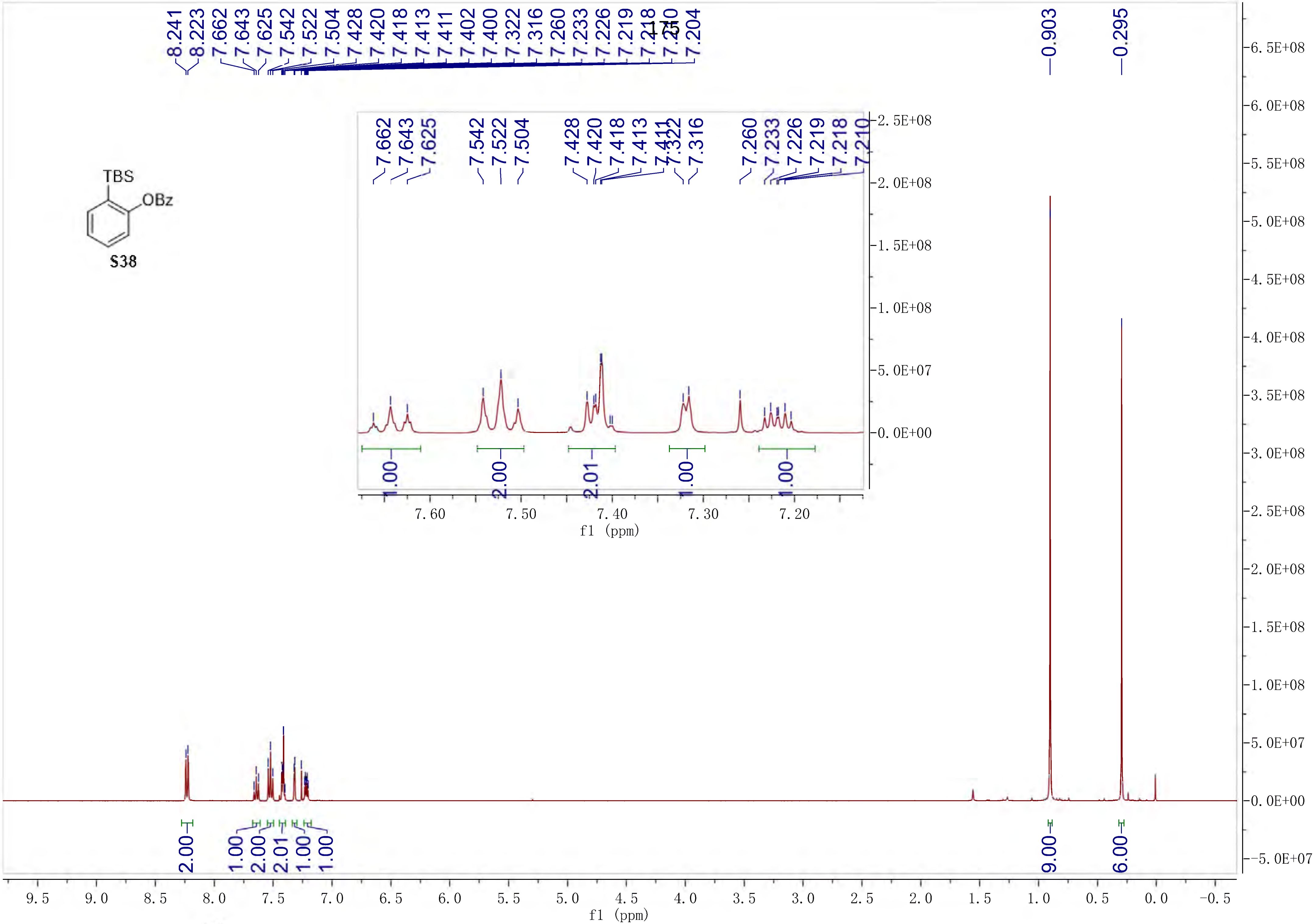

Supplementary Fig 103. ^{1}H NMR spectrum (400 MHz, $CDCl_3$, r.t.) of **S38**.

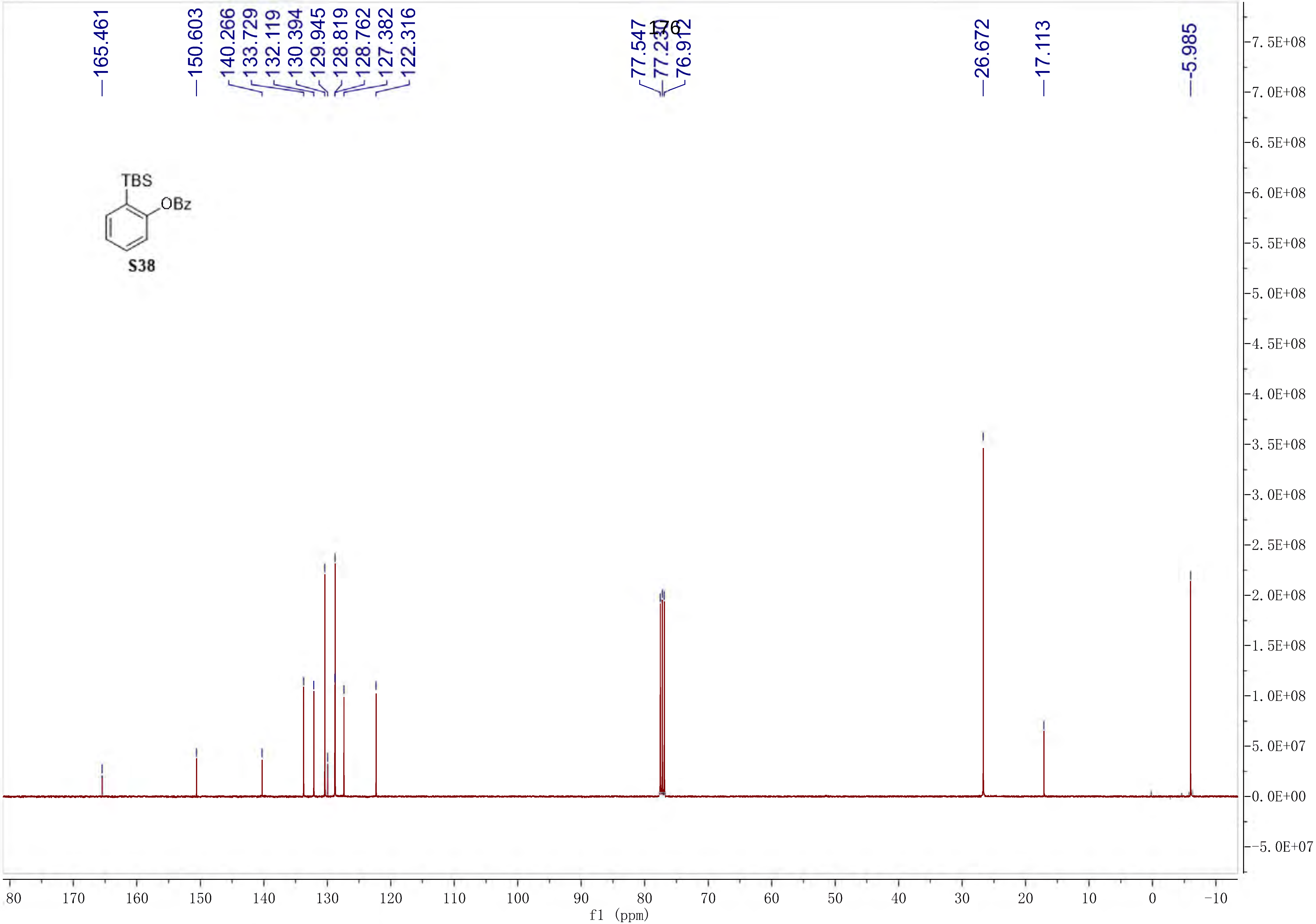

Supplementary Fig 104. ^{13}C NMR spectrum (400 MHz, $CDCl_3$, r.t.) of **S38**.

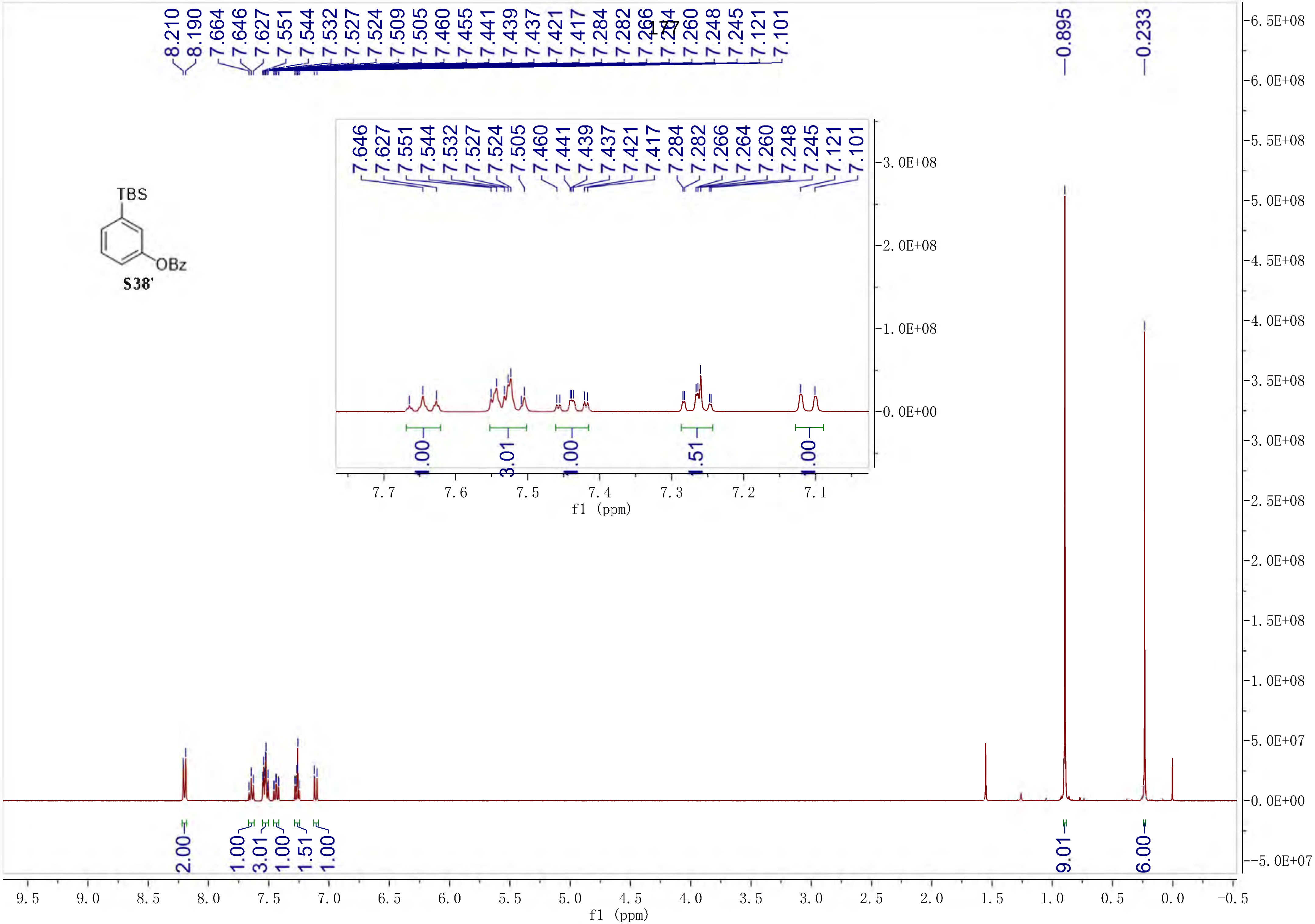

Supplementary Fig 105. ^{1}H NMR spectrum (400 MHz, $CDCl_3$, r.t.) of **S38’**.

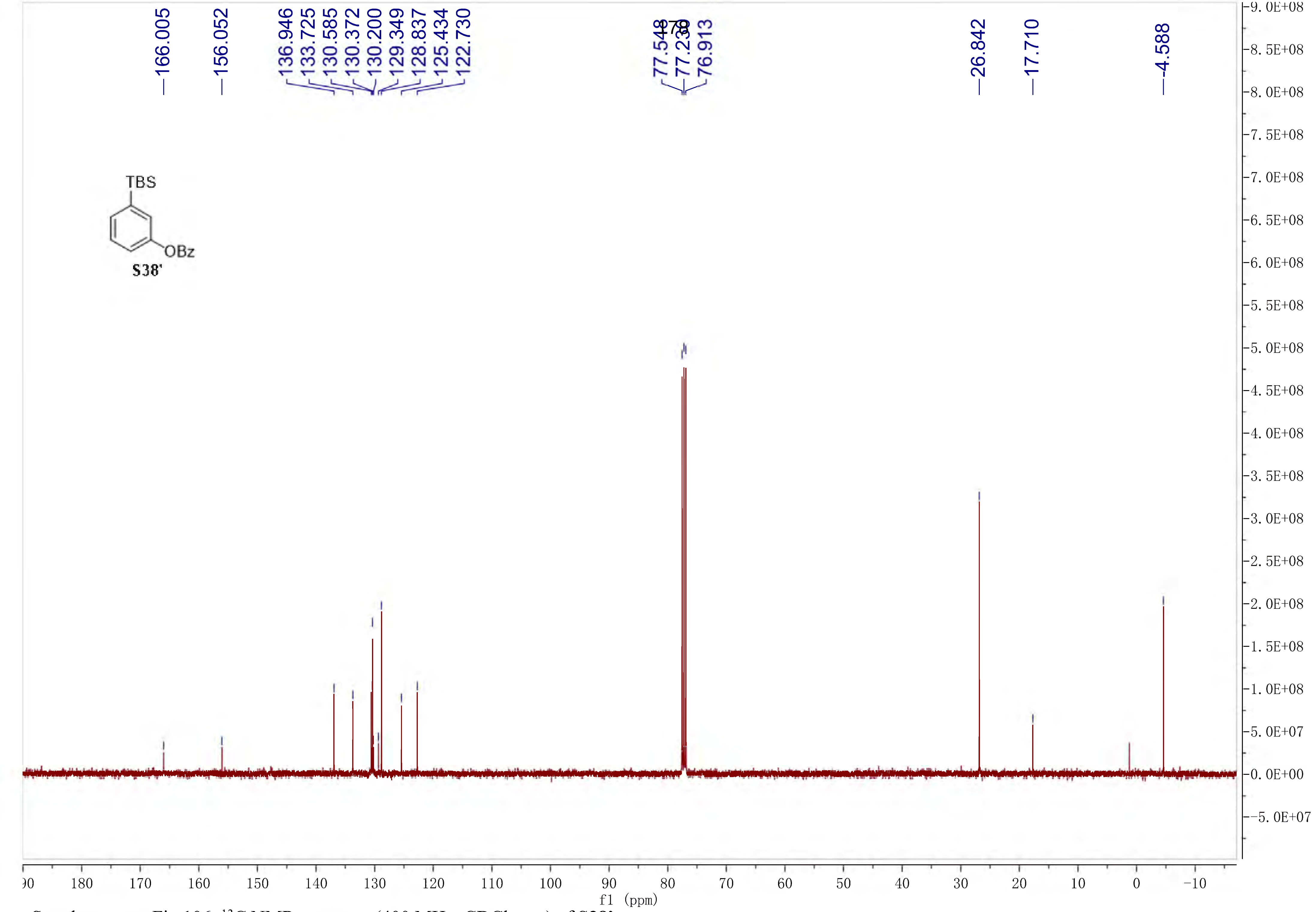

Supplementary Fig 106. ^{13}C NMR spectrum (400 MHz, $CDCl_3$, r.t.) of **S38’**.

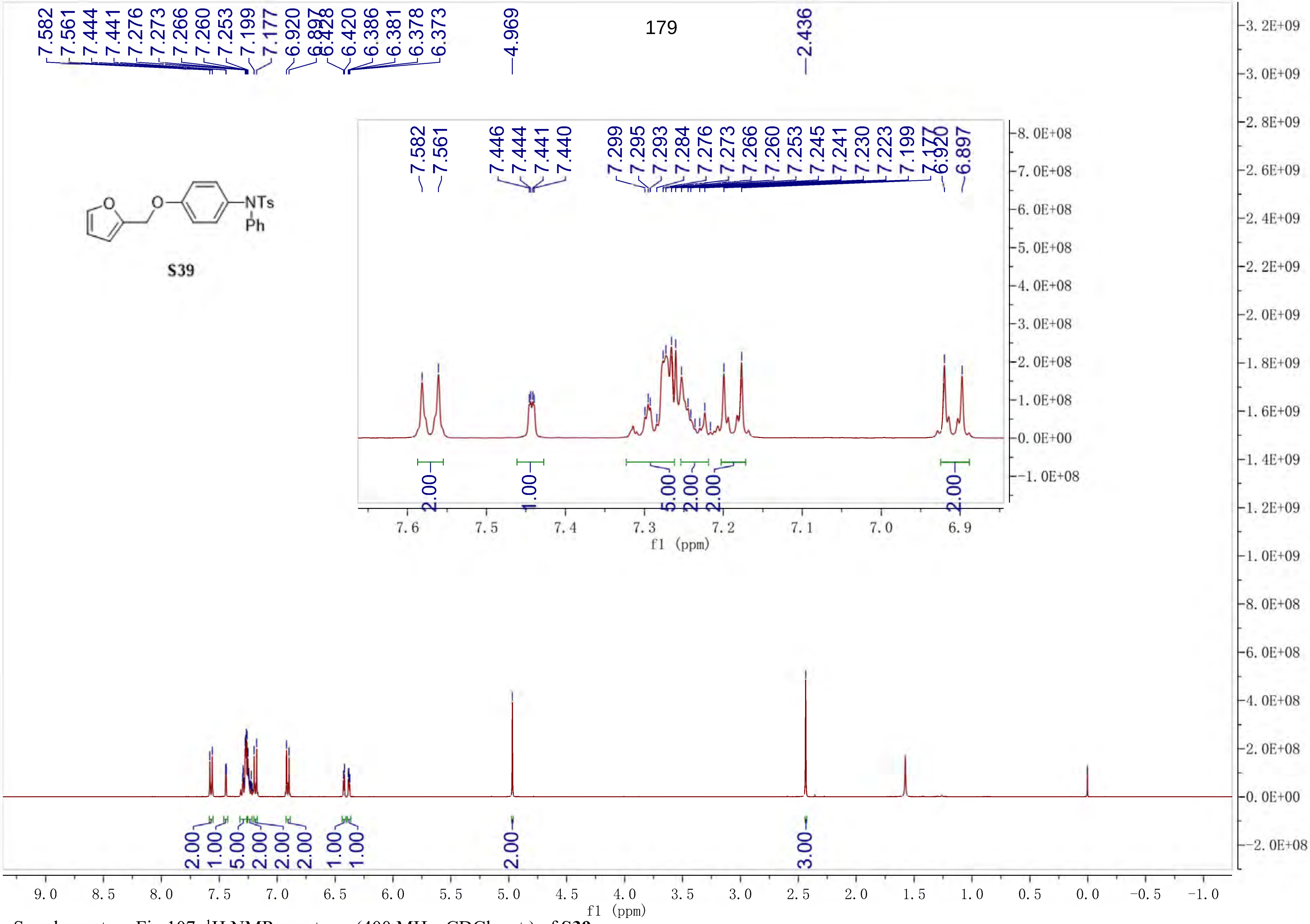

Supplementary Fig 107. ^{1}H NMR spectrum (400 MHz, $CDCl_3$, r.t.) of **S39**.

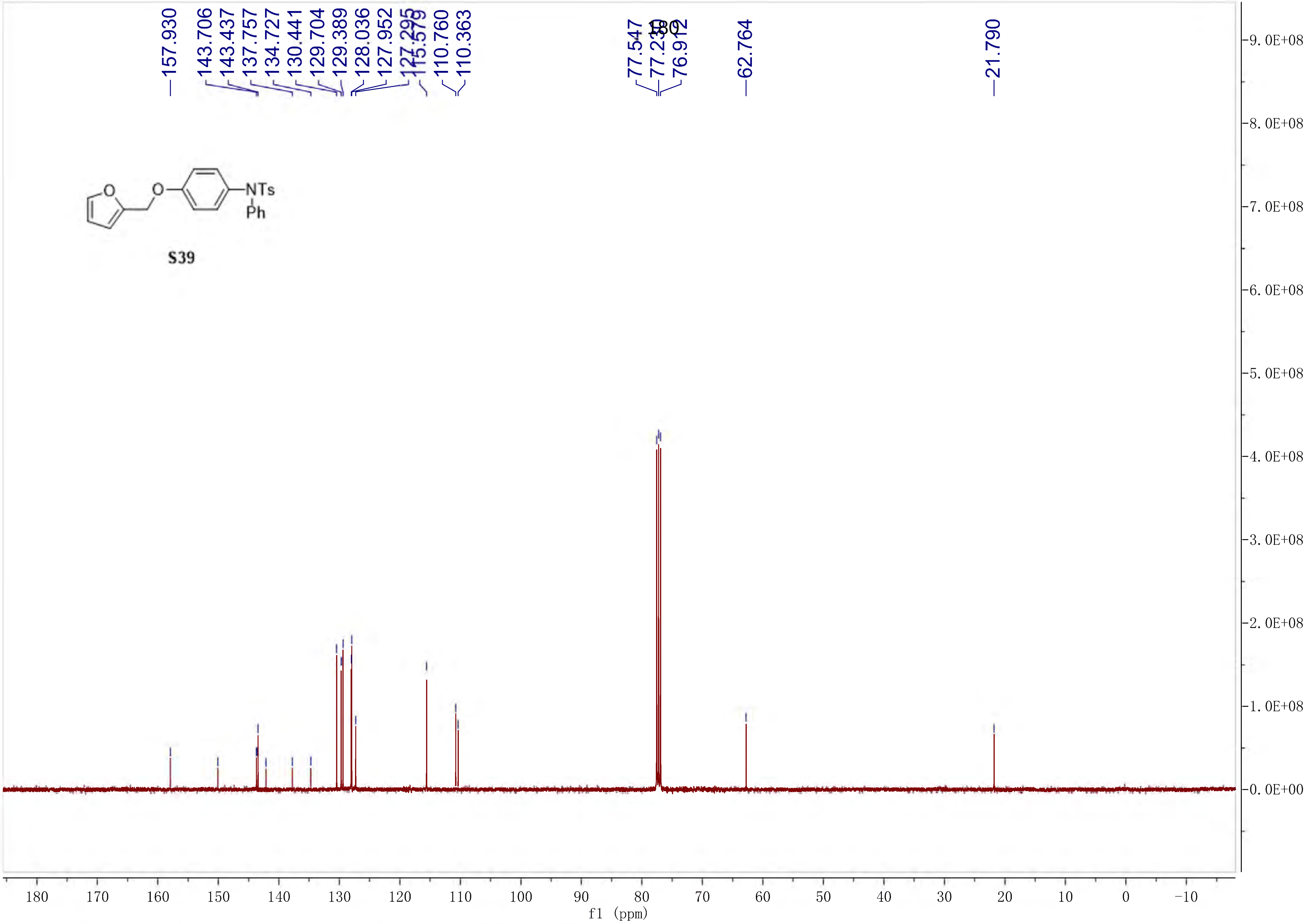

Supplementary Fig 108. ^{13}C NMR spectrum (400 MHz, $CDCl_3$, r.t.) of **S39**.

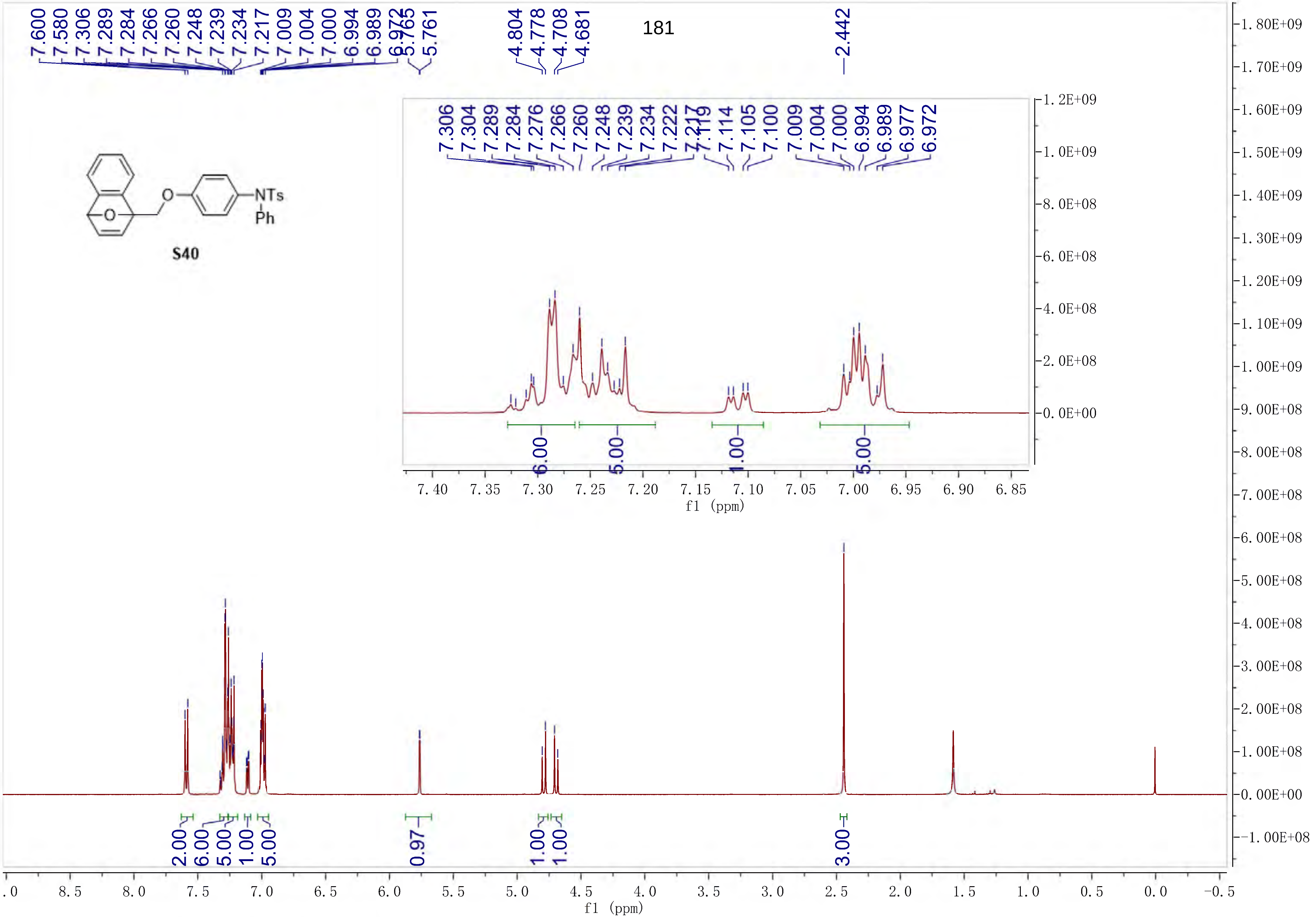

Supplementary Fig 109. ^{1}H NMR spectrum (400 MHz, $CDCl_3$, r.t.) of **S40**.

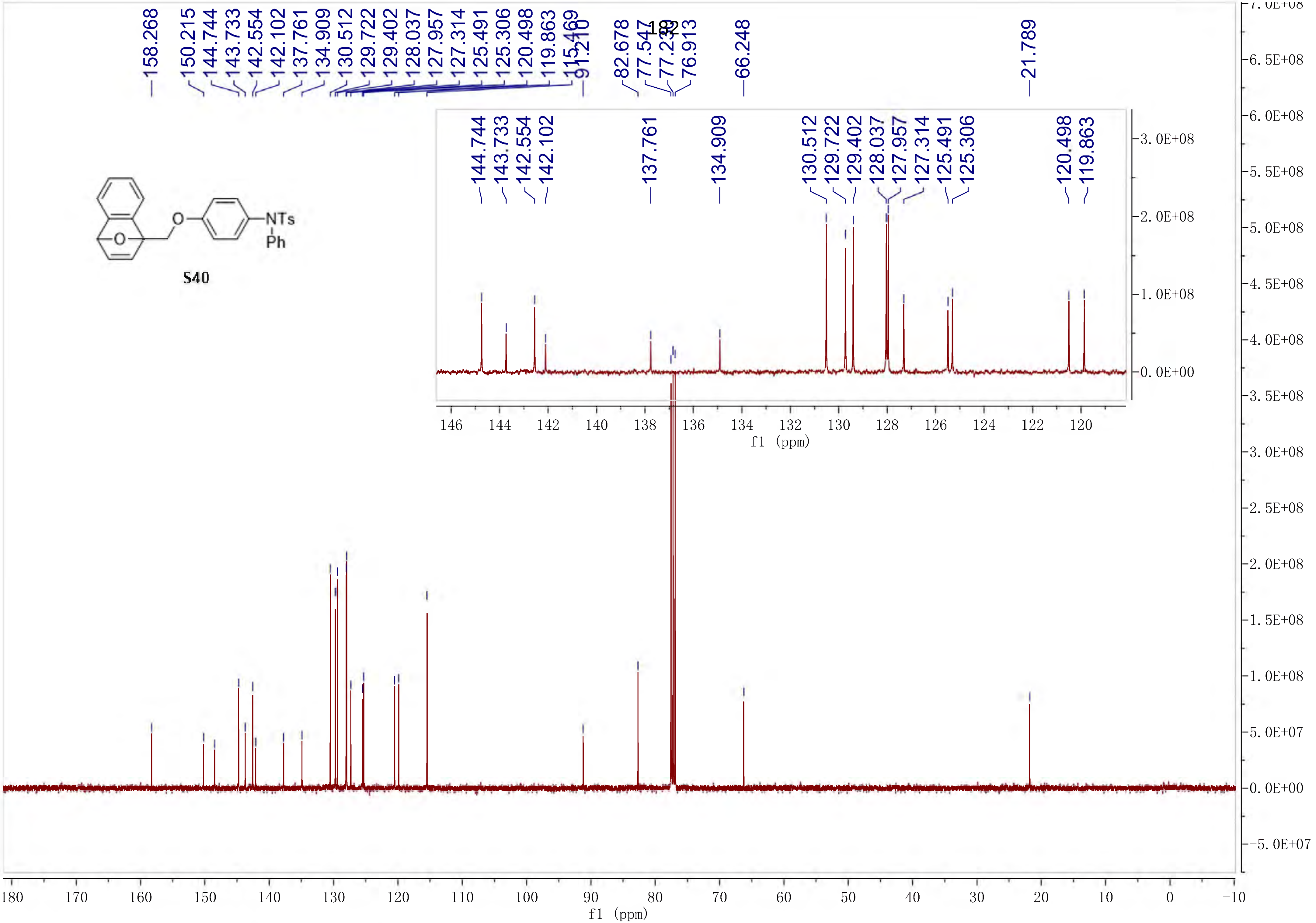

Supplementary Fig 110. ^{13}C NMR spectrum (400 MHz, $CDCl_3$, r.t.) of **S40**.

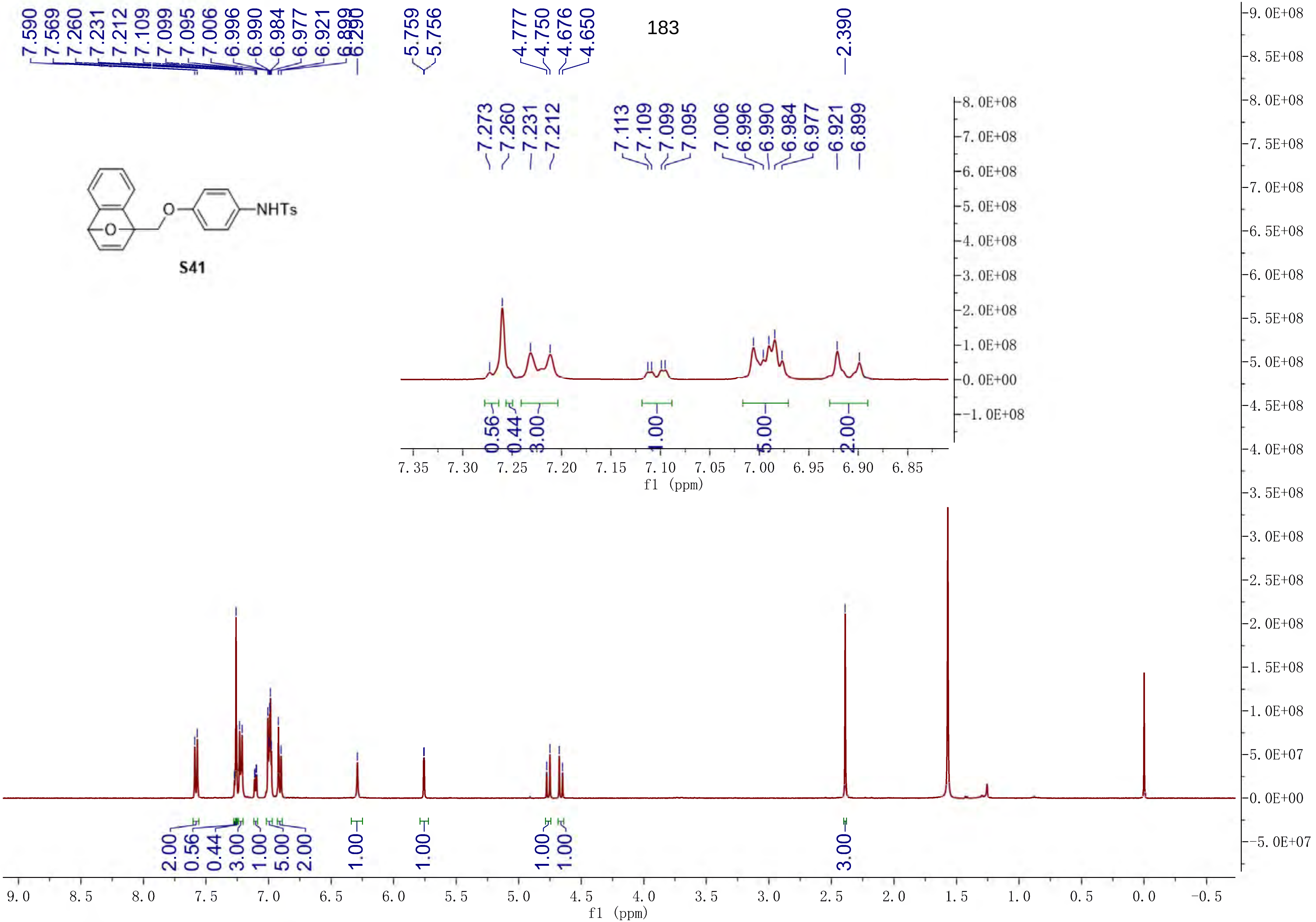

Supplementary Fig 111. ^{1}H NMR spectrum (400 MHz, $CDCl_3$, r.t.) of **S41**.

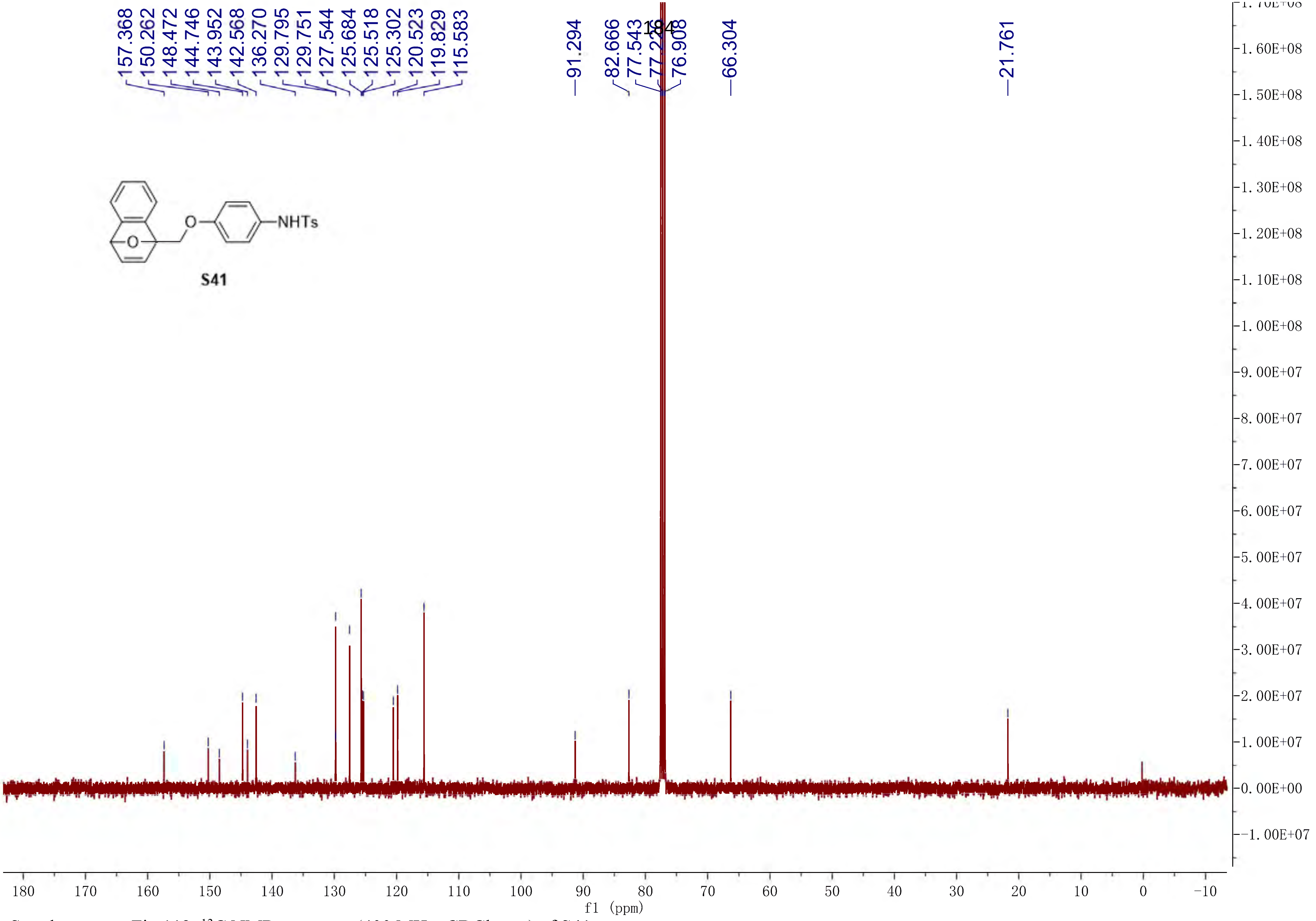

Supplementary Fig 112. ^{13}C NMR spectrum (400 MHz, $CDCl_3$, r.t.) of **S41**.

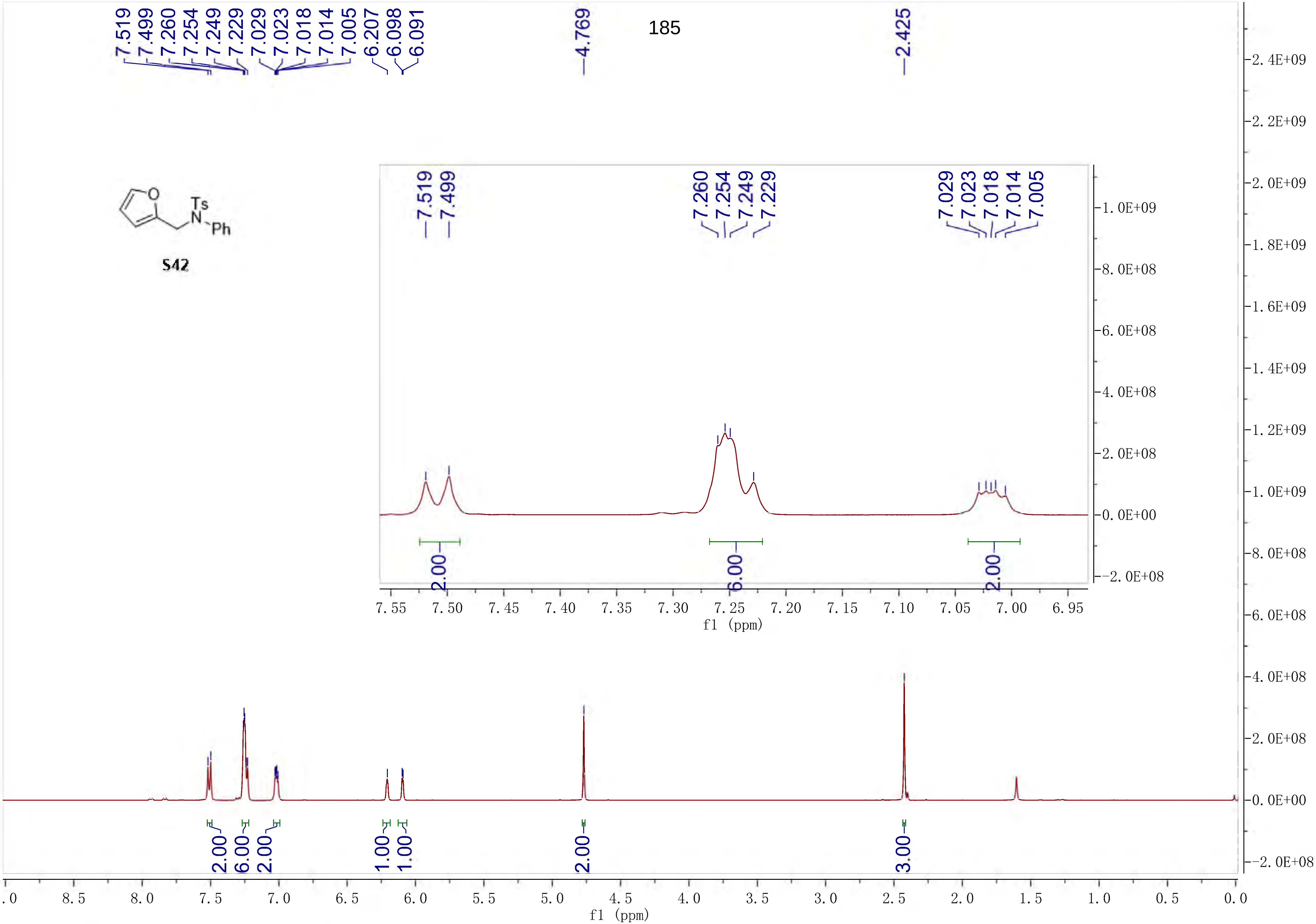

Supplementary Fig 113. ^{1}H NMR spectrum (400 MHz, $CDCl_3$, r.t.) of **S42**.

186

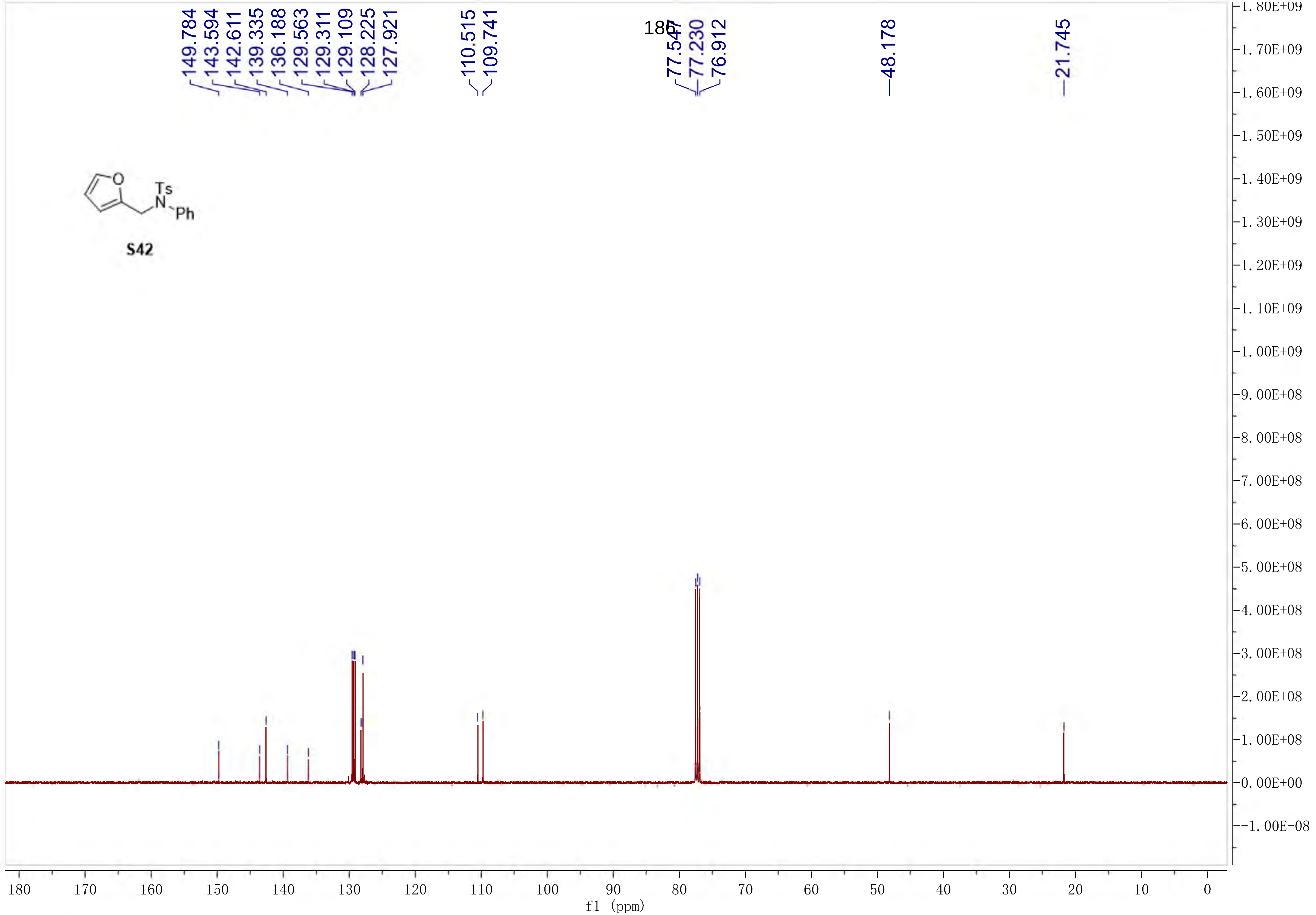

Supplementary Fig 114. ^{13}C NMR spectrum (400 MHz, $CDCl_3$, r.t.) of **S42**.

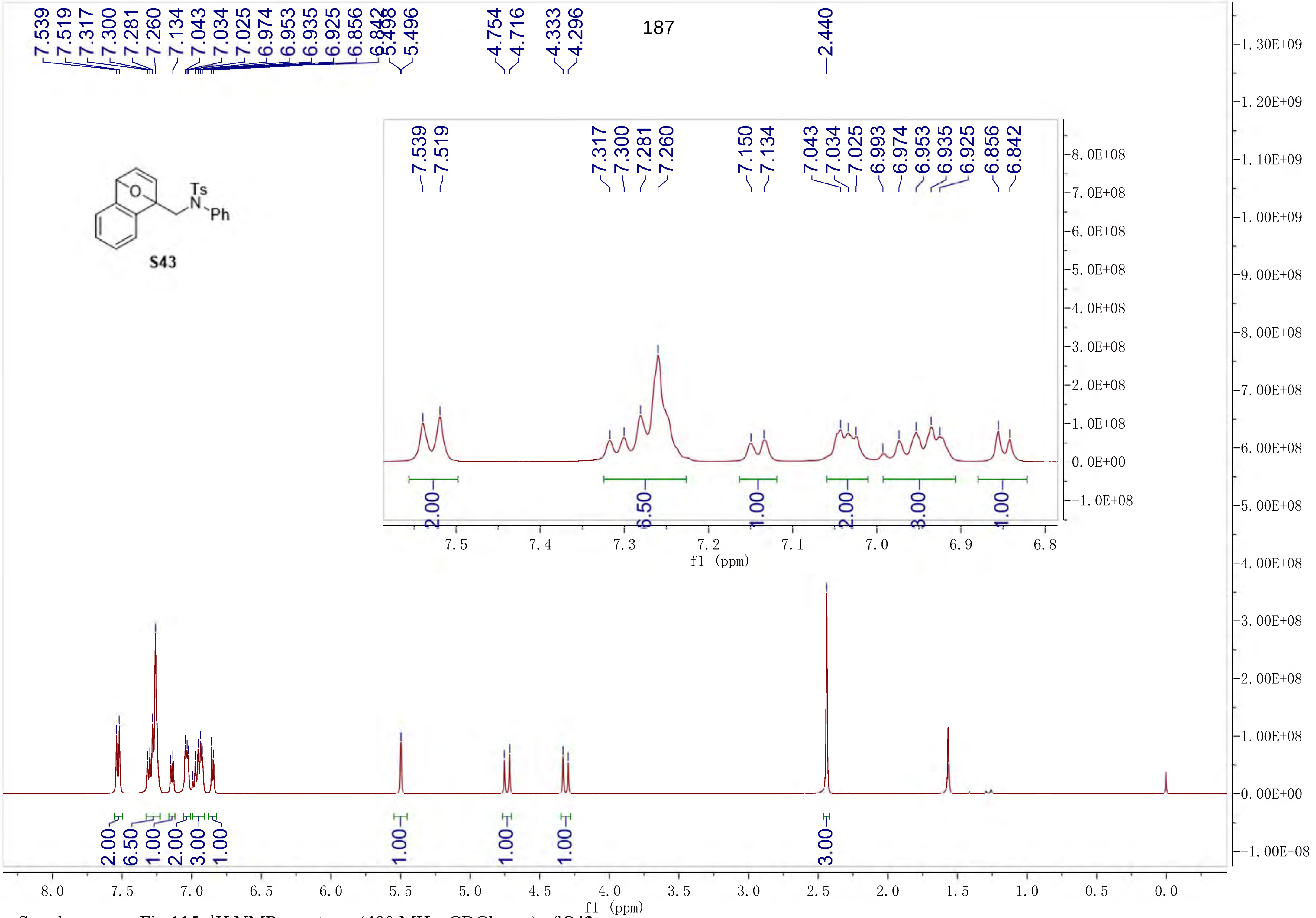

Supplementary Fig 115. ^{1}H NMR spectrum (400 MHz, $CDCl_3$, r.t.) of **S43**.

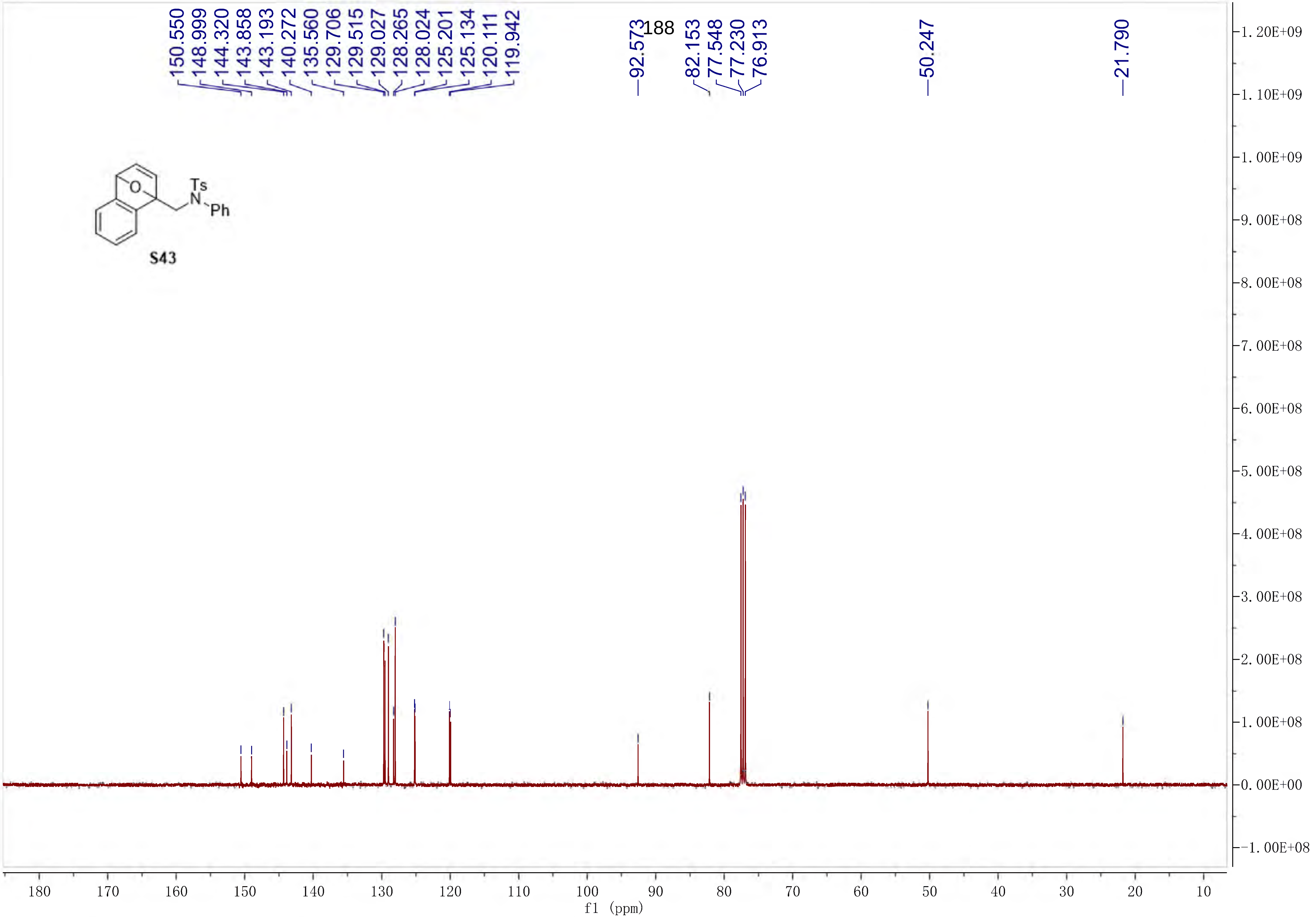

Supplementary Fig 116. ^{13}C NMR spectrum (400 MHz, $CDCl_3$, r.t.) of **S43**.

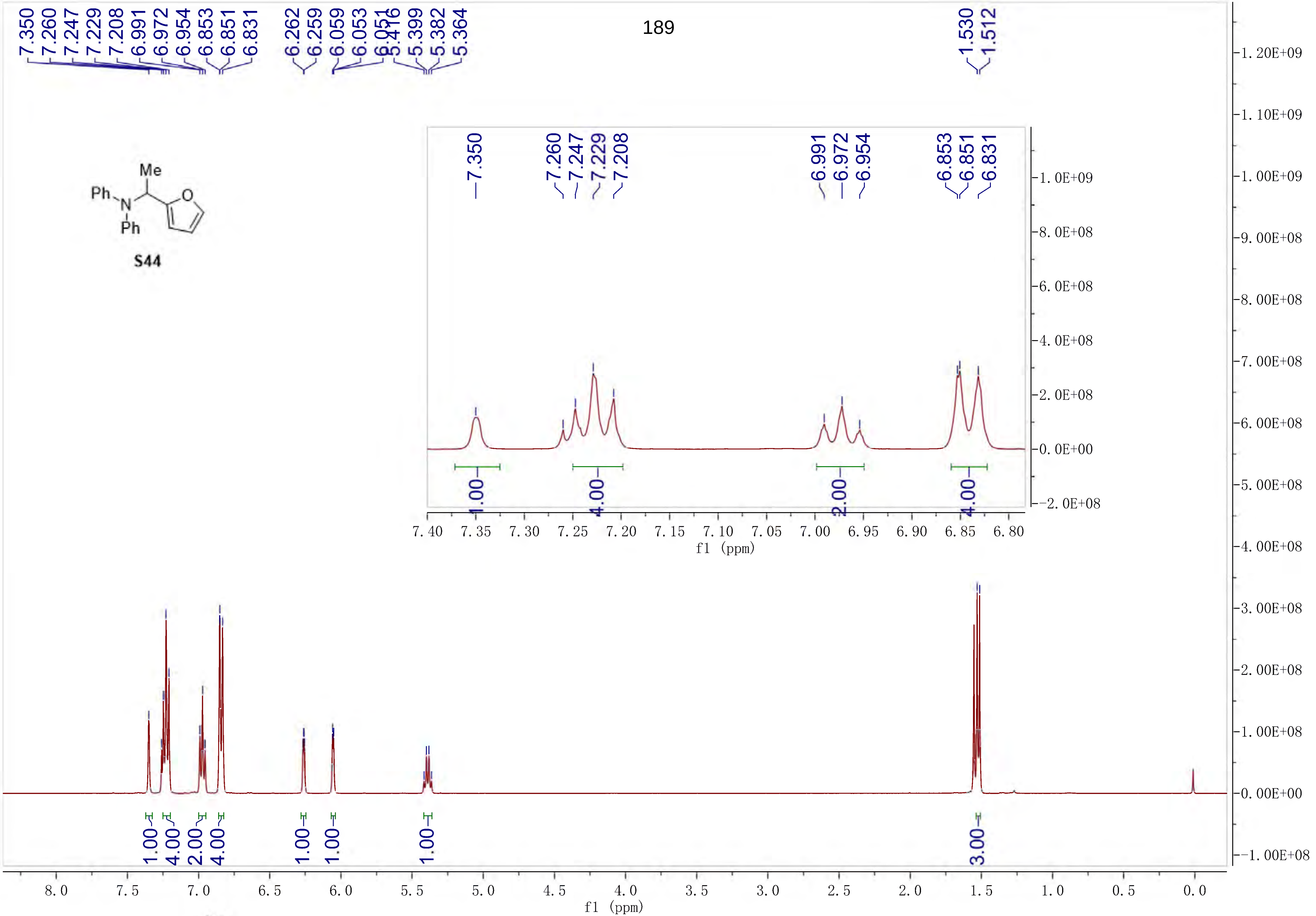

Supplementary Fig 117. ^{1}H NMR spectrum (400 MHz, $CDCl_3$, r.t.) of **S44**.

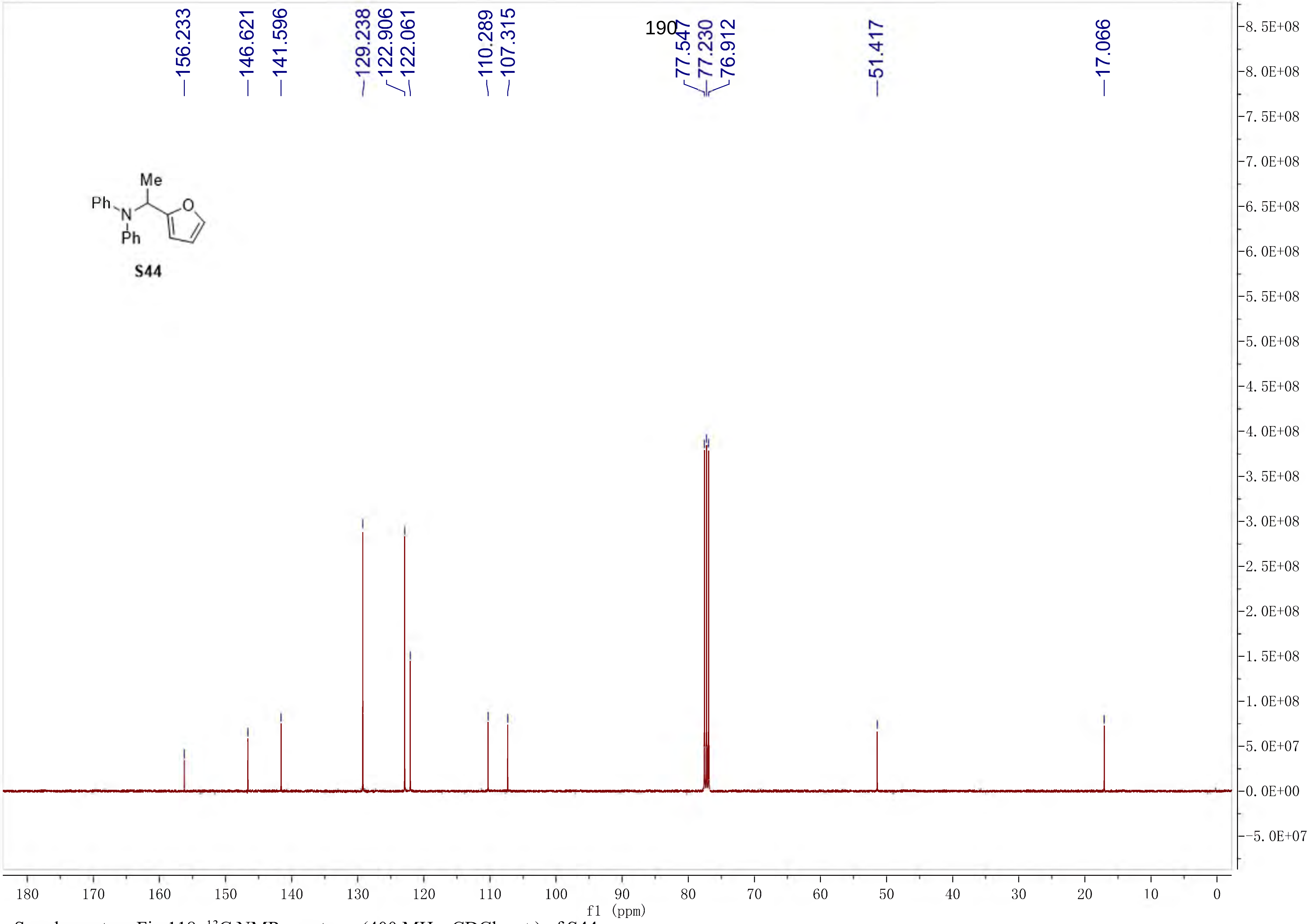

Supplementary Fig 118. ^{13}C NMR spectrum (400 MHz, $CDCl_3$, r.t.) of **S44**.

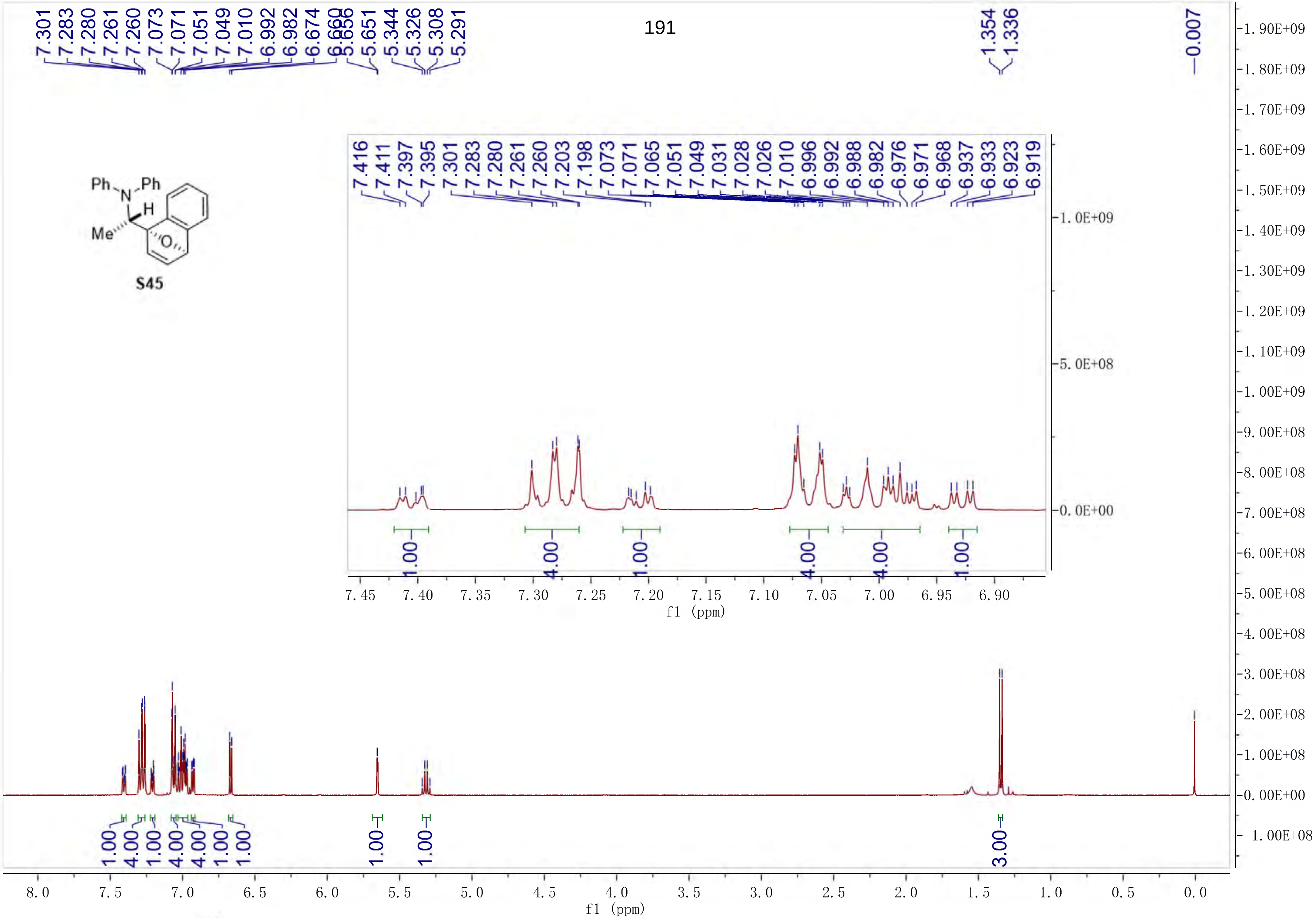

Supplementary Fig 119. ^{1}H NMR spectrum (400 MHz, $CDCl_3$, r.t.) of **S45**.

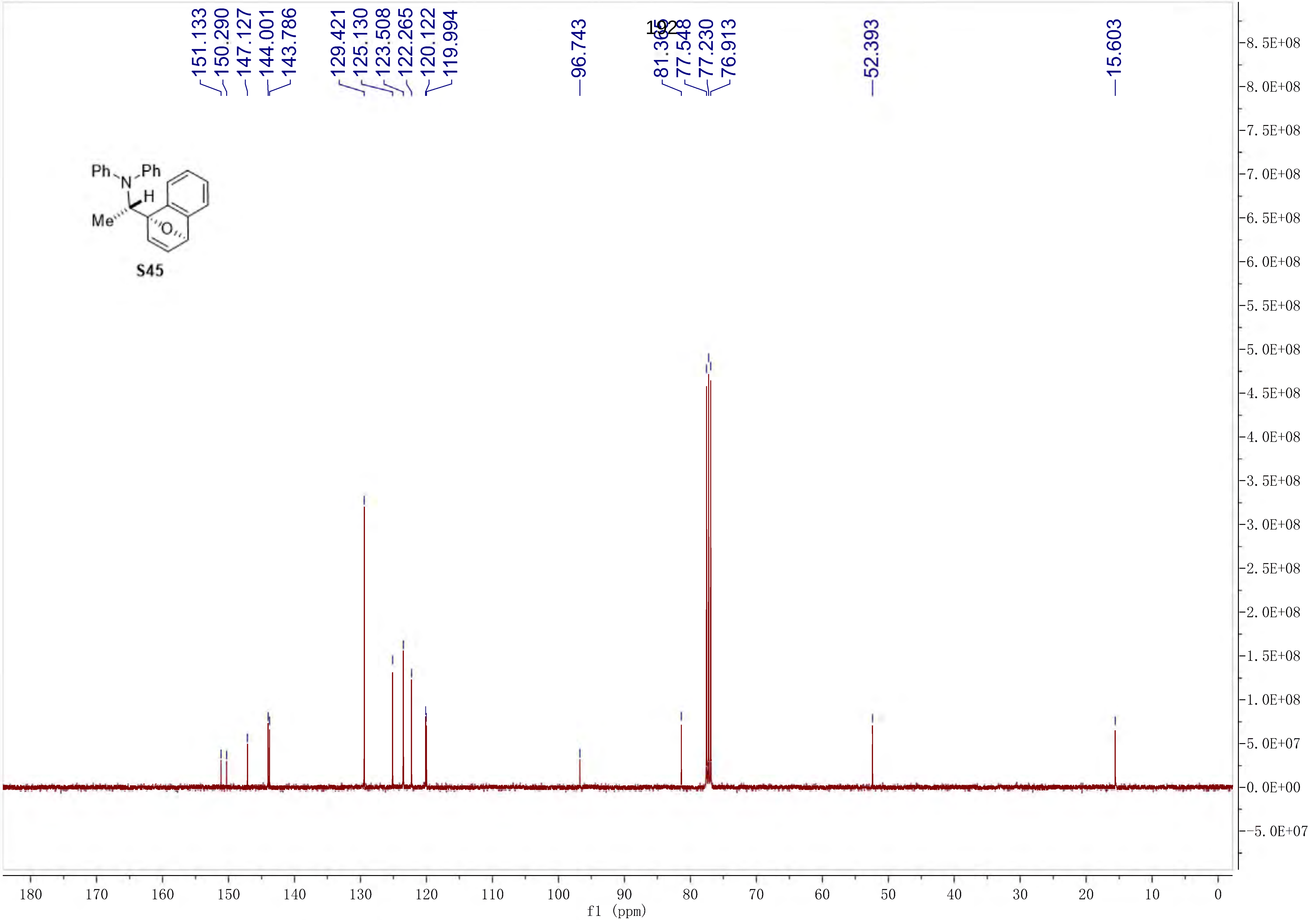

Supplementary Fig 120. ^{13}C NMR spectrum (400 MHz, $CDCl_3$, r.t.) of **S45**.

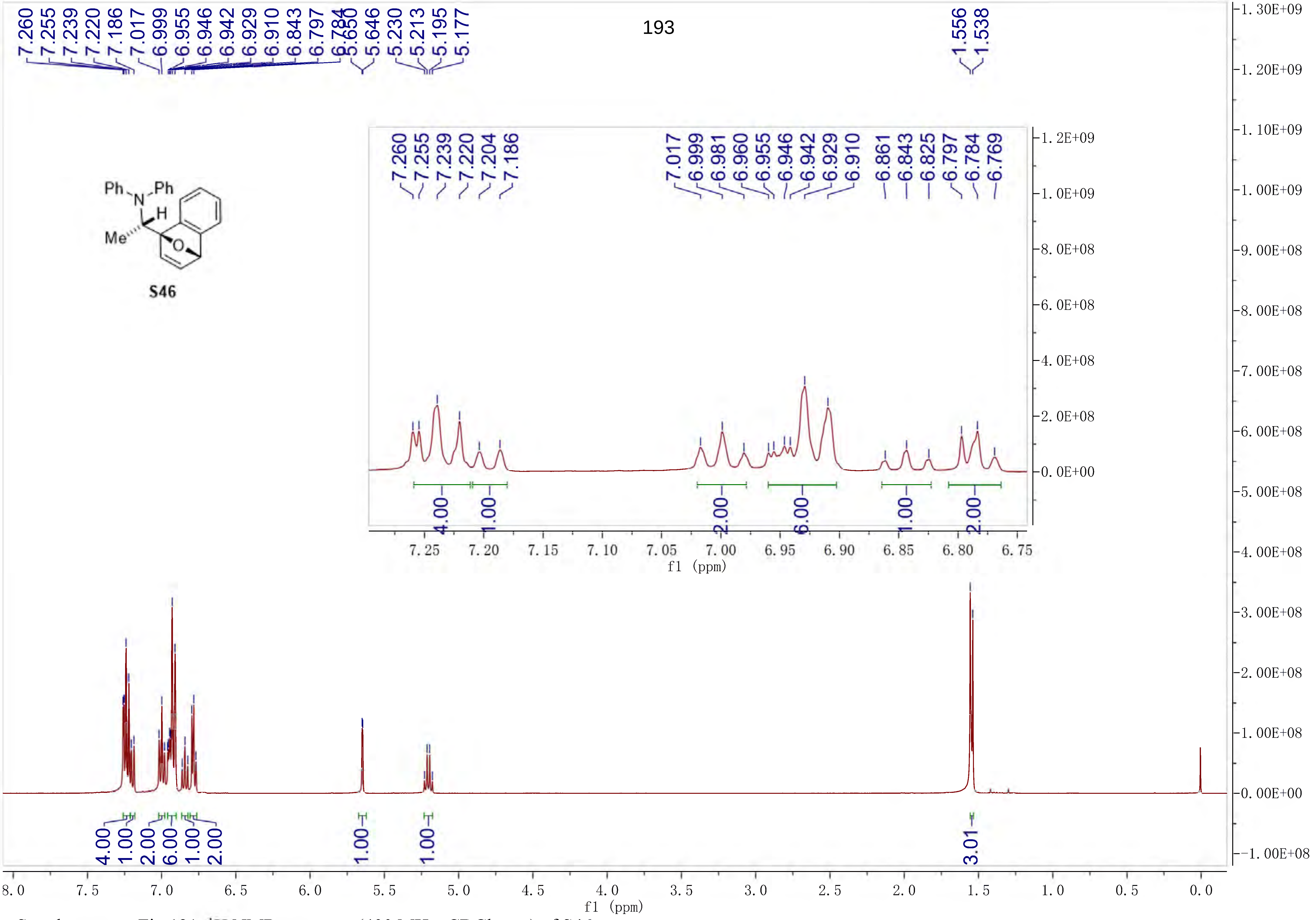

Supplementary Fig 121. ^{1}H NMR spectrum (400 MHz, $CDCl_3$, r.t.) of **S46**.

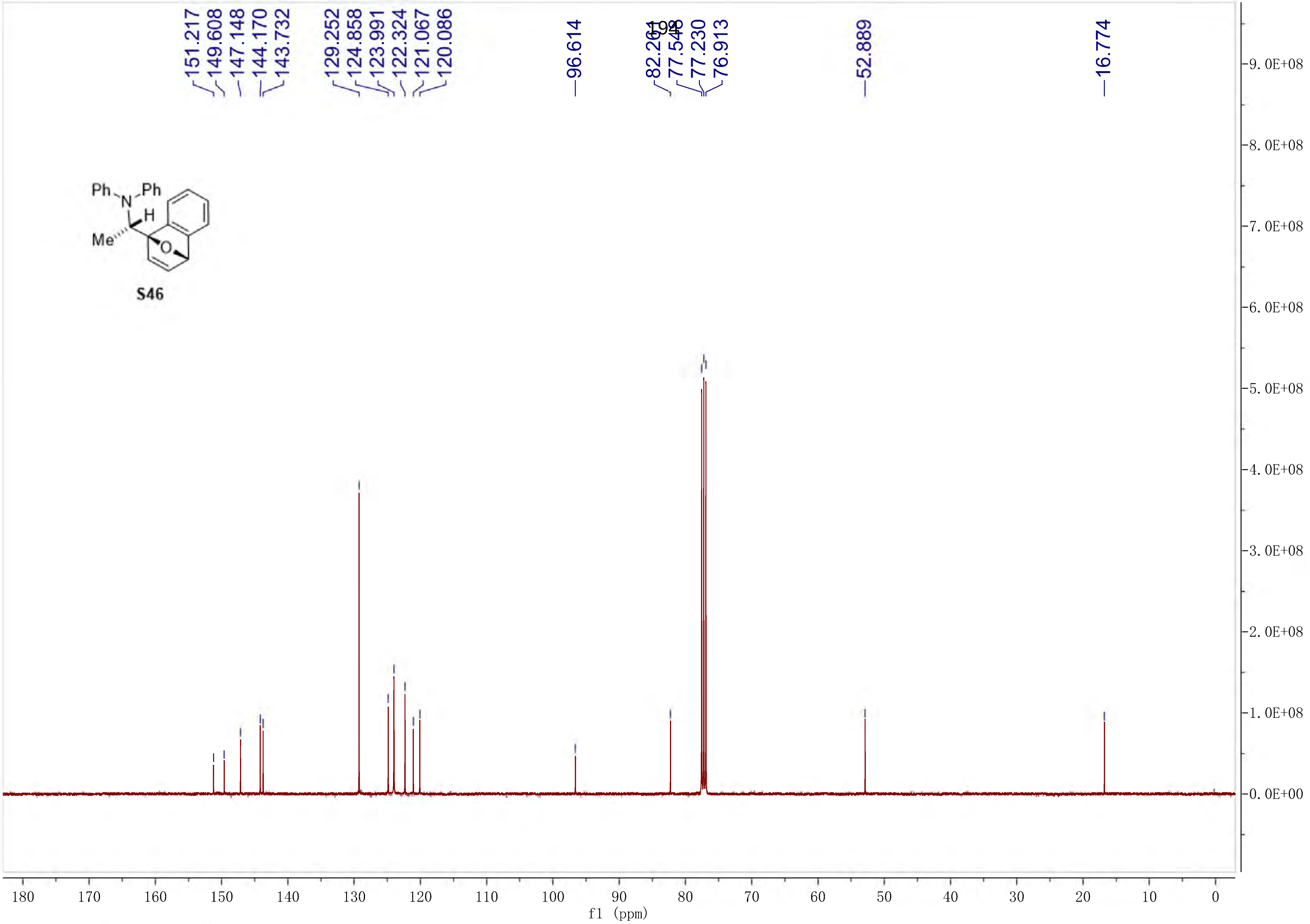

Supplementary Fig 122. ^{13}C NMR spectrum (400 MHz, $CDCl_3$, r.t.) of **S46**.

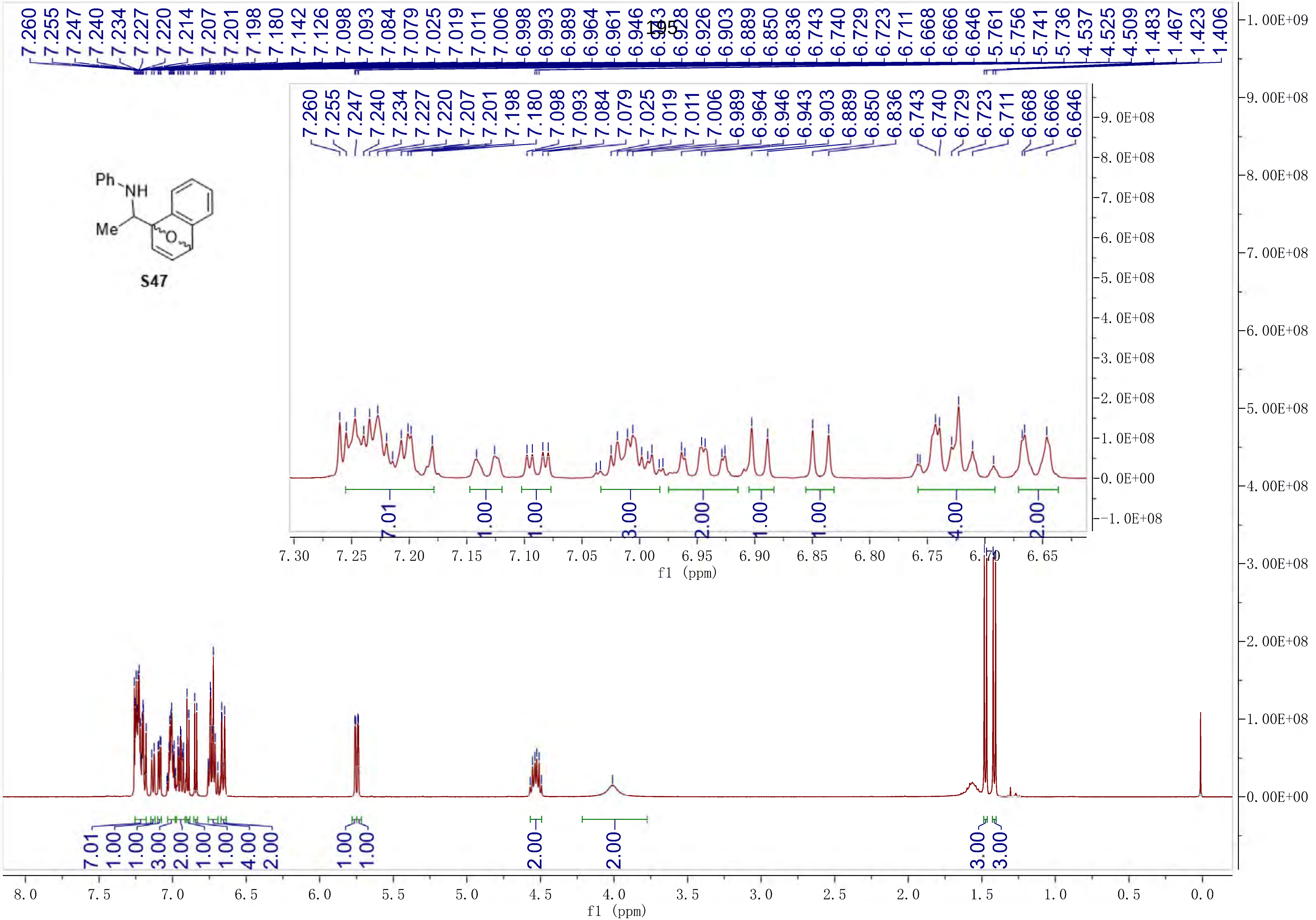

Supplementary Fig 123. ^{1}H NMR spectrum (400 MHz, $CDCl_3$, r.t.) of **S47**.

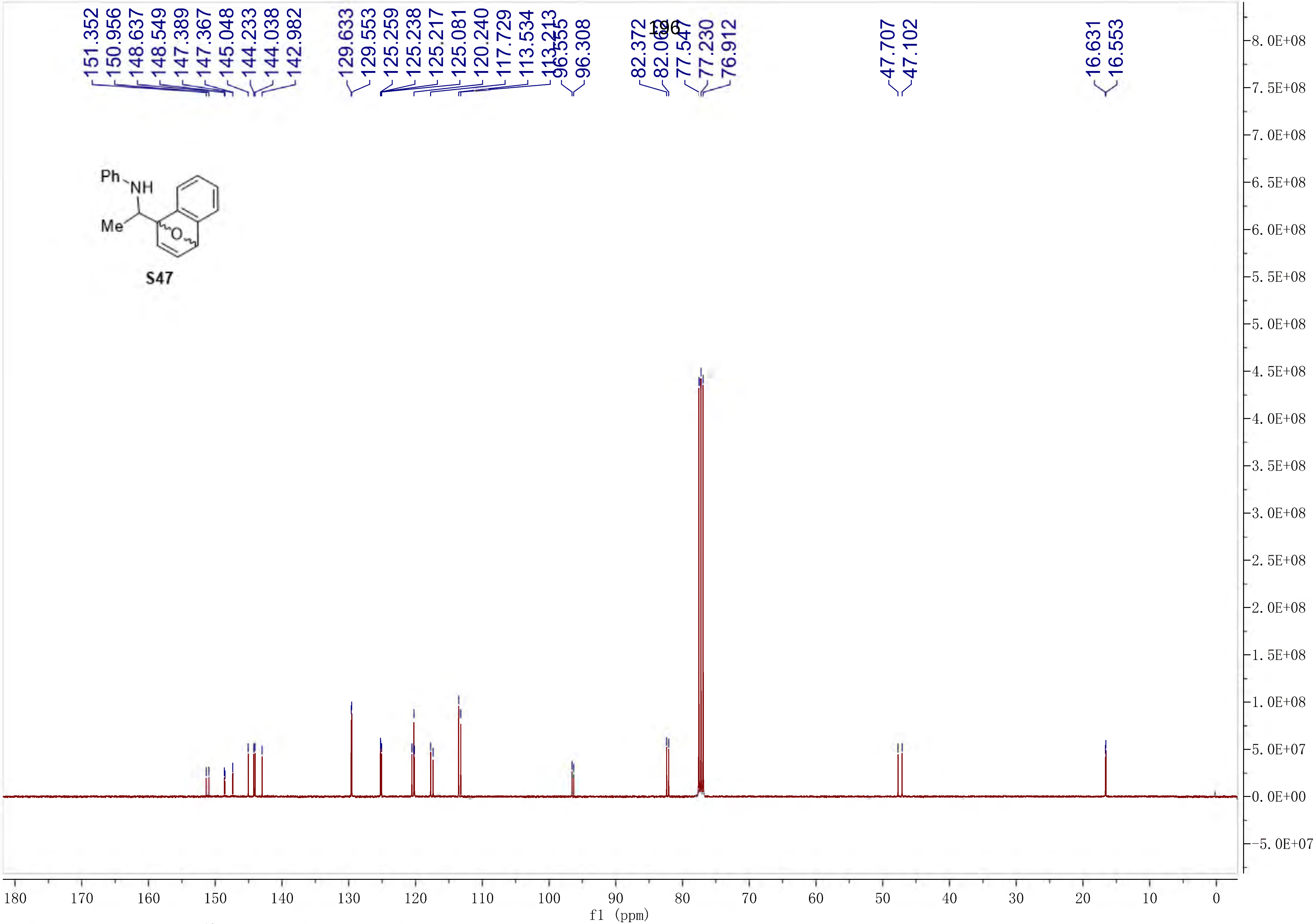

Supplementary Fig 124. ^{13}C NMR spectrum (400 MHz, $CDCl_3$, r.t.) of **S47**.

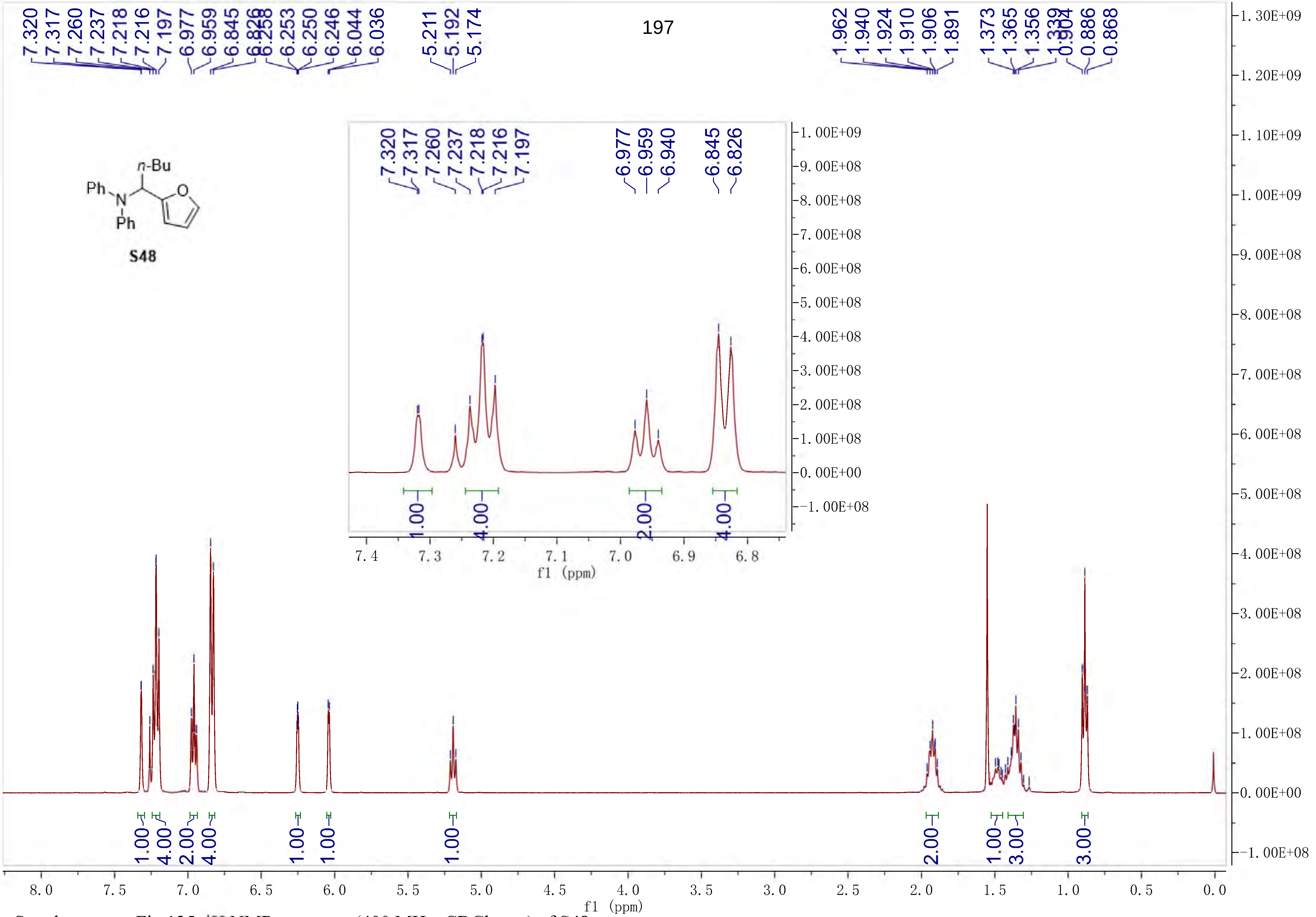

Supplementary Fig 125. ^{1}H NMR spectrum (400 MHz, $CDCl_3$, r.t.) of **S48**.

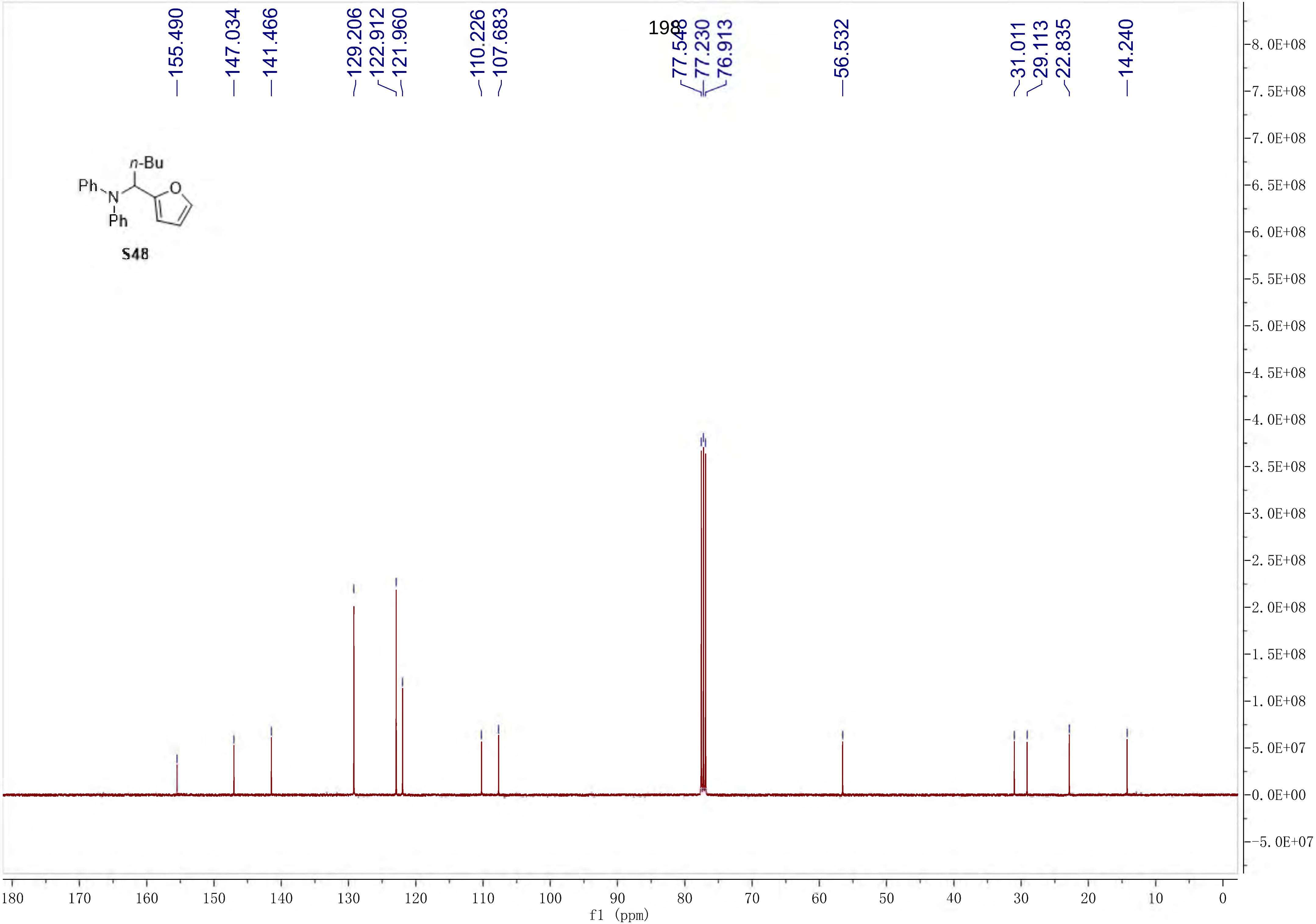

S48

Supplementary Fig 126. ^{13}C NMR spectrum (400 MHz, $CDCl_3$, r.t.) of **S48**.

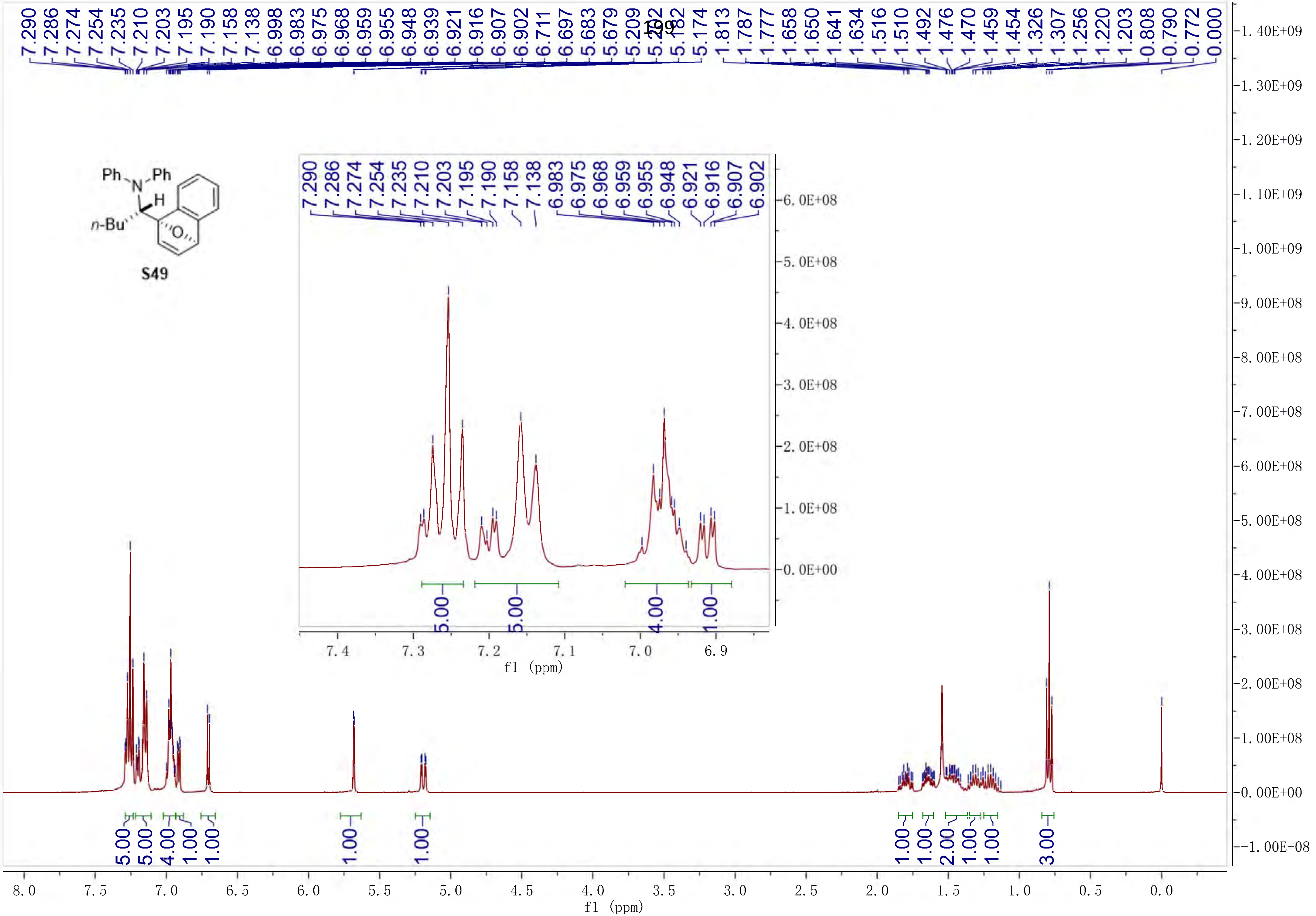

Supplementary Fig 127. ^{1}H NMR spectrum (400 MHz, $CDCl_3$, r.t.) of **S49**.

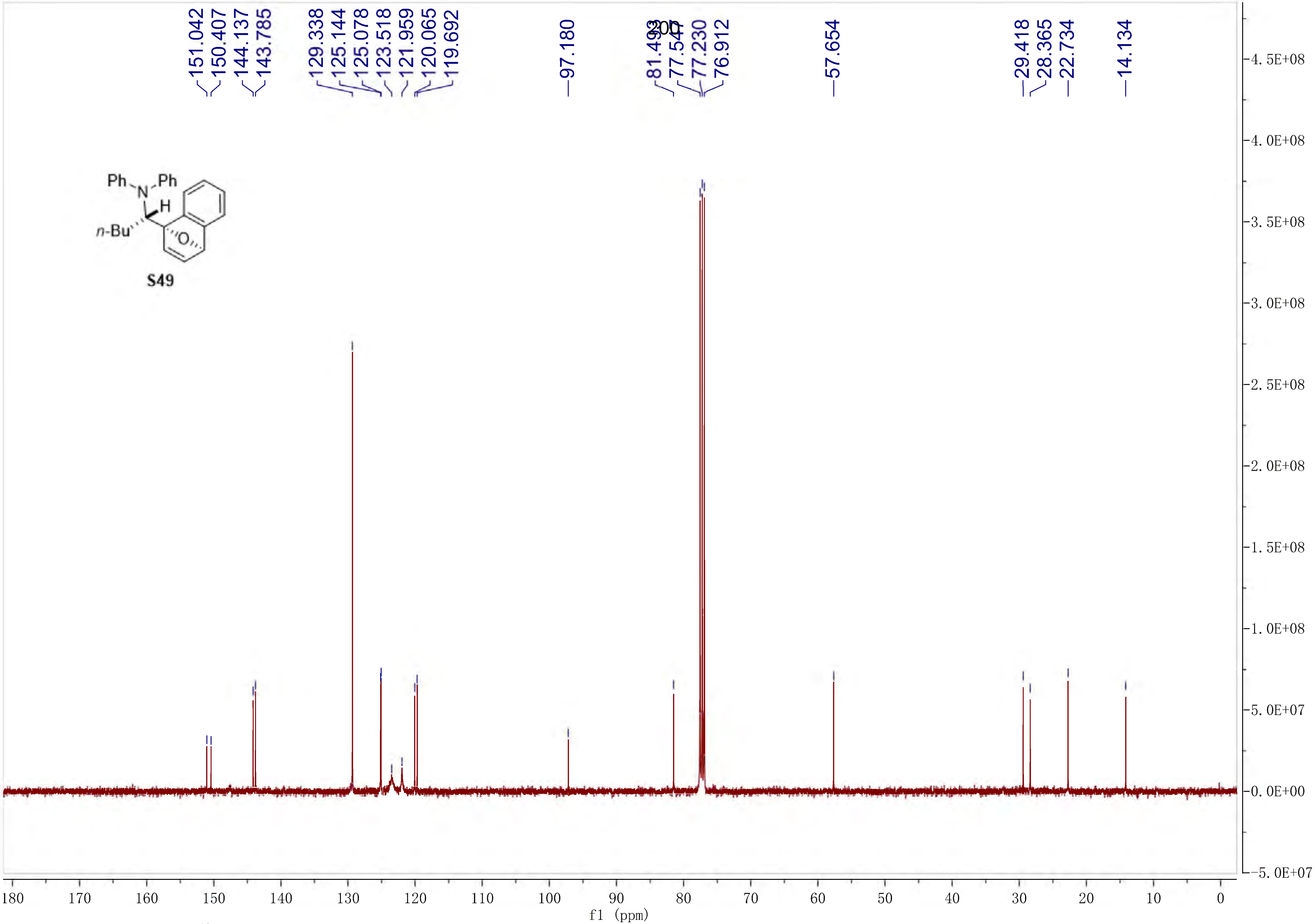

Supplementary Fig 128. ^{13}C NMR spectrum (400 MHz, $CDCl_3$, r.t.) of **S49**.

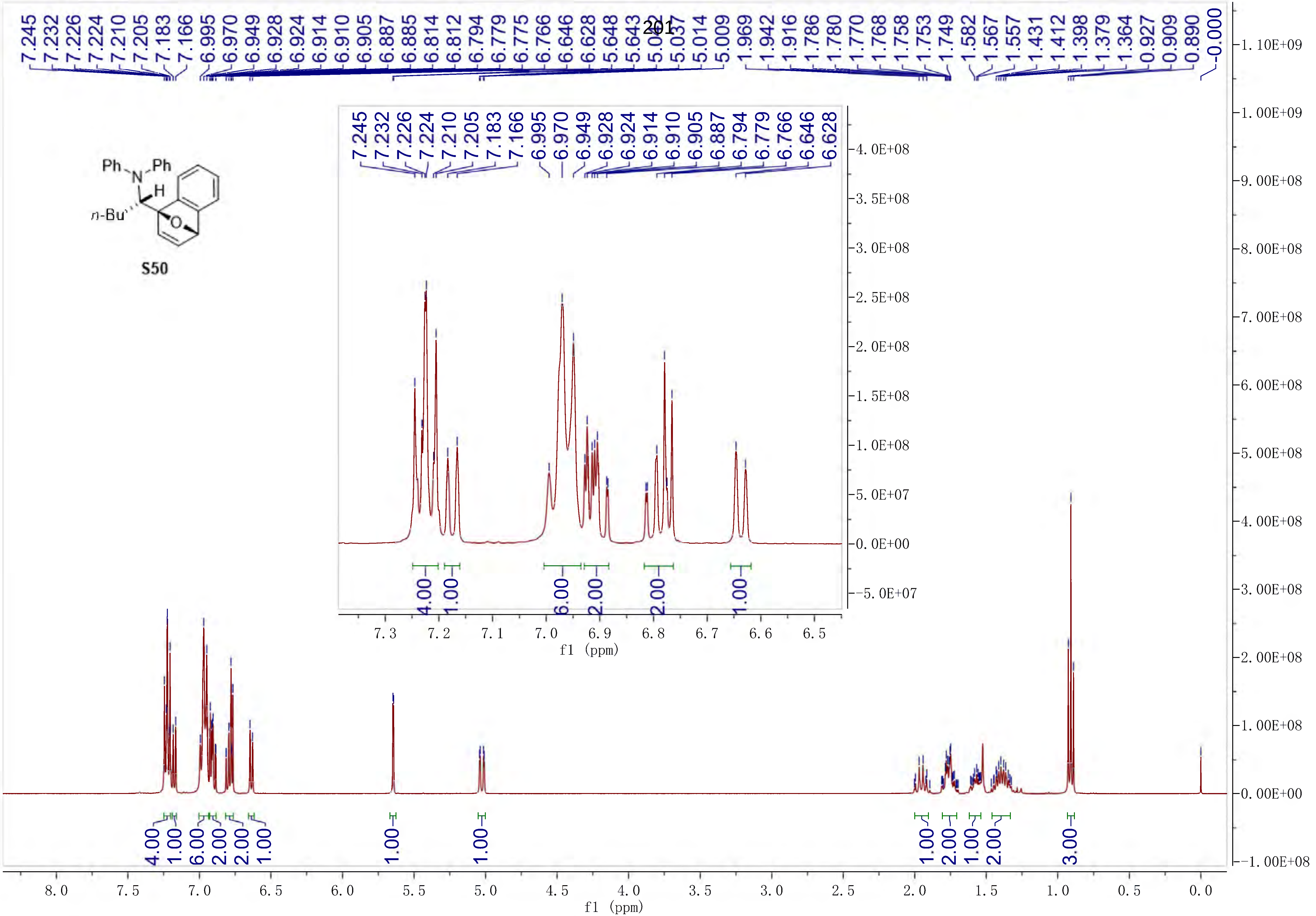

Supplementary Fig 129. ^{1}H NMR spectrum (400 MHz, $CDCl_3$, r.t.) of **S50**.

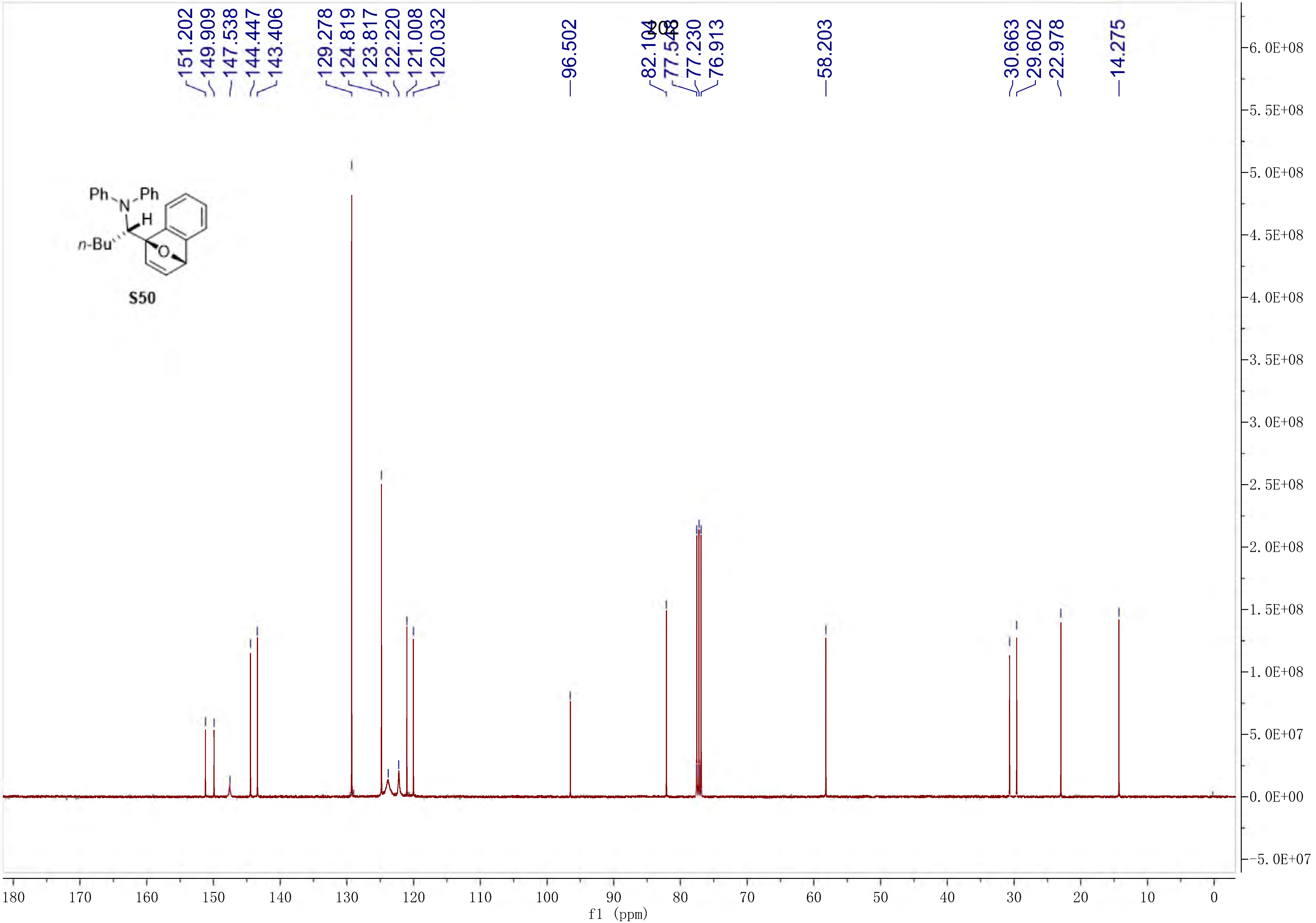

Supplementary Fig 130. ^{13}C NMR spectrum (400 MHz, $CDCl_3$, r.t.) of **S50**.

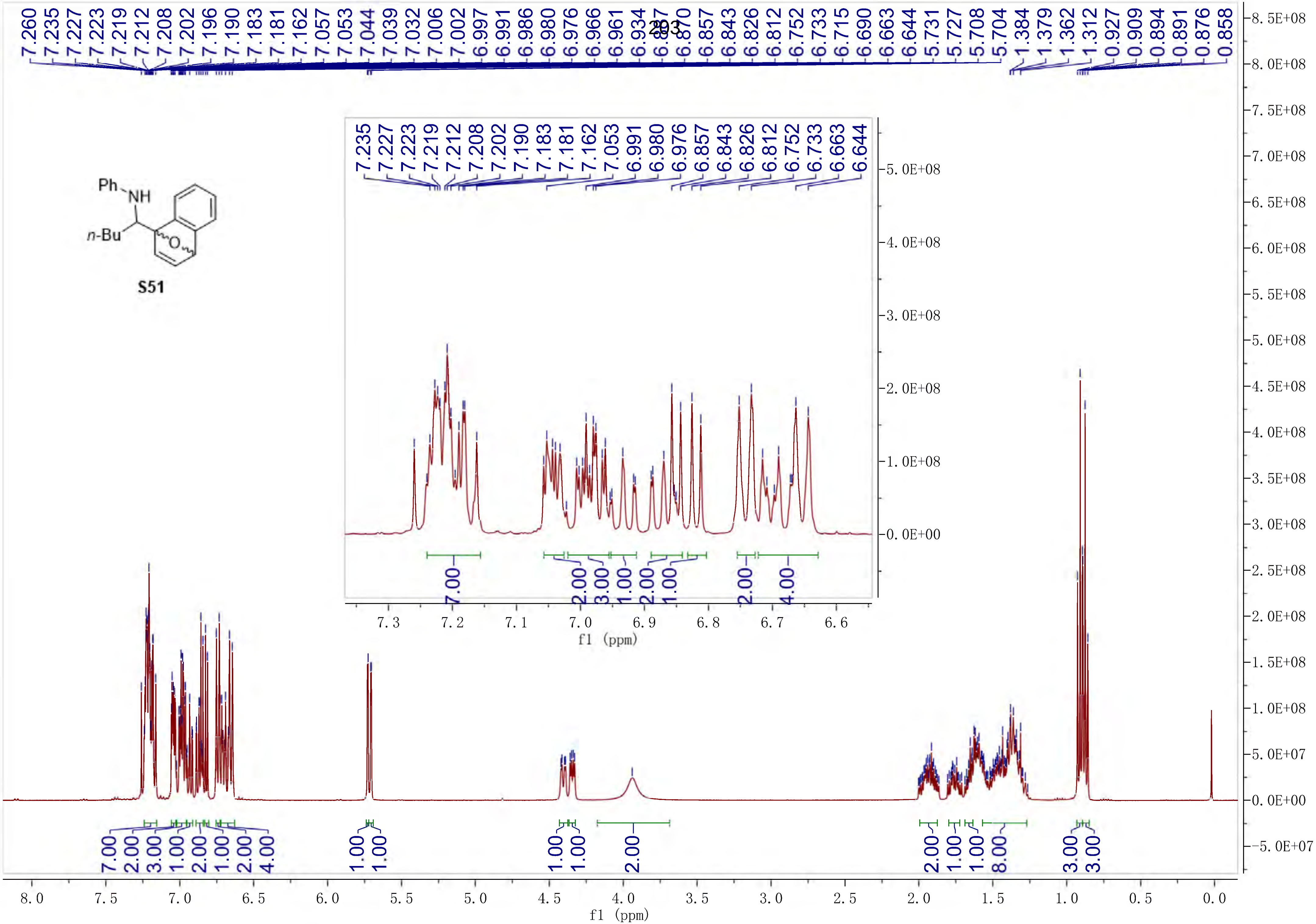

Supplementary Fig 131. ^{1}H NMR spectrum (400 MHz, $CDCl_3$, r.t.) of **S51**.

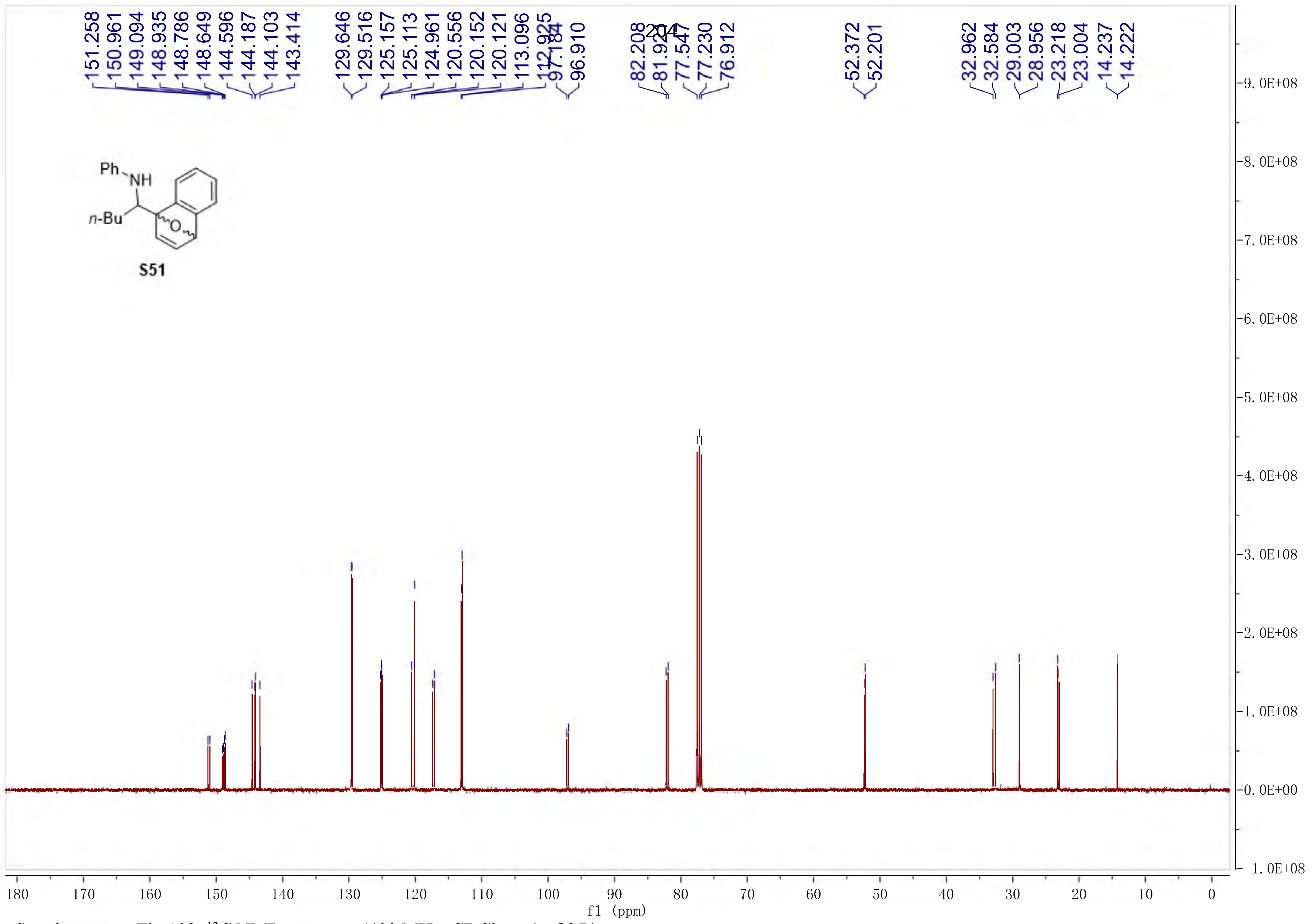

Supplementary Fig 132. ^{13}C NMR spectrum (400 MHz, $CDCl_3$, r.t.) of **S51**.

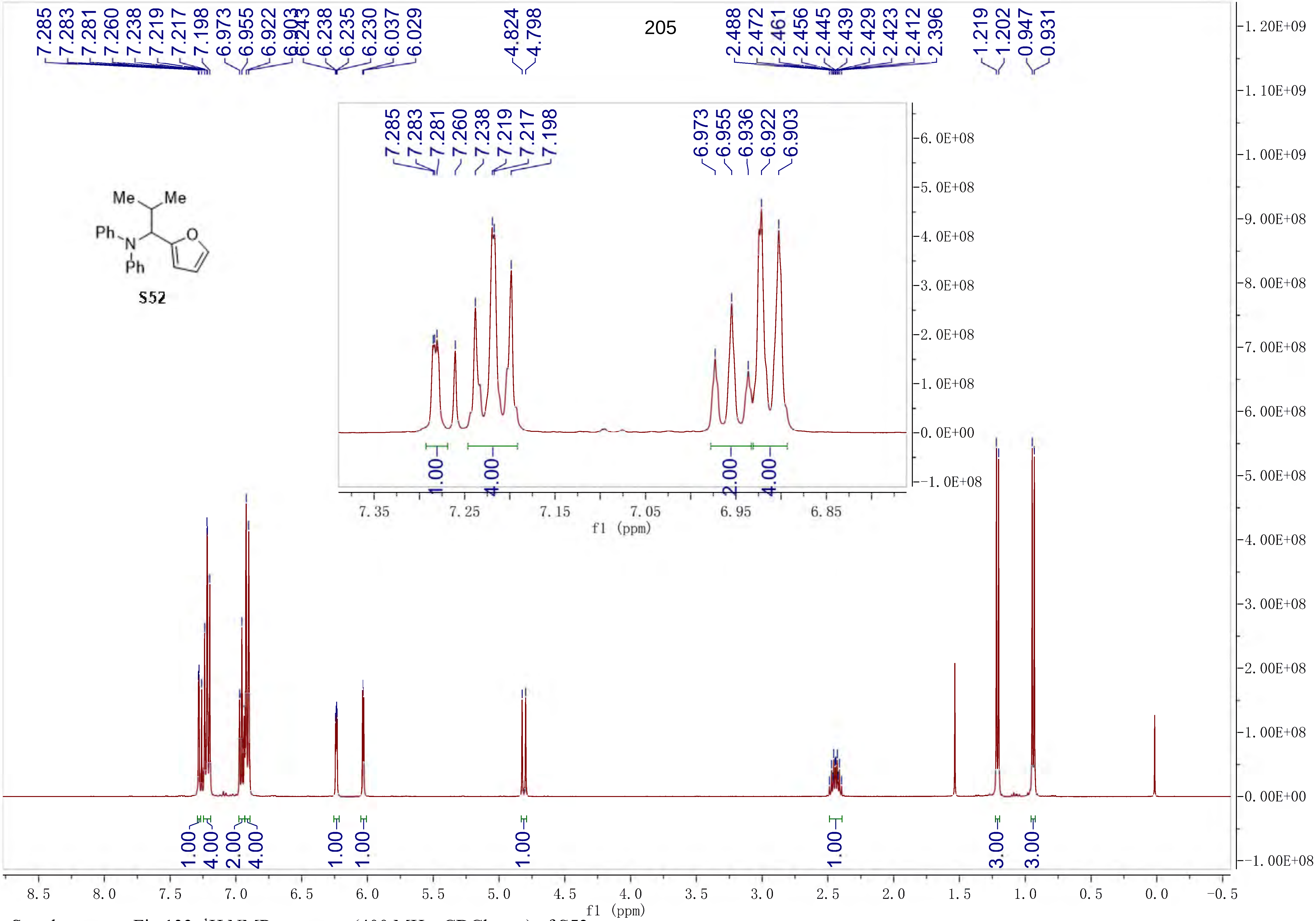

Supplementary Fig 133. ^1H NMR spectrum (400 MHz, $CDCl_3$, r.t.) of **S52**.

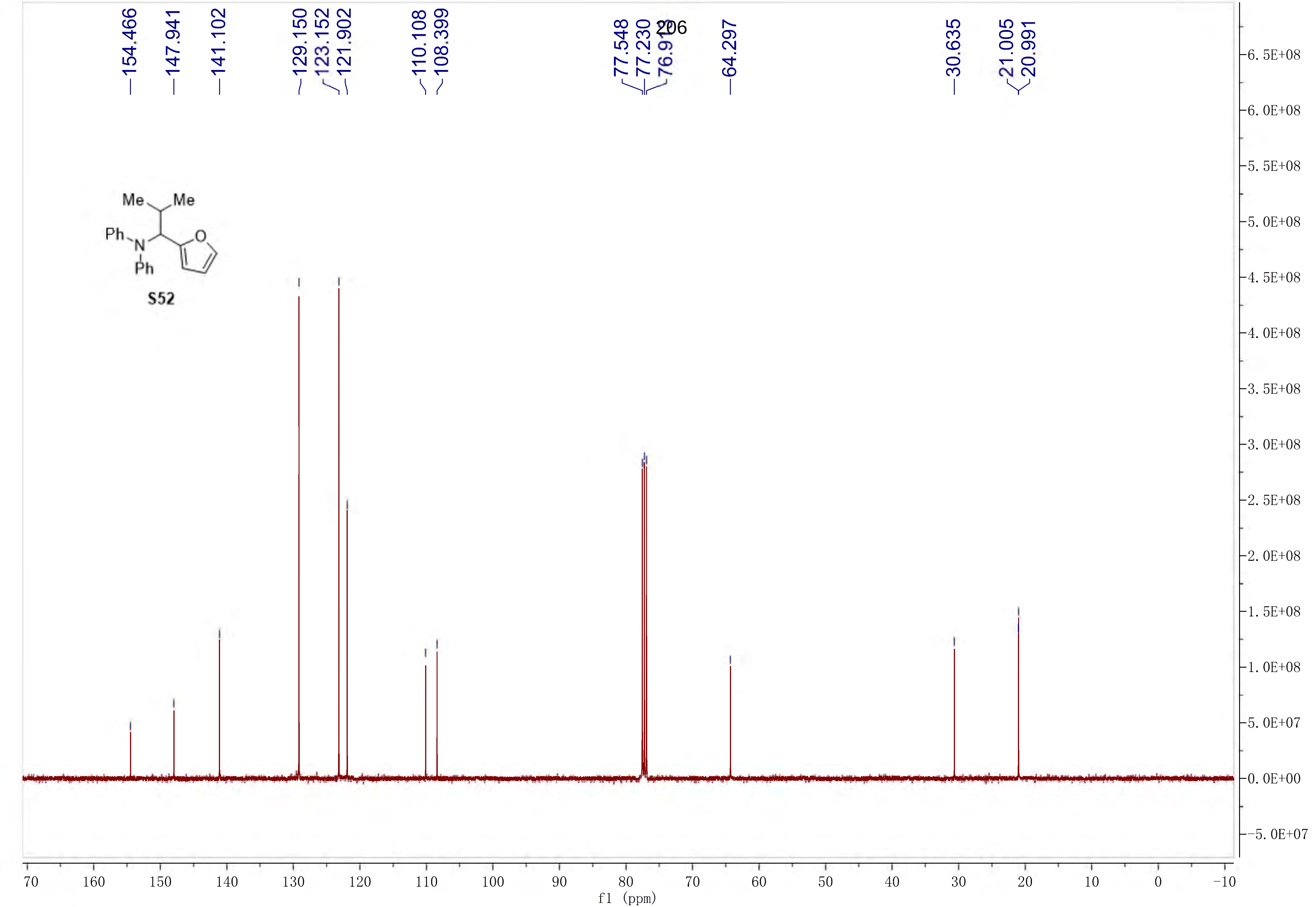

Supplementary Fig 134. ^{13}C NMR spectrum (400 MHz, $CDCl_3$, r.t.) of **S52**.

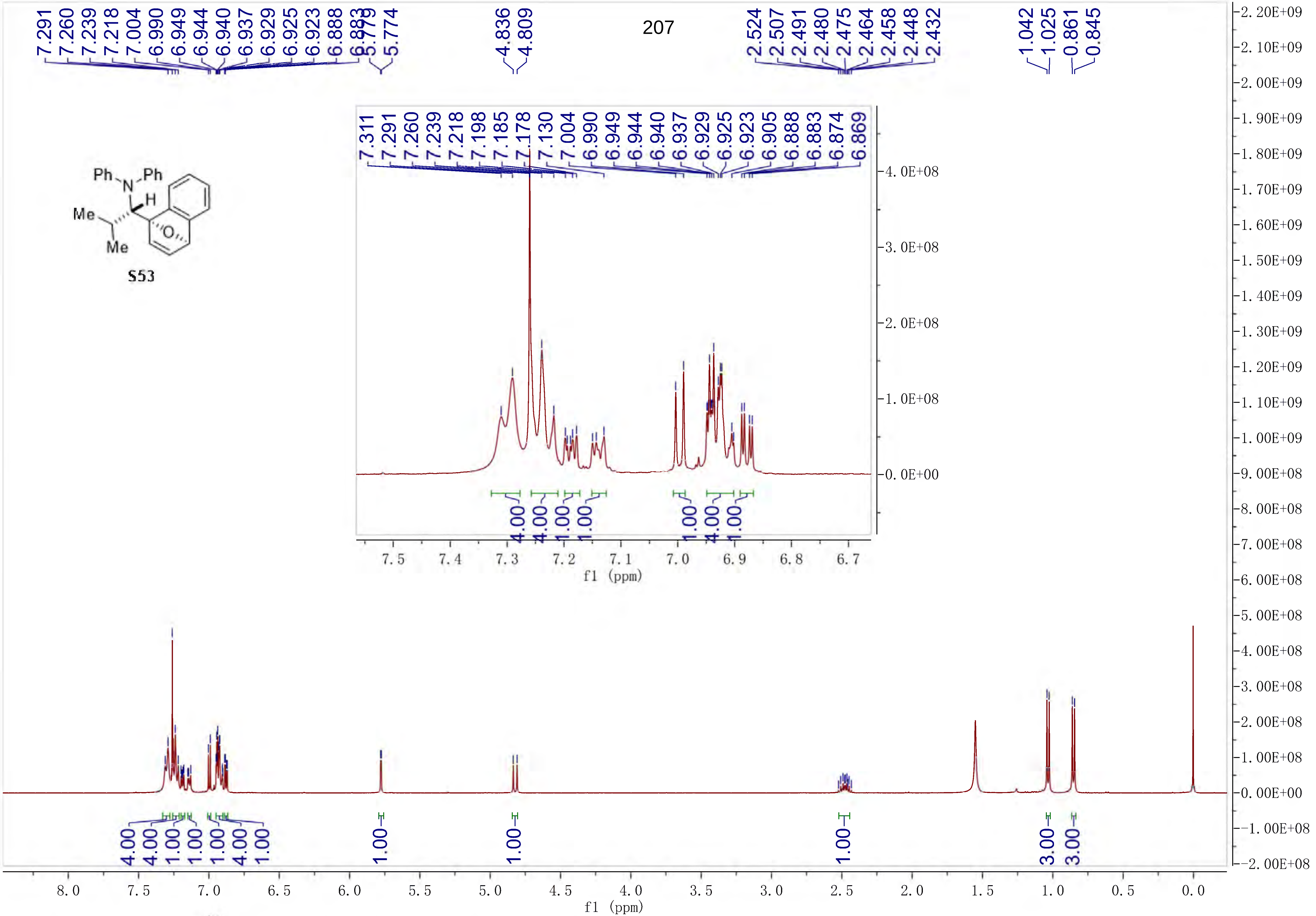

Supplementary Fig 135. ^{1}H NMR spectrum (400 MHz, $CDCl_3$, r.t.) of **S53**.

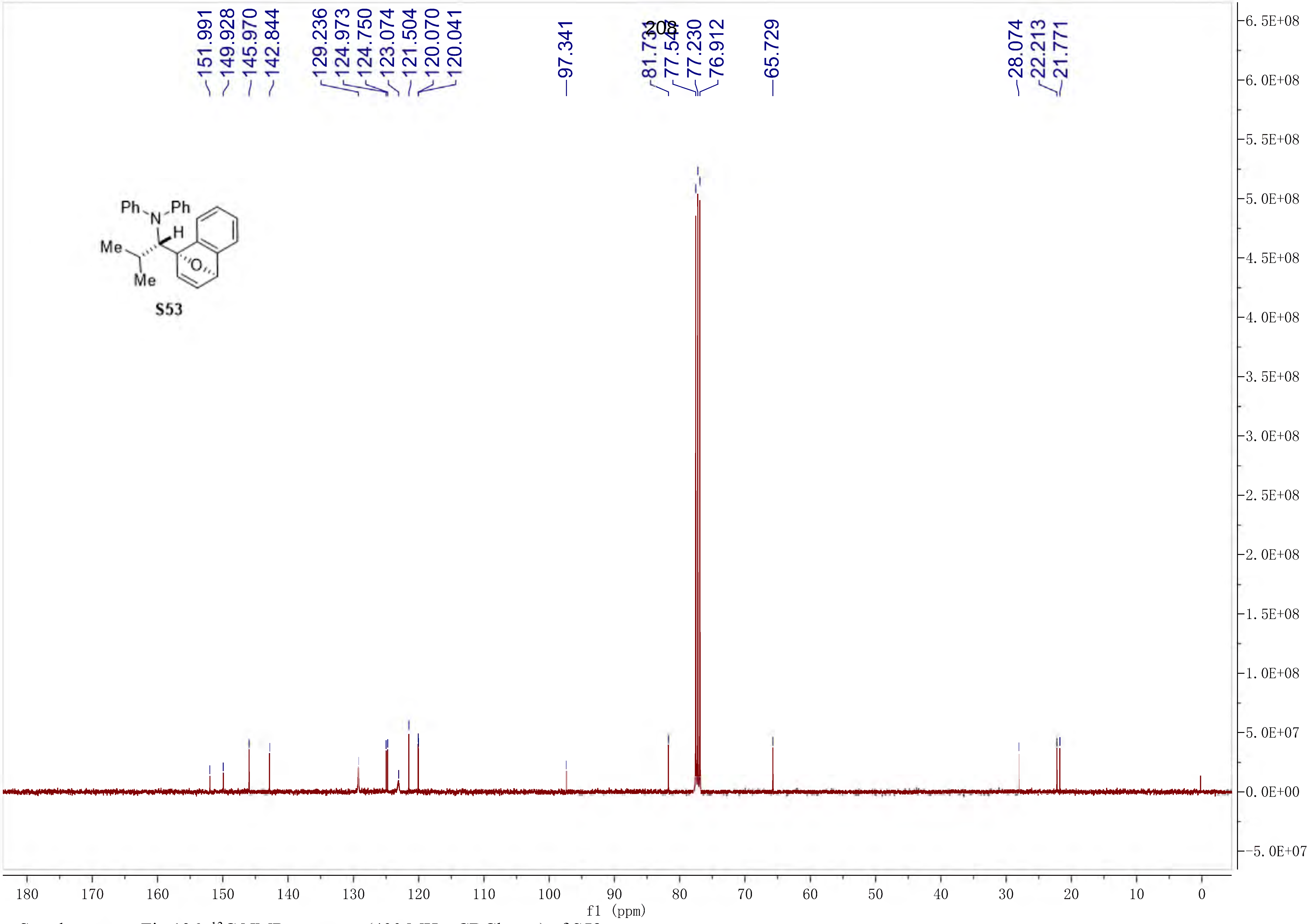

Supplementary Fig 136. ^{13}C NMR spectrum (400 MHz, $CDCl_3$, r.t.) of **S53**.

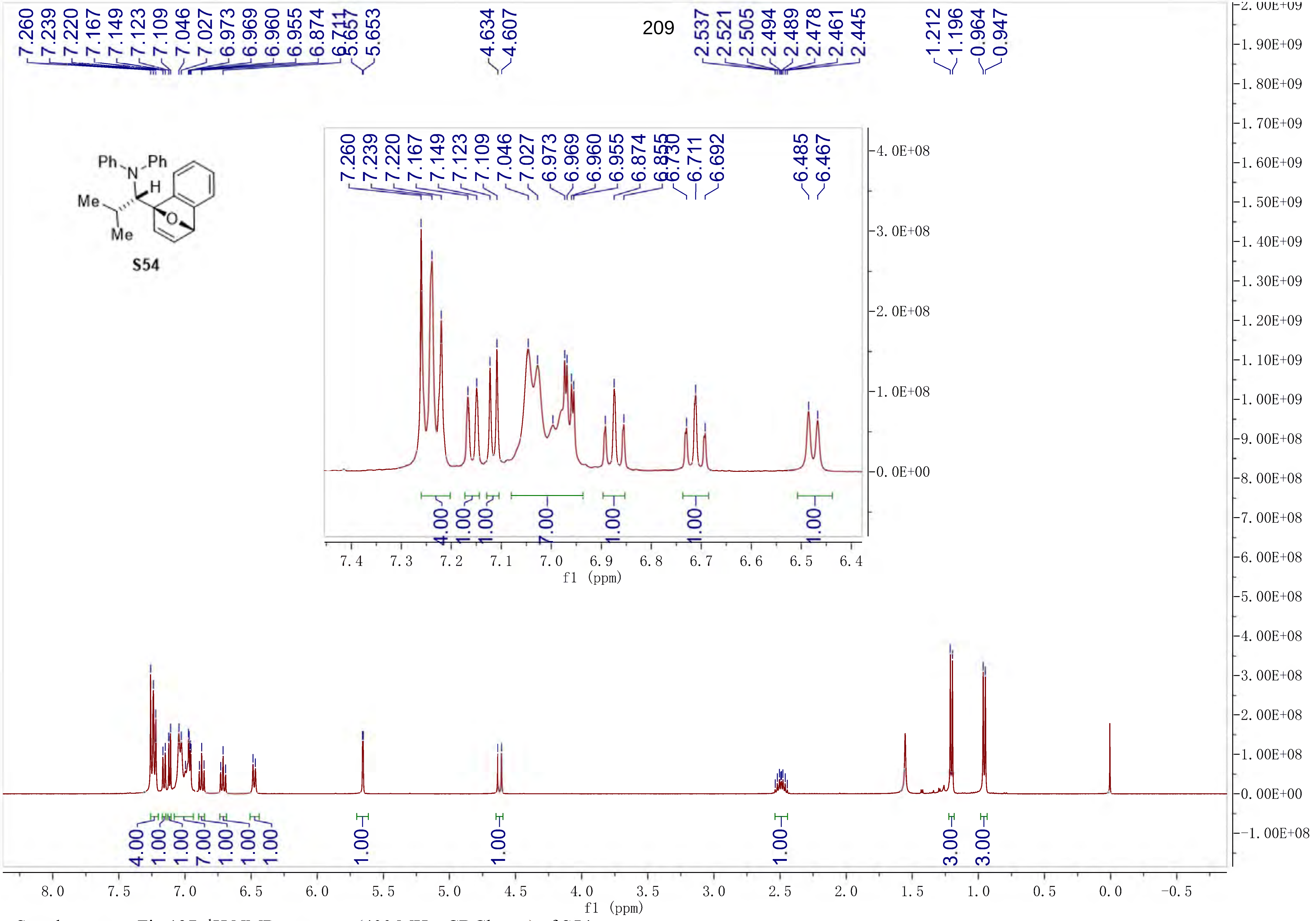

Supplementary Fig 137. ^{1}H NMR spectrum (400 MHz, $CDCl_3$, r.t.) of **S54**.

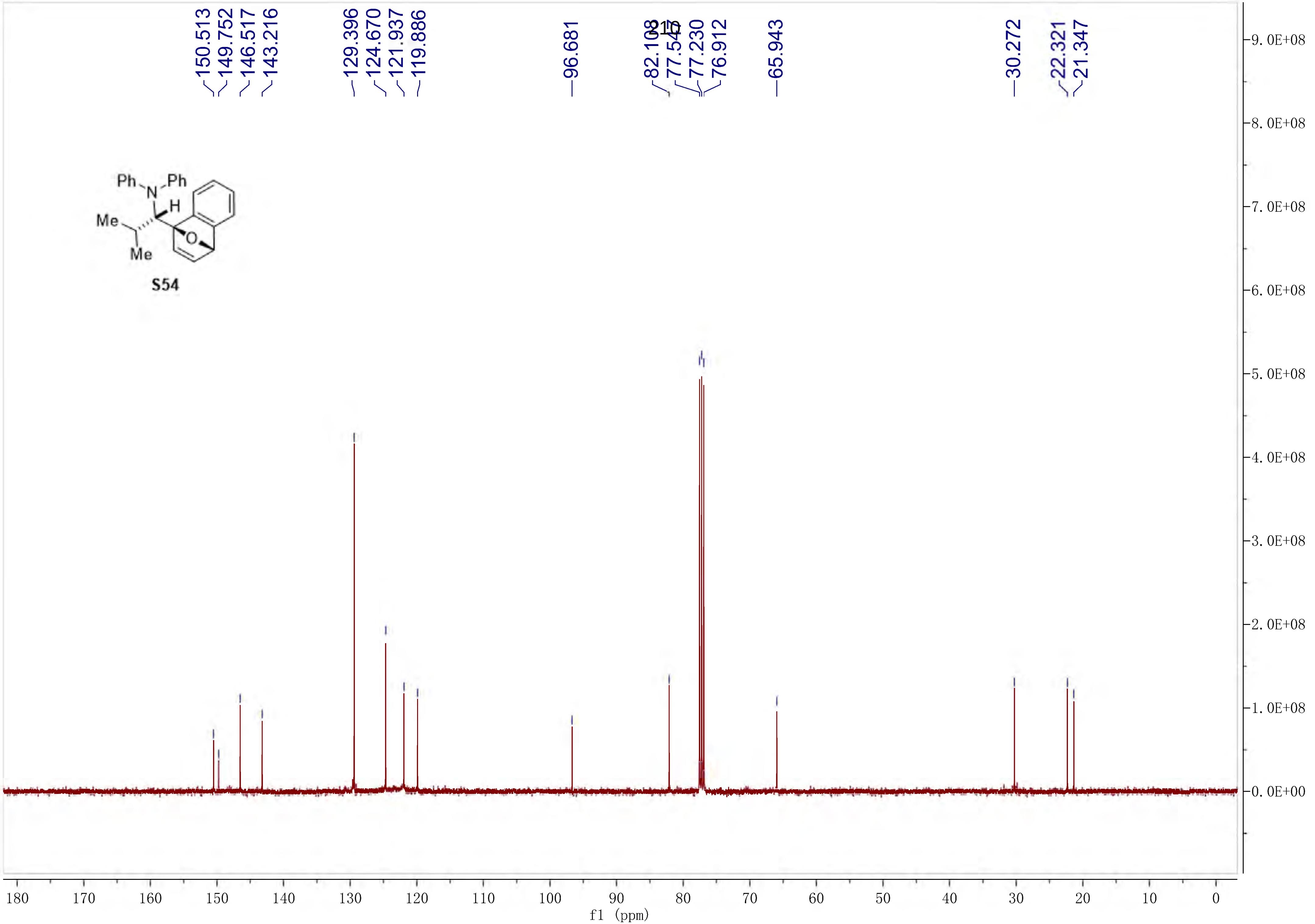

Supplementary Fig 138. ^{13}C NMR spectrum (400 MHz, $CDCl_3$, r.t.) of **S54**.

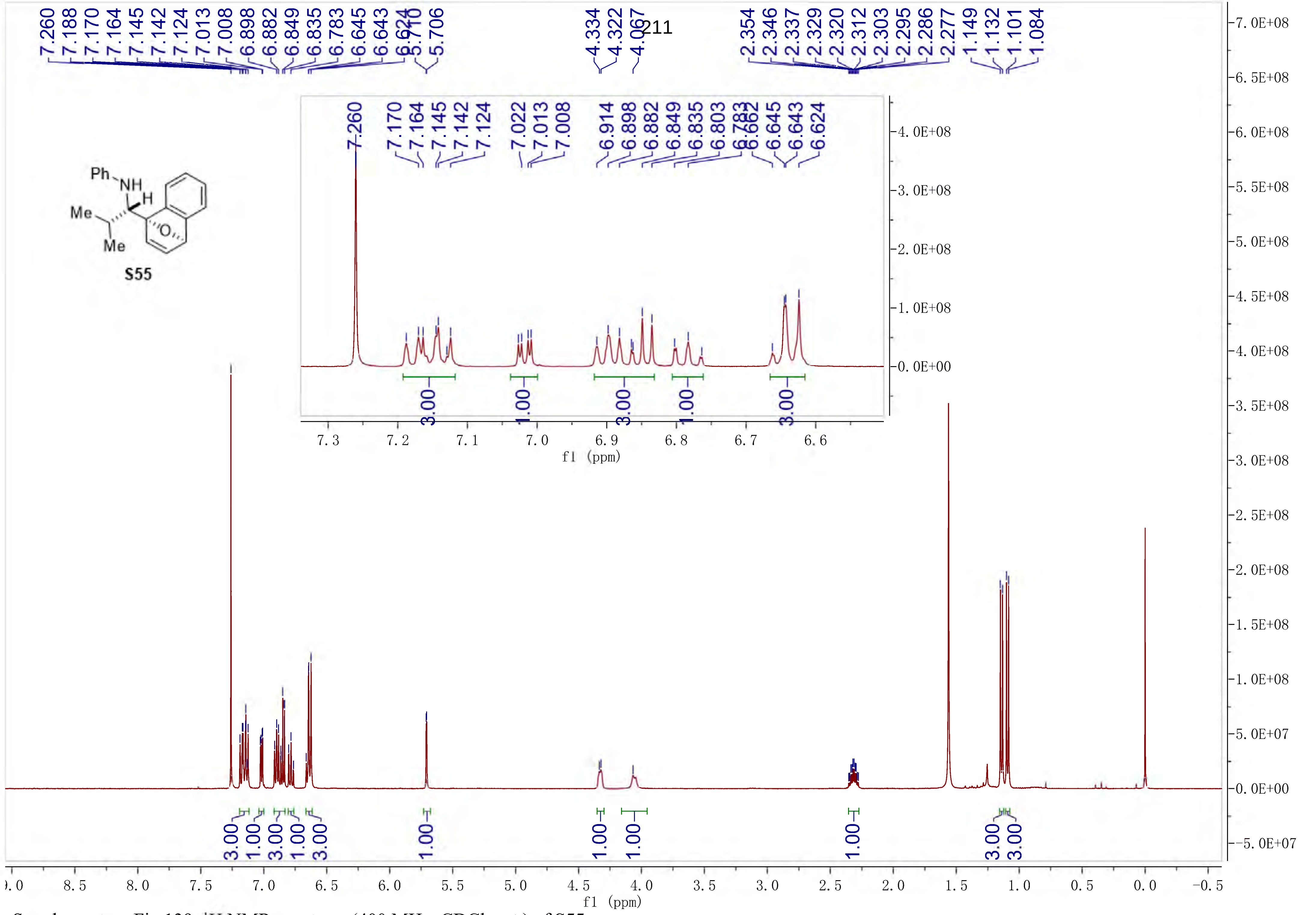

Supplementary Fig 139. ^{1}H NMR spectrum (400 MHz, $CDCl_3$, r.t.) of **S55**.

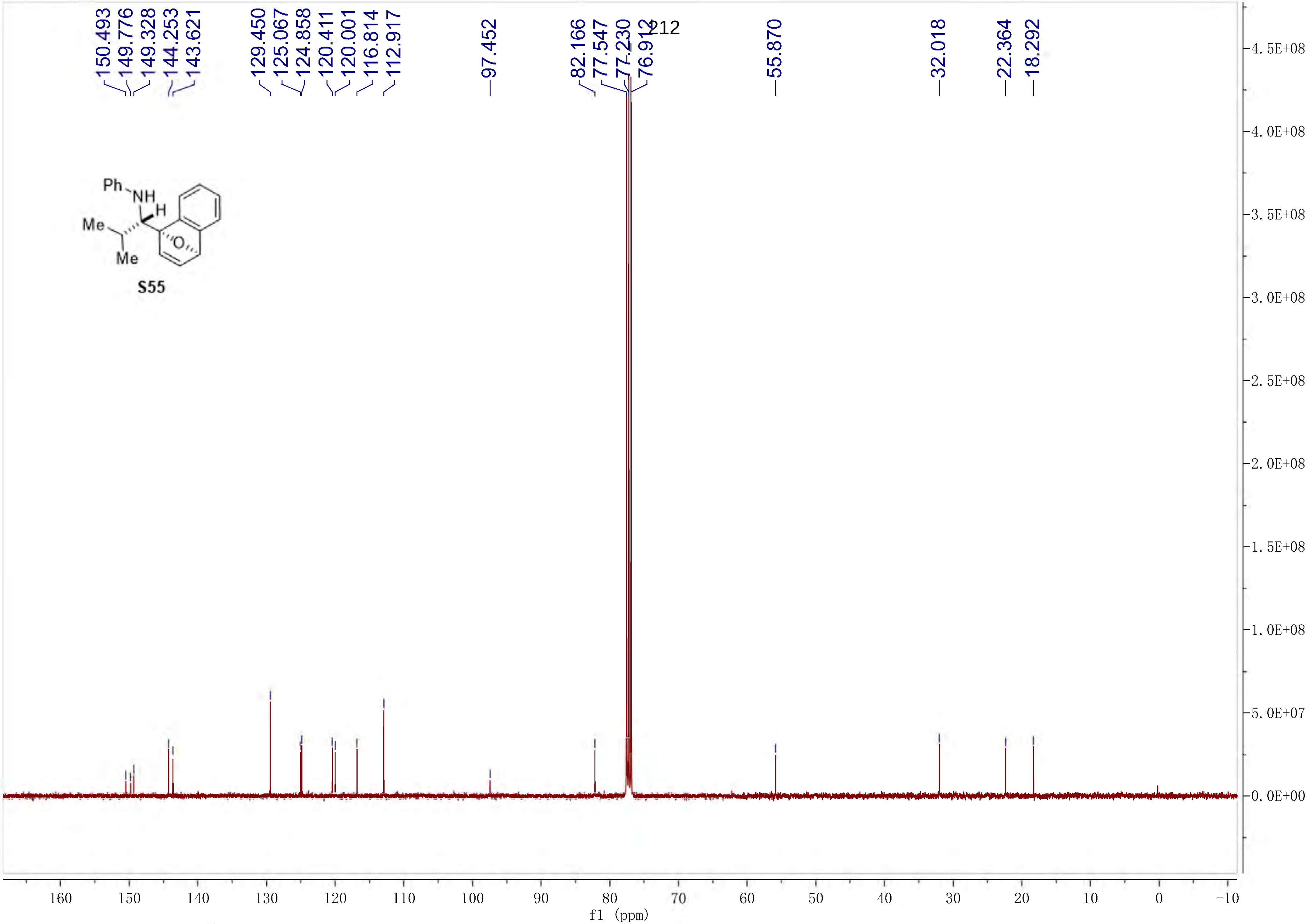

Supplementary Fig 140. ^{13}C NMR spectrum (400 MHz, $CDCl_3$, r.t.) of **S55**.

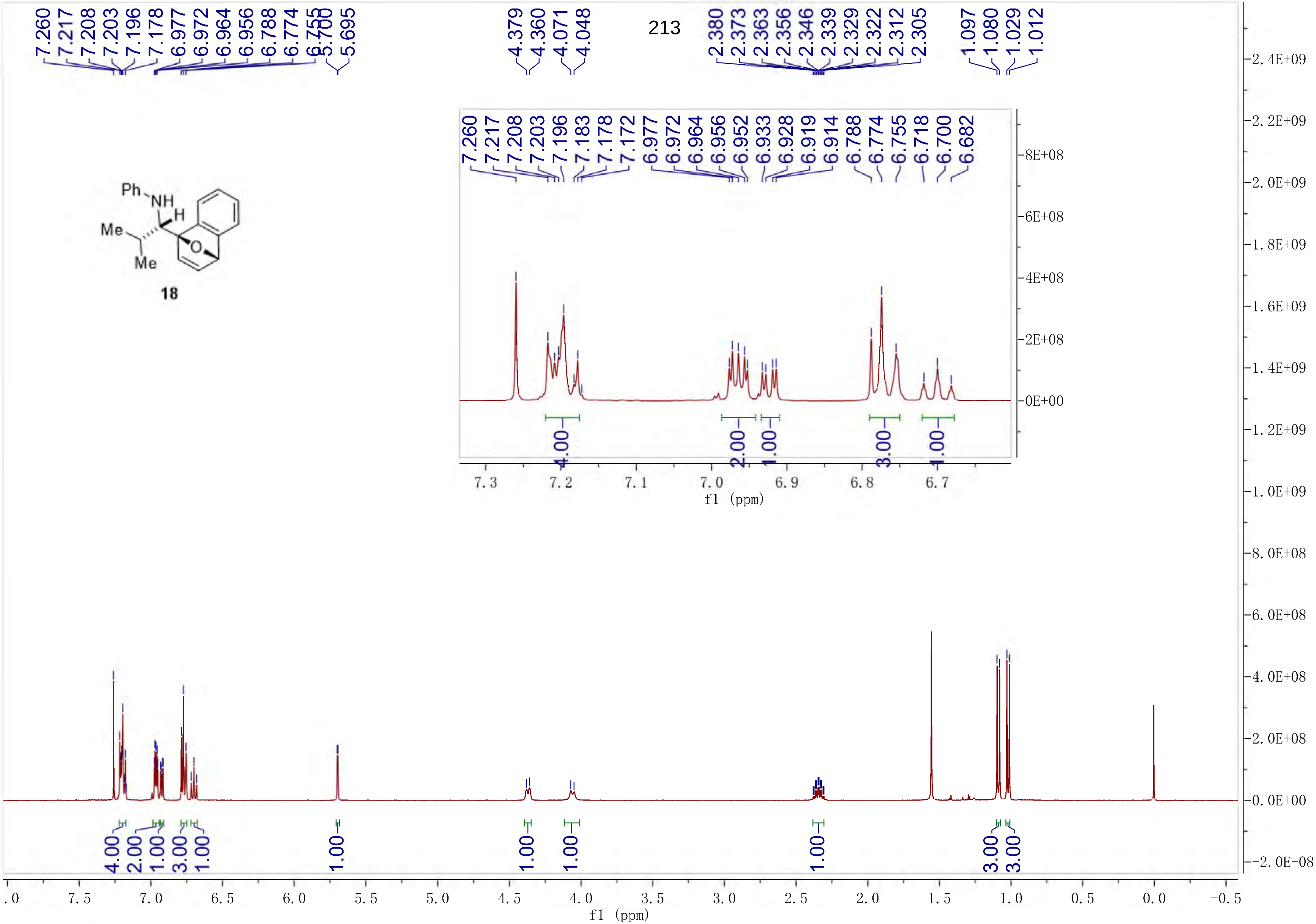

Supplementary Fig 141. ^{1}H NMR spectrum (400 MHz, $CDCl_3$, r.t.) of **S18**.

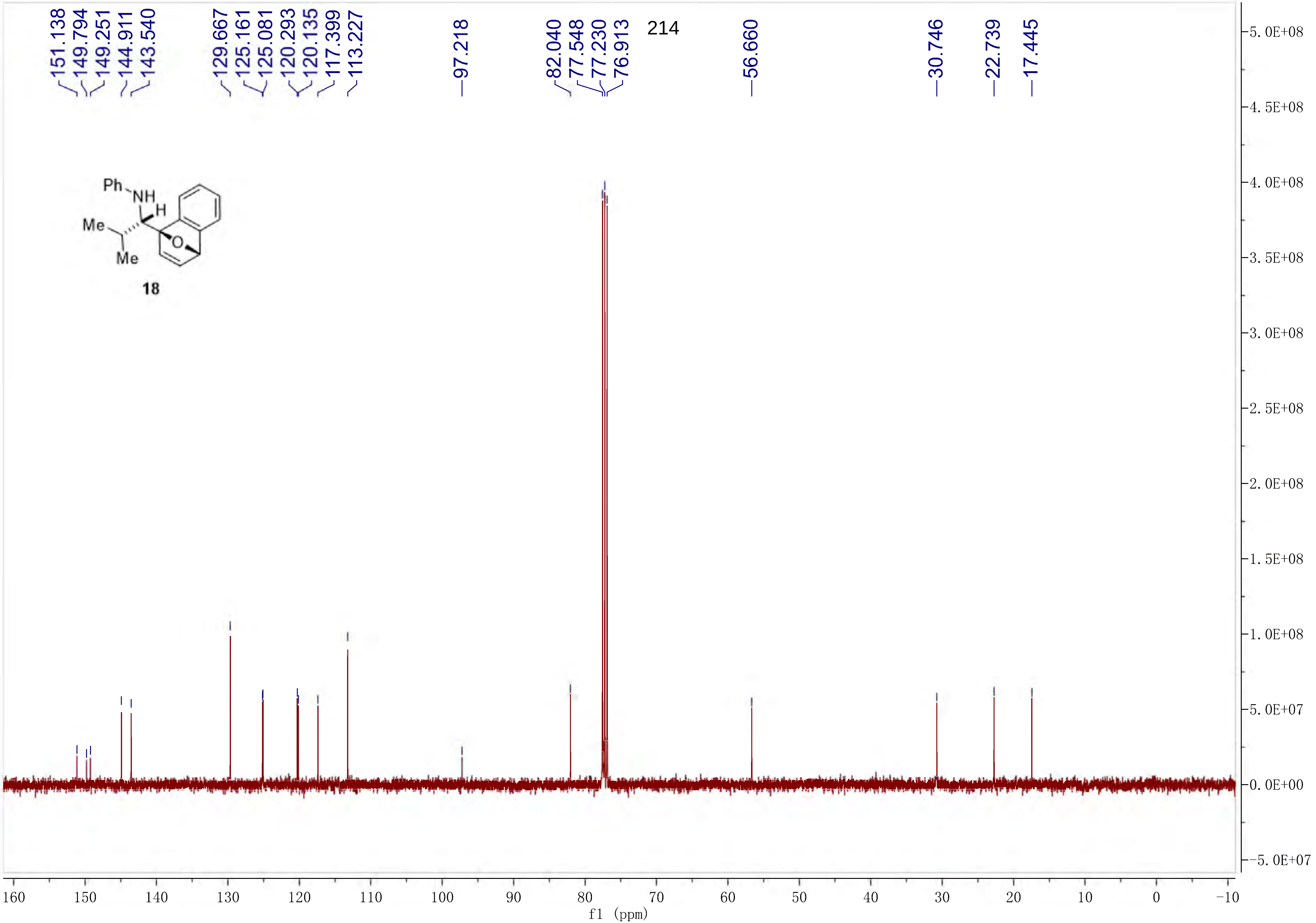

Supplementary Fig 142. ^{13}C NMR spectrum (400 MHz, $CDCl_3$, r.t.) of **S18**.

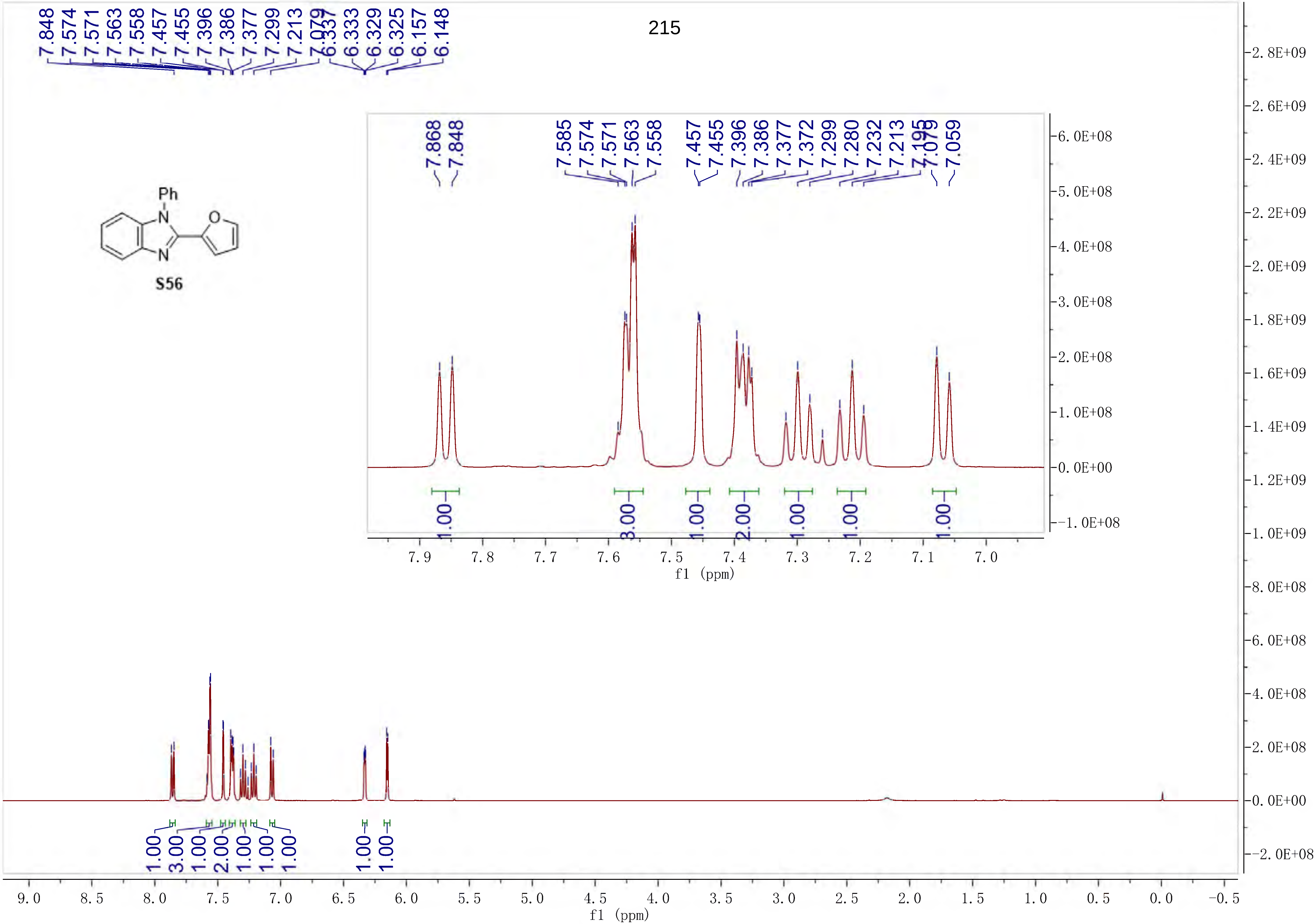

Supplementary Fig 143. ^{1}H NMR spectrum (400 MHz, $CDCl_3$, r.t.) of **S56**.

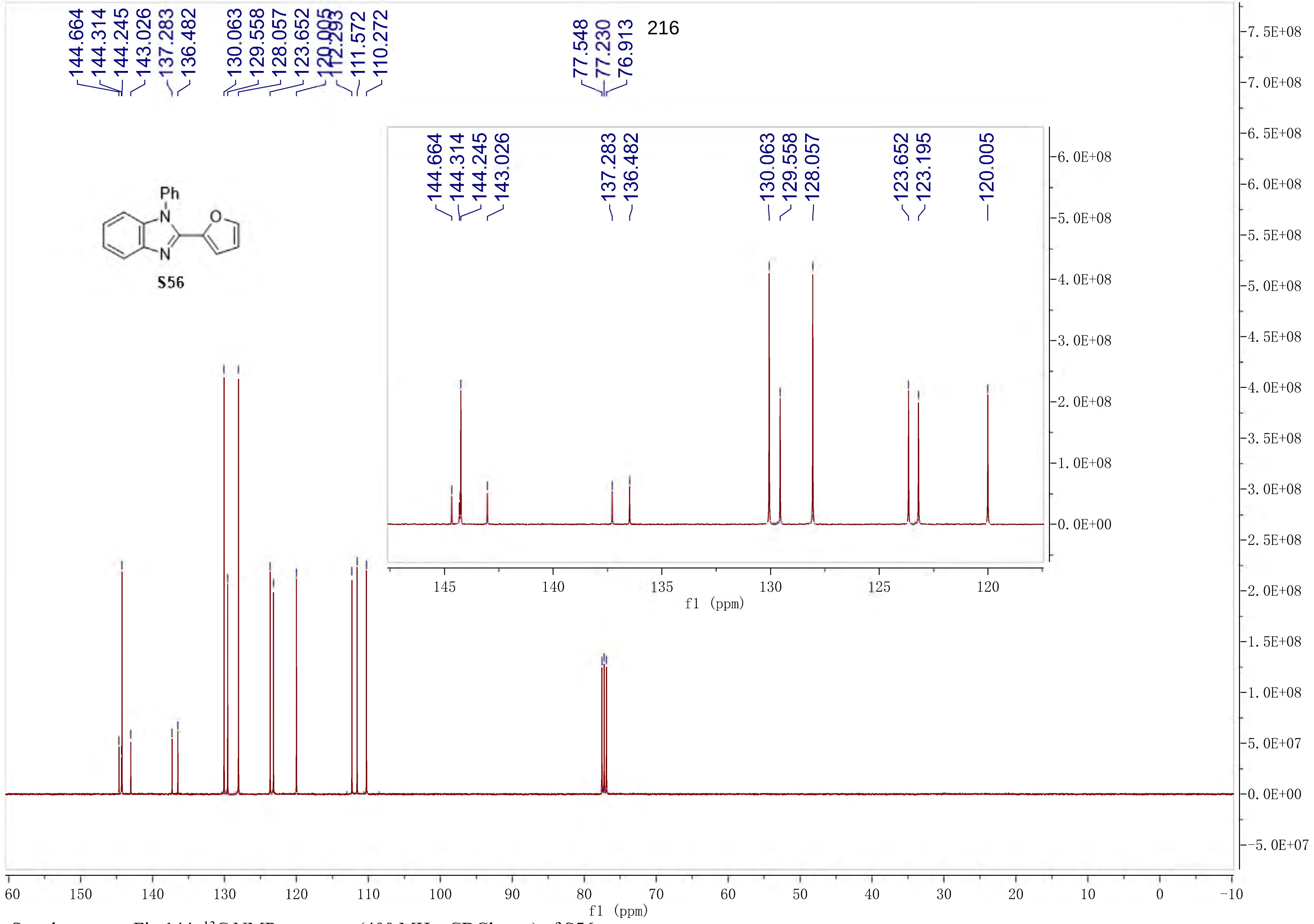

Supplementary Fig 144. ^{13}C NMR spectrum (400 MHz, $CDCl_3$, r.t.) of **S56**.

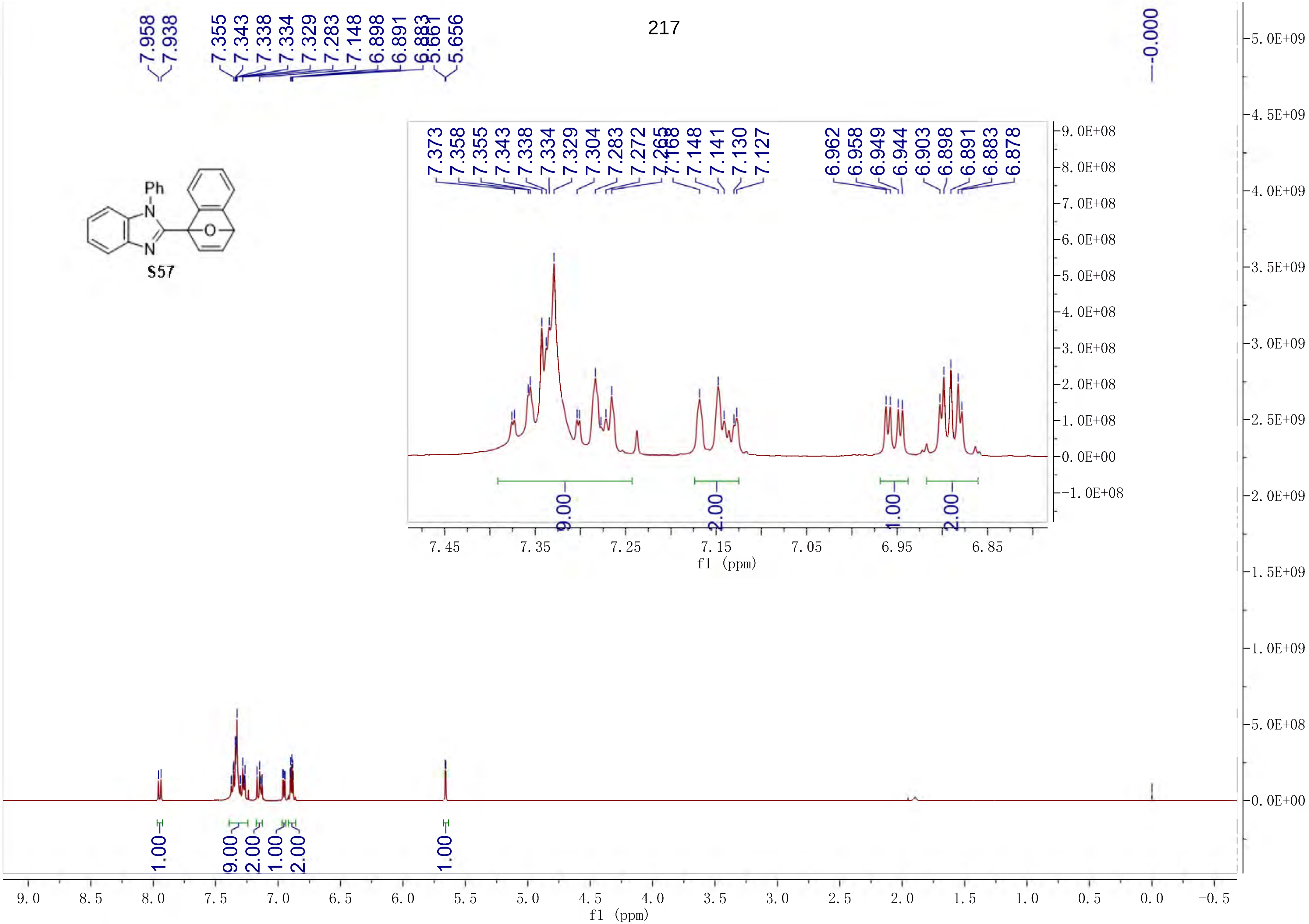

Supplementary Fig 145. ^{1}H NMR spectrum (400 MHz, $CDCl_3$, r.t.) of **S57**.

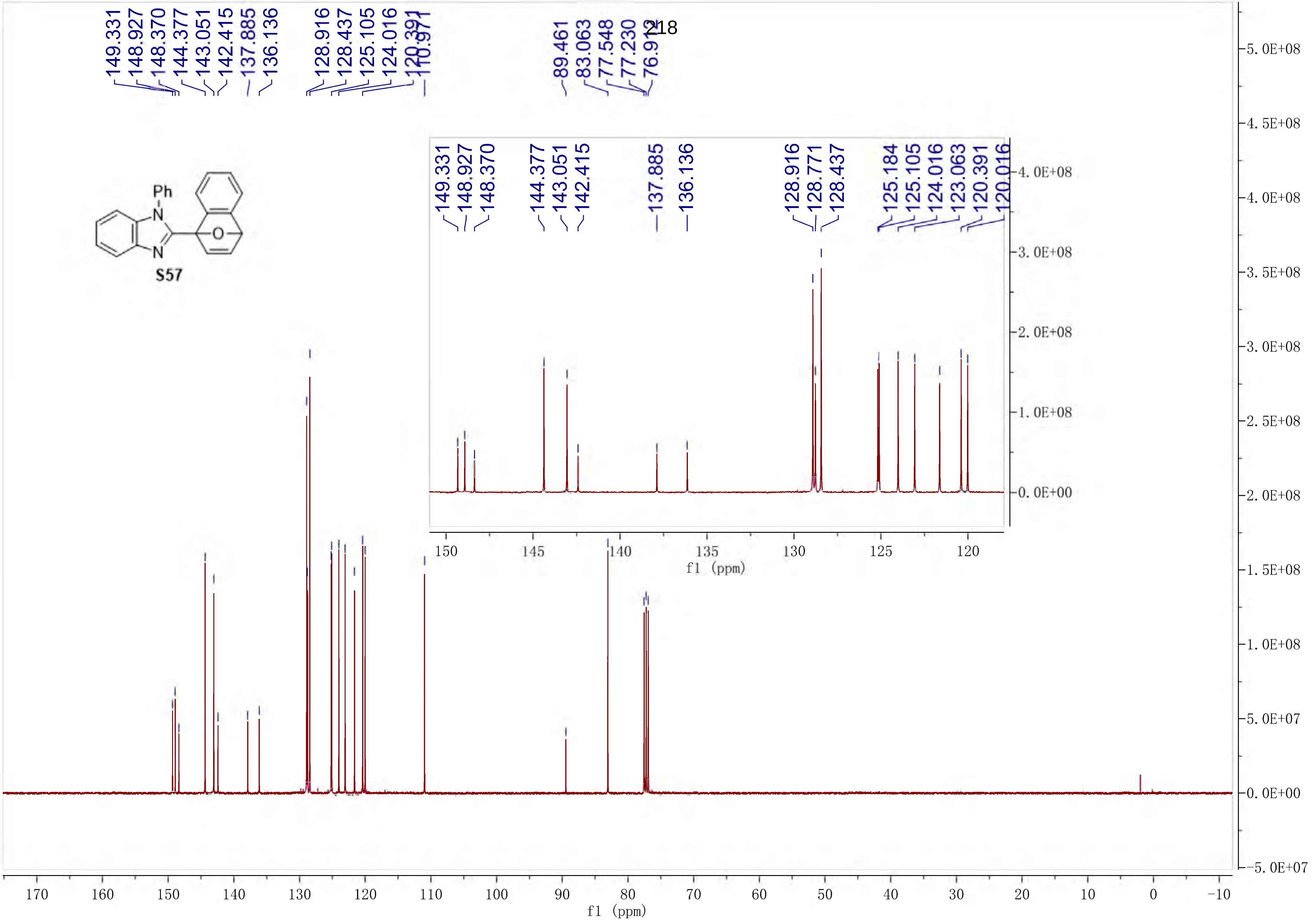

Supplementary Fig 146. ^{13}C NMR spectrum (400 MHz, $CDCl_3$, r.t.) of **S57**.

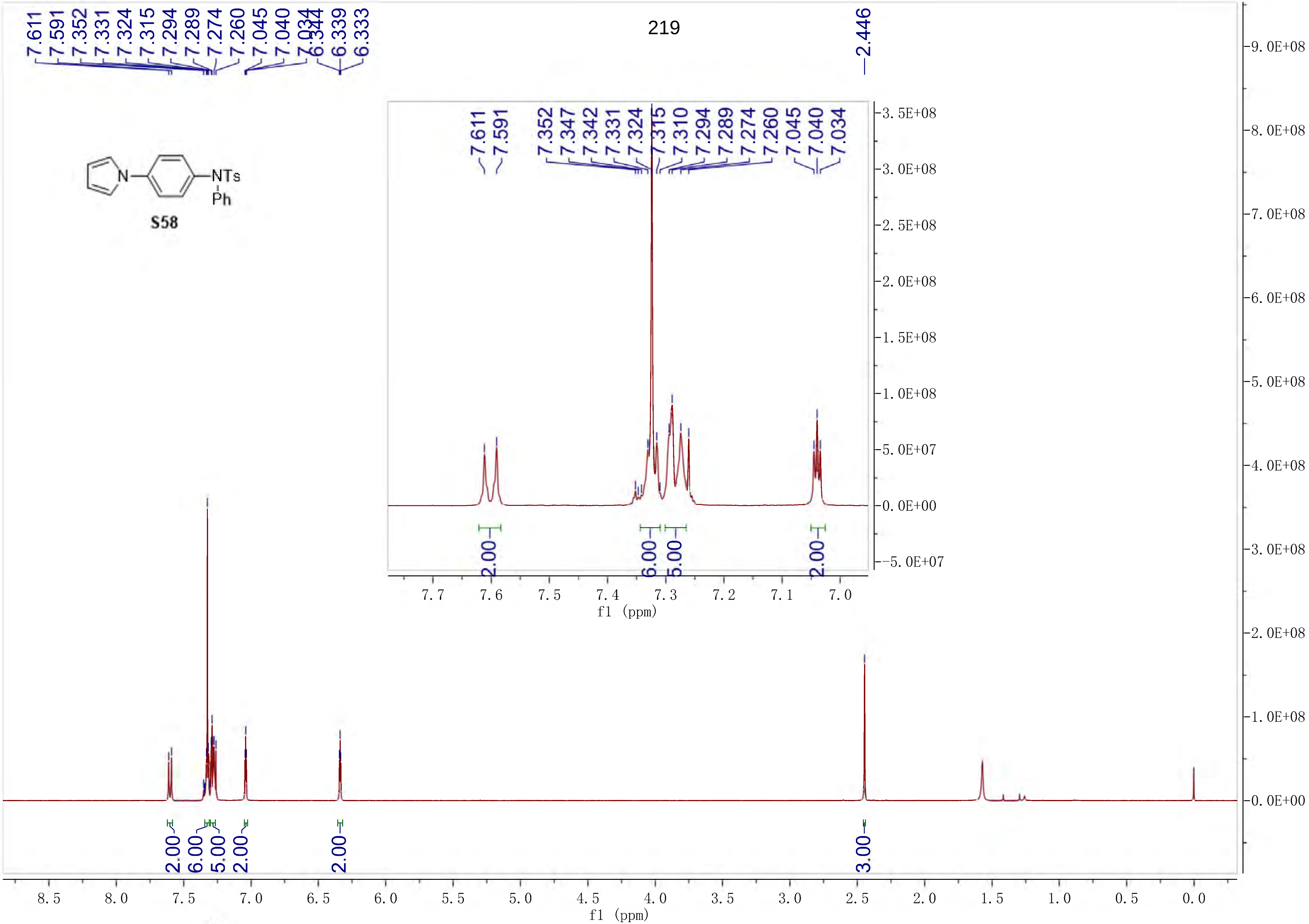

Supplementary Fig 147. ^{1}H NMR spectrum (400 MHz, $CDCl_3$, r.t.) of **S58**.

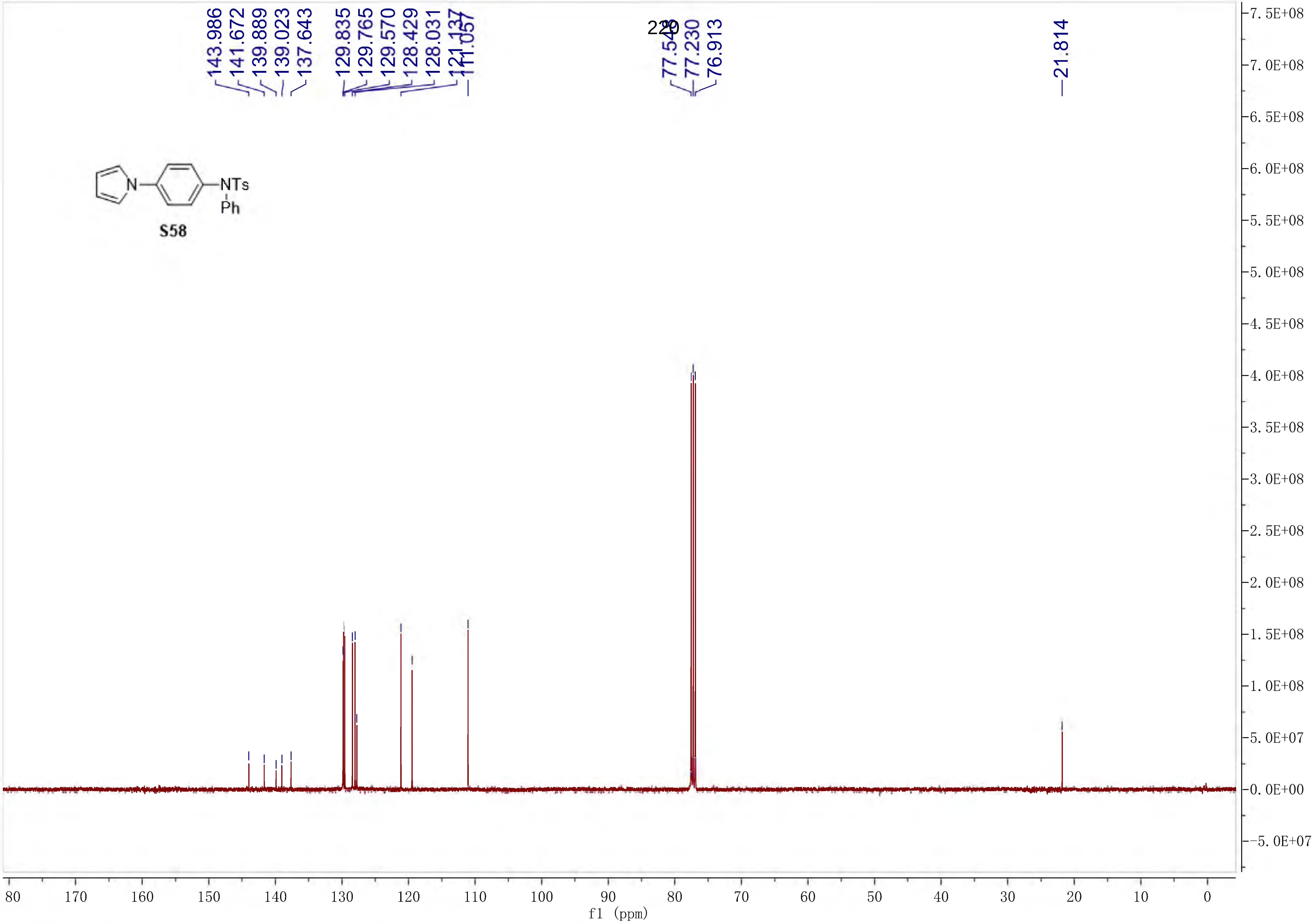

Supplementary Fig 148. ^{13}C NMR spectrum (400 MHz, $CDCl_3$, r.t.) of **S58**.

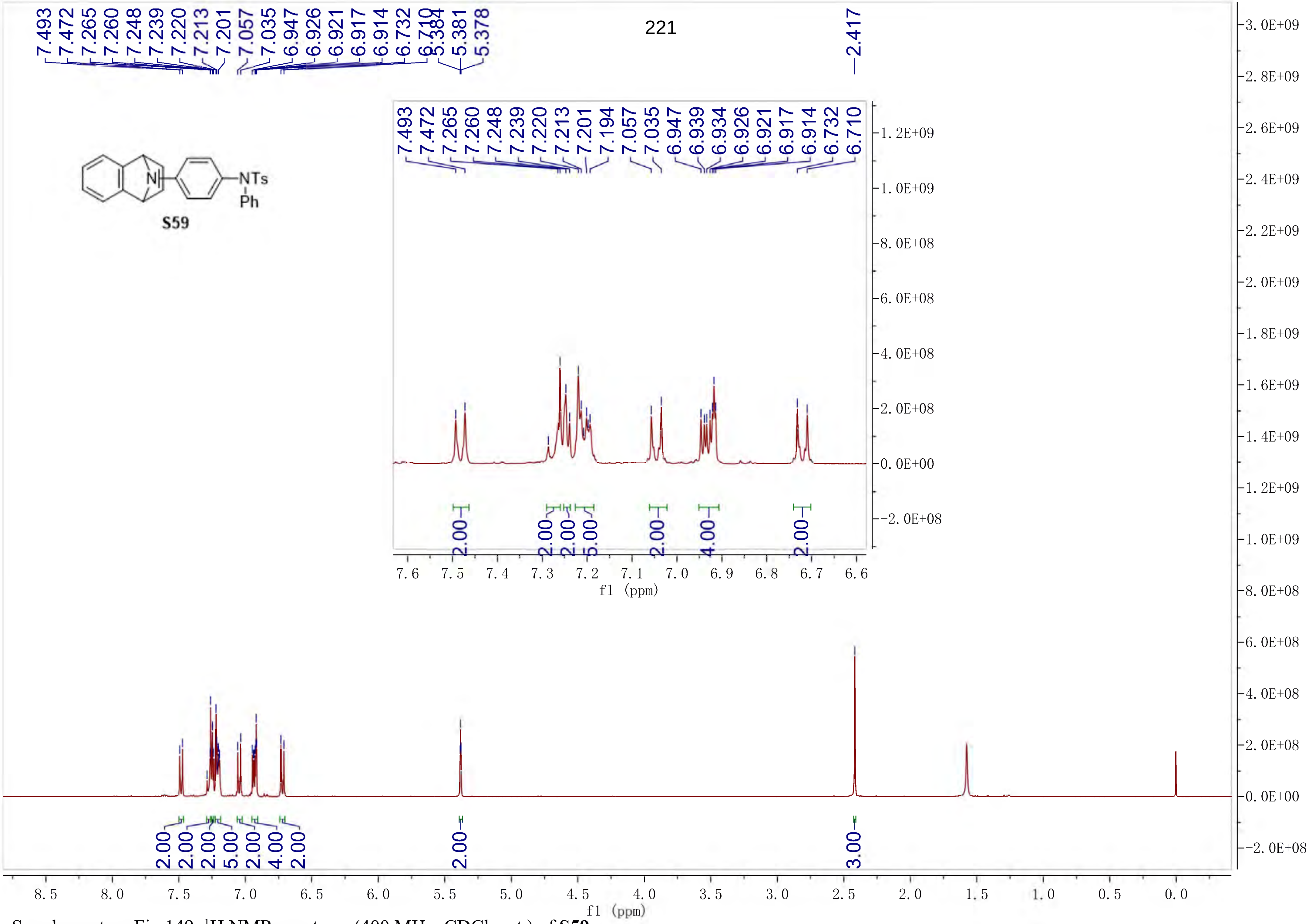

Supplementary Fig 149. ^{1}H NMR spectrum (400 MHz, $CDCl_3$, r.t.) of **S59**.

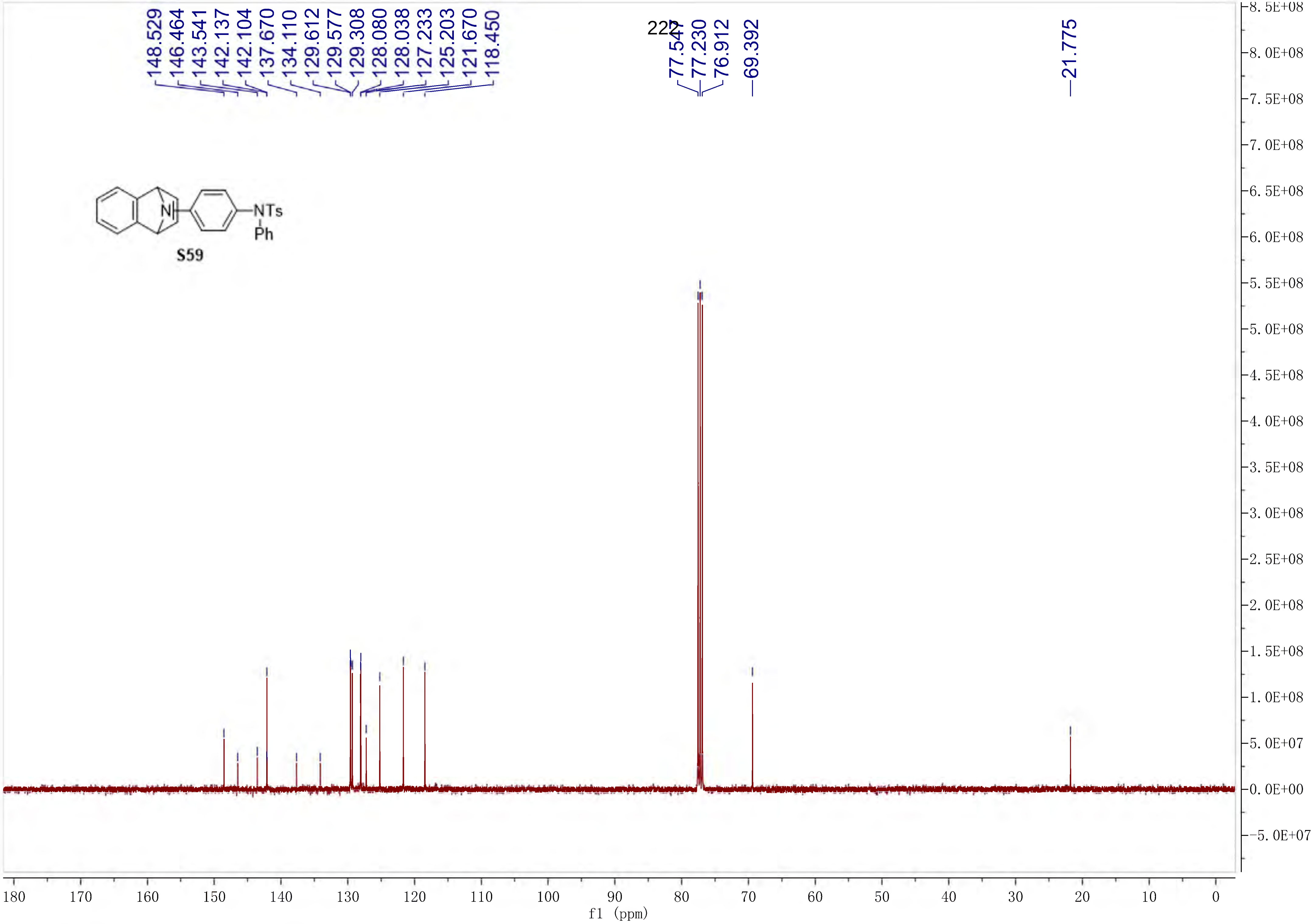

Supplementary Fig 150. ^{13}C NMR spectrum (400 MHz, $CDCl_3$, r.t.) of **S59**.

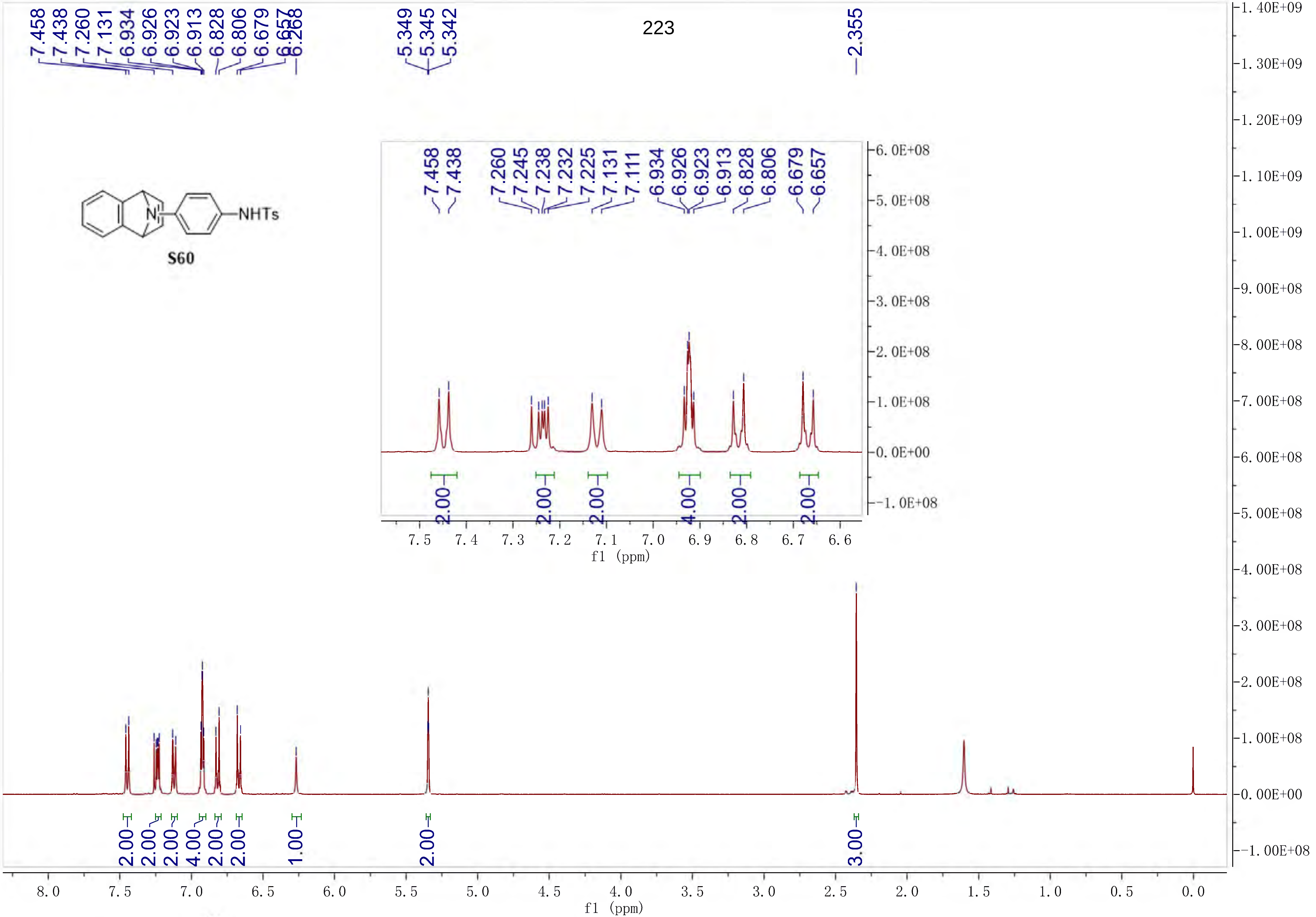

Supplementary Fig 151. ^{1}H NMR spectrum (400 MHz, $CDCl_3$, r.t.) of **S60**.

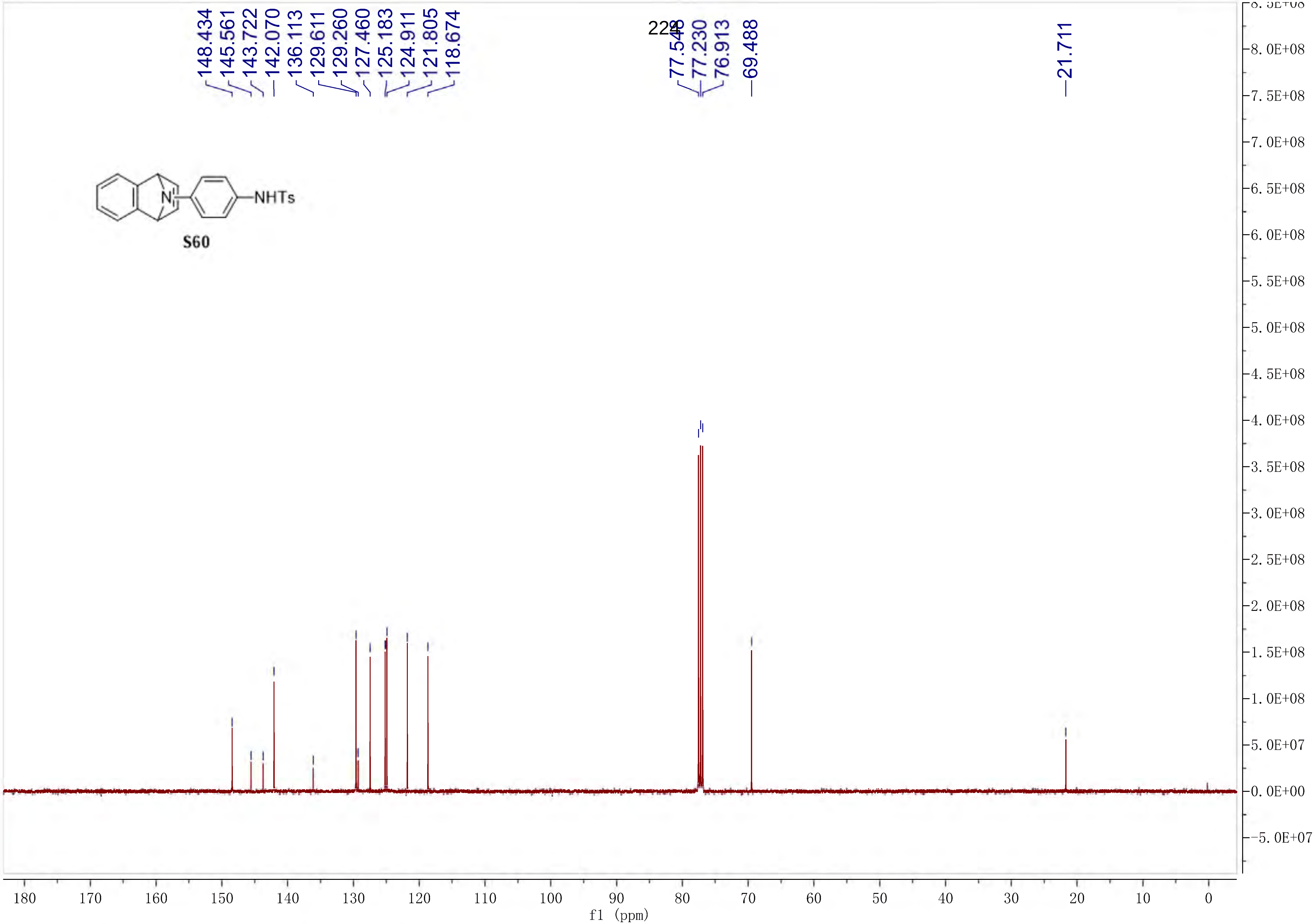

Supplementary Fig 152. ^{13}C NMR spectrum (400 MHz, $CDCl_3$, r.t.) of **S60**.

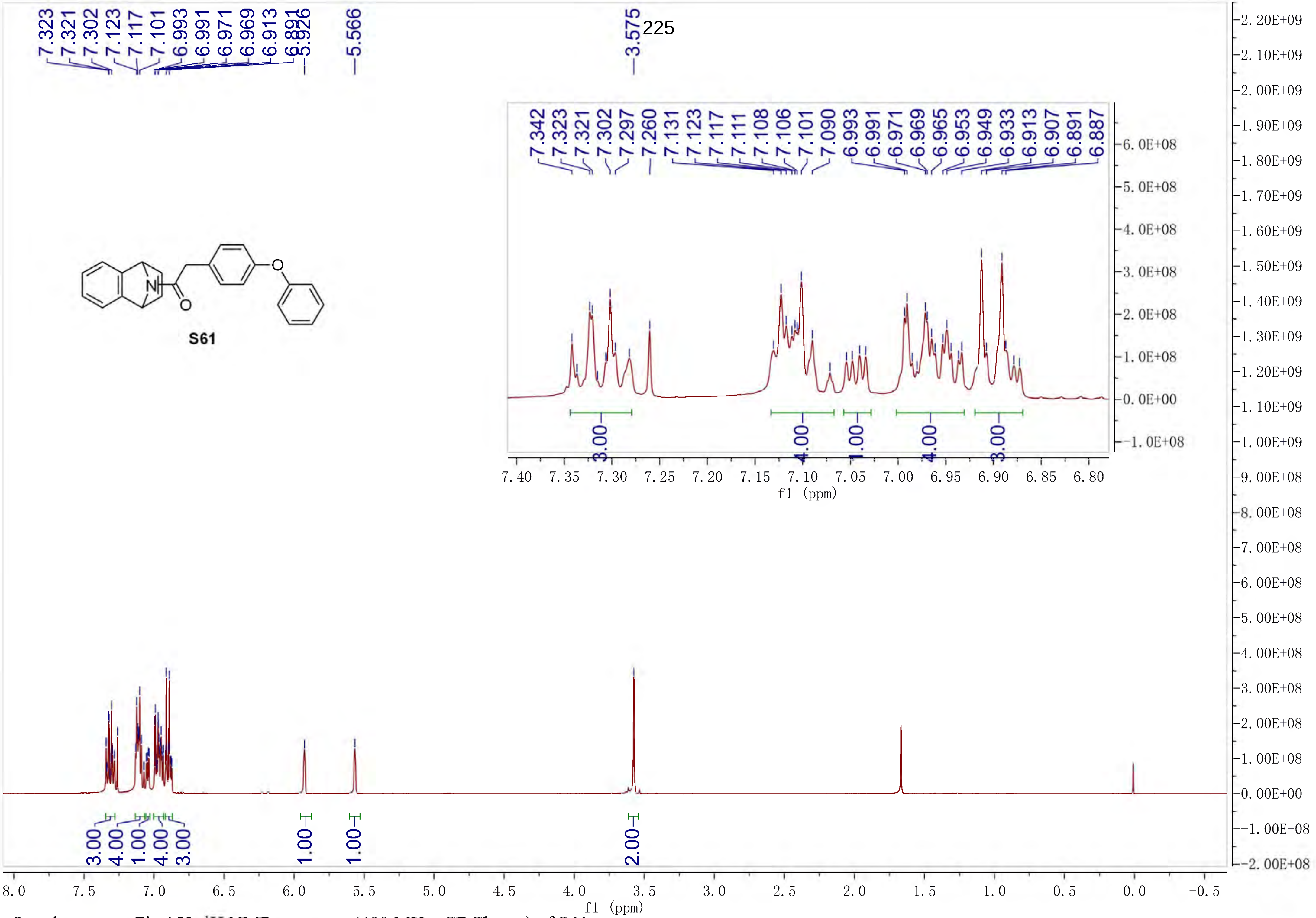

Supplementary Fig 153. ^{1}H NMR spectrum (400 MHz, $CDCl_3$, r.t.) of **S61**.

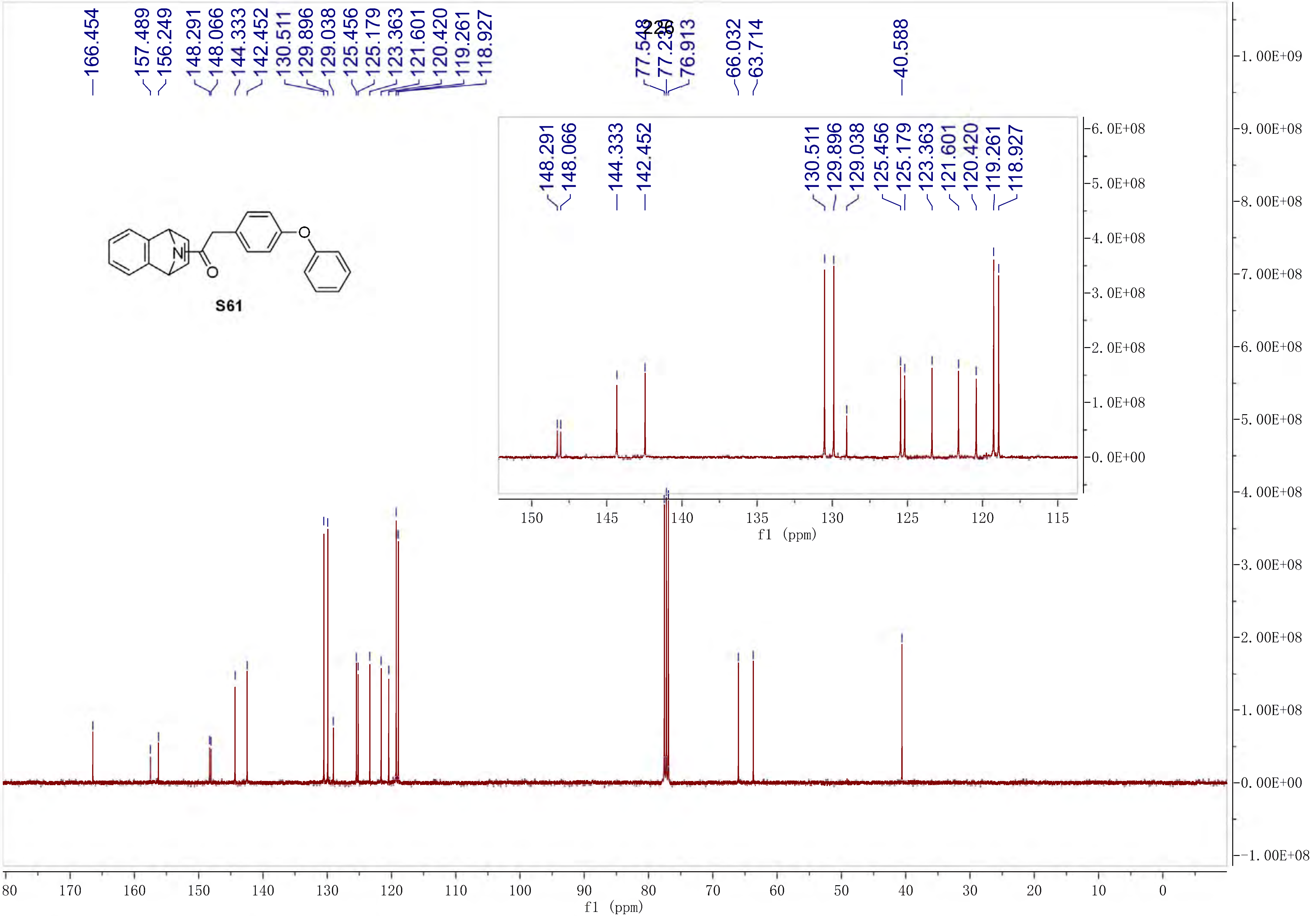

Supplementary Fig 154. ^{13}C NMR spectrum (400 MHz, $CDCl_3$, r.t.) of **S61**.

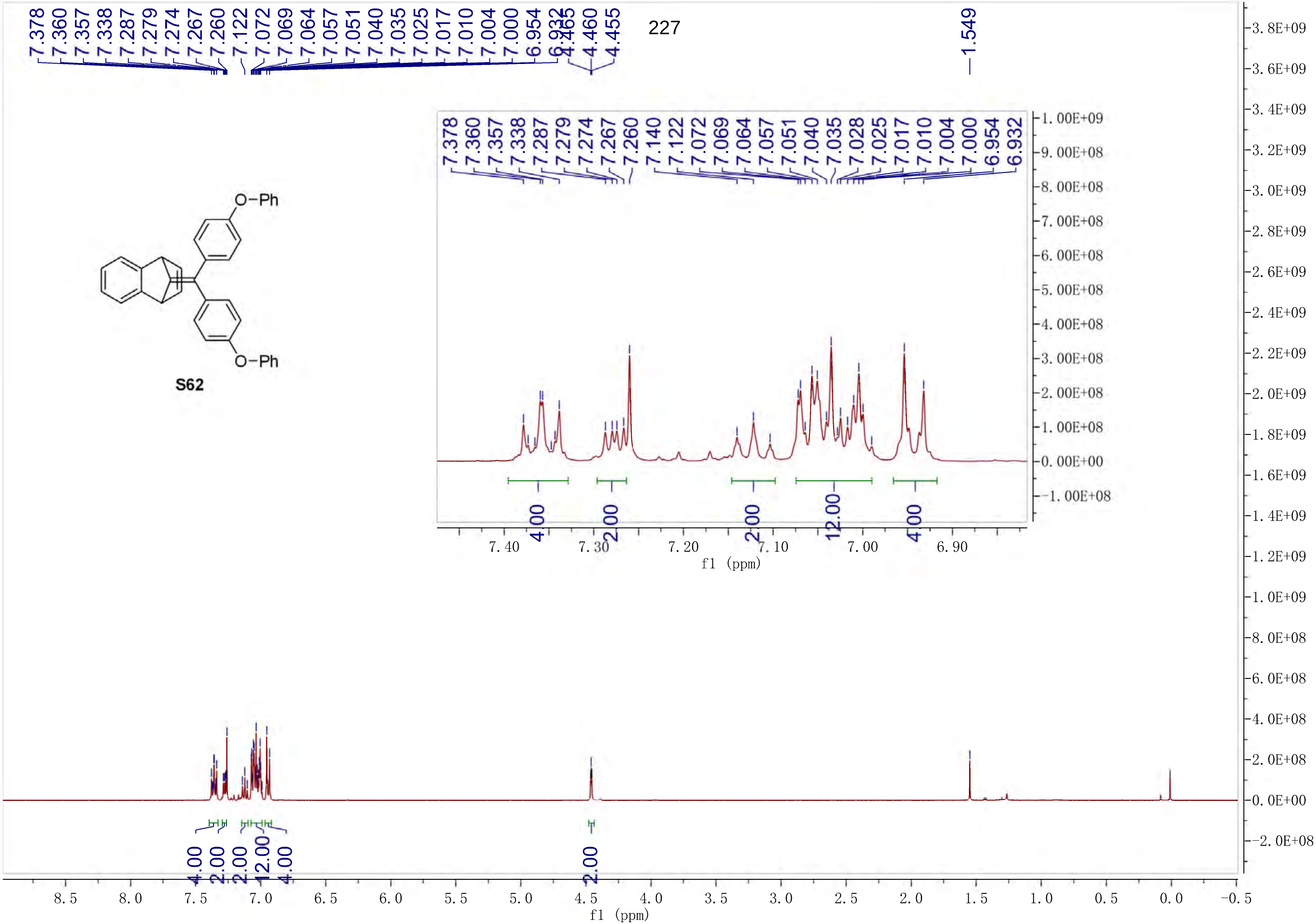

Supplementary Fig 155. ^{1}H NMR spectrum (400 MHz, $CDCl_3$, r.t.) of **S62**.

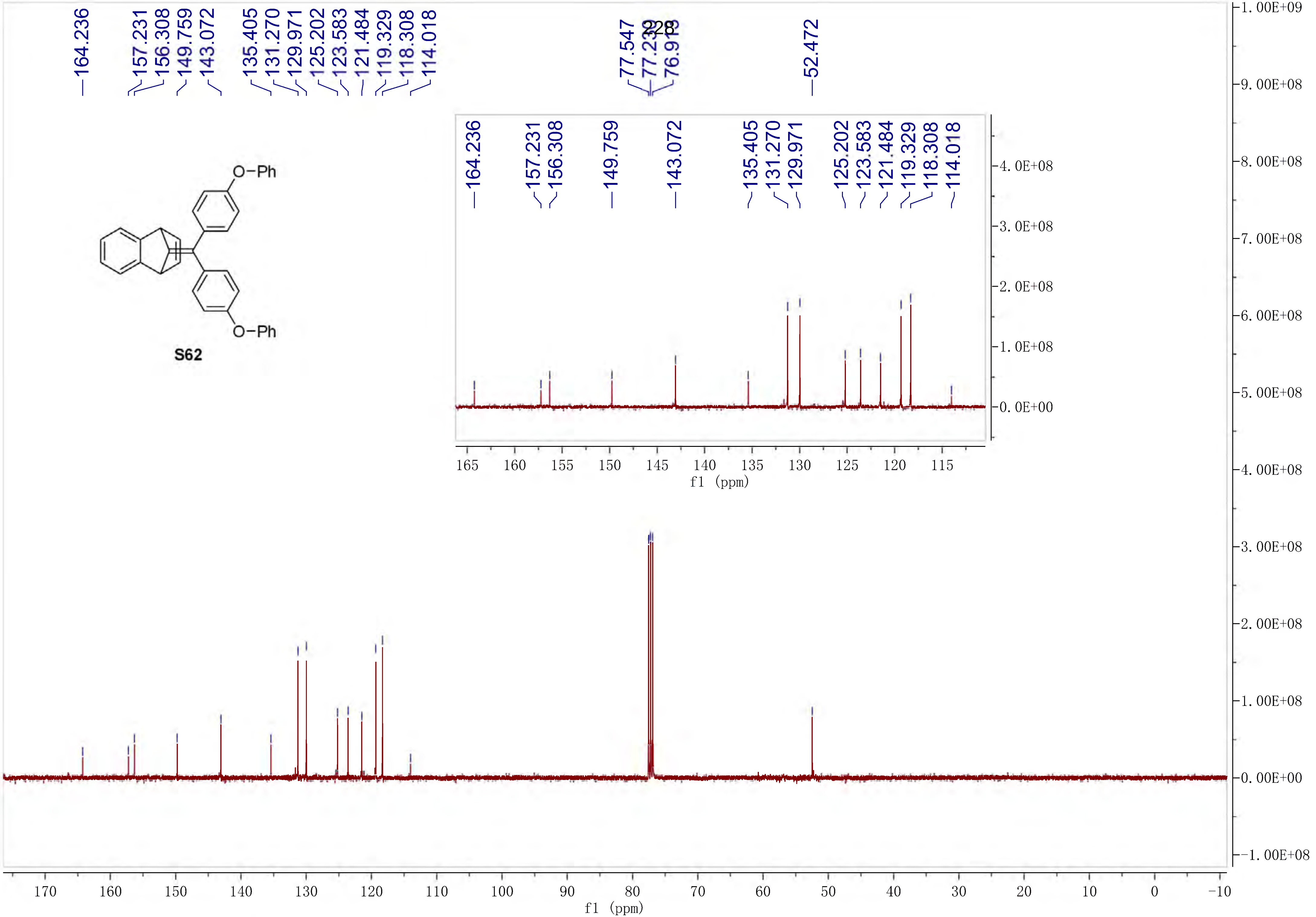

Supplementary Fig 156. ^{13}C NMR spectrum (400 MHz, $CDCl_3$, r.t.) of **S62**.

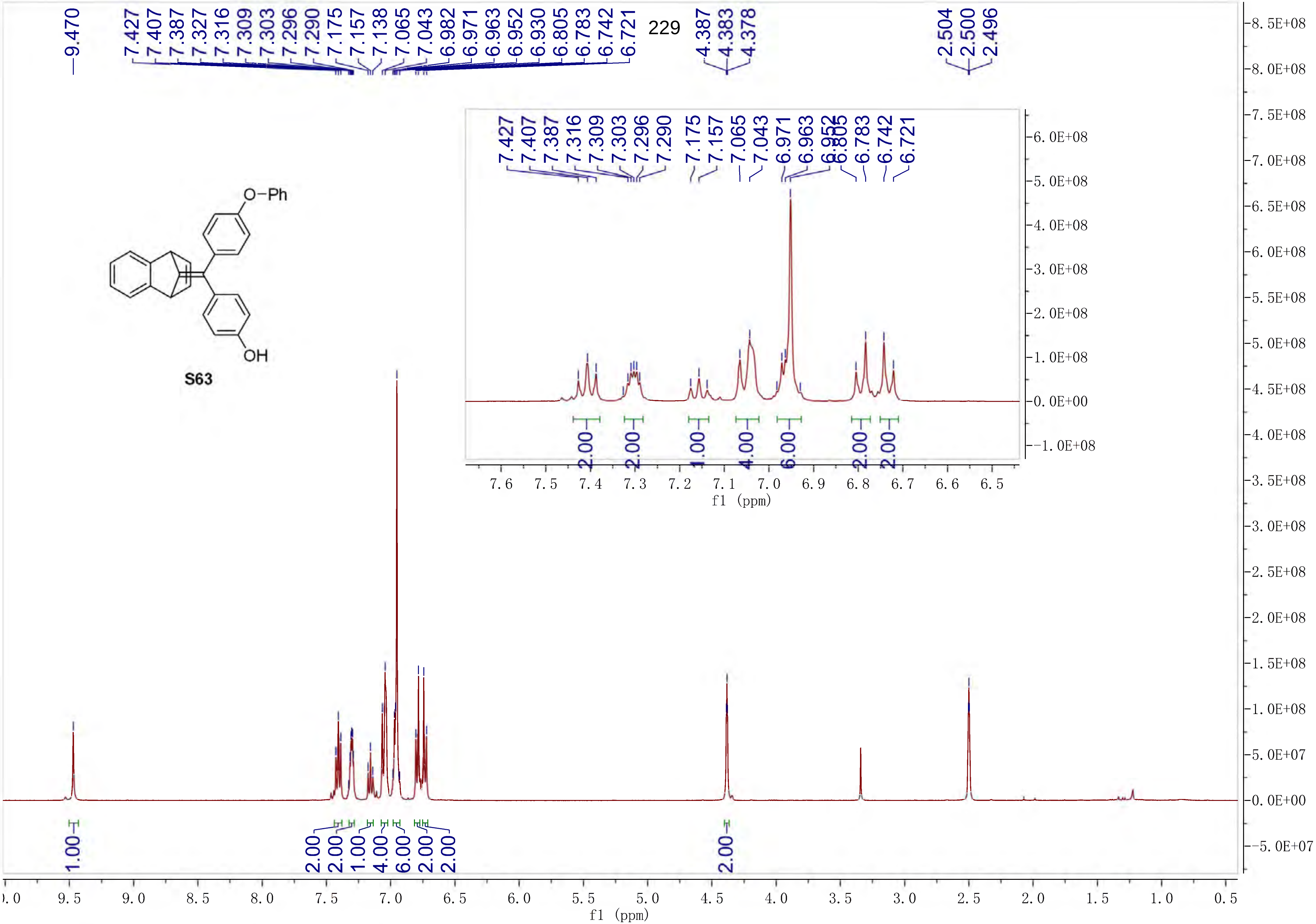

Supplementary Fig 157. ^{1}H NMR spectrum (400 MHz, $CDCl_3$, r.t.) of **S63**.

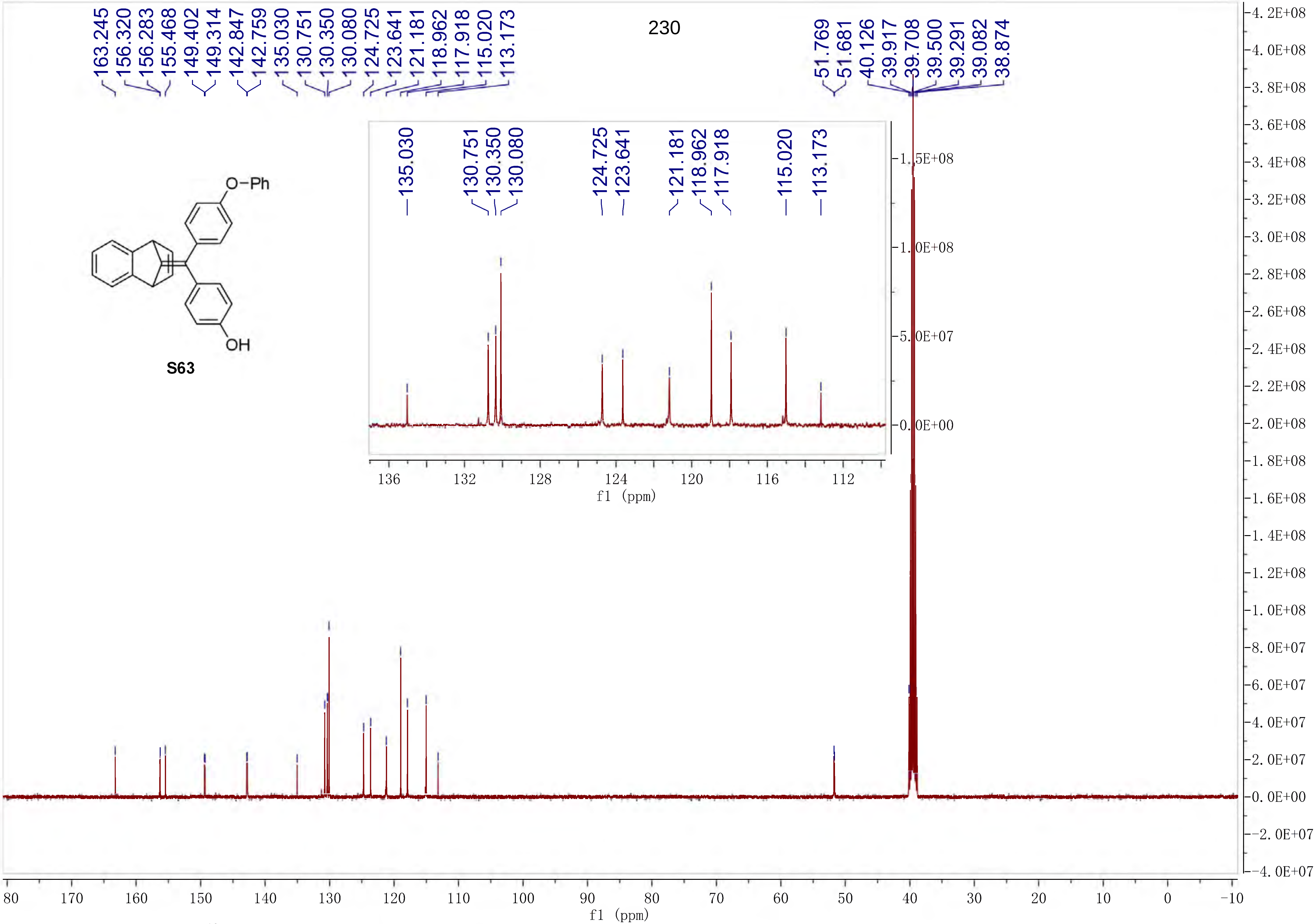

Supplementary Fig 158. ^{13}C NMR spectrum (400 MHz, $CDCl_3$, r.t.) of **S63**.

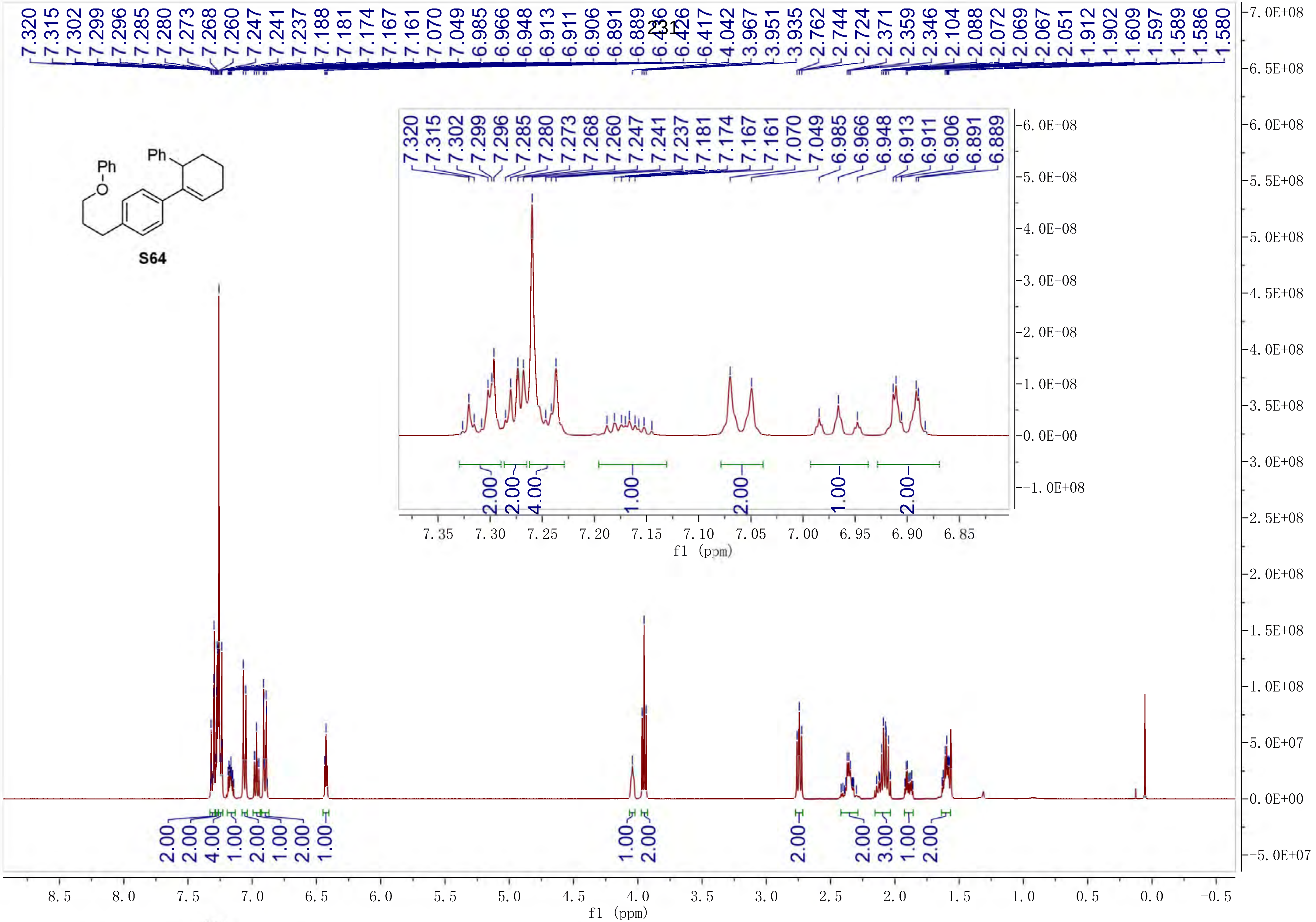

Supplementary Fig 159. ^{1}H NMR spectrum (400 MHz, $CDCl_3$, r.t.) of **S64**.

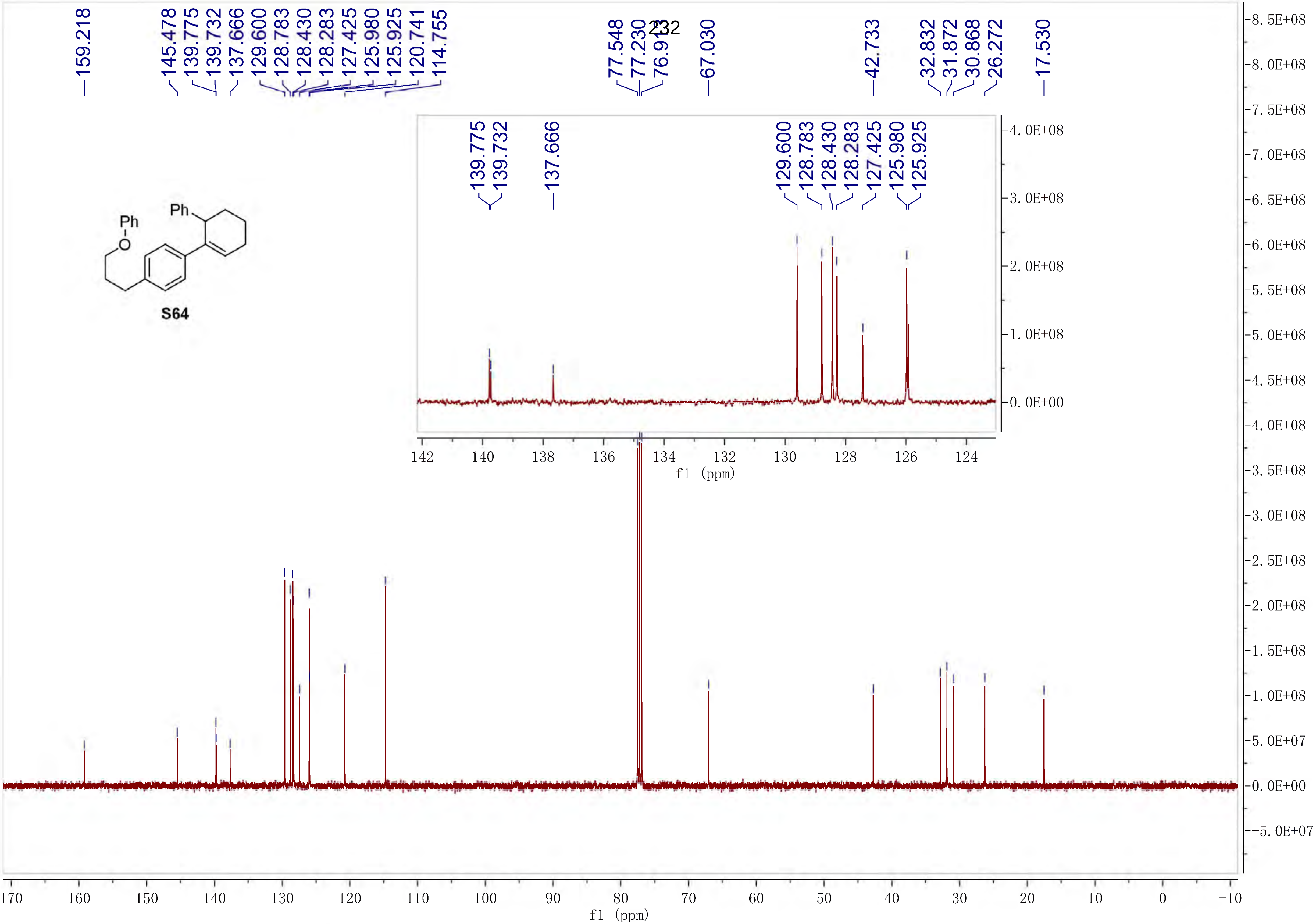

Supplementary Fig 160. ^{13}C NMR spectrum (400 MHz, $CDCl_3$, r.t.) of **S64**.

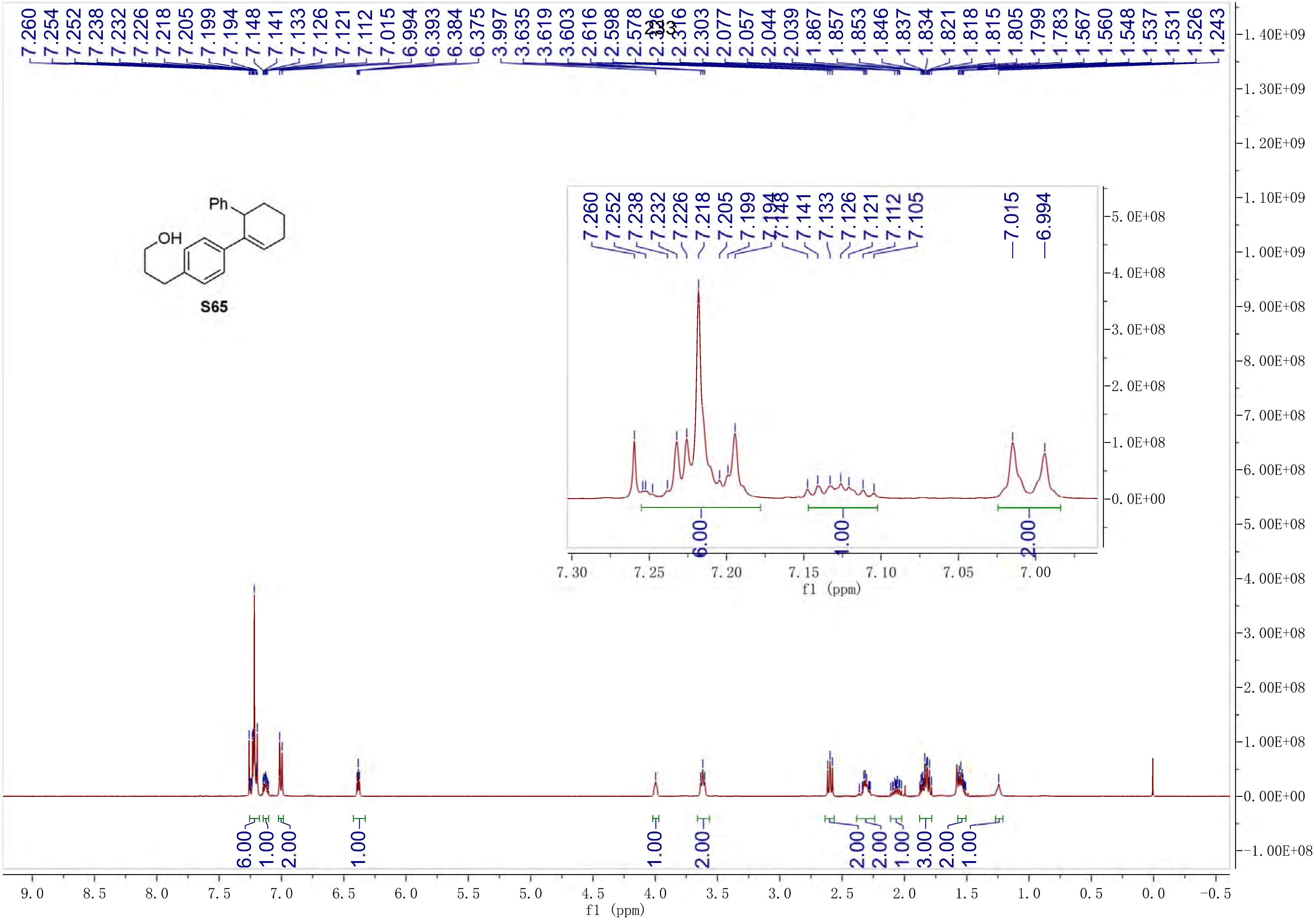

Supplementary Fig 161. ^{1}H NMR spectrum (400 MHz, $CDCl_3$, r.t.) of **S65**.

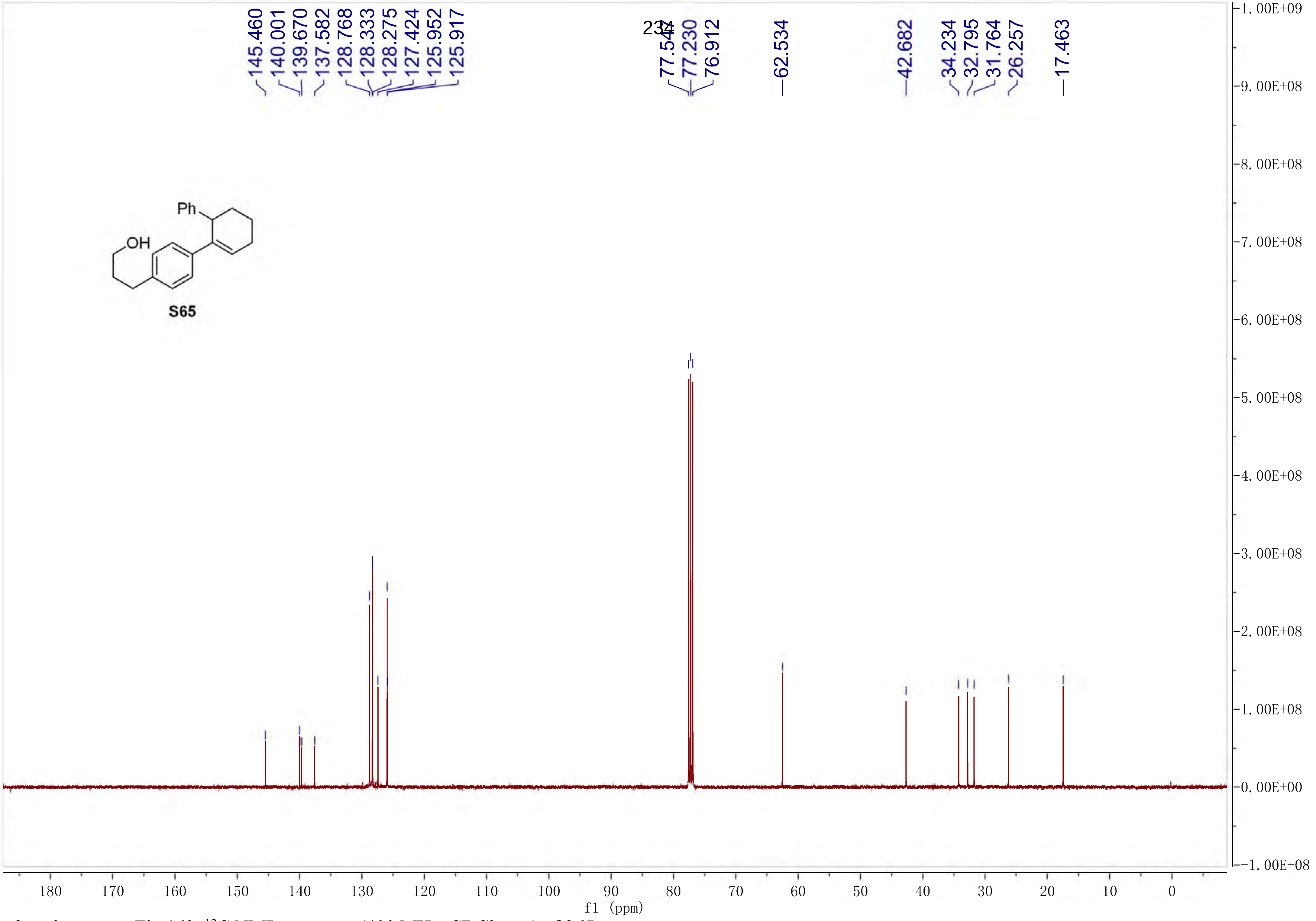

Supplementary Fig 162. ^{13}C NMR spectrum (400 MHz, $CDCl_3$, r.t.) of **S65**.

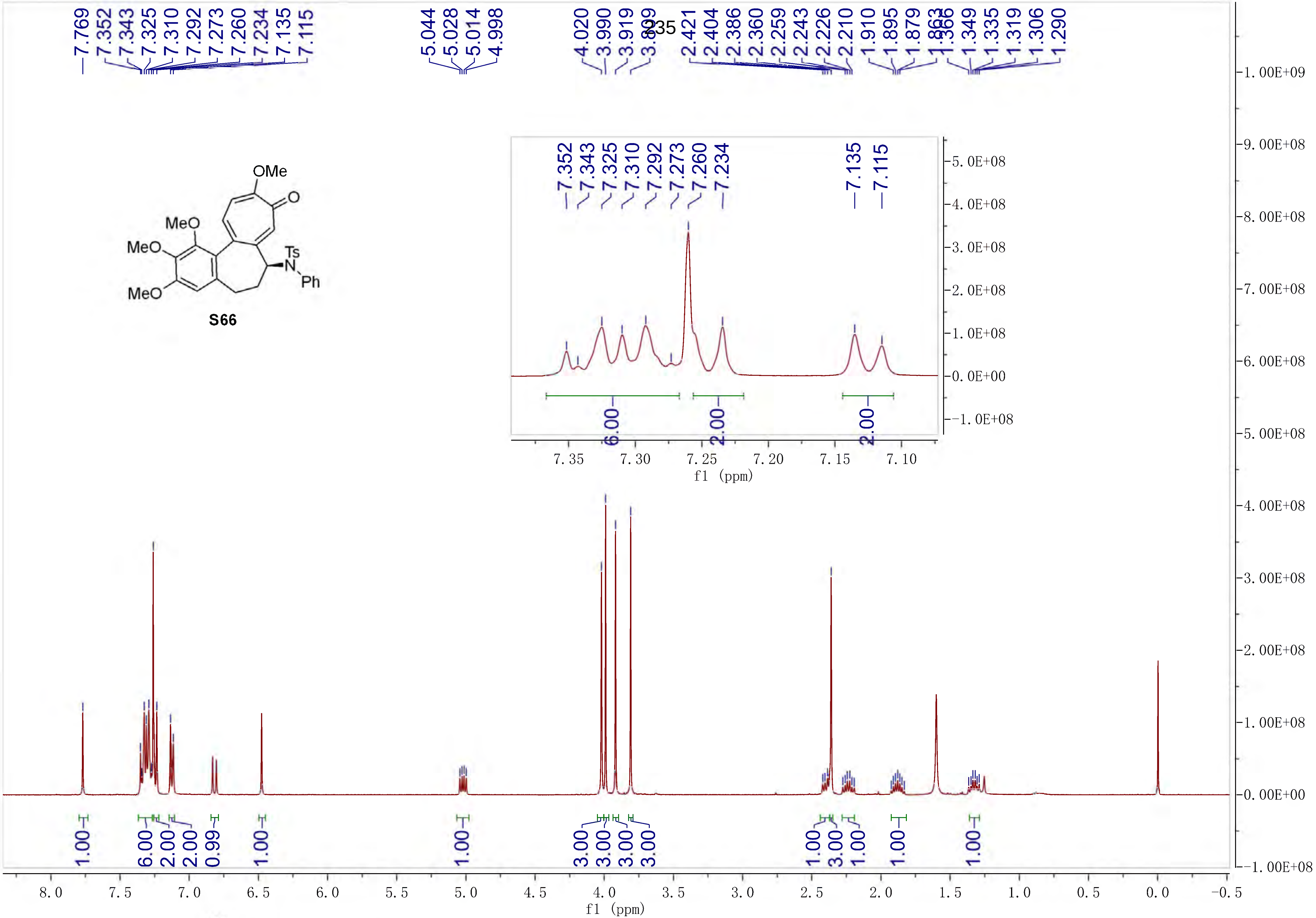

Supplementary Fig 163. ^{1}H NMR spectrum (400 MHz, $CDCl_3$, r.t.) of **S66**.

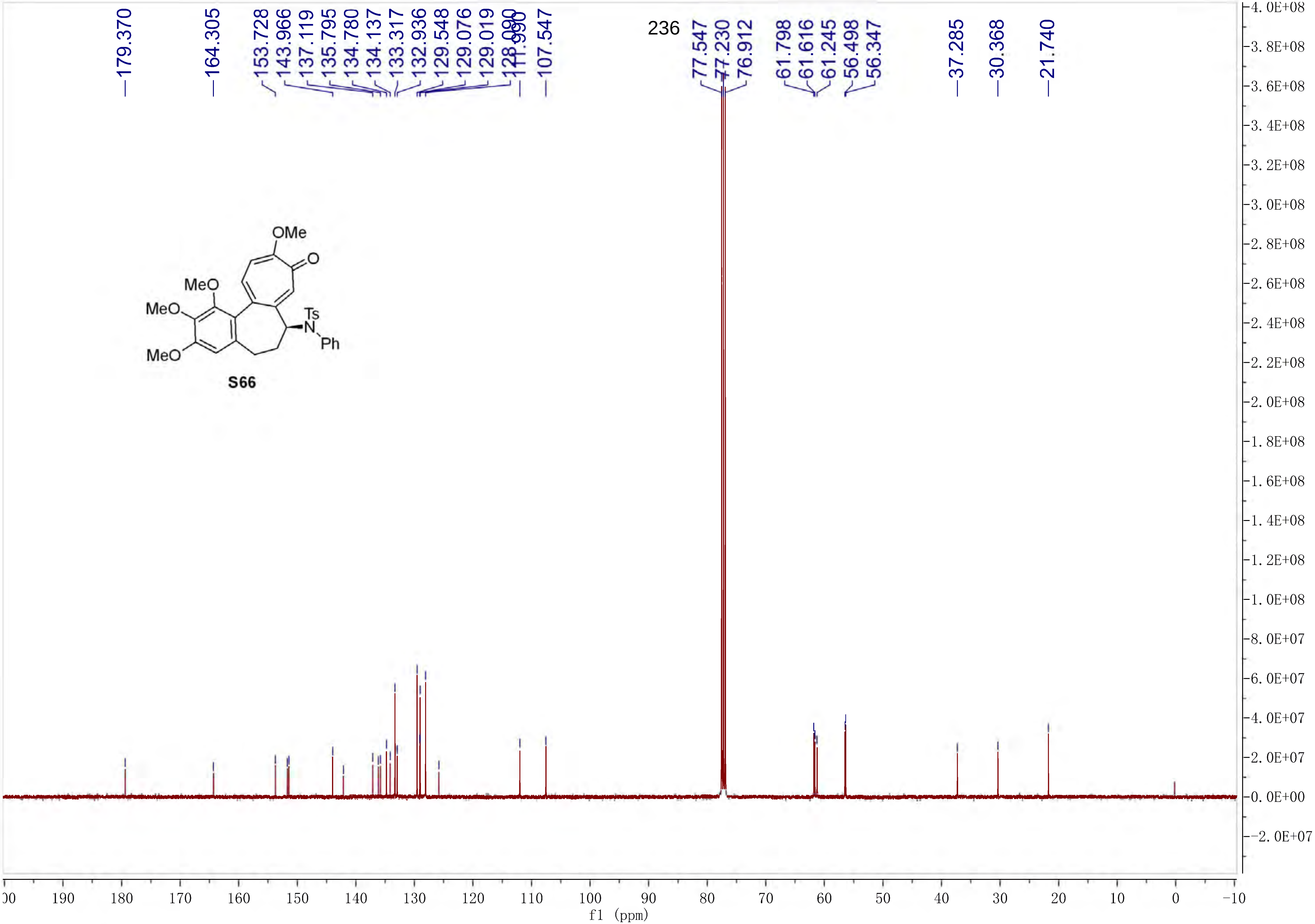

Supplementary Fig 164. ^{13}C NMR spectrum (400 MHz, $CDCl_3$, r.t.) of **S66**.

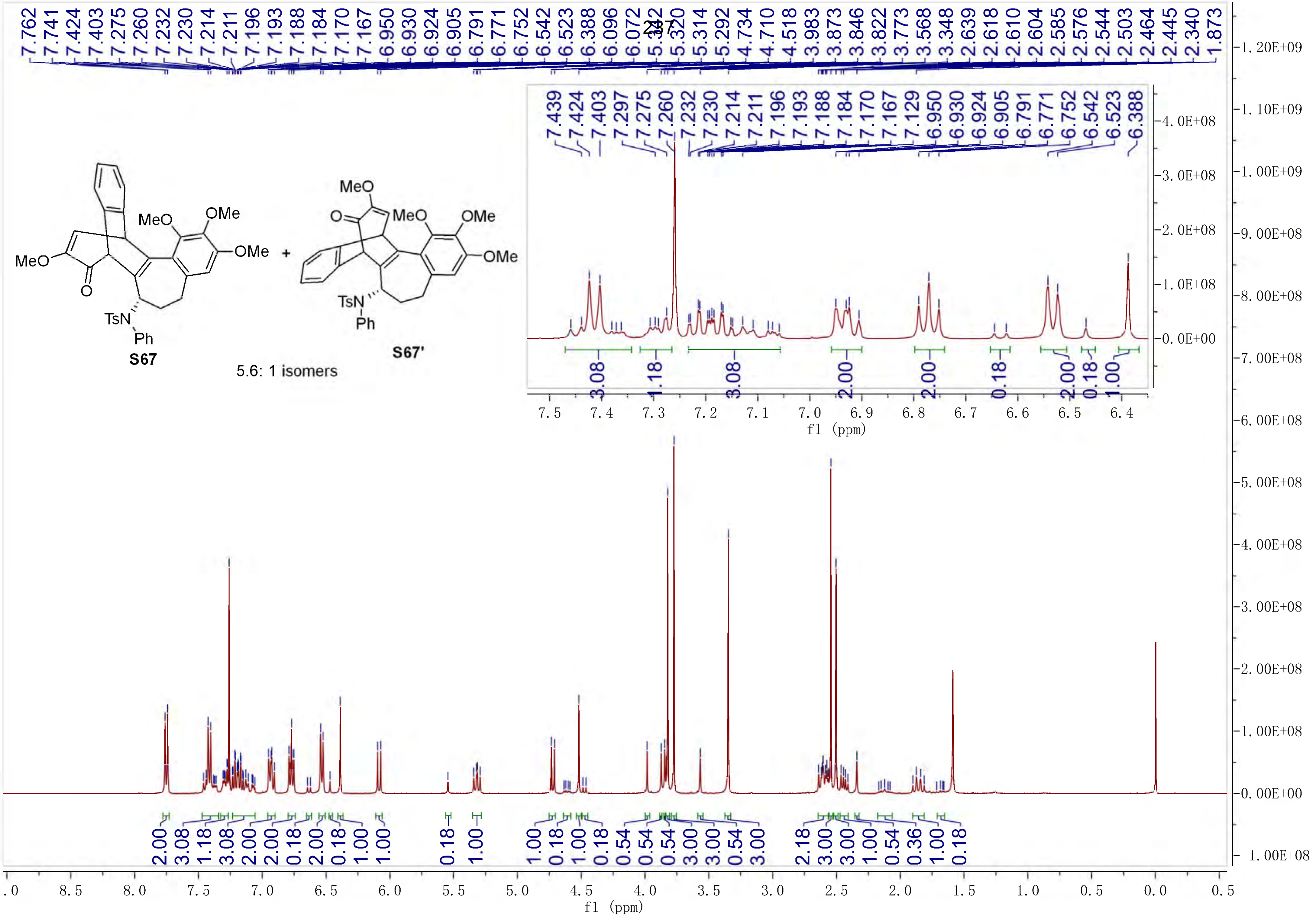

Supplementary Fig 165. ^{1}H NMR spectrum (400 MHz, $CDCl_3$, r.t.) of **S67** and **S67'**.

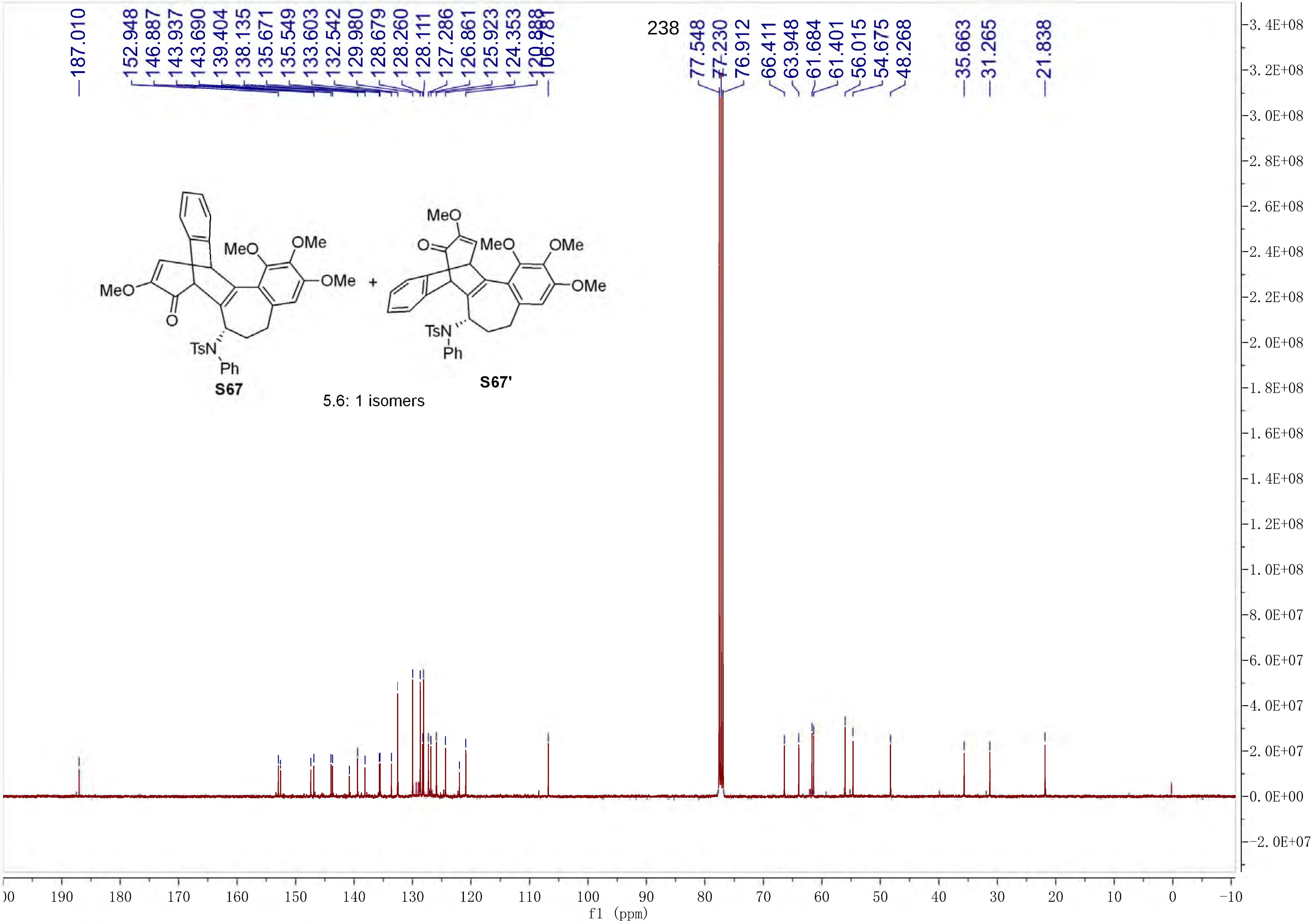

Supplementary Fig 166. ^{13}C NMR spectrum (400 MHz, $CDCl_3$, r.t.) of **S67** and **S67'**.

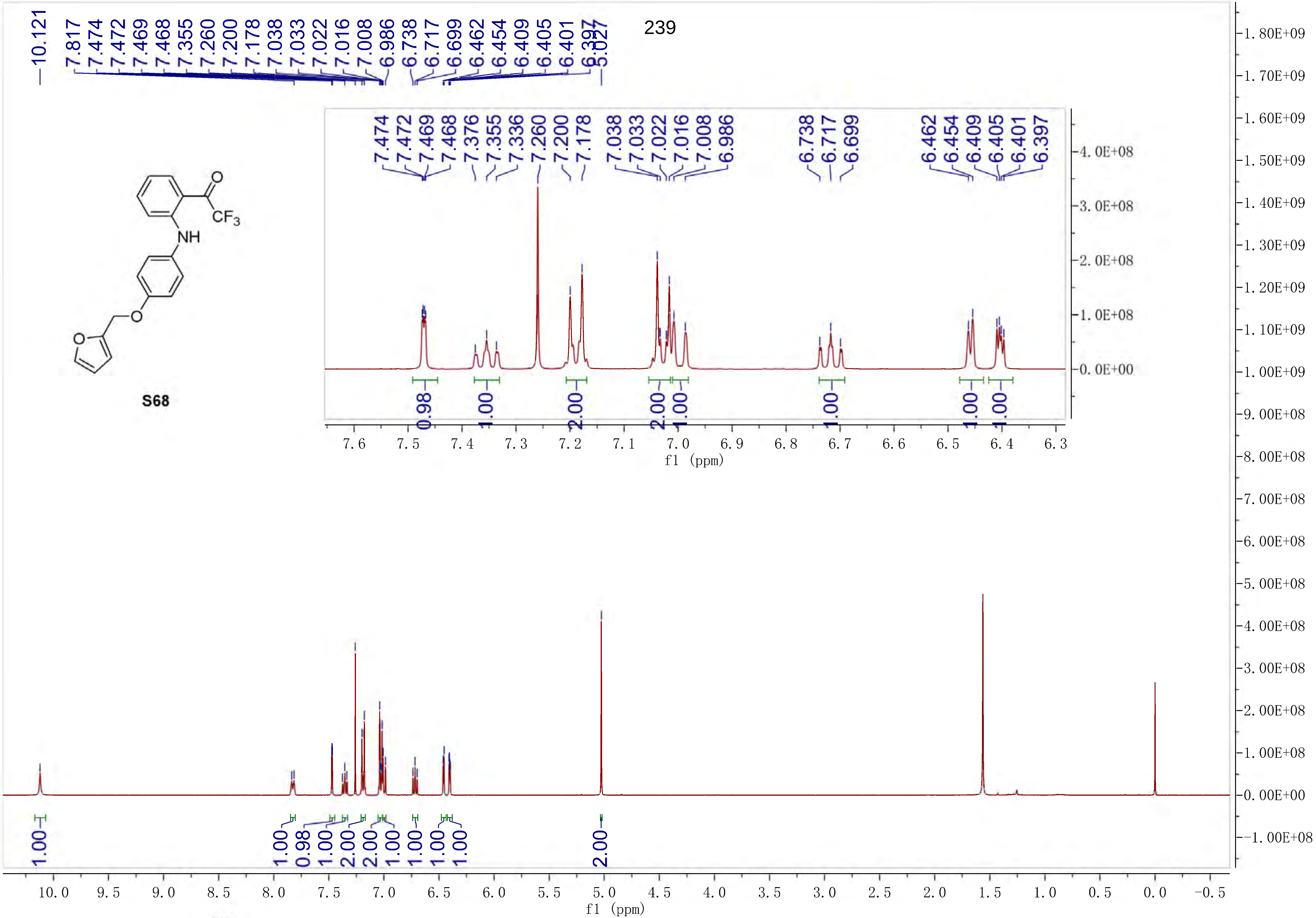

Supplementary Fig 167. ¹H NMR spectrum (400 MHz, $CDCl_3$, r.t.) of **S68**.

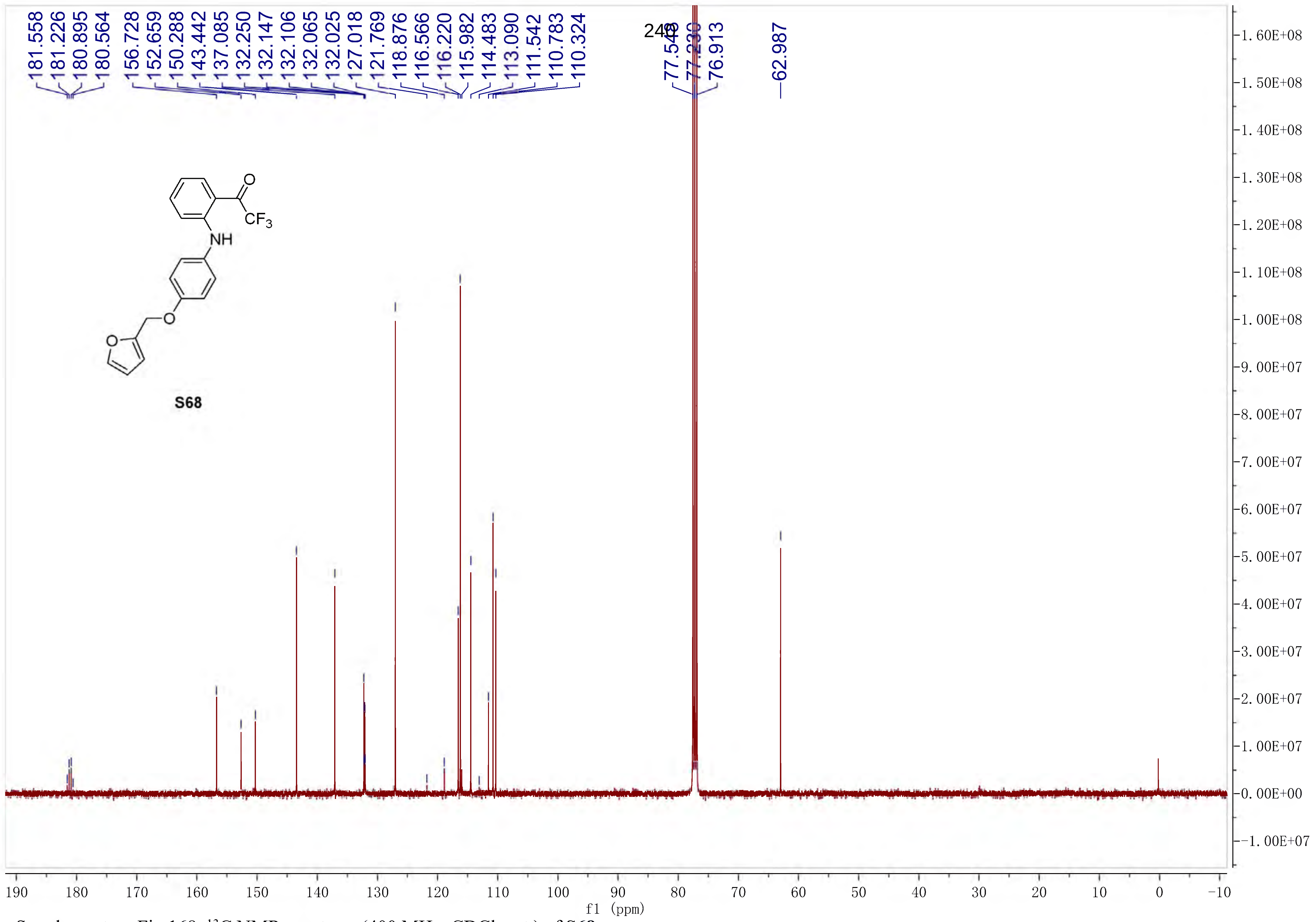

Supplementary Fig 168. ^{13}C NMR spectrum (400 MHz, $CDCl_3$, r.t.) of **S68**.

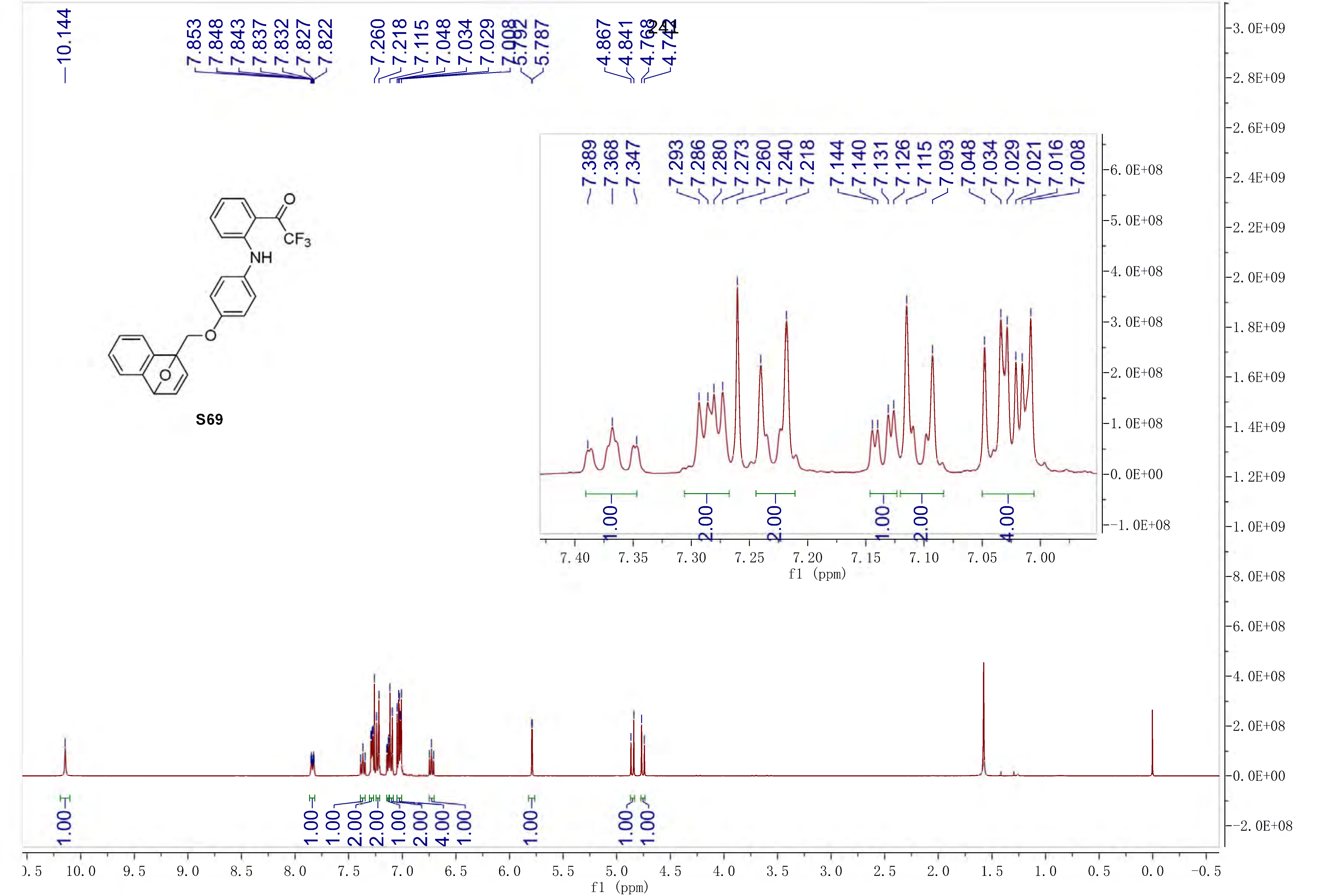

Supplementary Fig 169. ^{1}H NMR spectrum (400 MHz, $CDCl_3$, r.t.) of **S69**.

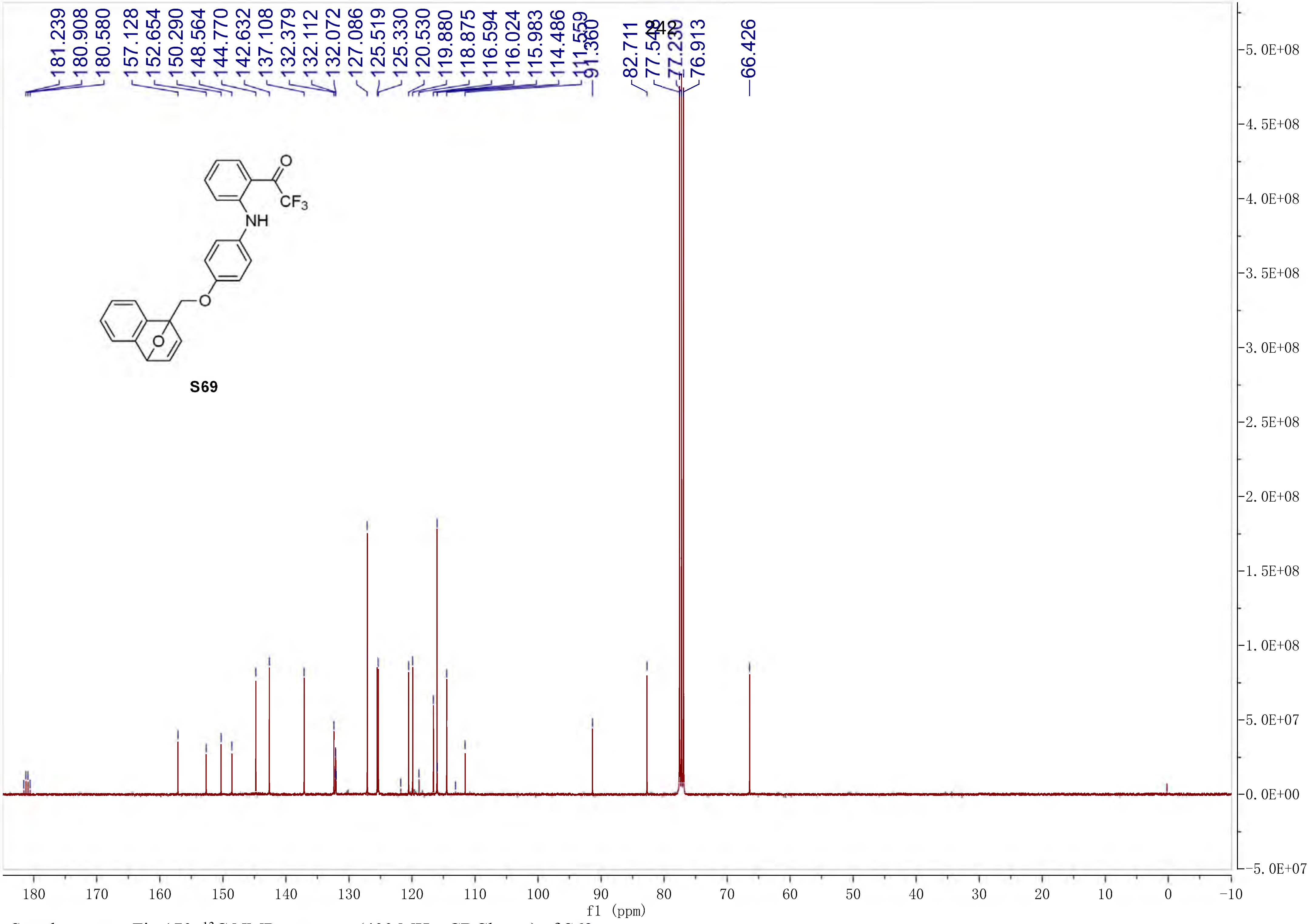

Supplementary Fig 170. ^{13}C NMR spectrum (400 MHz, $CDCl_3$, r.t.) of **S69**.

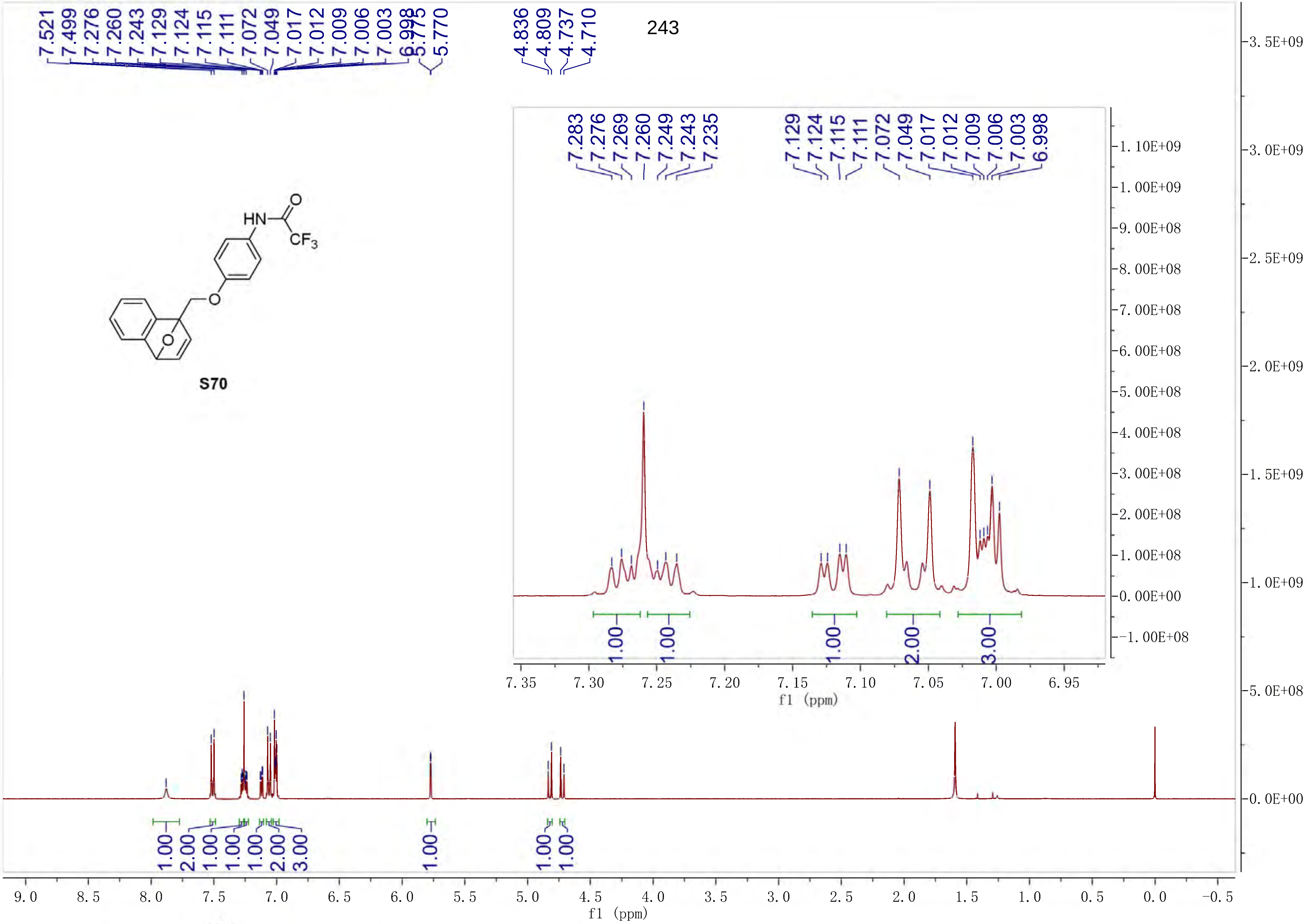

Supplementary Fig 171. ^{1}H NMR spectrum (400 MHz, $CDCl_3$, r.t.) of **S70**.

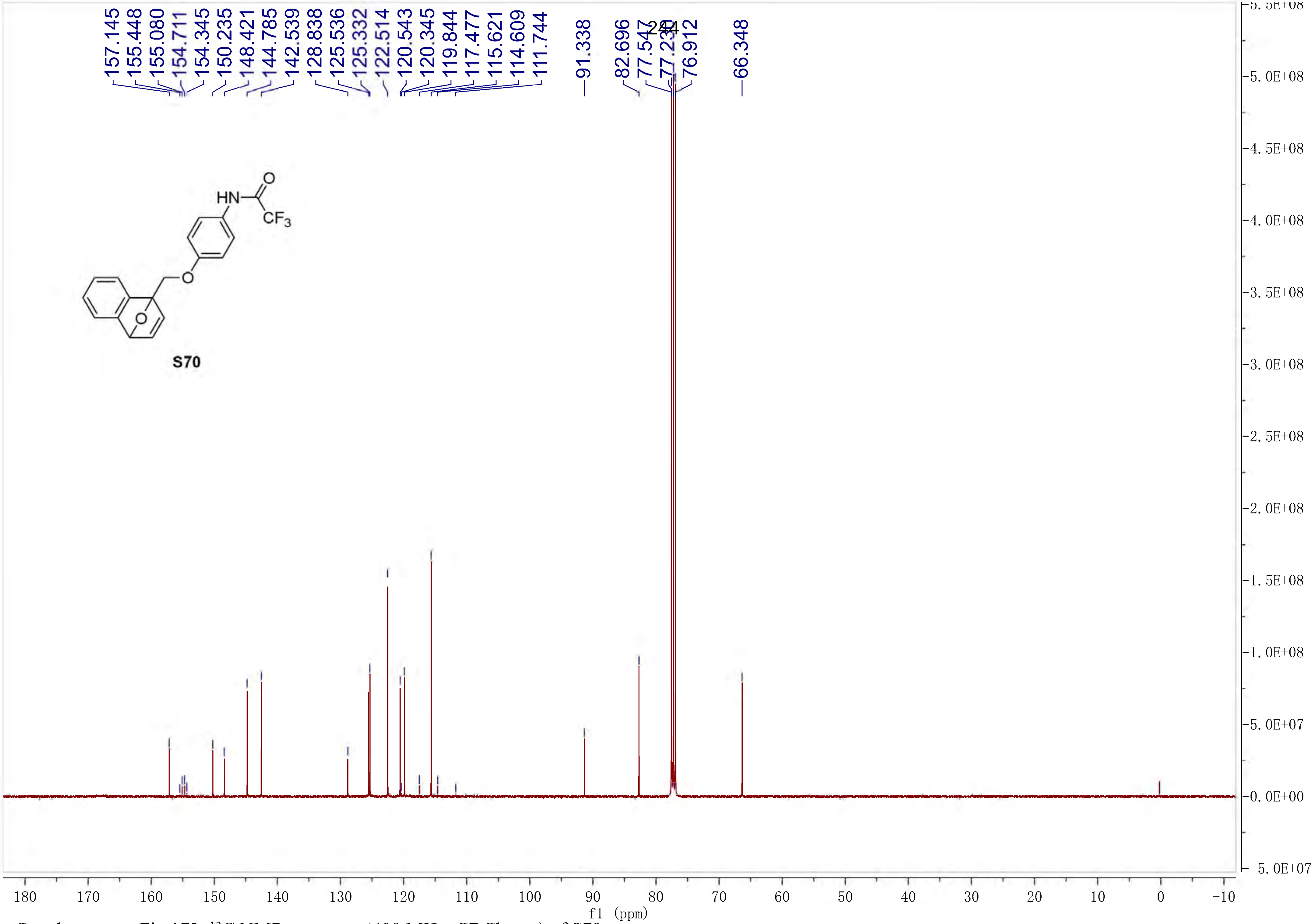

Supplementary Fig 172. ^{13}C NMR spectrum (400 MHz, $CDCl_3$, r.t.) of **S70**.

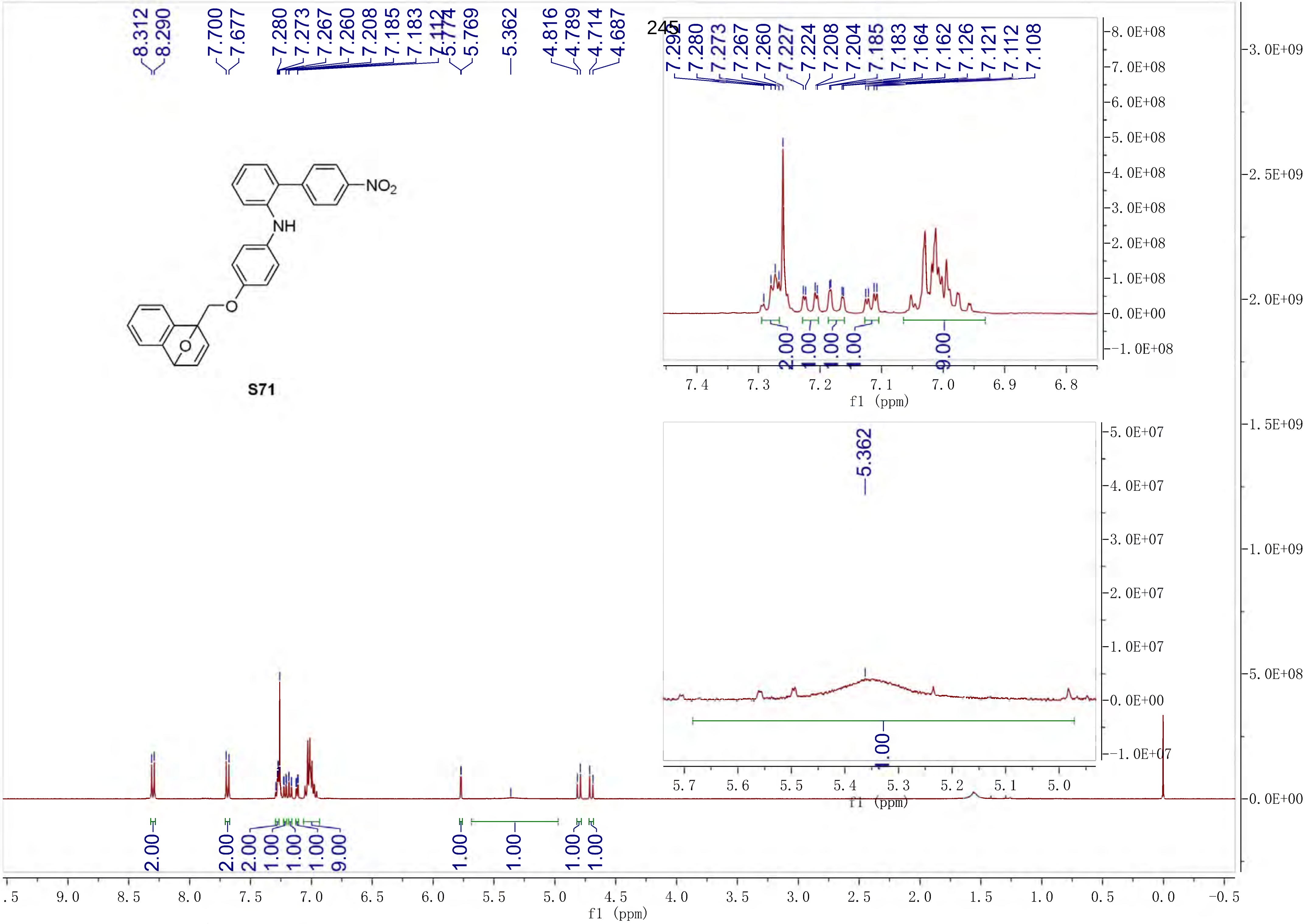

Supplementary Fig 173. ^{1}H NMR spectrum (400 MHz, $CDCl_3$, r.t.) of **S71**.

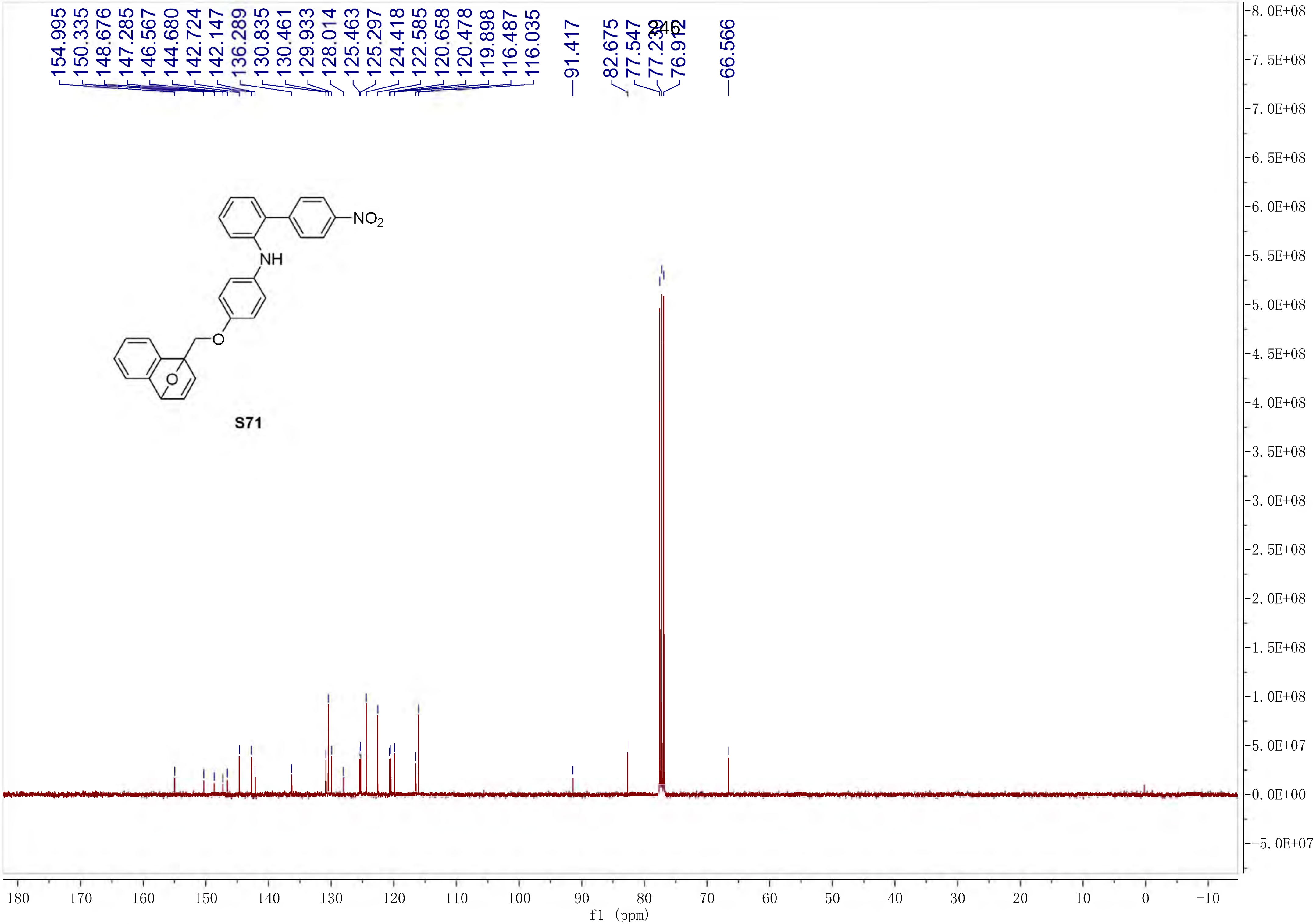

Supplementary Fig 174. ^{13}C NMR spectrum (400 MHz, $CDCl_3$, r.t.) of **S71**.

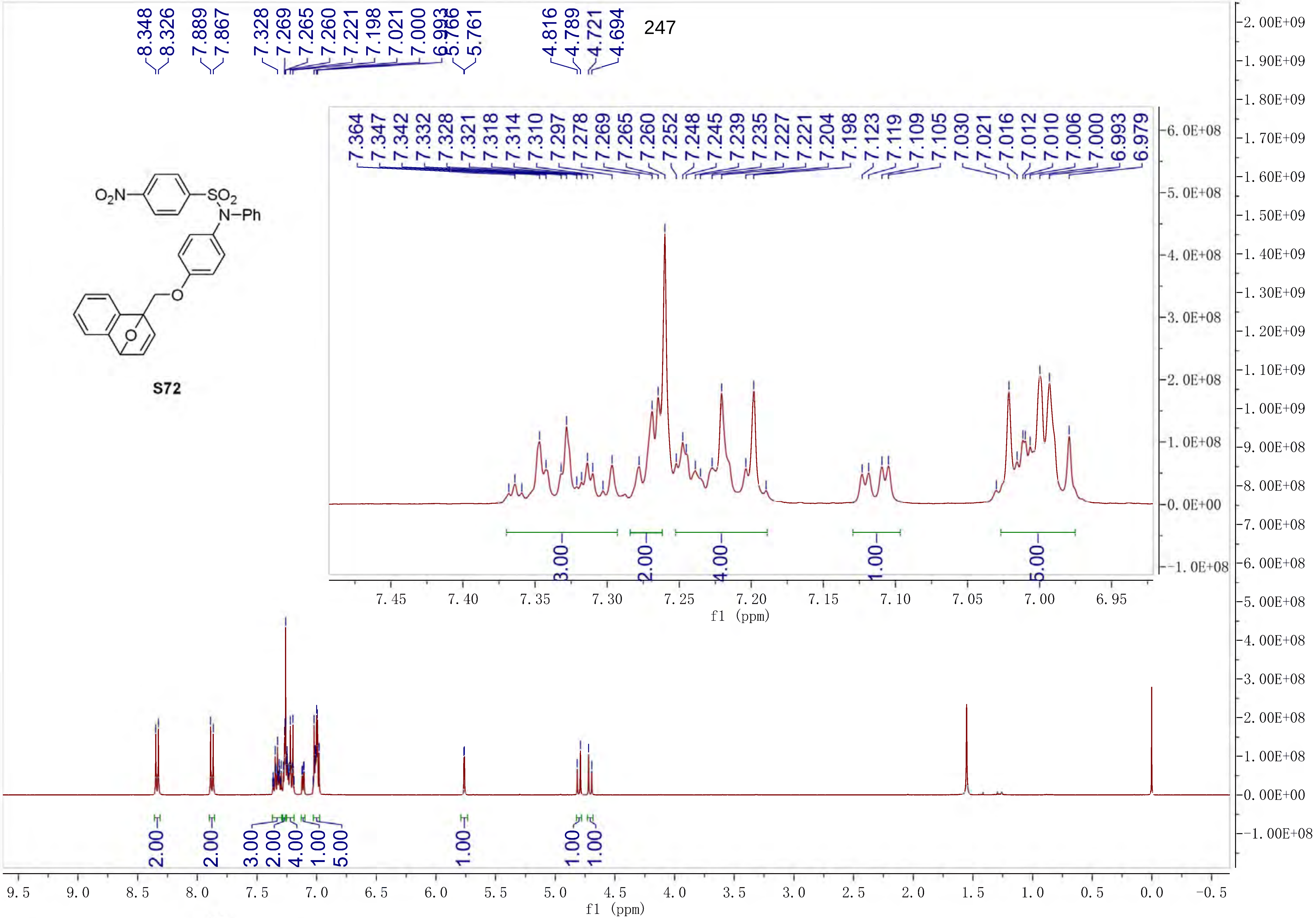

Supplementary Fig 175. ^{1}H NMR spectrum (400 MHz, $CDCl_3$, r.t.) of **S72**.

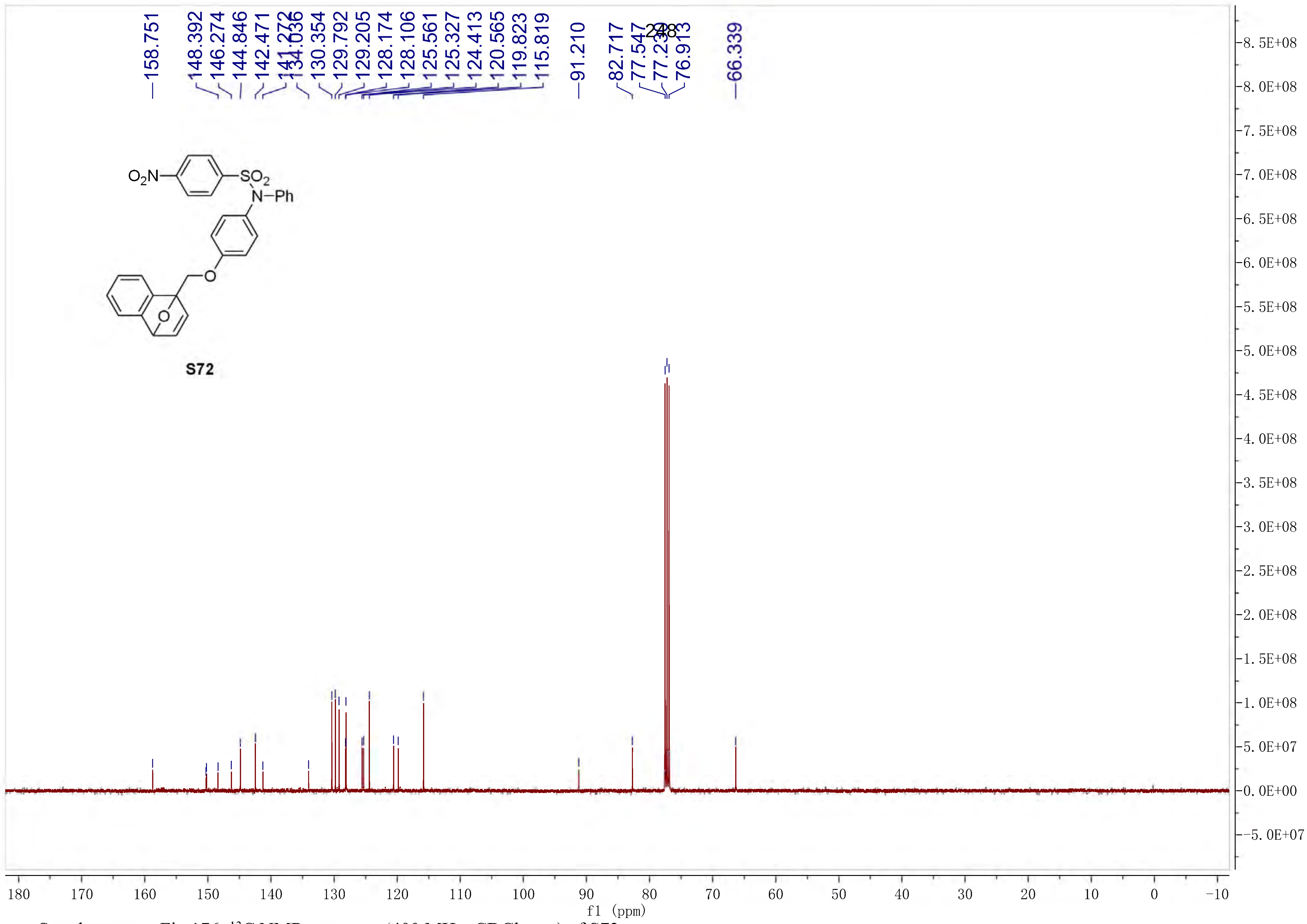

Supplementary Fig 176. ^{13}C NMR spectrum (400 MHz, $CDCl_3$, r.t.) of **S72**.

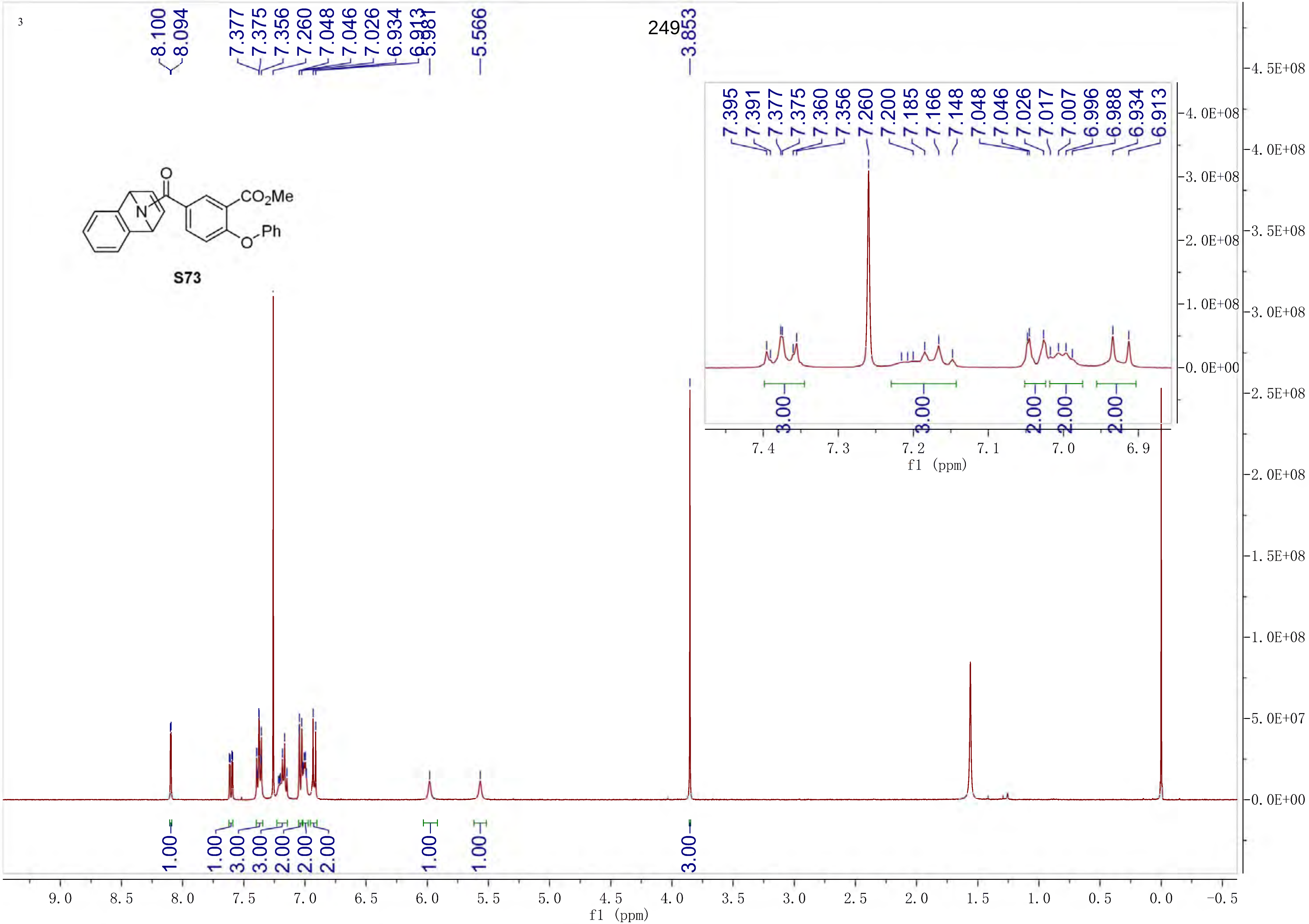

Supplementary Fig 177. ^{1}H NMR spectrum (400 MHz, $CDCl_3$, r.t.) of **S73**.

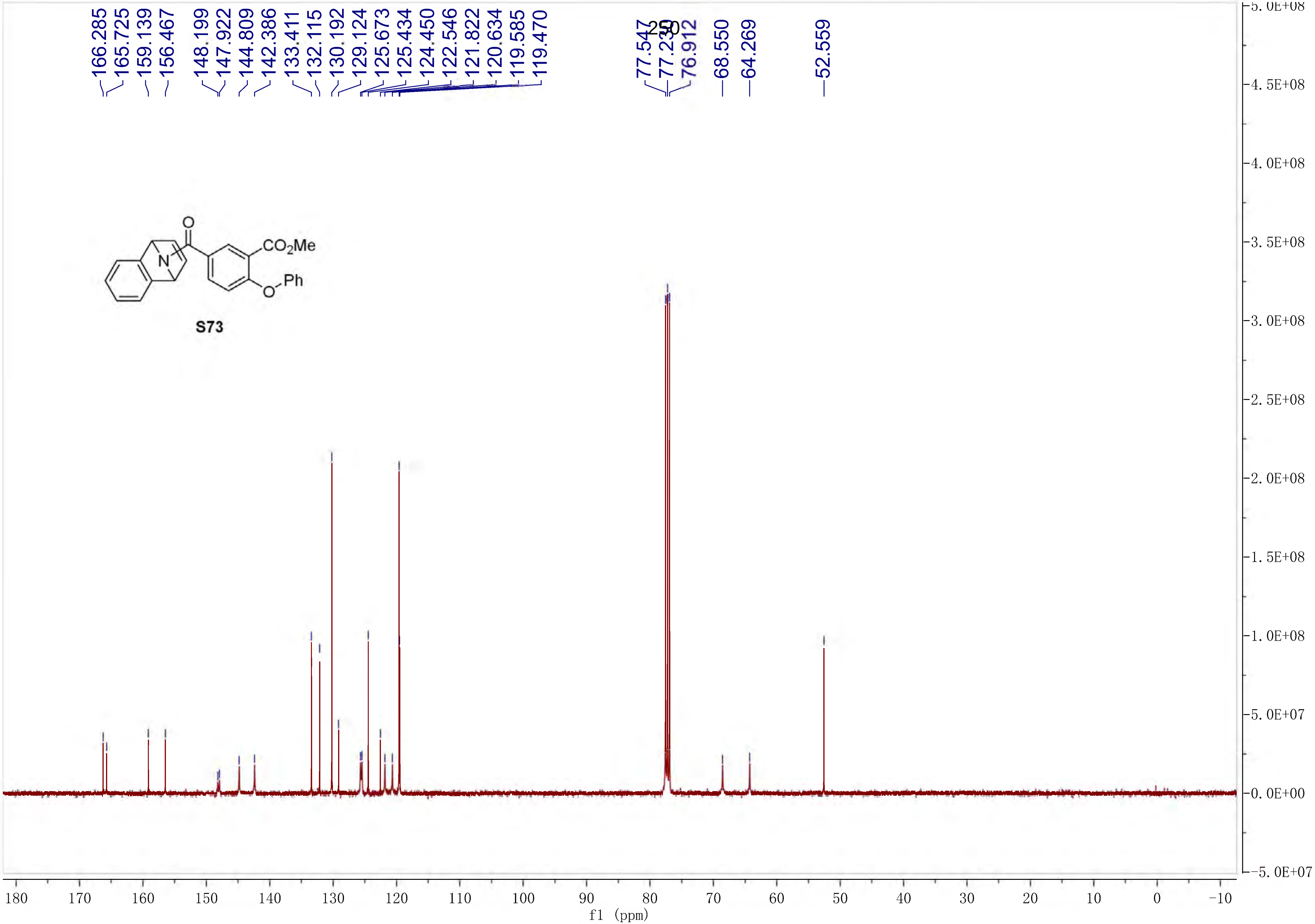

Supplementary Fig 178. ^{13}C NMR spectrum (400 MHz, $CDCl_3$, r.t.) of **S73**.

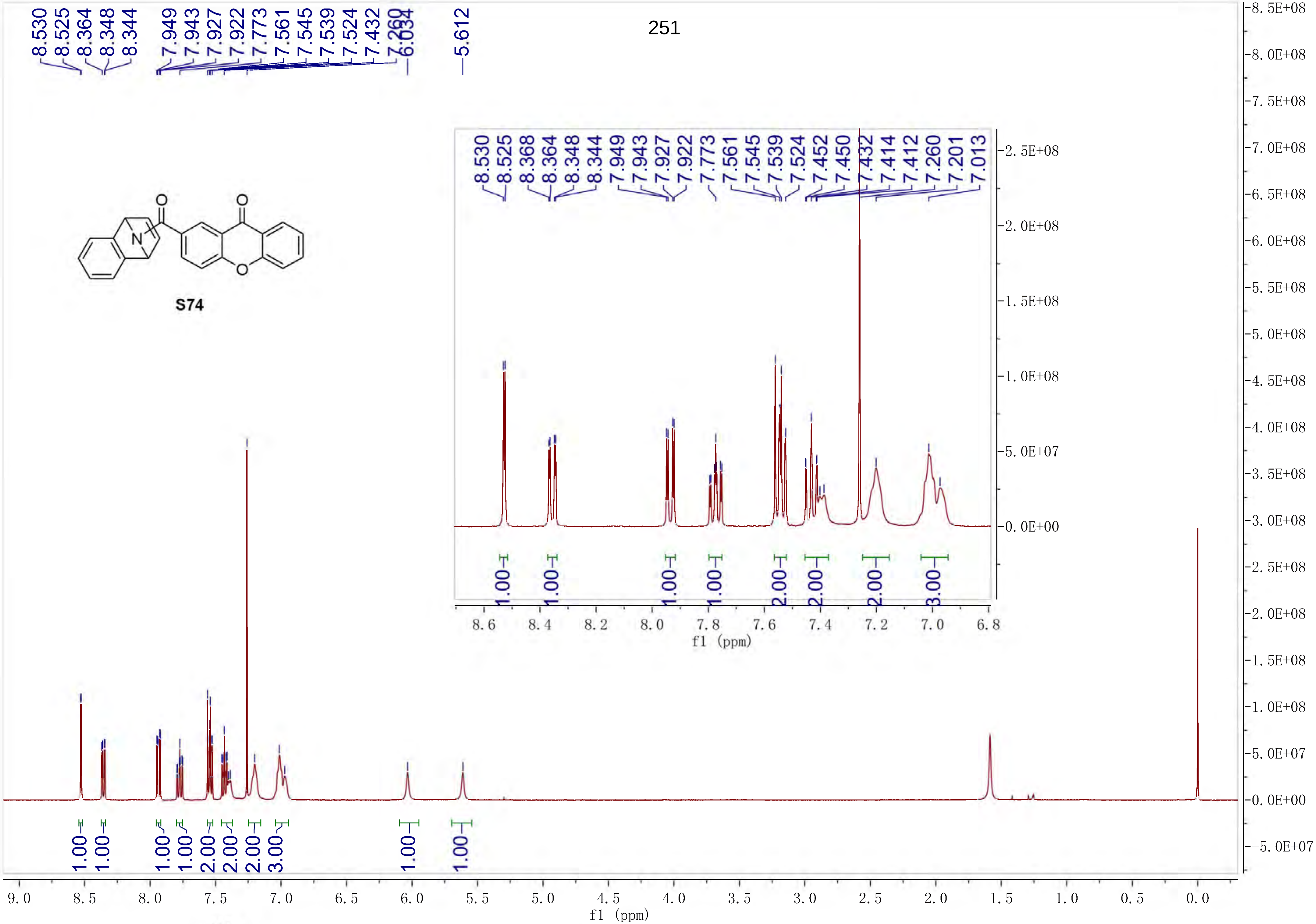

Supplementary Fig 179. ^{1}H NMR spectrum (400 MHz, $CDCl_3$, r.t.) of **S74**.

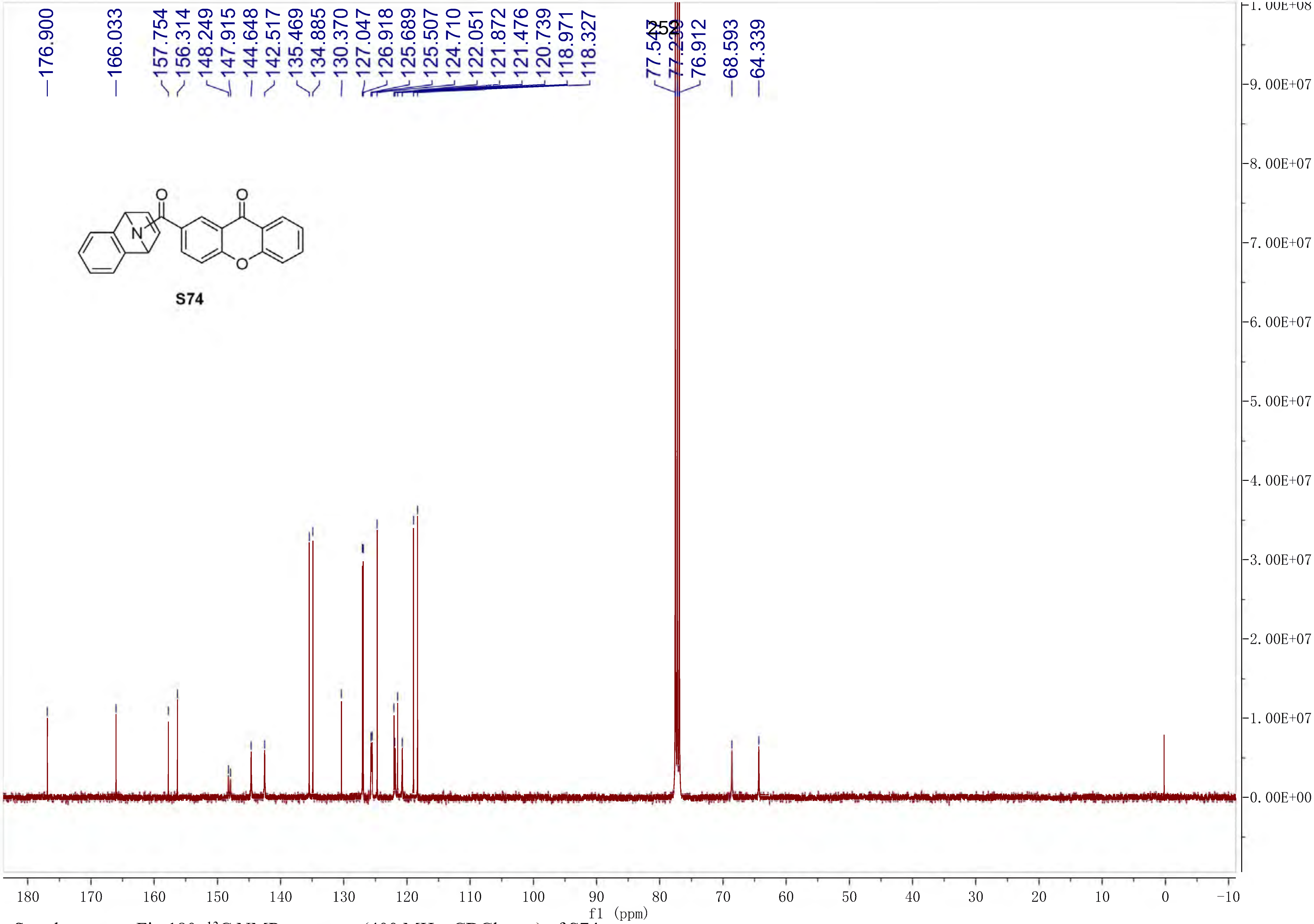

Supplementary Fig 180. ^{13}C NMR spectrum (400 MHz, $CDCl_3$, r.t.) of **S74**.

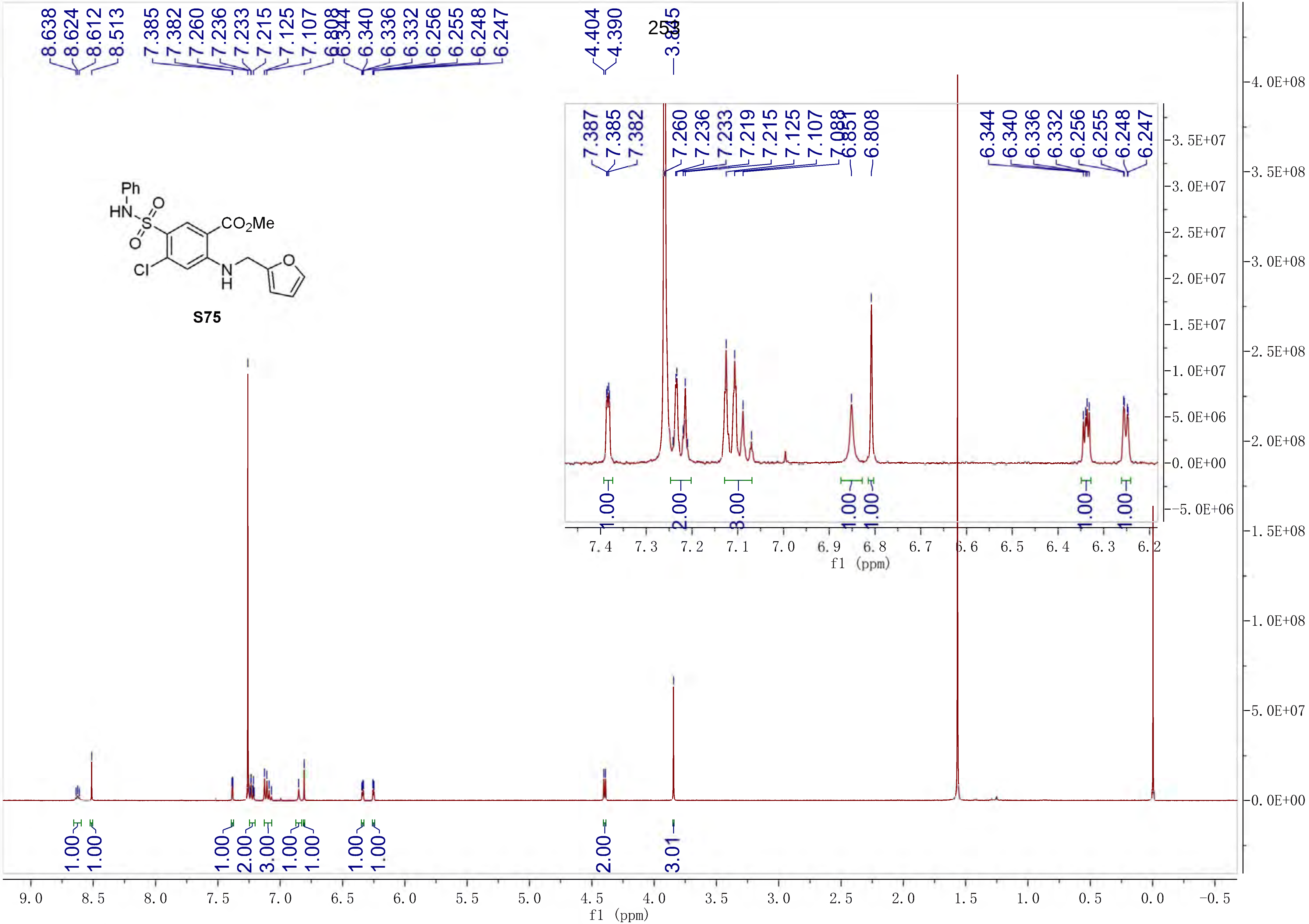

Supplementary Fig 181. ^{1}H NMR spectrum (400 MHz, $CDCl_3$, r.t.) of **S75**.

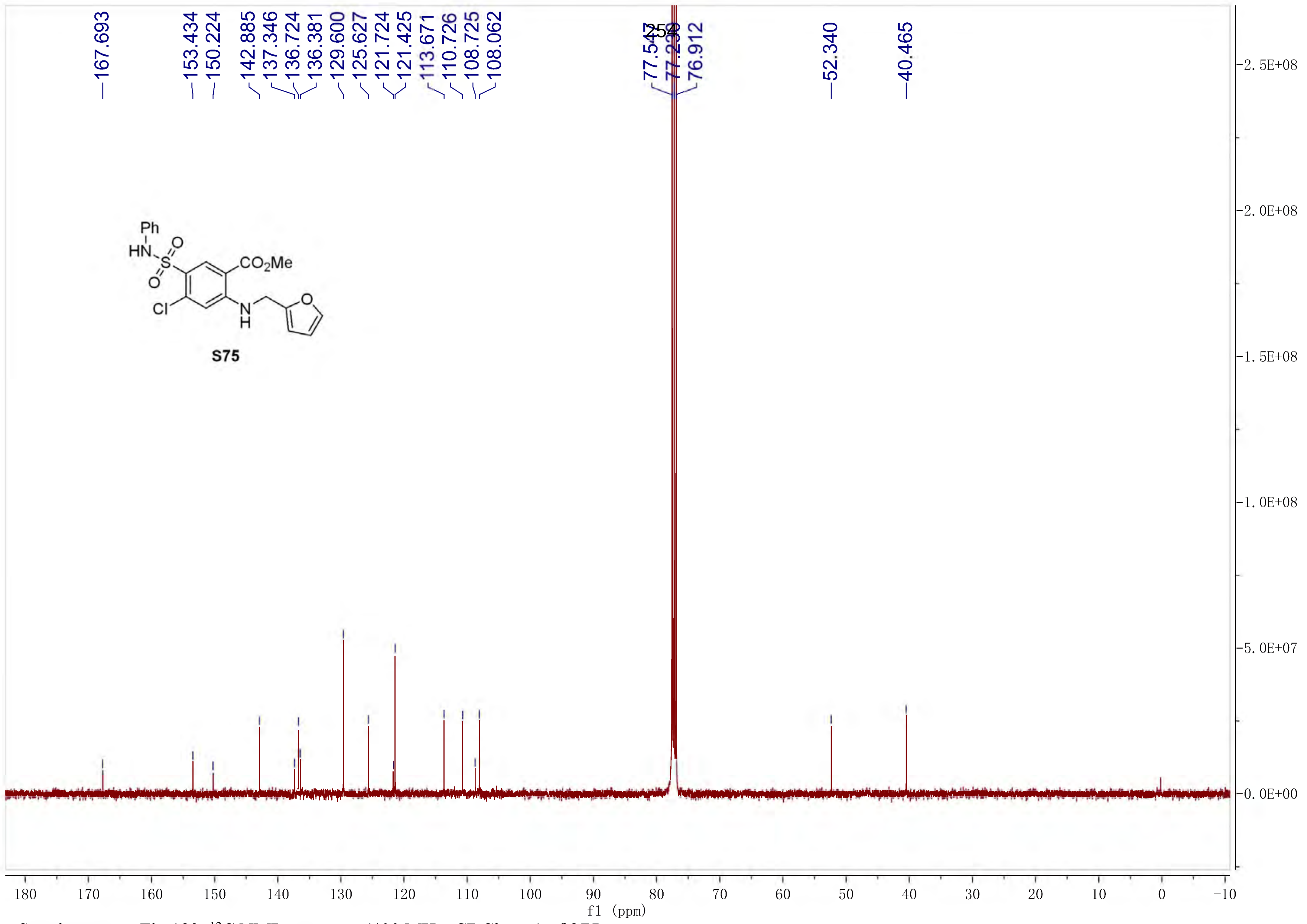

Supplementary Fig 182. ^{13}C NMR spectrum (400 MHz, $CDCl_3$, r.t.) of **S75**.

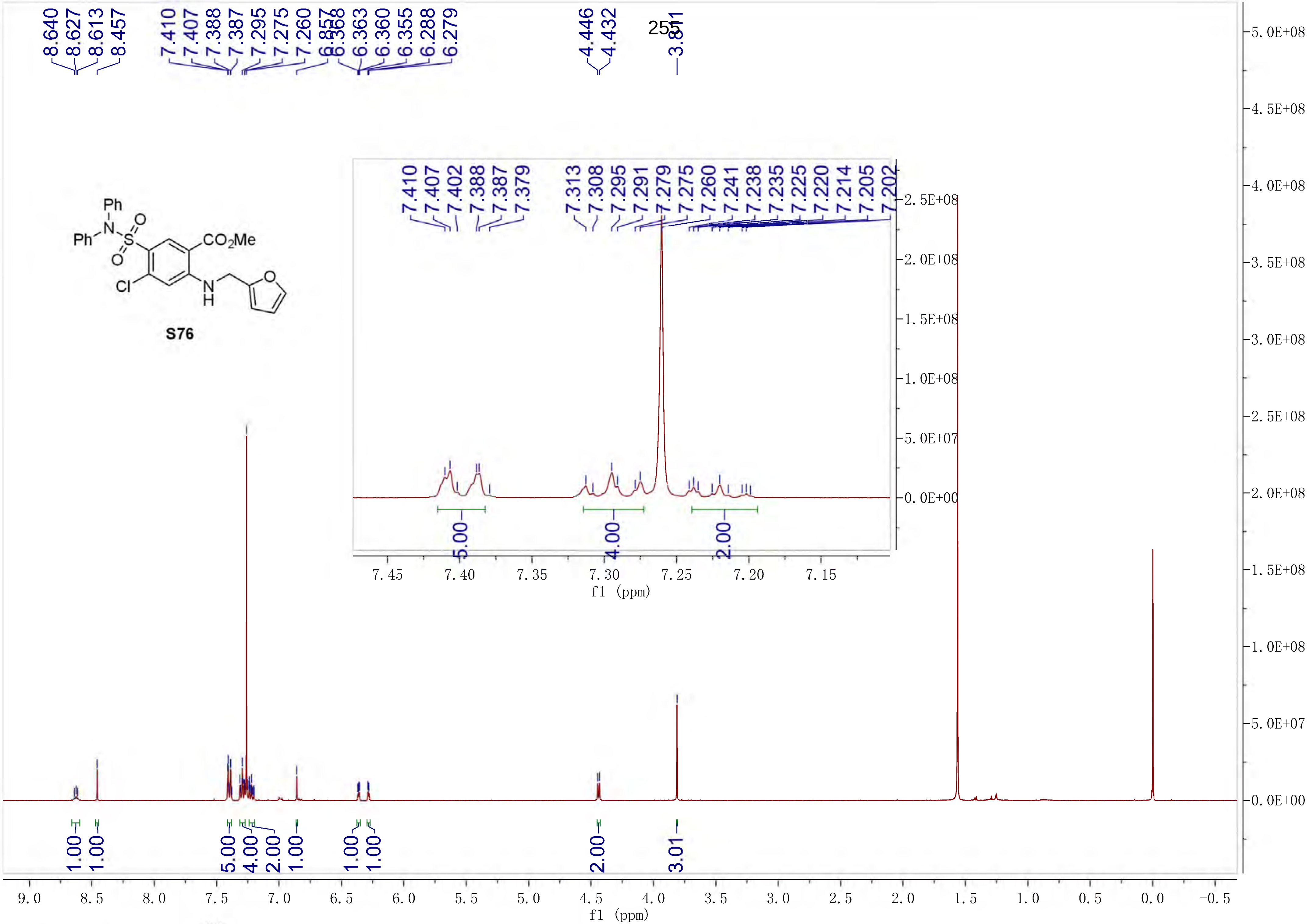

Supplementary Fig 183. ^{1}H NMR spectrum (400 MHz, $CDCl_3$, r.t.) of **S76**.

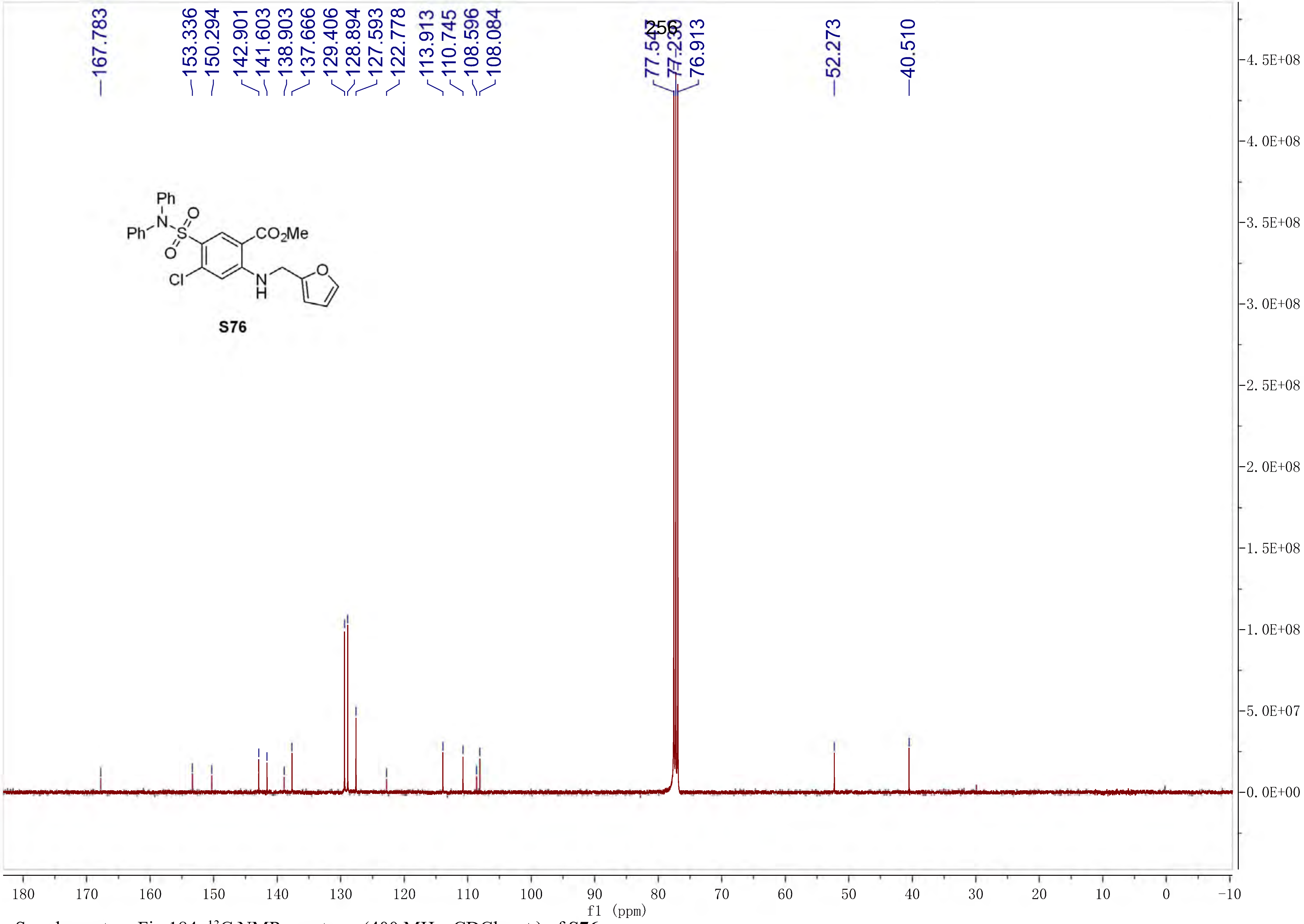

Supplementary Fig 184. ^{13}C NMR spectrum (400 MHz, $CDCl_3$, r.t.) of **S76**.

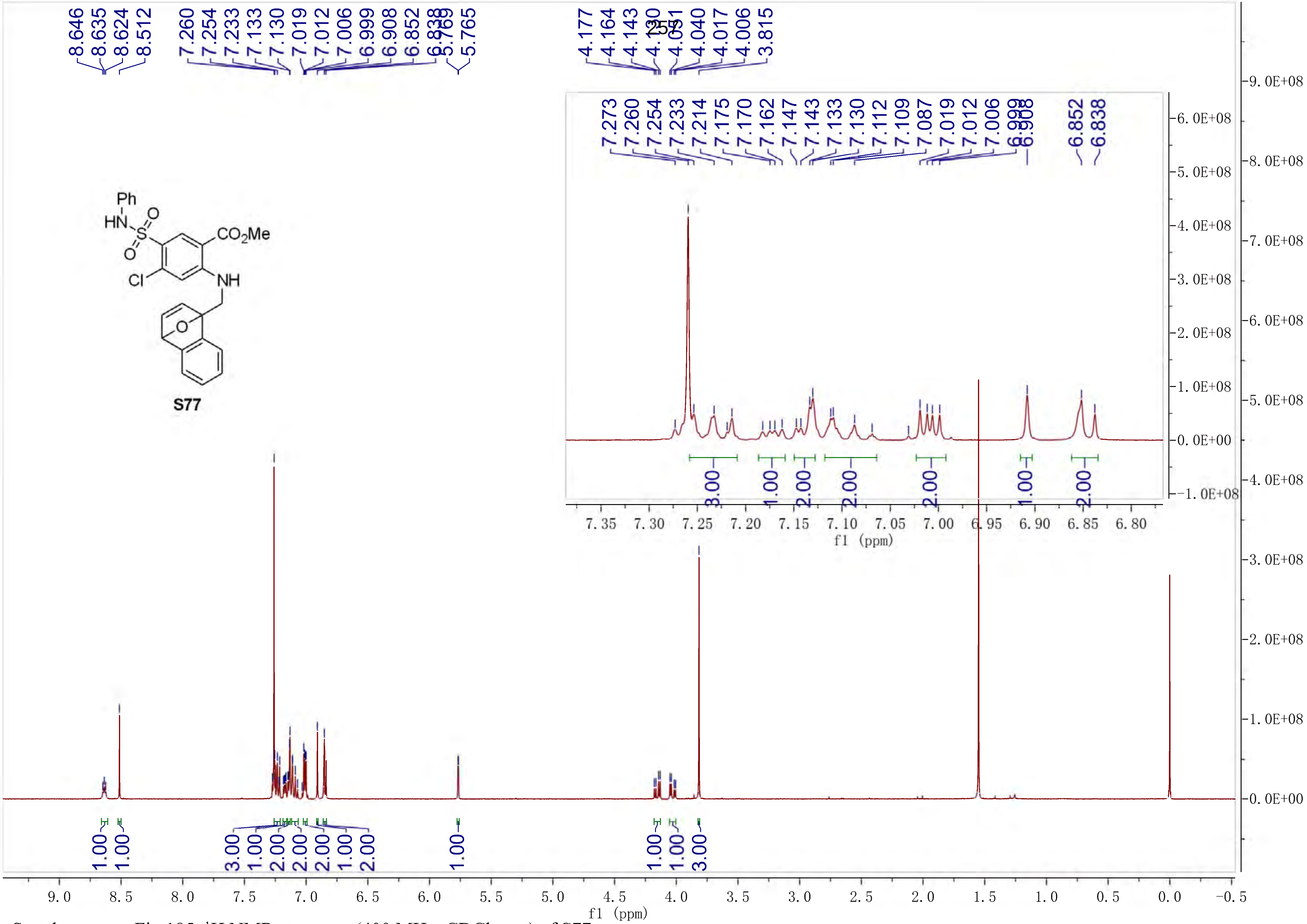

Supplementary Fig 185. ^{1}H NMR spectrum (400 MHz, $CDCl_3$, r.t.) of **S77**.

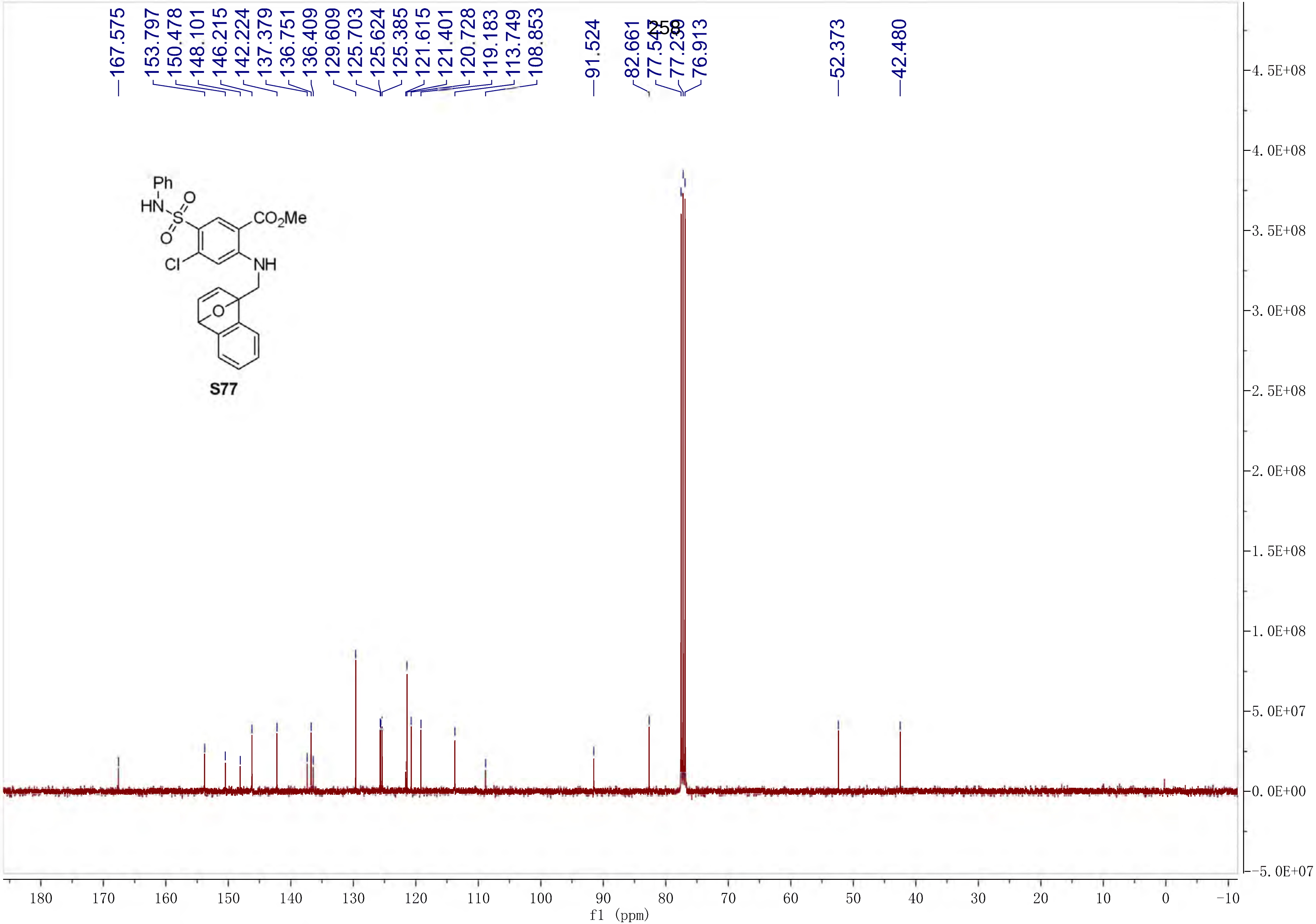

Supplementary Fig 186. ^{13}C NMR spectrum (400 MHz, $CDCl_3$, r.t.) of **S77**.

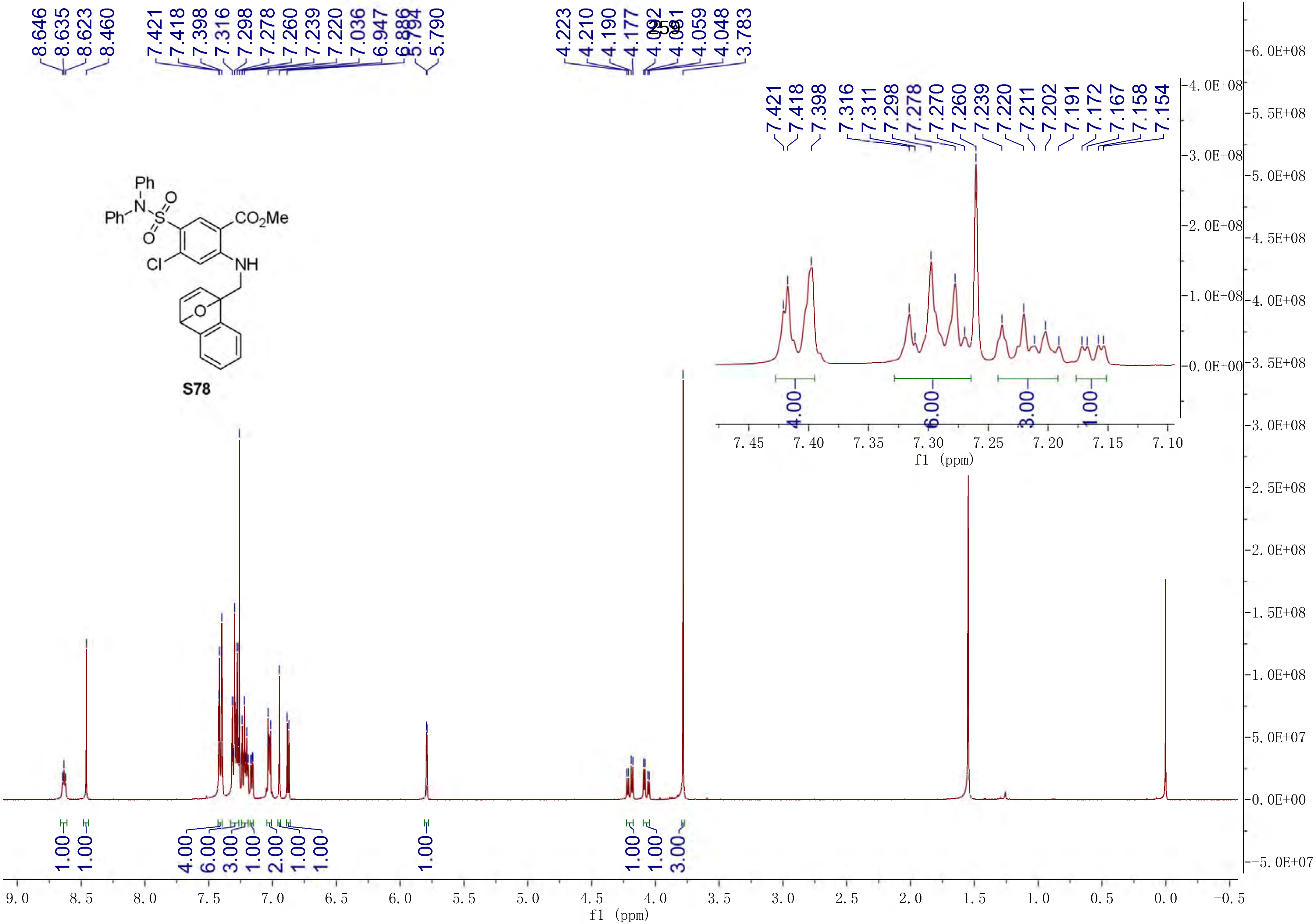

Supplementary Fig 187. ^{1}H NMR spectrum (400 MHz, $CDCl_3$, r.t.) of **S78**.

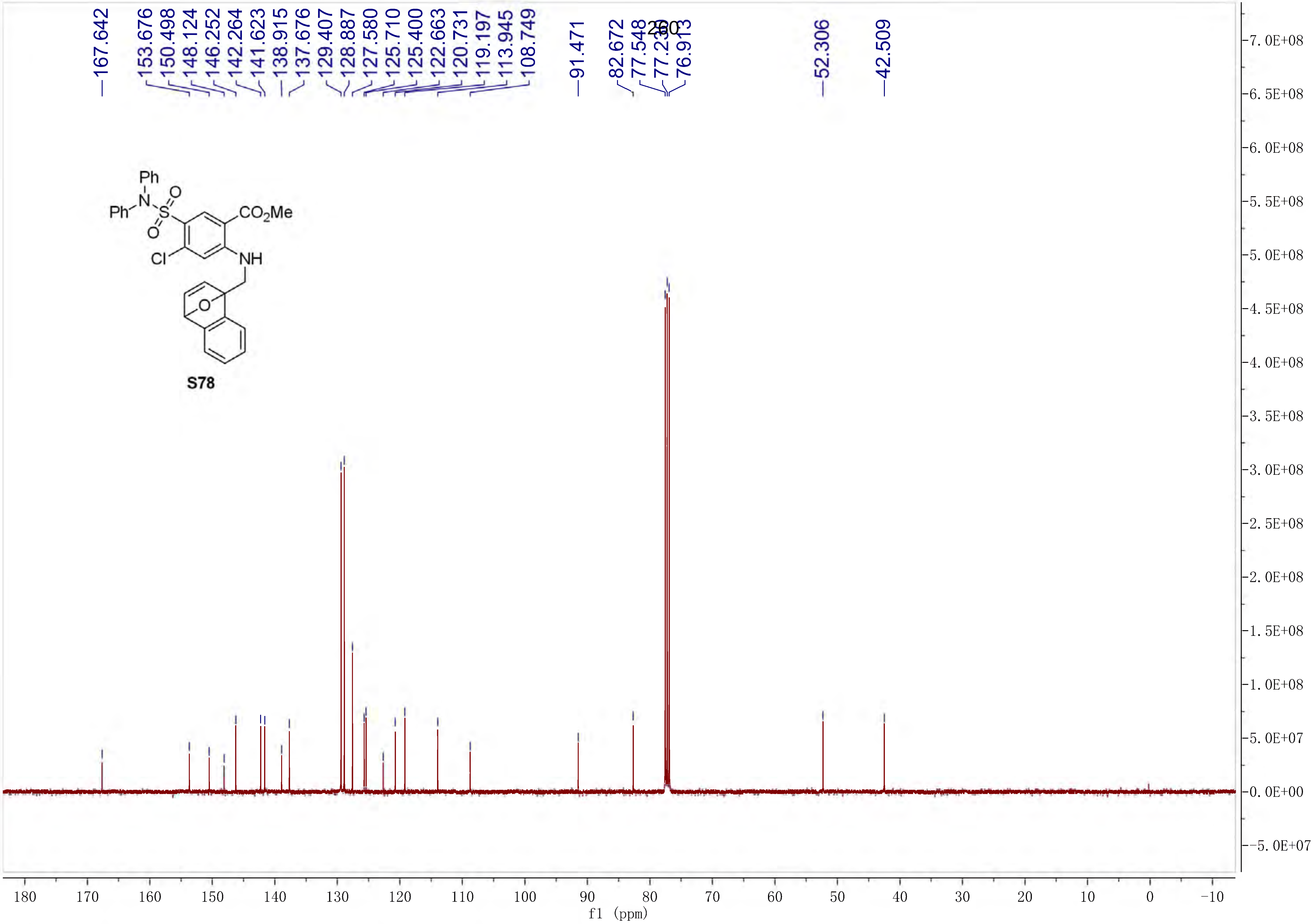

Supplementary Fig 188. ^{13}C NMR spectrum (400 MHz, $CDCl_3$, r.t.) of **S78**.

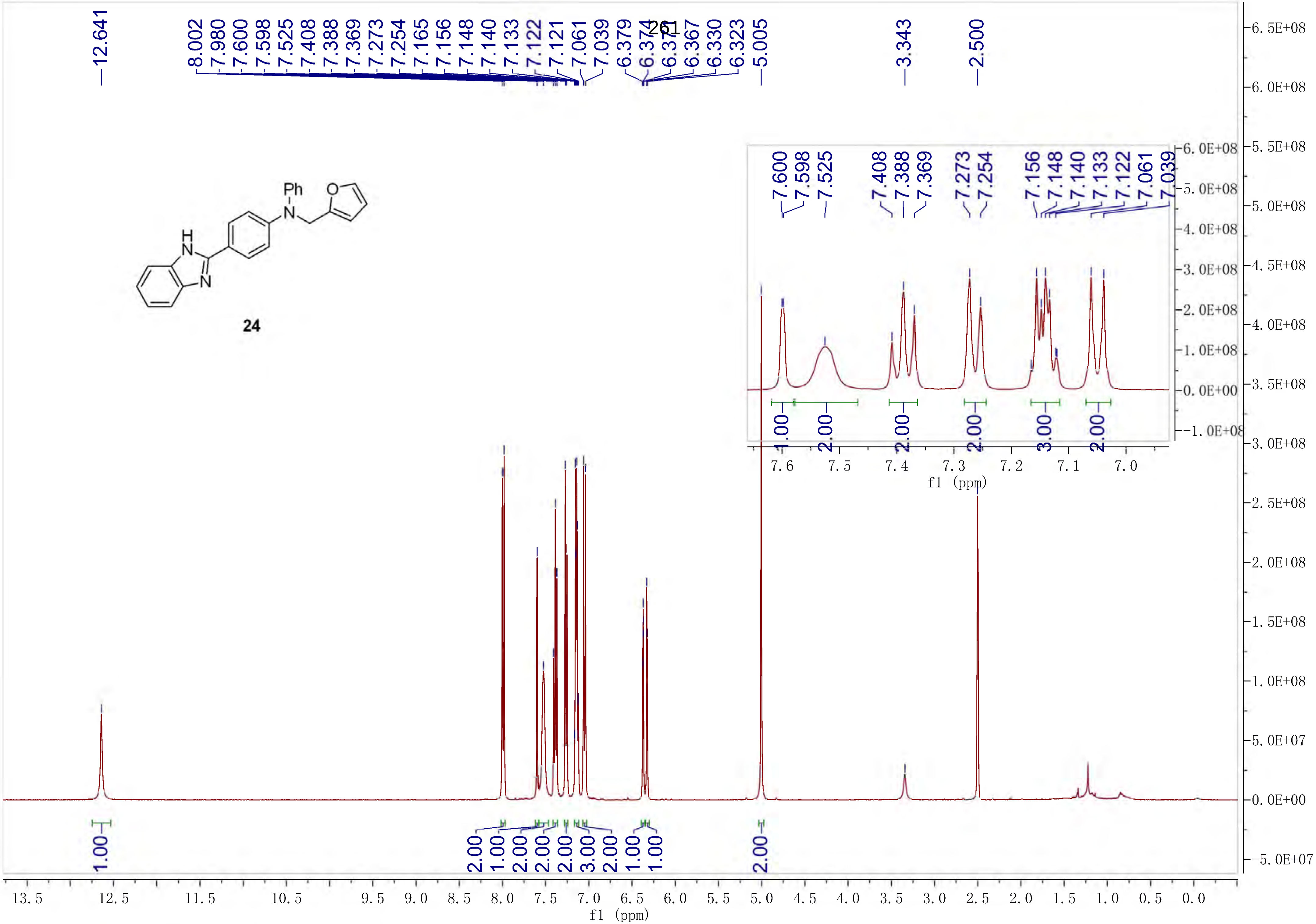

Supplementary Fig 189. ^{1}H NMR spectrum (400 MHz, $CDCl_3$, r.t.) of **24**.

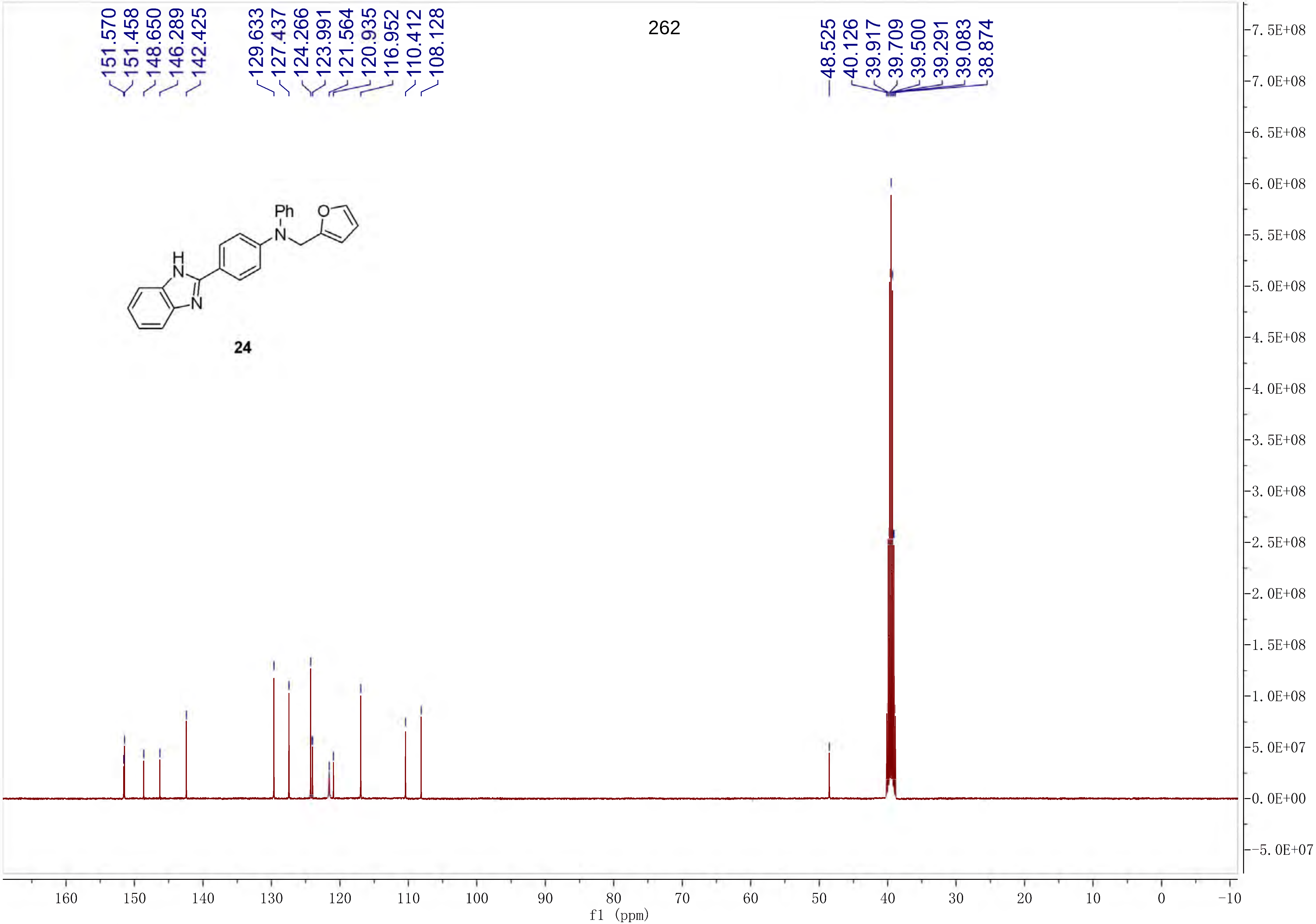

Supplementary Fig 190. ^{13}C NMR spectrum (400 MHz, $CDCl_3$, r.t.) of **24**.

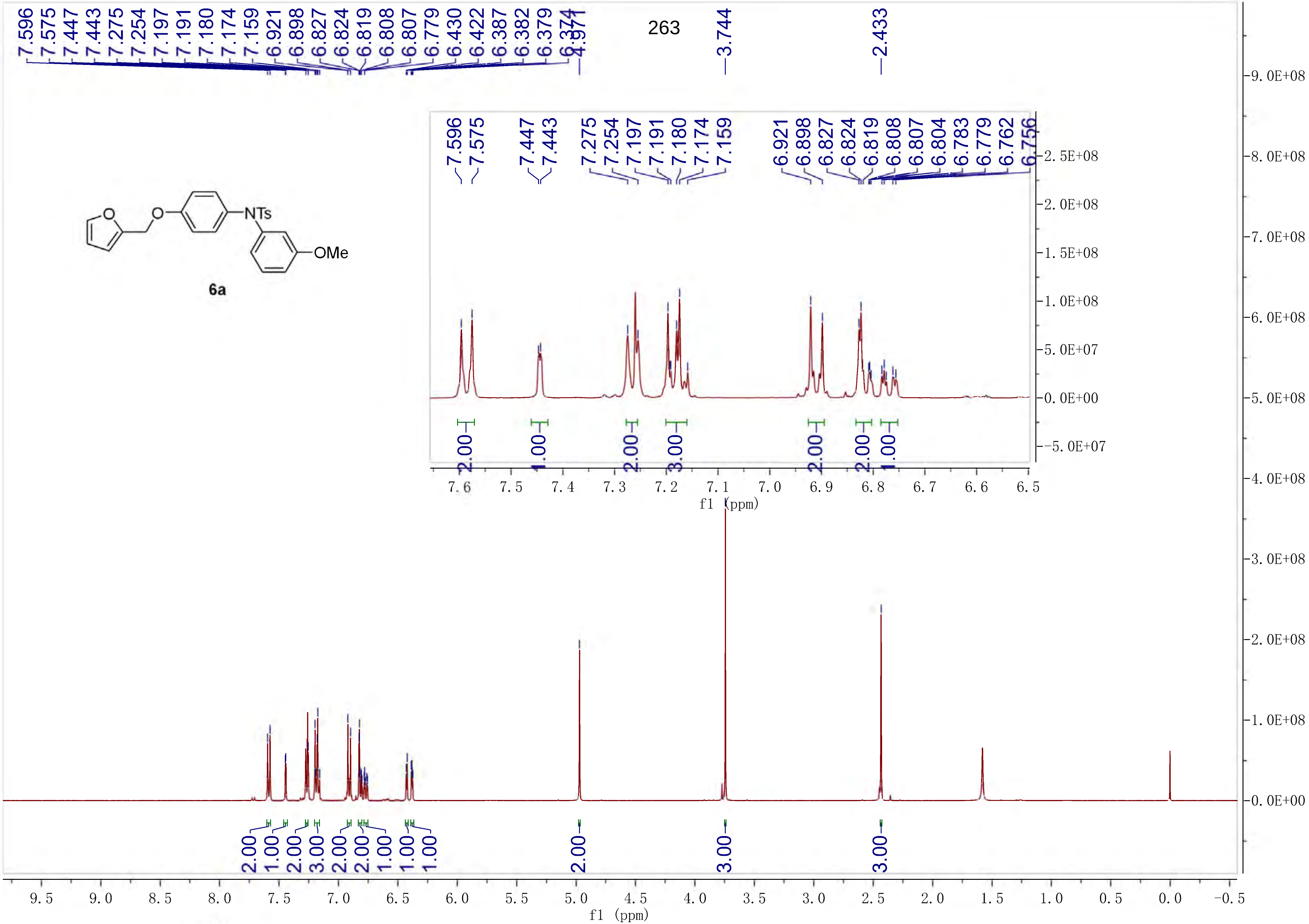

Supplementary Fig 191. ^{1}H NMR spectrum (400 MHz, $CDCl_3$, r.t.) of **6a**.

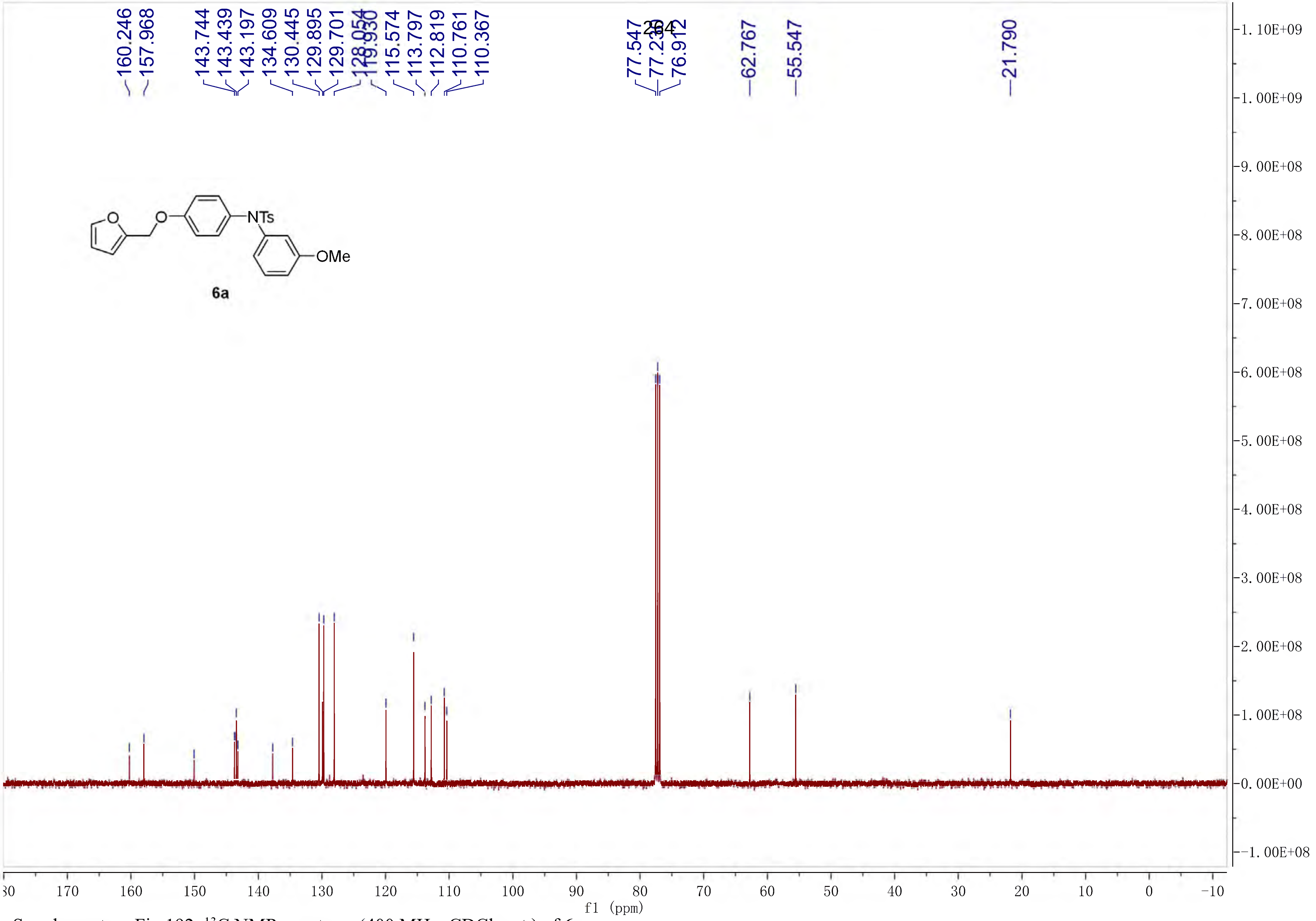

Supplementary Fig 192. ^{13}C NMR spectrum (400 MHz, $CDCl_3$, r.t.) of **6a**.

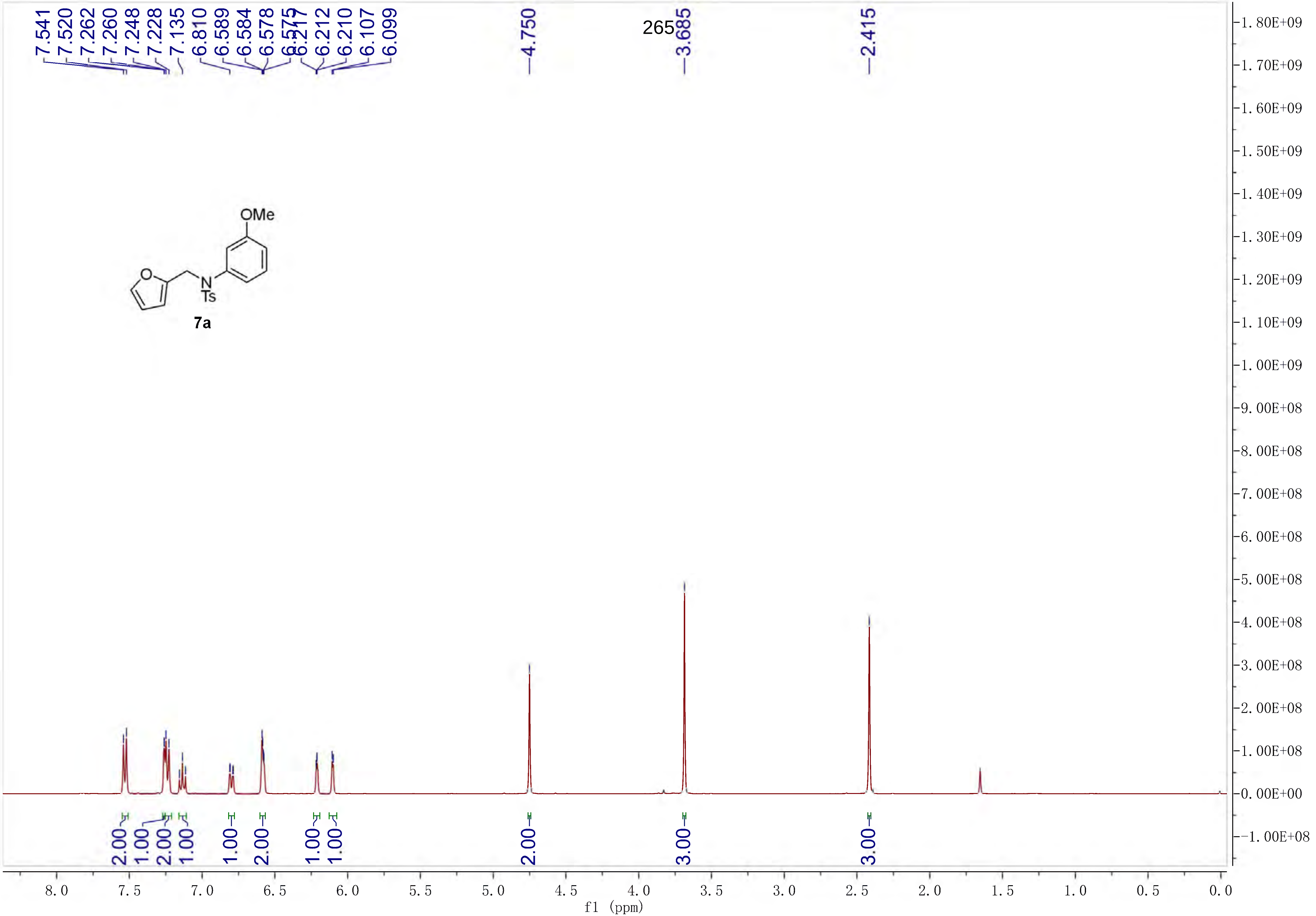

7a

Supplementary Fig 193. ^{1}H NMR spectrum (400 MHz, $CDCl_3$, r.t.) of **7a**.

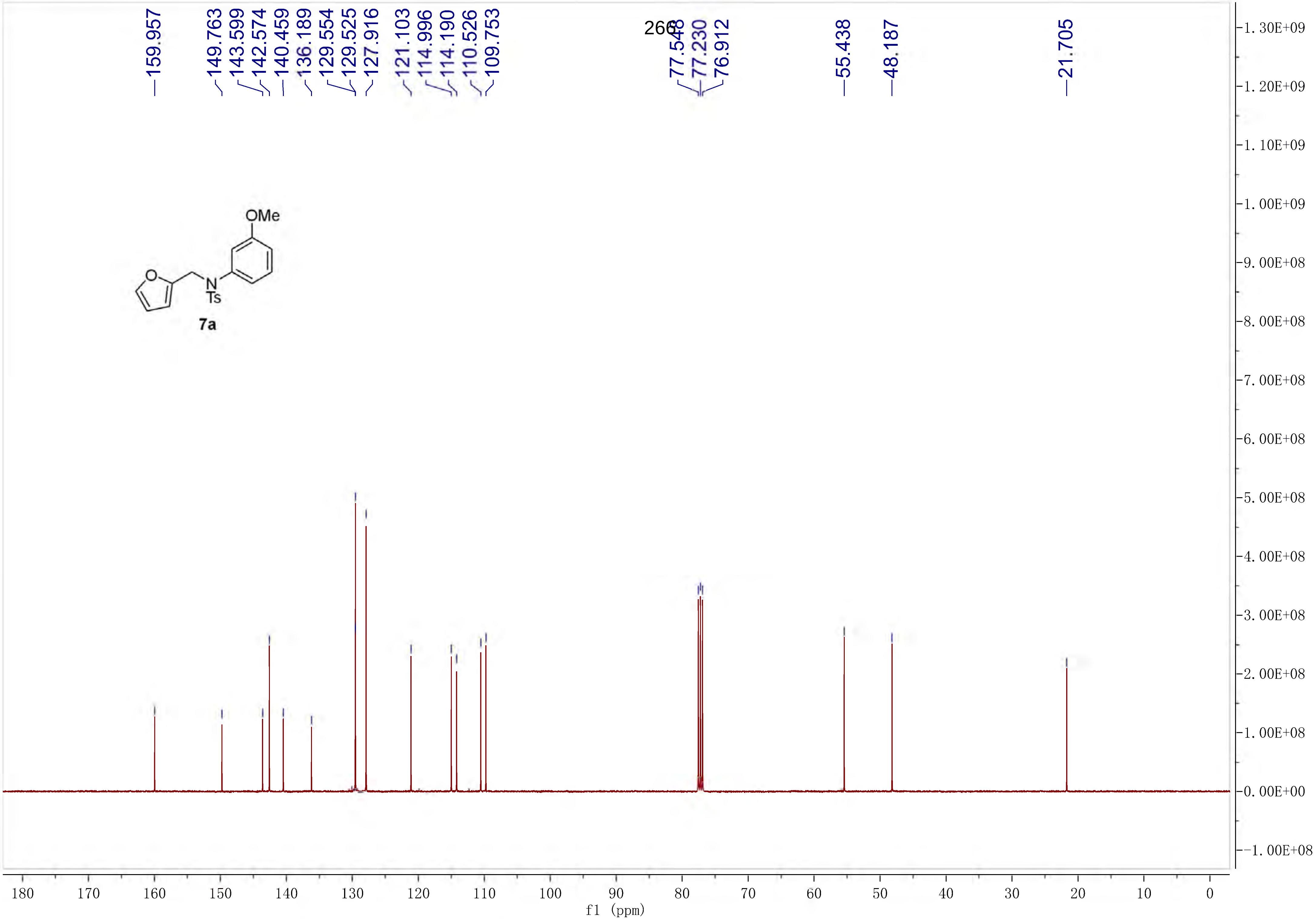

Supplementary Fig 194. ^{13}C NMR spectrum (400 MHz, $CDCl_3$, r.t.) of **7a**.

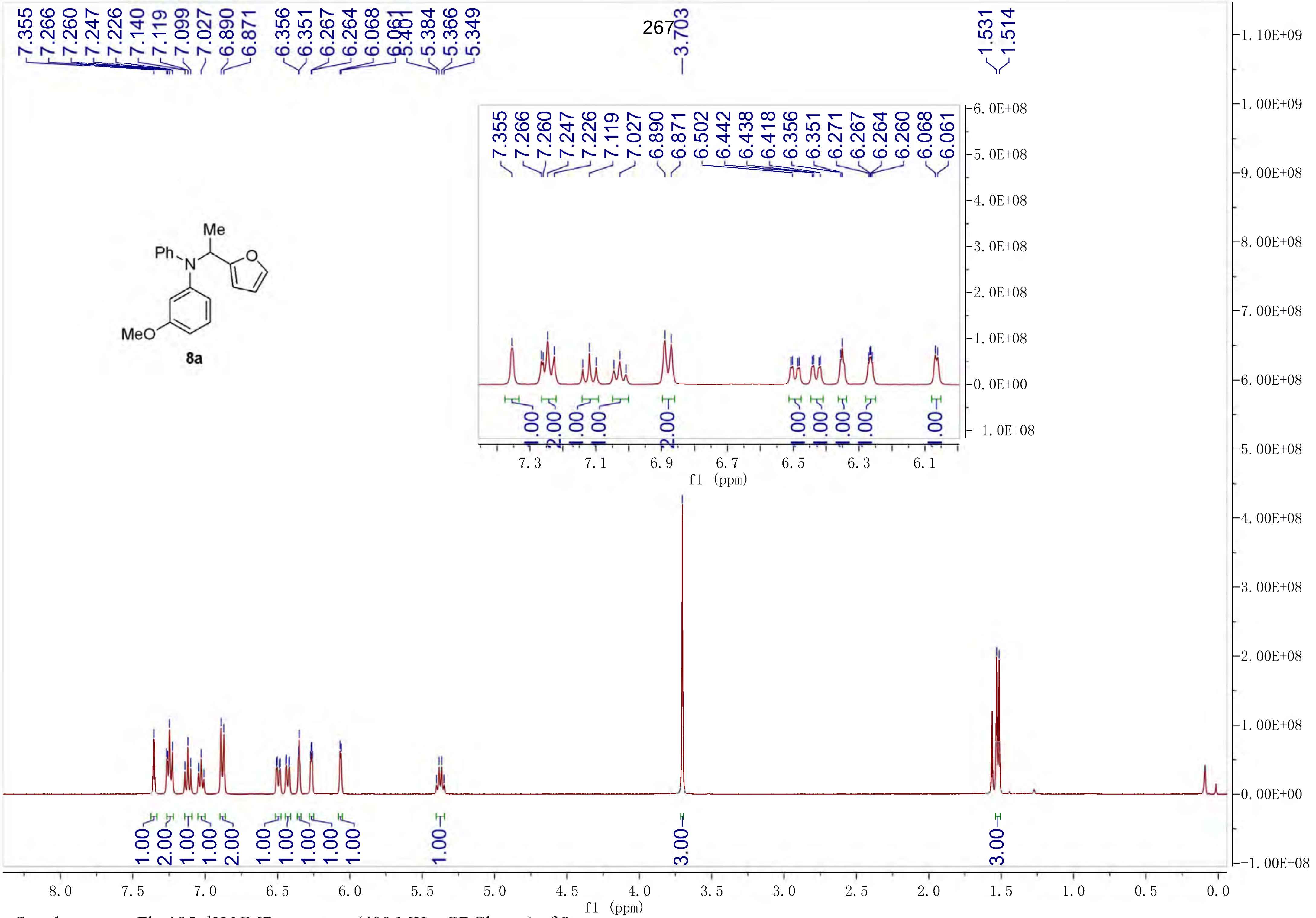

Supplementary Fig 195. ^{1}H NMR spectrum (400 MHz, $CDCl_3$, r.t.) of **8a**.

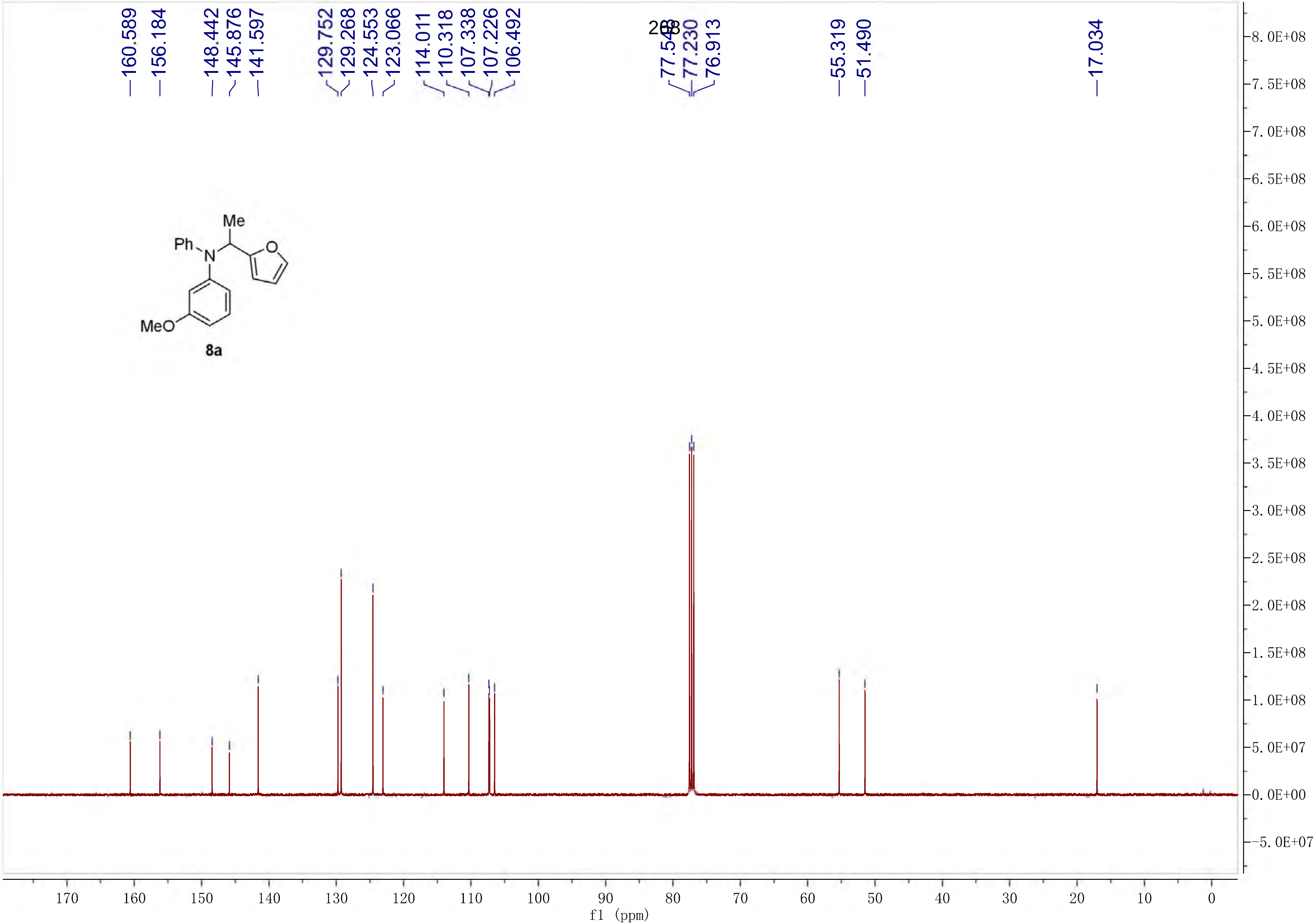

Supplementary Fig 196. ^{13}C NMR spectrum (400 MHz, $CDCl_3$, r.t.) of **8a**.

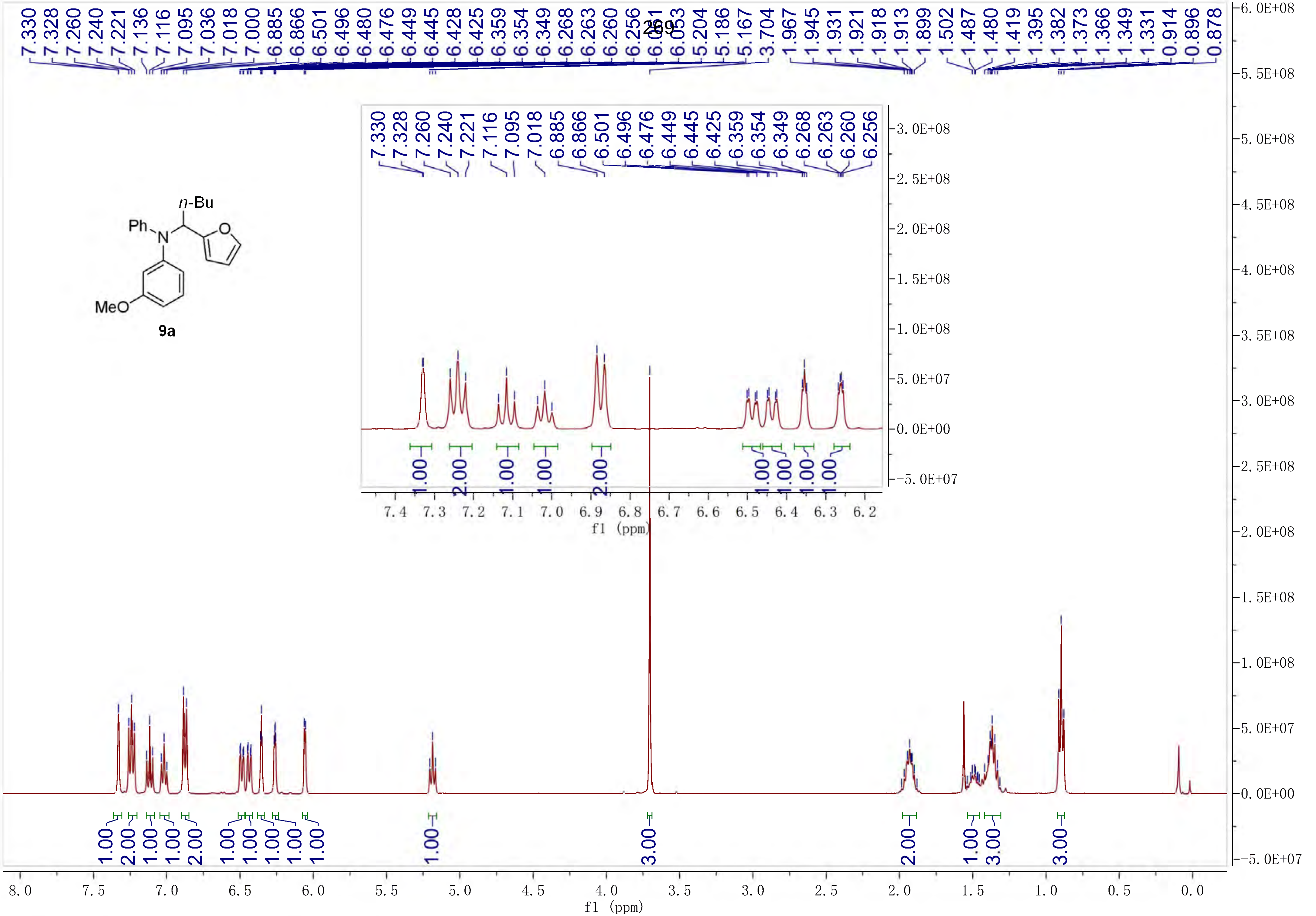

Supplementary Fig 197. ^{1}H NMR spectrum (400 MHz, $CDCl_3$, r.t.) of **9a**.

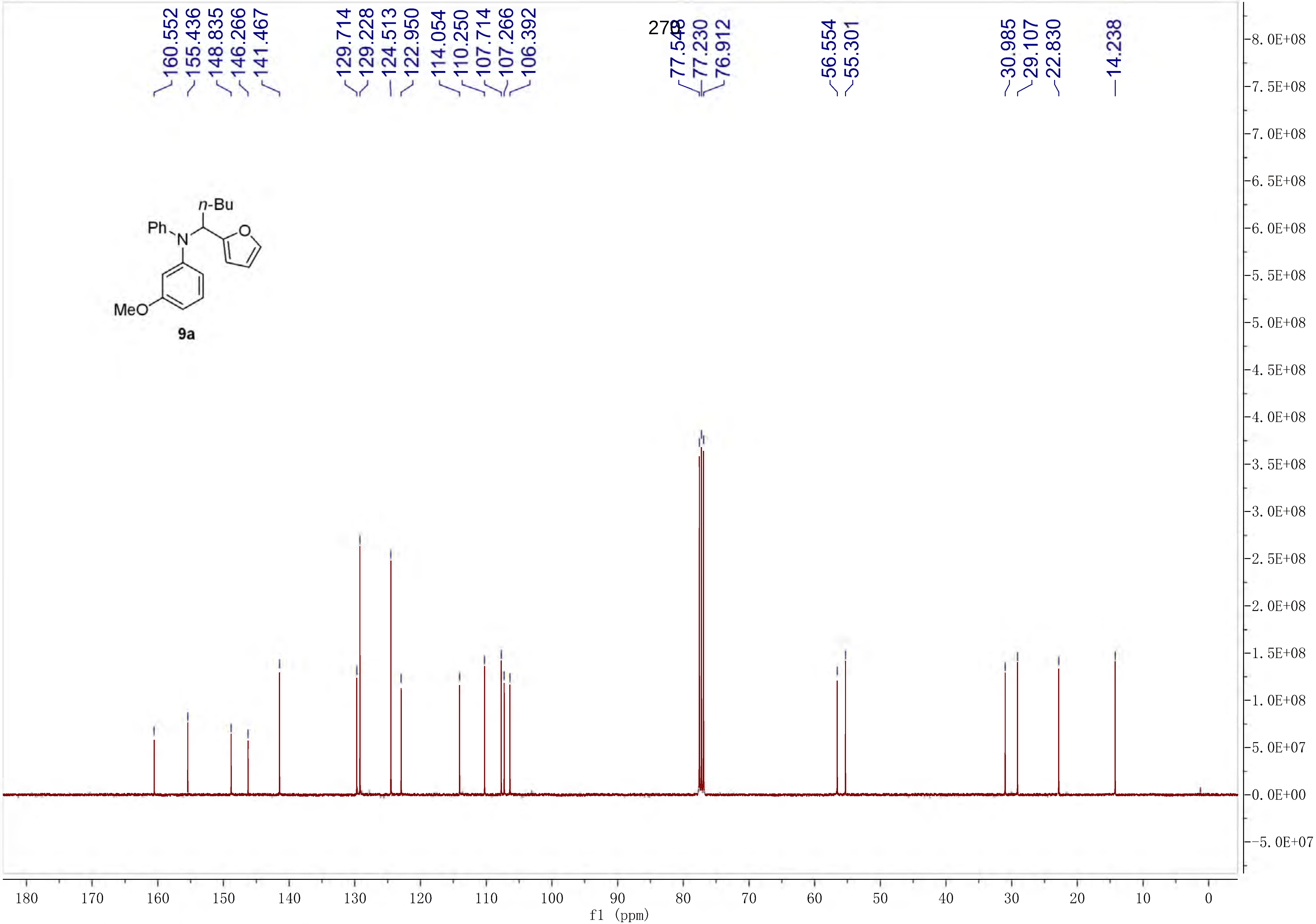

Supplementary Fig 198. ^{13}C NMR spectrum (400 MHz, $CDCl_3$, r.t.) of **9a**.

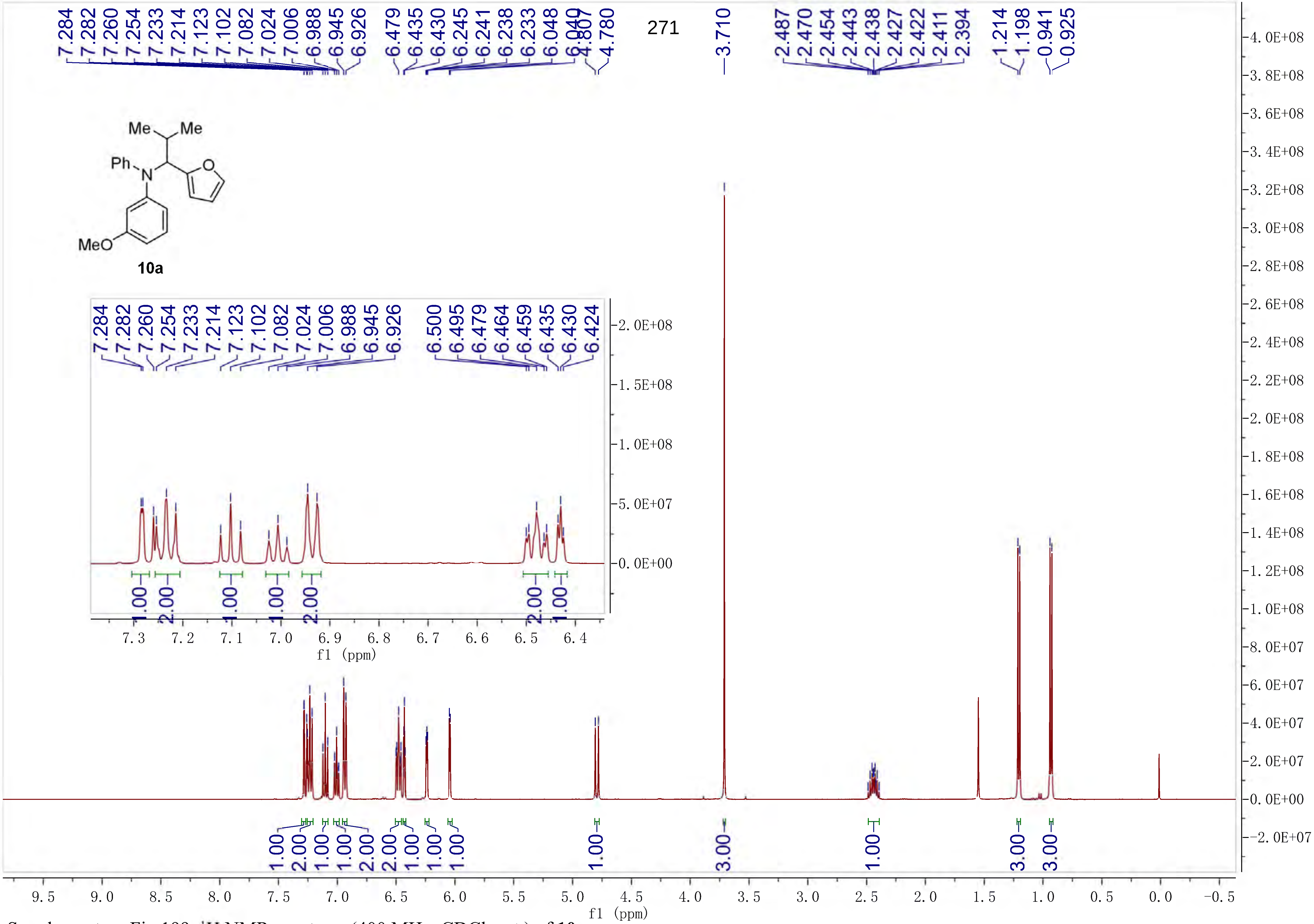

Supplementary Fig 199. ^{1}H NMR spectrum (400 MHz, $CDCl_3$, r.t.) of **10a**.

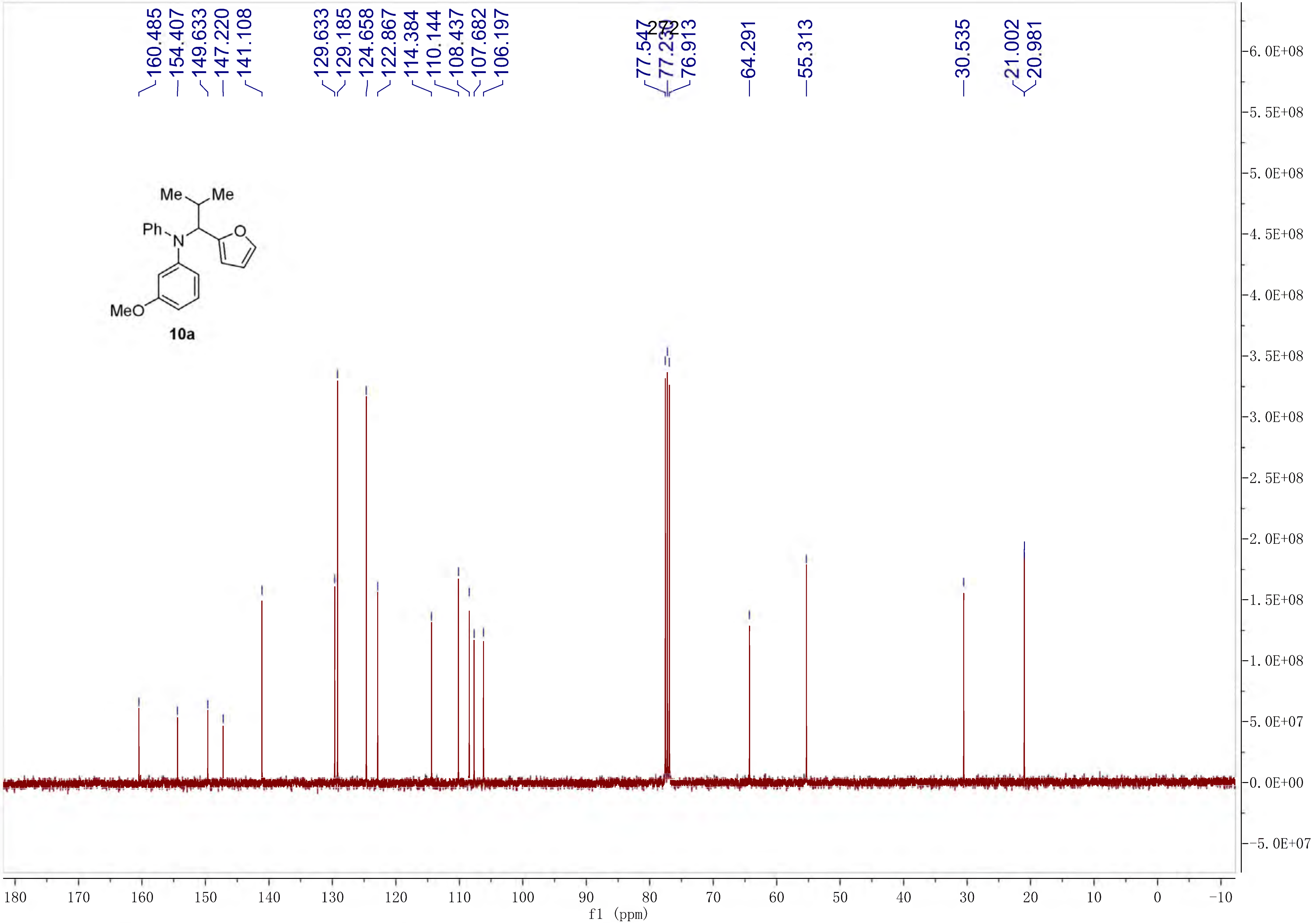

Supplementary Fig 200. ^{13}C NMR spectrum (400 MHz, $CDCl_3$, r.t.) of **10a**.

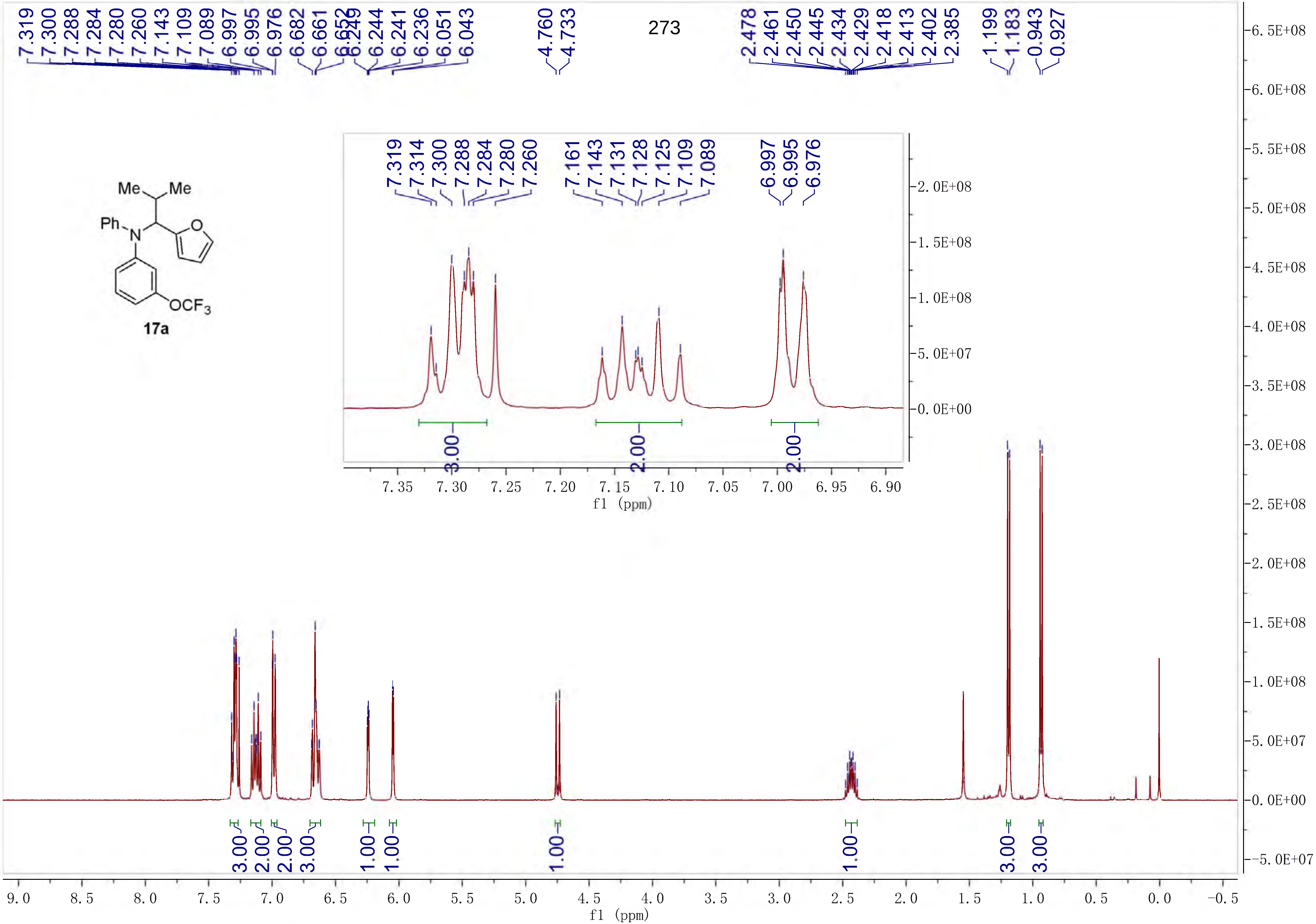

Supplementary Fig 201. ^{1}H NMR spectrum (400 MHz, $CDCl_3$, r.t.) of **17a**.

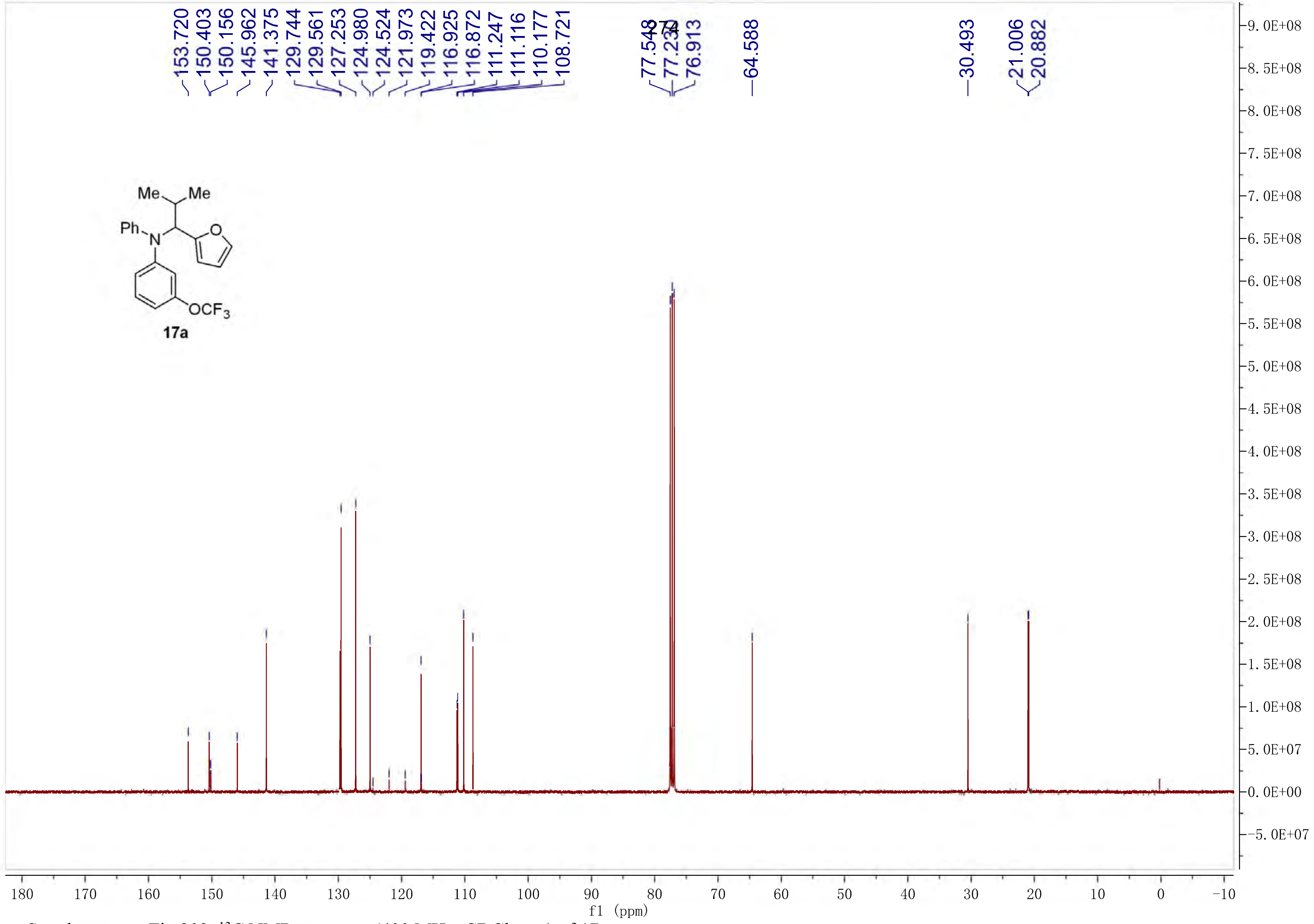

Supplementary Fig 202. ^{13}C NMR spectrum (400 MHz, $CDCl_3$, r.t.) of **17a**.

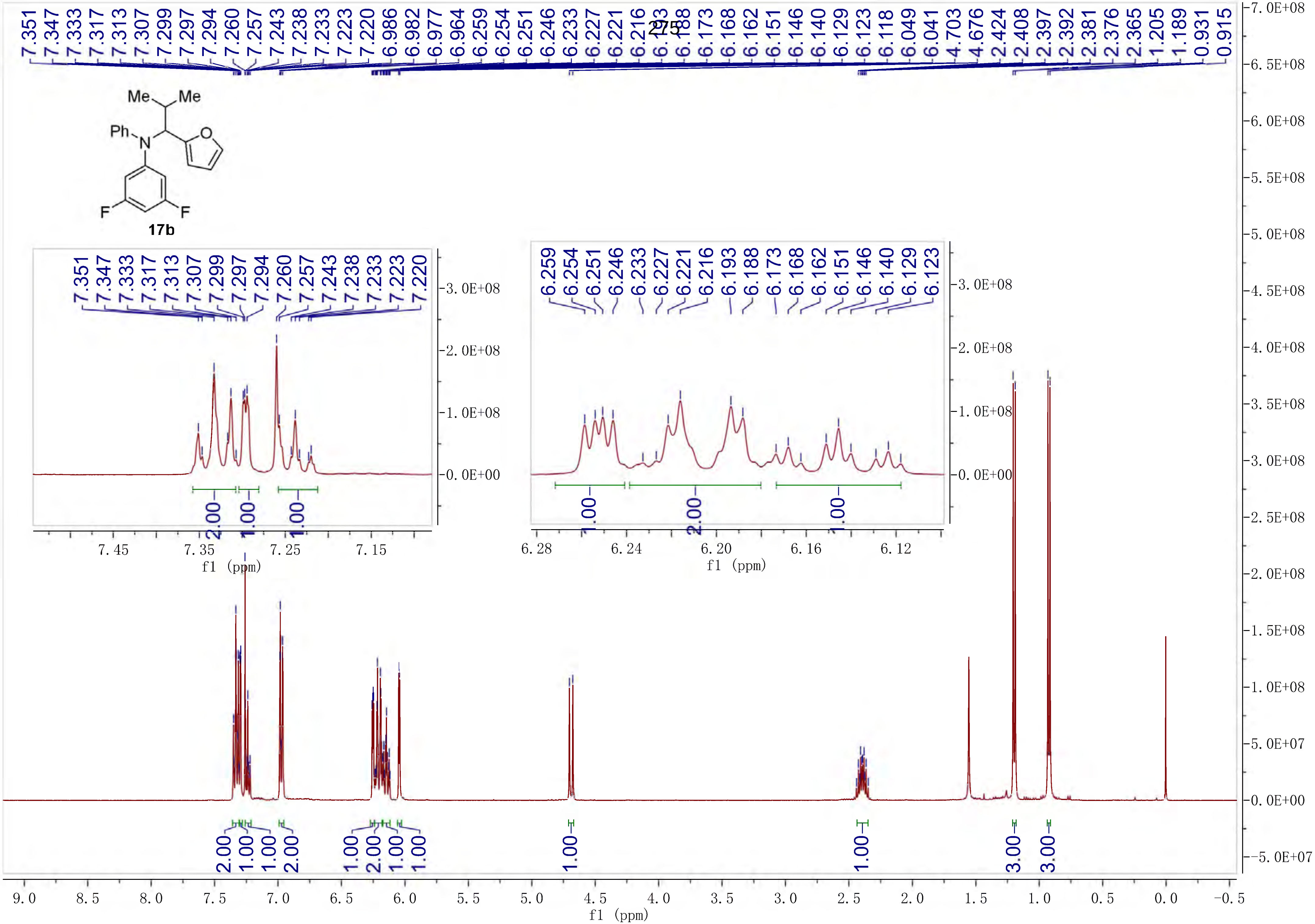

Supplementary Fig 203. ^{1}H NMR spectrum (400 MHz, $CDCl_3$, r.t.) of **17b**.

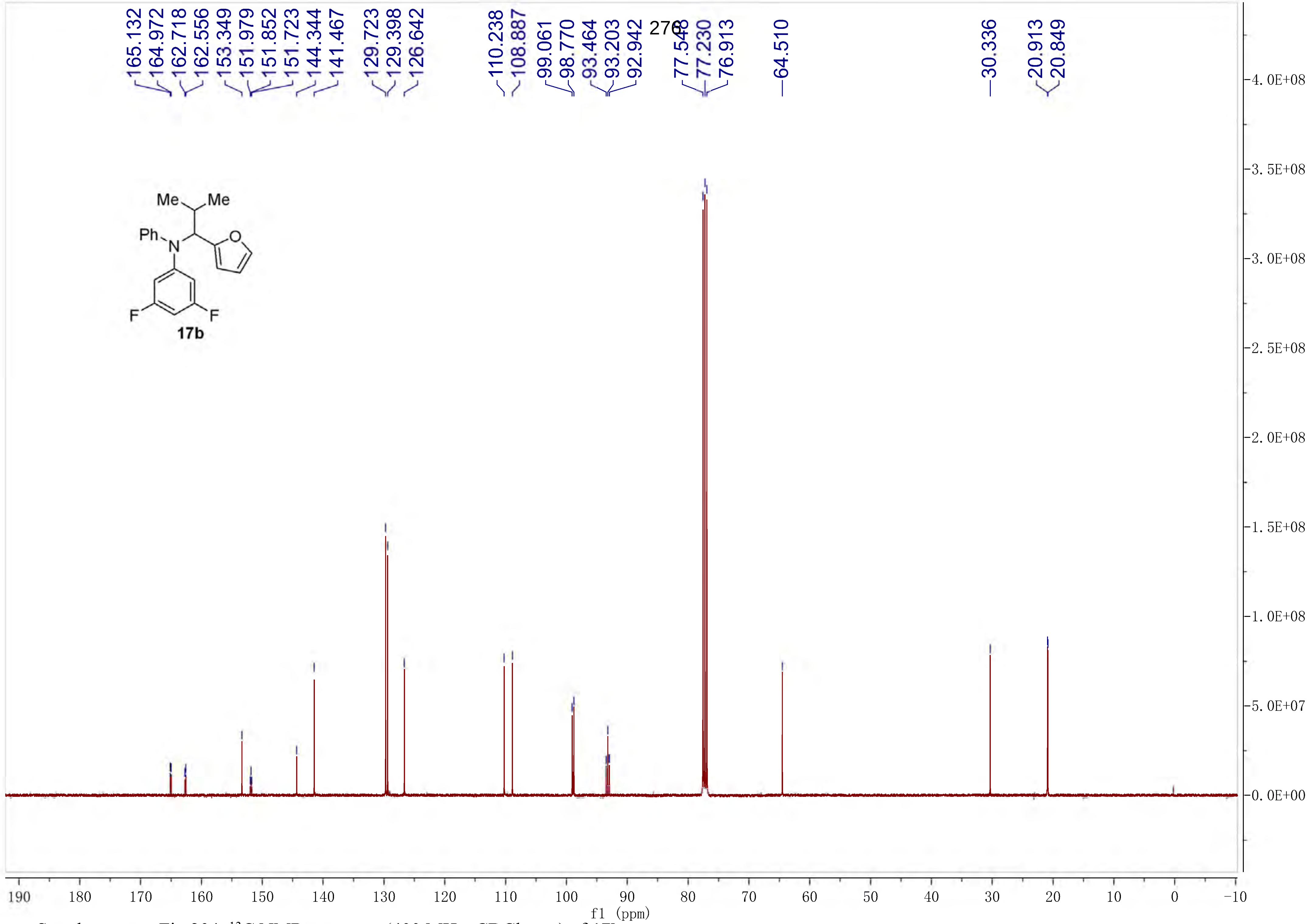

Supplementary Fig 204. ^{13}C NMR spectrum (400 MHz, $CDCl_3$, r.t.) of **17b**.

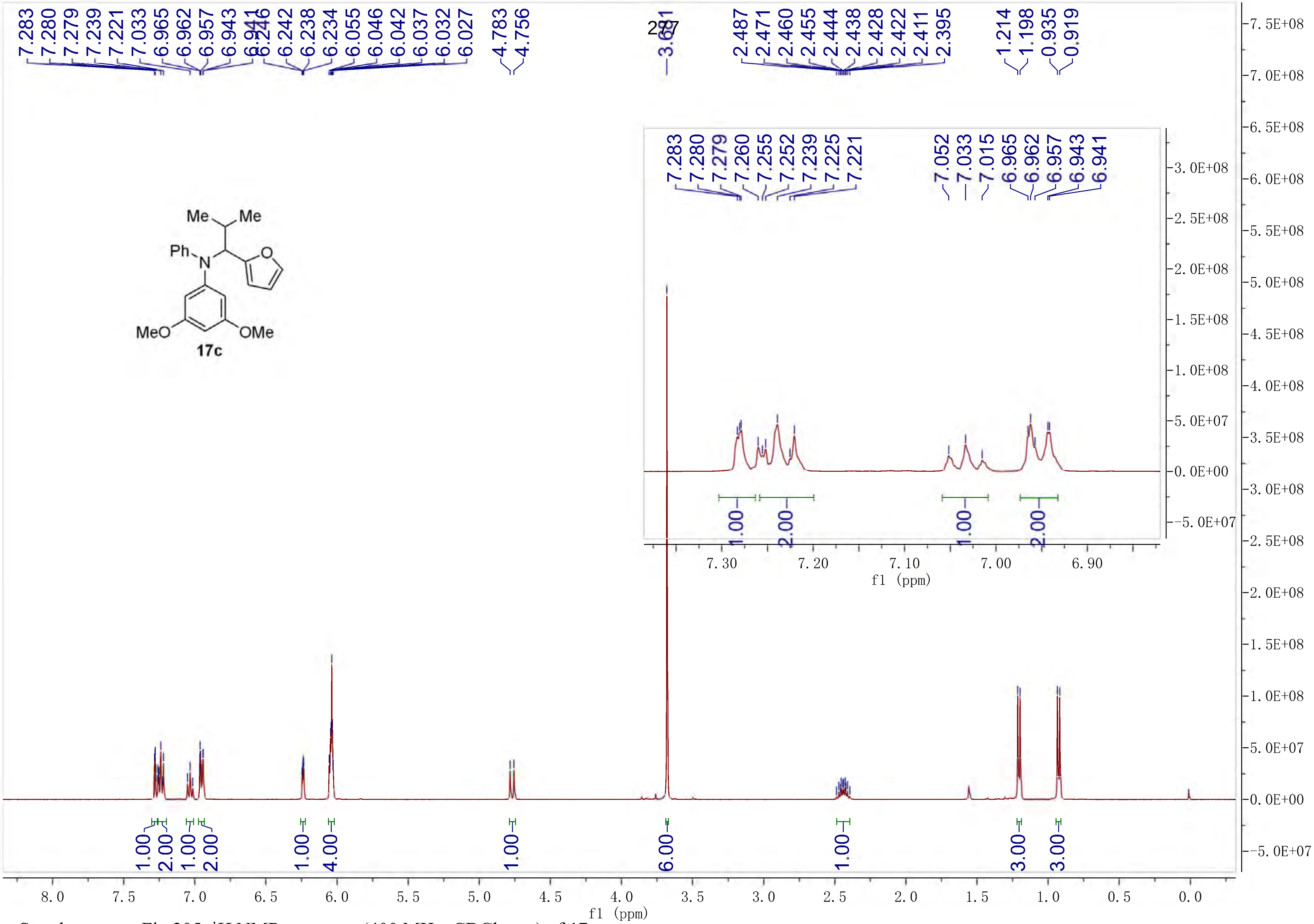

Supplementary Fig 205. ^{1}H NMR spectrum (400 MHz, $CDCl_3$, r.t.) of **17c**.

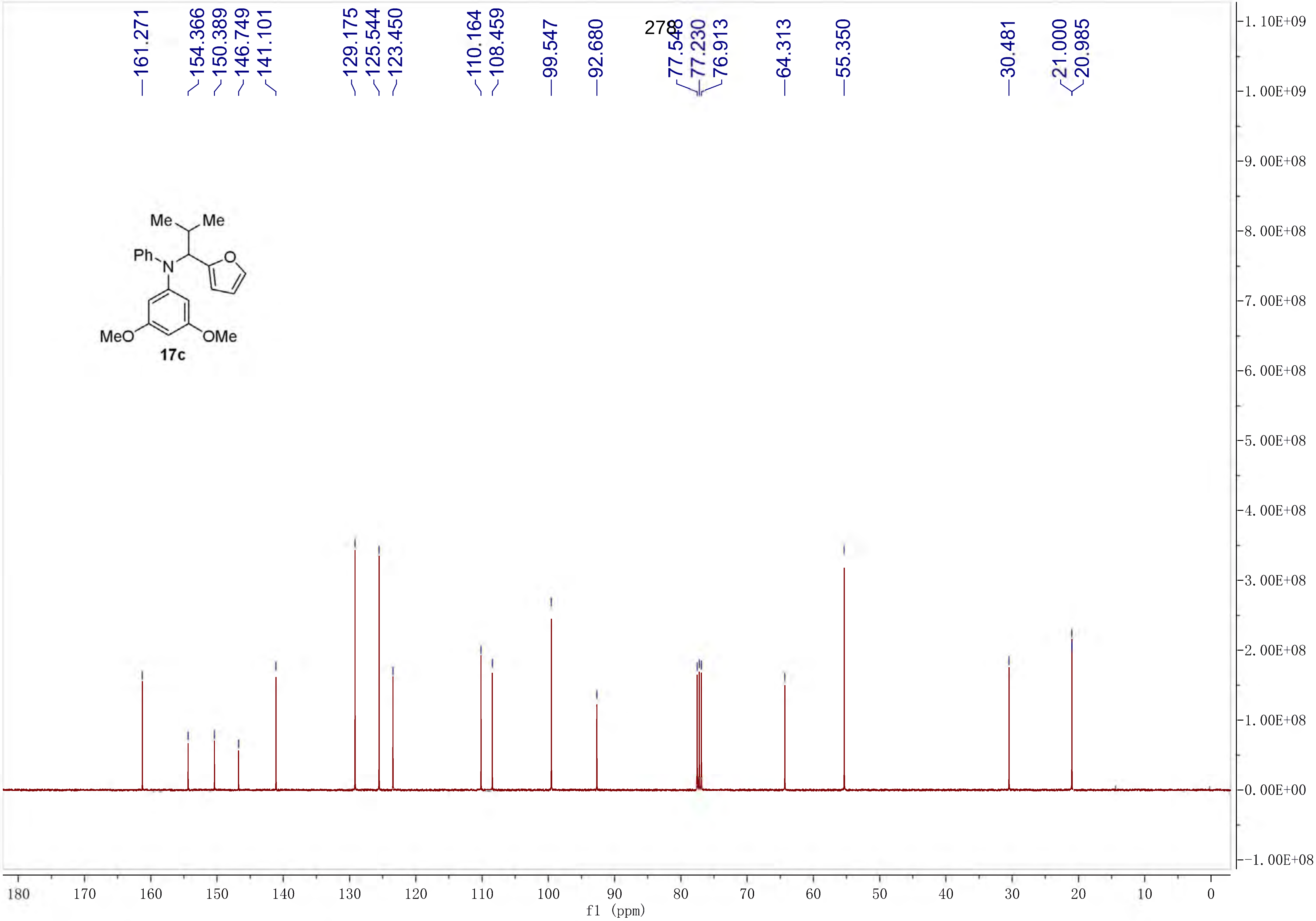

Supplementary Fig 206. ^{13}C NMR spectrum (400 MHz, $CDCl_3$, r.t.) of **17c**.

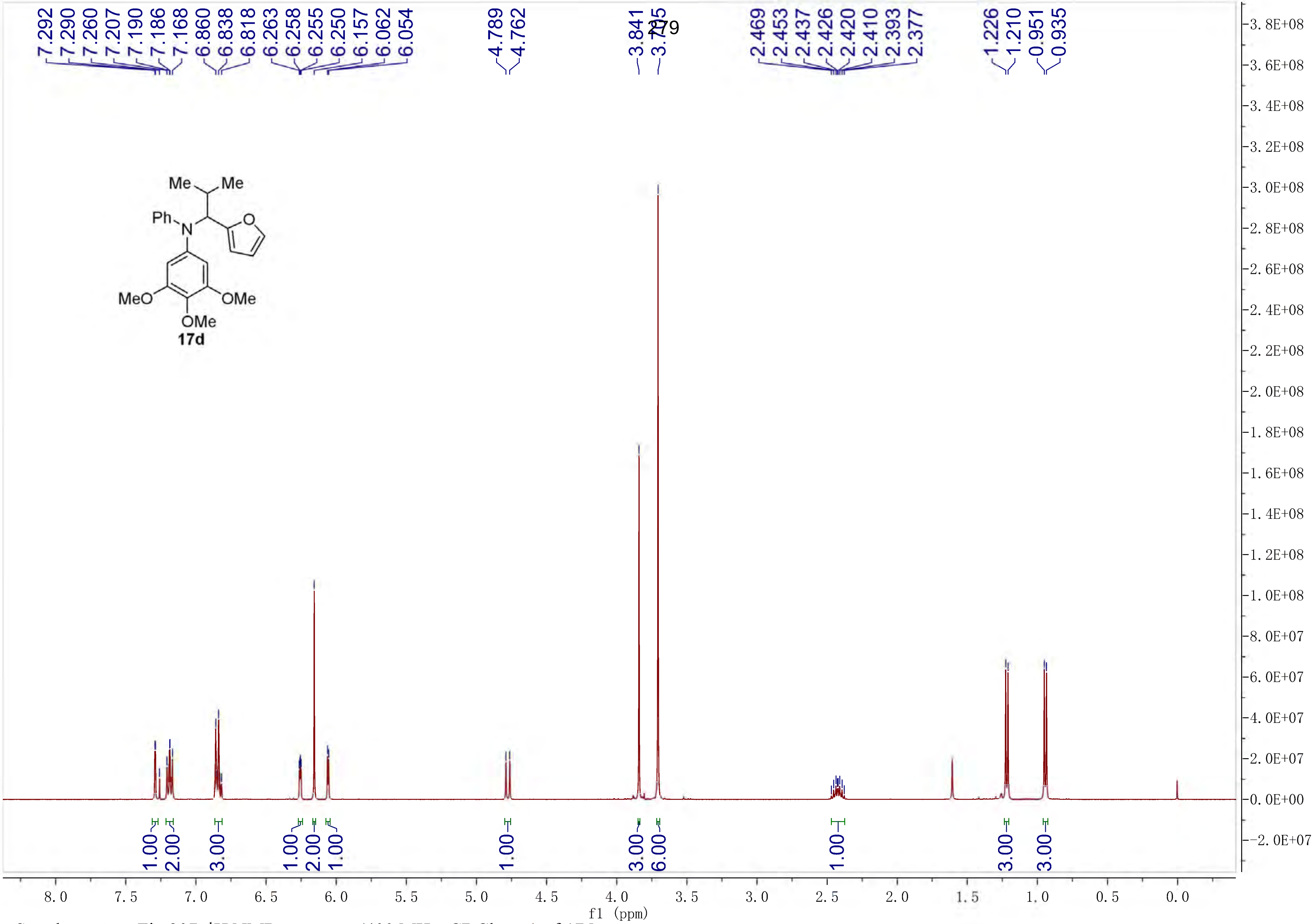

Supplementary Fig 207. ^{1}H NMR spectrum (400 MHz, $CDCl_3$, r.t.) of **17d**.

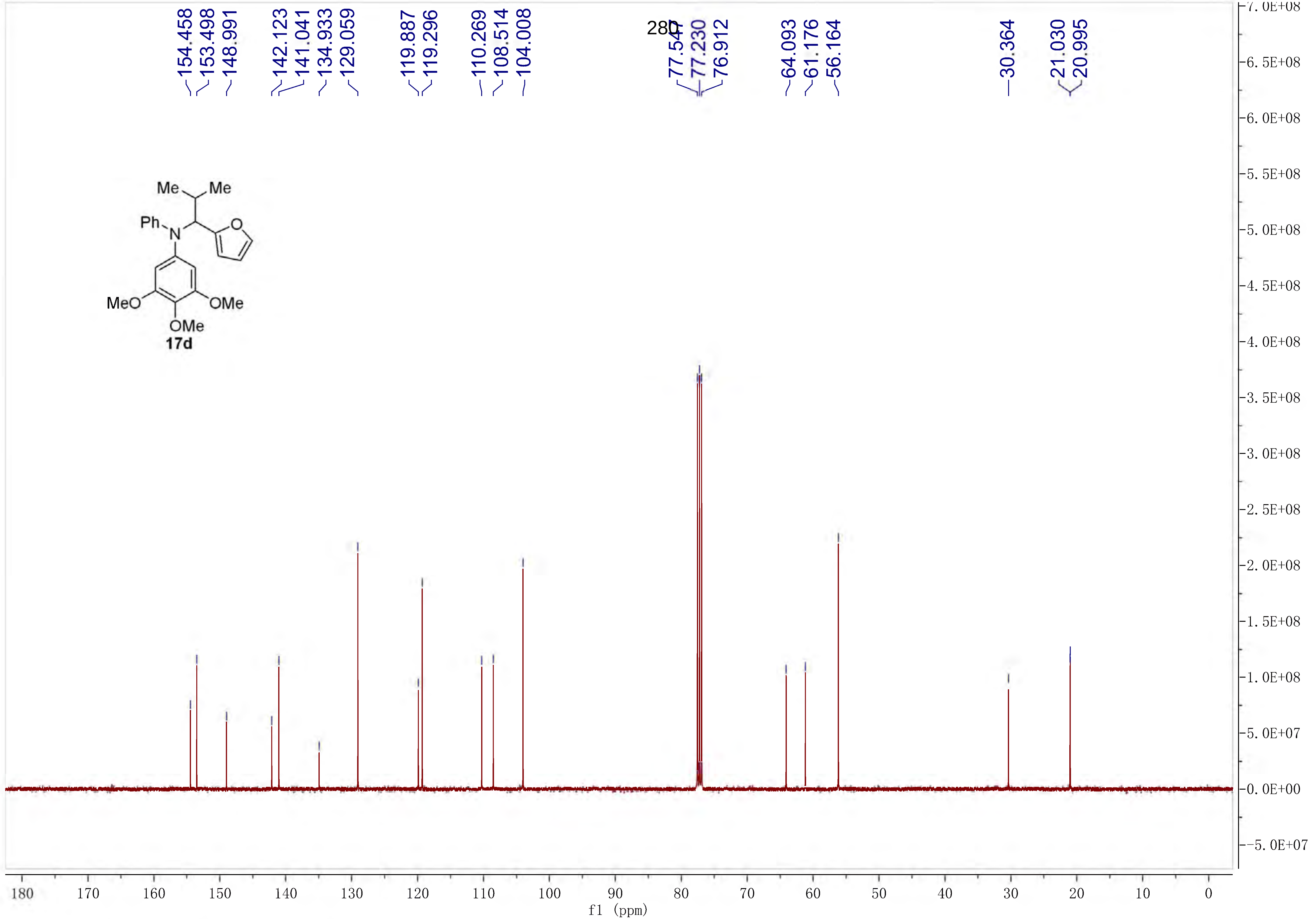

Supplementary Fig 208. ^{13}C NMR spectrum (400 MHz, $CDCl_3$, r.t.) of **17d**.

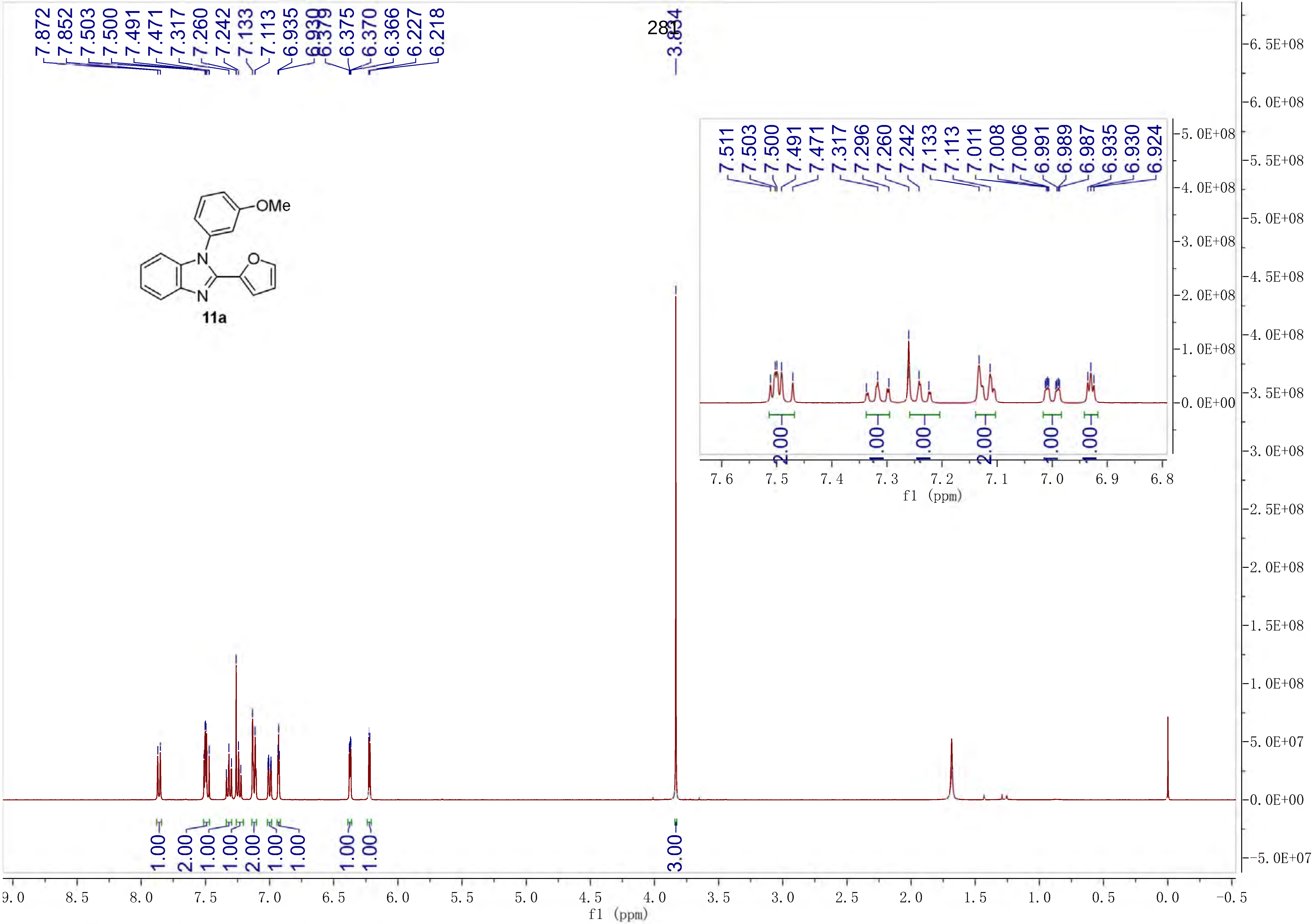

Supplementary Fig 209. ^{1}H NMR spectrum (400 MHz, $CDCl_3$, r.t.) of **11a**.

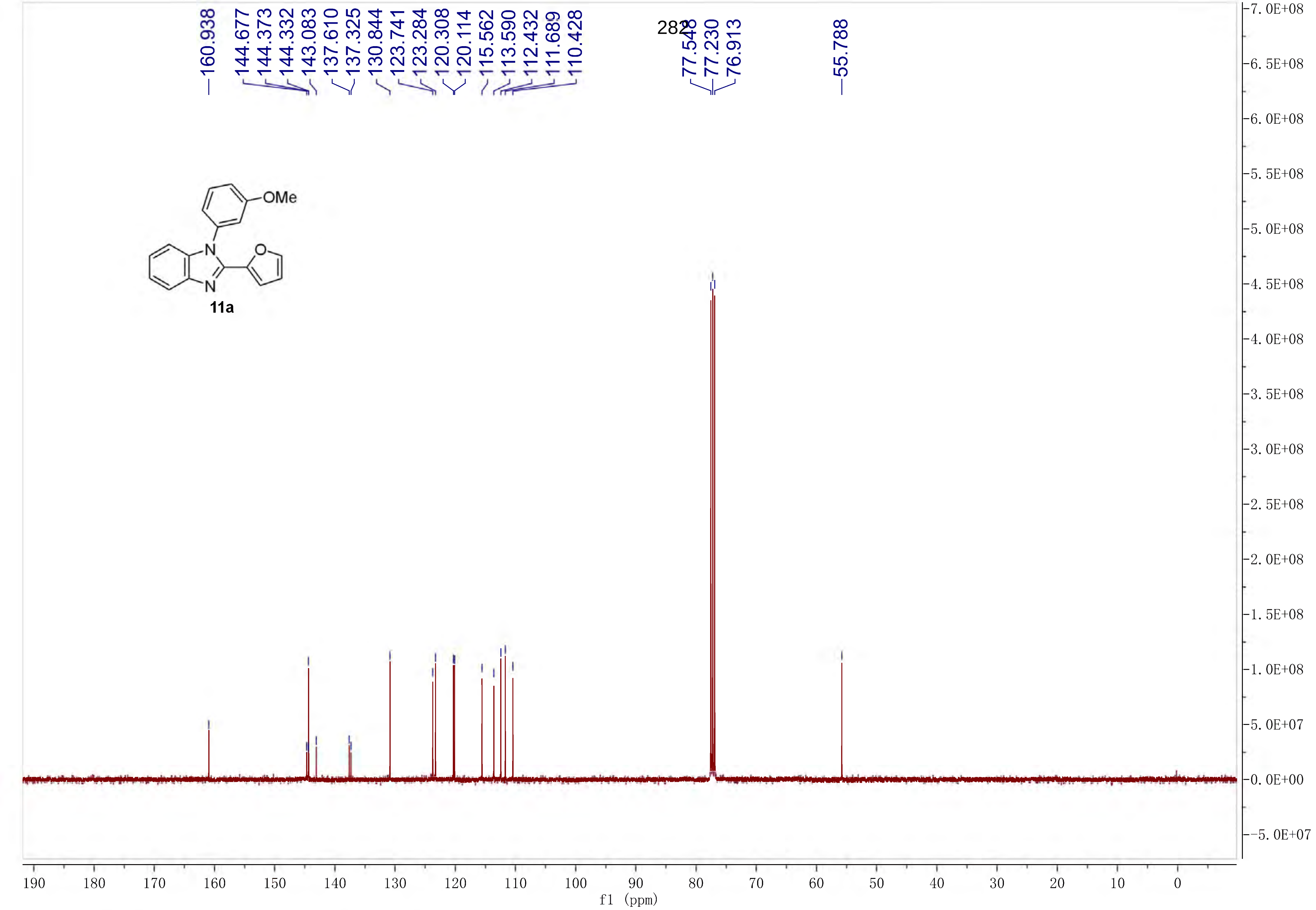

Supplementary Fig 210. ^{13}C NMR spectrum (400 MHz, $CDCl_3$, r.t.) of **11a**.

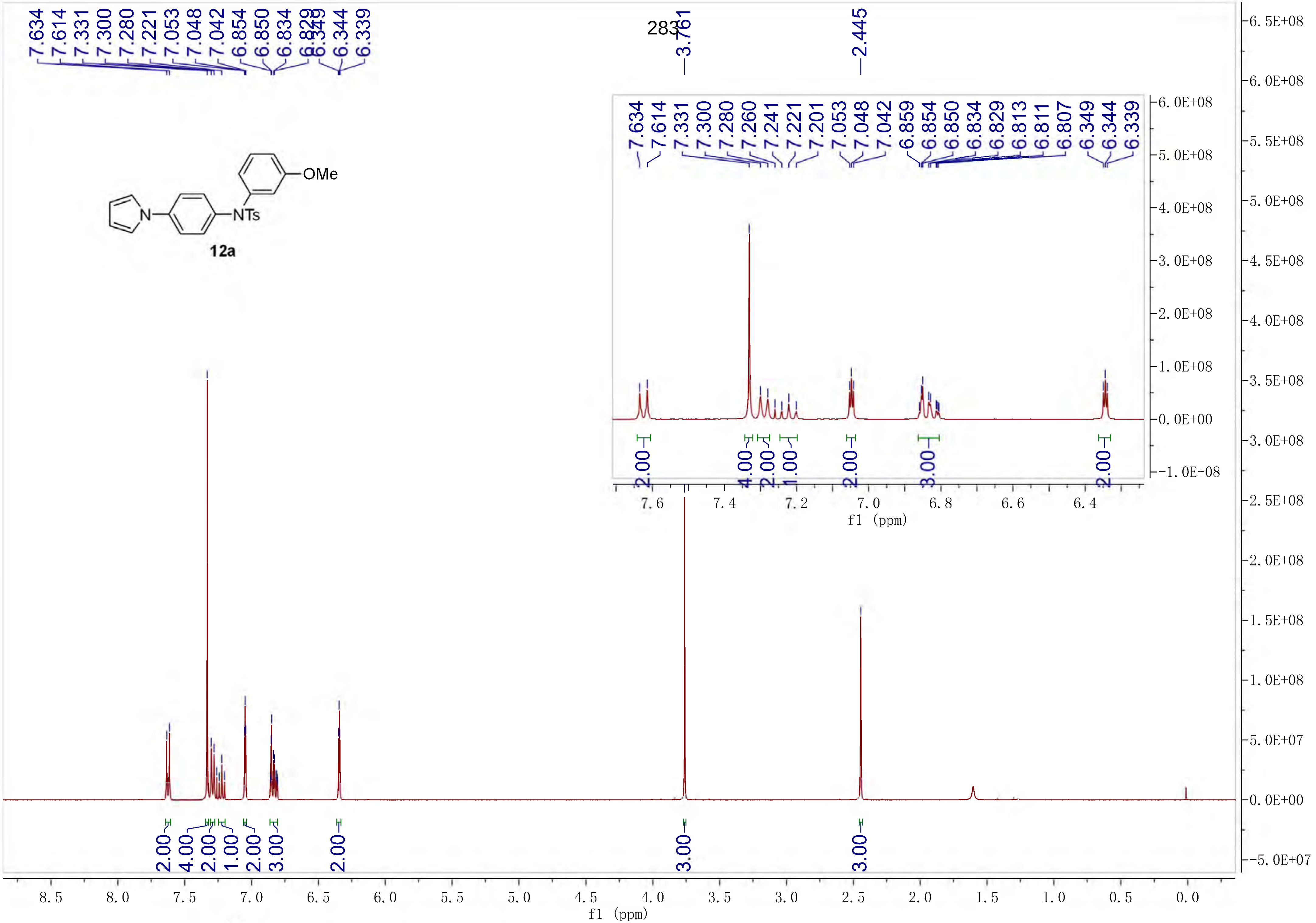

Supplementary Fig 211. ^{1}H NMR spectrum (400 MHz, $CDCl_3$, r.t.) of **12a**.

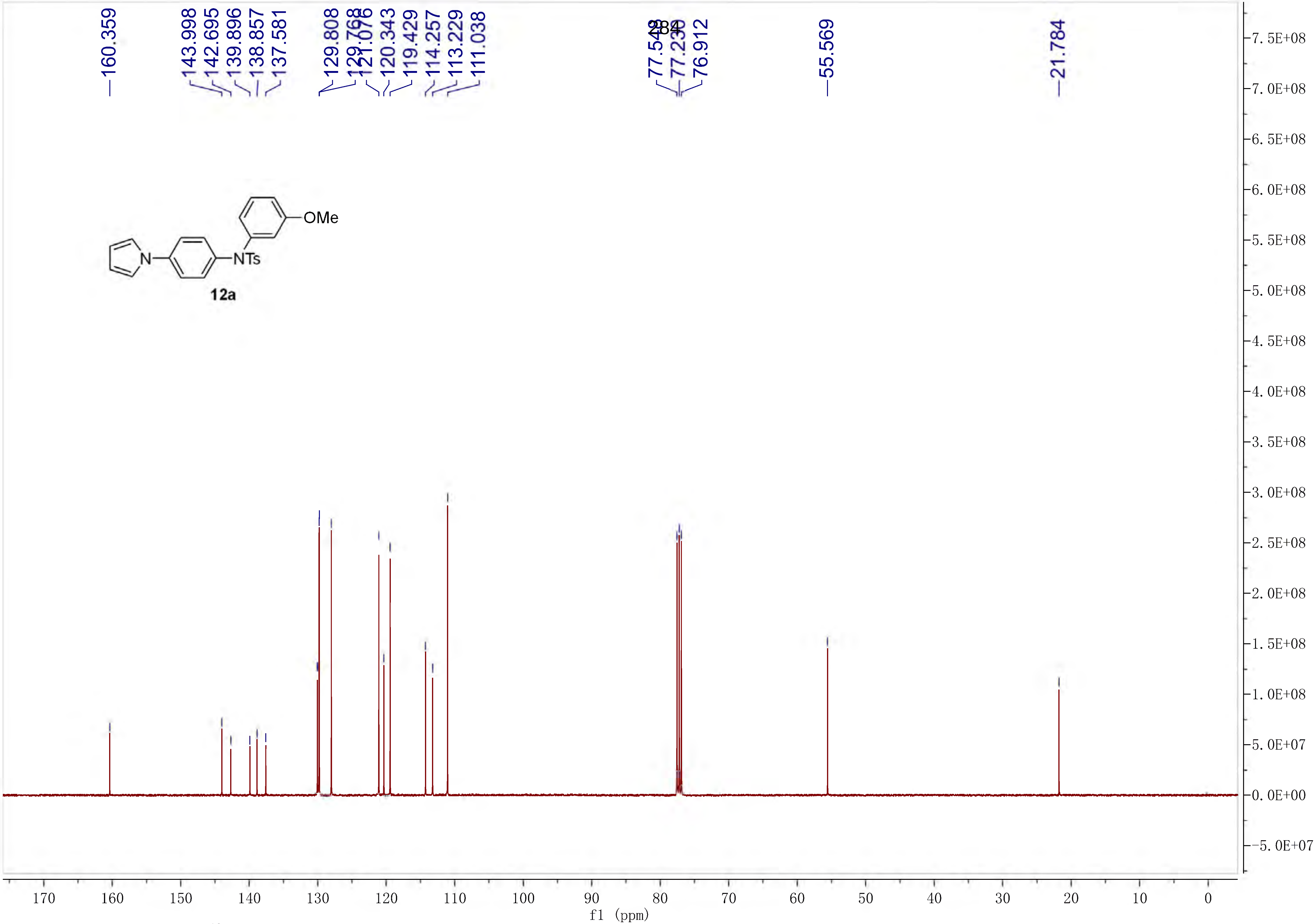

Supplementary Fig 212. ^{13}C NMR spectrum (400 MHz, $CDCl_3$, r.t.) of **12a**.

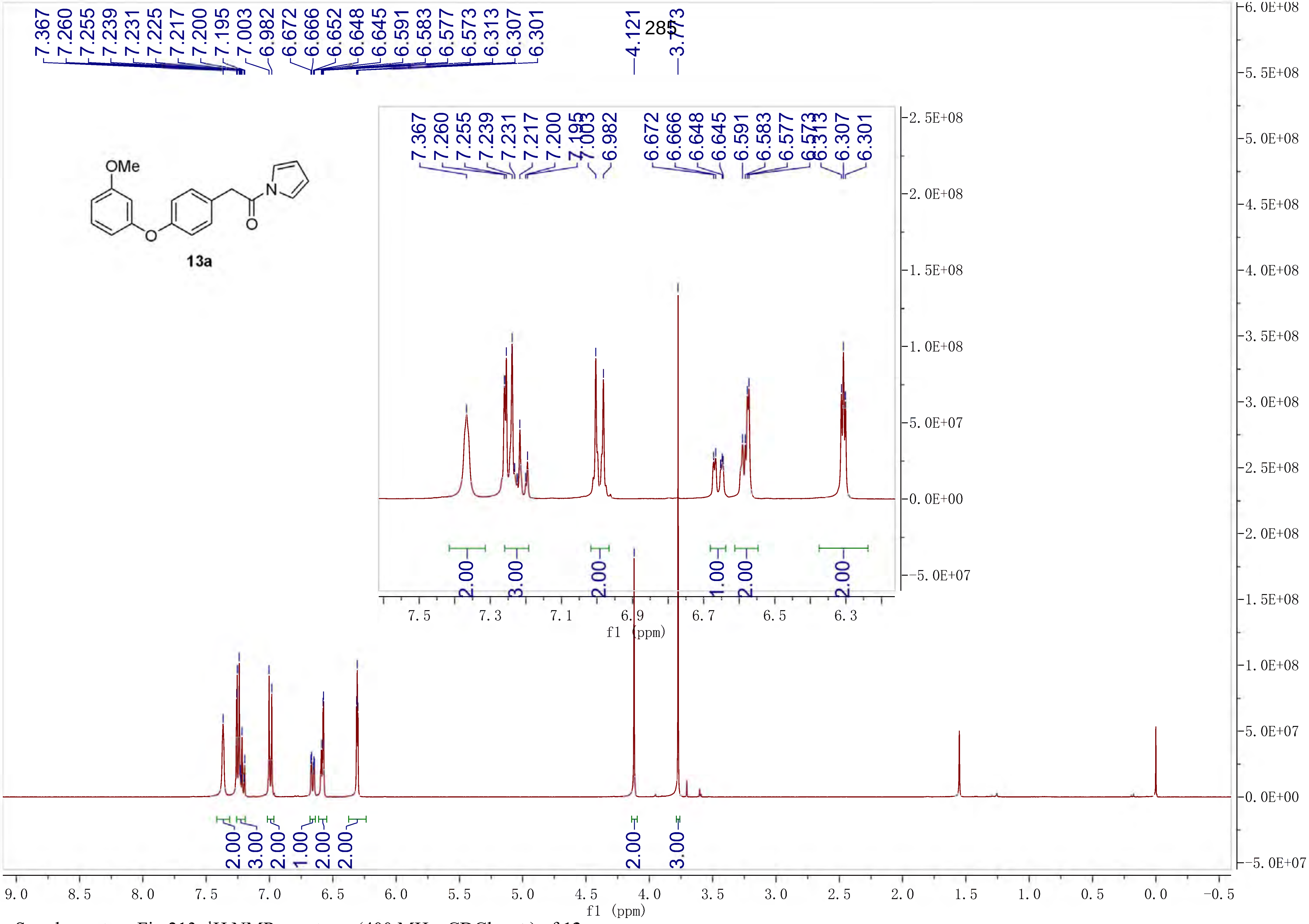

13a

Supplementary Fig 213. ^{1}H NMR spectrum (400 MHz, $CDCl_3$, r.t.) of **13a**.

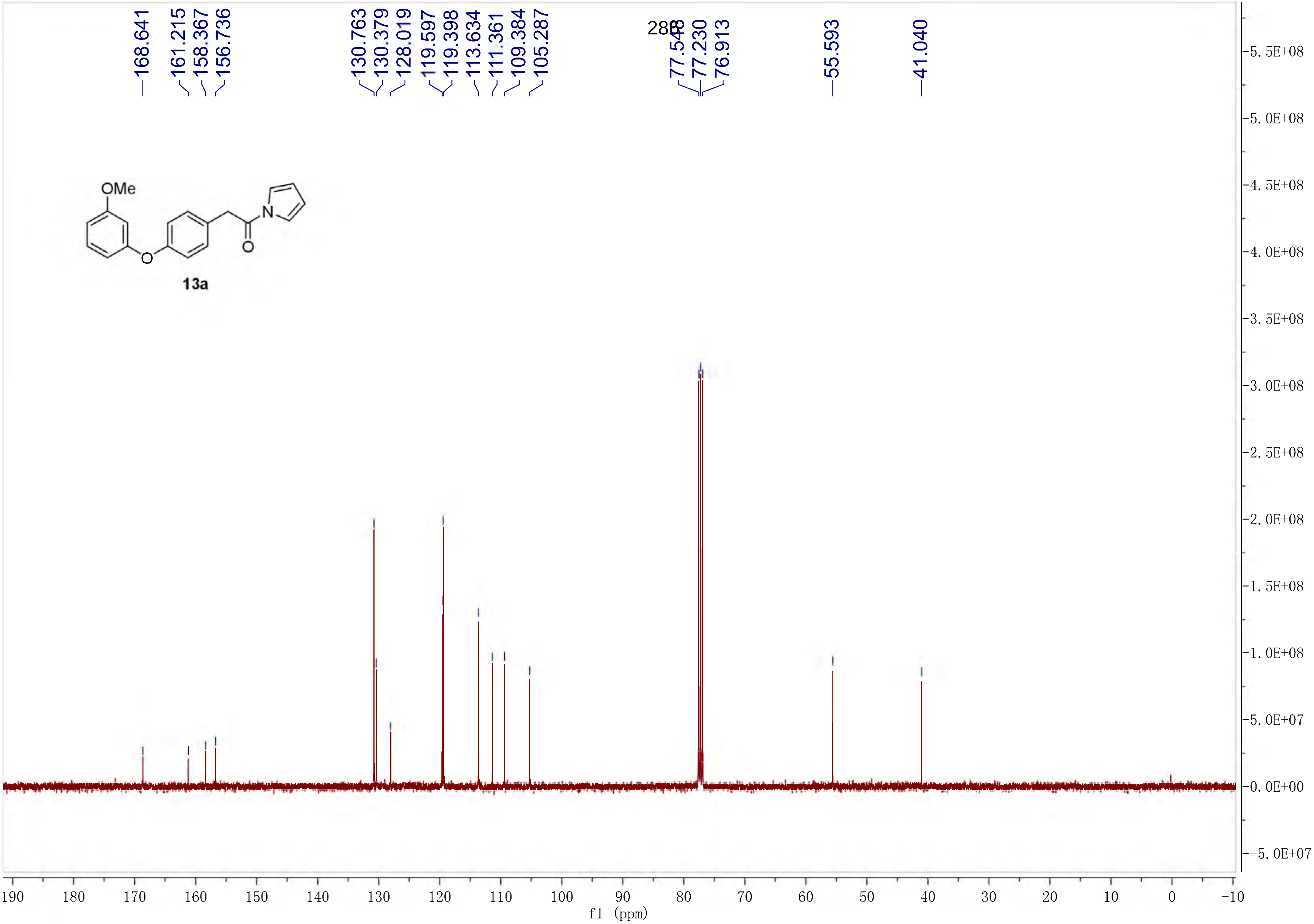

Supplementary Fig 214. ^{13}C NMR spectrum (400 MHz, $CDCl_3$, r.t.) of **13a**.

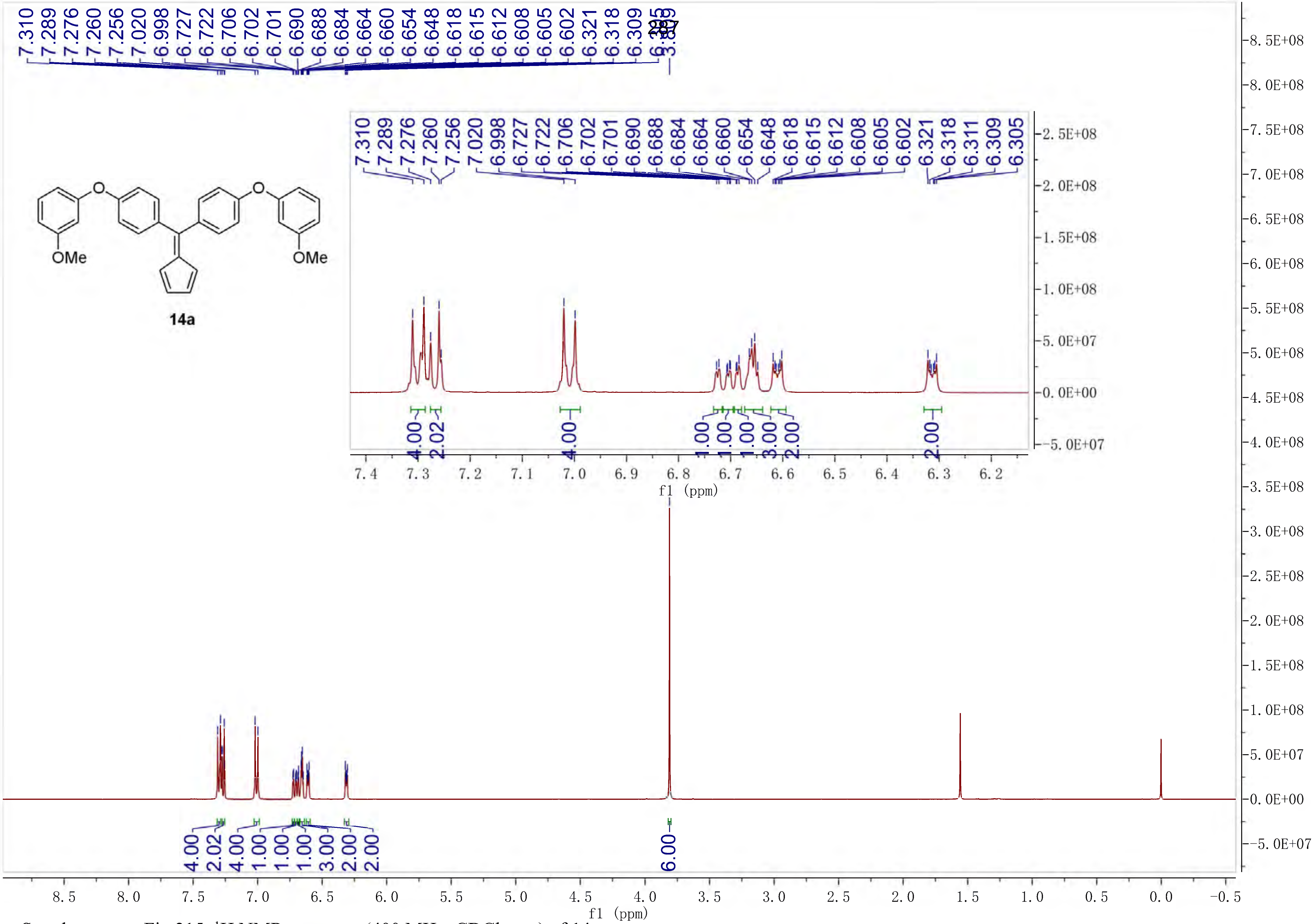

Supplementary Fig 215. ^{1}H NMR spectrum (400 MHz, $CDCl_3$, r.t.) of **14a**.

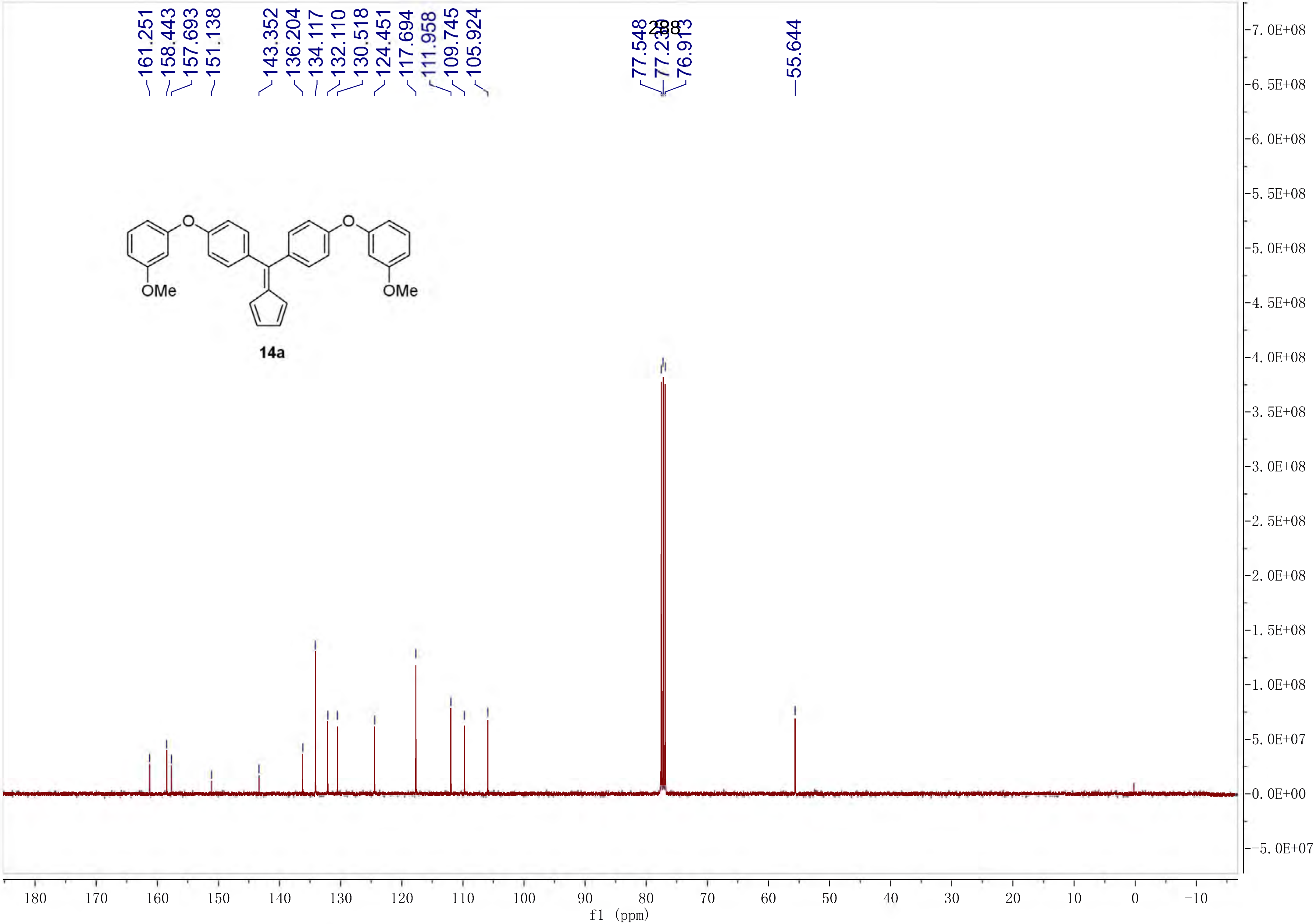

Supplementary Fig 216. ^{13}C NMR spectrum (400 MHz, $CDCl_3$, r.t.) of **14a**.

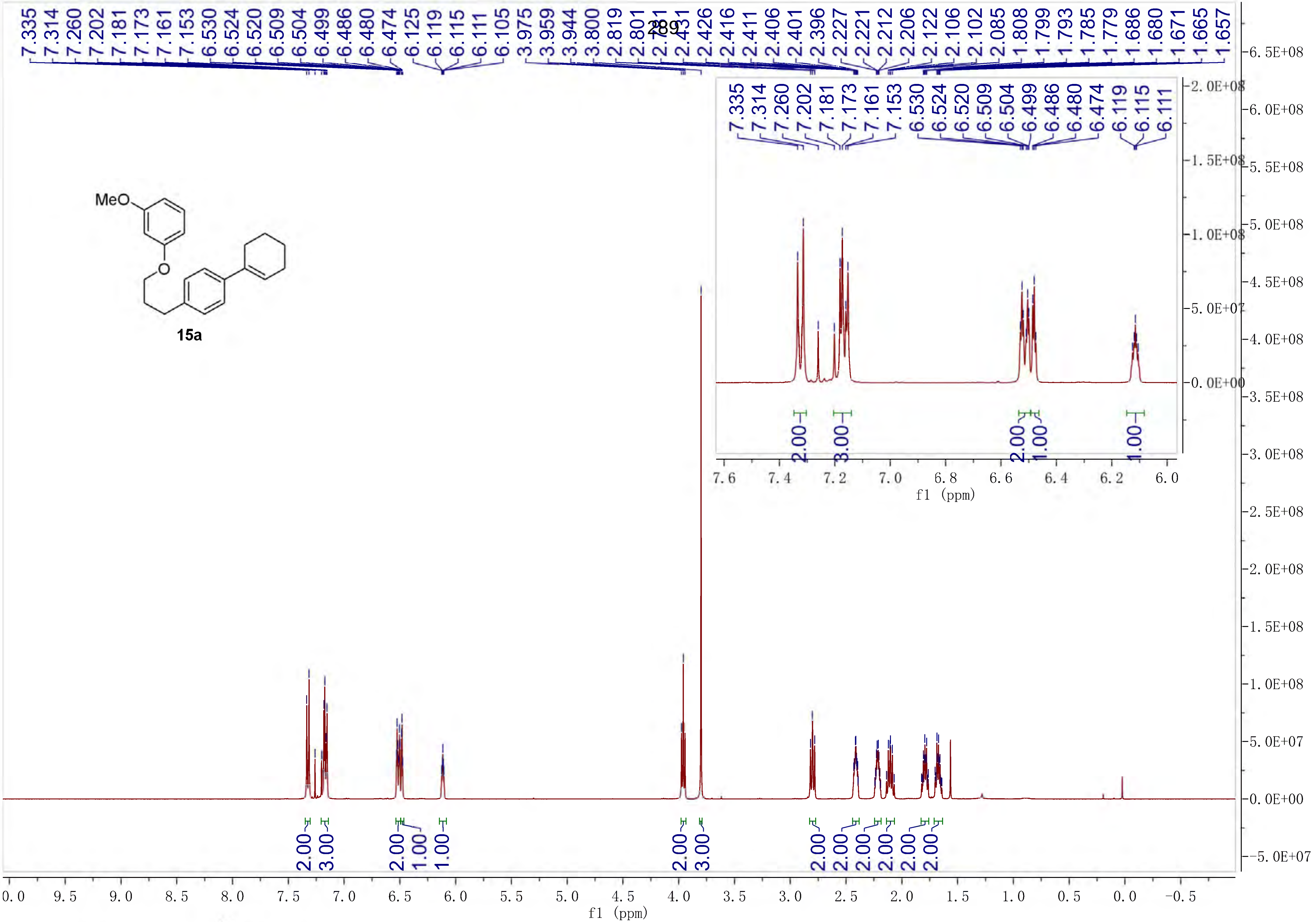

Supplementary Fig 217. ^{1}H NMR spectrum (400 MHz, $CDCl_3$, r.t.) of **15a**.

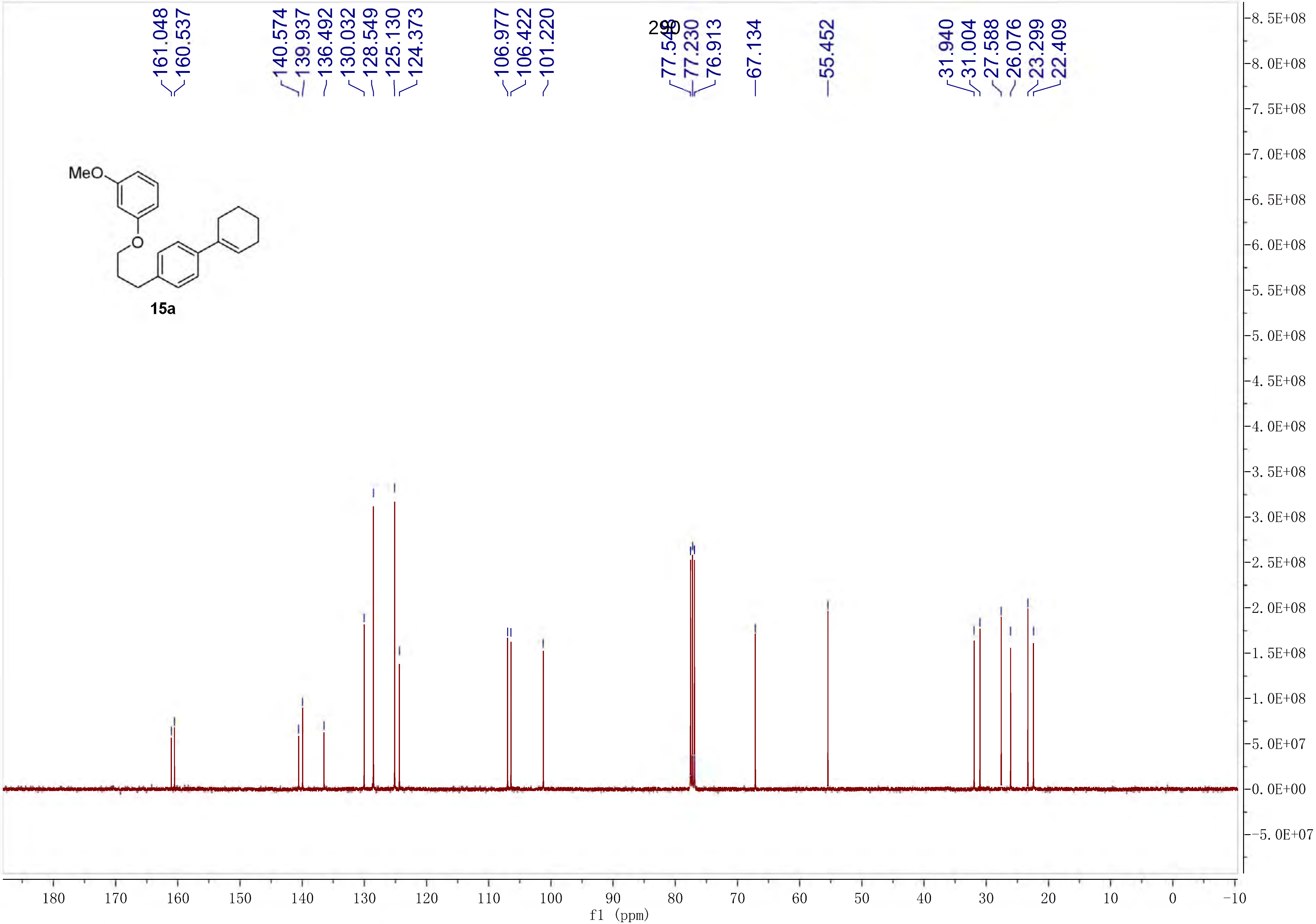

Supplementary Fig 218. ^{13}C NMR spectrum (400 MHz, $CDCl_3$, r.t.) of **15a**.

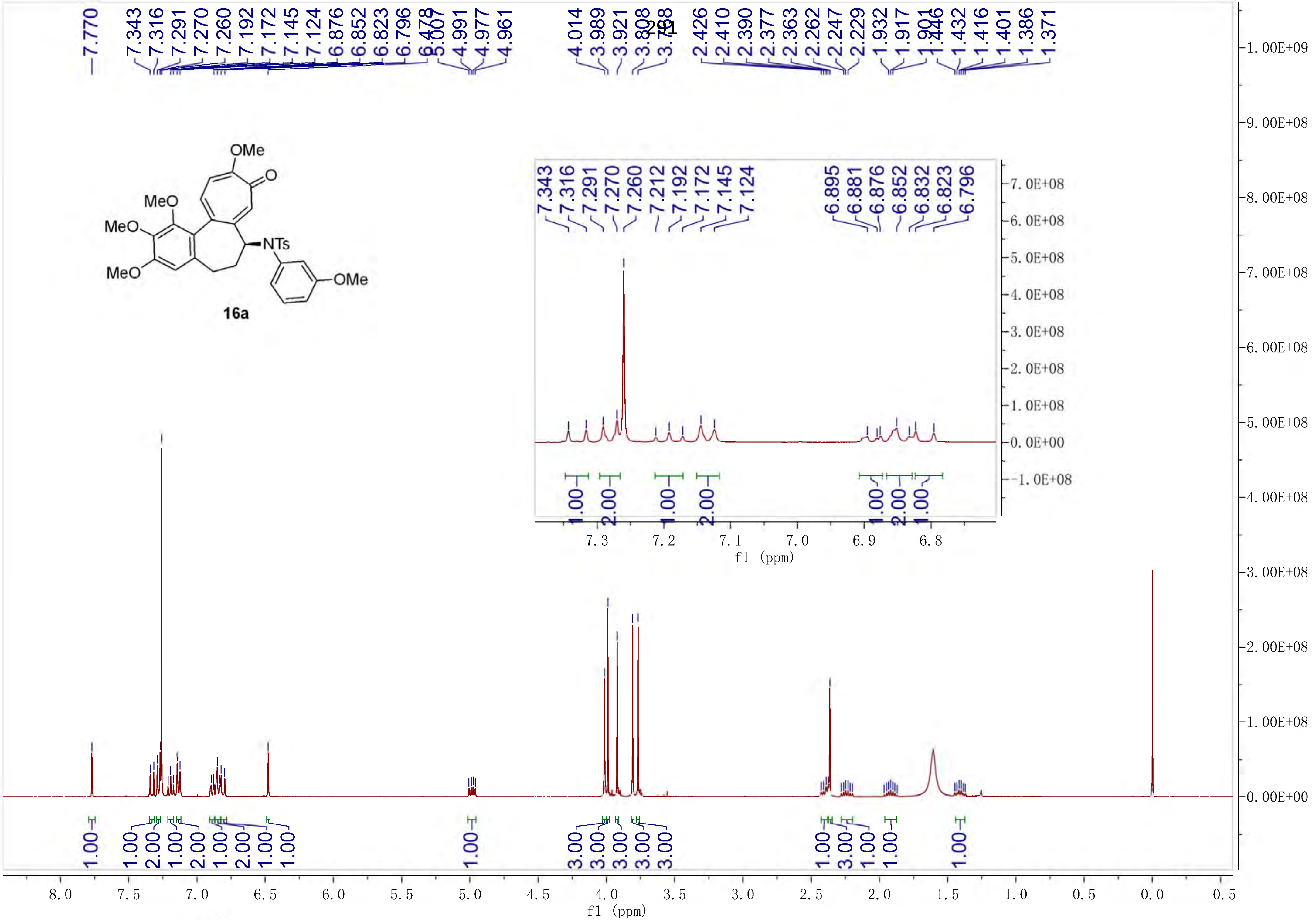

Supplementary Fig 219. ^{1}H NMR spectrum (400 MHz, $CDCl_3$, r.t.) of **16a**.

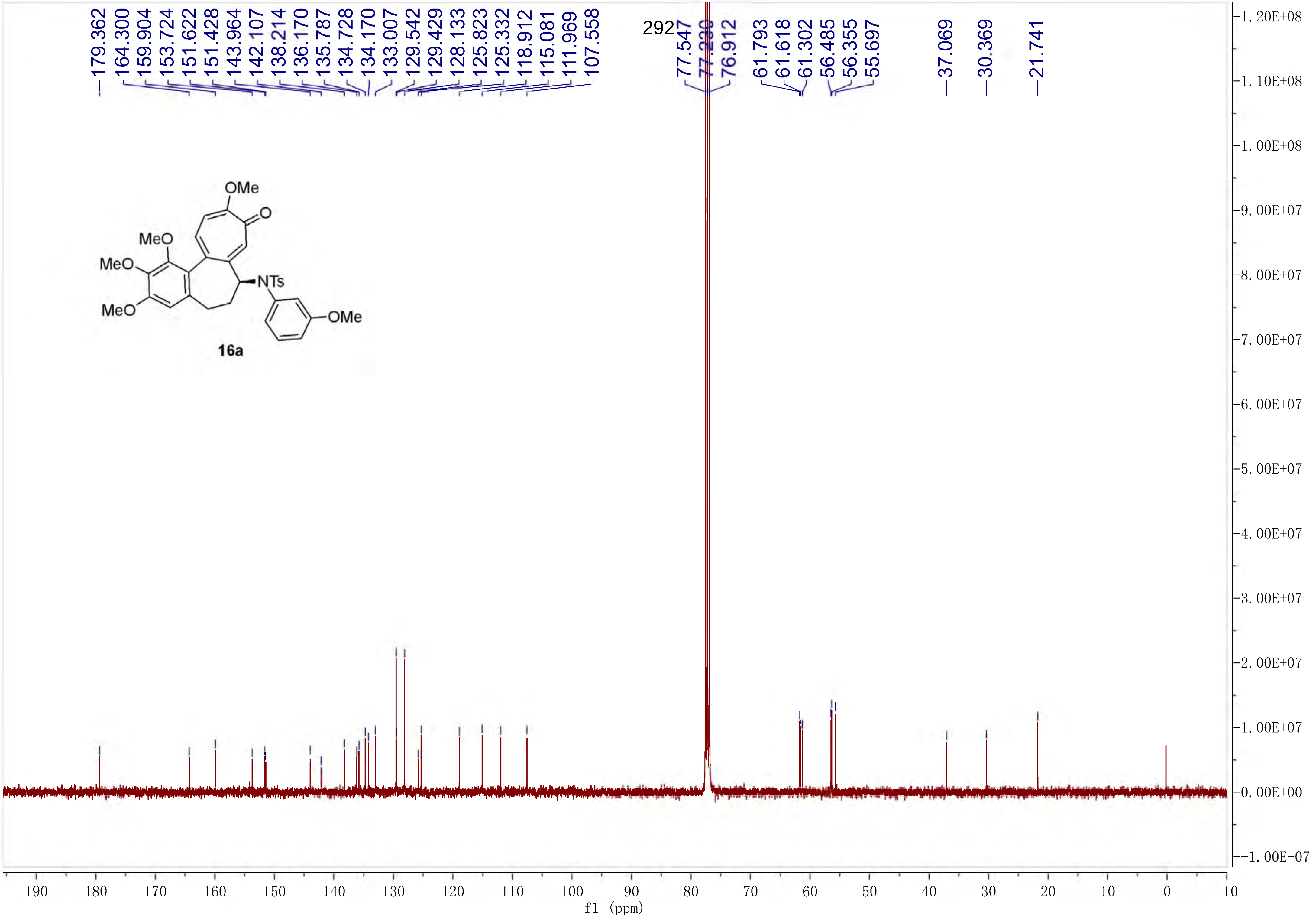

Supplementary Fig 220. ^{13}C NMR spectrum (400 MHz, $CDCl_3$, r.t.) of **16a**.

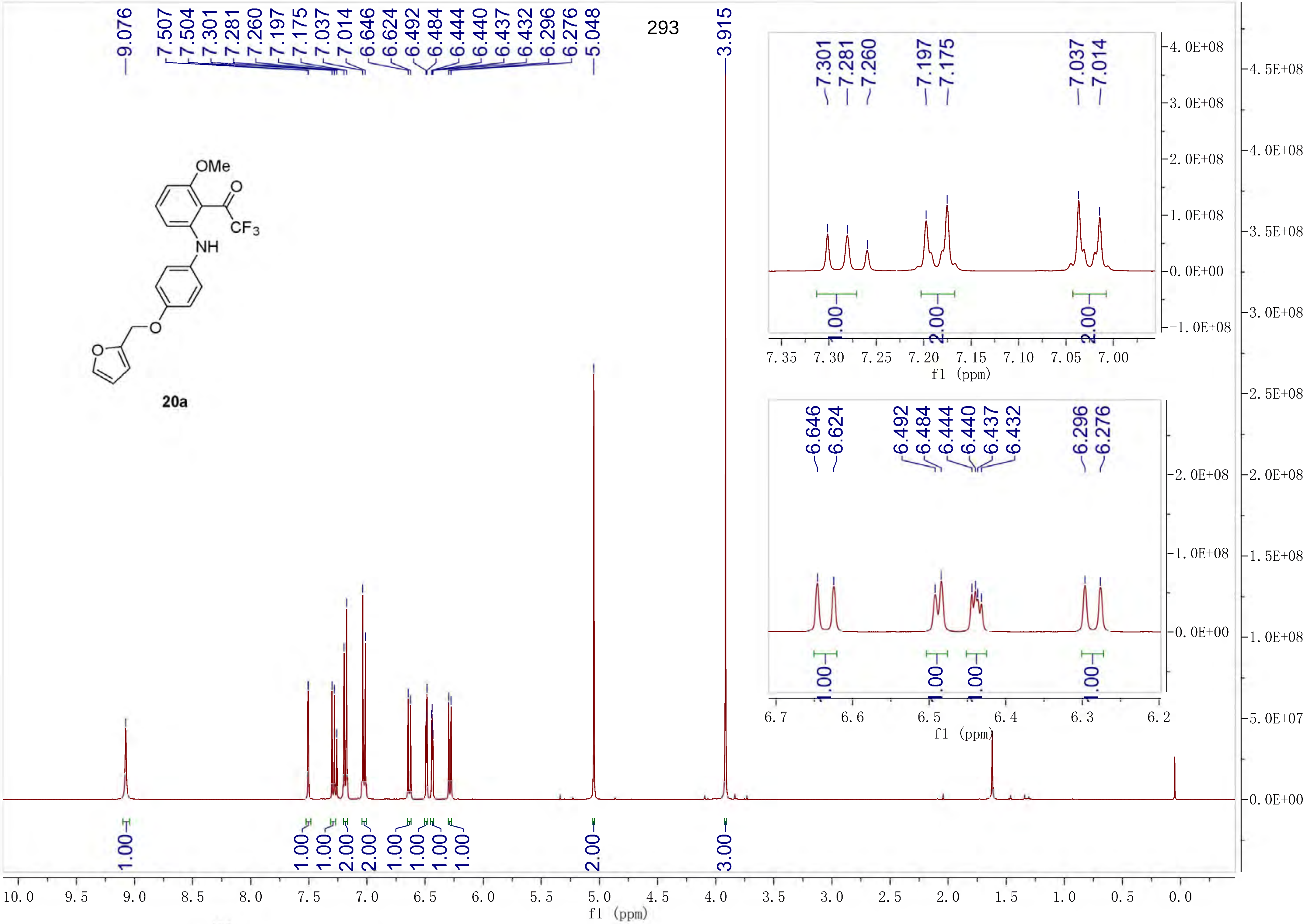

Supplementary Fig 221. ^{1}H NMR spectrum (400 MHz, $CDCl_3$, r.t.) of **20a**.

294

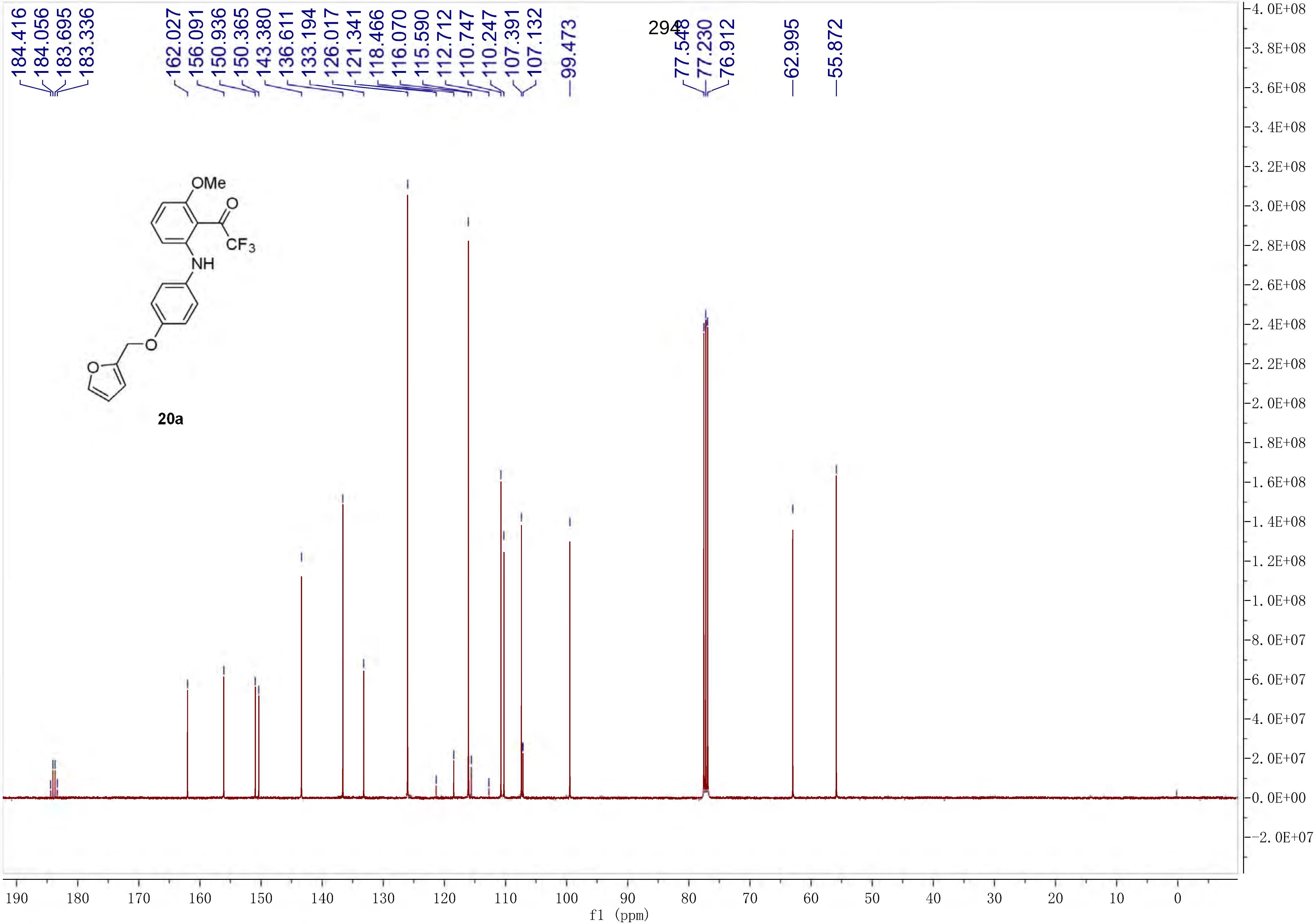

Supplementary Fig 222. ^{13}C NMR spectrum (400 MHz, $CDCl_3$, r.t.) of **20a**.

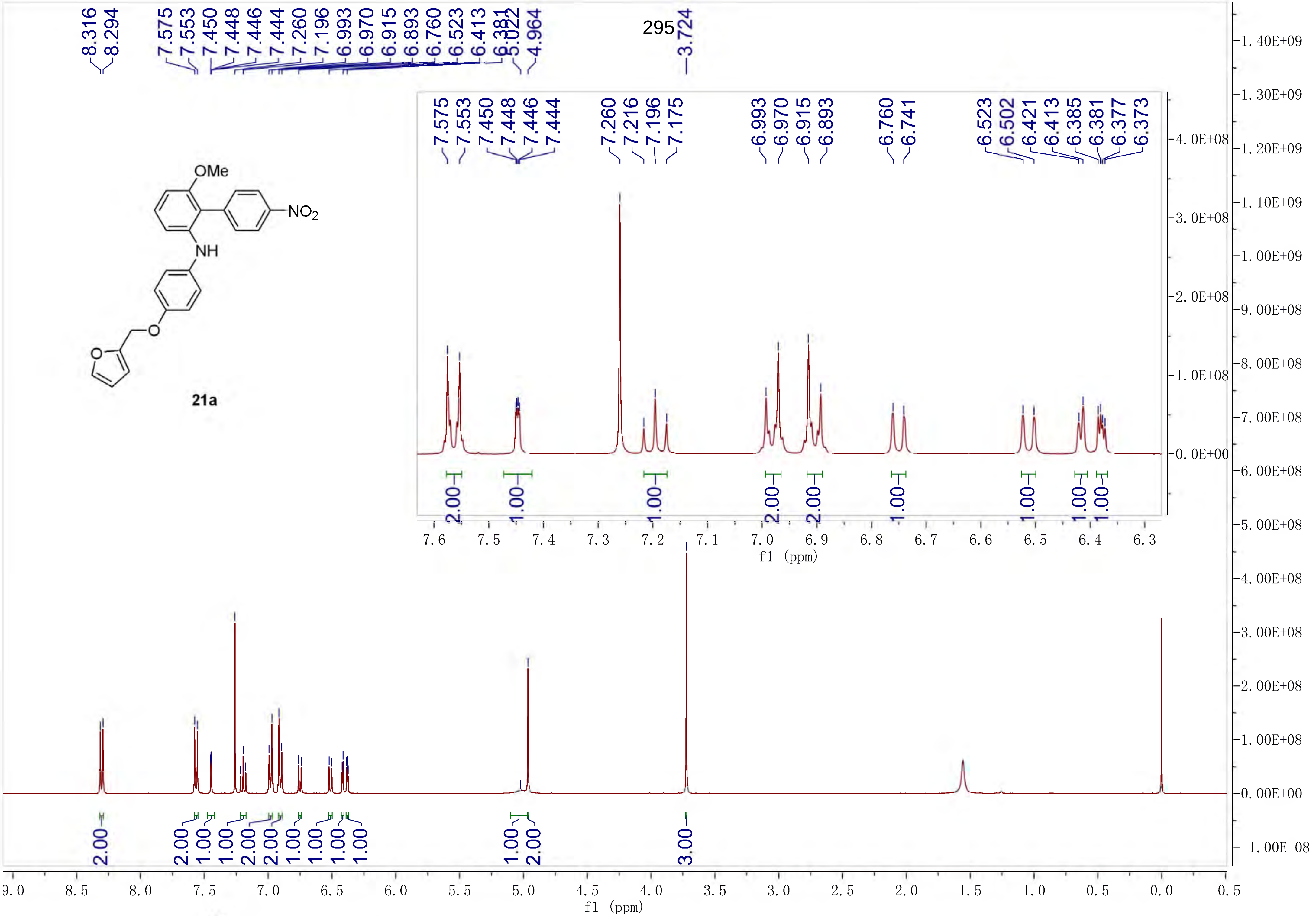

Supplementary Fig 223. ^{1}H NMR spectrum (400 MHz, $CDCl_3$, r.t.) of **21a**.

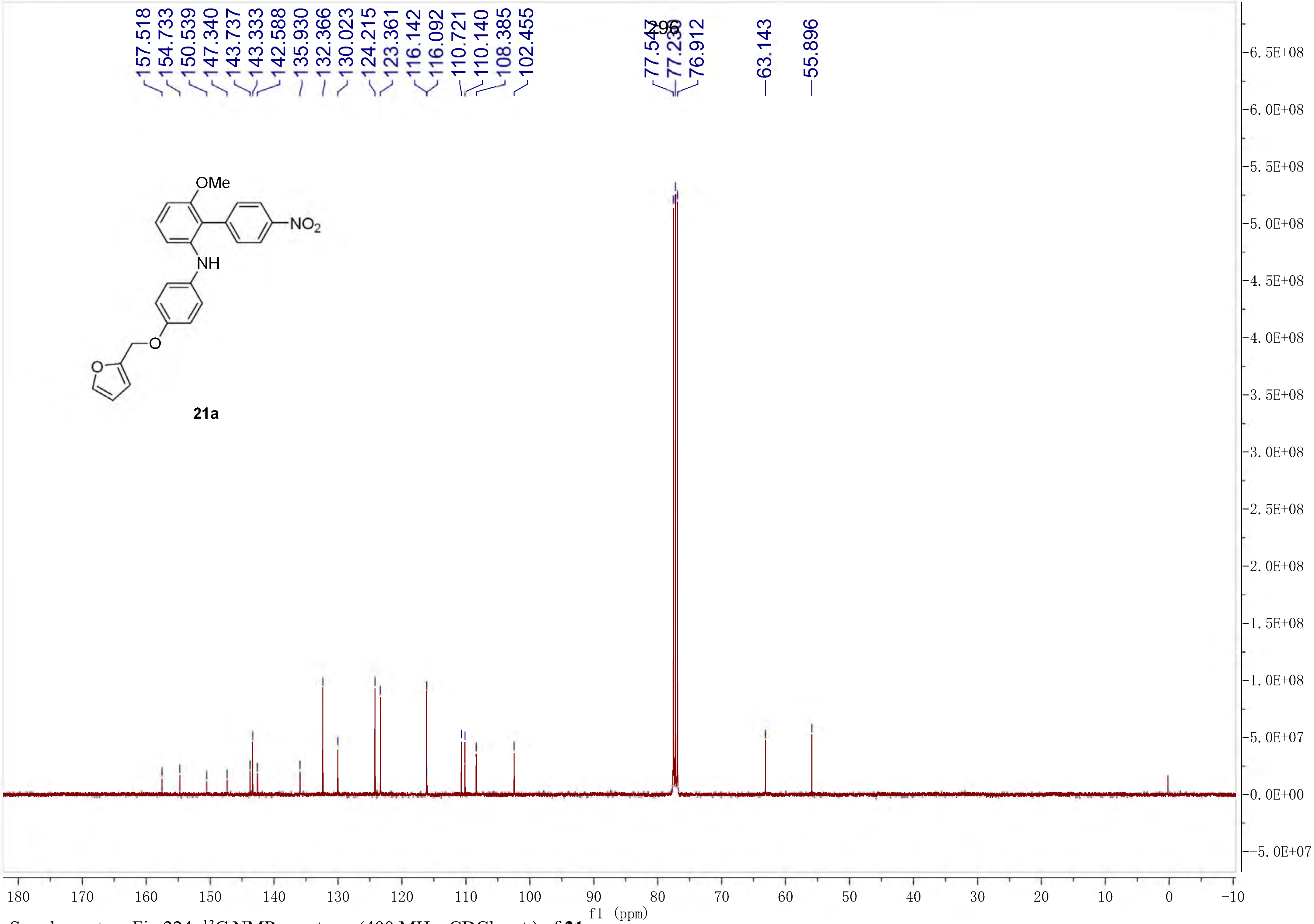

Supplementary Fig 224. ^{13}C NMR spectrum (400 MHz, $CDCl_3$, r.t.) of **21a**.

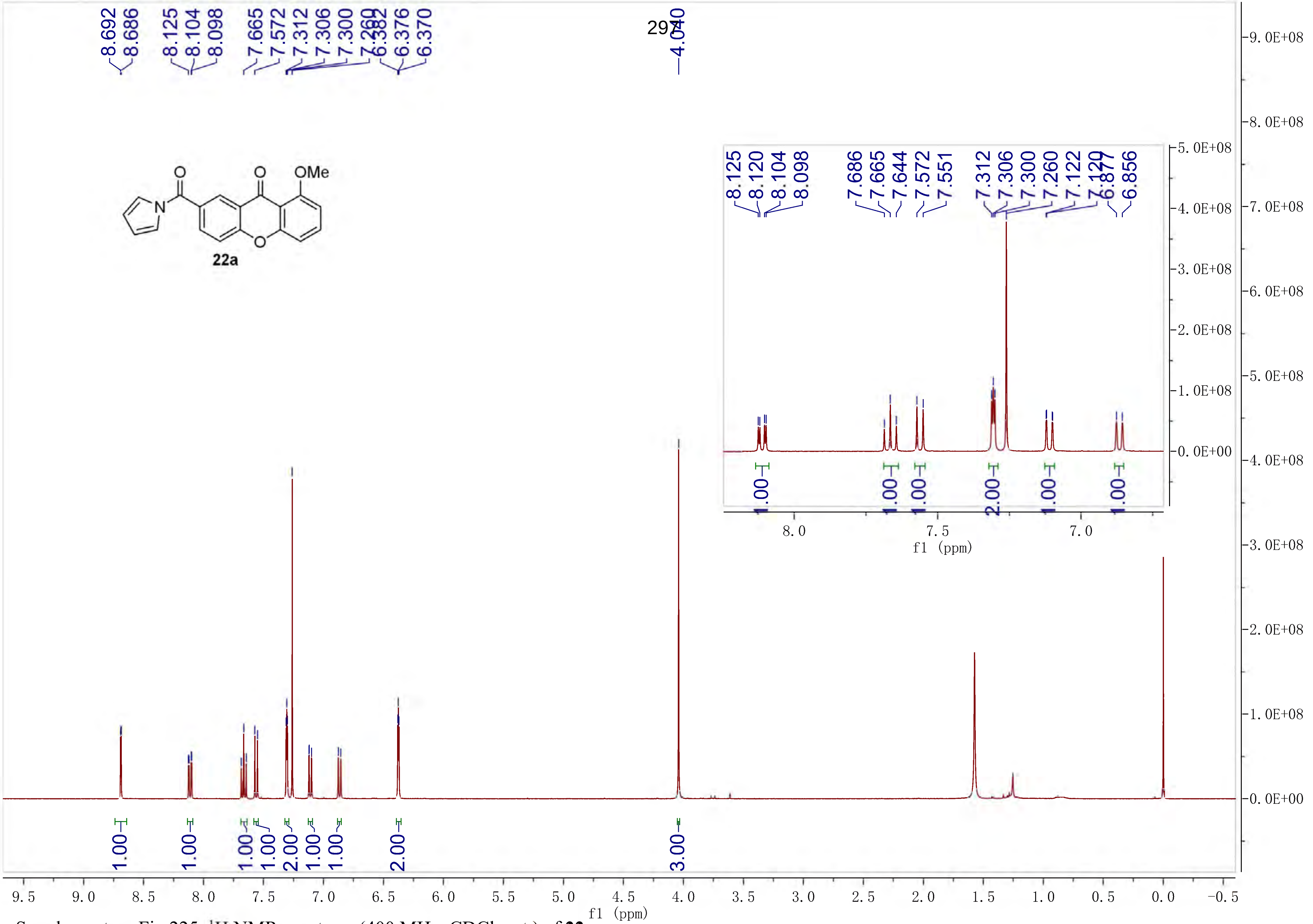

Supplementary Fig 225. ^{1}H NMR spectrum (400 MHz, $CDCl_3$, r.t.) of **22a**.

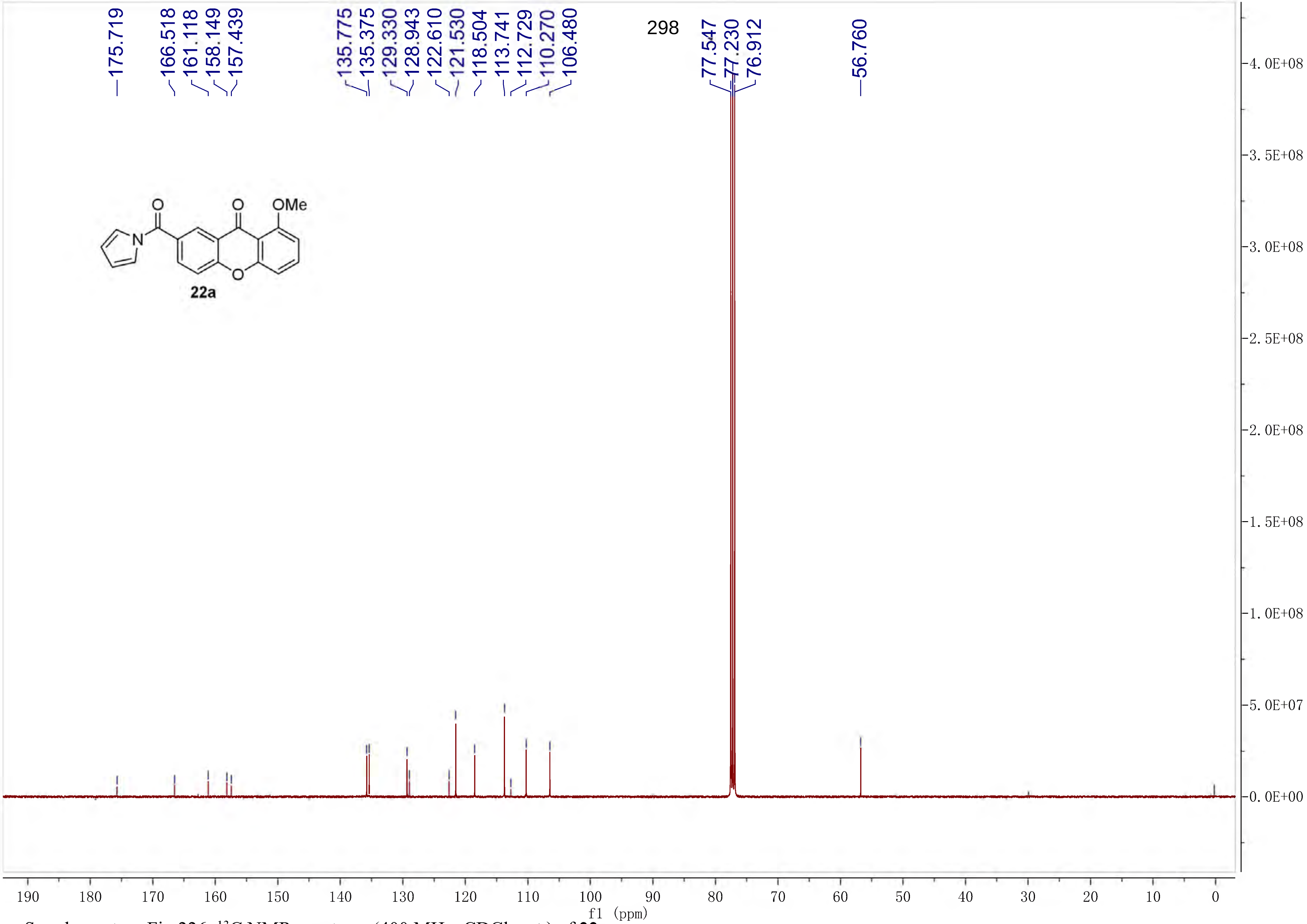

Supplementary Fig 226. ^{13}C NMR spectrum (400 MHz, $CDCl_3$, r.t.) of **22a**.

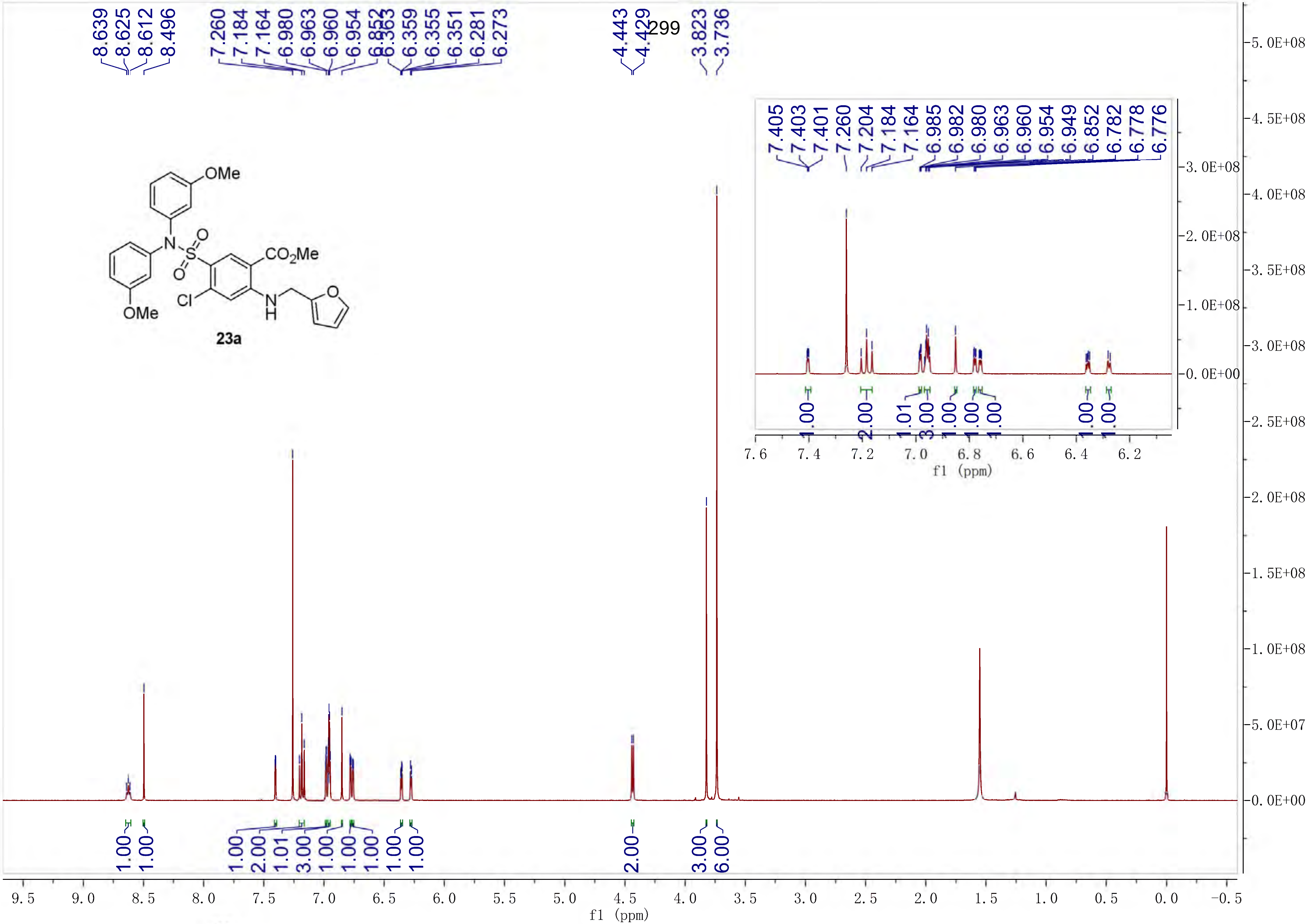

Supplementary Fig 227. ^{1}H NMR spectrum (400 MHz, $CDCl_3$, r.t.) of **23a**.

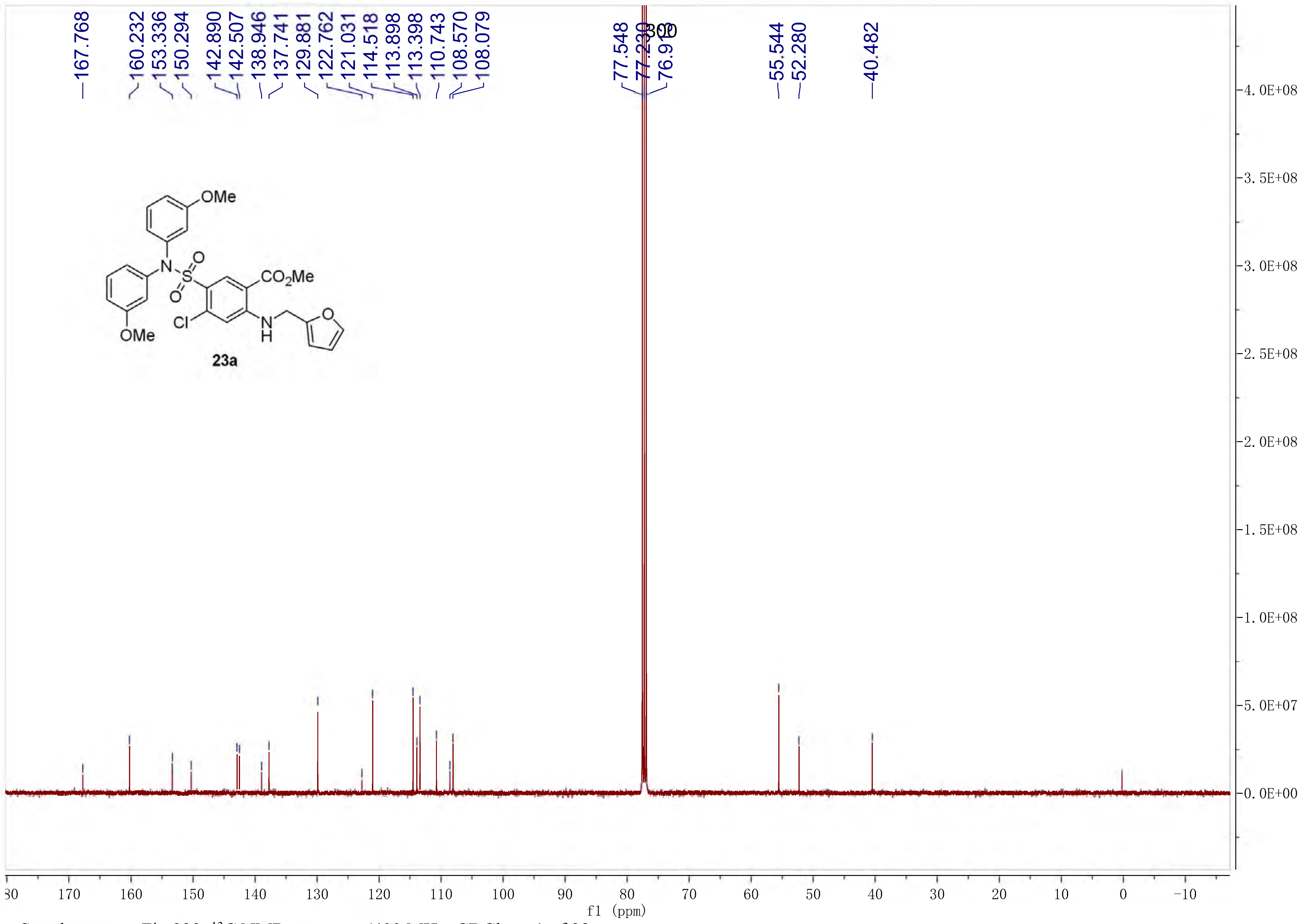

Supplementary Fig 228. ^{13}C NMR spectrum (400 MHz, $CDCl_3$, r.t.) of **23a**.

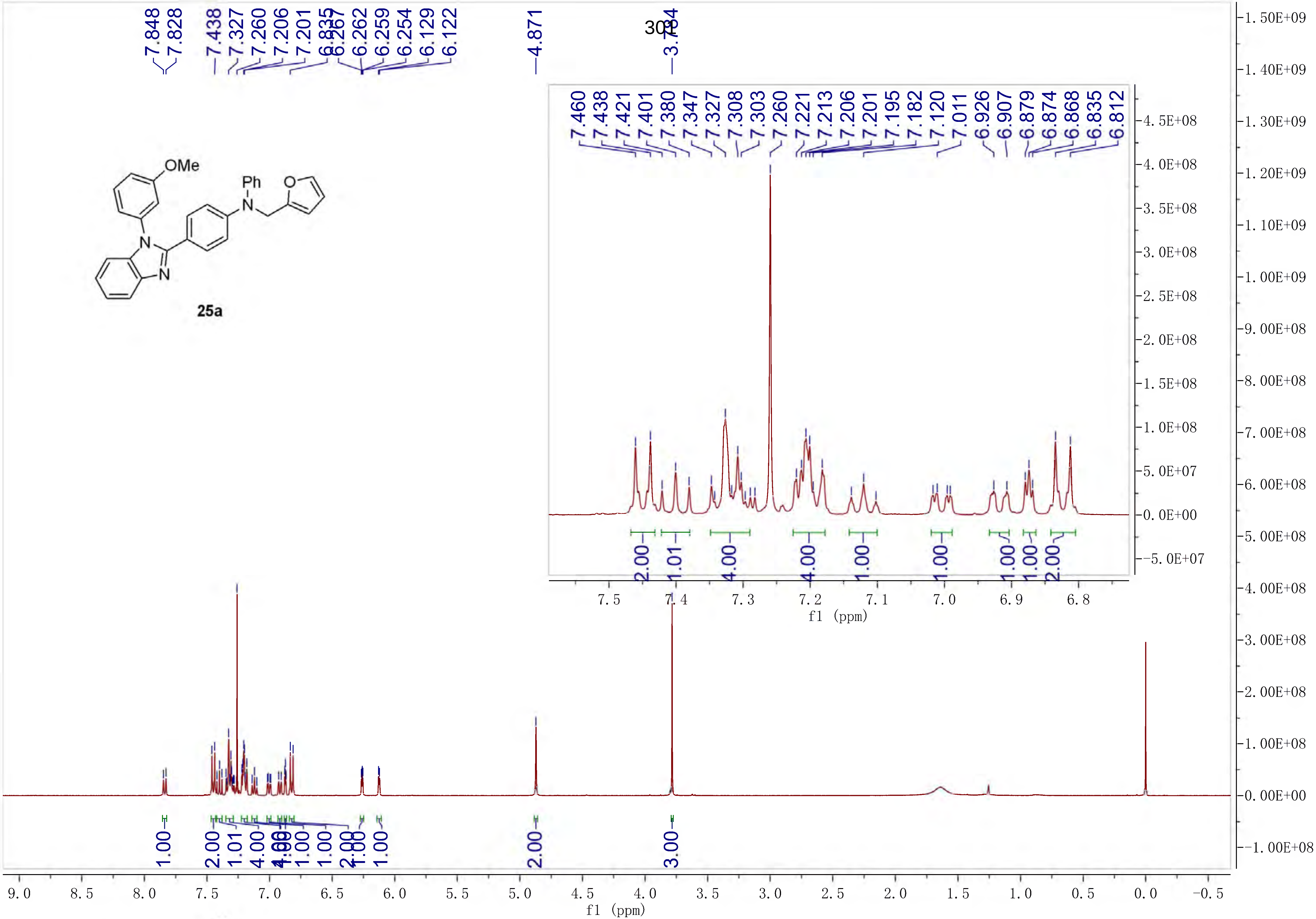

Supplementary Fig 229. ^{1}H NMR spectrum (400 MHz, $CDCl_3$, r.t.) of **25a**.

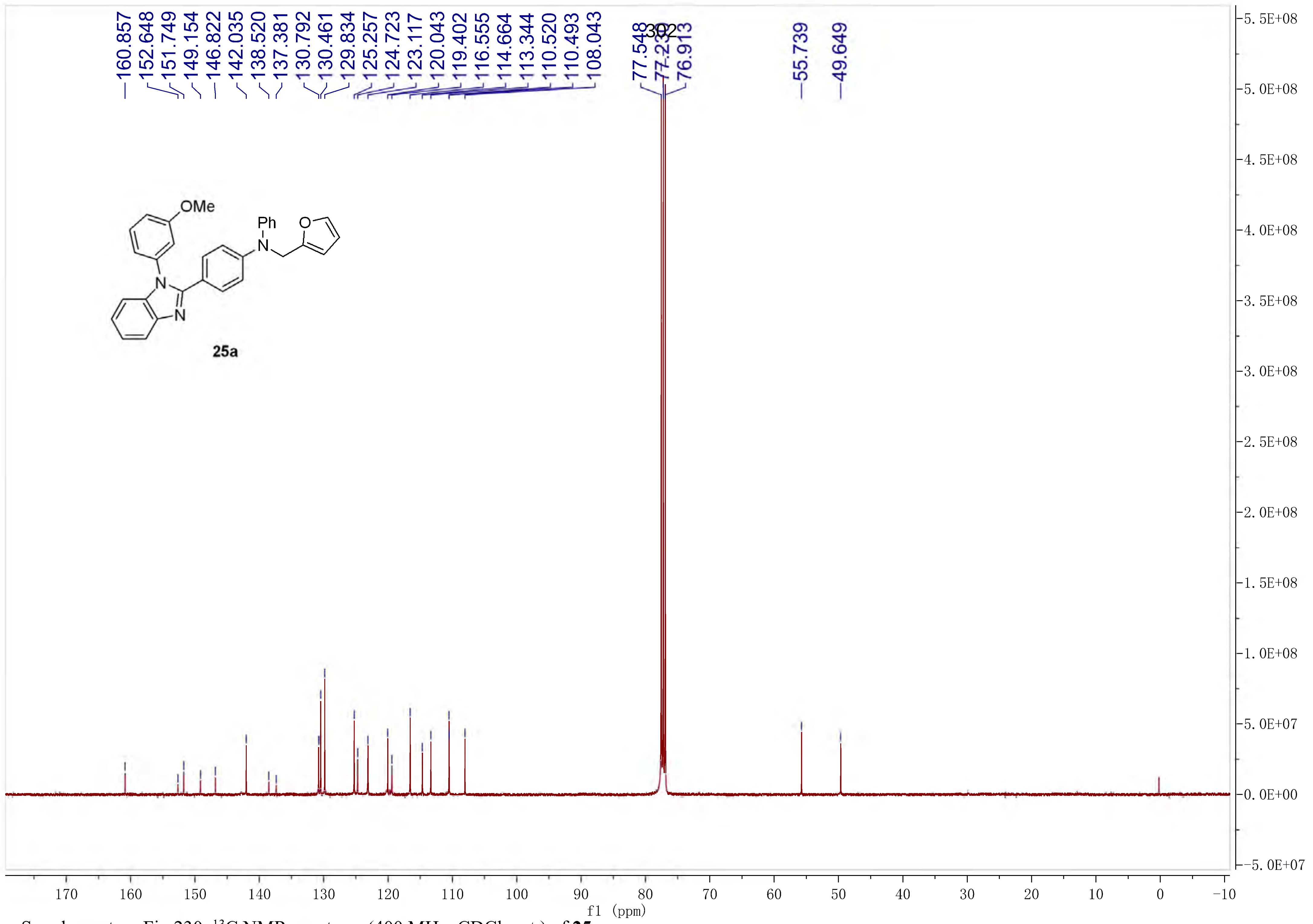

Supplementary Fig 230. ^{13}C NMR spectrum (400 MHz, $CDCl_3$, r.t.) of **25a**.

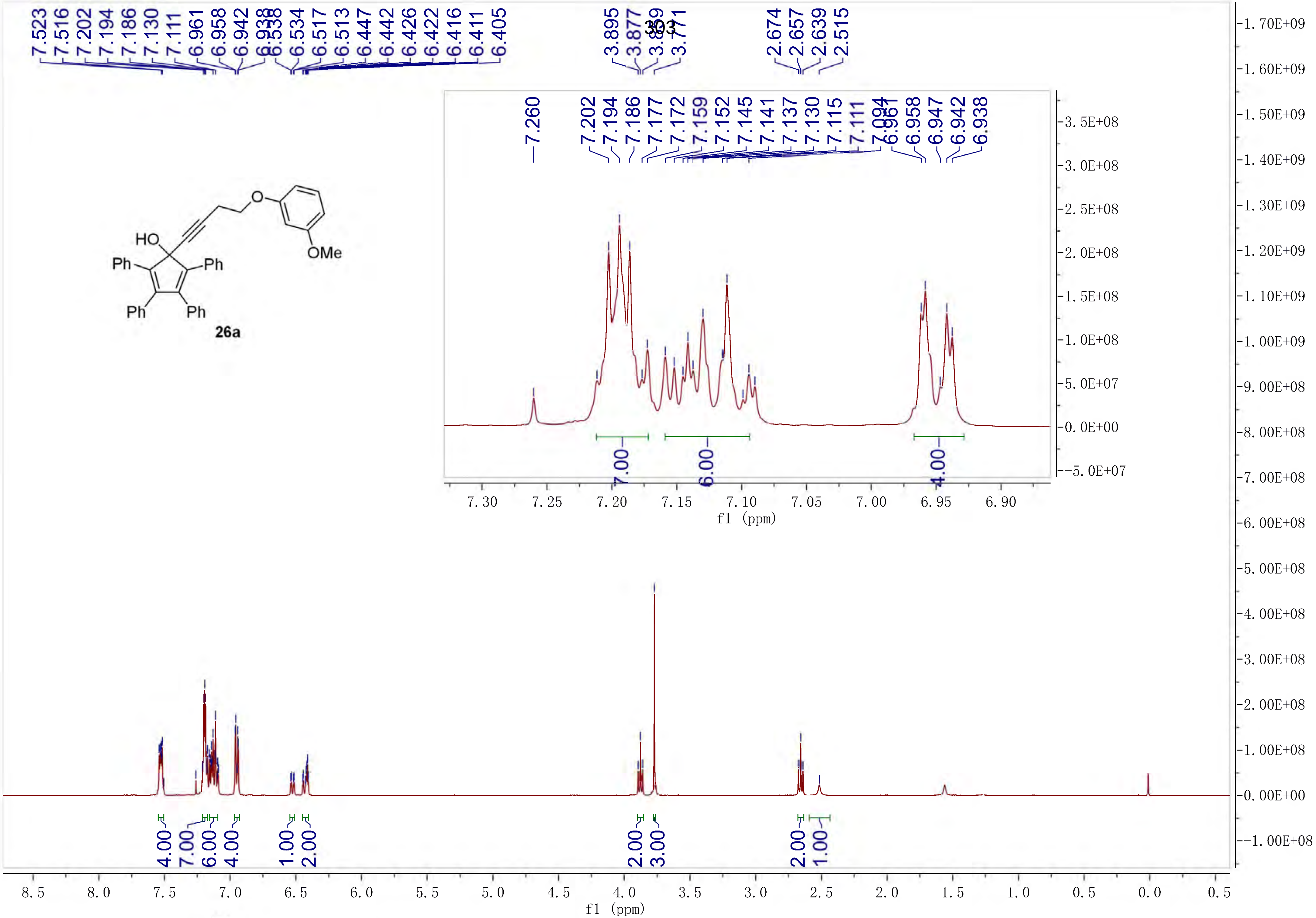

Supplementary Fig 231. ^{1}H NMR spectrum (400 MHz, $CDCl_3$, r.t.) of **26a**.

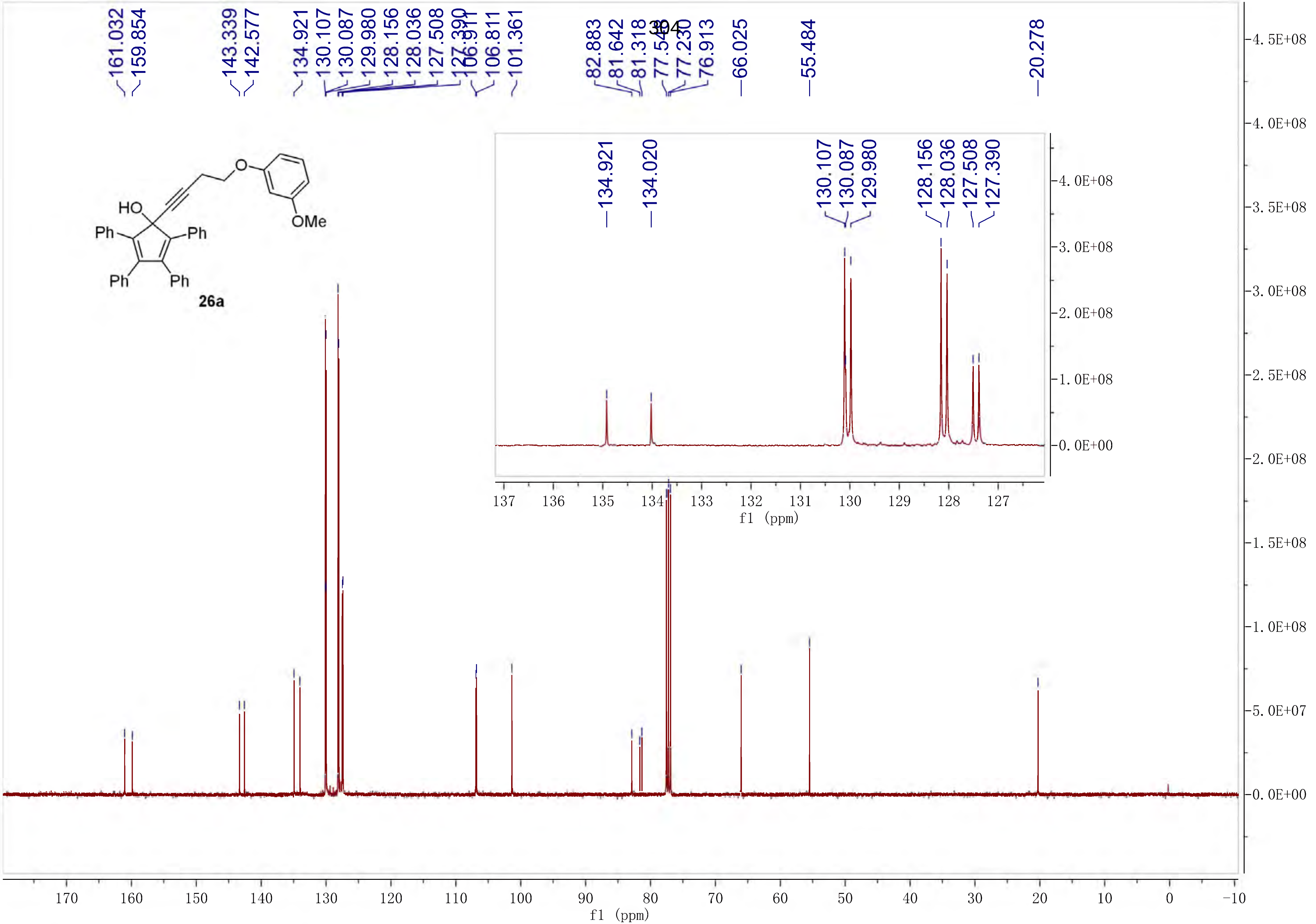

Supplementary Fig 232. ^{13}C NMR spectrum (400 MHz, $CDCl_3$, r.t.) of **26a**.

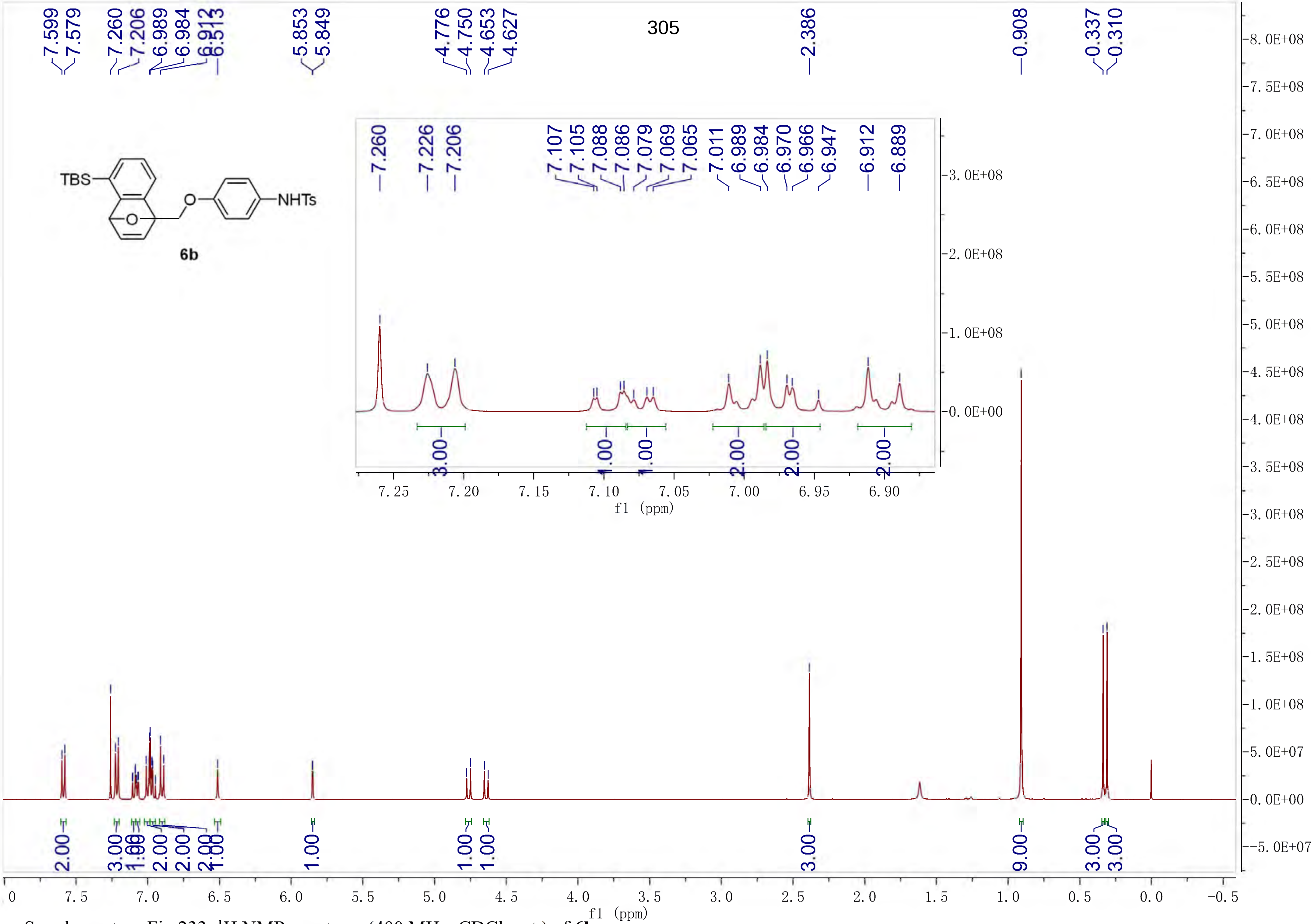

Supplementary Fig 233. ^{1}H NMR spectrum (400 MHz, $CDCl_3$, r.t.) of **6b**.

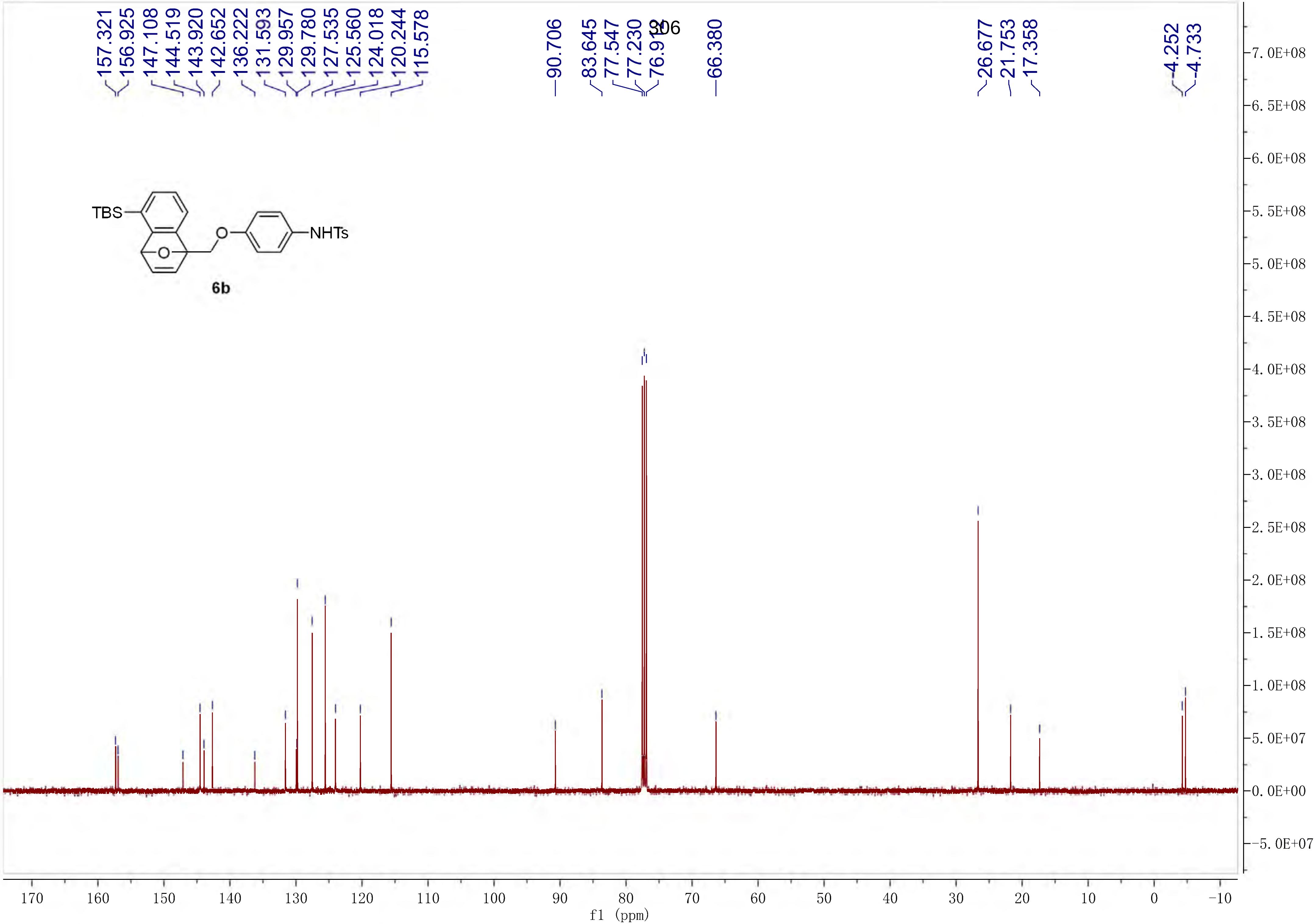

Supplementary Fig 234. ^{13}C NMR spectrum (400 MHz, $CDCl_3$, r.t.) of **6b**.

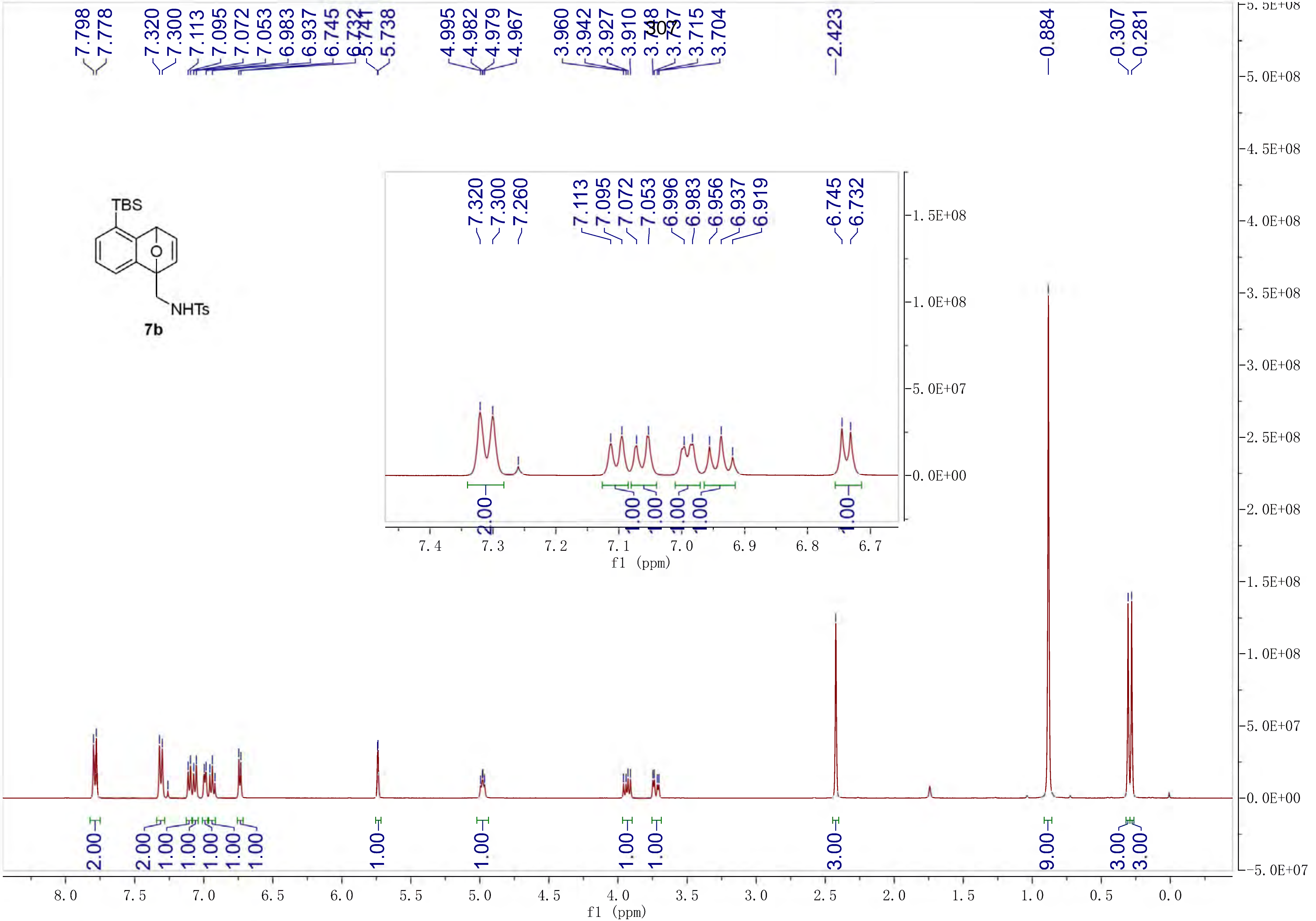

Supplementary Fig 235. ^{1}H NMR spectrum (400 MHz, $CDCl_3$, r.t.) of **7b**.

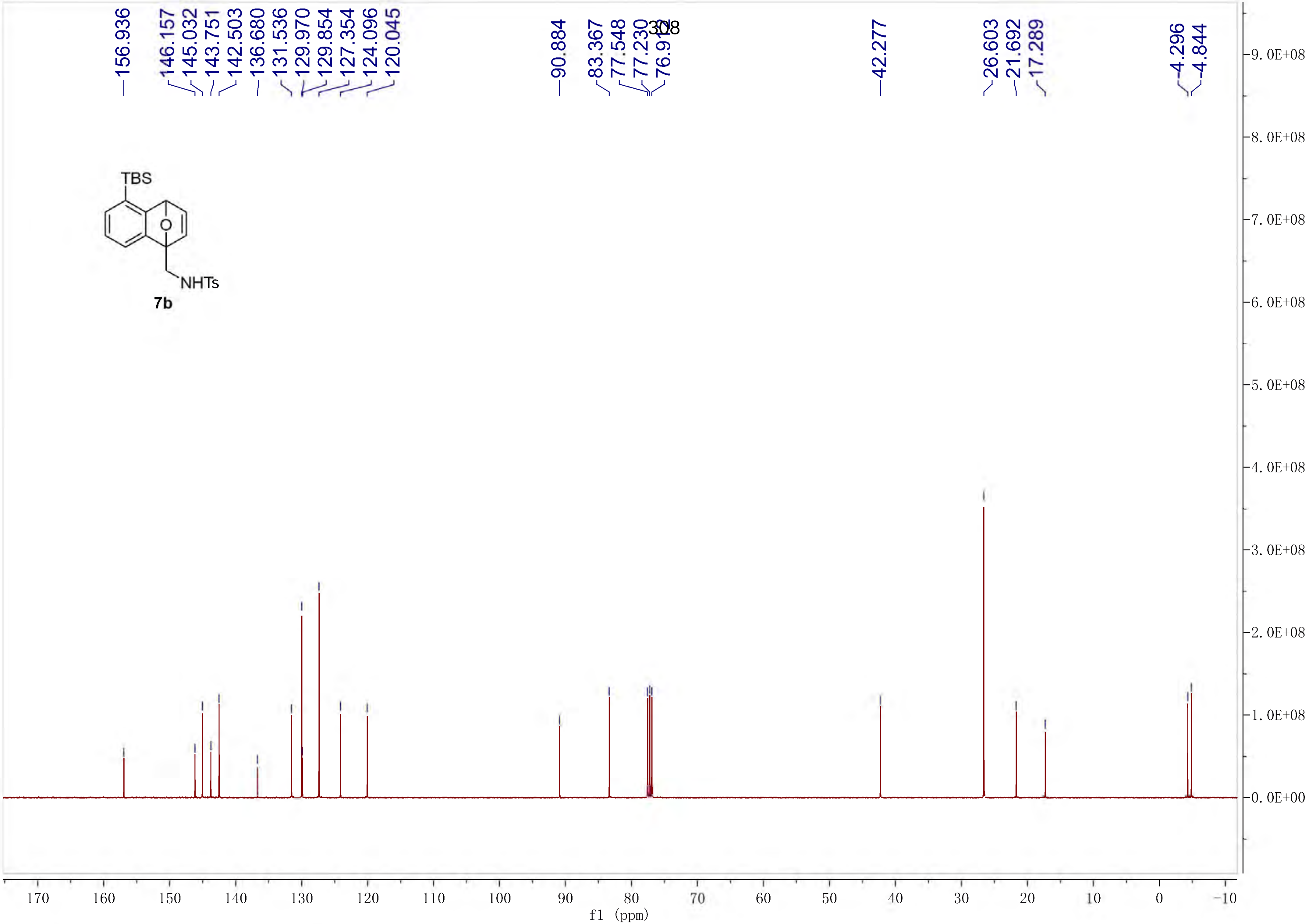

Supplementary Fig 236. ^{13}C NMR spectrum (400 MHz, $CDCl_3$, r.t.) of **7b**.

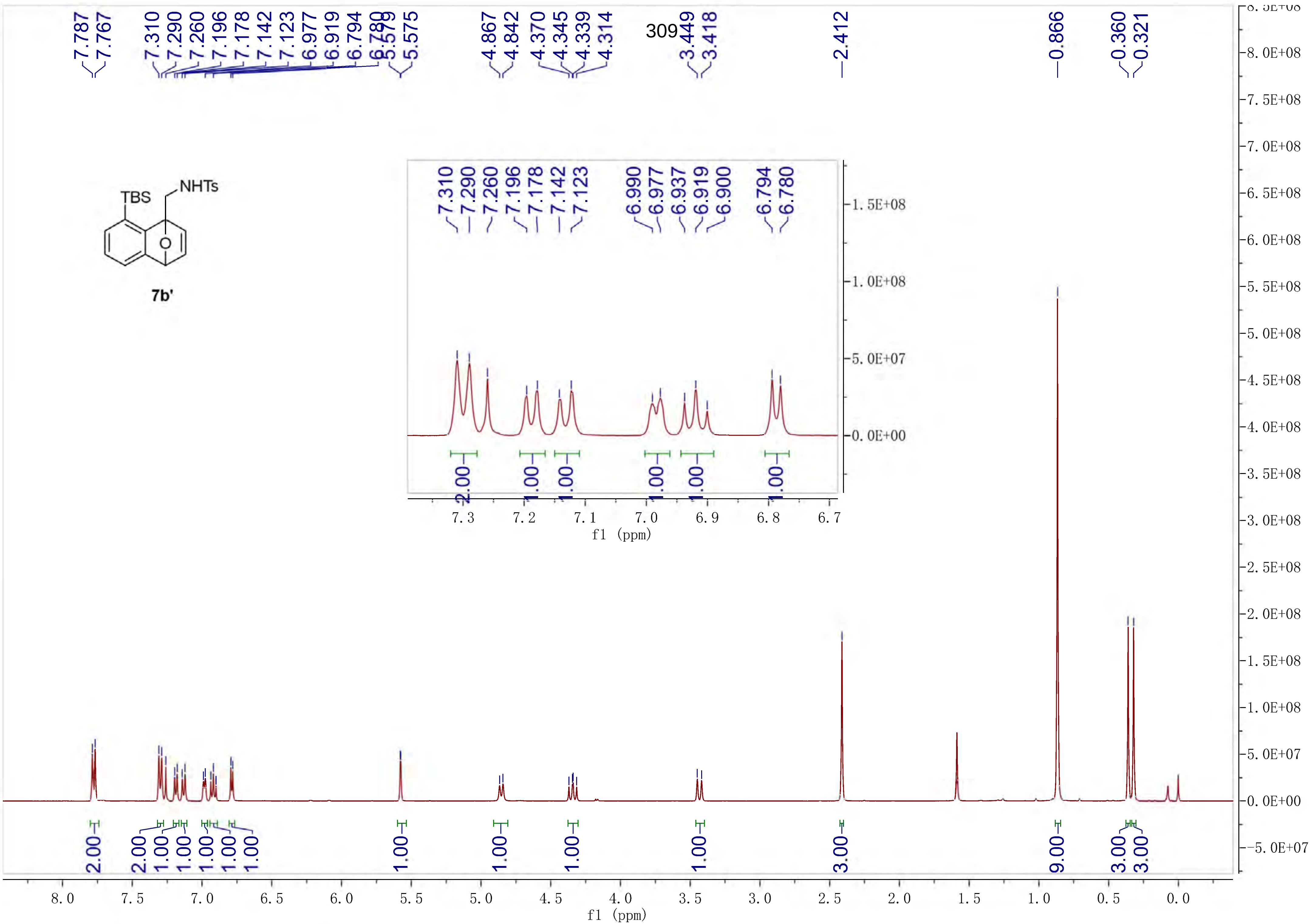

Supplementary Fig 237. ^{1}H NMR spectrum (400 MHz, $CDCl_3$, r.t.) of **7b'**.

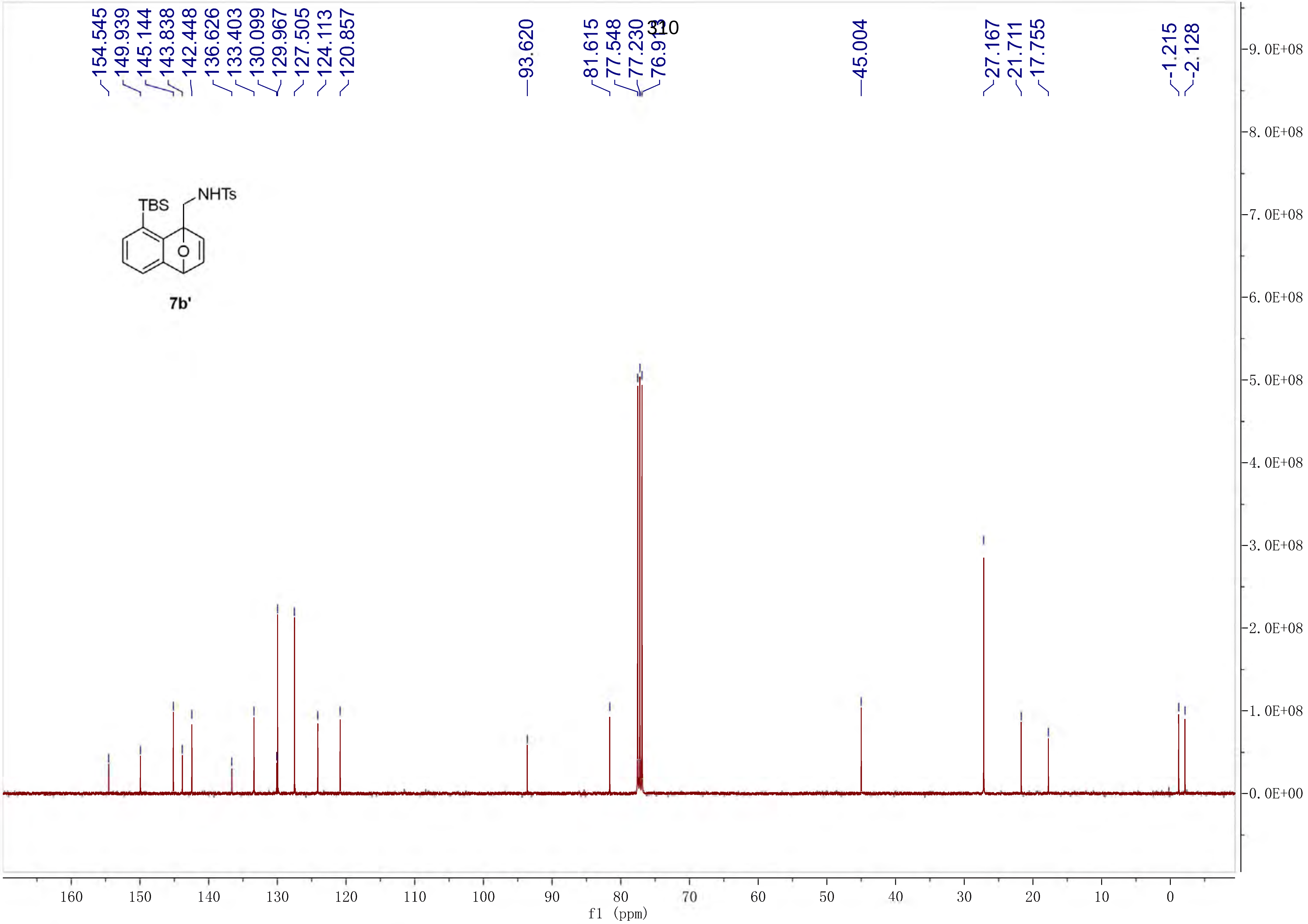

Supplementary Fig 238. ^{13}C NMR spectrum (400 MHz, $CDCl_3$, r.t.) of **7b’**.

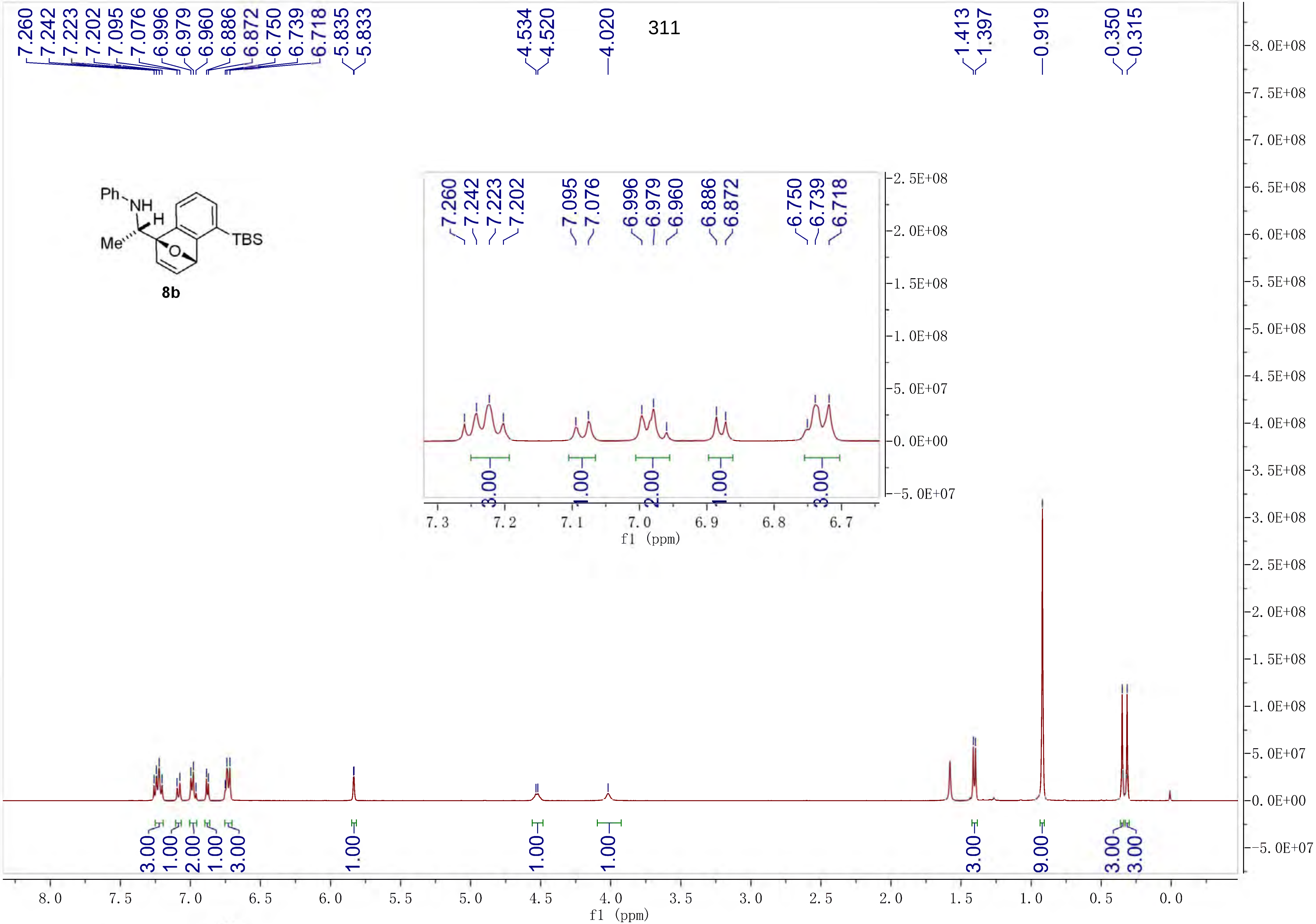

Supplementary Fig 239. ^{1}H NMR spectrum (400 MHz, $CDCl_3$, r.t.) of **8b**.

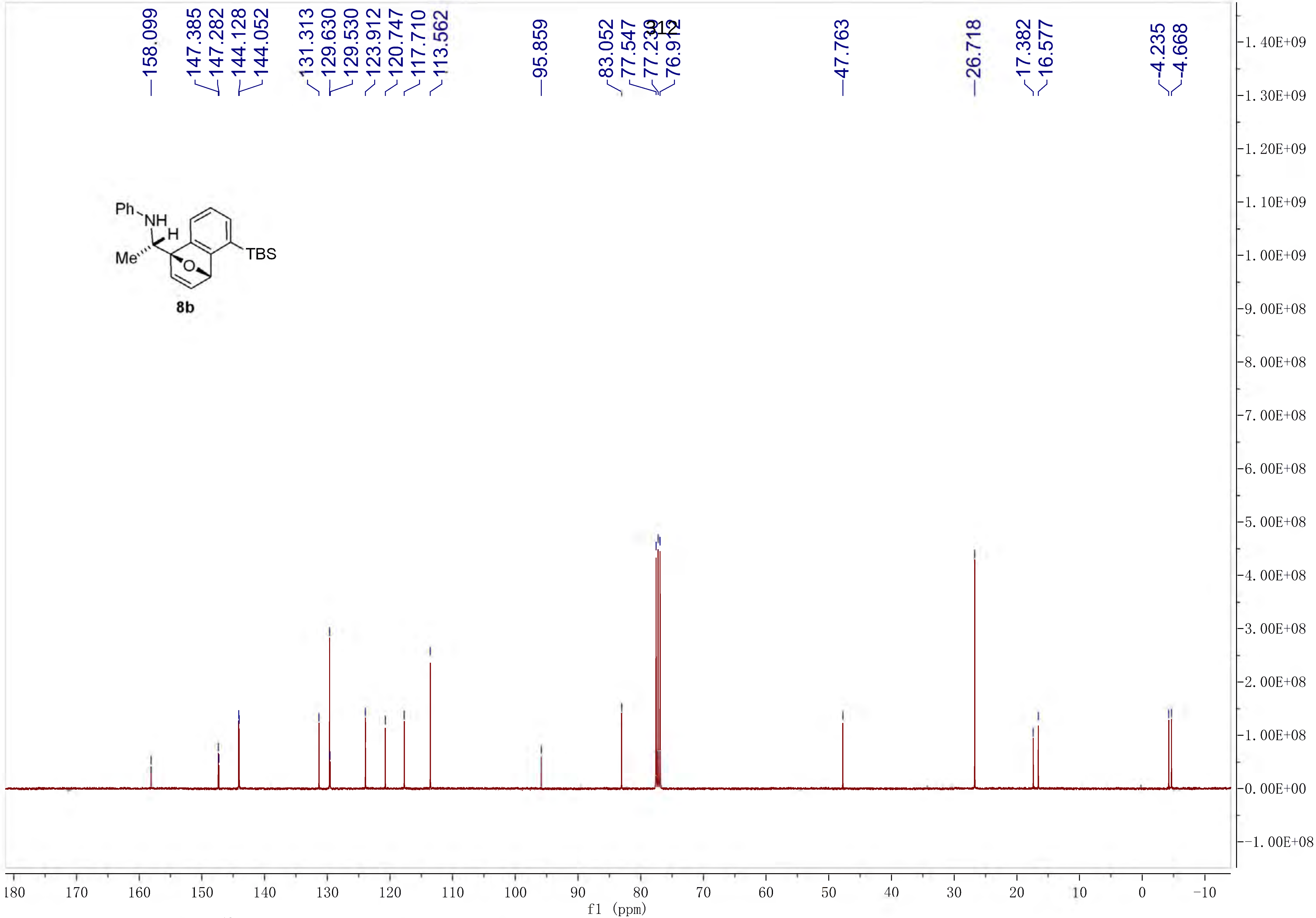

Supplementary Fig 240. ^{13}C NMR spectrum (400 MHz, $CDCl_3$, r.t.) of **8b**.

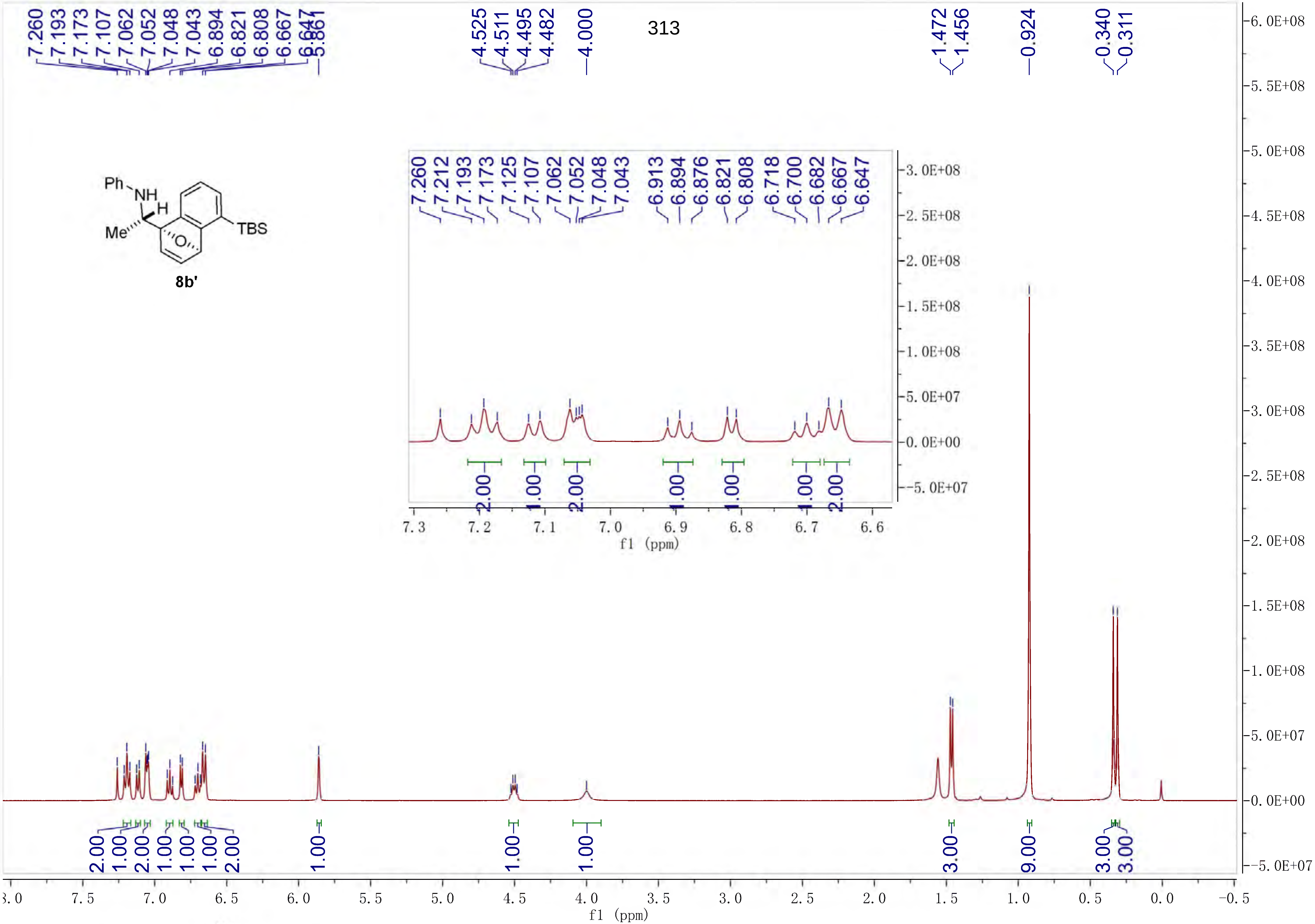

Supplementary Fig 241. ^{1}H NMR spectrum (400 MHz, $CDCl_3$, r.t.) of **8b’**.

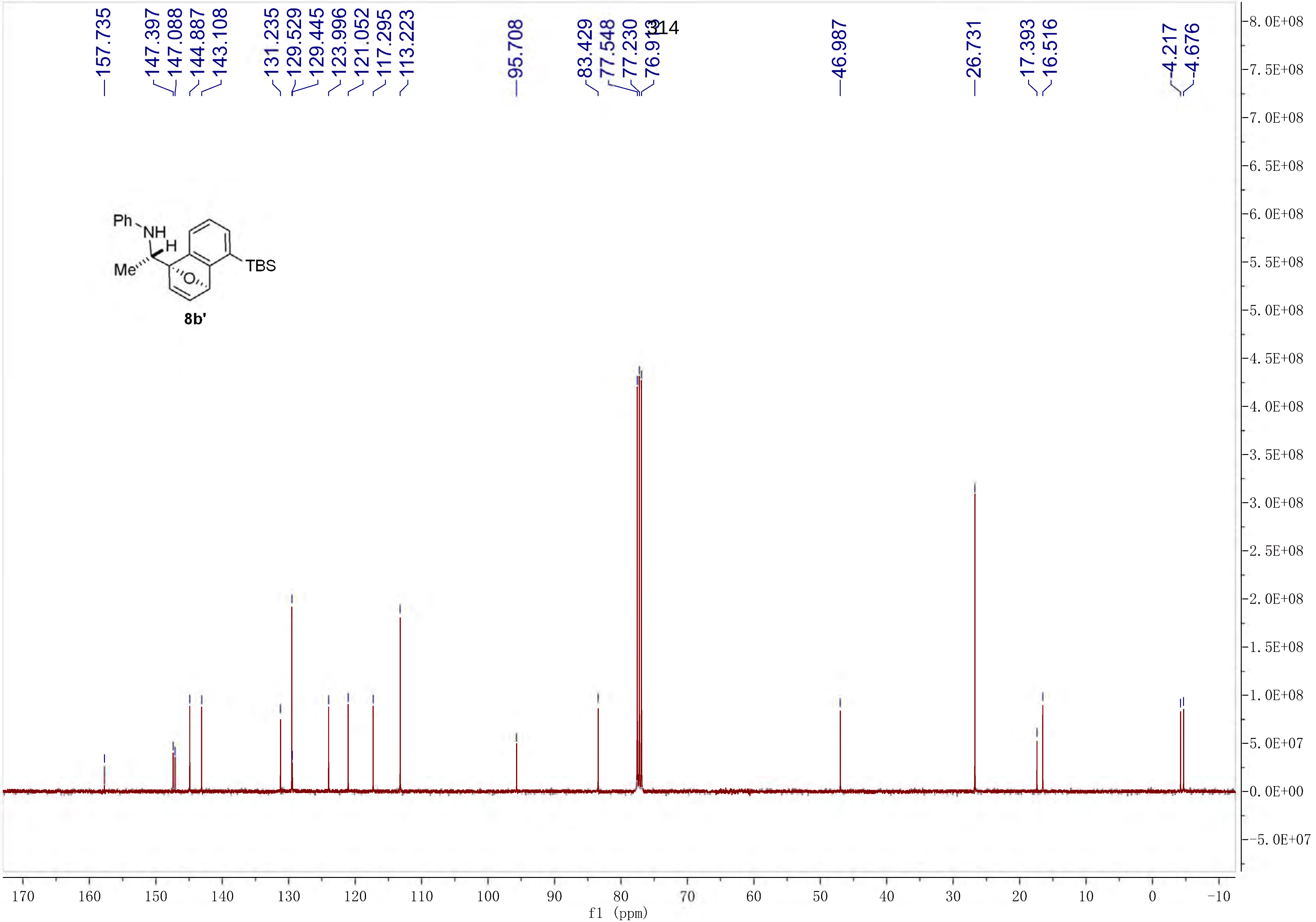

Supplementary Fig 242. ^{13}C NMR spectrum (400 MHz, $CDCl_3$, r.t.) of **8b'**.

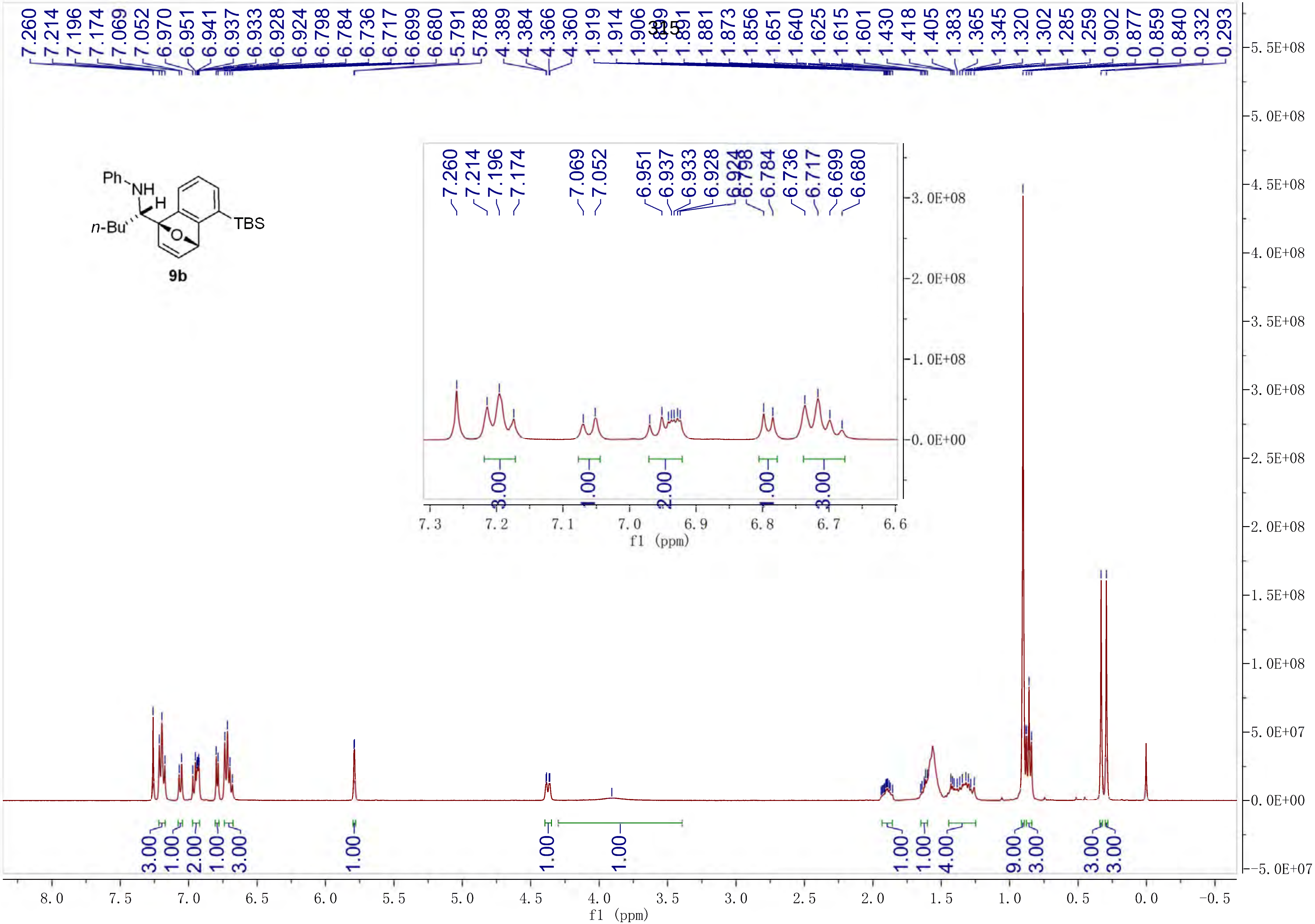

Supplementary Fig 243. ^{1}H NMR spectrum (400 MHz, $CDCl_3$, r.t.) of **9b**.

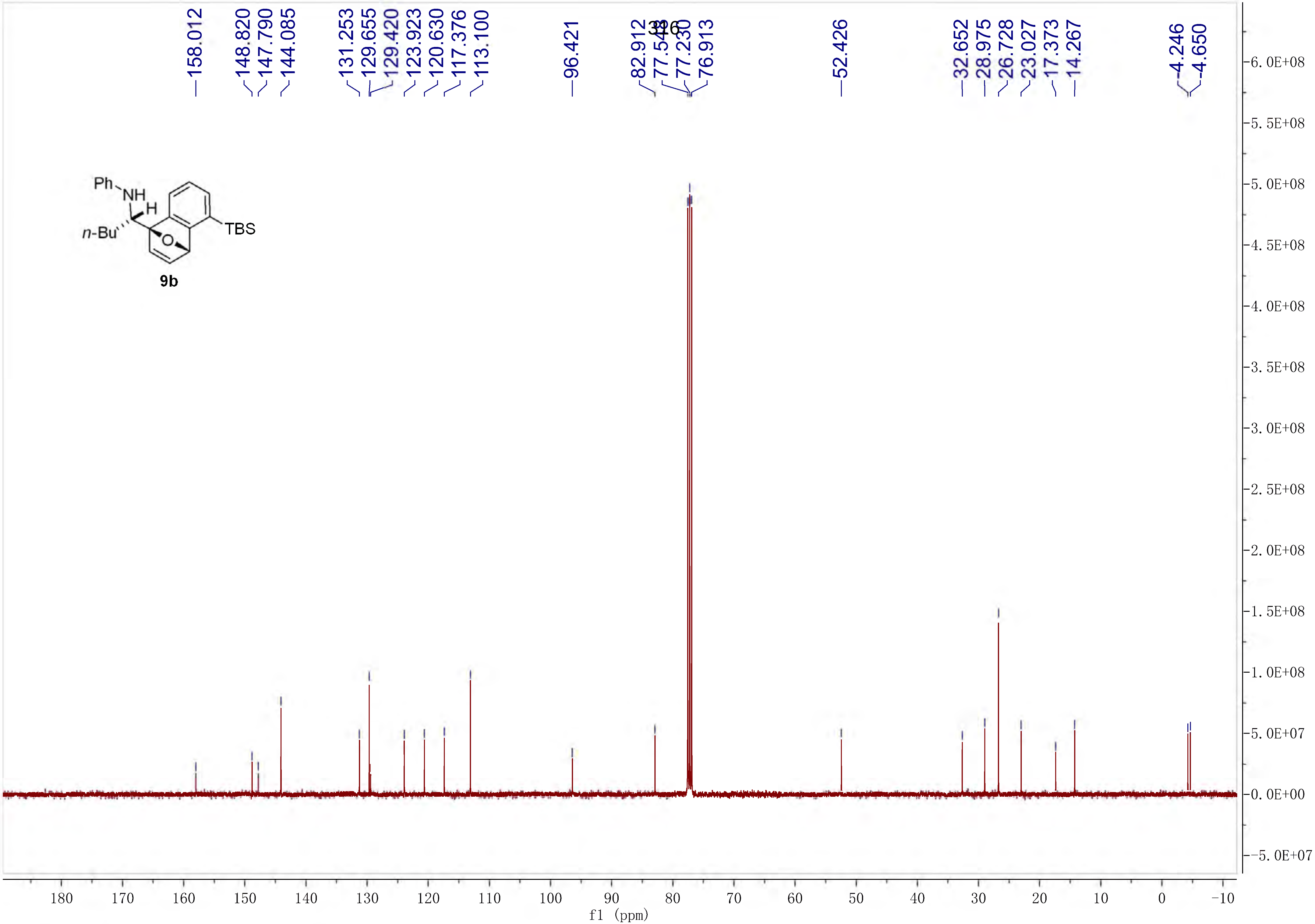

Supplementary Fig 244. ^{13}C NMR spectrum (400 MHz, $CDCl_3$, r.t.) of **9b**.

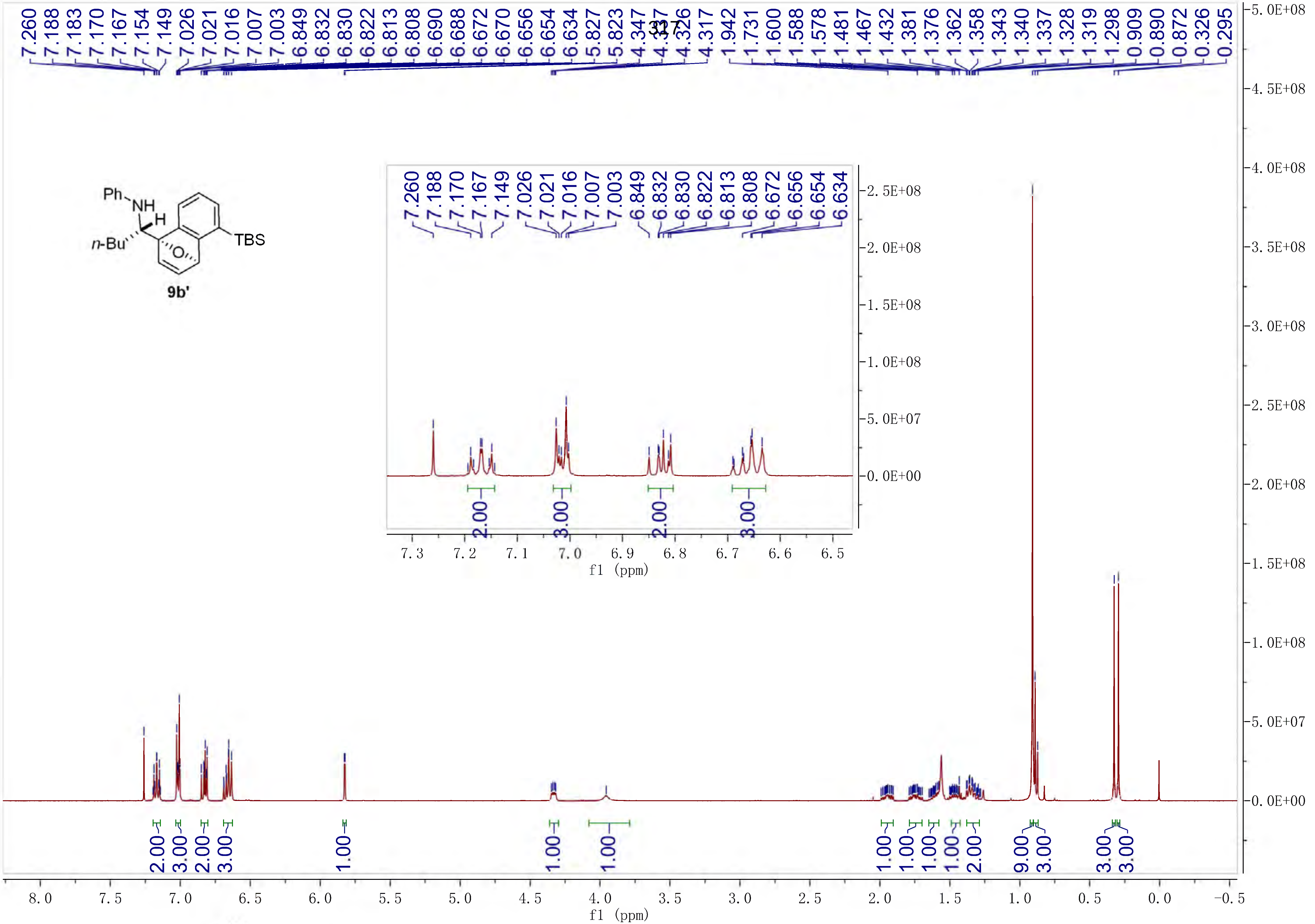

Supplementary Fig 245. ^{1}H NMR spectrum (400 MHz, $CDCl_3$, r.t.) of **9b’**.

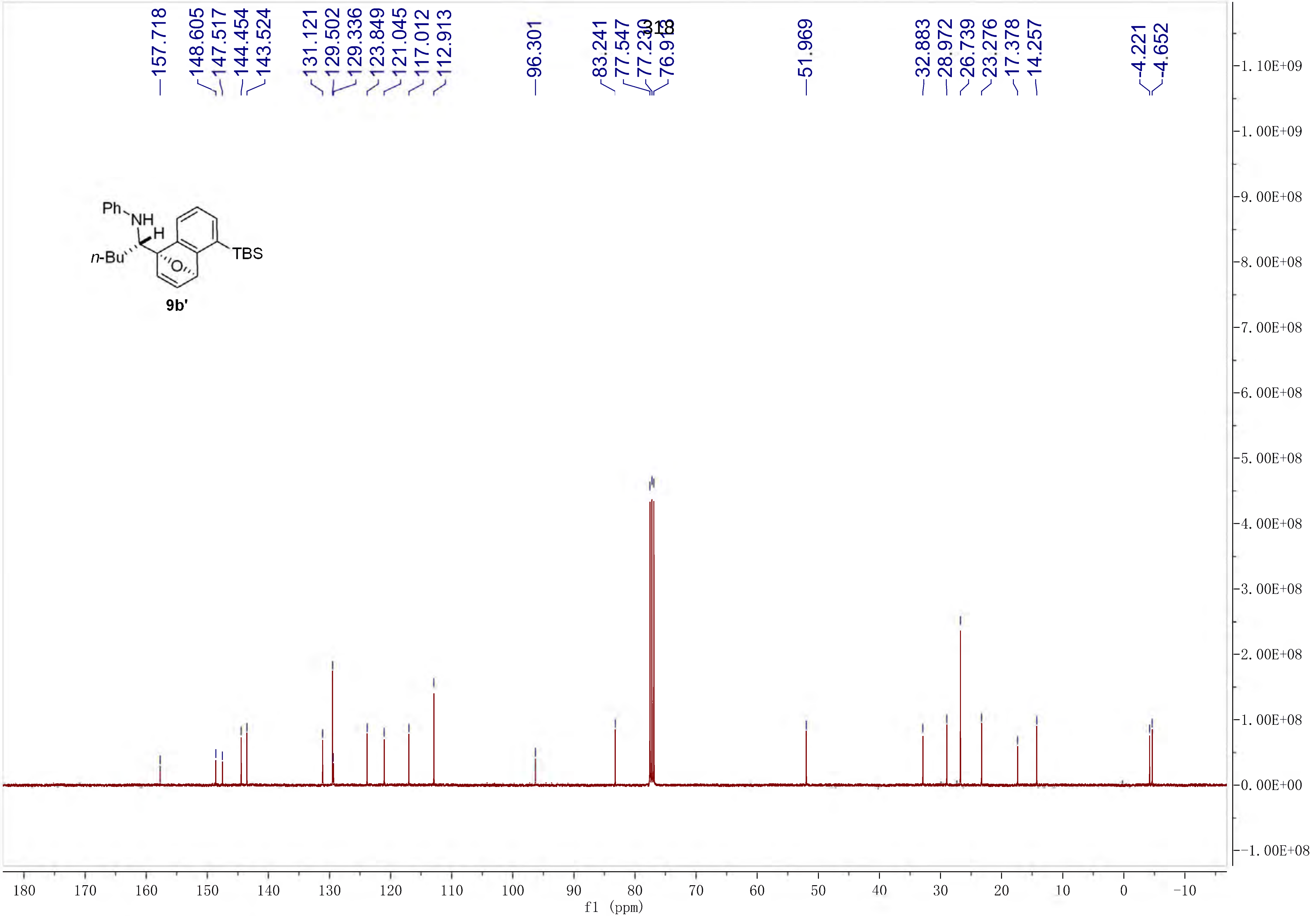

Supplementary Fig 246. ^{13}C NMR spectrum (400 MHz, $CDCl_3$, r.t.) of **9b’**.

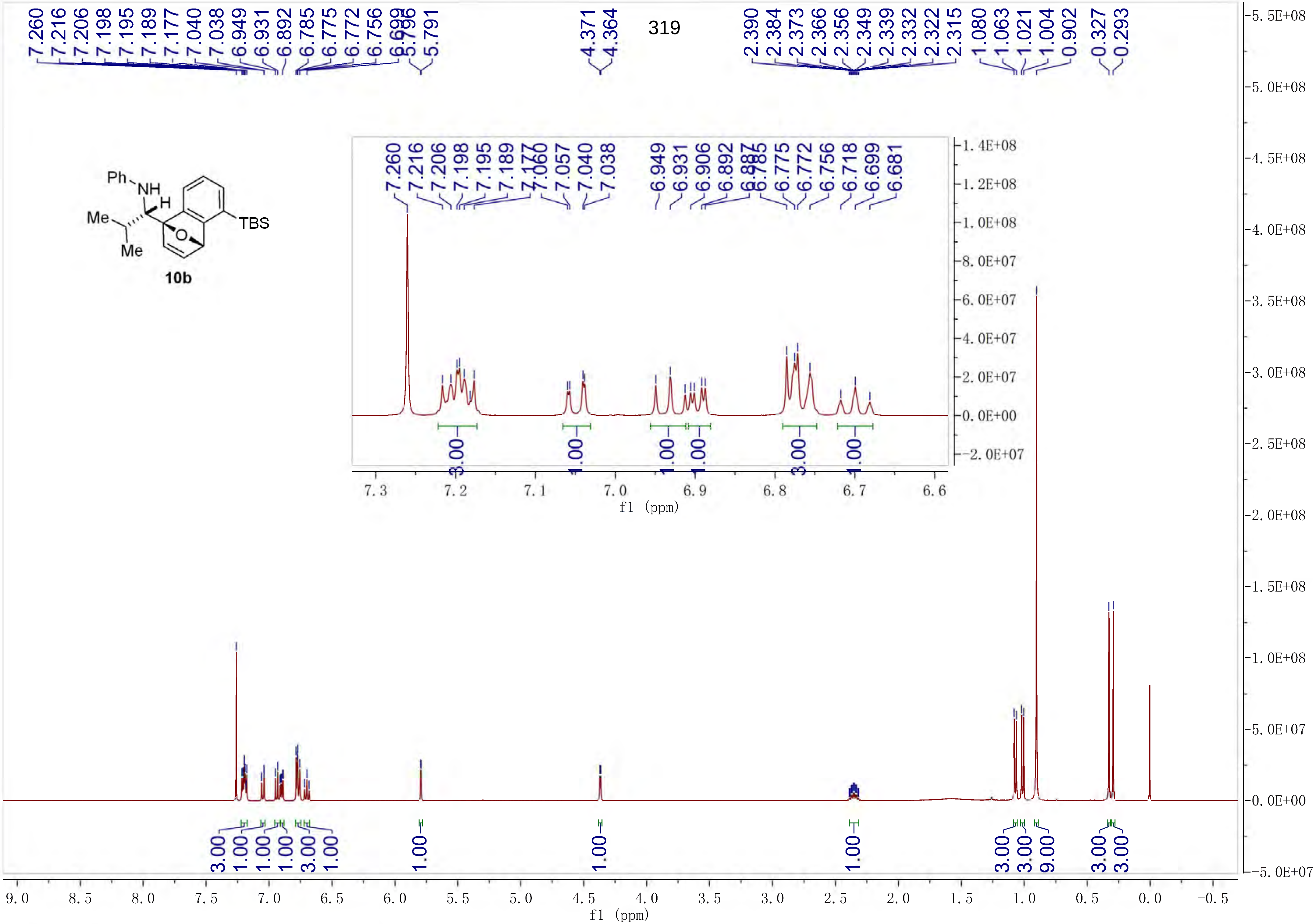

Supplementary Fig 247. ^{1}H NMR spectrum (400 MHz, $CDCl_3$, r.t.) of **10b**.

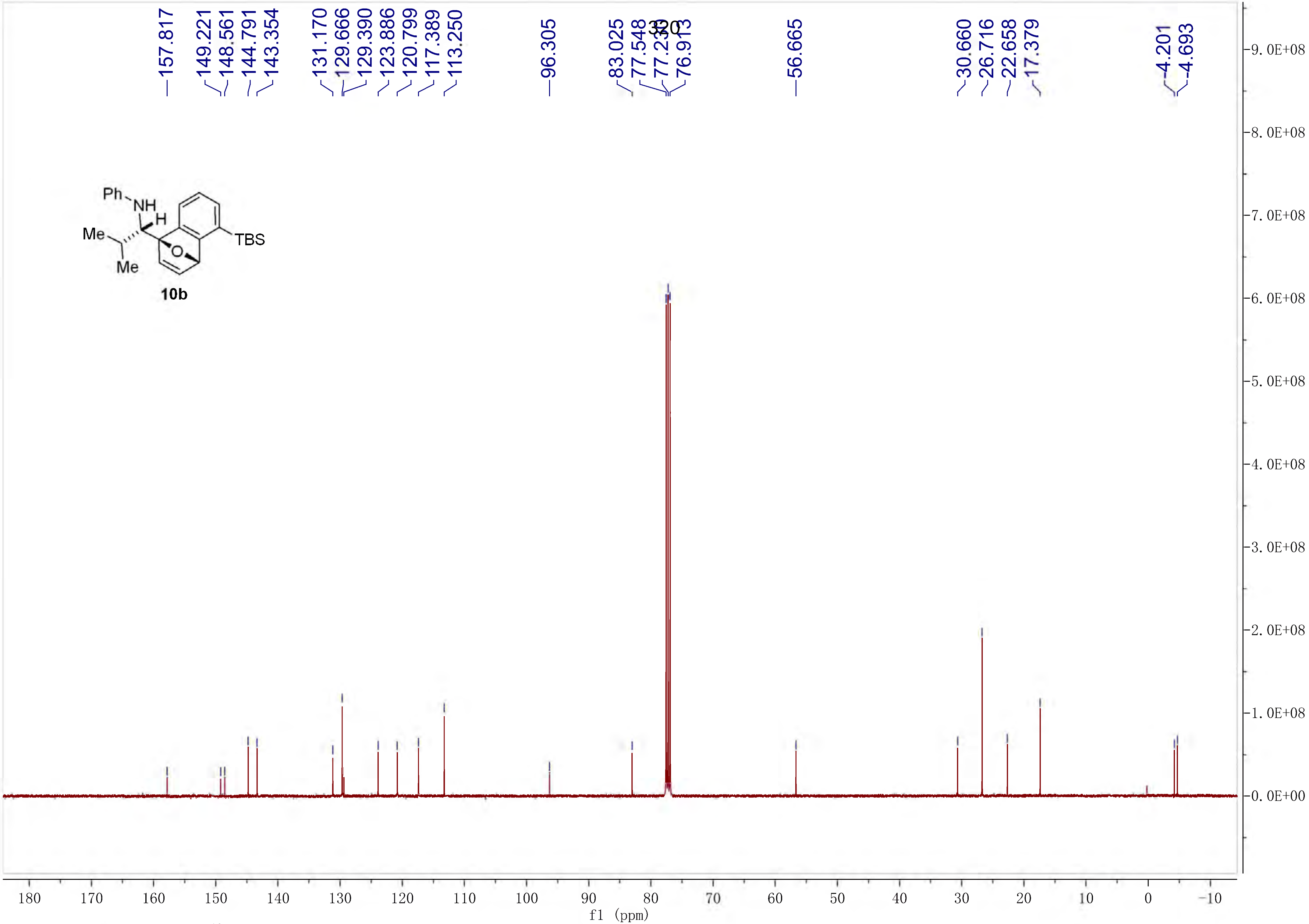

Supplementary Fig 248. ^{13}C NMR spectrum (400 MHz, $CDCl_3$, r.t.) of **10b**.

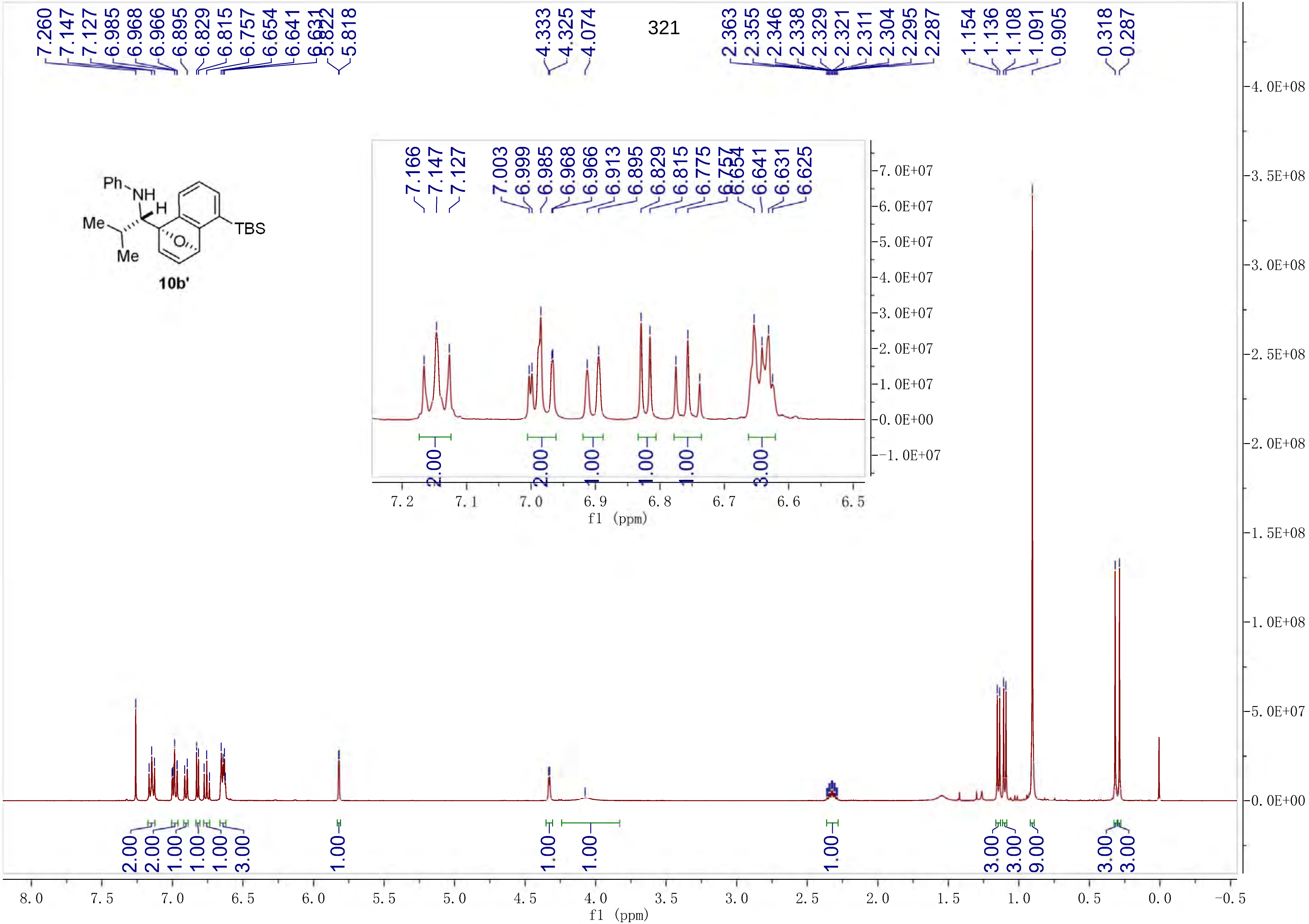

Supplementary Fig 249. ^{1}H NMR spectrum (400 MHz, $CDCl_3$, r.t.) of 1**0b'**.

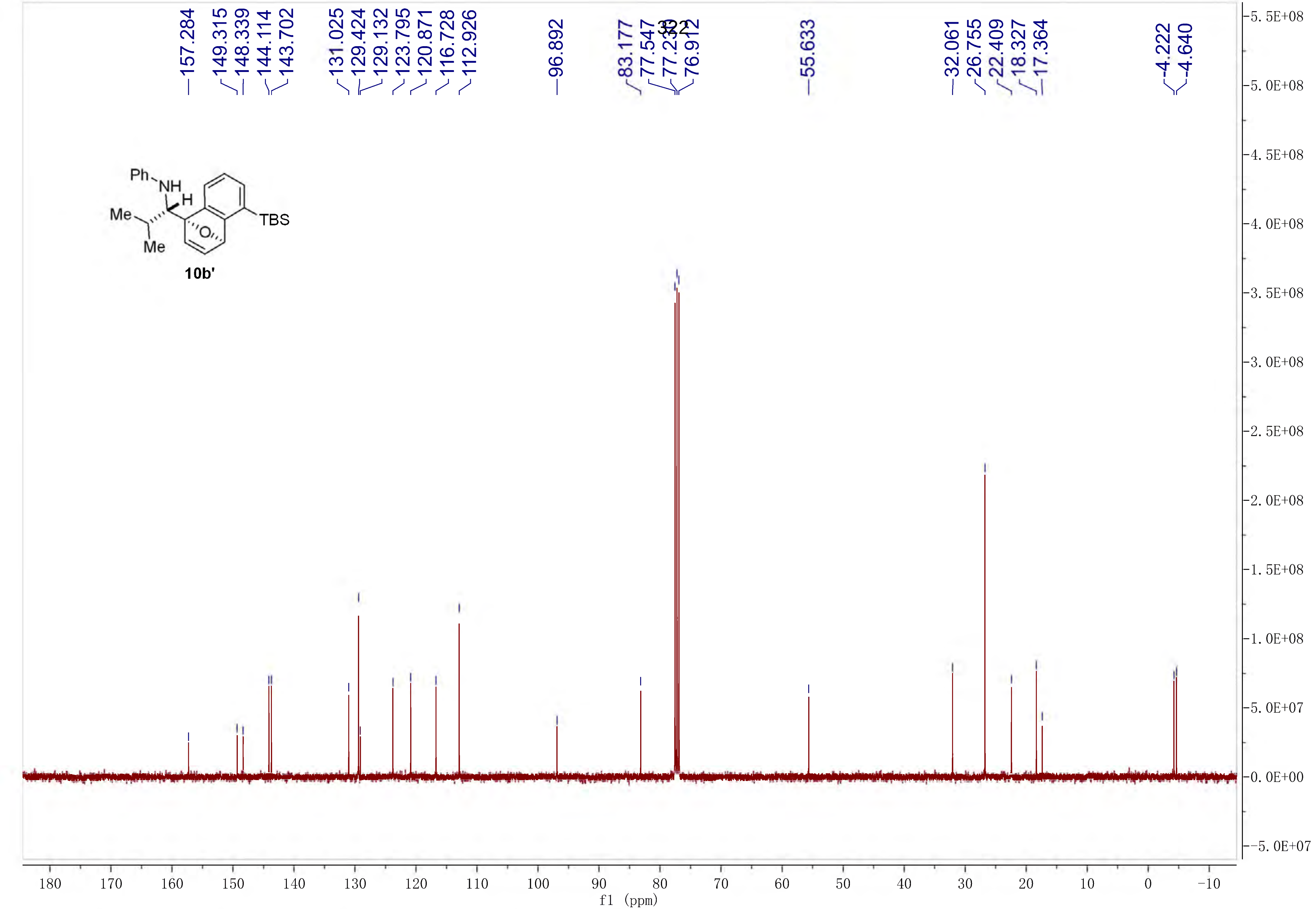

Supplementary Fig 250. ^{13}C NMR spectrum (400 MHz, $CDCl_3$, r.t.) of **10b’**.

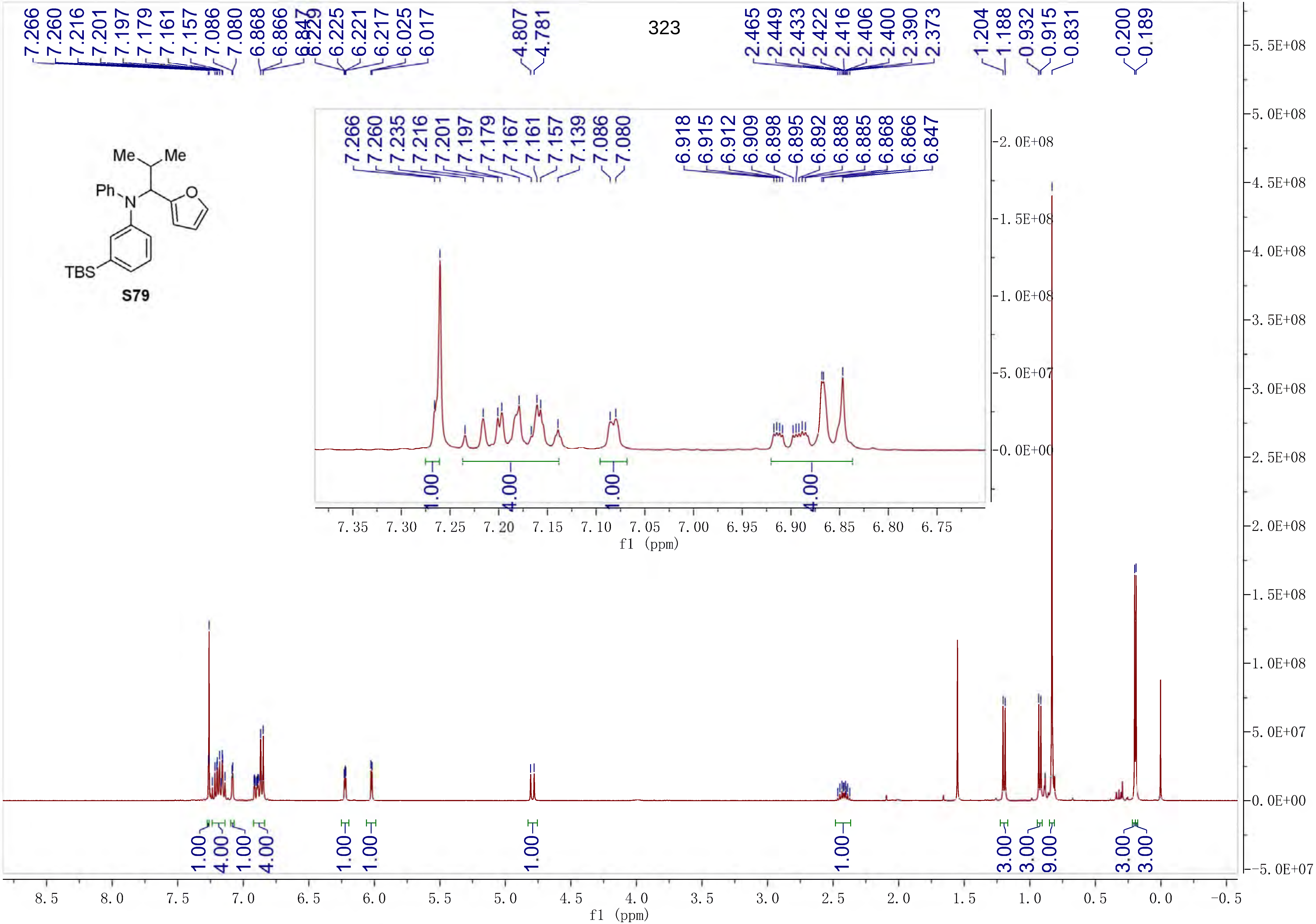

Supplementary Fig 251. ^{1}H NMR spectrum (400 MHz, $CDCl_3$, r.t.) of **S79**.

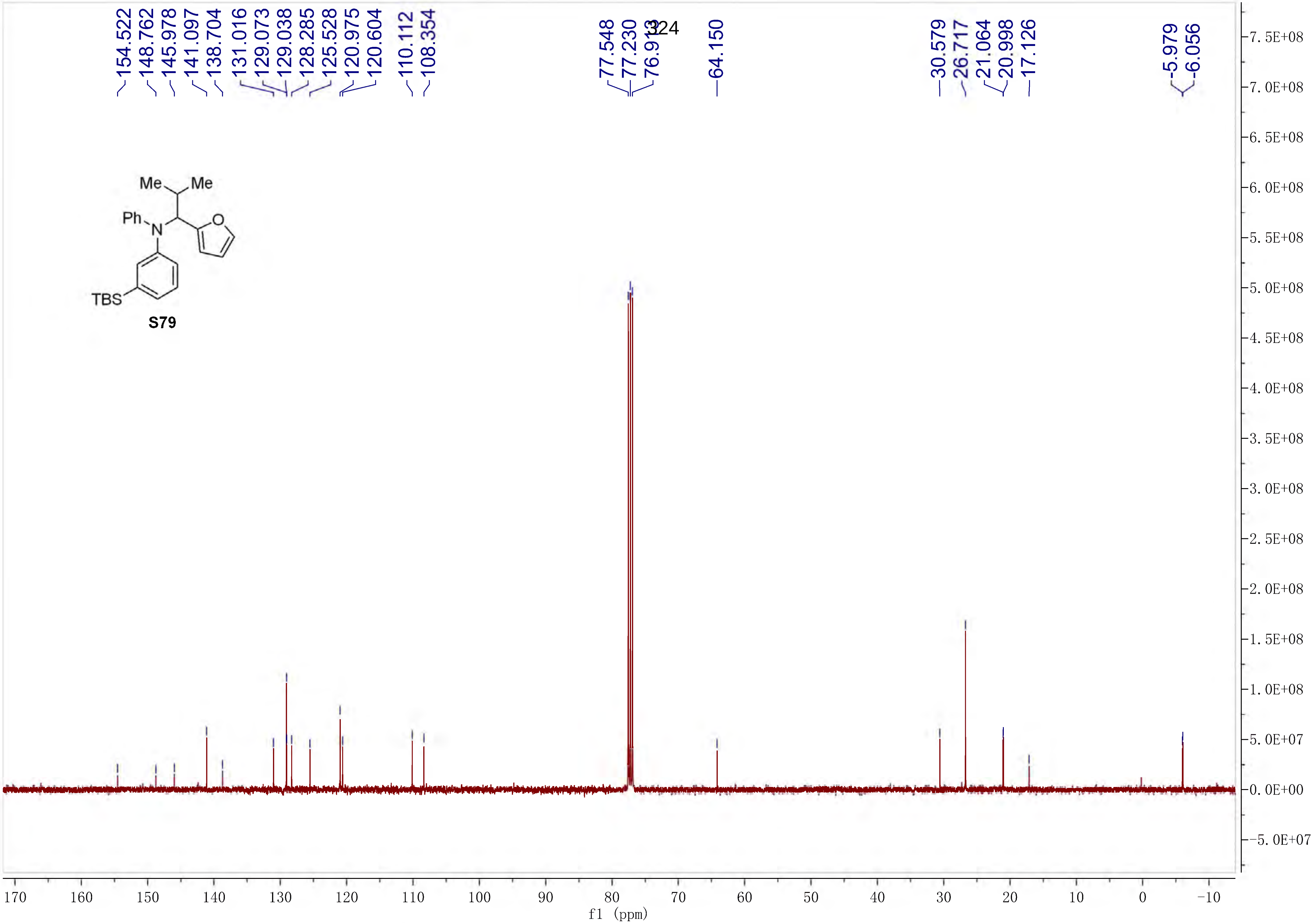

Supplementary Fig 252. ^{13}C NMR spectrum (400 MHz, $CDCl_3$, r.t.) of **S79**.

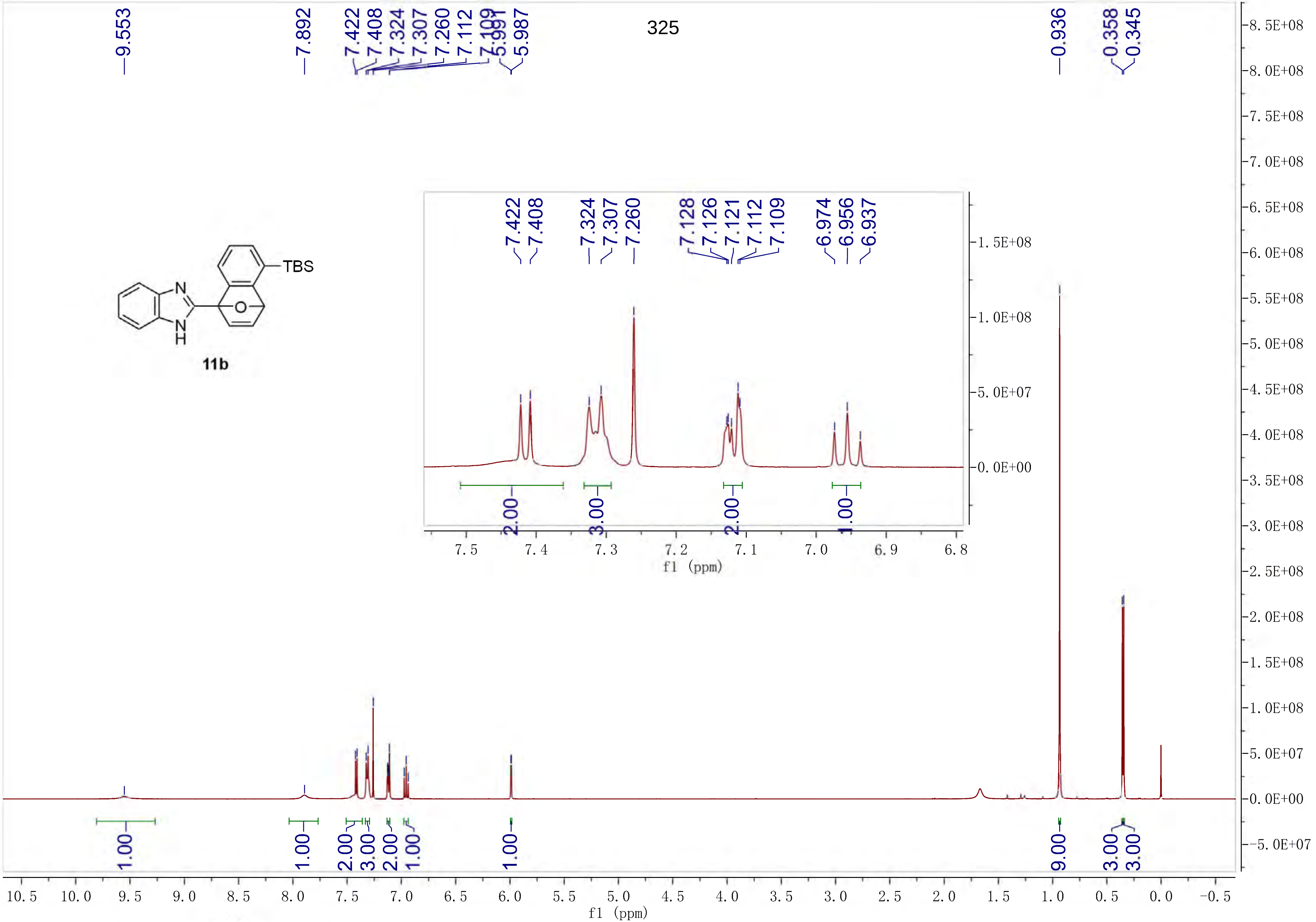

Supplementary Fig 253. ^{1}H NMR spectrum (400 MHz, $CDCl_3$, r.t.) of **11b**.

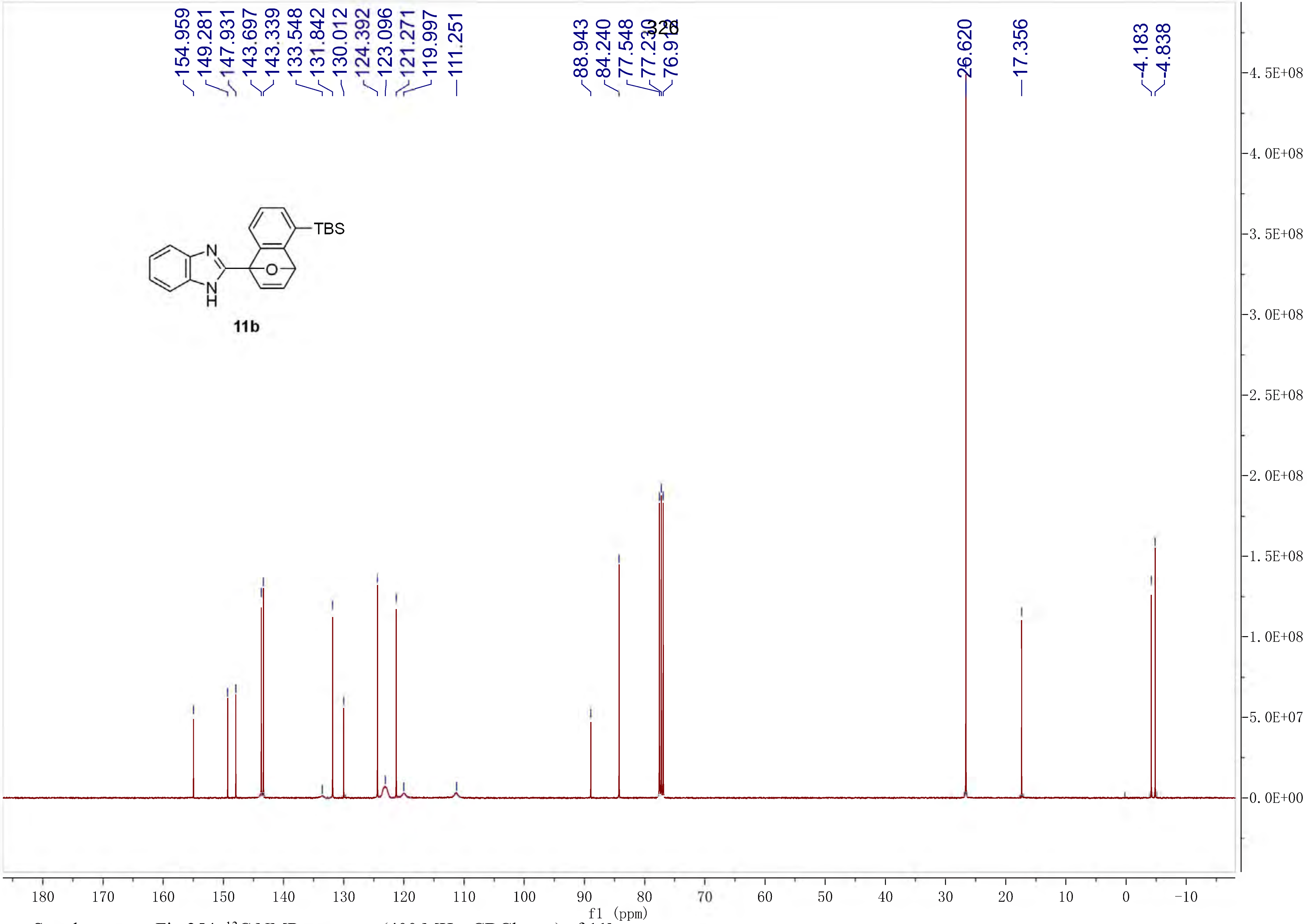

Supplementary Fig 254. ^{13}C NMR spectrum (400 MHz, $CDCl_3$, r.t.) of **11b**.

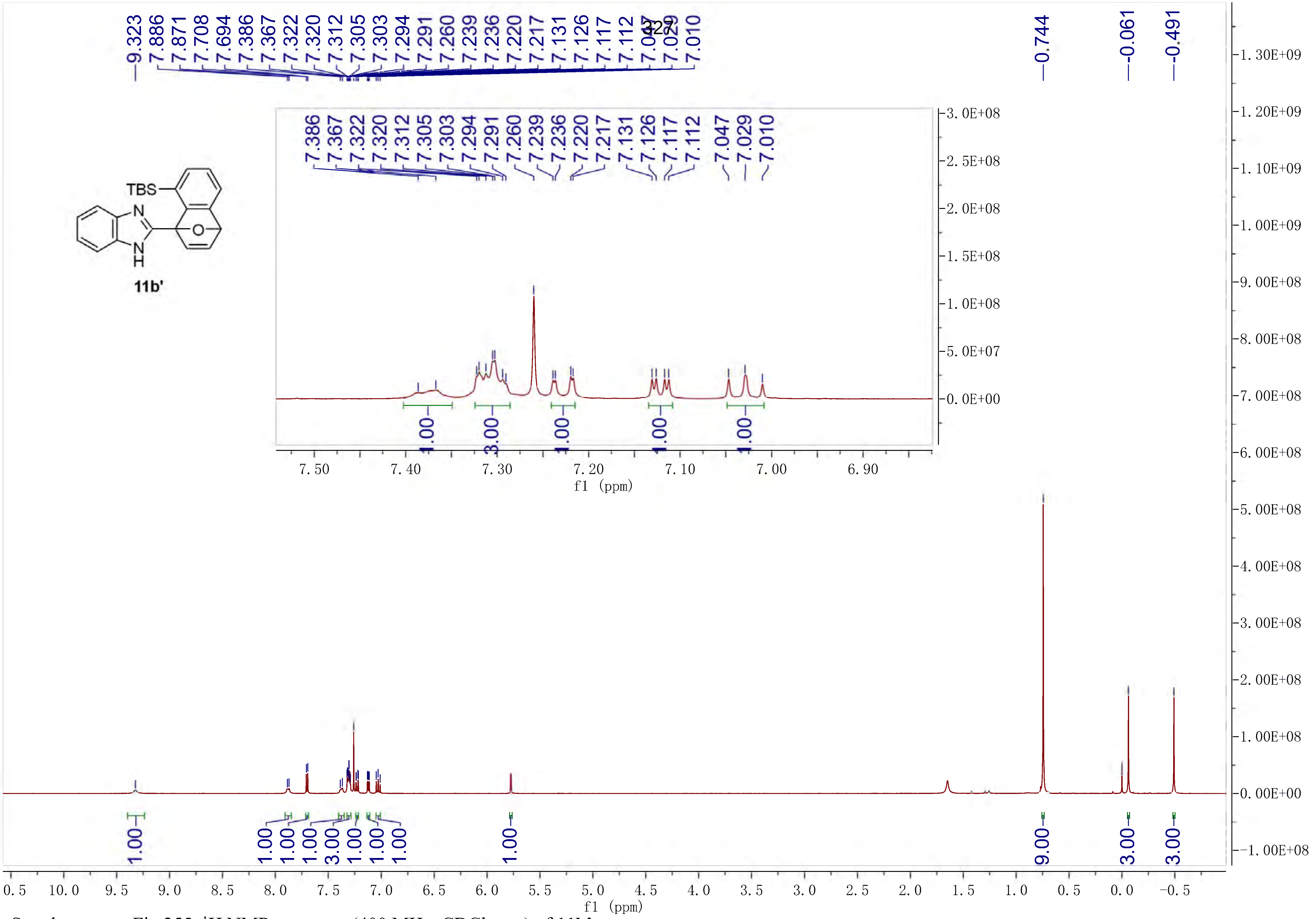

Supplementary Fig 255. ^{1}H NMR spectrum (400 MHz, $CDCl_3$, r.t.) of **11b’**.

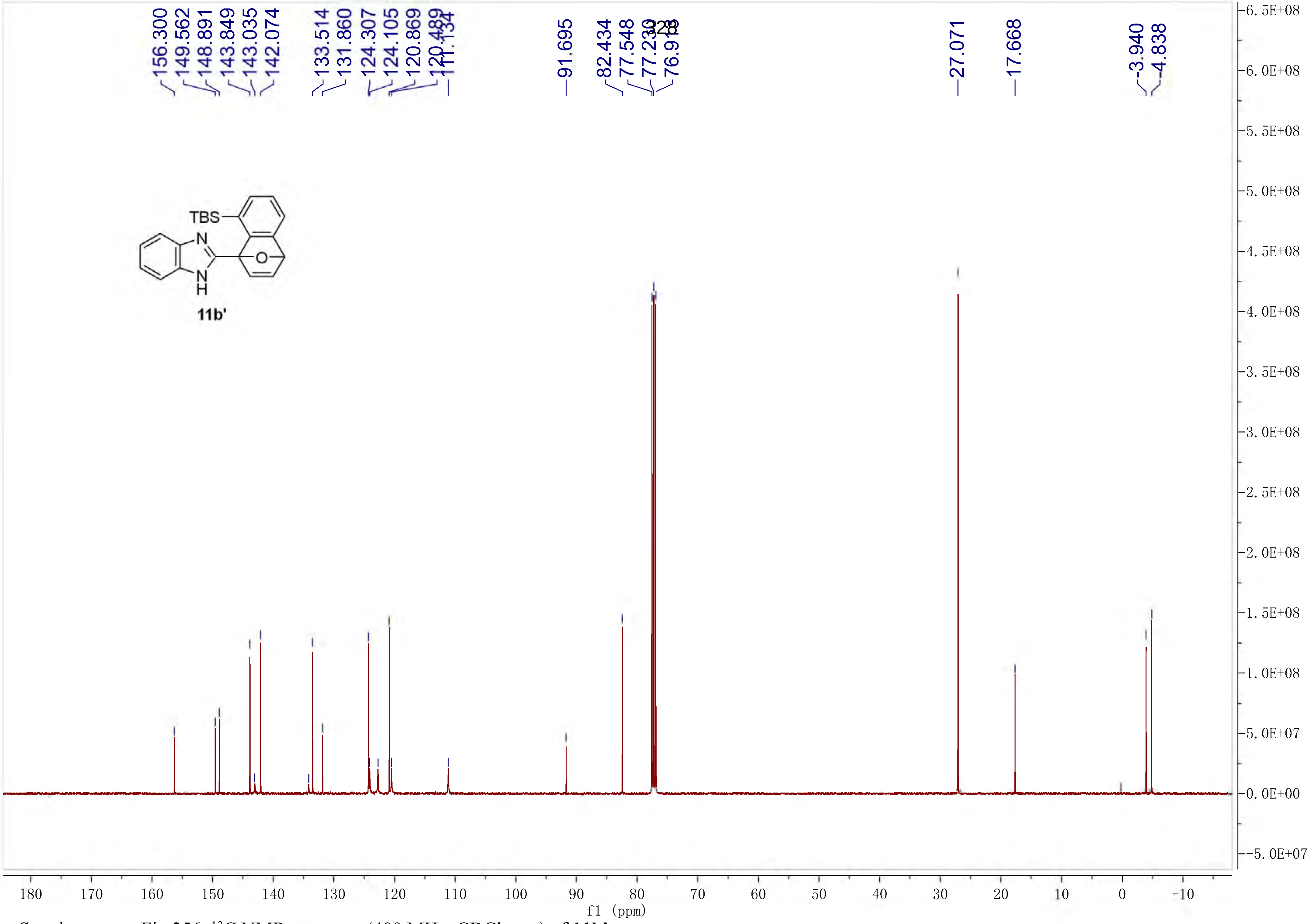

Supplementary Fig 256. ^{13}C NMR spectrum (400 MHz, $CDCl_3$, r.t.) of **11b'**.

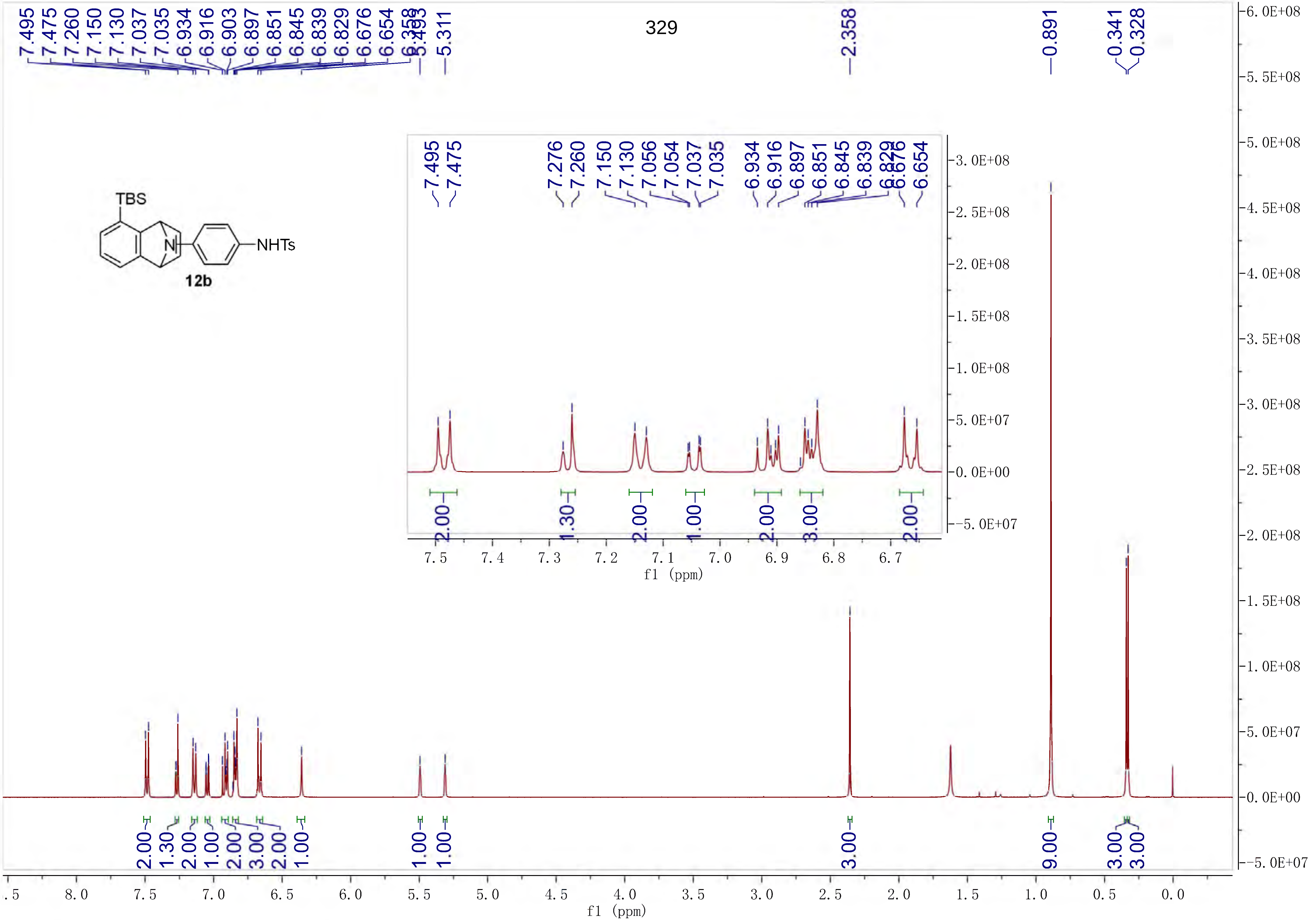

Supplementary Fig 257. ^{1}H NMR spectrum (400 MHz, $CDCl_3$, r.t.) of **12b**.

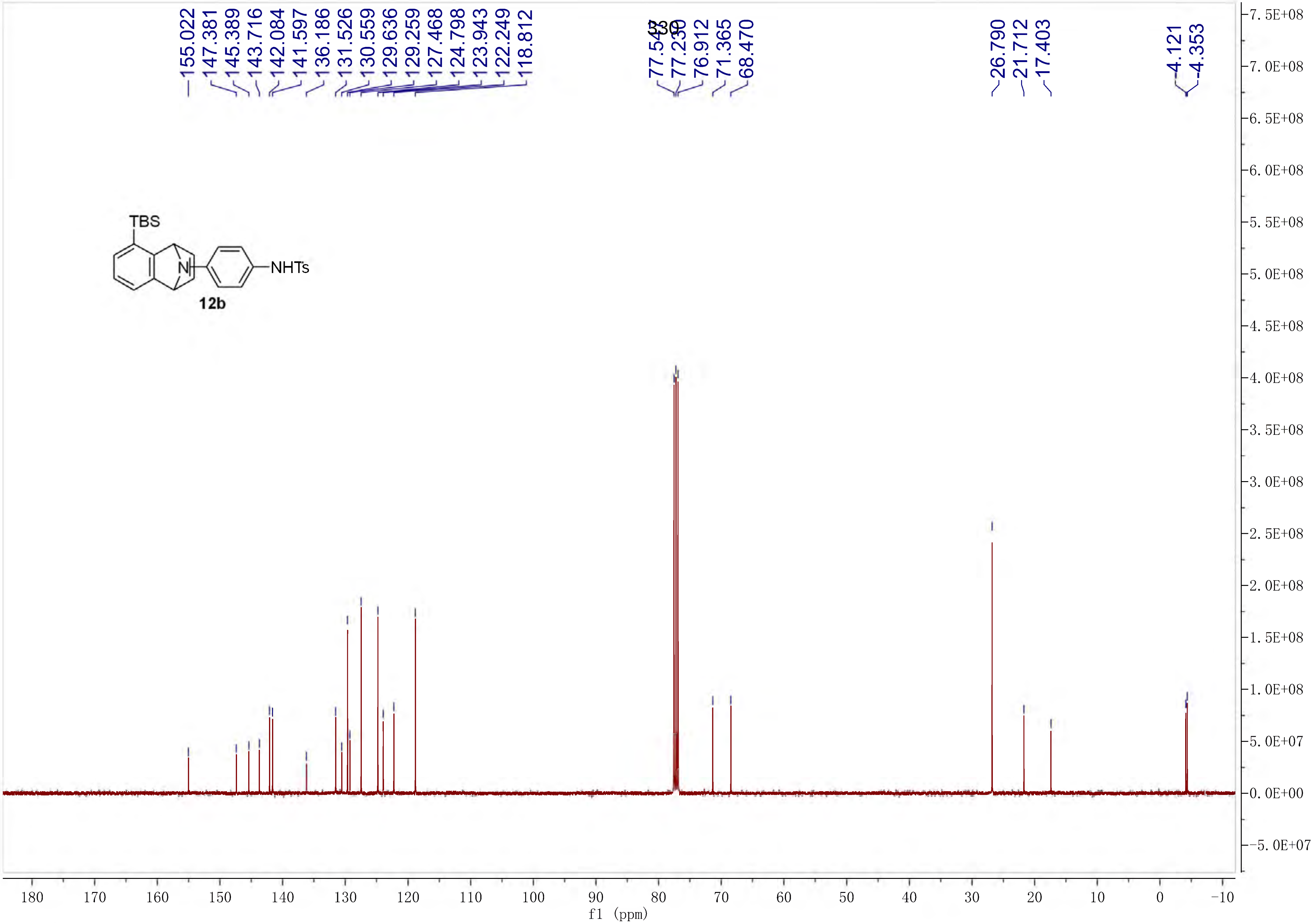

Supplementary Fig 258. ^{13}C NMR spectrum (400 MHz, $CDCl_3$, r.t.) of **12b**.

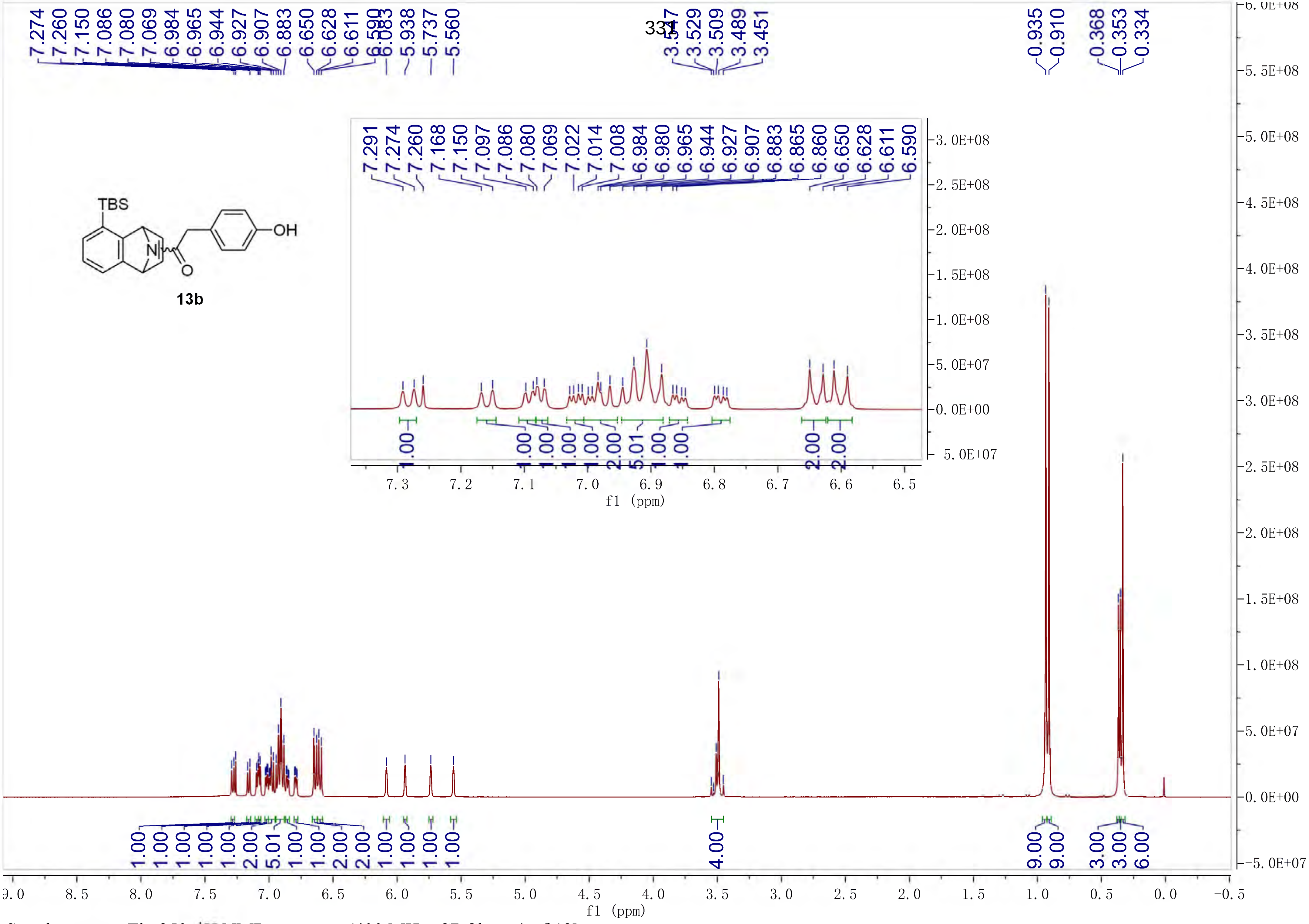

Supplementary Fig 259. ^{1}H NMR spectrum (400 MHz, $CDCl_3$, r.t.) of **13b**.

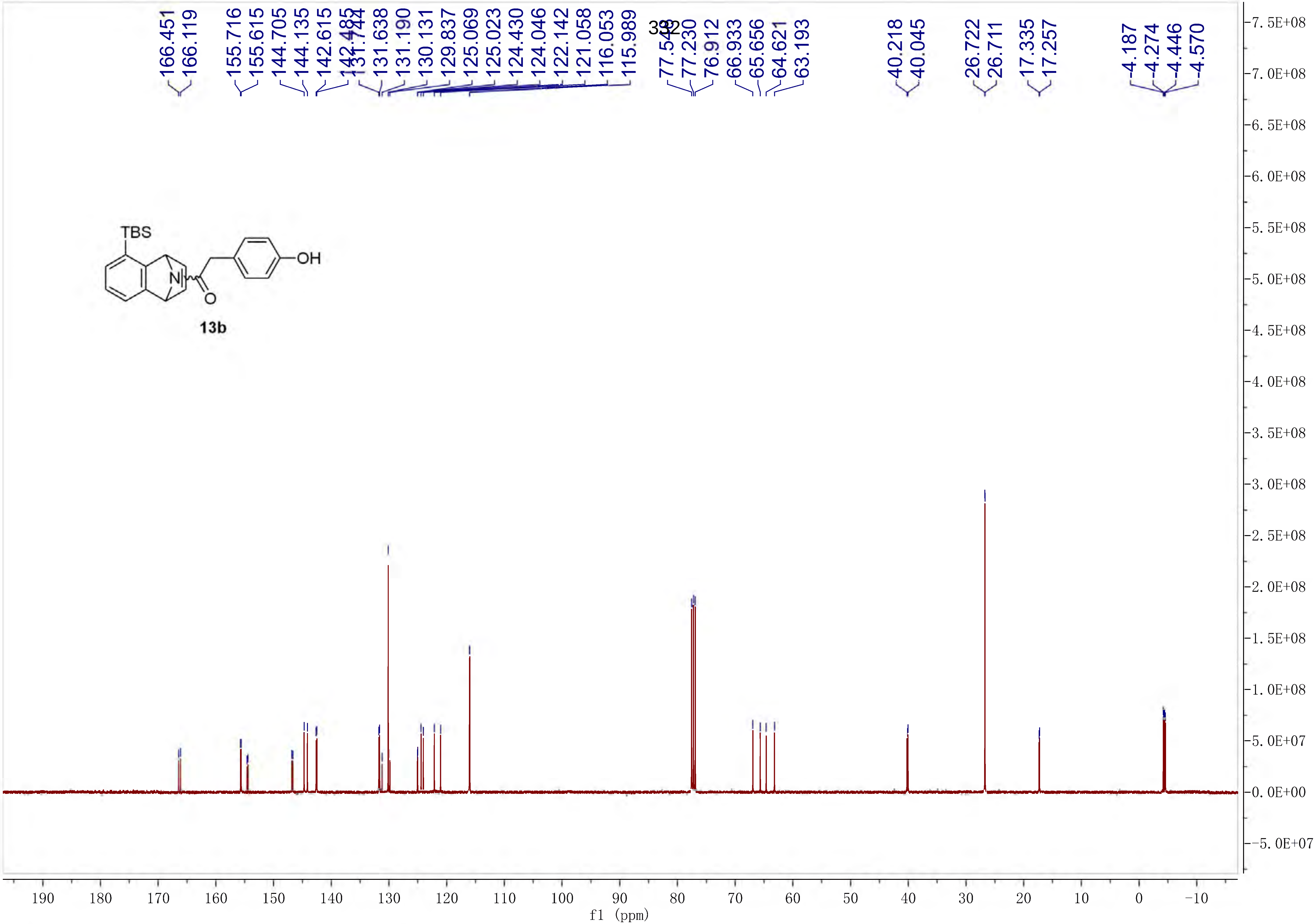

Supplementary Fig 260. ^{13}C NMR spectrum (400 MHz, $CDCl_3$, r.t.) of **13b**.

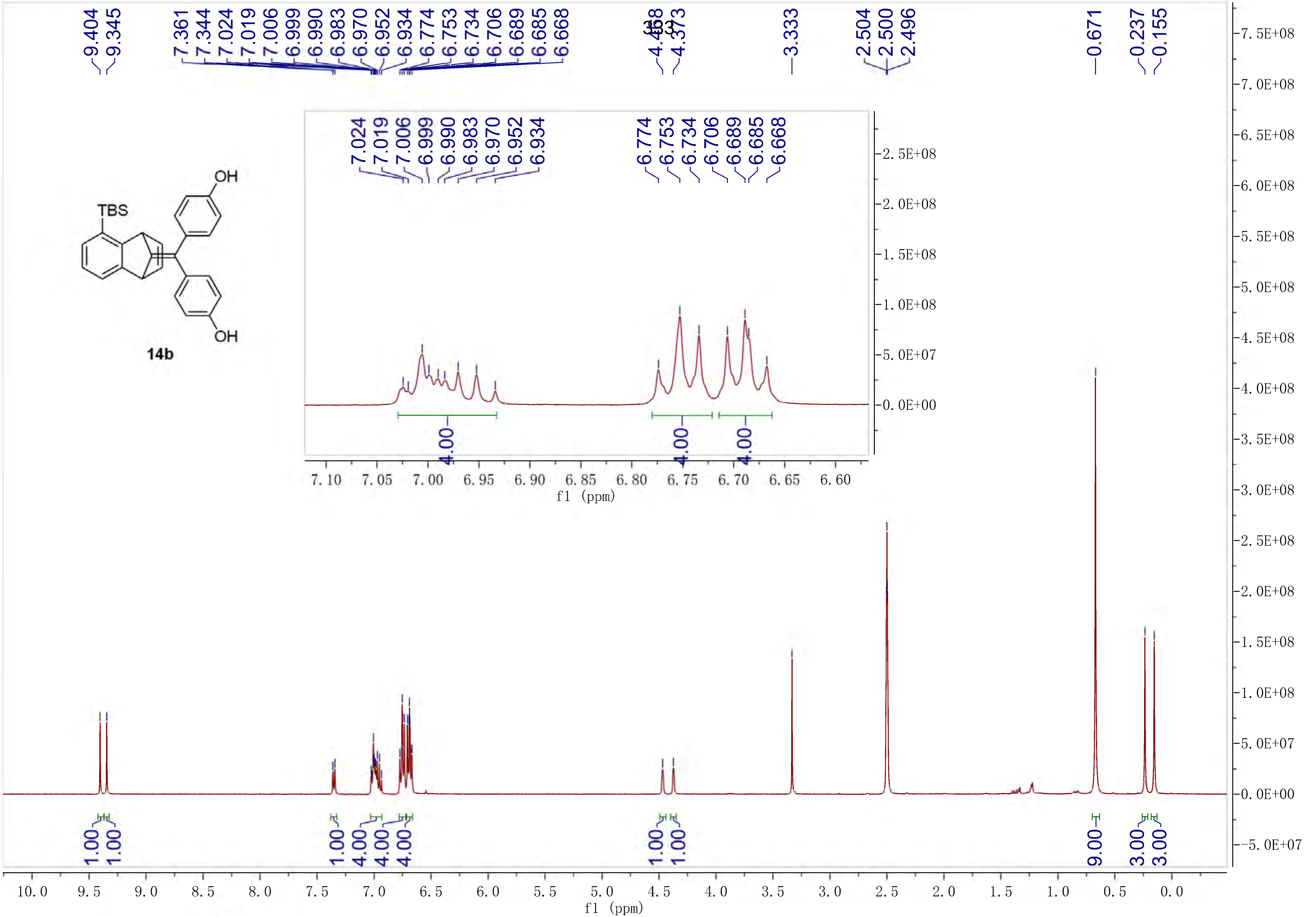

Supplementary Fig 261. ^{1}H NMR spectrum (400 MHz, $CDCl_3$, r.t.) of **14b**.

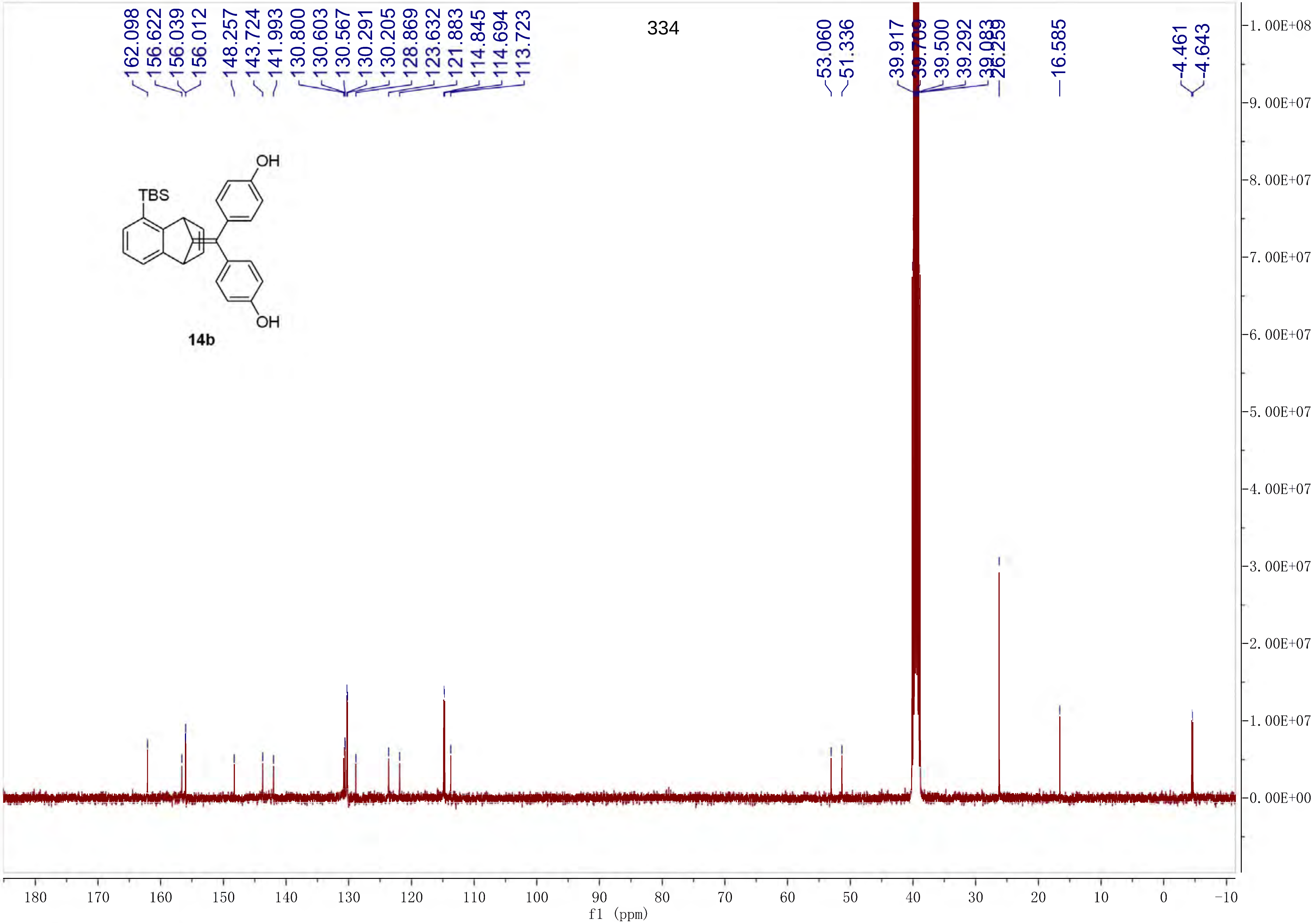

Supplementary Fig 262. ^{13}C NMR spectrum (400 MHz, $CDCl_3$, r.t.) of **14b**.

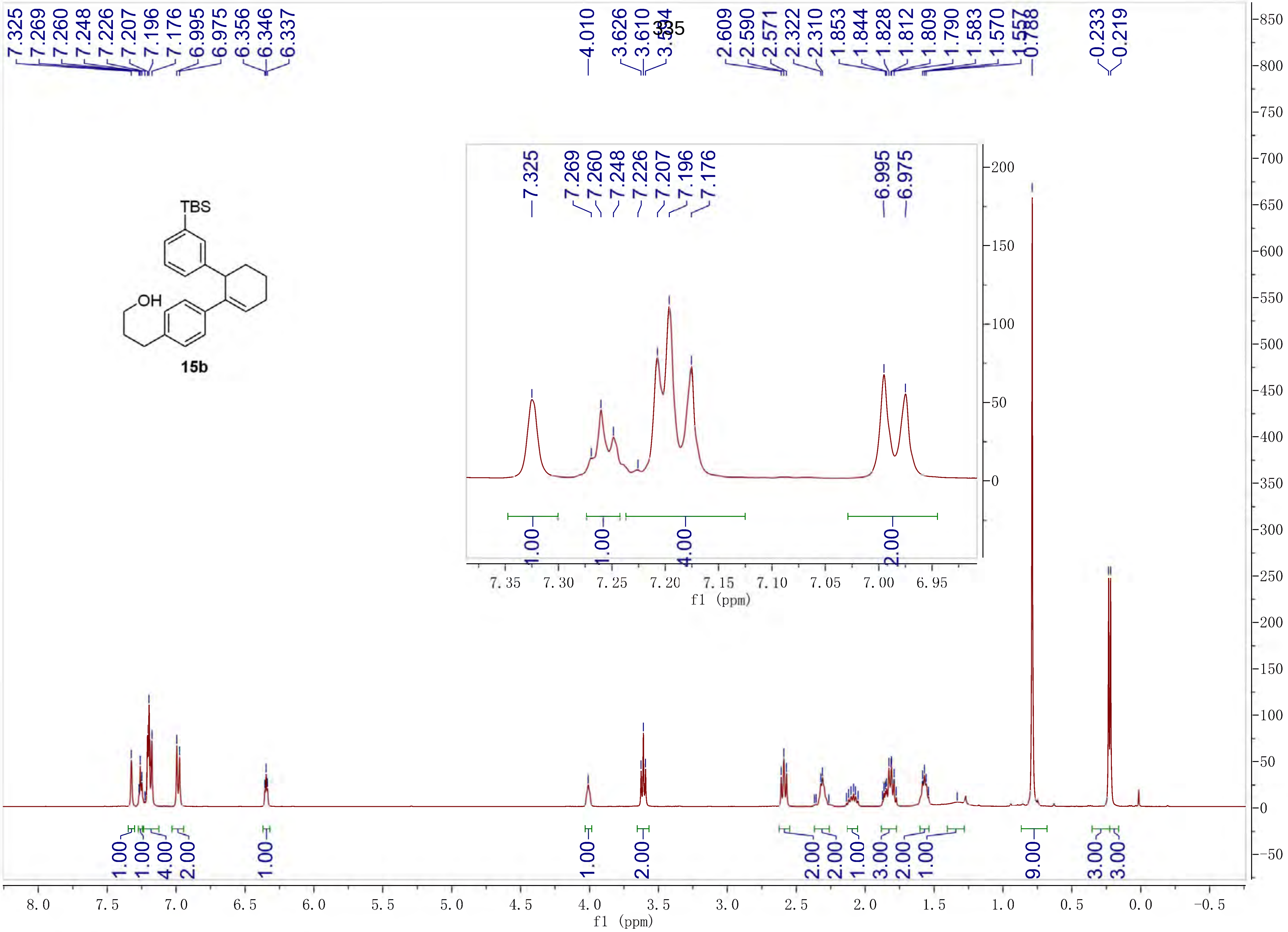

Supplementary Fig 263. ^{1}H NMR spectrum (400 MHz, $CDCl_3$, r.t.) of **15b**.

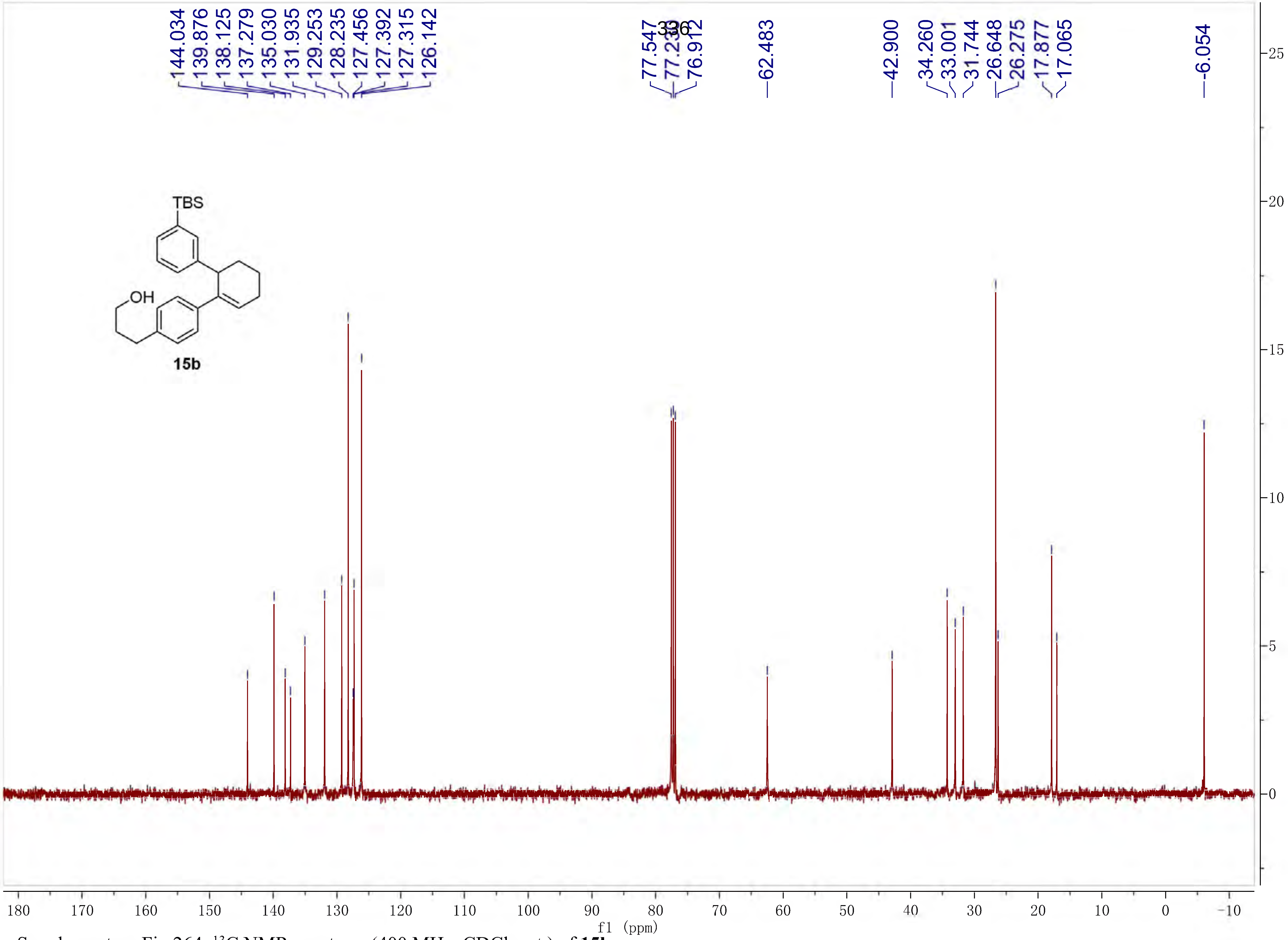

Supplementary Fig 264. ^{13}C NMR spectrum (400 MHz, $CDCl_3$, r.t.) of **15b**.

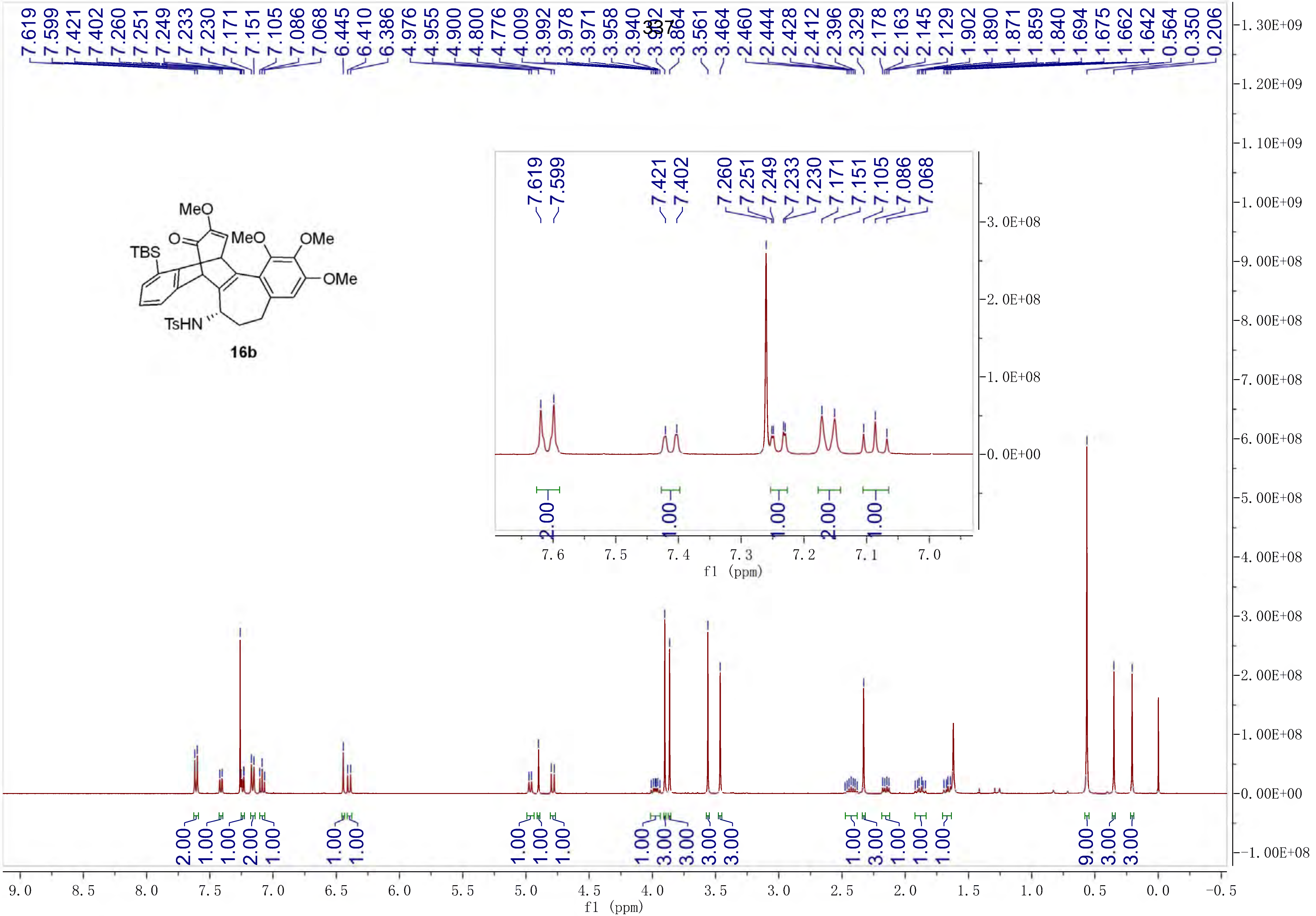

Supplementary Fig 265. ^{1}H NMR spectrum (400 MHz, $CDCl_3$, r.t.) of **16b**.

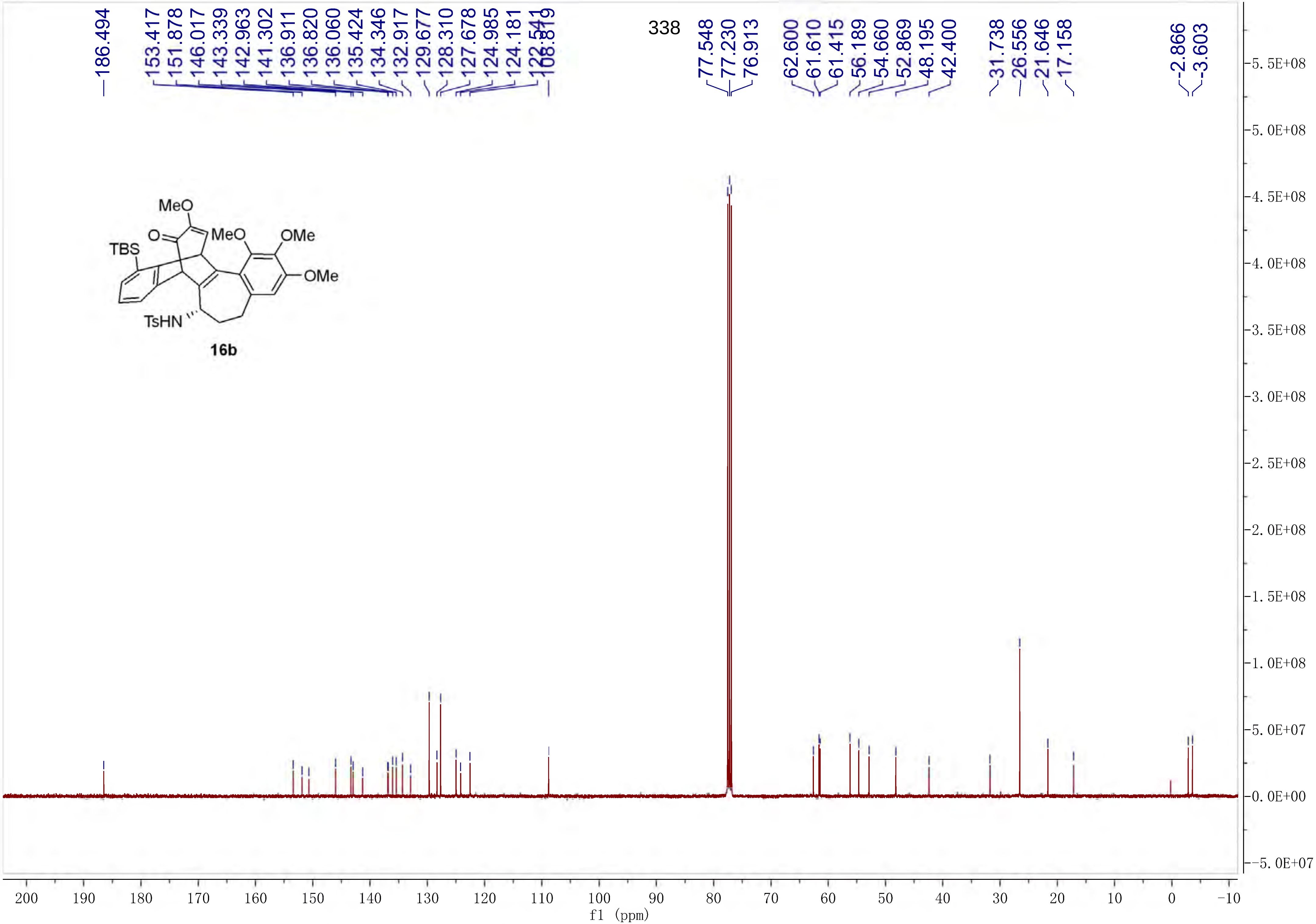

Supplementary Fig 266. ^{13}C NMR spectrum (400 MHz, $CDCl_3$, r.t.) of **16b**.

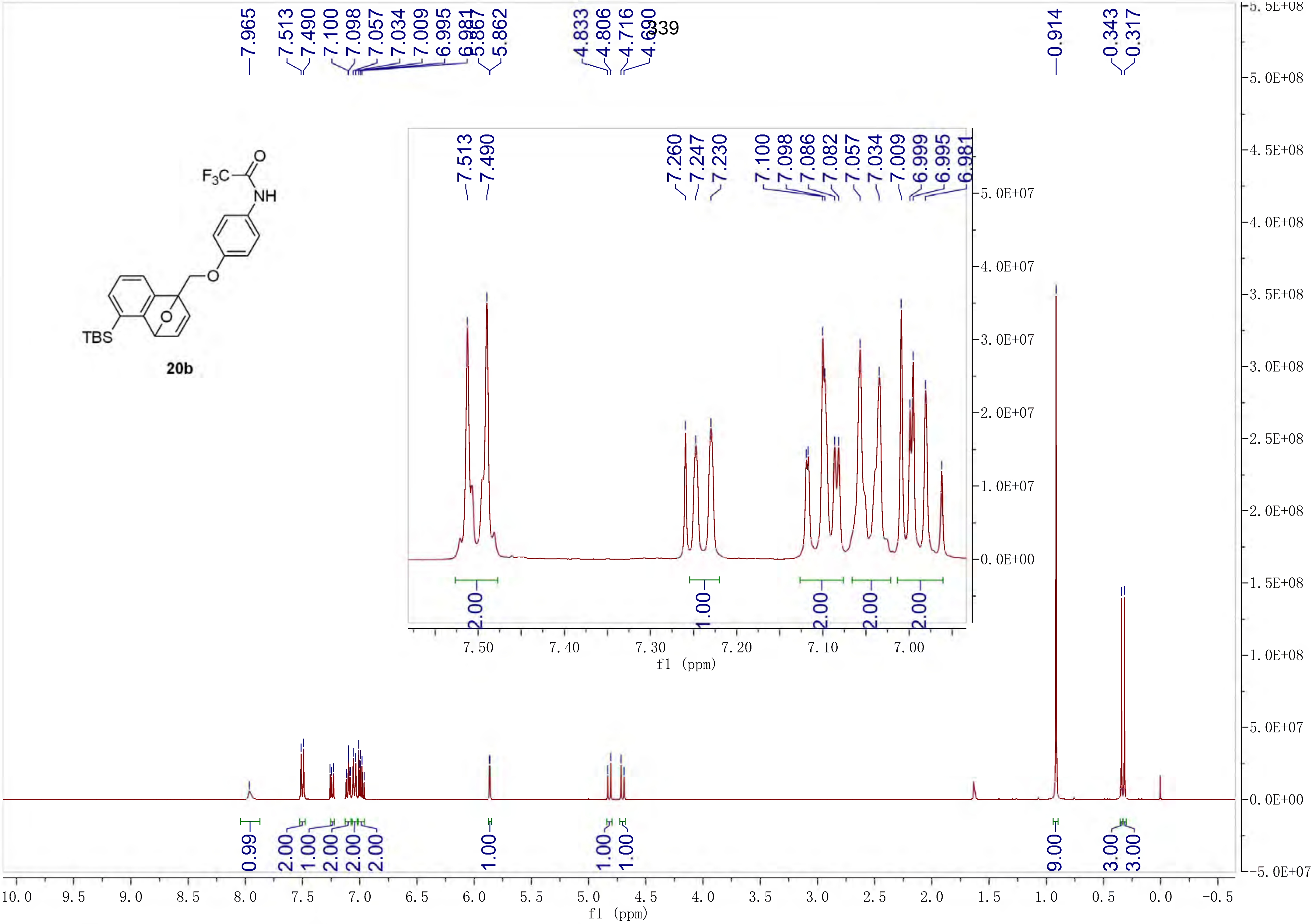

Supplementary Fig 267. ^{1}H NMR spectrum (400 MHz, $CDCl_3$, r.t.) of **20b**.

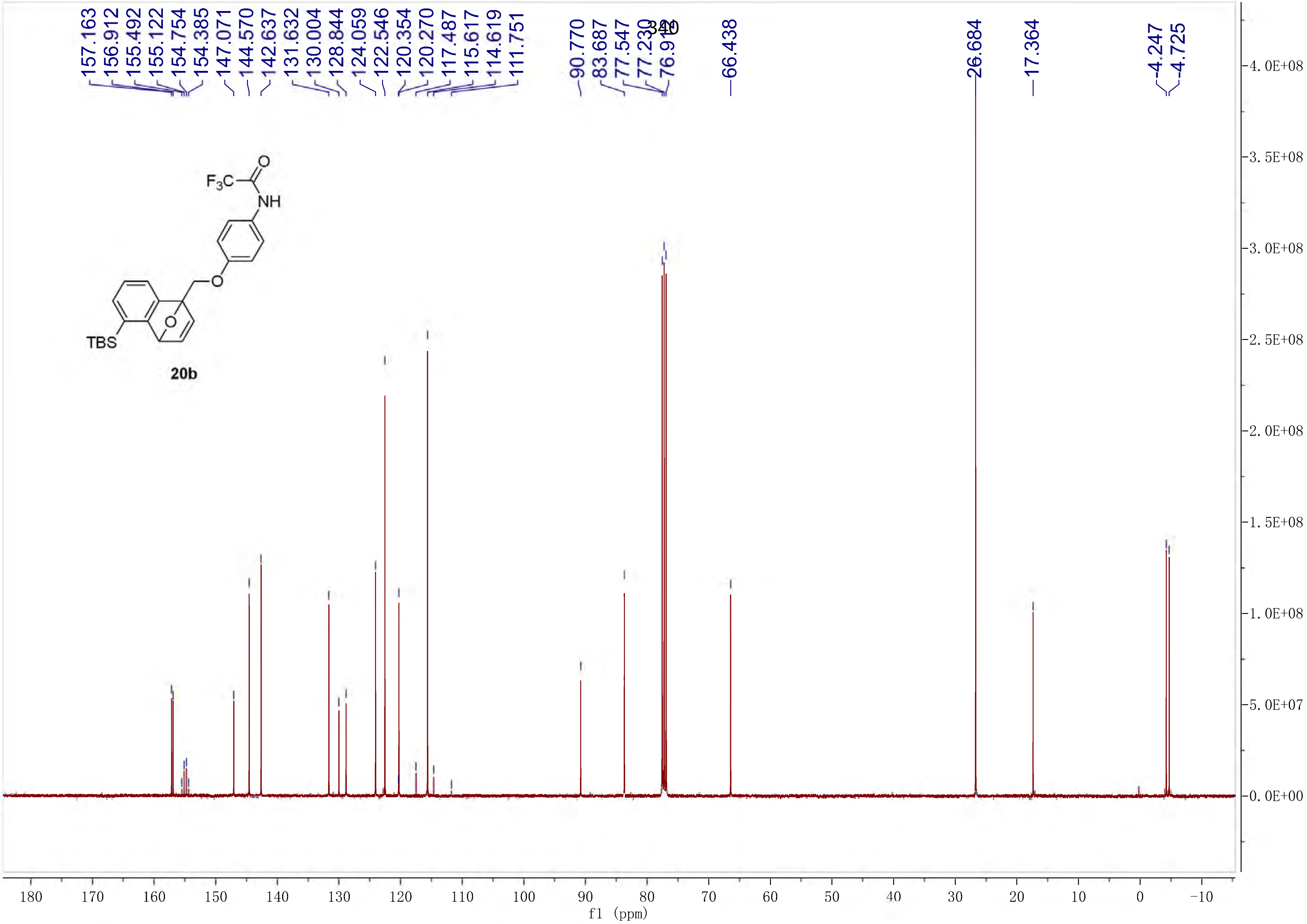

Supplementary Fig 268. ^{13}C NMR spectrum (400 MHz, $CDCl_3$, r.t.) of **20b**.

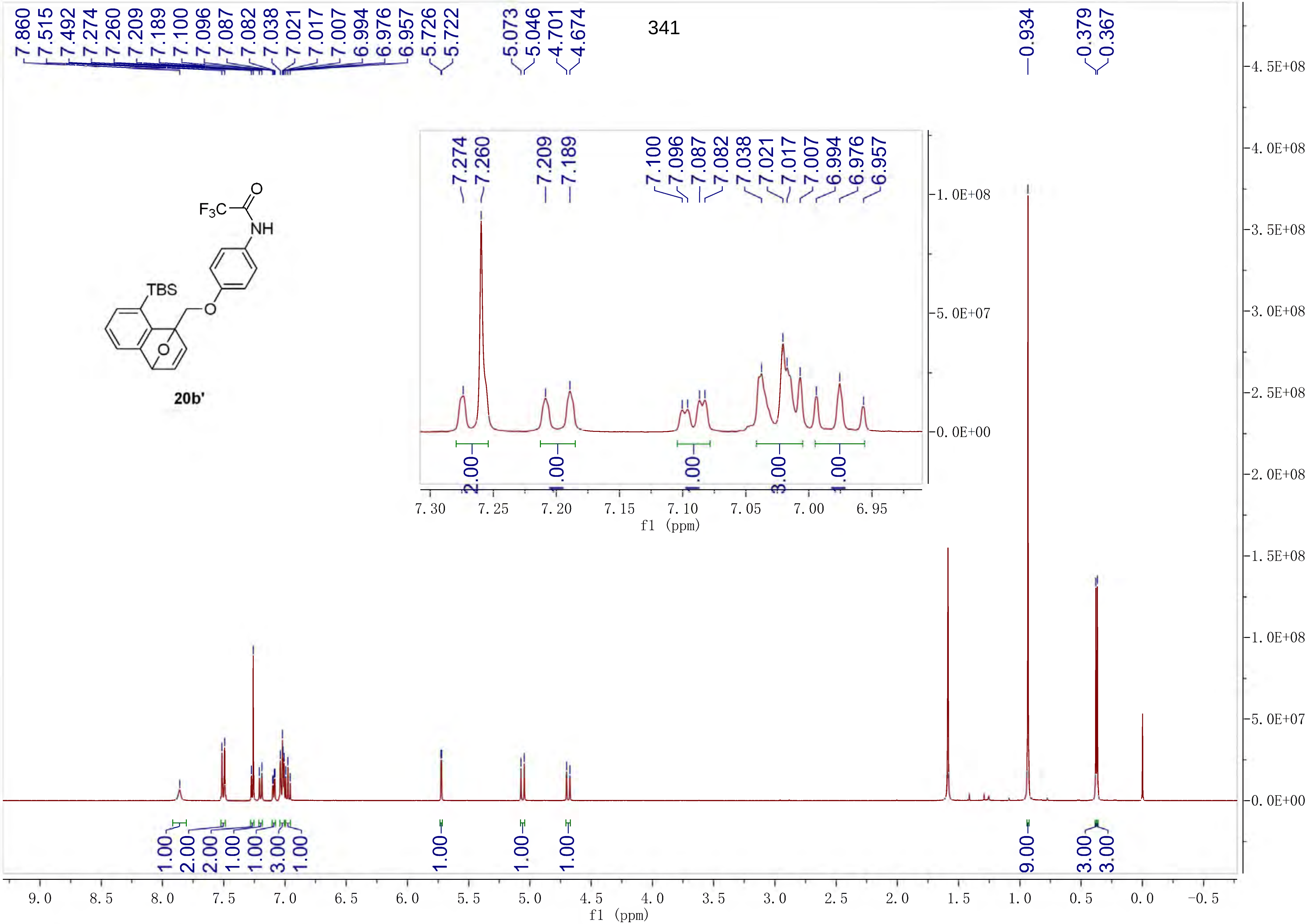

Supplementary Fig 269. ^{1}H NMR spectrum (400 MHz, $CDCl_3$, r.t.) of **20b'**.

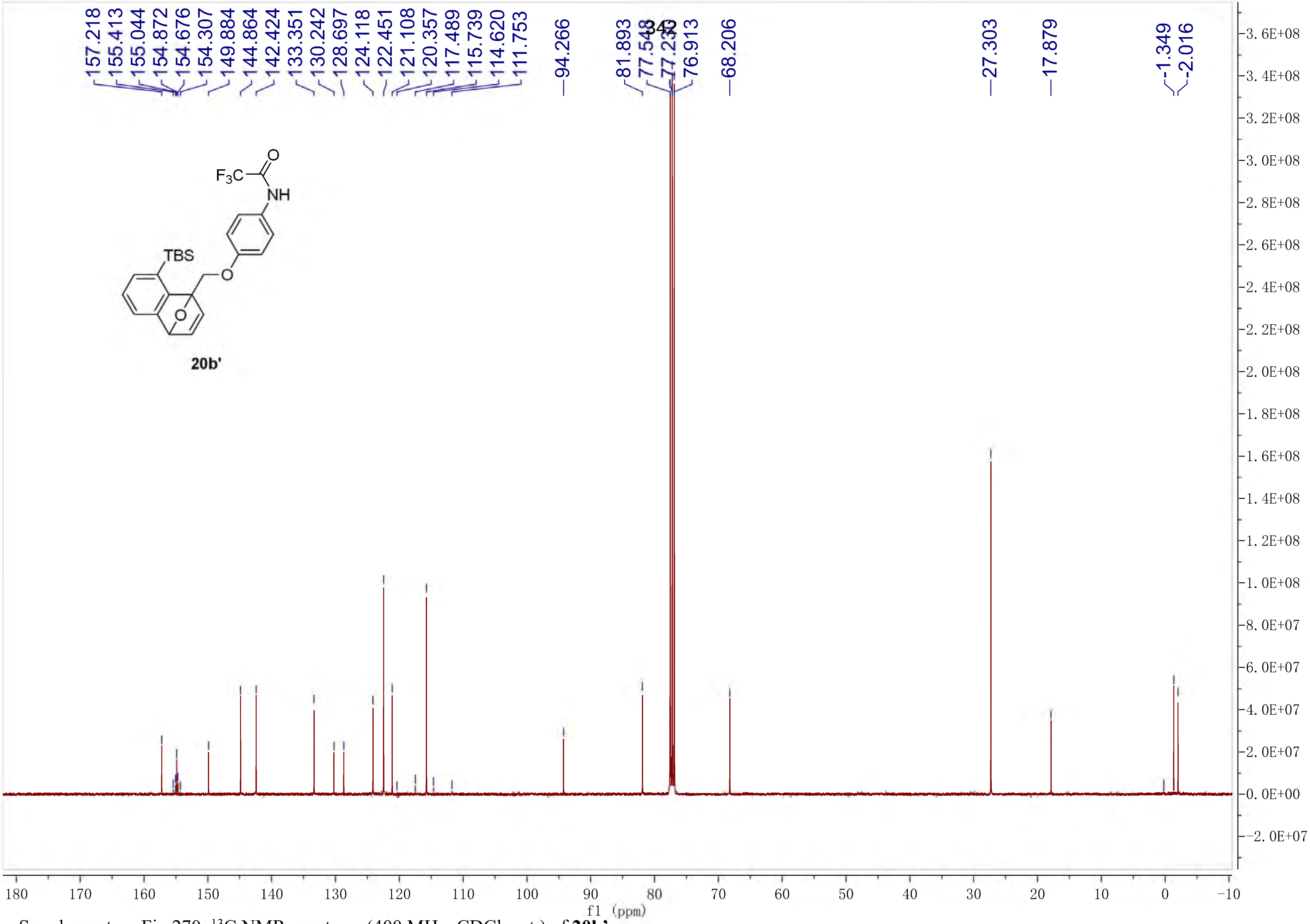

Supplementary Fig 270. ^{13}C NMR spectrum (400 MHz, $CDCl_3$, r.t.) of **20b'**.

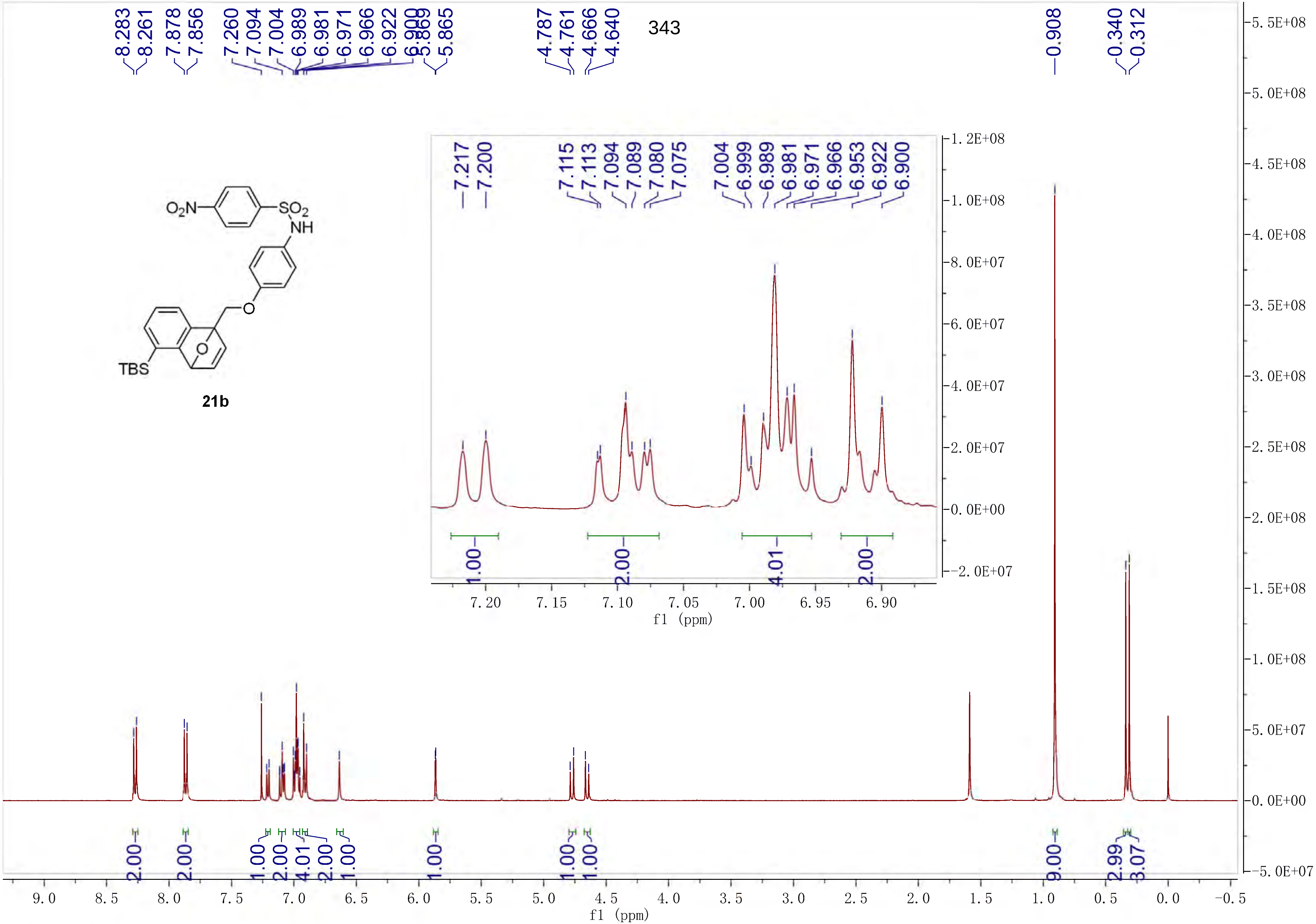

Supplementary Fig 271. ^{1}H NMR spectrum (400 MHz, $CDCl_3$, r.t.) of **21b**.

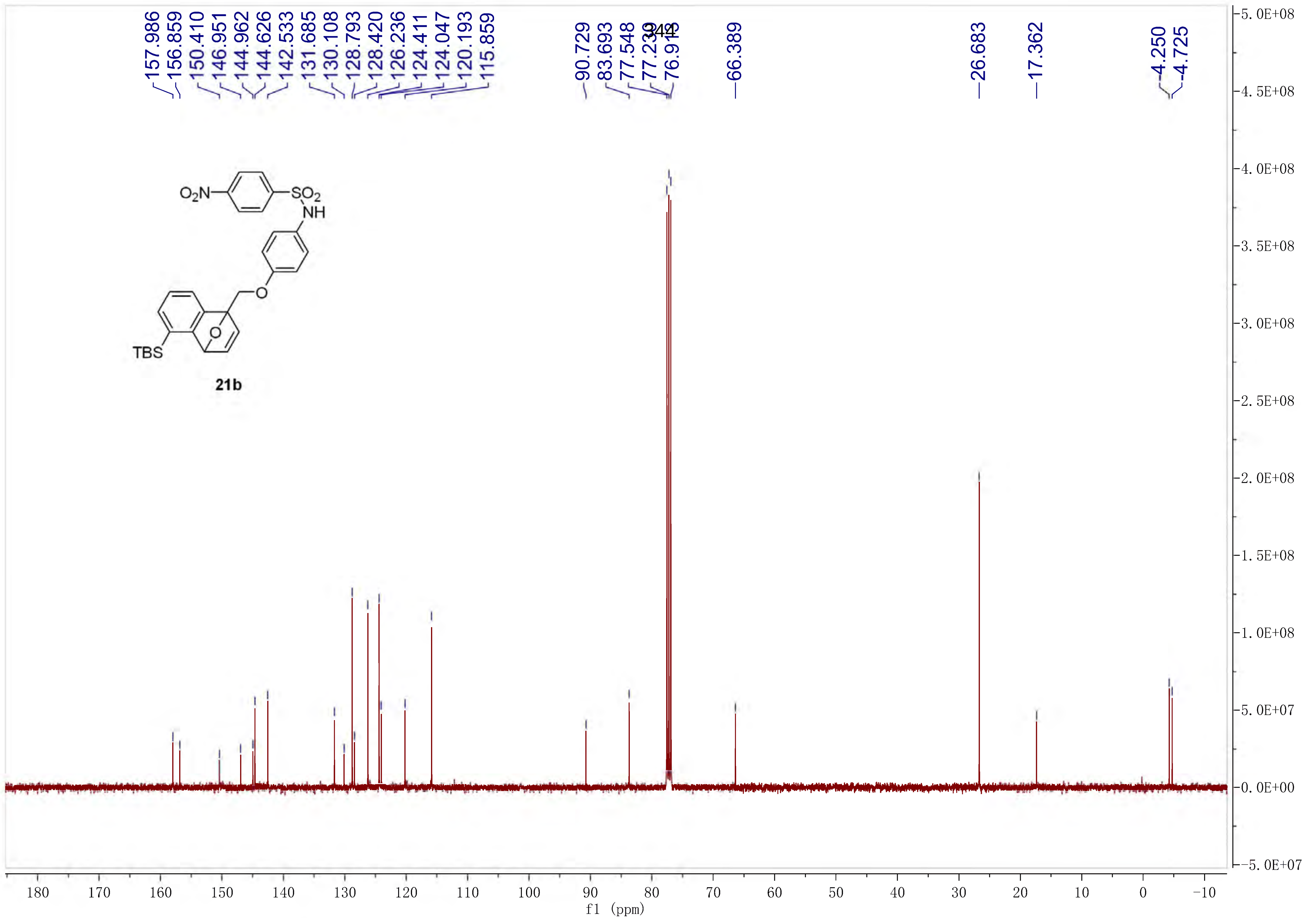

Supplementary Fig 272. ^{13}C NMR spectrum (400 MHz, $CDCl_3$, r.t.) of **21b**.

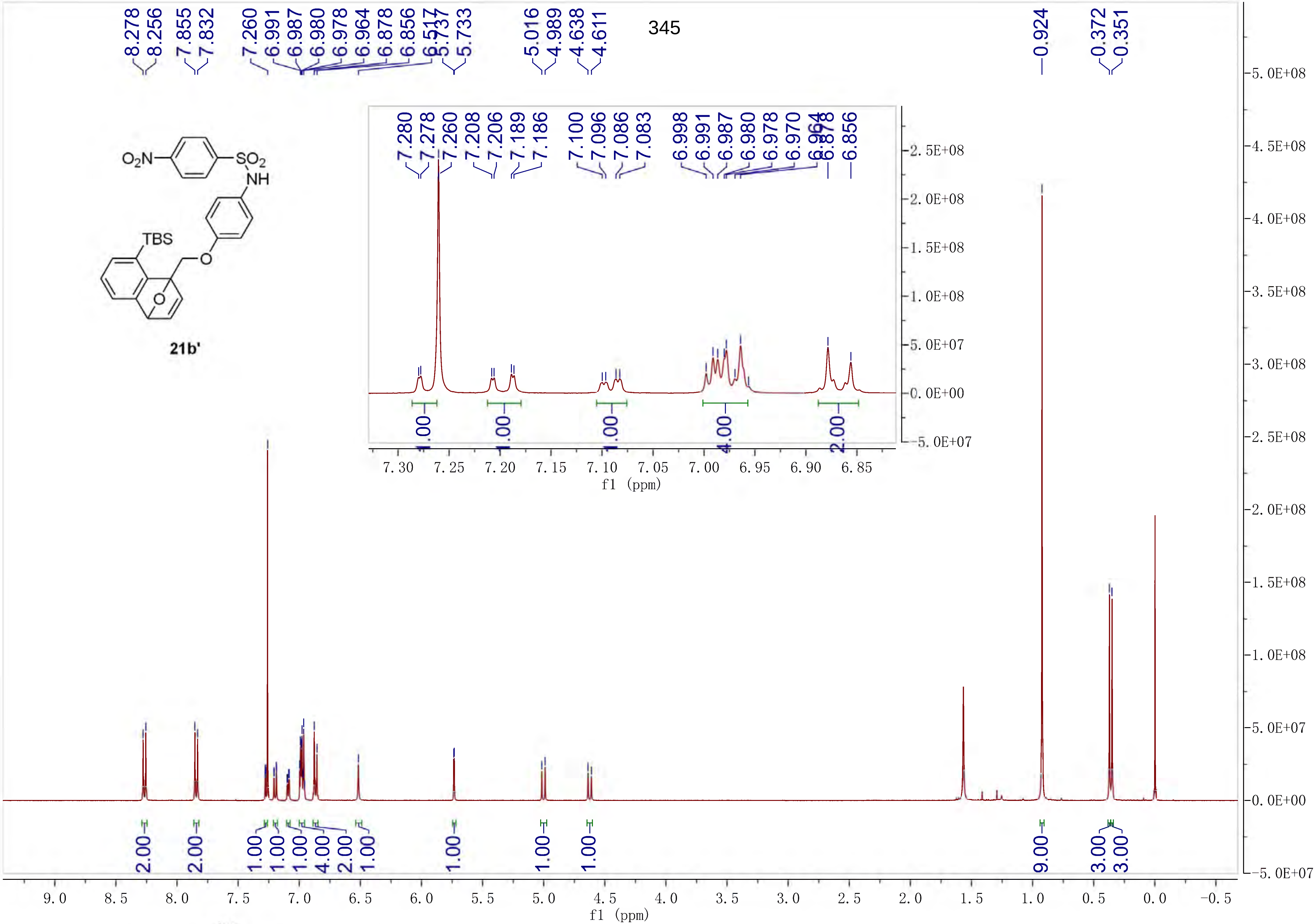

Supplementary Fig 273. ^{1}H NMR spectrum (400 MHz, $CDCl_3$, r.t.) of **21b'**.

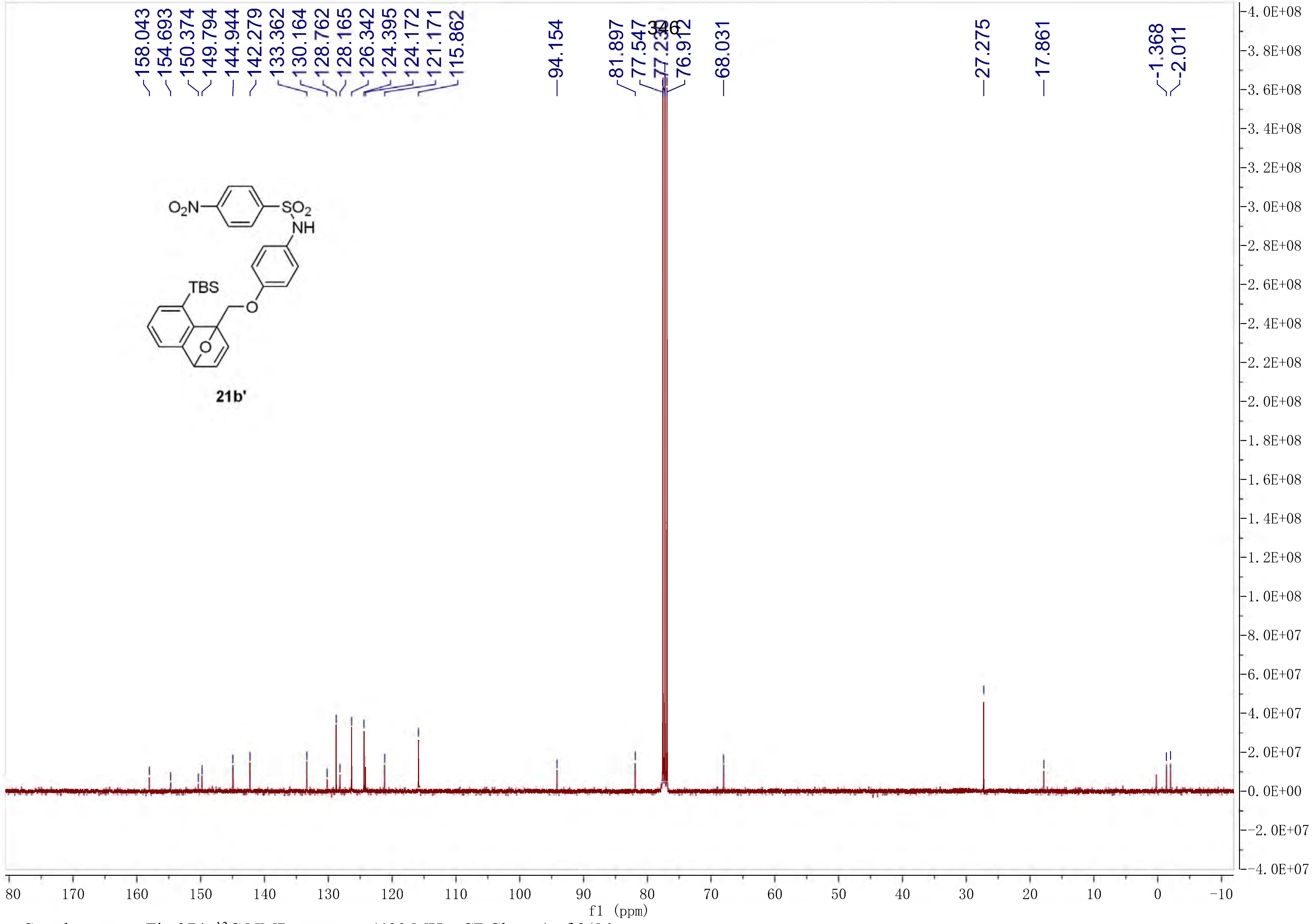

Supplementary Fig 274. ^{13}C NMR spectrum (400 MHz, $CDCl_3$, r.t.) of **21b’**.

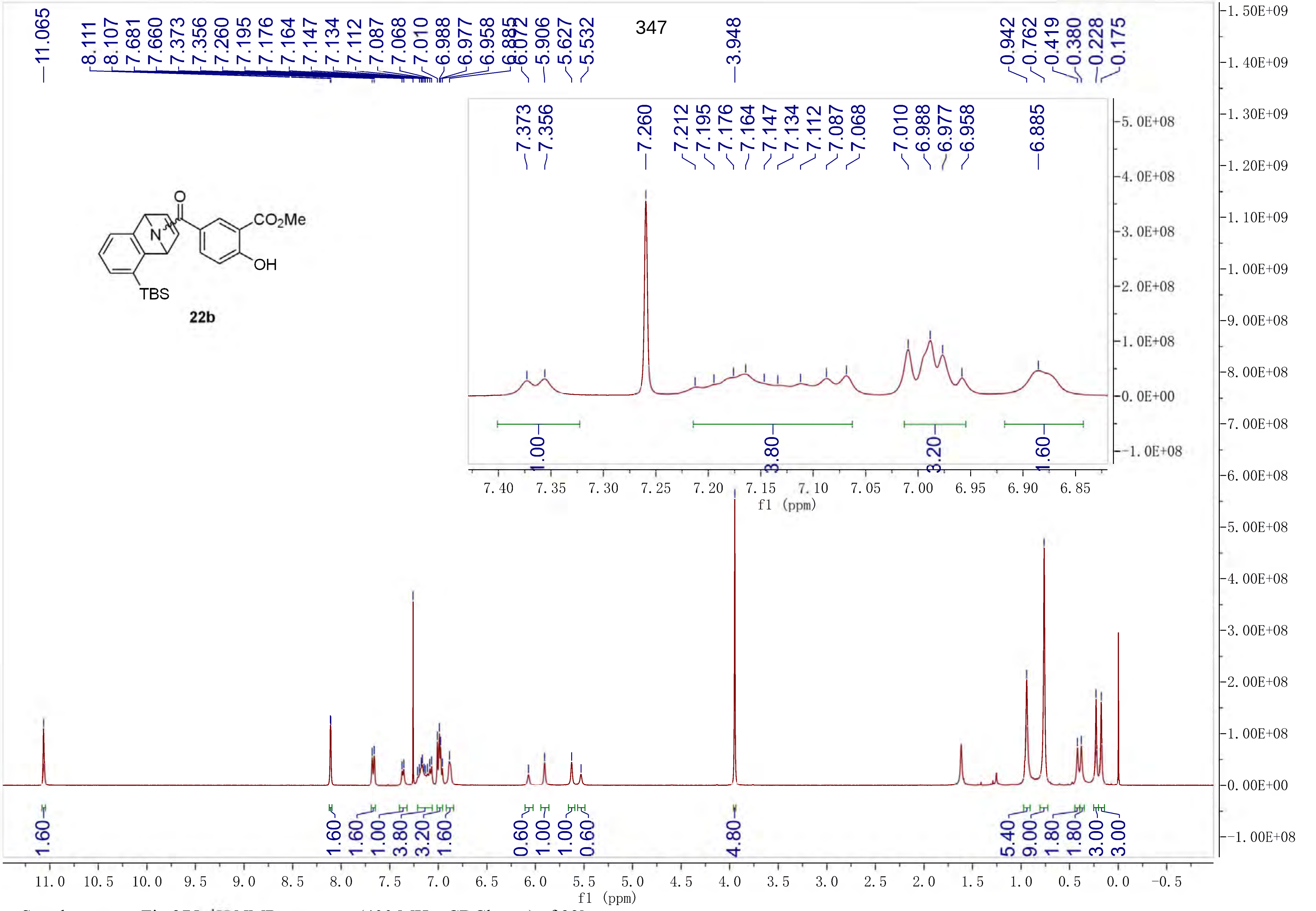

Supplementary Fig 275. ^{1}H NMR spectrum (400 MHz, $CDCl_3$, r.t.) of **22b**.

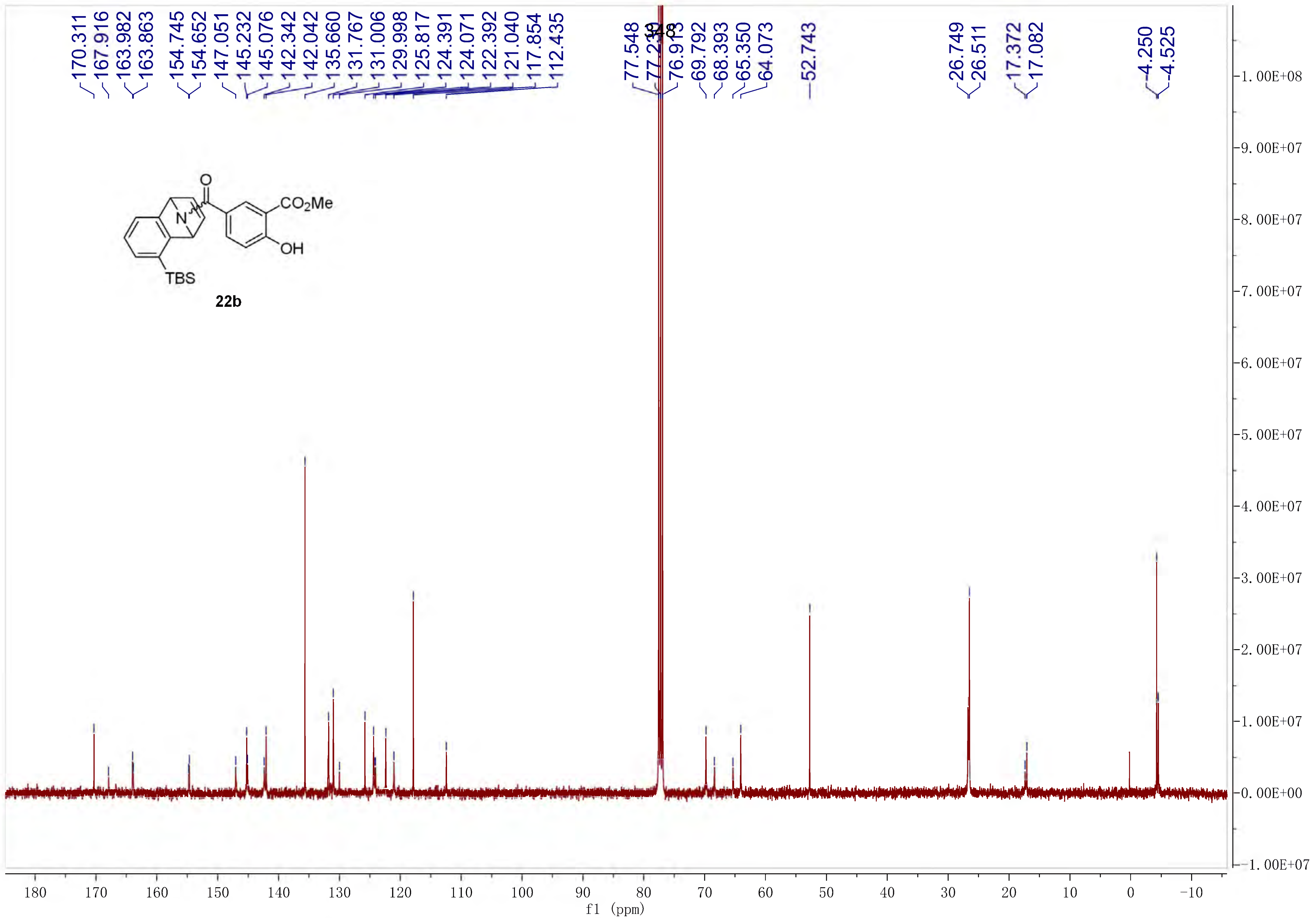

Supplementary Fig 276. ^{13}C NMR spectrum (400 MHz, $CDCl_3$, r.t.) of **22b**.

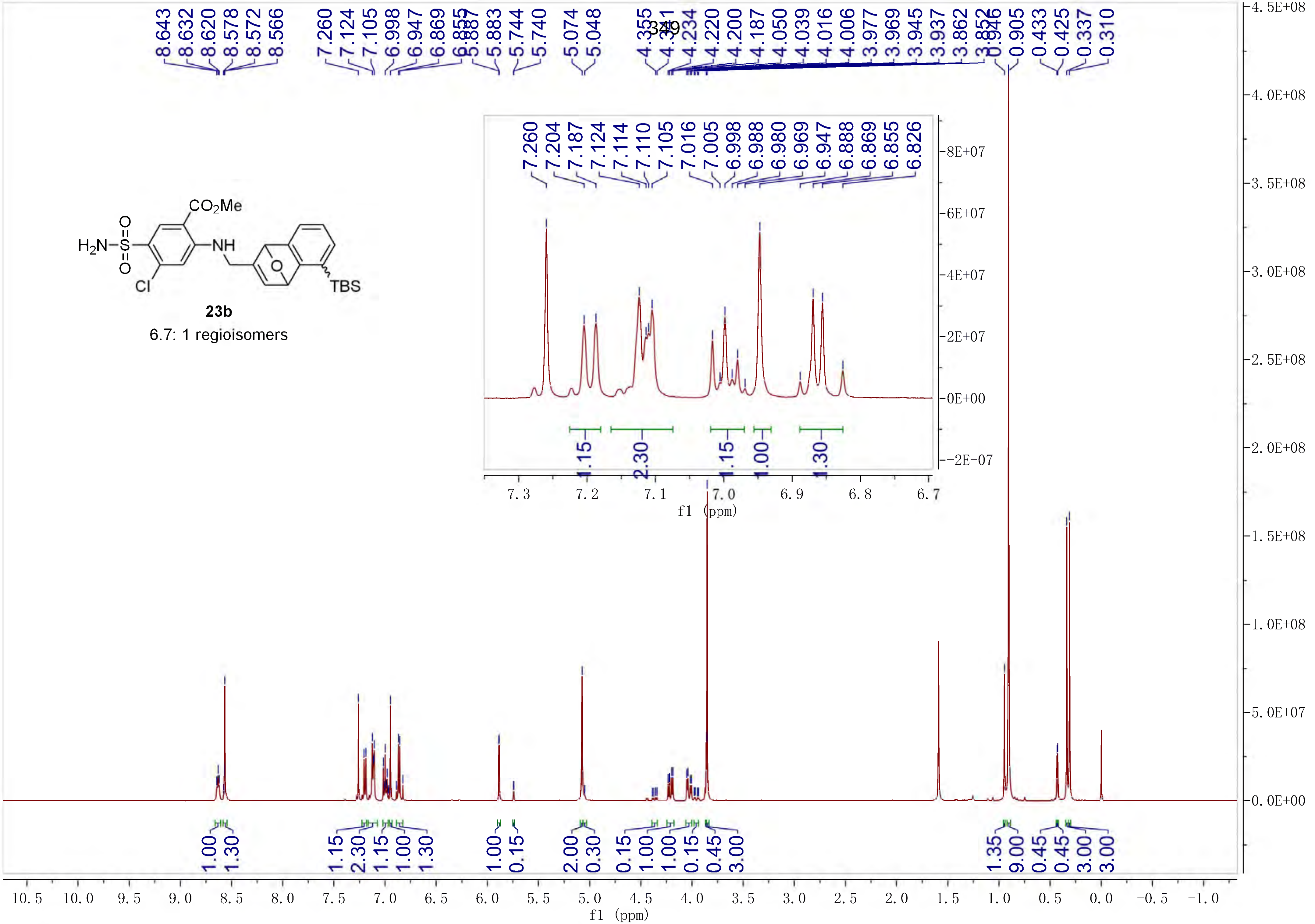

Supplementary Fig 277. ^{1}H NMR spectrum (400 MHz, $CDCl_3$, r.t.) of **23b**.

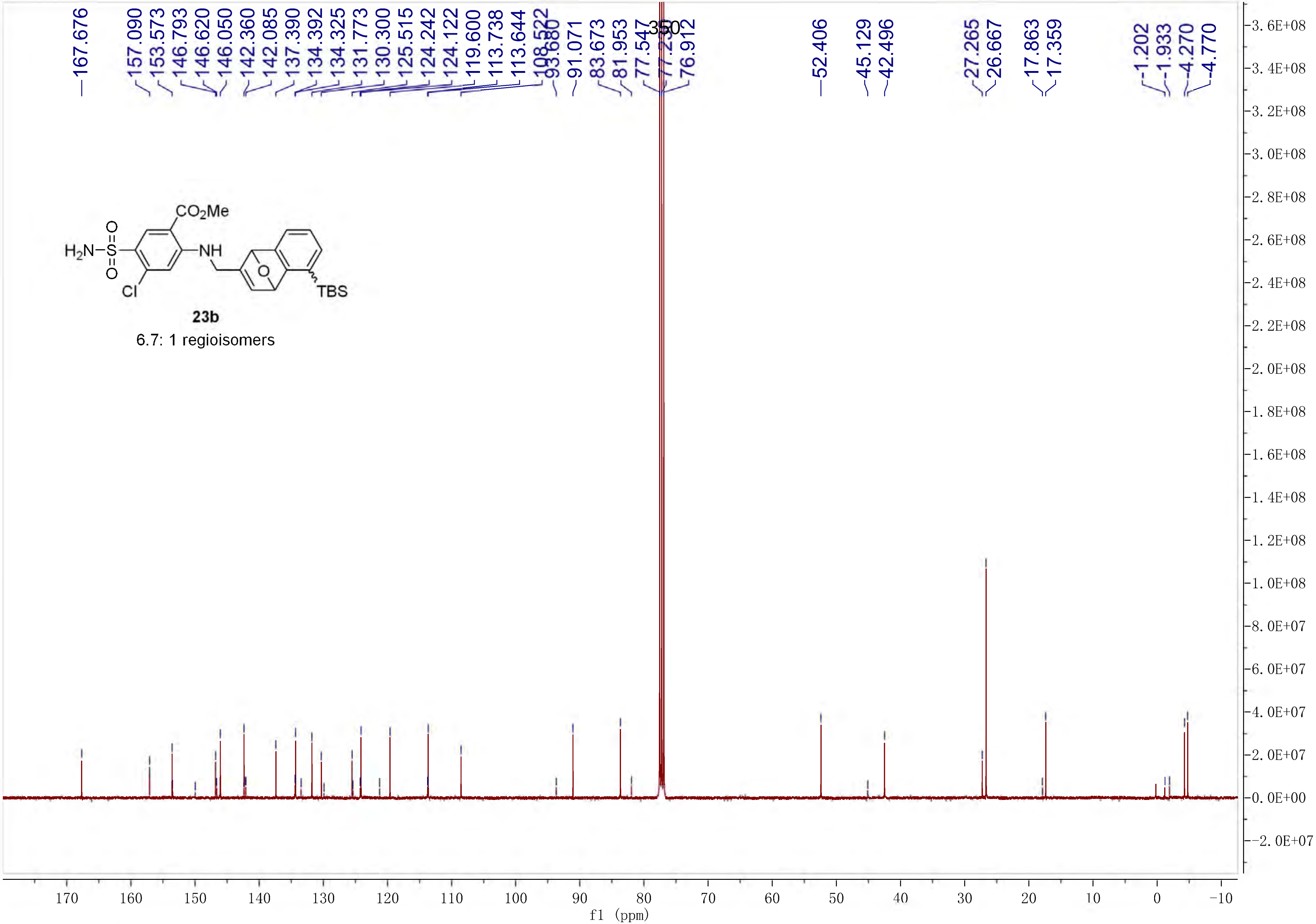

Supplementary Fig 278. ^{13}C NMR spectrum (400 MHz, $CDCl_3$, r.t.) of **23b**.

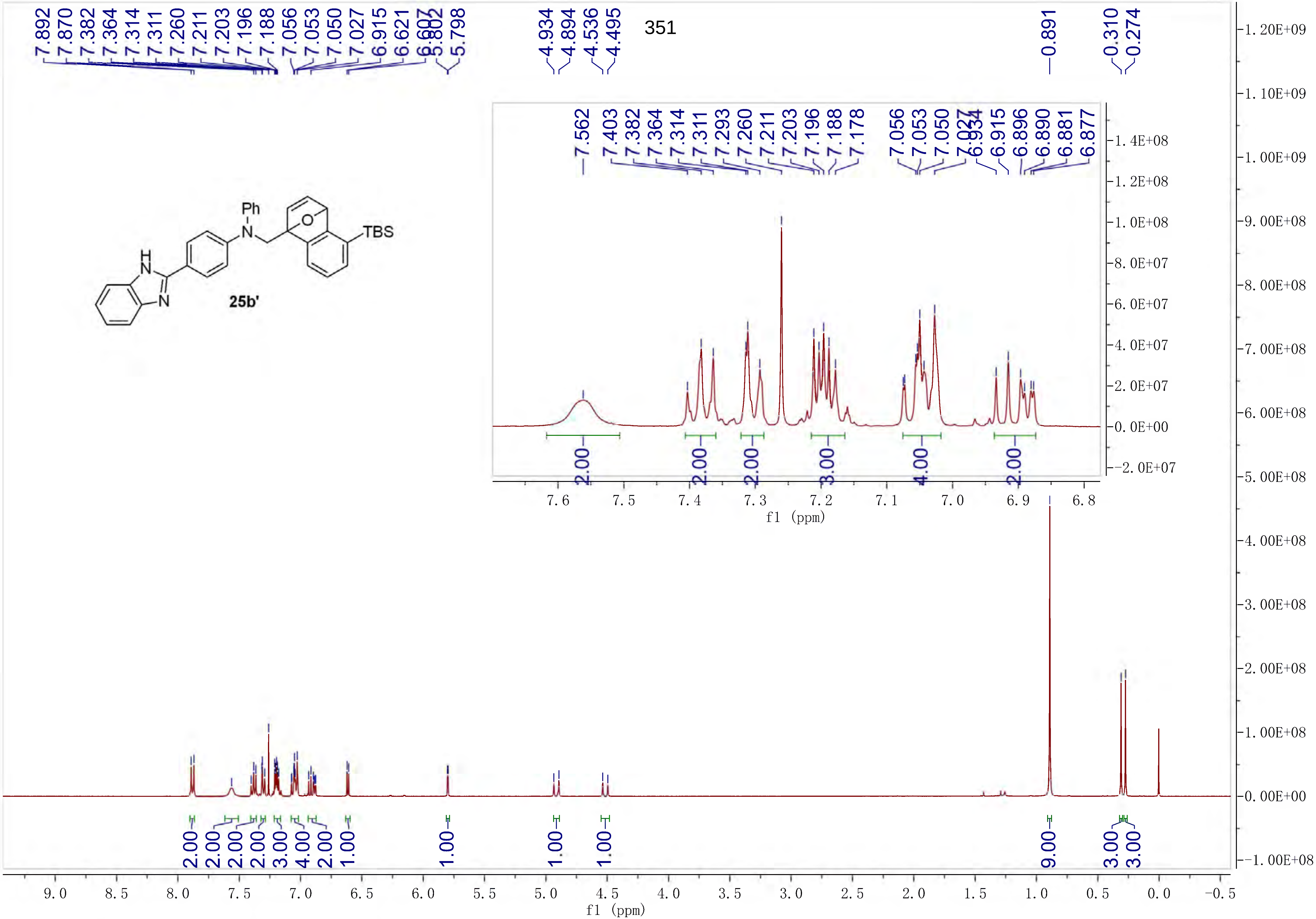

Supplementary Fig 279. ^{1}H NMR spectrum (400 MHz, $CDCl_3$, r.t.) of **25b'**.

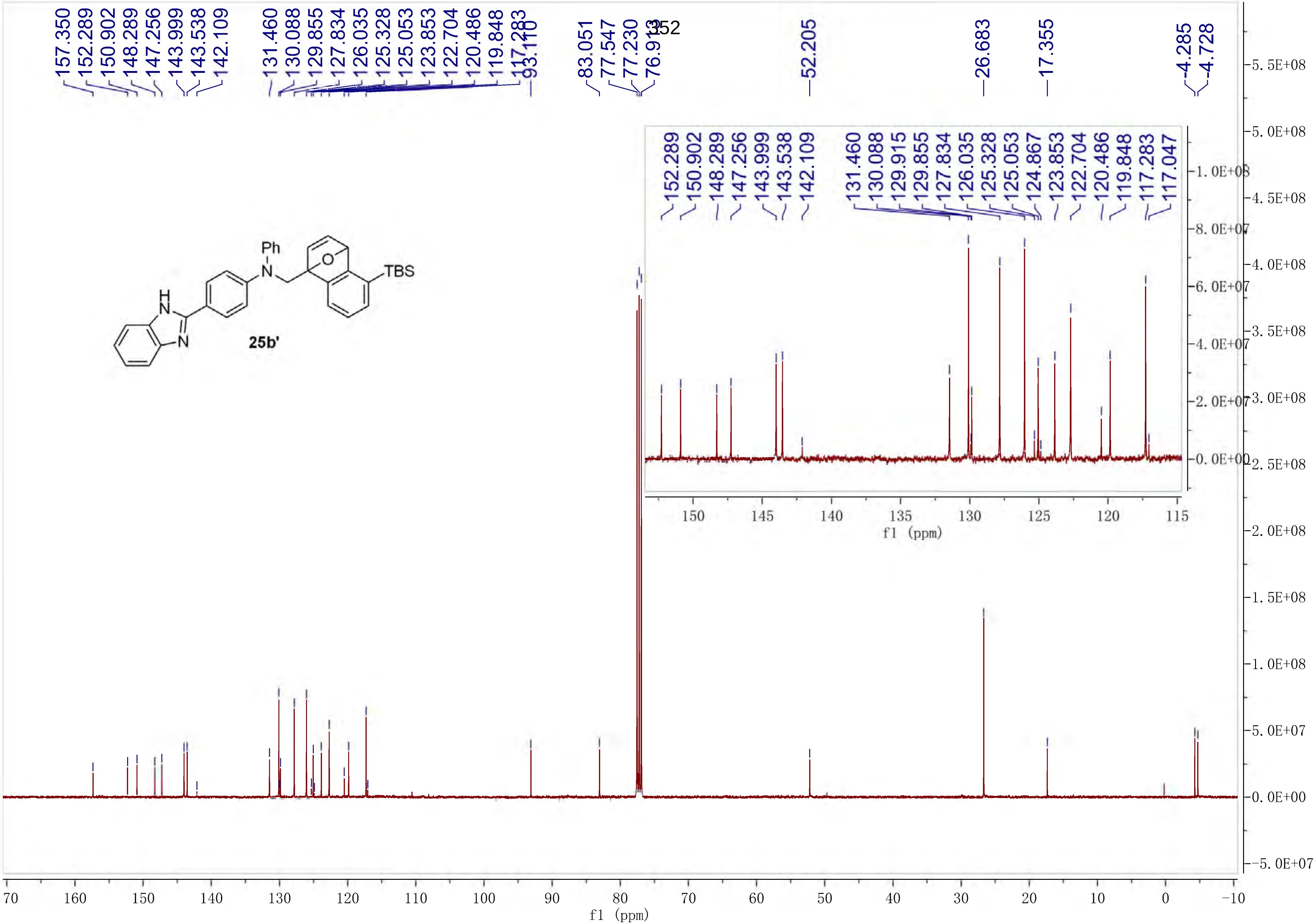

Supplementary Fig 280. ^{13}C NMR spectrum (400 MHz, $CDCl_3$, r.t.) of **25b’**.

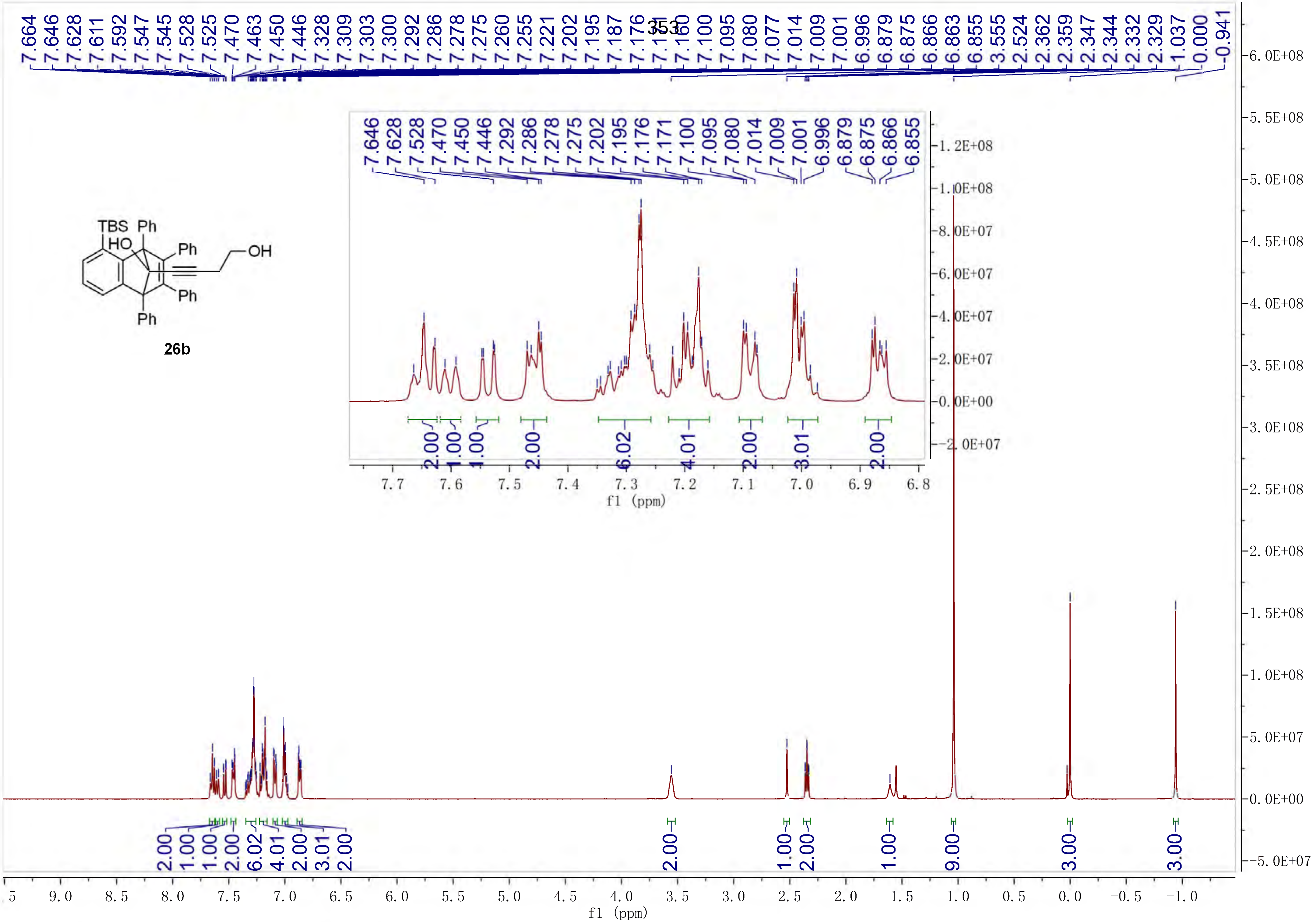

Supplementary Fig 281. ^{1}H NMR spectrum (400 MHz, $CDCl_3$, r.t.) of **26b**.

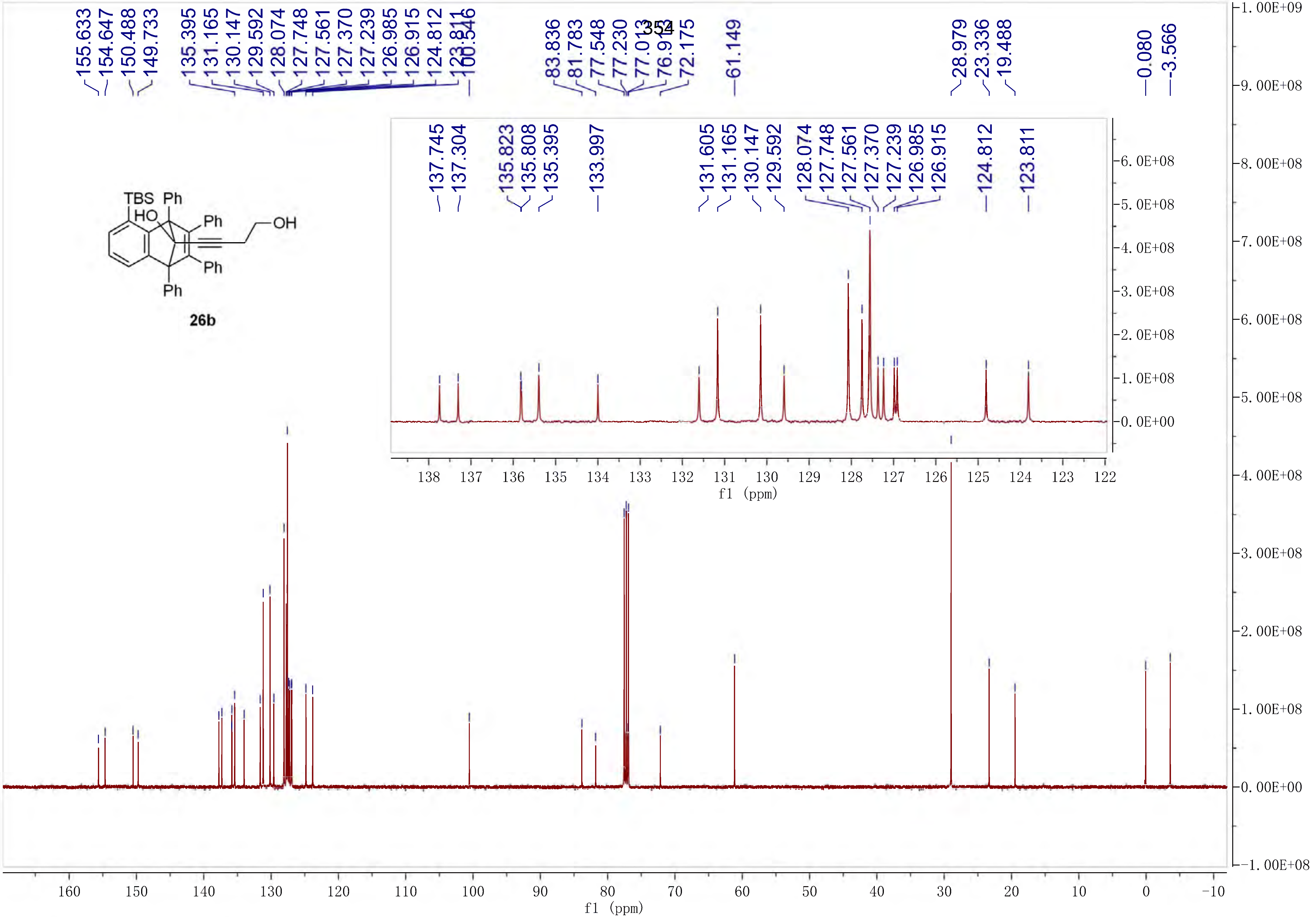

Supplementary Fig 282. ^{13}C NMR spectrum (400 MHz, $CDCl_3$, r.t.) of **26b**.

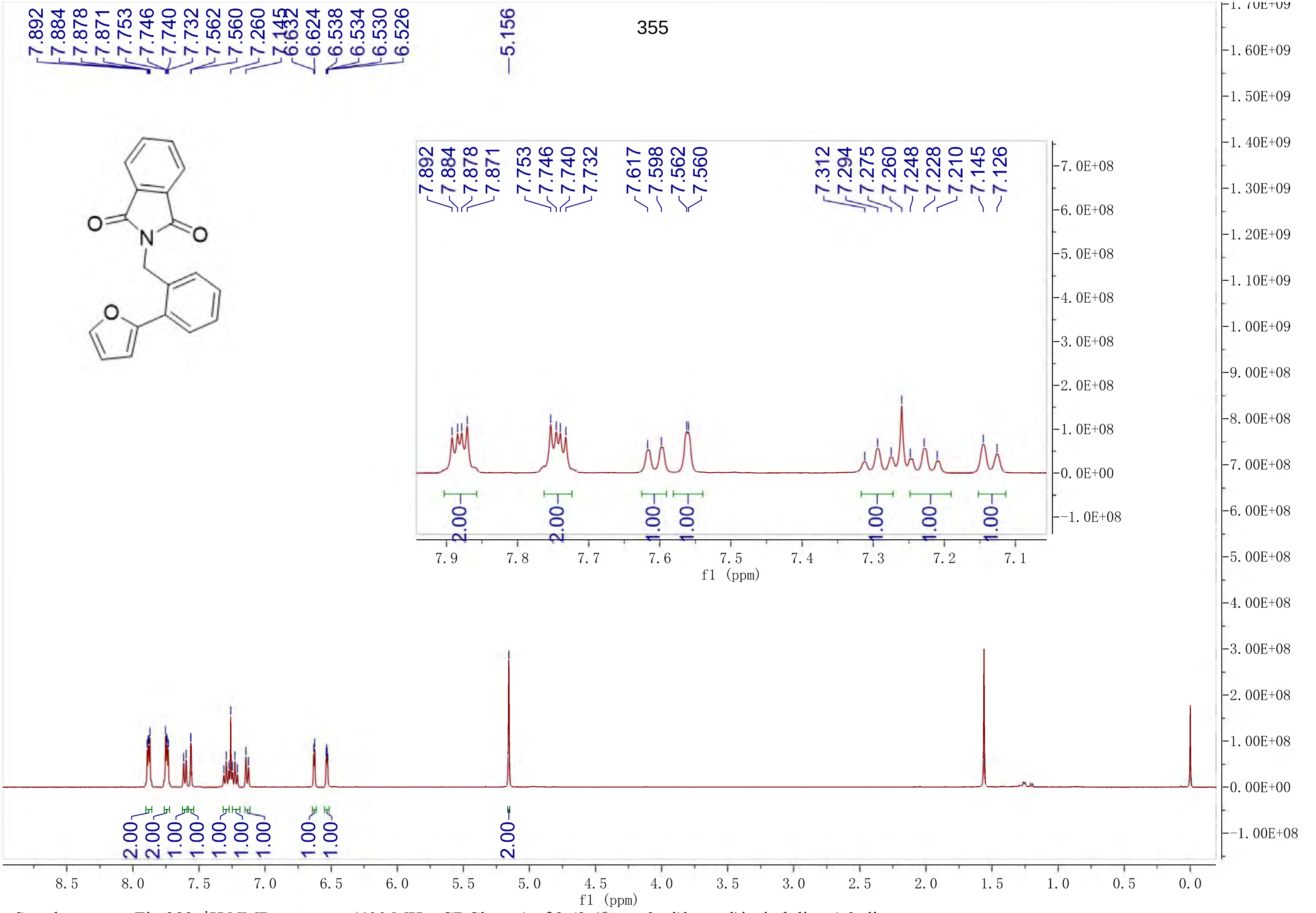

Supplementary Fig 283. ^{1}H NMR spectrum (400 MHz, $CDCl_3$, r.t.) of 2-(2-(furan-2-yl)benzyl)isoindoline-1,3-dione.

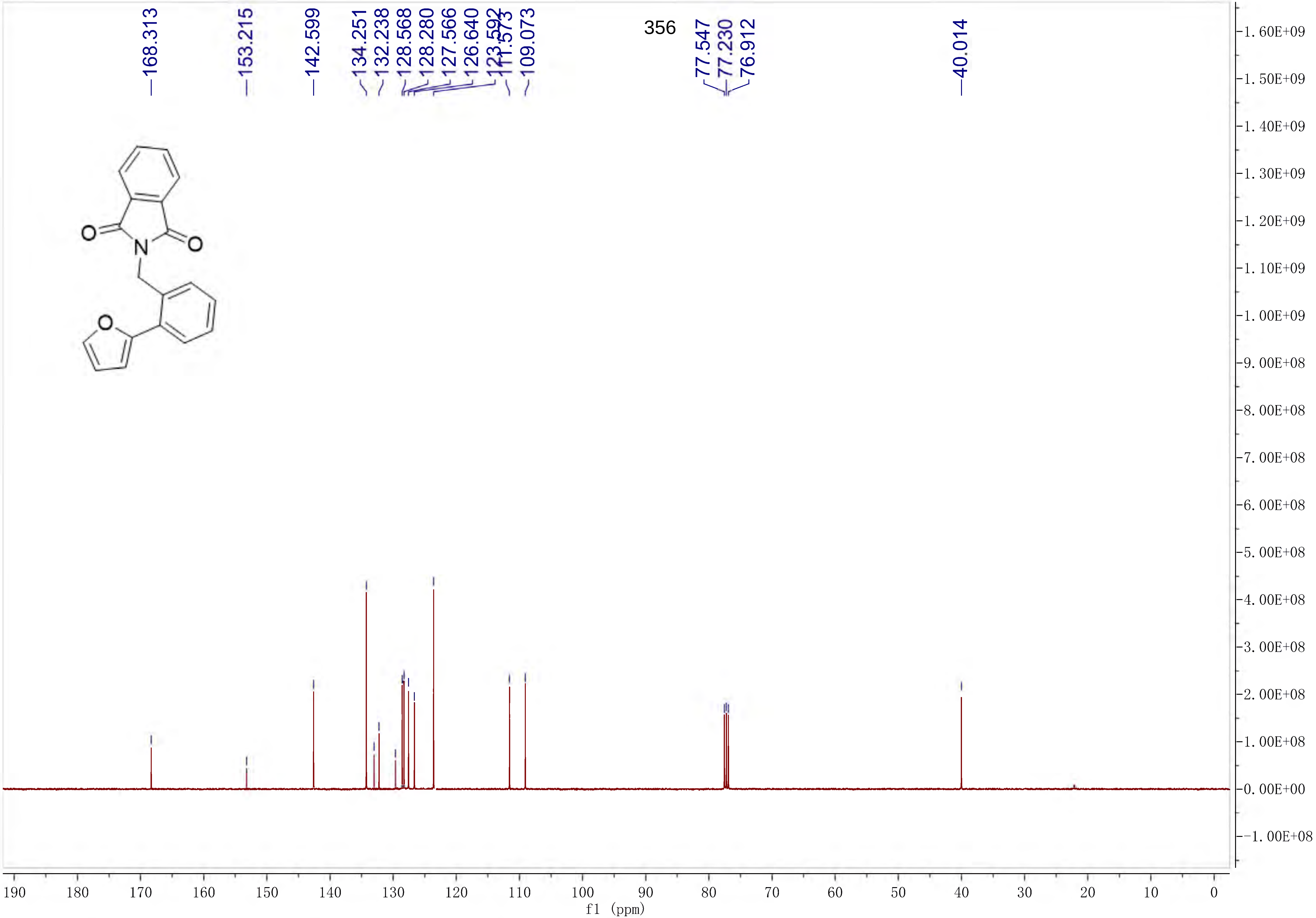

Supplementary Fig 284. ^{13}C NMR spectrum (400 MHz, $CDCl_3$, r.t.) of 2-(2-(furan-2-yl)benzyl)isoindoline-1,3-dione.

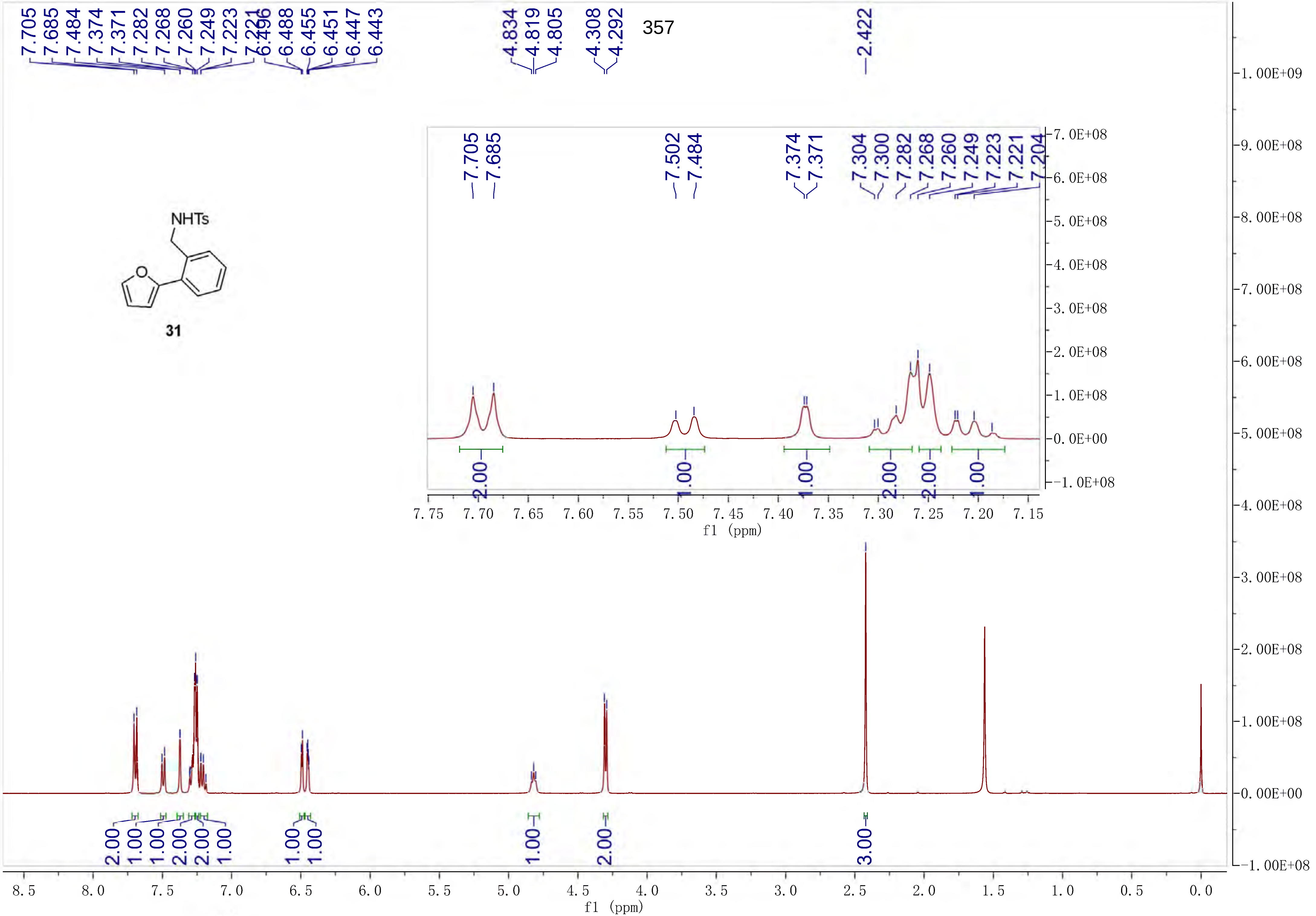

Supplementary Fig 285. ^{1}H NMR spectrum (400 MHz, $CDCl_3$, r.t.) of **31**.

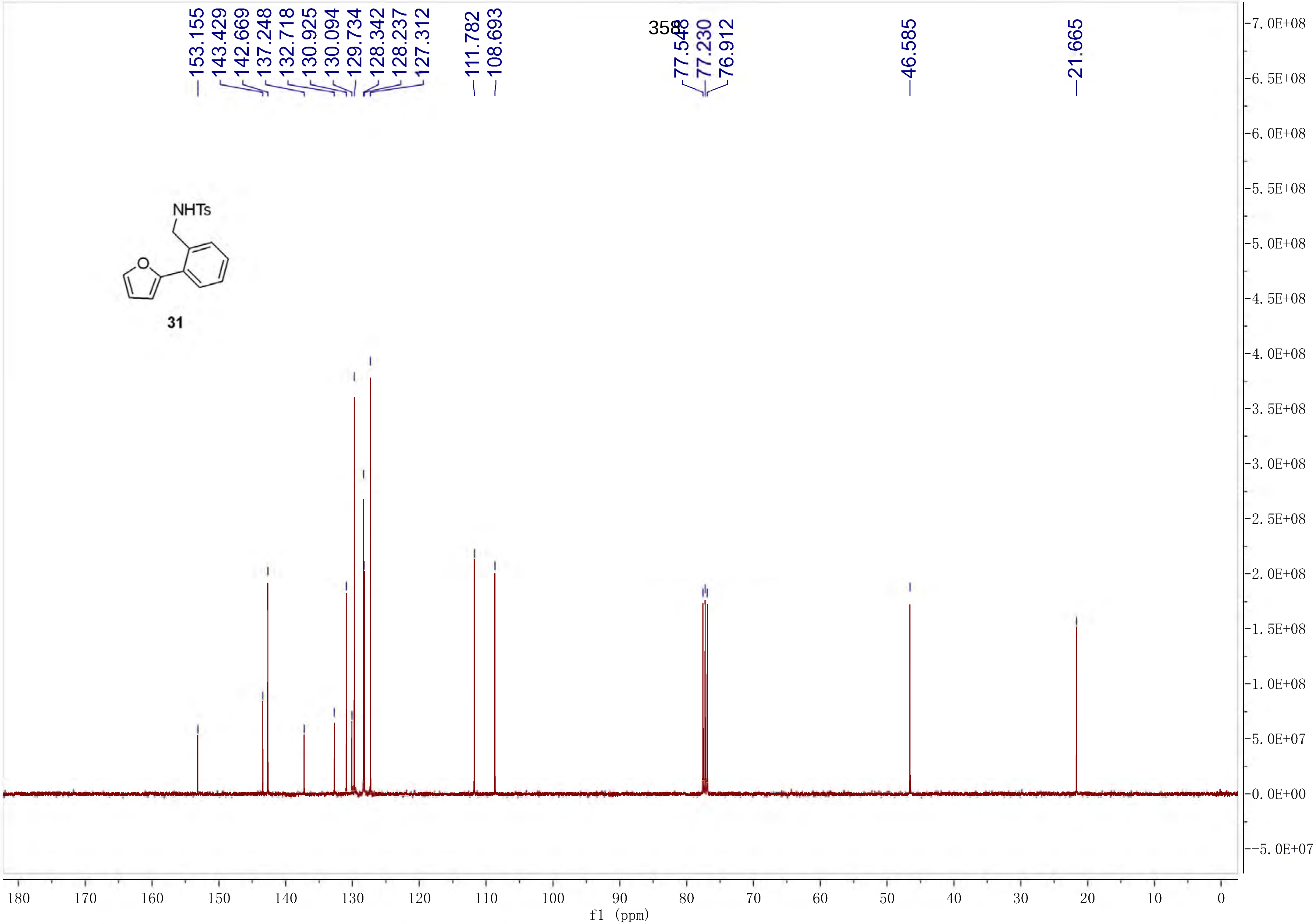

Supplementary Fig 286. ^{13}C NMR spectrum (400 MHz, $CDCl_3$, r.t.) of **31**.

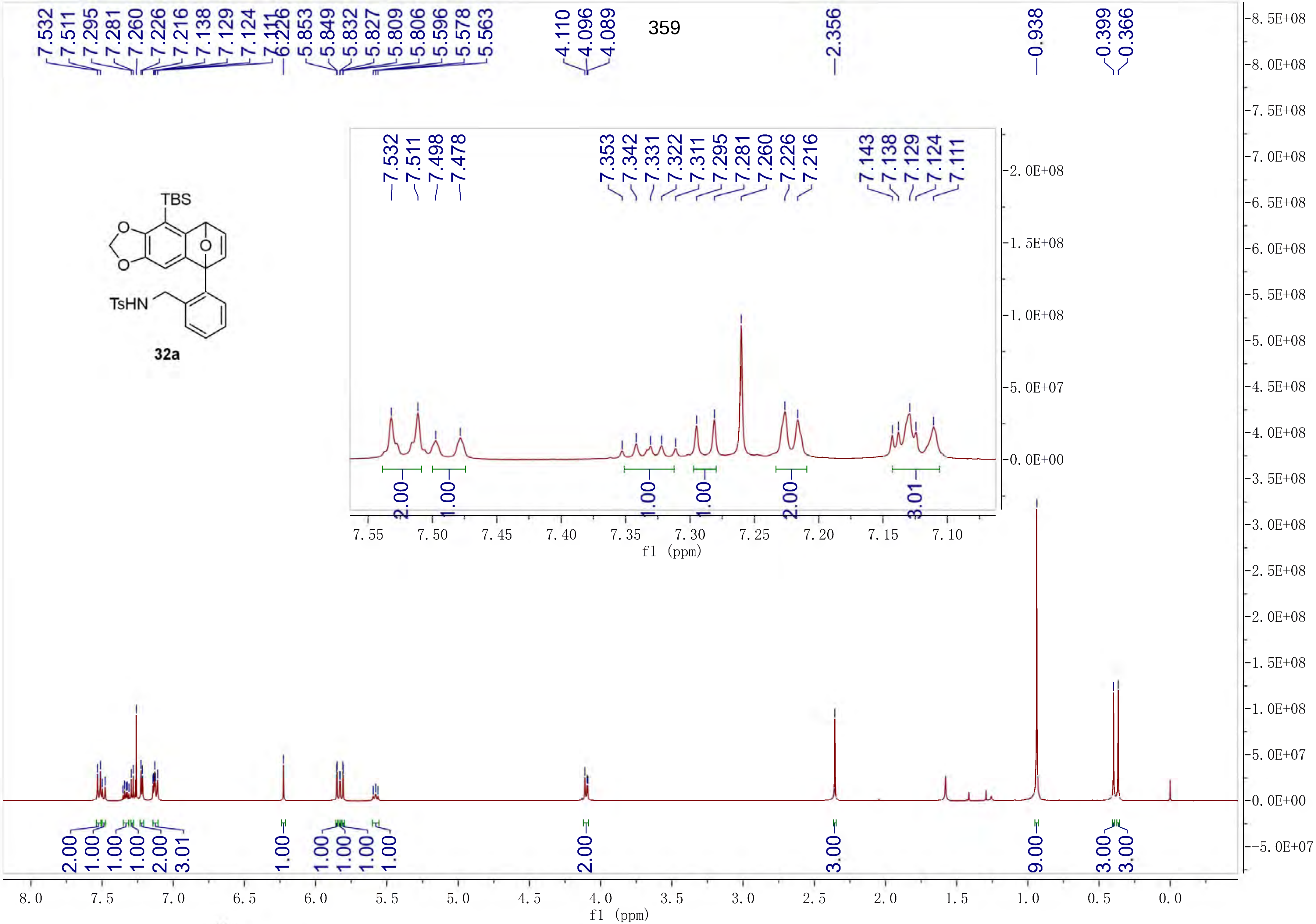

Supplementary Fig 287. ^{1}H NMR spectrum (400 MHz, $CDCl_3$, r.t.) of **32a**.

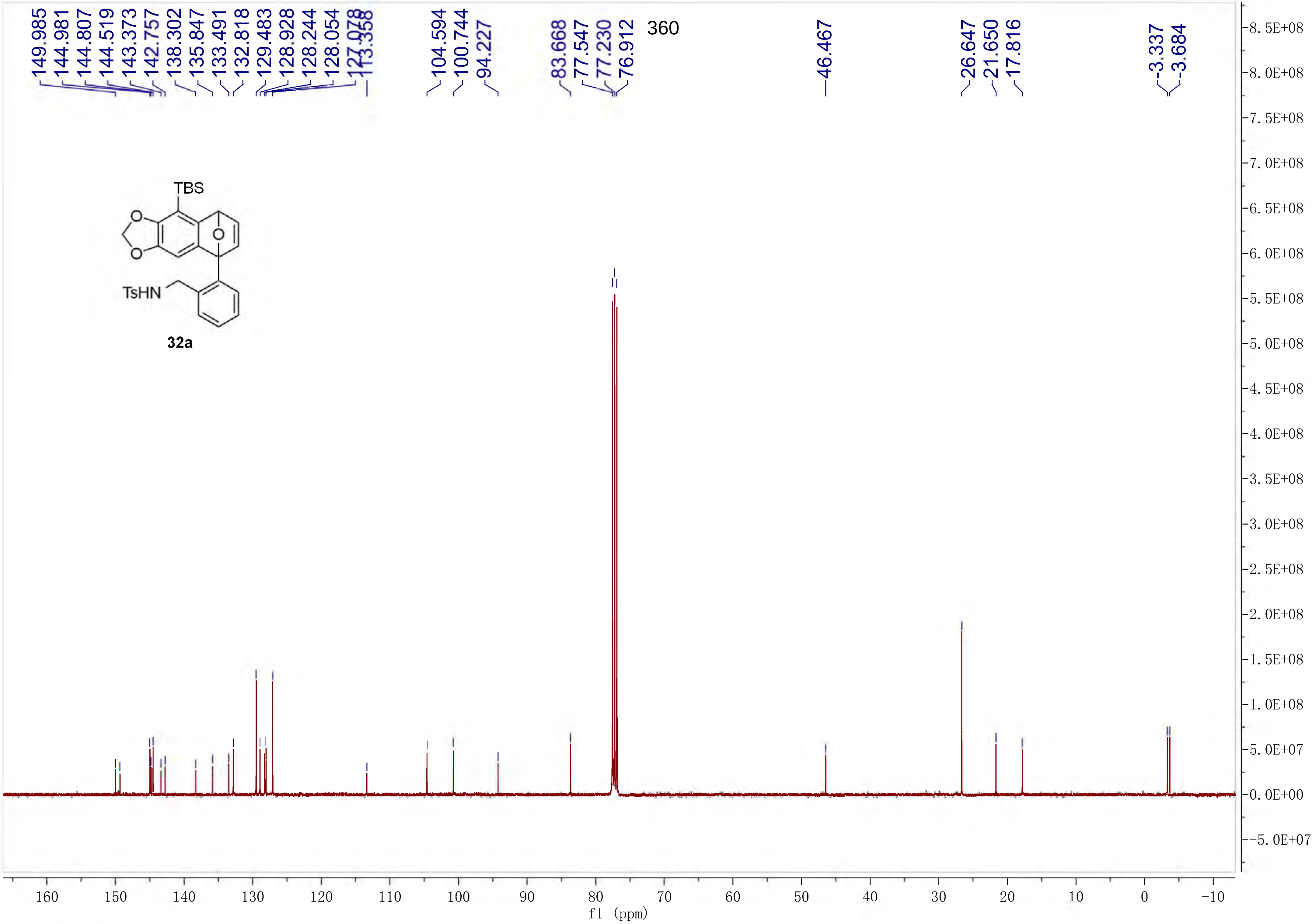

Supplementary Fig 288. ^{13}C NMR spectrum (400 MHz, $CDCl_3$, r.t.) of **32a**.

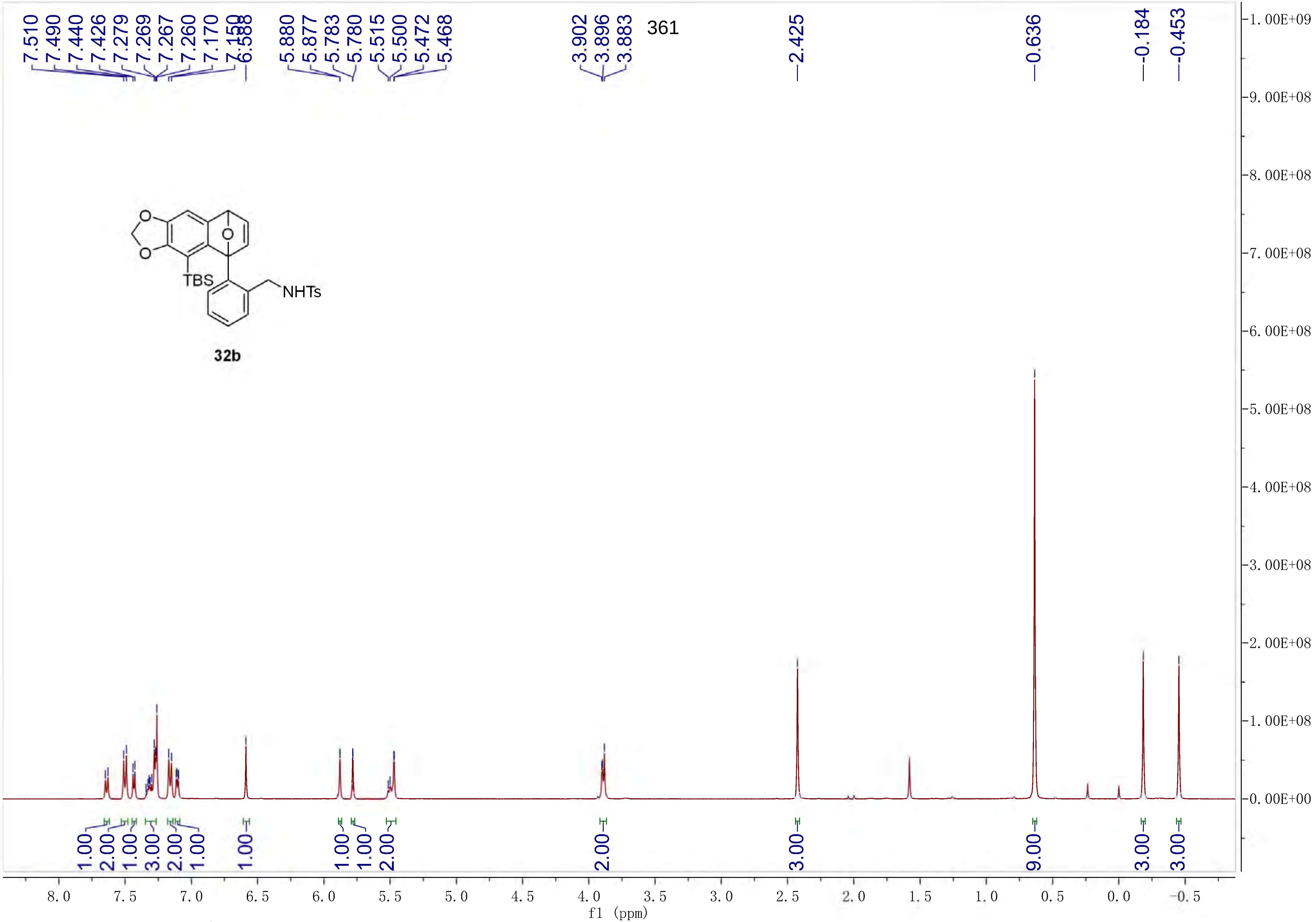

Supplementary Fig 289. ^{1}H NMR spectrum (400 MHz, $CDCl_3$, r.t.) of **32b**.

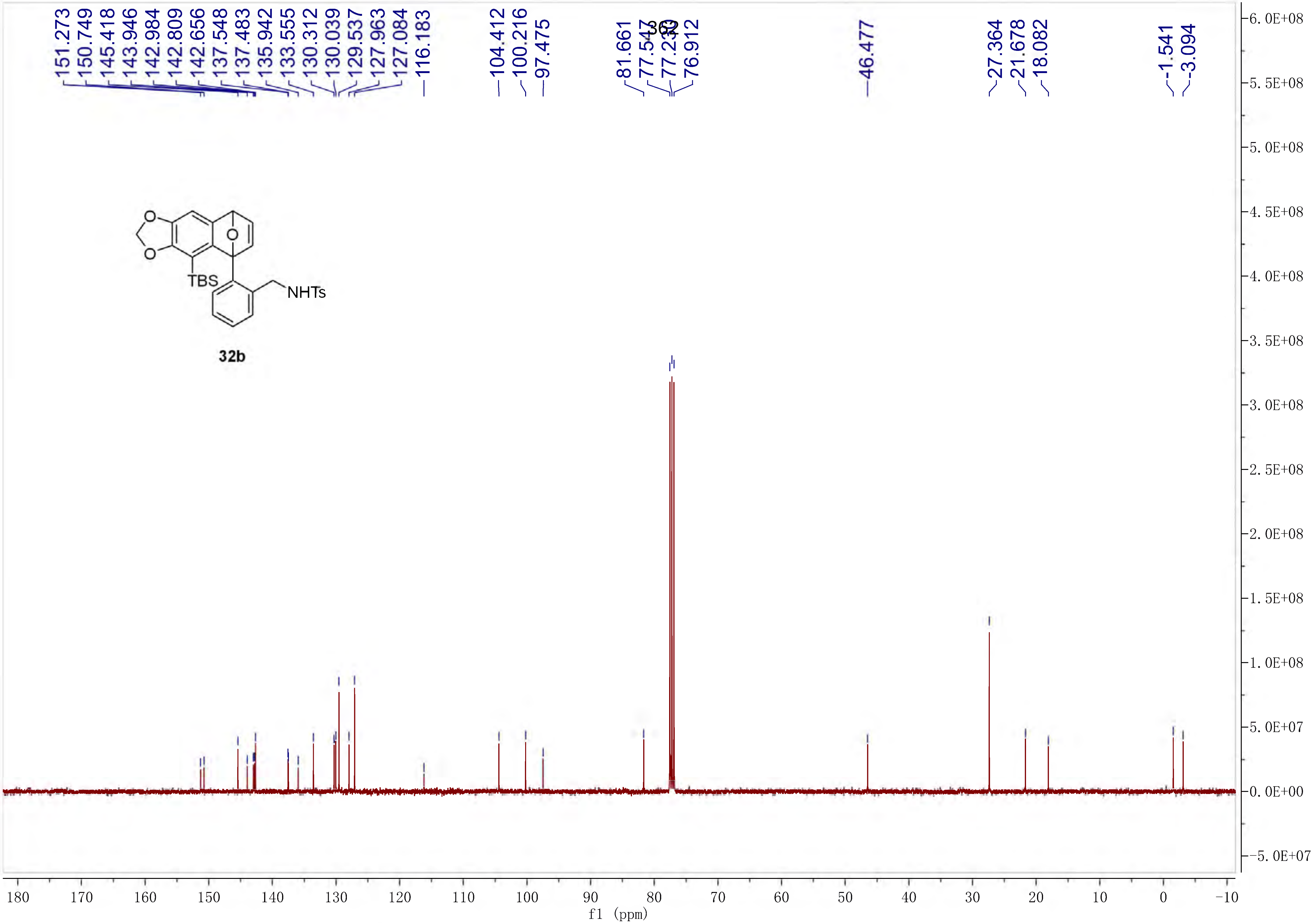

Supplementary Fig 290. ^{13}C NMR spectrum (400 MHz, $CDCl_3$, r.t.) of **32b**.

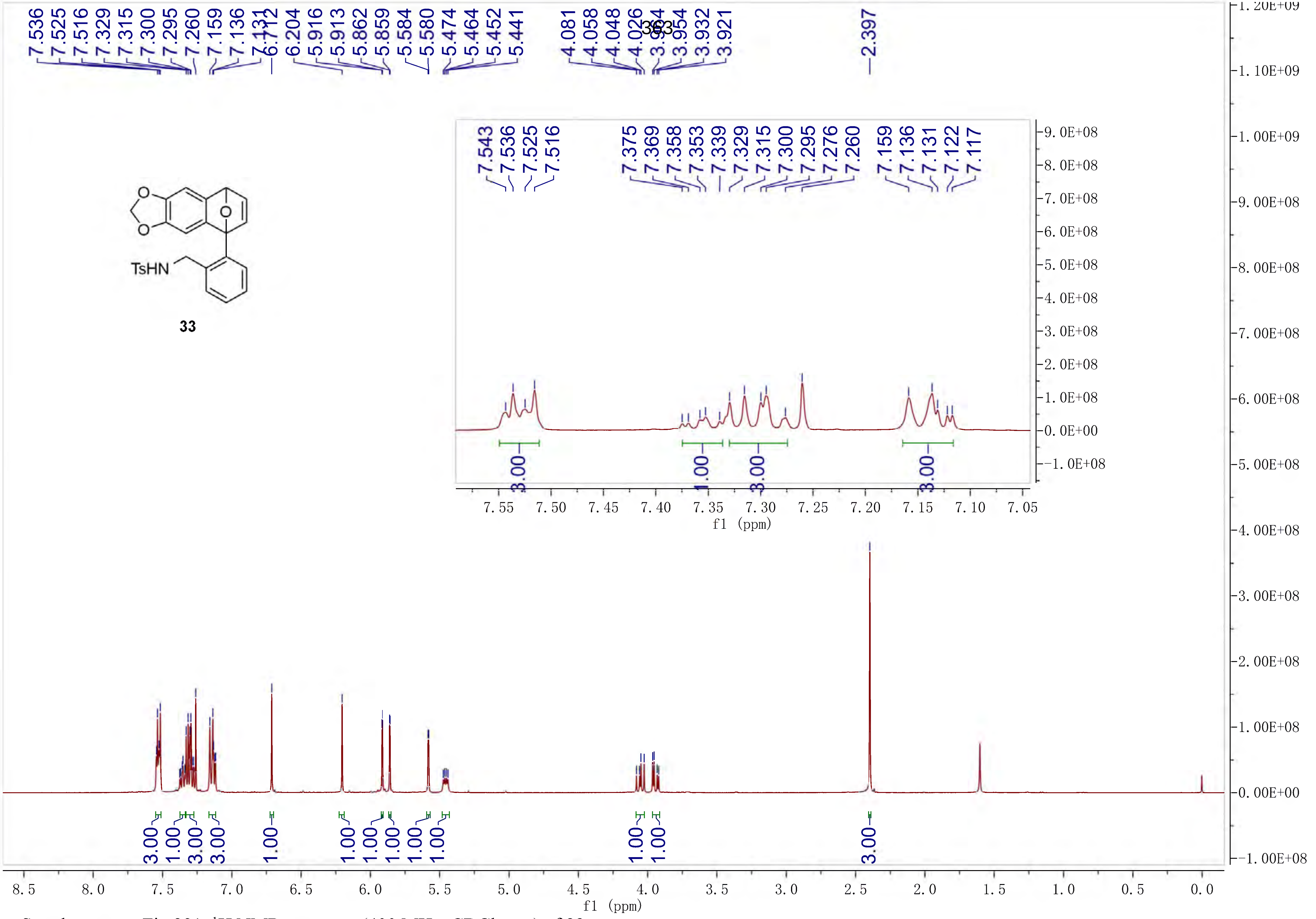

Supplementary Fig 291. ^{1}H NMR spectrum (400 MHz, $CDCl_3$, r.t.) of **33**.

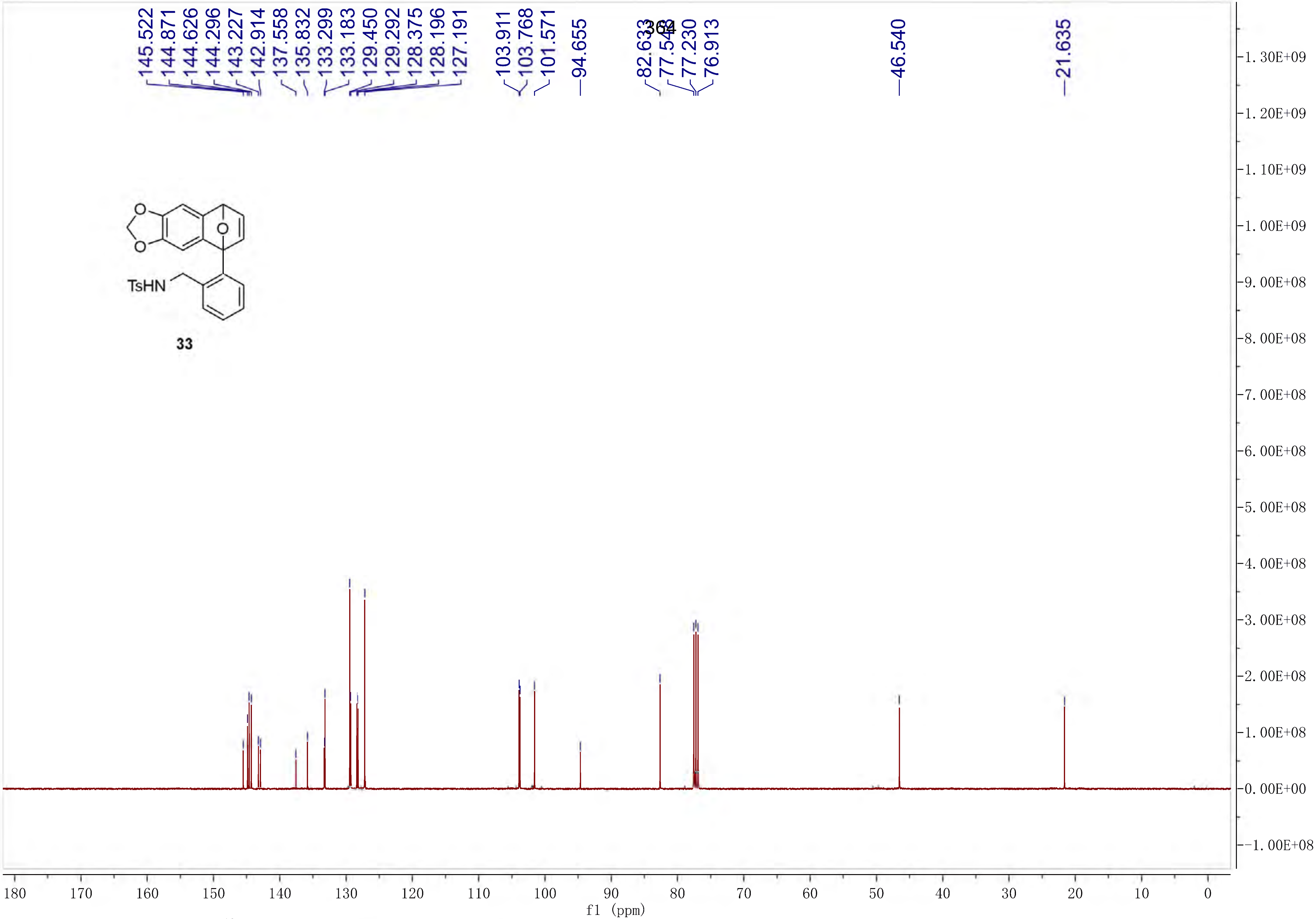

Supplementary Fig 292. ^{13}C NMR spectrum (400 MHz, $CDCl_3$, r.t.) of **33**.

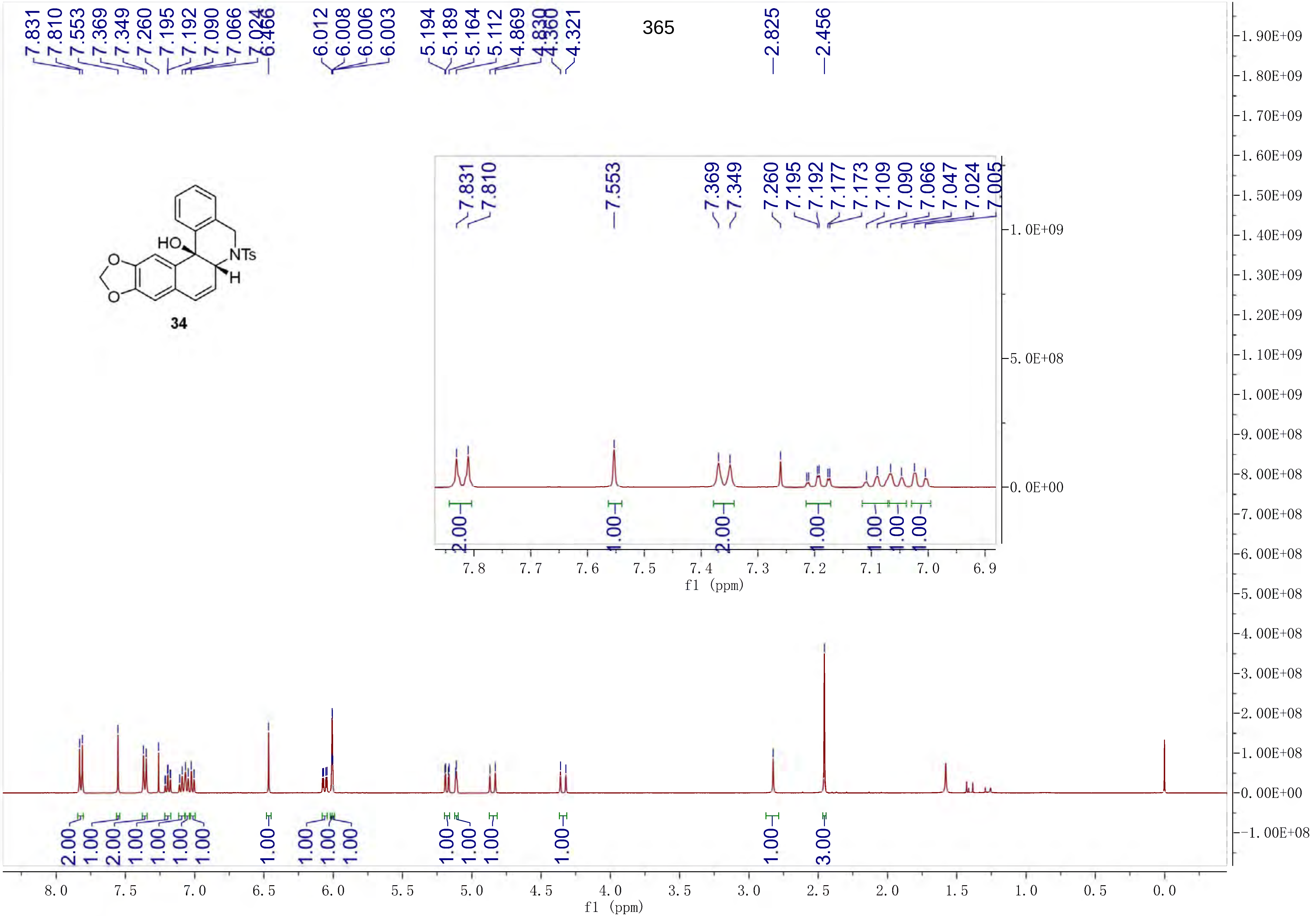

Supplementary Fig 293. ^{1}H NMR spectrum (400 MHz, $CDCl_3$, r.t.) of **34**.

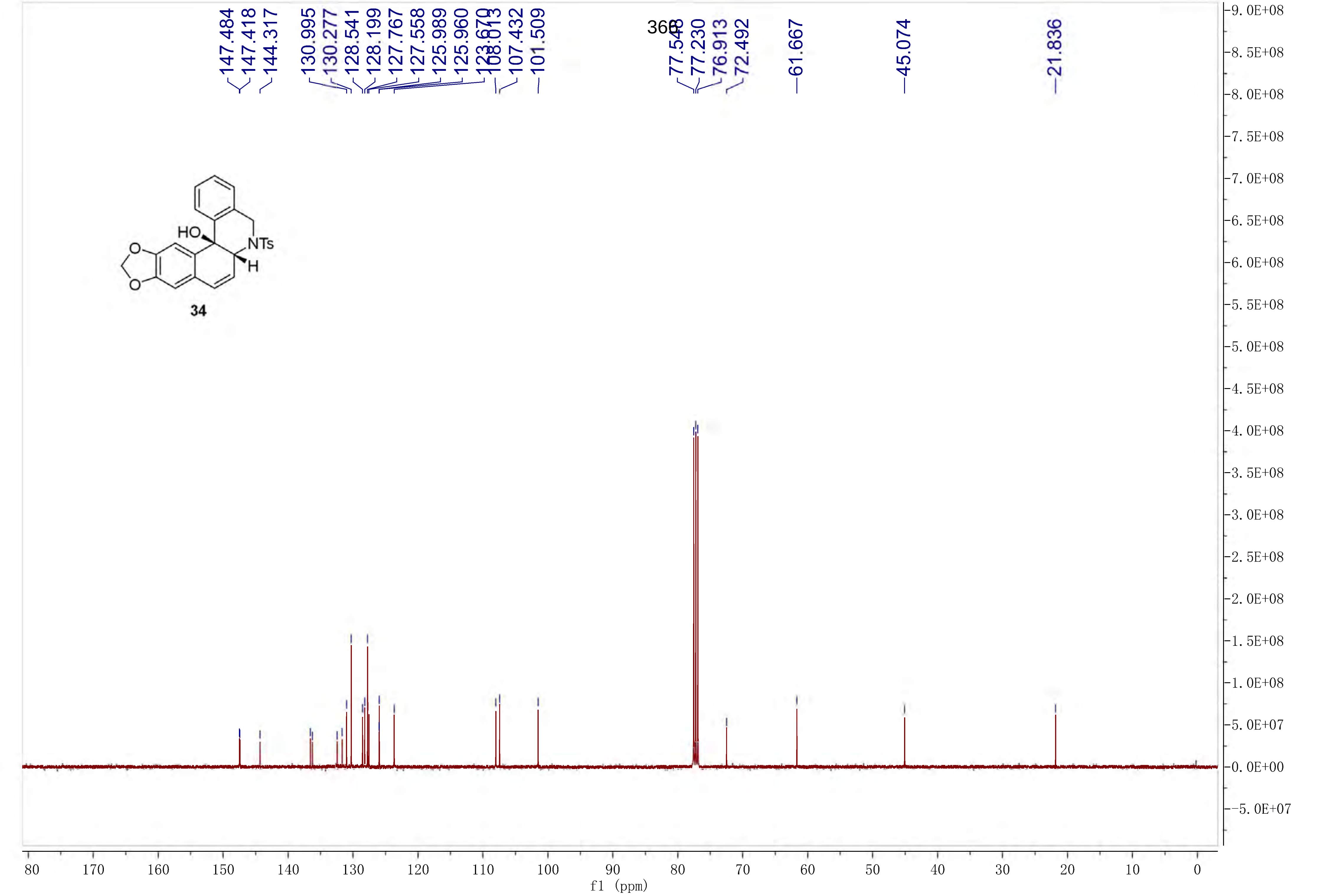

Supplementary Fig 294. ^{13}C NMR spectrum (400 MHz, $CDCl_3$, r.t.) of **34**.

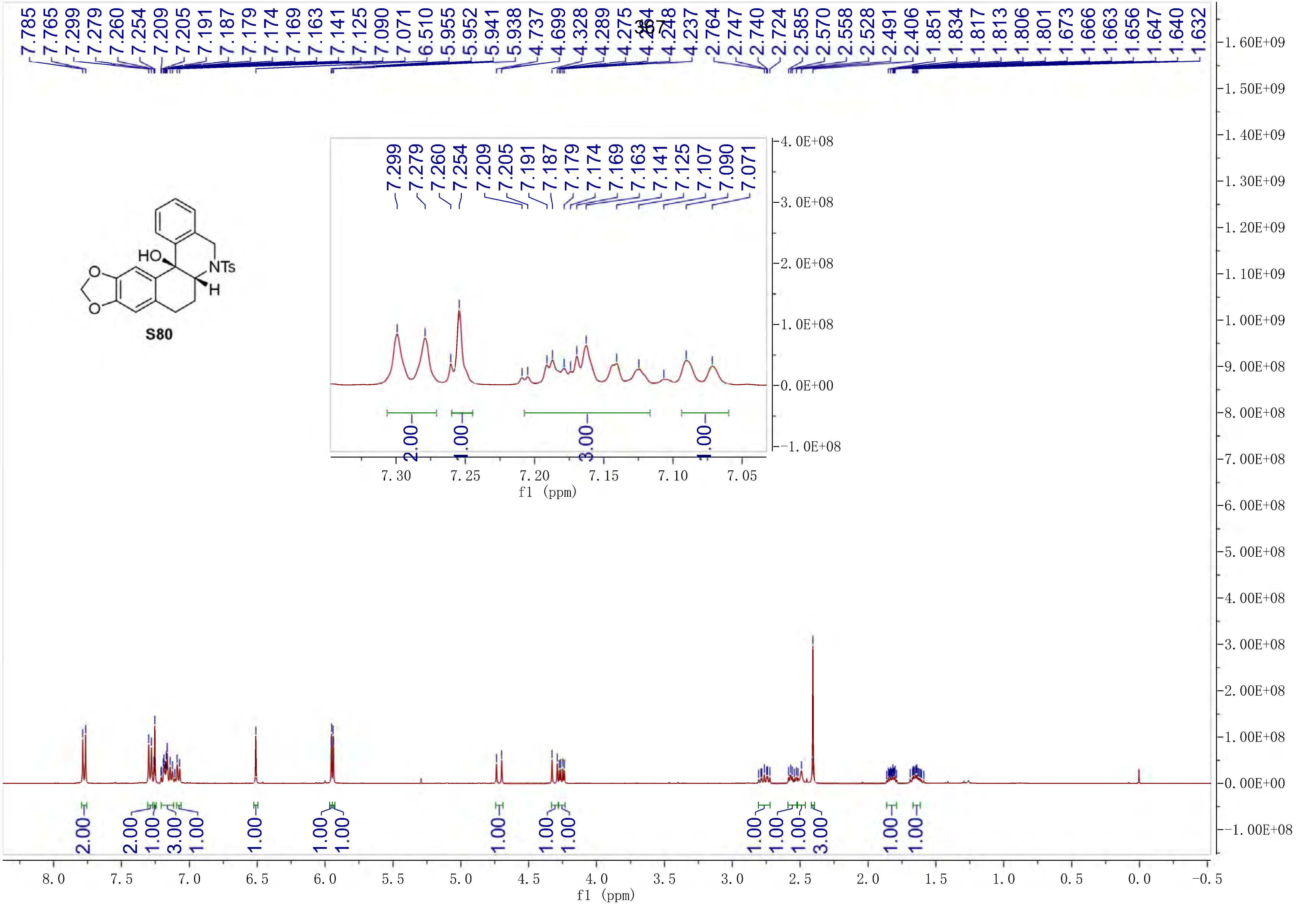

S80

Supplementary Fig 295. ^{1}H NMR spectrum (400 MHz, $CDCl_3$, r.t.) of **S80**.

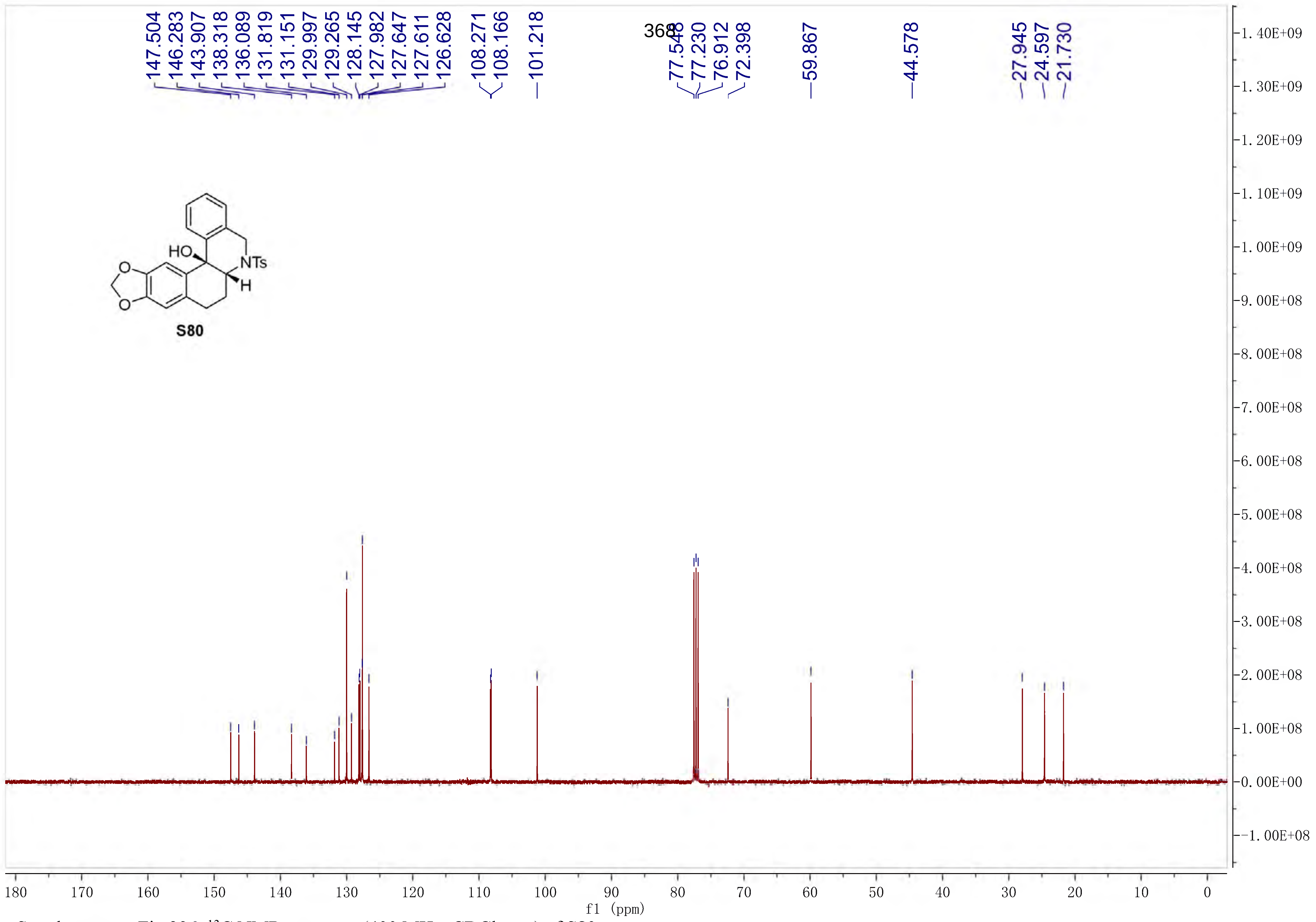

Supplementary Fig 296. ^{13}C NMR spectrum (400 MHz, $CDCl_3$, r.t.) of **S80**.

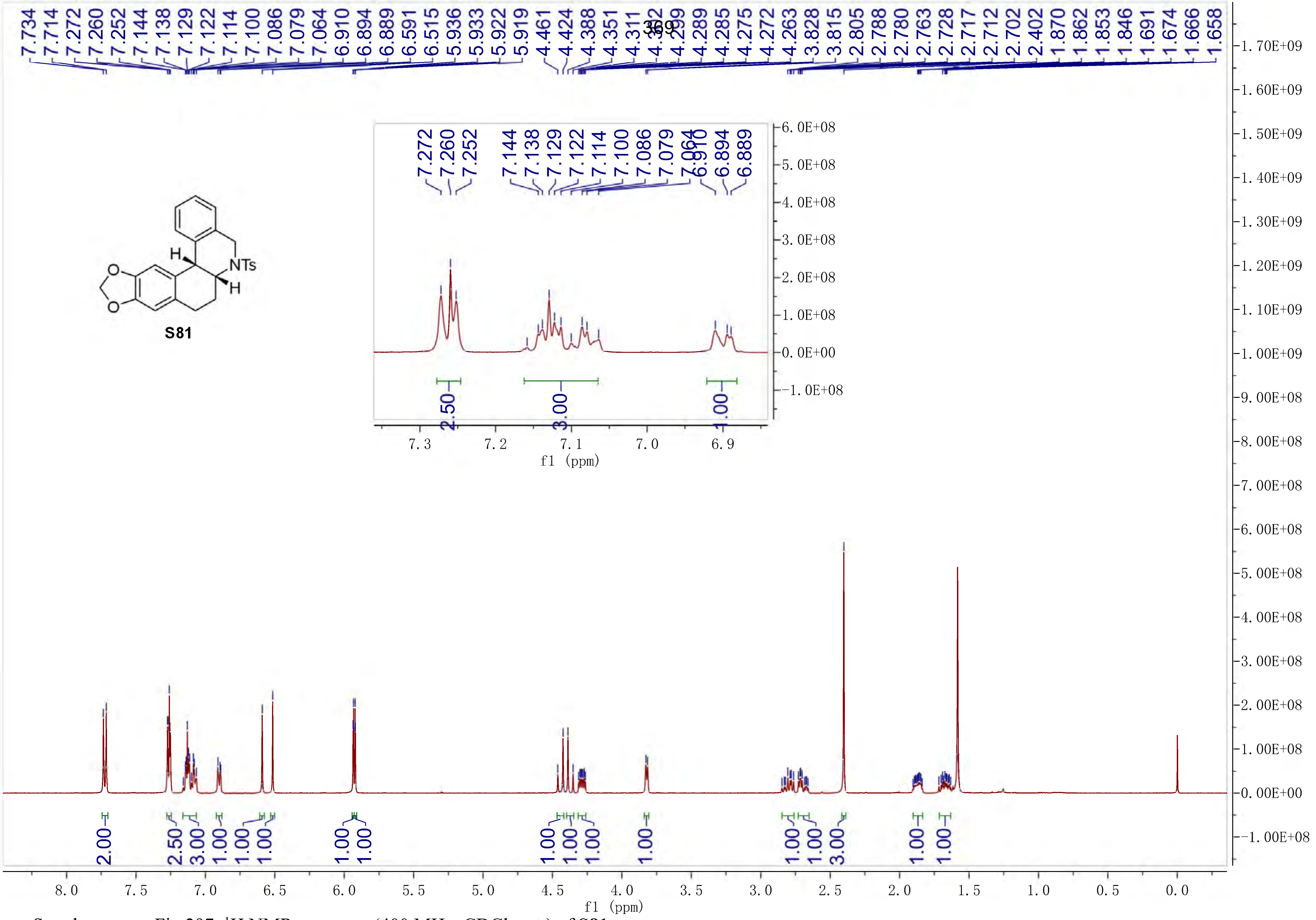

Supplementary Fig 297. ^{1}H NMR spectrum (400 MHz, $CDCl_3$, r.t.) of **S81**.

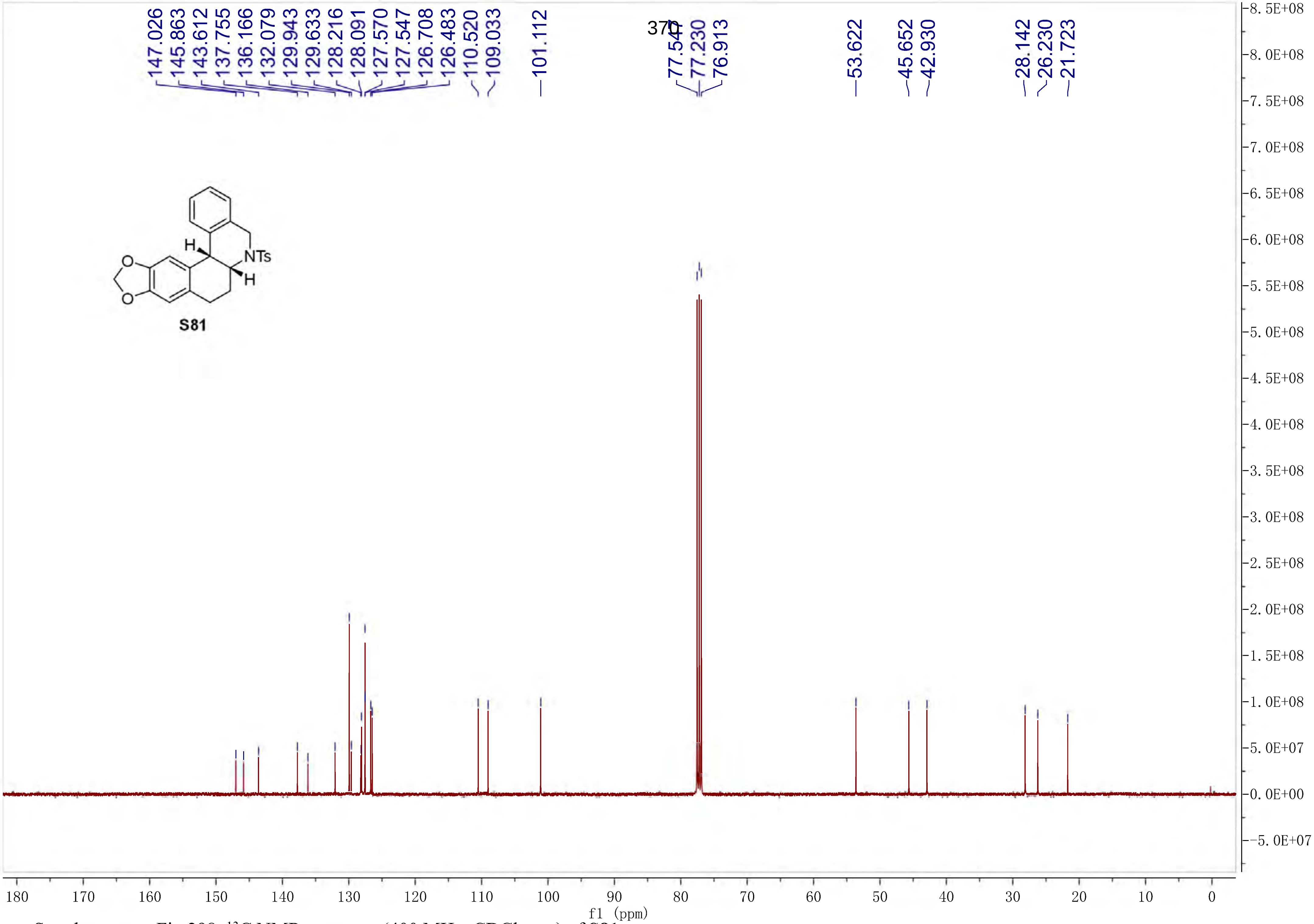

Supplementary Fig 298. ^{13}C NMR spectrum (400 MHz, $CDCl_3$, r.t.) of **S81**.

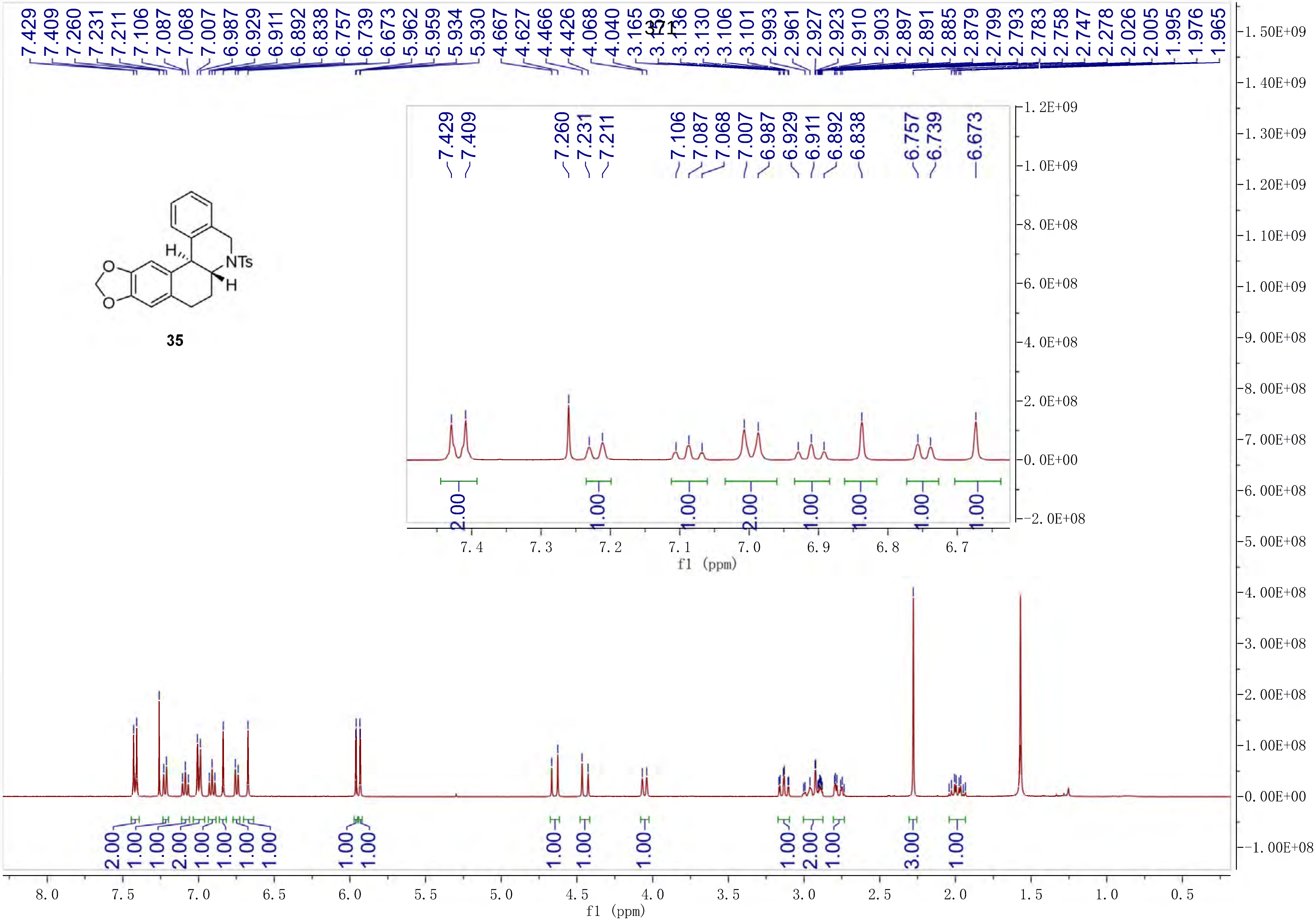

Supplementary Fig 299. ^{1}H NMR spectrum (400 MHz, $CDCl_3$, r.t.) of **35**.

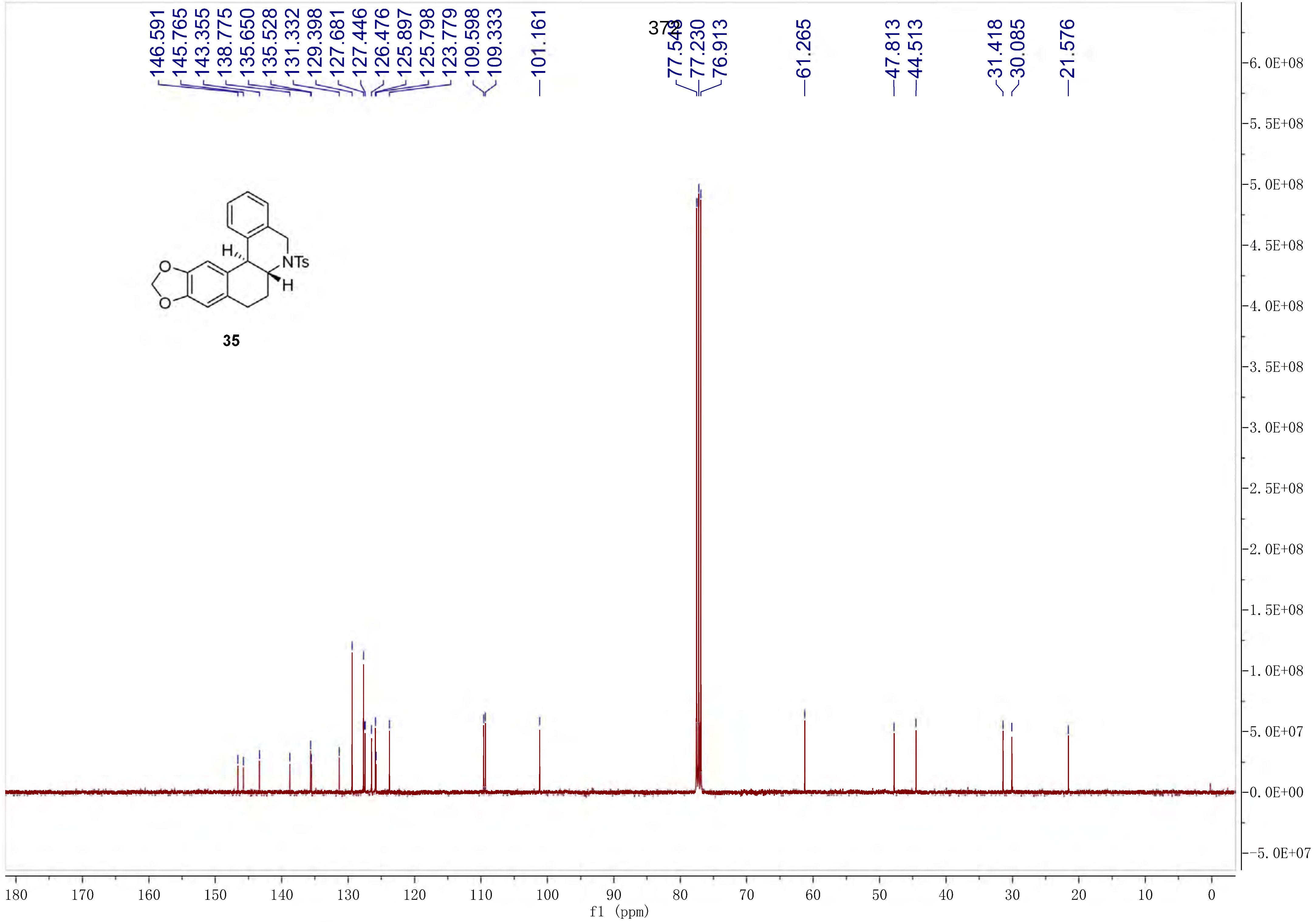

Supplementary Fig 300. ^{13}C NMR spectrum (400 MHz, $CDCl_3$, r.t.) of **35**.

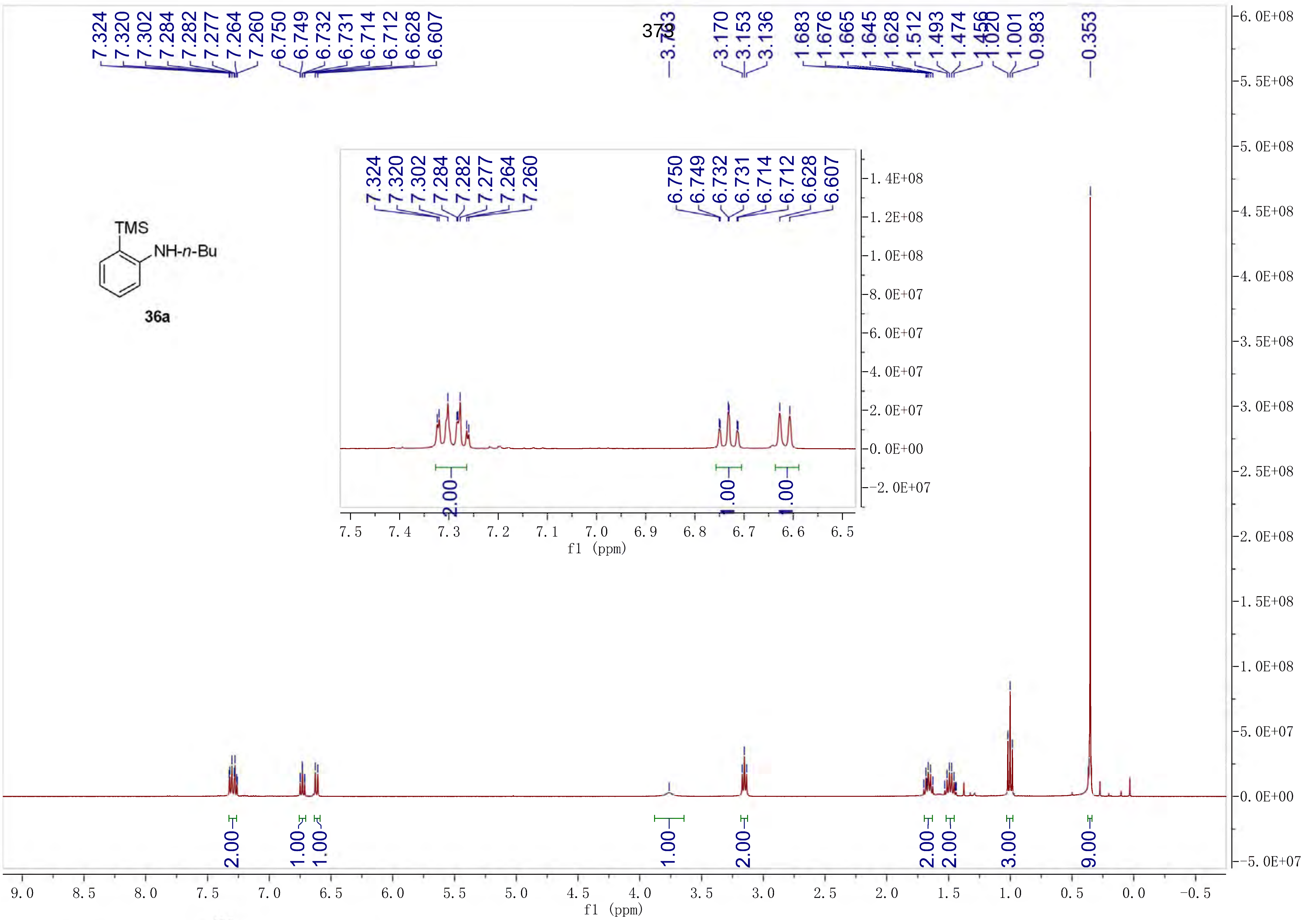

Supplementary Fig 301. ^{1}H NMR spectrum (400 MHz, $CDCl_3$, r.t.) of **36a**.

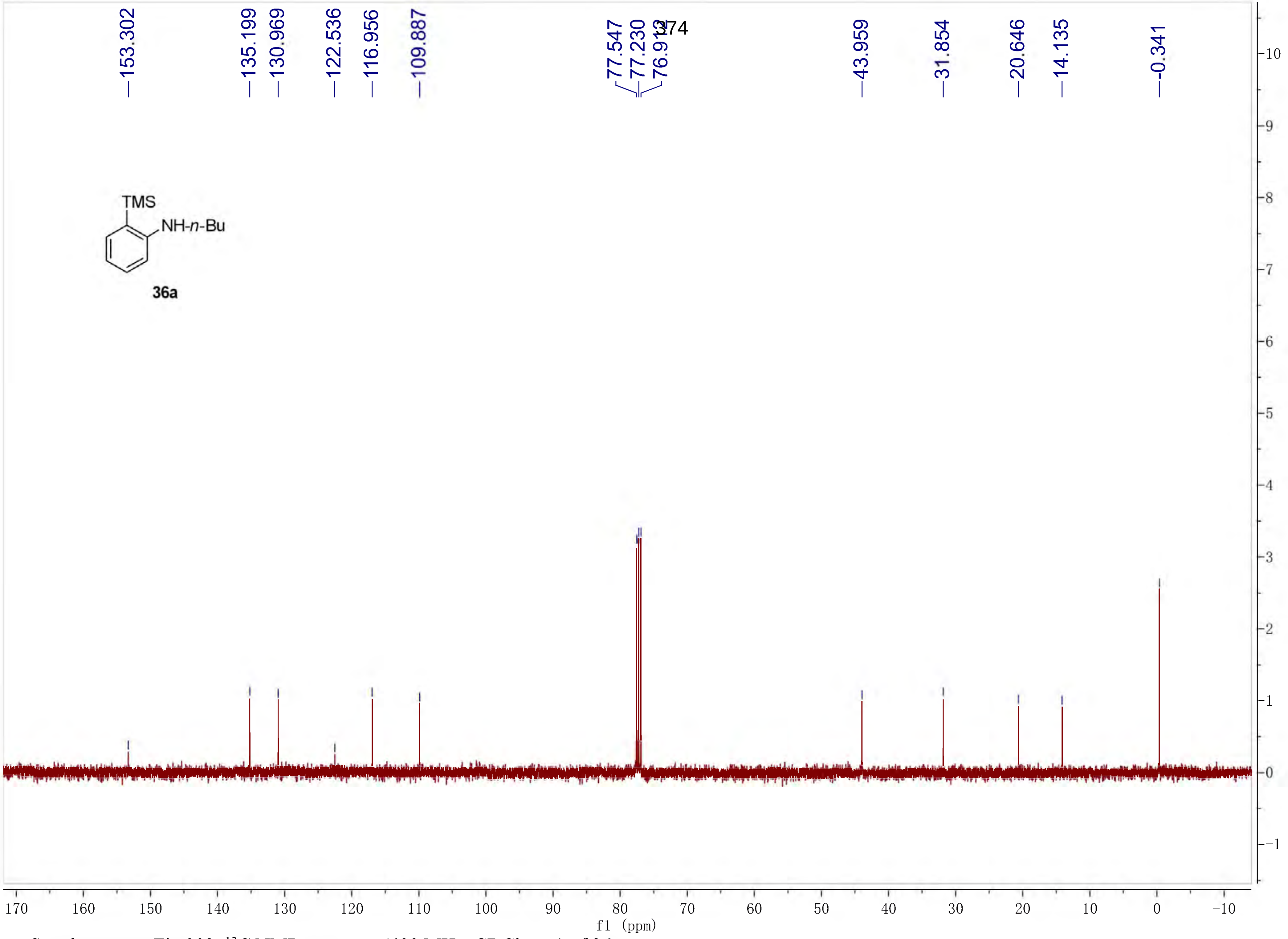

Supplementary Fig 302. ^{13}C NMR spectrum (400 MHz, $CDCl_3$, r.t.) of **36a**.

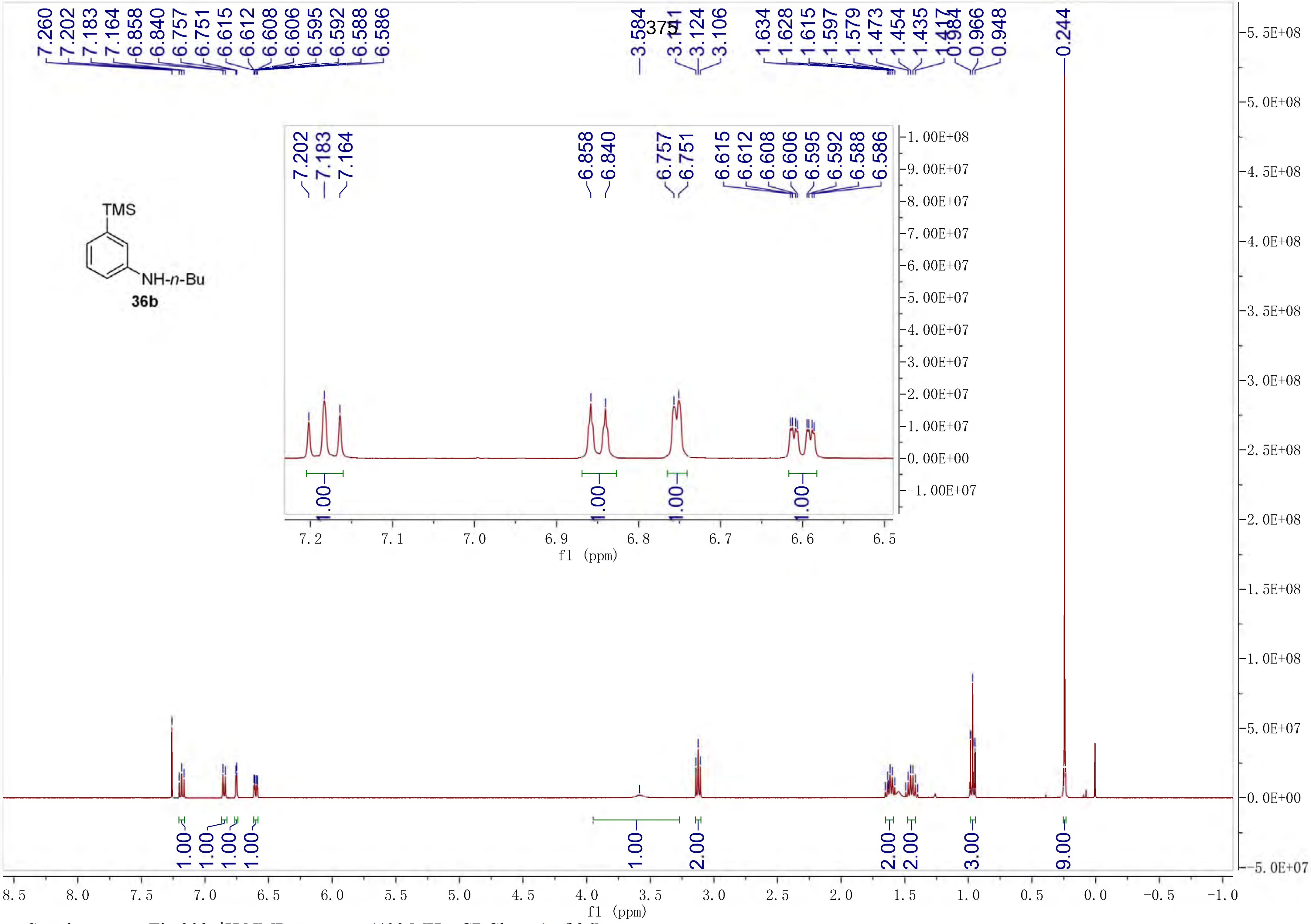

Supplementary Fig 303. ^{1}H NMR spectrum (400 MHz, $CDCl_3$, r.t.) of **36b**.

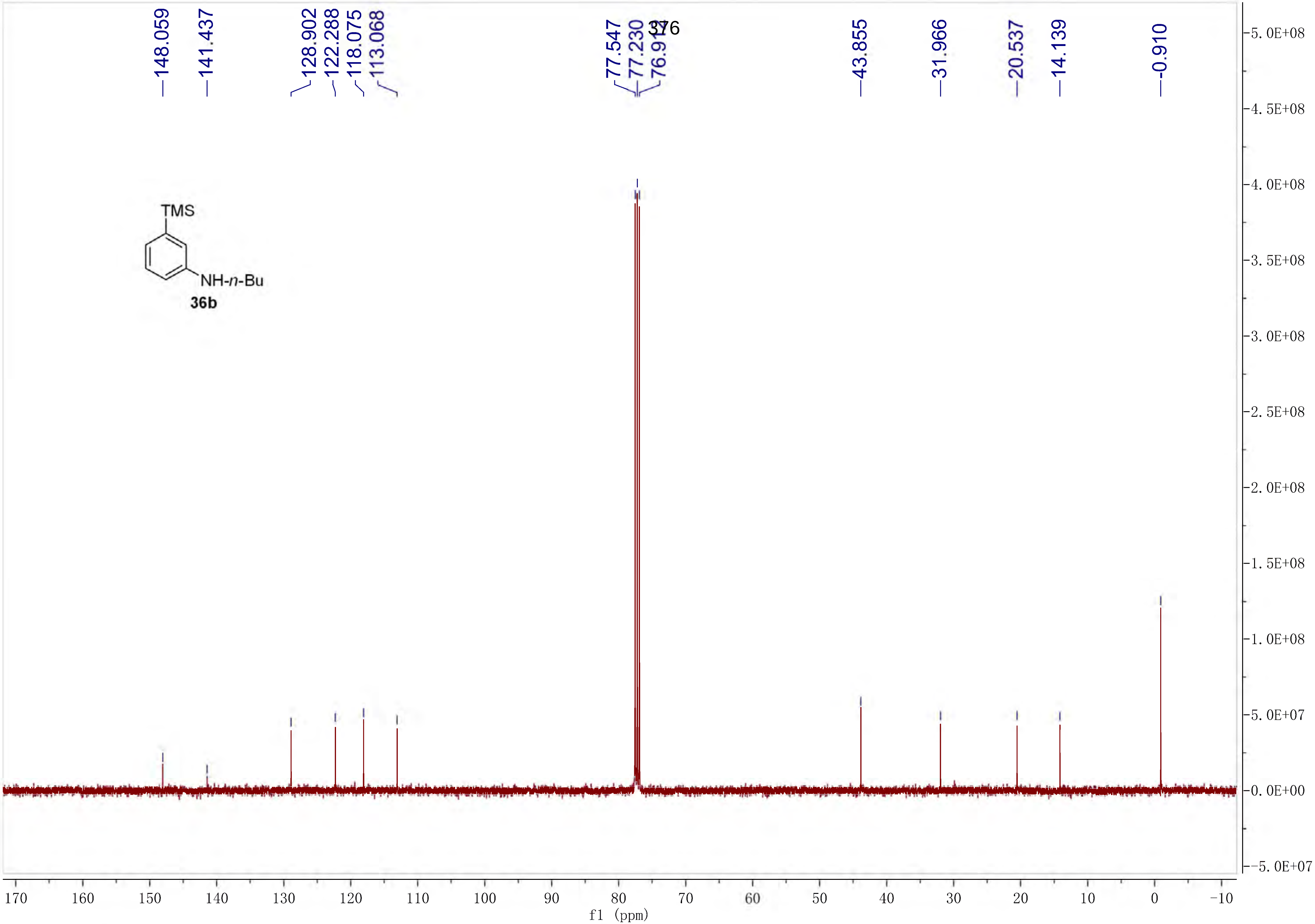

Supplementary Fig 304. ^{13}C NMR spectrum (400 MHz, $CDCl_3$, r.t.) of **36b**.

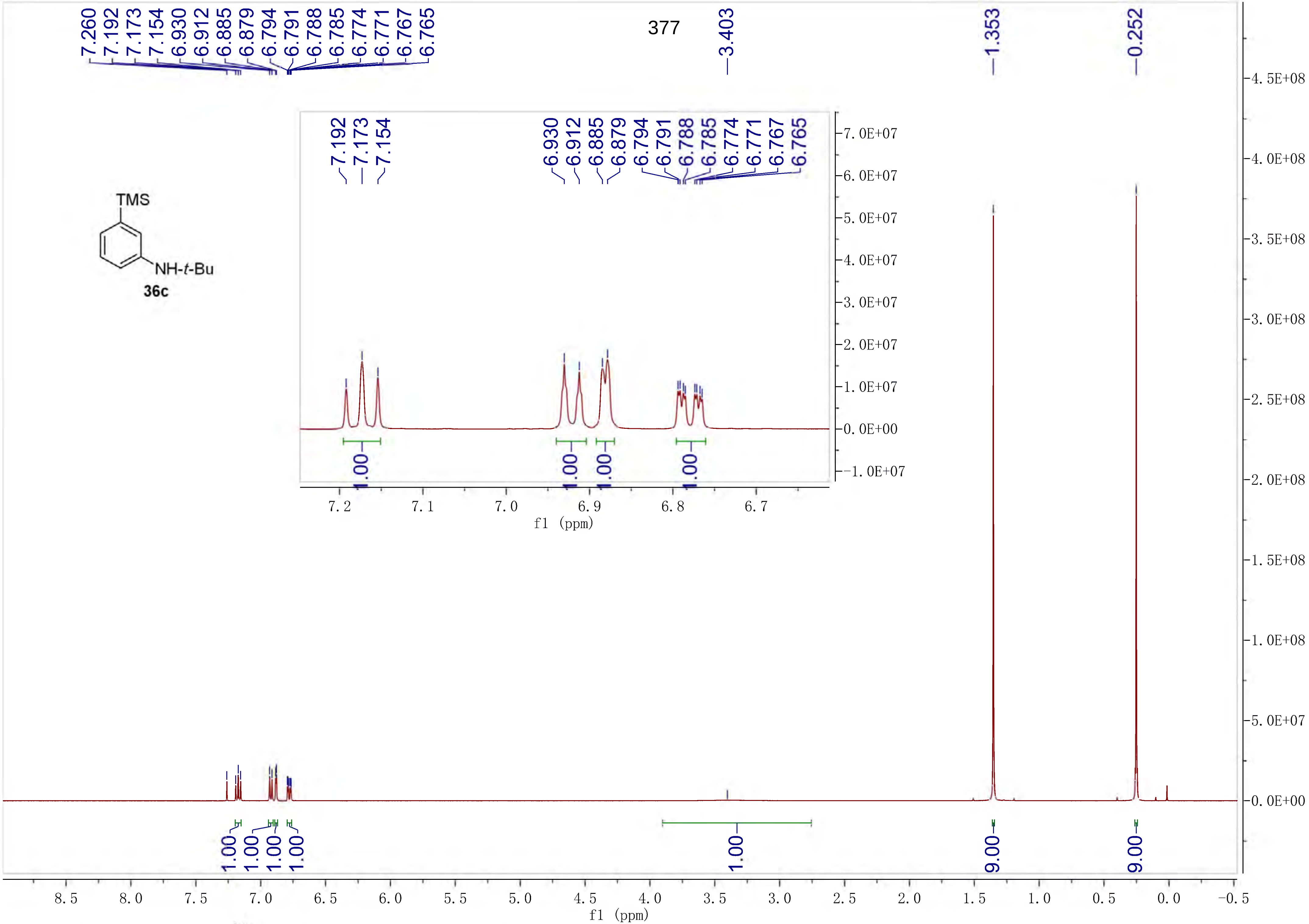

Supplementary Fig 305. ^{1}H NMR spectrum (400 MHz, $CDCl_3$, r.t.) of **36c**.

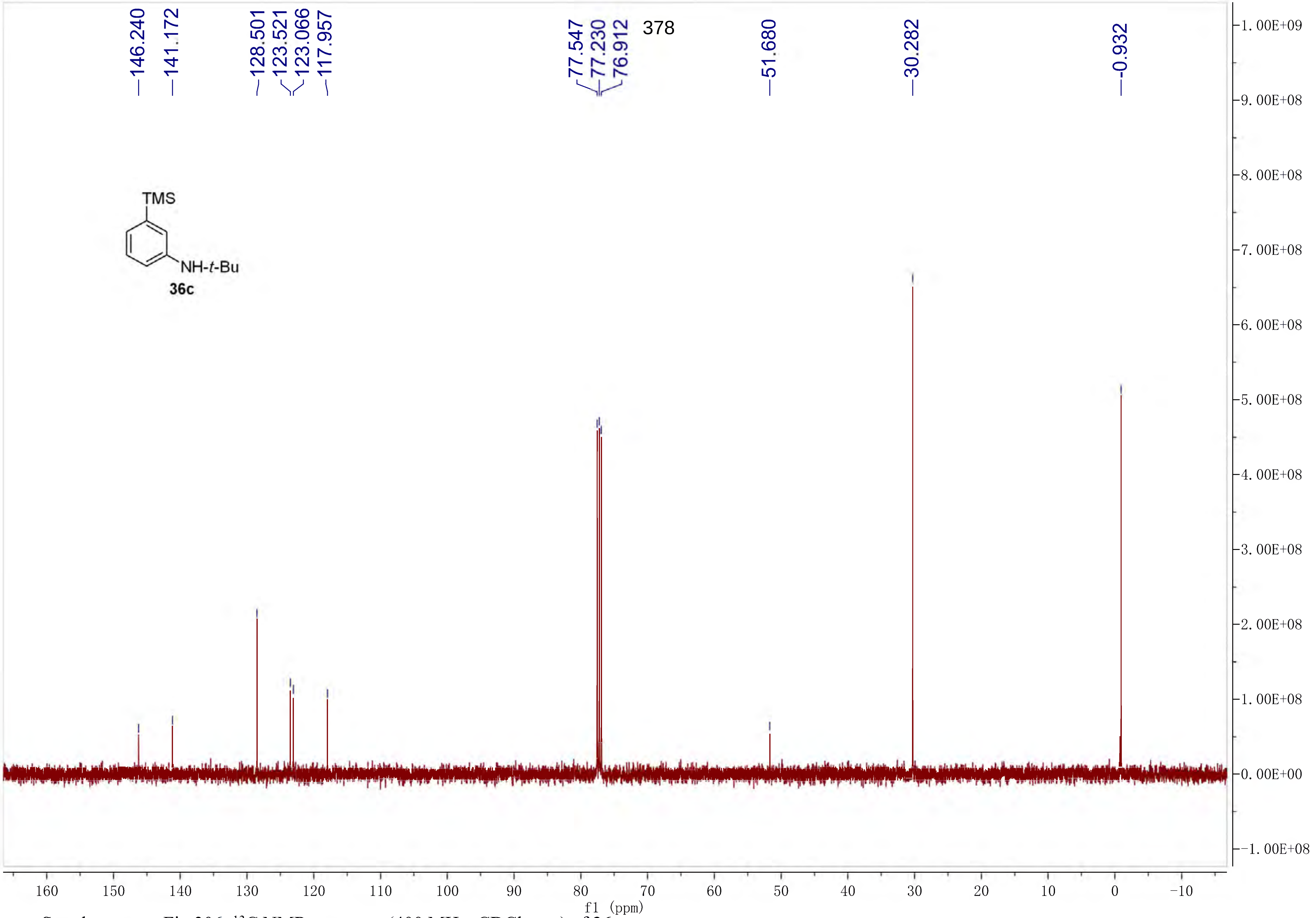

Supplementary Fig 306. ^{13}C NMR spectrum (400 MHz, $CDCl_3$, r.t.) of **36c**.

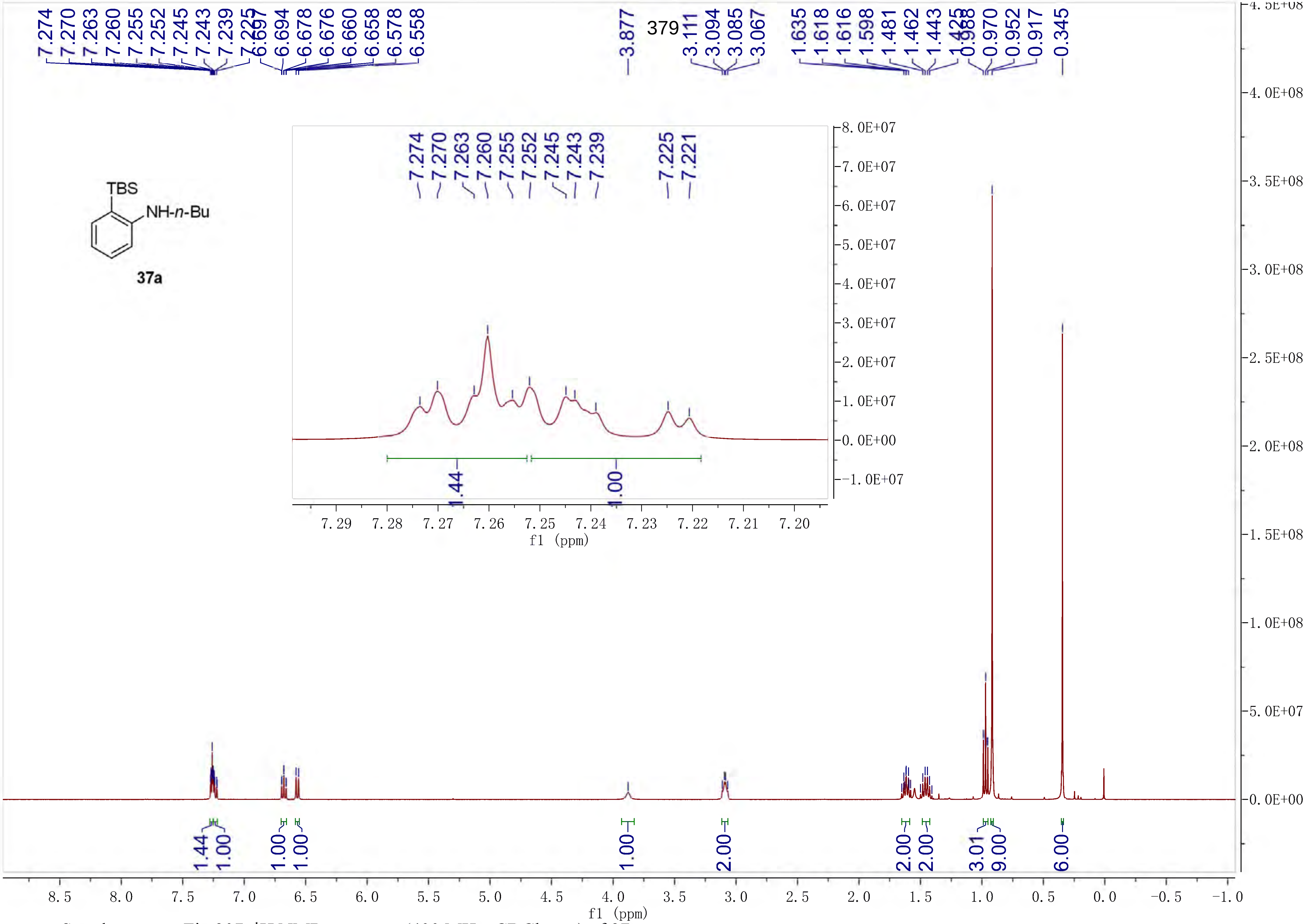

Supplementary Fig 307. ^{1}H NMR spectrum (400 MHz, $CDCl_3$, r.t.) of **37a**.

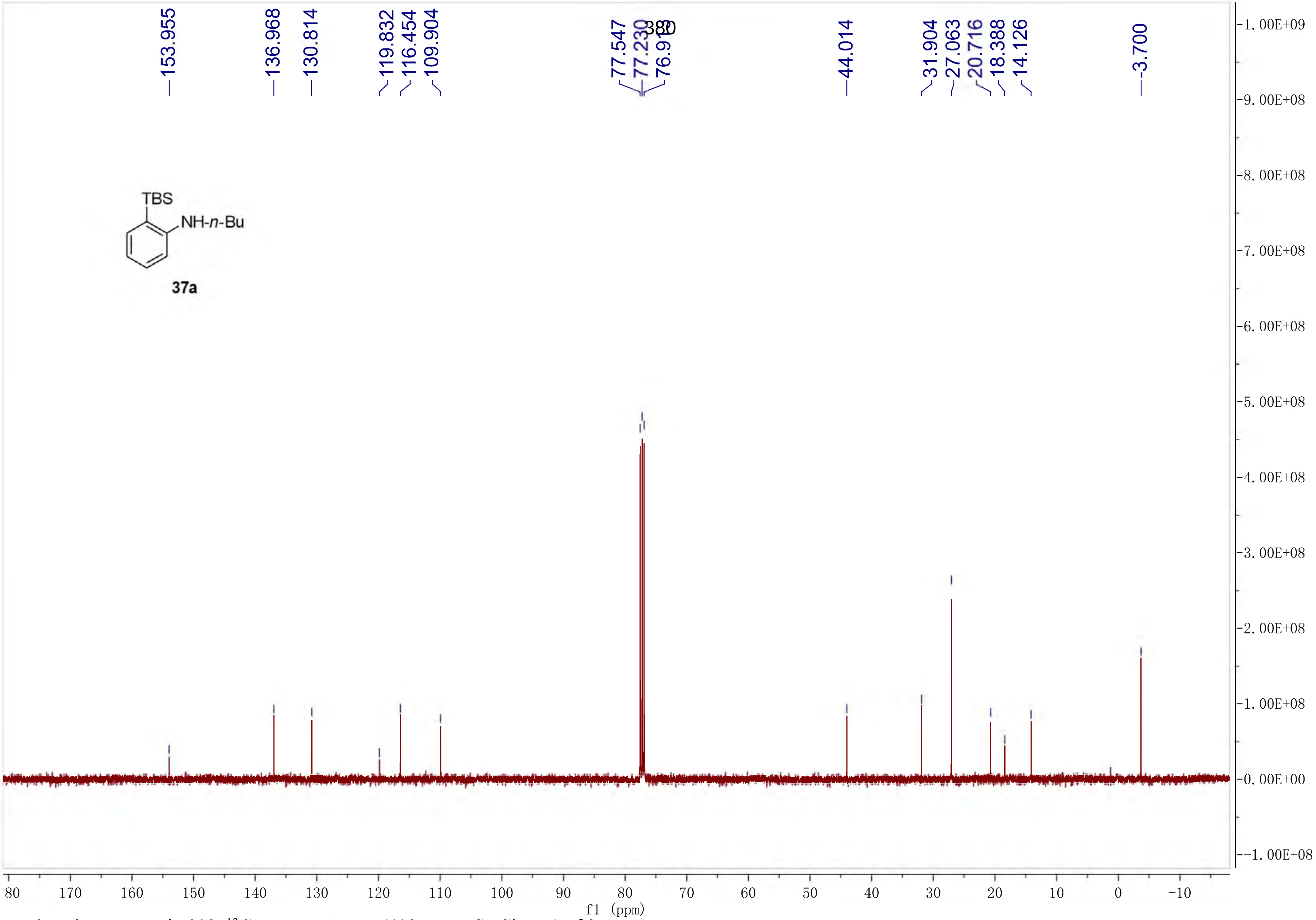

Supplementary Fig 308. ^{13}C NMR spectrum (400 MHz, $CDCl_3$, r.t.) of **37a**.

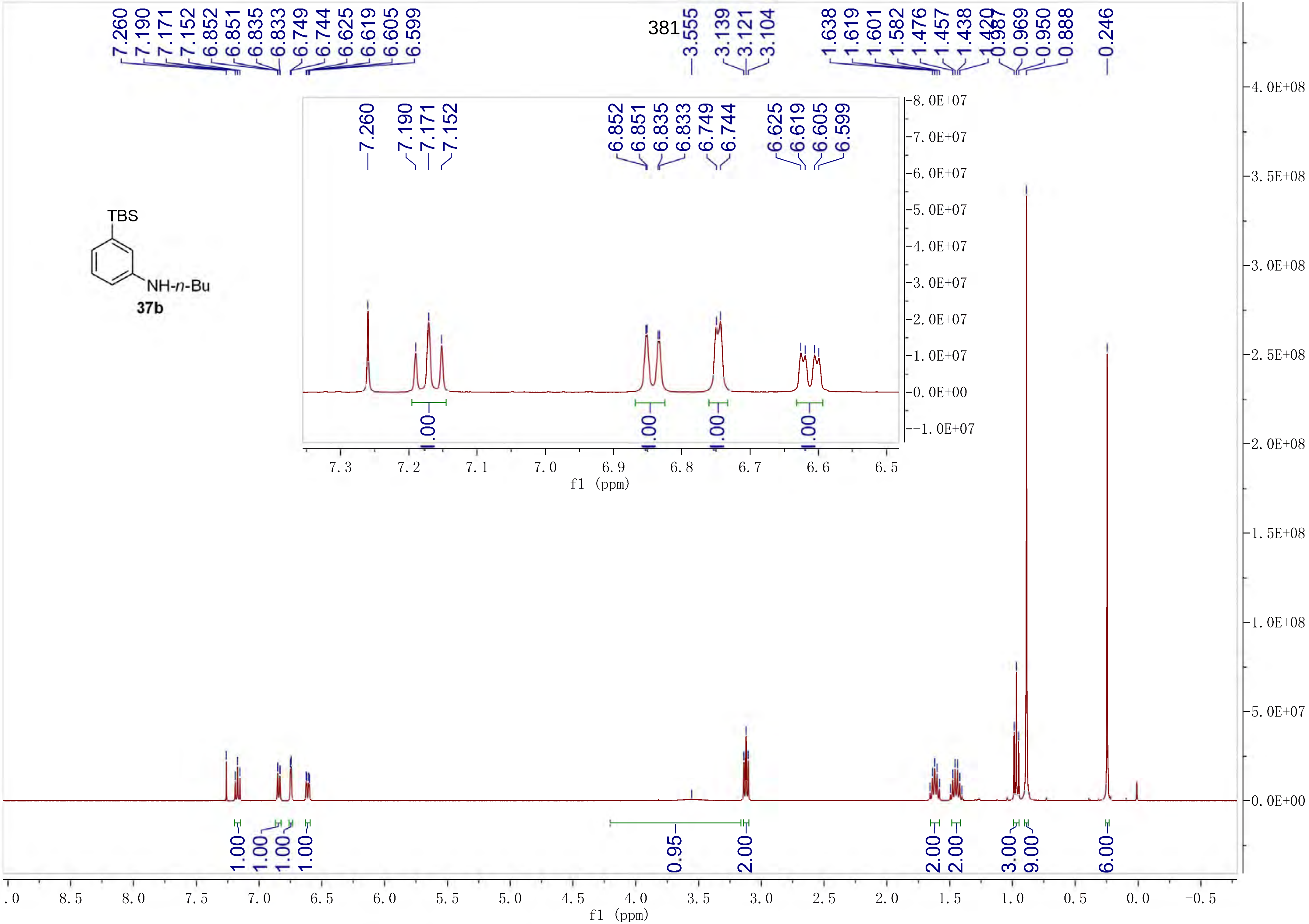

Supplementary Fig 309. ^{1}H NMR spectrum (400 MHz, $CDCl_3$, r.t.) of **37b**.

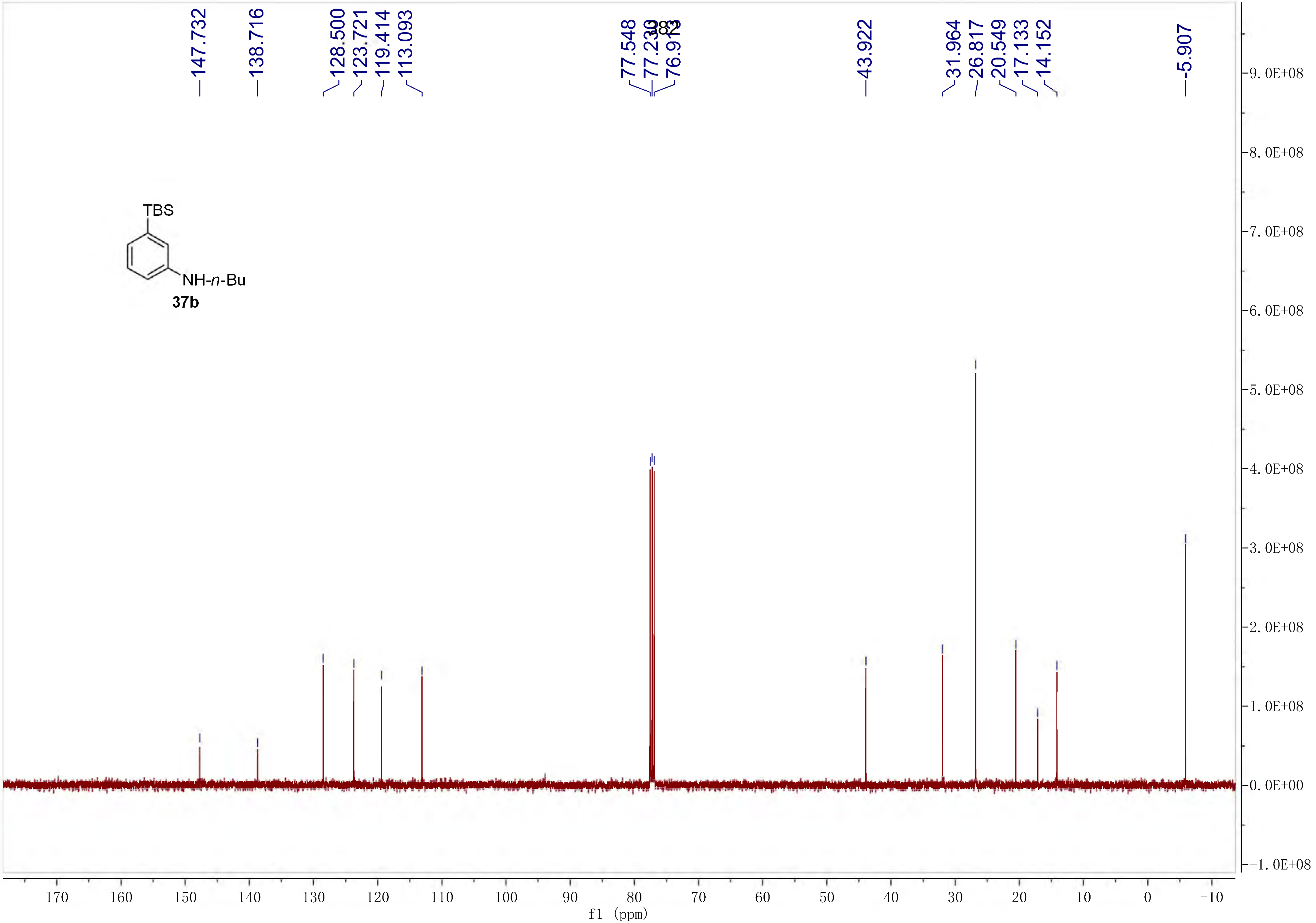

Supplementary Fig 310. ^{13}C NMR spectrum (400 MHz, $CDCl_3$, r.t.) of **37b**.

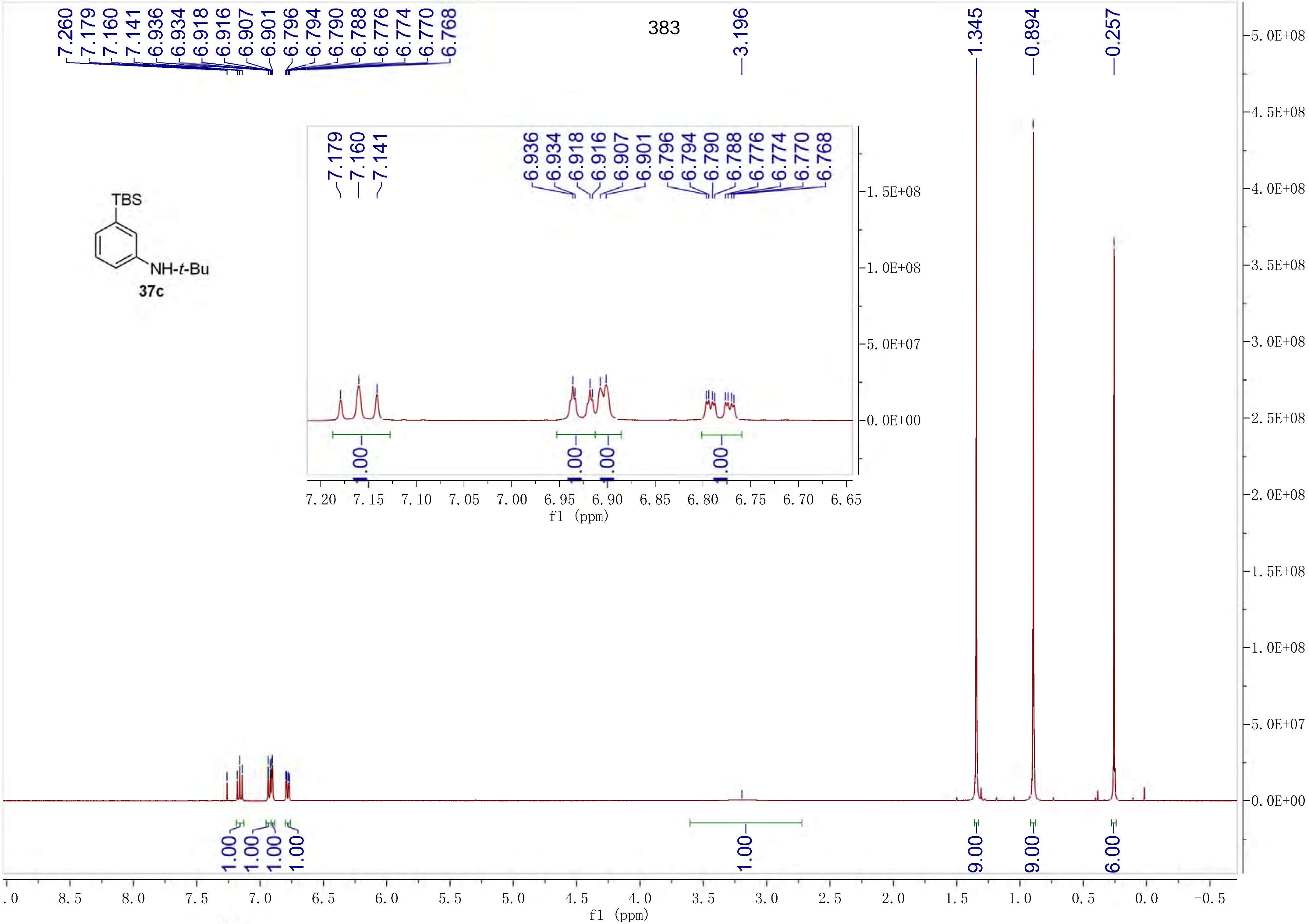

Supplementary Fig 311. ^{1}H NMR spectrum (400 MHz, $CDCl_3$, r.t.) of **37c**.

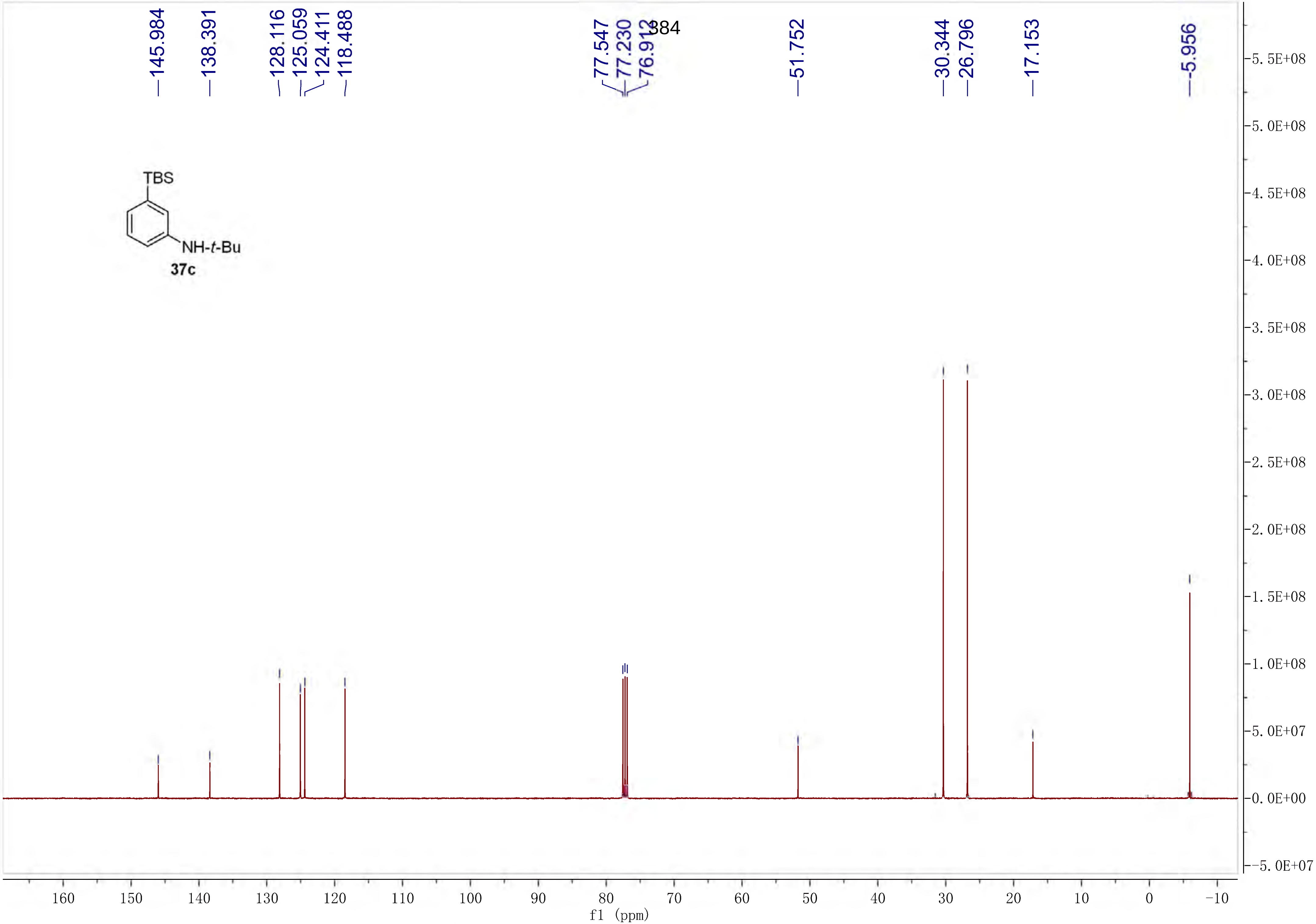

Supplementary Fig 312. ^{13}C NMR spectrum (400 MHz, $CDCl_3$, r.t.) of **37c**.

4. Supplementary References

1. Ueda, H.; Yoshida, K. & Tokuyama, H. Acetic Acid Promoted Metal-Free Aerobic Carbon−Carbon Bond Forming Reactions at *α*-Position of Tertiary Amines. *Org. Lett.* **16**, 4194−4197 (2014).
2. Zhou, C.; Wang, J.; Jin, J.; Lu, P. & Wang, Y. Three-Component Synthesis of α-Amino-α-aryl Carbonitriles from Arynes, Aroyl Cyanides, and *N*,*N*-Dimethylformamide. *Eur. J. Org. Chem.* **9**, 1832−1835 (2014).
3. Shi, J. et al. Aryne 1,2,3,5-Tetrasubstitution Enabled by 3-Silylaryne and Allyl Sulfoxide via an Aromatic 1,3-Silyl Migration. *J. Am. Chem. Soc*. **143**, 2178−2184 (2021).
4. Medina, J. M.; Mackey, J. L.; Garg, N. K. & Houk, K. N. The Role of Aryne Distortions, Steric Effects, and Charges in Regioselectivities of Aryne Reactions. *J. Am. Chem. Soc*. **136**, 15798−15805 (2014).
5. Bronner, S. M.; Mackey, J. L.; Houk, K. N. & Garg, N. K. Steric Effects Compete with Aryne Distortion To Control Regioselectivities of Nucleophilic Additions to 3-Silylarynes. *J. Am. Chem. Soc*. **134**, 13966−13969 (2012).
6. Dai, M.; Wang, Z. & Danishefsky, S. J. A novel α,β-unsaturated nitrone-aryne [3+2] cycloaddition and its application in the synthesis of the cortistatin core. *Tetrahedron Letters*. **49**, 6613−6616 (2008).
7. Jiang, H. et al. A Three-Phase Four-Component Coupling Reaction: Selective Synthesis of *o*-Chloro Benzoates by KCl, Arynes, CO_2, and Chloroalkanes. *Org. Lett.* **21**, 345−349 (2019).
8. Yudasaka, M.; Shimbo, D.; Maruyama, T.; Tada, N. & Itoh, A. Synthesis, Characterization, and Reactivity of an Ethynyl Benziodoxolone (EBX)−Acetonitrile Complex. *Org. Lett.* **21**, 1098−1102 (2019).
9. Tanaka, K.; Asada, Y.; Hoshino, Y. & Honda, K. Visible-light-induced [4 + 2] cycloaddition of pentafulvenes by organic photoredox catalysis. *Org. Biomol. Chem.* **18**, 8074−8078 (2020).
10. Stein, A. et al. Some Surprising Transformations of Colchicone and Other Colchicine-Derived Tropolones. *Eur. J. Org. Chem.* **46**, 6375−6382 (2021).
11. Li, Z.; Meng, F.; Zhang, J.; Xie, J. & Dai, B. Efficient and recyclable copper-based MOF-catalyzed *N*-arylation of *N*-containing heterocycles with aryliodides. *Org. Biomol. Chem.* **14**, 10861−10865 (2020).
12. Li, L.; Li, Y.; Fu, N.; Zhang, L. & Luo, S. Catalytic Asymmetric Electrochemical α-Arylation of Cyclic β-Ketocarbonyls with Anodic Benzyne Intermediates. *Angew. Chem. Int. Ed*. **59**, 14347−14351 (2022).
13. Inaloo, I. D.; Majnooni, S.; Eslahi, H. & Esmaeilpour, M. *N*-Arylation of (hetero)arylamines using aryl sulfamates and carbamates via C–O bond activation enabled by a reusable and durable nickel(0) catalyst. *New J. Chem.* **44**, 13266–13278 (2020).
14. Diallo, A. G.; Roy, D.; Gaillard, S.; Lautens, M. & Renaud, J-L. Aminomethylation of Oxabenzonorbornadienes via the Merger of Photoredox and Nickel Catalysis. *Org. Lett.* **22**, 2442–2447 (2020).
15. Ferlin, F. et al. A waste-minimized protocol for copper-catalyzed Ullmann-type reaction in a biomass derived furfuryl alcohol/water azeotrope. *Green Chem*. **20**, 1634–1639 (2018).
16. Nilova, A.; Metze, B. & Stuart, D. R. Aryl(TMP)iodonium Tosylate Reagents as a Strategic Entry Point to Diverse Aryl Intermediates: Selective Access to Arynes. *Org. Lett.* **23**, 4813–4817 (2021).
17. Masson, E. & Schlosser, M. Fluoronaphthalene Building Blocks via Arynes: A Solution to the Problem of Positional Selectivity. *Eur. J. Org. Chem.* **20**, 4401–4405 (2005).

18. Lübbering, T.; Dutschke, P. D.; Hepp, A. & Hahn, F. E. Reactivity of Ruthenium(II) Complexes Bearing Bis-NHC Ligands. *Organometallics* **40**, 3775–3784 (2021).
19. 19. Maity, T.; Ghosh, P.; Das, S.; Sahaac, D. & Koner, S. A post-synthetically modified metal–organic framework for copper catalyzed denitrative C–N coupling of nitroarenes under heterogeneous condition*s*. *New J. Chem.* **45**, 5568–5575 (2021).
20. Sundalam, S. K.; Nilova, A.; Seidl, T. L. & Stuart, D. R. A Selective C–H Deprotonation Strategy to Access Functionalized Arynes by Using Hypervalent Iodine. *Angew. Chem. Int. Ed*. **55**, 8431–8434 (2016).
21. Rodriguez, J.; Adet, N.; Saffffon-Merceron, N. & Bourissou, D. Au(I)/Au(III)-Catalyzed C–N coupling. *Chem. Commun*. **56**, 94–97 (2020).
22. Guo, C.; Zhang, F.; Yu, C. & Luo, Y. Reduction of Amides to Amines with Pinacolborane Catalyzed by Heterogeneous Lanthanum Catalyst La($CH_2C_6H_4NMe_2$-*o*)3@SBA-15. *Inorg. Chem*. **60**, 13122–13135 (2021).
23. Adiloğlu, Y.; Şahin, E.; Tutar, A. & Menzek, A. Cycloaddition Reactions of Benzonorbornadiene and Homonorbornadiene: New Isoxazoline and Pyridazine Derivatives. *J. Heterocyclic Chem*. **55**, 1917–1925 (2018).
24. Fan, M.; Zhou, W.; Jiang, Y. & Ma, D. CuI/Oxalamide Catalyzed Couplings of (Hetero)aryl Chlorides and Phenols for Diaryl Ether Formation. *Angew. Chem. Int. Ed*. **55**, 6211–6215 (2016).
25. Kaupp, G.; Perreten, J.; Leute, R. & Prinzbach, H. Photochemische Isomerisierung anellierter 7-Aza-norbornadiene. *Chem. Ber*. **103**, 2288–2301 (1970).
26. Matsushita, N.; Kashihara, M.; Formica, M. & Nakao, Y. Pd-Catalyzed Etherification of Nitroarenes. *Organometallics* **40**, 2209–2214 (2021).
27. Salanouve, E. et al. Tandem C–H Activation/Arylation Catalyzed by Low-Valent Iron Complexes with Bisiminopyridine Ligands. *Chem. Eur. J.* **20**, 4754‒4761 (2014).
28. Chen, Z.; Liang, J.; Yin, J.; Yu, G. & Liu, S. Alder-ene reaction of aryne with olefins. *Tetrahedron Letters*. **54**, 5785–5787 (2013).
29. Kuwano, R. & Kusano, H. Benzyl Protection of Phenols under Neutral Conditions: Palladium-Catalyzed Benzylations of Phenols. *Org. Lett.* **10**, 1978–1982 (2008).
30. Liu, Z.; Ma, Q.; Liu, Y. & Wang, Q. 4-(*N*,*N*-Dimethylamino)pyridine Hydrochloride as a Recyclable Catalyst for Acylation of Inert Alcohols: Substrate Scope and Reaction Mechanism. *Org. Lett.* **16**, 236–239 (2014).
31. Chun, S. & Chung, Y. K. Transition-Metal-Free Poly(thiazolium) Iodide/1,8-Diazabicyclo[5.4.0]undec-7-ene/Phenazine-Catalyzed Esterification of Aldehydes with Alcohols. *Org. Lett.* **19**, 3787–3790 (2017).
32. Sherly, P. B. et al. Amphiphilic Dendrimer as Reverse Micelle: Synthesis, Characterization and Application as Homogeneous Organocatalyst. *Tetrahedron* **75**, 130676–130688 (2019).
33. Xu, Z.; Yu, X.; Yang, D. & Li, C. Metal-free synthesis of 2-aminonaphthalenes by intramolecular transannulation of 1-sulfonyl-4-(2-alkenylphenyl)-1,2,3-triazoles. *Org. Biomol. Chem.* **15**, 3161–3164 (2017).
34. Clive, D. L. J. & Kang, S. Synthesis of Biaryls by Intramolecular Radical Transfer in Phosphinates. *J. Org. Chem.* **66**, 6083–6091 (2001).
35. Hajra, S. & Bar, S. Asymmetric synthesis of the dopamine D1 agonist, dihydrexidine. *Tetrahedron: Asymmetry.* **22**, 775–779 (2011).

36. Kubo, T. et al. A mild inter- and intramolecular amination of aryl halides with a combination of CuI and CsOAc. *Tetrahedron* **64**, 11230–11236 (2008).

37. Nathel, N. F.; Morrill, L. A.; Mayr, H. & Garg, N. K. Quantification of the Electrophilicity of Benzyne and Related Intermediates. *J. Am. Chem. Soc.* **138**, 10402–10405 (2016).

38. Frisch, M. J. et al. Gaussian 09, revision D.01; Gaussian, Inc.: Wallingford, CT, 2013.

39. Zhao, Y. & Truhlar, D. G. The M06 suite of density functionals for main group thermochemistry, thermochemical kinetics, noncovalent interactions, excited states, and transition elements: two new functionals and systematic testing of four M06 functionals and 12 other functionals. *Theor. Chem. Acc.* **119**, 525 (2008).
